# Synthetic and Mechanistic Studies into the Reductive Functionalization of Nitro Compounds Catalyzed by an Iron(salen) Complex

**DOI:** 10.1021/jacs.4c02797

**Published:** 2024-07-12

**Authors:** Emily Pocock, Martin Diefenbach, Thomas M. Hood, Michael Nunn, Emma Richards, Vera Krewald, Ruth L. Webster

**Affiliations:** † Department of Chemistry, University of Bath, Claverton Down, Bath BA2 7AY, U.K.; ‡ Department of Chemistry, TU Darmstadt, Darmstadt 64287, Germany; § Early Chemical Development, Pharmaceutical Sciences, Biopharmaceuticals R&D, AstraZeneca, Macclesfield SK10 2NA, U.K.; ∥ School of Chemistry, Cardiff University, Main Building, Park Place, Cardiff CF10 3AT, U.K.; ⊥ Yusuf Hamied Department of Chemistry, University of Cambridge, Lensfield Road, Cambridge CB2 1EW, U.K.

## Abstract

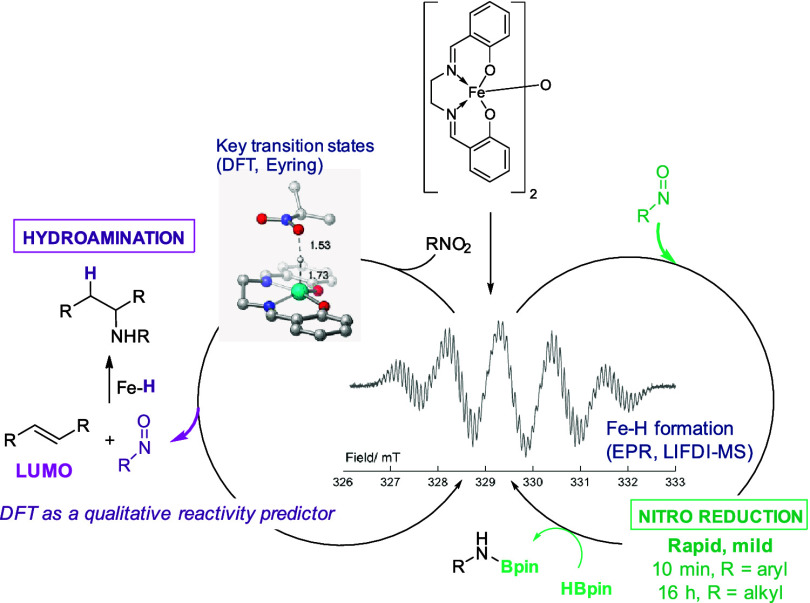

We report on the use of a simple, bench-stable [Fe(salen)_2_]-μ-oxo precatalyst in the reduction of nitro compounds. The reaction proceeds at room temperature across a range of substrates, including nitro aromatics and aliphatics. By changing the reducing agent from pinacol borane (HBpin) to phenyl silane (H_3_SiPh), we can chemoselectively reduce nitro compounds while retaining carbonyl functionality. Our mechanistic studies, which include kinetics, electron paramagnetic resonance (EPR), mass spectrometry, and quantum chemistry, indicate the presence of a nitroso intermediate and the generation of an on-cycle iron hydride as a key catalytic intermediate. Based on this mechanistic insight, we were able to extend the chemistry to hydroamination and identified a simple substrate feature (alkene lowest unoccupied molecular orbital (LUMO) energy) that could be used to predict which alkenes would result in productive catalysis.

## Introduction

**Scheme 1:**
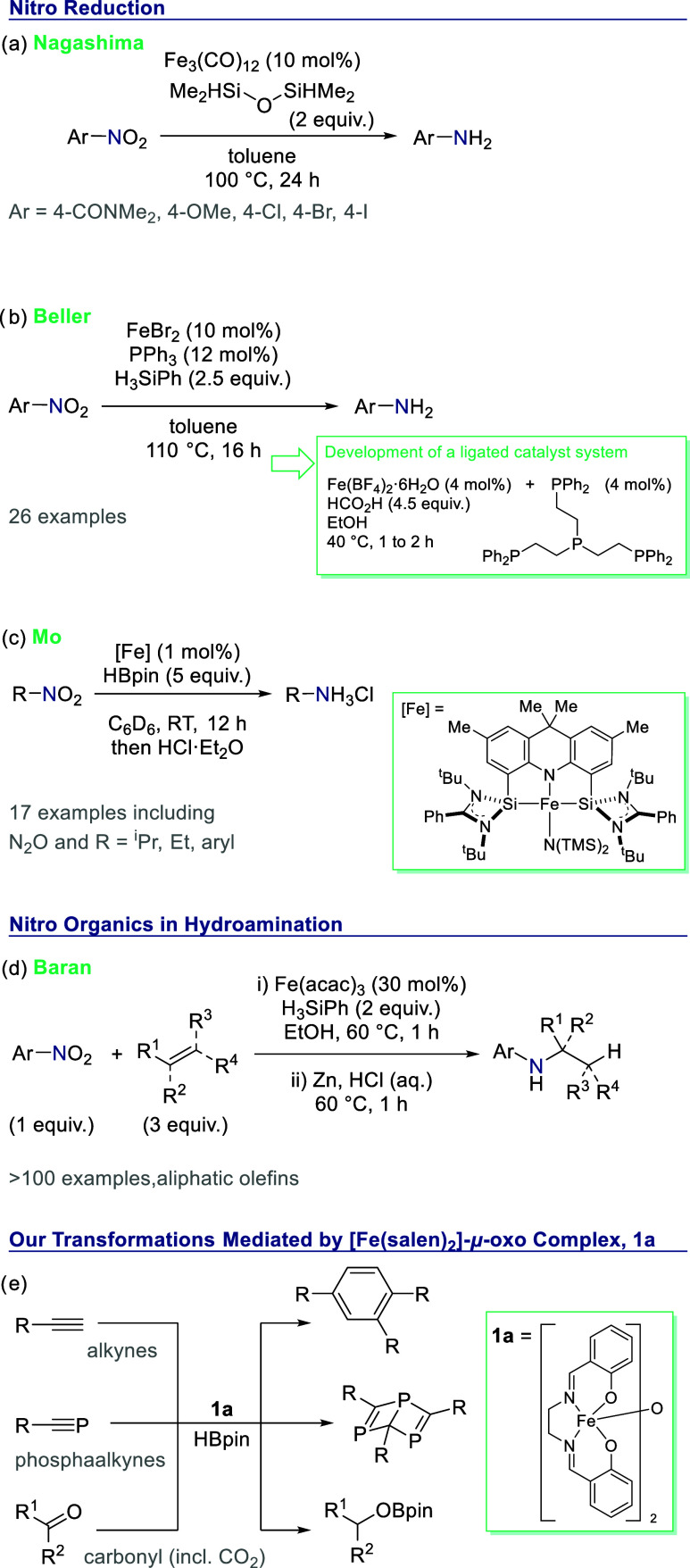
Previous Work on Iron-Catalyzed Nitro Reduction (a) Early work from Nagashima using an iron carbonyl compound, followed by (b) research from Beller using FeBr_2_, which then led to a phosphine-ligated system, and (c) more recently Mo has used an iron–silylene precatalyst. Intercepting the nitro reduction process allowed (d) seminal studies from Baran into iron-catalyzed hydroamination. (e) Our previous studies using precatalyst **1a** and HBpin have included cyclotrimerization and carbonyl reduction.

Traditionally, the reduction of a nitro moiety to its corresponding primary amine is carried out under harsh conditions (for example, a metal with concentrated acid at high temperature),^1^ and although an iron(0)/acetic acid route was reported as early as 1854,^2,3^ milder methods have since been established.^4^ Pertinent to this homogeneous catalysis work is that simple iron salts have been shown to catalyze the reduction of nitro aromatics,^5,6^ for example, Nagashima’s use of Fe_3_(CO)_12_ (Scheme 1a),^7^ Beller’s use of FeBr_2_ (Scheme 1b),^8^ and Fe(acac)_3_ has been employed by Lemaire and co-workers,^9,10^ but these reactions often require forcing conditions. A range of ligated iron complexes have also been employed with silanes^11−15^ or alternative hydride/hydrogen donors^16−25^ as reducing agents. More recently still, Mo reported a well-defined molecular iron catalyst capable of reducing nitro compounds under mild conditions.^26^ In this case, it is believed that the cooperative nature of the Fe–Si bond in the catalyst is necessary to facilitate these reductions (Scheme 1c).

Baran and co-workers’ seminal study on *in situ* hydroamination (HA) of olefins using nitro aromatics as the nitrogen source (Scheme 1d)^27^ has led to several analogous iron-catalyzed studies featuring both intra-^28−35^ and intermolecular^11,28^ HA. Baran’s work exemplifies the capacity of this transformation through the report of substituent-diverse synthesis of secondary amines from a range of nitro aromatics. There are some limitations in the substrate scope: free thiophenol and phenol-nitros are not tolerated and the olefin coupling partner must contain aliphatic substituents. On the other hand, Wang and co-workers developed an iron(Cp*) system that operates across a range of styrenes.^28^ However, it is important to stress how challenging this transformation is based on the postulated mechanism: iron-catalyzed partial reduction of the nitro group to a nitroso is necessary, which then must be trapped by an alkyl radical formed from a hydrogen atom transfer (HAT) event that is mediated by the same iron species. Therefore, there is the need to balance high activity while avoiding over-reduction (i.e., reduction of the nitro to an amine) and runaway radical reactions (i.e., alkene polymerization). Thus, iron-mediated intermolecular HA from nitro compounds remains a challenge.

**Scheme 2:**
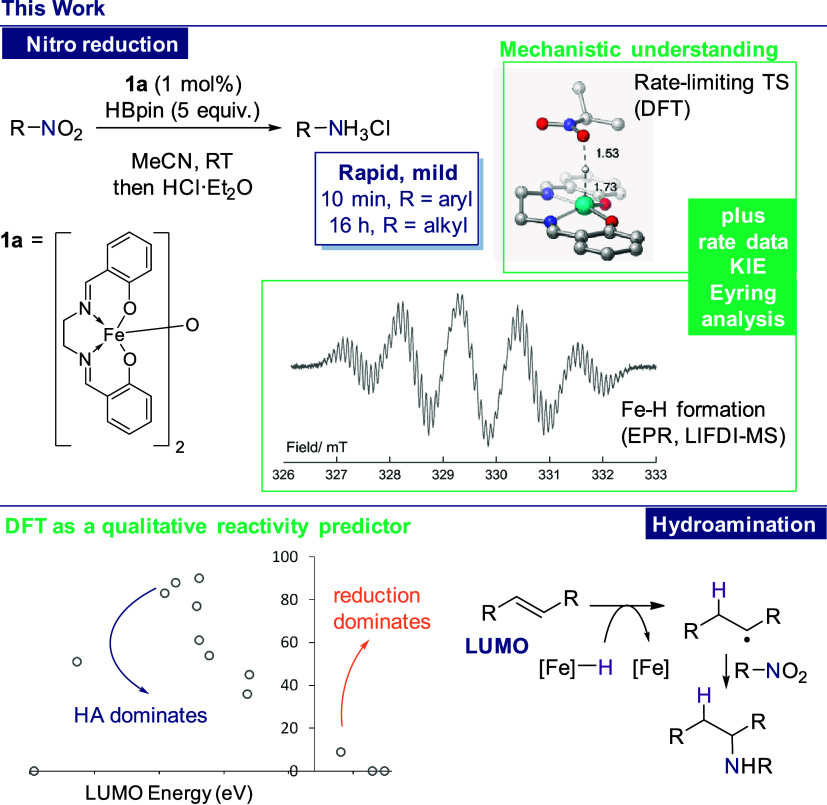
Development of a Mild Nitro Reduction Protocol Using a Simple, Air-Stable Precatalyst in This Work Our mechanistic studies indicate the presence of a nitroso intermediate, which is then exploited in hydroamination.

Previous work in our group has focused on the use of iron(salen) complexes, and these have proven to be highly active in hydrophosphination,^36,37^ hydroboration,^38^ and organo-alkyne^39,40^ and phosphaalkyne^41^ cyclotrimerization (Scheme 1e). These complexes benefit from being air-stable with a scalable synthesis. They are also highly tunable (via the ligand)^42−46^ and thus more soluble, and potentially more active and selective, than iron salts or *in situ* formed complexes. Our previous studies have indicated that we may be able to access a particularly “hot” (i.e., reactive) iron(salen)-hydride species,^40^ and we postulated that we should be able to sequester this for fast and facile reduction chemistry. The short-lived nature of the iron hydride means that an appropriate target substrate is needed. To this end, we found that nitro compounds are an ideal substrate for reduction using Fe(salen)-μ-oxo (**1a**) as the precatalyst and HBpin as the reductant. We herein present the results of our synthetic scope of nitro reduction, associated mechanistic studies, and the extension to HA which is driven by predictive density functional theory (DFT) studies (Scheme 2).

## Results and Discussion

### Nitro Reduction Scope

**Figure 1:**
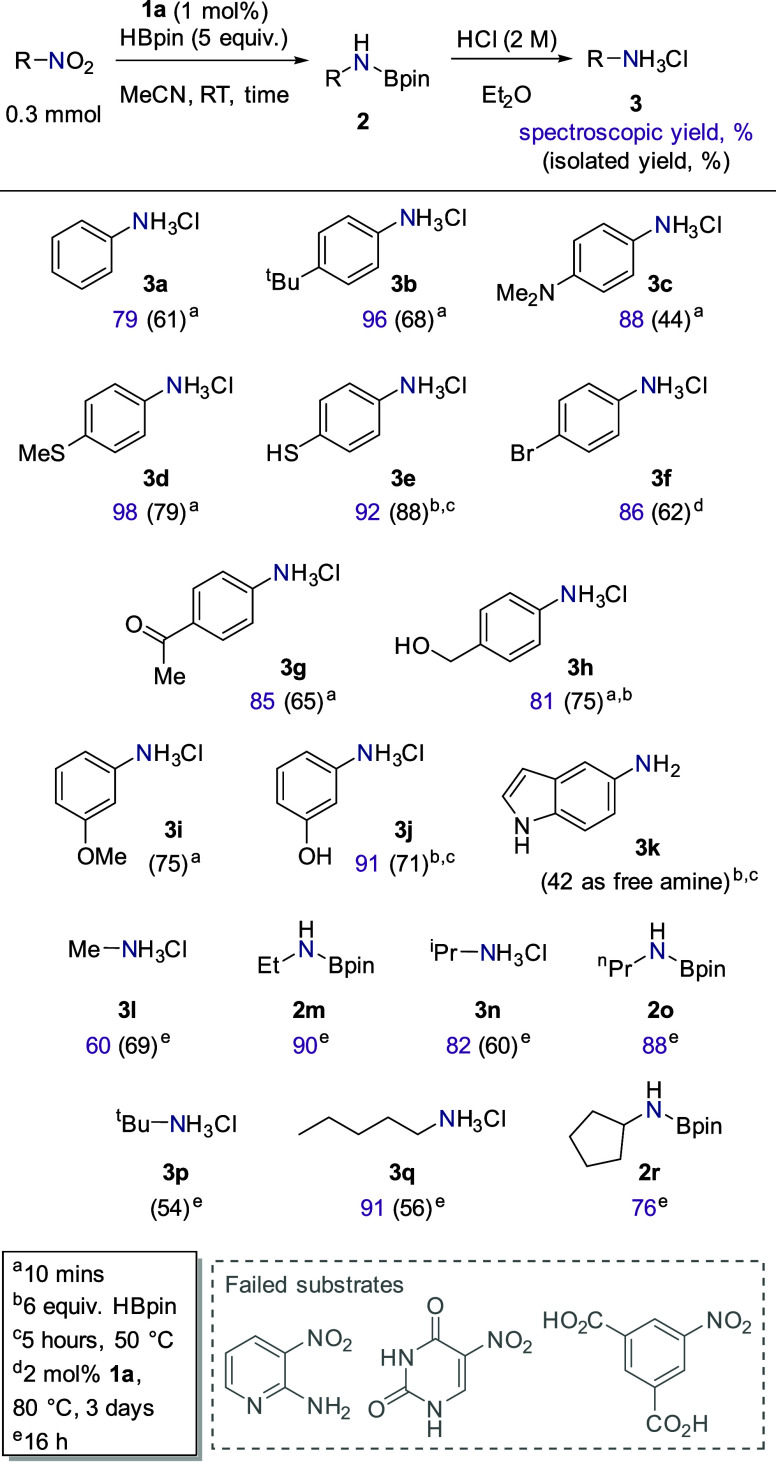
Substrate scope for the reduction of nitro compounds using HBpin and precatalyst **1a**.

Following a short optimization study (see the Supporting Information), treatment of a range of nitro compounds with 1 mol % precatalyst **1a** and 5 equiv of HBpin in acetonitrile results in facile reduction across a range of substrates in only 10 min at room temperature. No reaction is observed in the absence of **1a**. Reduction does take place when H_3_SiPh (2 equiv) is employed, but the reaction requires heating to 50 °C for 16 h (see the Supporting Information). Purification by column chromatography results in considerable loss of the desired primary amine. Instead, formation of the ammonium salt by treatment with HCl in Et_2_O is an operationally simple method of isolation (Figure 1). Generally, good to excellent spectroscopic yields are achieved for nitro aromatics bearing both electron-donating and -withdrawing substituents, with good functional group tolerance (**3a**–**3k**). Most notably, substrates bearing “free” thiophenol and phenol moieties (generating products **3e** and **3j**, respectively) are extremely well tolerated under our conditions. Such protic substrates are typically not well tolerated under nitro reduction conditions (or are, at least, unreported). In our case, the addition of an additional equivalent of HBpin (totaling 6 equiv) facilitates an *in situ* dehydrocoupling^47^ of the phenol moiety (see the Supporting Information for NMR analysis of **3j**, which contains N-Bpin and O-Bpin protected functionality), thus preventing any potential catalytic poisoning. **3h** is derived from the aldehyde and requires an extra equivalent of HBpin in order to facilitate clean reduction of the aldehyde and the nitro group (*vide infra*). For comparison, Beller’s work using 4.5 equiv formic acid at 40 °C for 1 h operates well across a range of halogenated substrates along with vinyl-, methoxy-, and methylsulfane-substituted nitro aromatics,^23^ while Mo has demonstrated that nitro aromatics can be cleanly reduced using HBpin without adversely affecting heteroaromatic and cyano functionality.^26^ Our substrate scope is not exhaustive, but we can state that pyridyl-, pyrimidinedione-, and carboxylic acid-containing substrates do not react cleanly under our standard reaction conditions.

The iron-catalyzed reduction of nitro aliphatics is surprisingly sparse in the literature. Mo and co-workers demonstrated the competent reduction of nitrous oxide as well as nitroethane and 2-nitropropane,^26^ but other studies tend to omit these more challenging substrates. Gratifyingly, our conditions are readily applied to a small number of aliphatic nitro compounds (Figure 1, generating salt products **3l**, **3n**, **3p**, **3q** and Bpin adducts to **2m**, **2o**, and **2r**), including nitromethane (to form **3l**). The reaction proceeds smoothly at room temperature, but the reactions have been left for 16 h to ensure they go to completion. This methodology serves as an attractive tool for the *in situ* generation of anhydrous methylamine.

**Scheme 3:**
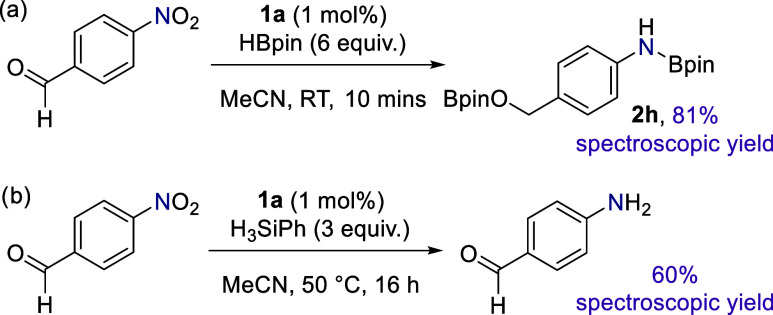
Change in Reducing Agent from HBpin (a) to H_3_SiPh (b) Allows for Chemoselective Reduction

Owing to the high levels of catalytic activity we observe, 4-nitrobenzaldehyde undergoes reduction at both the nitro and carbonyl groups (Figure 1 and Scheme 3a). This reduction proceeds via hydroboration of the aldehyde, which we have previously demonstrated occurs readily in the presence of precatalyst **1a** under similar reaction conditions.^38^ We hypothesized that changing the highly active reductant, HBpin, for a less active one, e.g. H_3_SiPh, should allow us to chemoselectively reduce the nitro group and leave the aldehyde intact. This is the case: H_3_SiPh allows the smooth reduction of the nitro moiety without considerable reduction of the aldehyde unit (Scheme 3b). The reduced yield for this reaction is due to the formation of an unknown polymeric precipitate but boasts greater overall chemoselectivity.

### Mechanistic Studies

**Figure 2:**
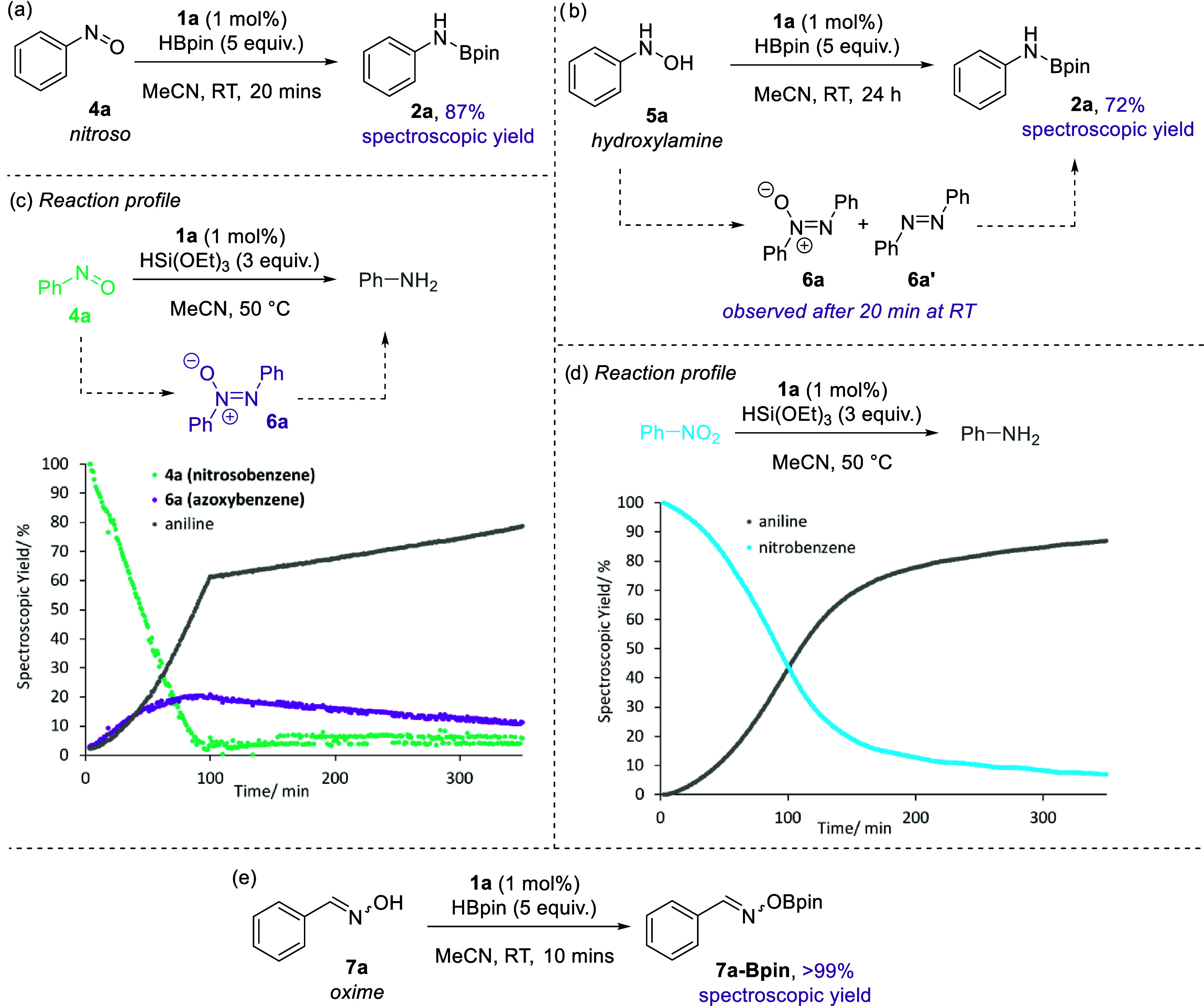
Investigation into likely intermediates formed during nitro reduction catalyzed by **1a**. (a) Reaction of a nitroso compound under catalysis conditions; (b) a hydroxylamine proceeds via intermediates **6a** and **6a**′ and over a longer time period than standard catalysis; (c) reaction profile showing the conversion of a nitroso in the presence of a silane, via **6a** (data collected every 60 s); (d) nitrobenzene converts cleanly to aniline in the presence of silane (data collected every 60 s); (e) a hydoxylamine is an unlikely intermediate as dehydrocoupling is observed.

To begin our mechanistic investigation, we identified a range of possible N–O intermediates and exposed them to our catalytic conditions. A nitrosoarene, an *N*-alkoxyaniline, and an oxime (**4a**, **5a**, and **7a**, respectively, Figure 2) were subjected to the reduction conditions to probe their plausibility as intermediates. Nitrosobenzene (**4a**, Figure 2a) forms **2a** in an 87% spectroscopic yield under standard reaction conditions. **4a** can be reduced to **2a** using HBpin in the absence of **1a** but requires 18 h at 80 °C to do so.

*N*-phenylhydroxylamine (**5a**) is reduced to **2a** in a 72% spectroscopic yield over an extended time period (Figure 2b). Interestingly, *in situ* NMR monitoring shows an appreciable buildup of azoxybenzene (**6a**) and azobenzene (**6a′**) after 20 min at room temperature (RT). This clearly indicates a switch in mechanism, one where these N–N coupled products are intermediates. These intermediates are not observed during *in situ* NMR monitoring of the reduction of nitrobenzene, and coupled with the extended time scale of this process indicates that hydroxylamines, although reducible, are not intermediates in our nitro reduction catalysis. This trend is also observed when HSi(OEt)_3_ is used as a reductant (see the Supporting Information).

Monitoring the reaction by NMR spectroscopy using nitrosobenzene (**4a**), rather than nitrobenzene, is challenging due to the fast nature of the reaction. To gain insight and useful reaction profiles, HSi(OEt)_3_ was employed as the reductant. Based on the reaction profiles obtained (Figure 2c), it can be further confirmed that nitrosobenzene is likely to be a short-lived intermediate and does not build up in the reaction. This is because using **4a** as a drop-in replacement for nitrobenzene results in its zeroth-order decay and rapid formation of azoxybenzene (**6a**, approximately 20% after 95 min), which then depletes over the remainder of the reaction period as aniline continues to form. In contrast, under identical reaction conditions over the same time period using nitrobenzene (Figure 2d), we see clean conversion to aniline with no intermediates **4a** or **6a** being observed. The reduction of nitro compounds is clean and only (pinB)_2_O (δ_11B_ 21 ppm) and dihydrogen (δ_1H_ 4.57 ppm) are observed. There is a slower induction-type phase to catalysis that is not observed when starting from **4a**. This is likely linked to the fact that the silane is not as effective at converting the precatalyst into an active catalyst. Unfortunately, the difference in reactivity of **4a** compared to nitrobenzene also indicates that we cannot deconvolute the proposed interlinked catalytic cycles (*vide infra*).

However, the time scale of the reduction of **4a** to aniline via **6a** using HSi(OEt)_3_ (Figure 2c) is in line with silane-mediated reduction of nitrobenzene to aniline (Figure 2d), and therefore, we feel a nitroso intermediate is likely.

In contrast, oxime **7a** quantitatively dehydrocouples with HBpin to generate **7a-Bpin** and no further reduction can be facilitated (Figure 2e). Oximes can be ruled out as intermediates.

**Figure 3:**
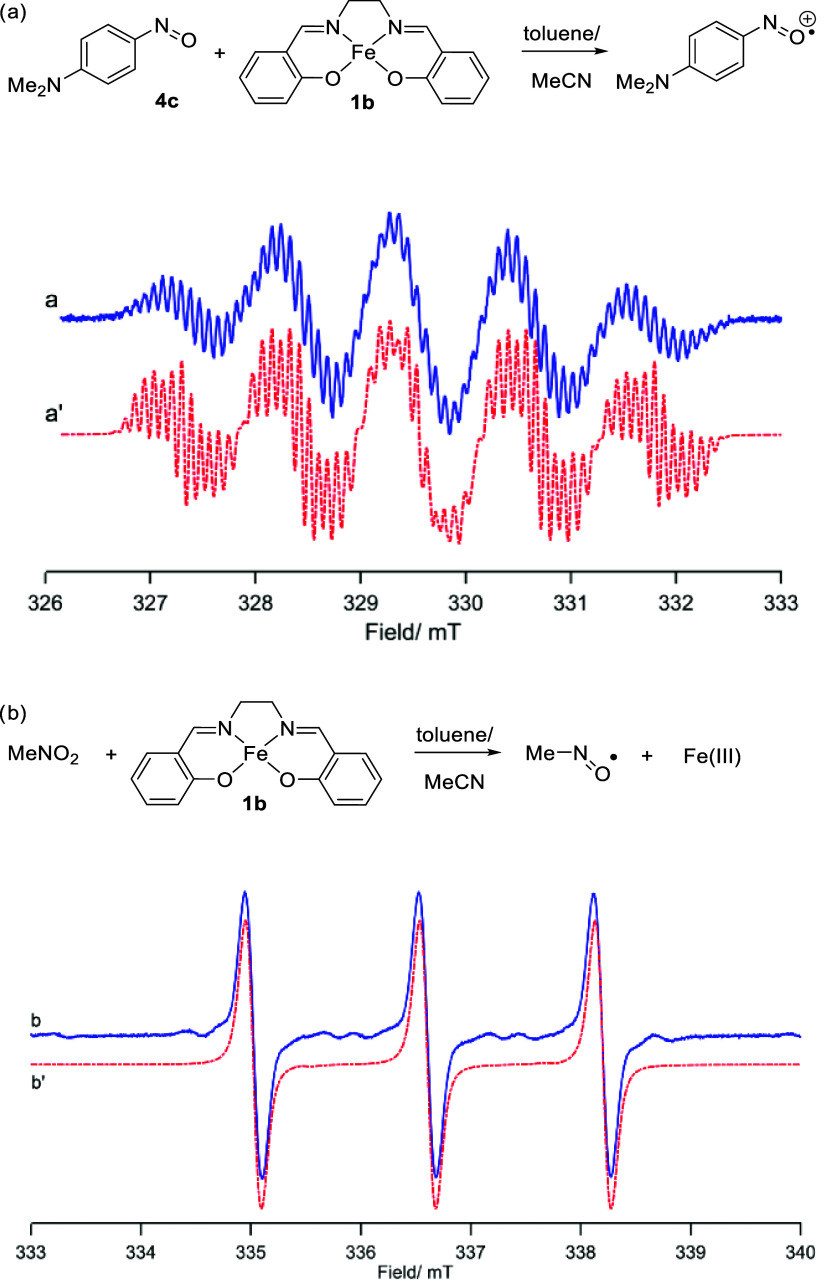
(a) **1b** and **4c** generate a nitroso radical cation, whereas only an EPR-silent spectrum is observed when **1a** is reacted with HBpin and **4c**; (b) nitromethane and **1b** generate a nitroxide radical. Corresponding simulations are presented in a′ and b′ (see the Supporting Information for details).

The related precatalyst [Fe(II)salen] (**1b**, high spin, *S* = 2), which we could envision forms from reduction of **1a**, is active in catalysis. However, based on our previous work, reaction of **1a** to form [Fe(III)(H)salen] (**1c**) and elimination of H_2_ from [Fe(H)salen]_2_ to form **1b**(40) is slower than the reduction of a nitro compound, so this process is unlikely. Electron paramagnetic resonance (EPR) studies also show that **1a** and **1b** behave very differently, indicating that **1b** is not involved in the catalytic cycle.^48,49^ It is important to note that the spectrum of **1a** (120 K, X-band) is characteristic of Fe(III) (see the Supporting Information), while **1b** (*S* = 2) is not observed by EPR (140 K, X-band) and is thus EPR-silent. Reaction of **1a**, **4c**, and HBpin affords an EPR-silent spectrum at 298 K (i.e. no organo radicals are detected), whereas reaction of **1b** with **4c** affords a nitroso radical cation, observed at 298 K (Figure 3a). Oxidation of **1b** to Fe(III) is also observed, as evidenced by the characteristic signal at 140 K. Unlike the results with **1a** in the presence of the reducing agent, there is clear evidence of an organic radical species in the 298 K spectrum.

That organo radicals are not involved in our nitro reduction catalysis is further supported through a series of synthetic trapping experiments. Addition of 1 equiv TEMPO to 2-nitropropane, HBpin (5 equiv), and **1a** (1 mol %) affords **2n** (*i*PrNHBpin) in a 60% spectroscopic yield, with the reaction going to a 71% spectroscopic yield after another equivalent of HBpin is added (in this case, we believe TEMPO is interfering with reactivity by reacting with the B–H bond). Adding 1 equiv of chloromethylcyclopropane to 2-nitropropane, HBpin (5 equiv), and **1a** (1 mol %) affords a 76% spectroscopic yield of **2n**, compared to 80% observed in the absence of a trapping agent.^50^

When nitrobenzene is employed in an *in situ* EPR study with **1a** and HBpin, the reaction is EPR-silent at 298 K (again, no organo radicals are observed). In contrast, the production of a nitroxide radical is observed when complex **1b** is reacted with nitromethane in the absence of HBpin, evidenced by a 1:1:1 triplet signal originating from the ^14^N nucleus (Figure 3b).

The *in situ* monitoring of the reaction of **1a** with HBpin by liquid injection field desorption ionization mass spectrometry (LIFDI-MS) indicates the presence of a short-lived iron(III) hydride (**1c**; [Fe(III)(H)salen]). No further intermediates can be identified unambiguously by LIFDI-MS, and there is no parent ion associated with **1b** (see the Supporting Information), adding further weight to the assertion that this species is not present in catalysis. Electrospray ionization liquid chromatography mass spectrometry (ESI LCMS) has also been used to identify the analogous deuteride [Fe(III)(D)salen] (see the Supporting Information).

The likely presence of **1c** is further supported through EPR studies, where reaction of **1a** and HBpin or H_3_SiPh in the presence of a spin-trapping agent (DMPO, 5,5-dimethyl-pyrroline *N*-oxide, or PBN, *N*-tertiary-butylnitrone) results in hydride trapping (see the Supporting Information).

Electronic structure calculations predict **1b** as a high-spin species using Hartree–Fock and Kohn–Sham DFT-based coupled-cluster theory in the DLPNO–CCSD(T) variant in agreement with our previous work (see the Supporting Information for details).^40^ For the hydride species **1c**, the situation is less clear-cut: HF-DLPNO–CCSD(T) predicts a high-spin ground state with an energetically higher-lying low-spin configuration (Δ*G* = 5.2 kcal mol^–1^, Table S9), whereas KS-DLPNO–CCSD(T) predicts the reverse situation with the low-spin state stabilized over the high-spin state (Δ*G* = 3.6 kcal mol^–1^). The implications for the catalytic cycle are discussed below and in the Supporting Information.

To obtain kinetics data, we employed *i*PrNO_2_ and HBpin to allow adequate data collection. However, these reactions suffered from high levels of paramagnetism in the first 10–20 min, therefore initial rate data could not be collected. However, by using HSi(OEt)_3_ as the reducing agent and measuring the initial rates of reduction of PhNO_2_, the reaction is found to be approximately half-order in precatalyst **1a**. This is consistent with the formation of a monomeric active species, e.g., mononuclear iron hydride, **1c**. Its sensitivity is such that we have not been able to isolate **1c** and employ this in catalysis. Monitoring the overall reaction using HBpin as the reducing agent, where almost instantaneous precatalyst activation takes place, the reaction appears to be first order in on-cycle iron, indicating that the mononuclear species does not undergo dimerization during catalysis. The reaction is first order in 2-nitropropane. At catalytically-relevant regimes (4.5, 5.0, and 5.5 equiv HBpin), the reaction is zero order in HBpin, consistent with the reductant not being involved in the rate-limiting step. At higher loadings of HBpin (6.75, 7.5, and 10 equiv), the data do not fit zero- or first-order VTNA^51^ or integrated rate law plots. At this point, we assume that a change in mechanism and/or rate-limiting step is taking place.

When DBpin is used, a KIE of 1.13 ± 0.09 was measured. This indicates that HBpin is unlikely to be involved in the rate-limiting step. However, although large primary KIEs have been reported for B–H bond cleavage,^52^ small isotope effects have also been observed in hydride transfer reactions.^52−54^ All deuterium incorporation is limited to the amine protons.

Eyring analysis using *k*_obs_ data from the reduction of 2-nitropropane with 1 mol % **1a** and 5 HBpin over a range of temperatures gives Δ*G*^‡^ = 22.4 ± 4.0 kcal mol^–1^, Δ*H*^‡^ = 18.3 ± 1.1 kcal mol^–1^, and Δ*S*^‡^ = −14.0 ± 3.8 cal mol^–1^ K^–1^. These data are consistent with an early transition state, which is likely to be associative in nature, and a moderate barrier for the rate-limiting transition state, which is consistent with the facile (exergonic) nature of these nitro reduction reactions.

**Figure 4:**
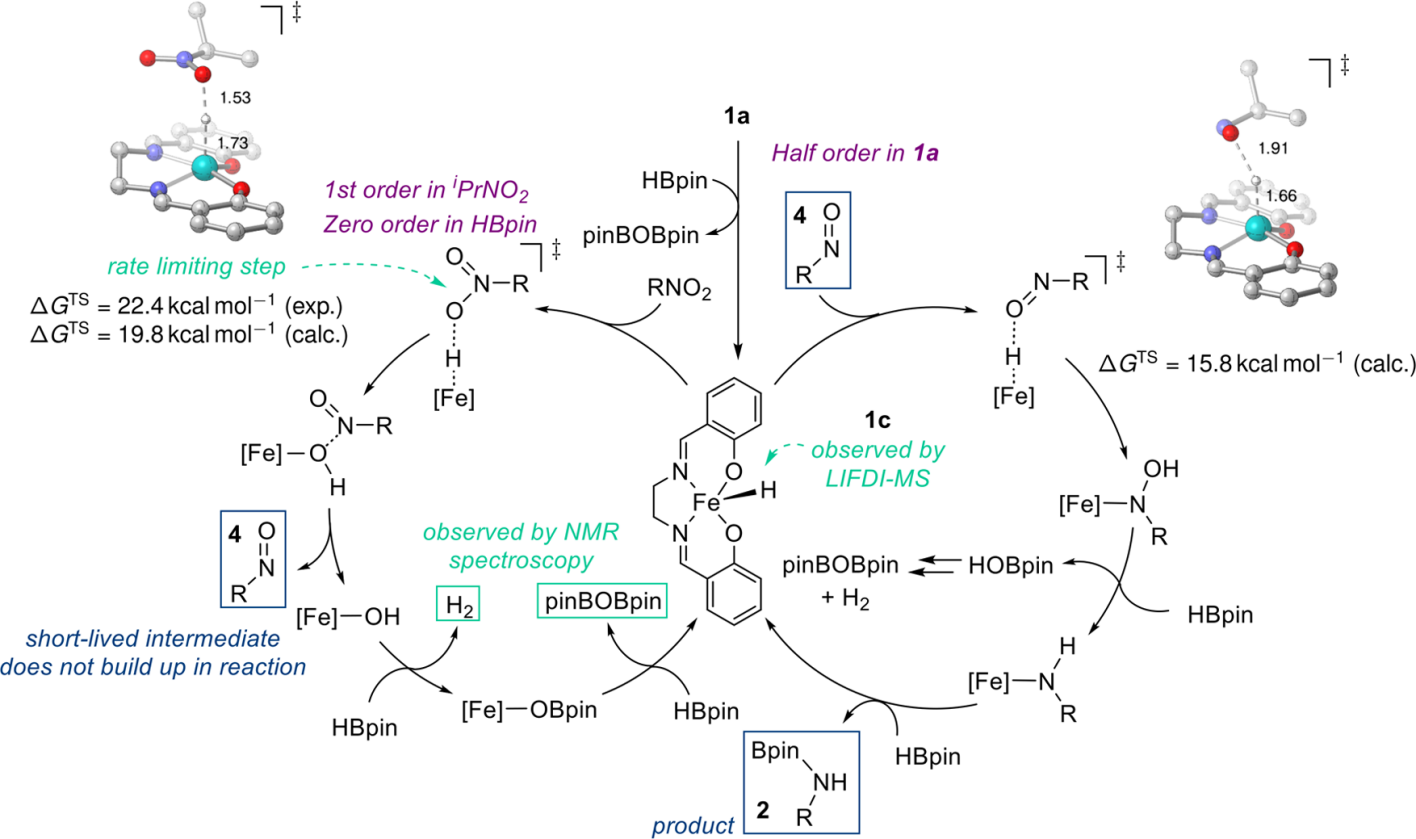
Postulated catalytic cycle proceeding via iron(III) hydride intermediate **1c**.

Based on the experimental data collected, we postulate that the reaction proceeds via the following interlinked catalytic cycles (Figure 4). Precatalyst **1a** is activated by HBpin (or silane), resulting in the iron(III) hydride species **1c**. This is evidenced by EPR showing that the reducing agents both generate a hydride that can be spin-trapped and LIFDI-MS data which show the presence of **1c** in a catalytic run. **1c** interacts with the nitro reactant, and then hydride transfer takes place. This is the rate-limiting step and has an experimentally determined barrier of 22.4 ± 4.0 kcal mol^–1^. An iron(III) hydroxide is then generated, along with release of the nitroso intermediate **4**. The hydroxide intermediate reacts with 2 equiv HBpin to ultimately regenerate **1c**, releasing H_2_ and pinBOBpin en route (the latter are observed by ^1^H and ^11^B NMR spectroscopy). **1c** further reacts with the nitroso intermediate, which is a very short-lived species. Another insertion reaction takes place, along with H_2_ and pinBOBpin release, and the generation of an iron(III) amido species. The final step in the interlinked cycles is reaction of the iron(III) amido with HBpin to release product (**2**) and regenerate **1c**. The complexities of this interlinked catalytic system have likely imparted a level of complexity to the kinetic data that we cannot deconvolute using standard VTNA or integrated rate law methods.

For the *in silico* evaluation of the interlinked catalytic cycles, the small energy gap between the high-spin and low-spin configurations of **1c** raises the question whether multistate reactivity will play a role. We find that all iron-containing intermediates have high-spin ground states at the density functional theory level PBE0-D3/def2-TZVP//PBE-D3/def2-SVP, which was benchmarked against HF-DLPNO–CCSD(T) calculations for **1b**, **1c**, and the hydroxy intermediate (see the Supporting Information, Table S9). The catalytic sequence is therefore computed on the high-spin surface throughout.

The interlinked catalytic cycles were evaluated computationally for the substrates 2-nitropropane and nitrobenzene; we discuss here explicitly the 2-nitropropane case. The rate-limiting transition state for hydride transfer from **1c** to 2-nitropropane obtained by DFT is fully consistent with the experimentally derived Eyring analysis. The barrier for this step is calculated as Δ*G*^‡^ = 19.8 kcal mol^–1^, equivalent to *t*_1/2_ ≈ 27 s. This is close to the computed BDFE for the iron-hydride bond of 18.7 kcal mol^–1^ at the same level of theory. As the next intermediate in the catalytic cycle, a nitroso–iron hydroxide complex is formed (Δ_r_*G* = −9.5 kcal mol^–1^). The barrier for liberating the short-lived nitroso intermediate is minute, Δ*G*^‡^ = 2.5 kcal mol^–1^ (see the Supporting Information). The catalyst is thus rendered as an iron(III) hydroxide, which again is most stable in its high-spin form with respect to the energetically closest spin state, in this case the quartet (Δ*G*^HF-DLPNO–CCSD(T)^ = 23.8 kcal mol^–1^). Formation of the iron–OBpin species from the iron hydroxide is further downhill at Δ_r_*G* = −61.3 kcal mol^–1^. The overall reaction of this first half-cycle, i.e., the reduction of the nitro to the nitroso species **4**, is exergonic by Δ_r_*G* = −59.6 kcal mol^–1^.

In the second half-cycle, the initial barrier for hydride transfer from **1c** to the nitroso species is found at Δ*G*^‡^ = 15.8 kcal mol^–1^, translating to *t*_1/2_ ≈ 43 ms. This is distinctly lower than that of the rate-limiting step for the initial nitro reduction, which is clearly in agreement with the experimental observation that **4** does not appreciably build up in the reaction. The *N*-hydroxy intermediate lies at Δ_r_*G* = −37.4 kcal mol^–1^. Further reduction to the iron amido species is again strongly exergonic (Δ_r_*G* = −108.3 kcal mol^–1^). For the net reduction reaction of the nitroso intermediate to the product **2** in the second half-cycle, the Gibbs energy amounts to Δ_r_*G* = −117.4 kcal mol^–1^.

For nitrobenzene, the same general picture emerges, though with a lower rate-limiting first reduction step of Δ*G*^‡^ = 15.6 kcal mol^–1^ (see the Supporting Information). This would correspond to an approximate acceleration in *t*_1/2_ by 3 orders of magnitude, which is consistent with the experimental finding of a much more rapid reactivity for nitrobenzene than that for 2-nitropropane.

For completeness, we note that a lower-energy low-spin form of the rate-limiting TS can be found with DFT, which suffers, however, from significant spin contamination. HF-DLPNO–CCSD(T1) calculations report a less-stable low-spin than high-spin state, albeit with a large *T*_1_ diagnostic of 0.08 for the doublet (see Table S9). This arises from single excitations between almost degenerate molecular orbitals delocalized between iron and the NO moiety of the substrate (see Figure S335). Such a scenario was discussed recently for a singlet biradicaloid intermediate en route to Pd(II) nitrene reactivity.^55^ Canonical HF-based coupled-cluster theory was found to suffer dramatic errors, whereas with more delocalized Kohn–Sham reference orbitals, the CCSD(T) results improved substantially. We observe a similar trend for the low-spin TS, where KS reference orbitals lower the *T*_1_ diagnostic to reasonable values below 0.04 (see Table S9). With KS-DLPNO–CCSD(T1), the low-spin TS is lower in energy than its HS congener.

Importantly though, regardless of the spin state, the initial barrier for nitro reduction predicted with KS-DLPNO–CCSD(T1) agrees with the experimentally observed rate-determining step (see Table S8). It is therefore not possible to discriminate between the two spin-state options by comparison with experiment. We emphasize that with both types of CC description, the TS of the first half-cycle is always higher in energy than that of the second half-cycle (see Figure S336). The only calculations where this would be predicted incorrectly are those using DFT, where the low-spin TS is suffering from artefacts due to spin contamination (see Figure S334, Table S8). If the route via the low-spin TS were the correct description, this would render the rate-determining step of the second half-cycle as the overall limiting step, in conflict with the experimental observations. We furthermore note that any purported intermediate-spin or low-spin transition structures would have to undergo a spin–orbit coupled spin-crossover twice between the corresponding reactant and product intermediate structures. An estimate of such spin-crossing probabilities is beyond the scope of this work. However, given the experimentally observed rapid reactivity and the high-spin description being consistent with the experimental information, we surmise that the high-spin surface will be more competitive during the catalytic cycle than a multistate scenario with several spin-crossover impediments.

### Hydroamination

Upon developing a greater understanding of the mechanism of nitro reduction, we believed that our catalytic system would be primed for an *in situ* HA of olefins, similar to that of Baran’s study. This reaction does not operate via a standard HA protocol, where N–H adds across a C–C double or triple bond; the active iron hydride should facilitate the partial reduction of the nitro moiety (forming a nitroso intermediate)^56,57^ and hydrogen atom transfer (HAT)^58^ to the unsaturated coupling partner. The transformation relies on a nitroso intermediate reacting with an alkyl radical, followed by a series of reduction steps.^59^

We initiated our HA studies by reacting 4-*tert*-butylnitrobenzene with HBpin in the presence of allylbenzene and **1a** but found that only the aniline product (**2b**, 4-*t*Bu-C_6_H_4_-NHBpin) was formed. From our initial reaction, it was clear that HBpin is too active in the reduction process; therefore, interception of a nitroso intermediate is not possible. However, use of H_3_SiPh should slow the entire catalytic process down and thus facilitate HA. Reaction of **1a**, 4-*tert*-butylnitrobenzene, allylbenzene, and H_3_SiPh results in a small amount of HA product being observed by ^1^H NMR spectroscopy. However, even after several rounds of optimization (see the the Supporting Information) and screening two other substrates with differing electronic properties, HA is not able to outcompete reduction (to form 4-*tert*-butylaniline) or the double addition product formation (**9**), Table 1. Although not atom-economic, these double functionalization products (of the form **9**) can be reduced using Zn/HCl in hot MeOH to cleave the N–O bond and generate the HA product, e.g., **8** from **9**. It quickly became apparent that a traditional screening approach would not be an efficient way to improve this catalysis, and we hypothesized that the issue was more fundamental, namely, that there was a mismatch between the singly occupied molecular orbital (SOMO) of the iron hydride **1c** and the lowest unoccupied molecular orbital (LUMO) of the alkene being employed. Using density functional theory (PBE0-D3/def2-TZVP//PBE-D3/def2-SVP including an implicit solvation model for acetonitrile), we calculated the energy of the alkene LUMO (see Table 1). This clearly shows a range of energies, from very positive (1-methylcyclohexene, 0.54 eV) which gives the aniline almost exclusively, through to very negative (α-methylstyrene, −1.16 eV) which gives the double functionalization product (**9**) almost exclusively. At an intermediate energy (allylbenzene), we form a mixture of HA product (**8**) and aniline.

**Table 1:**
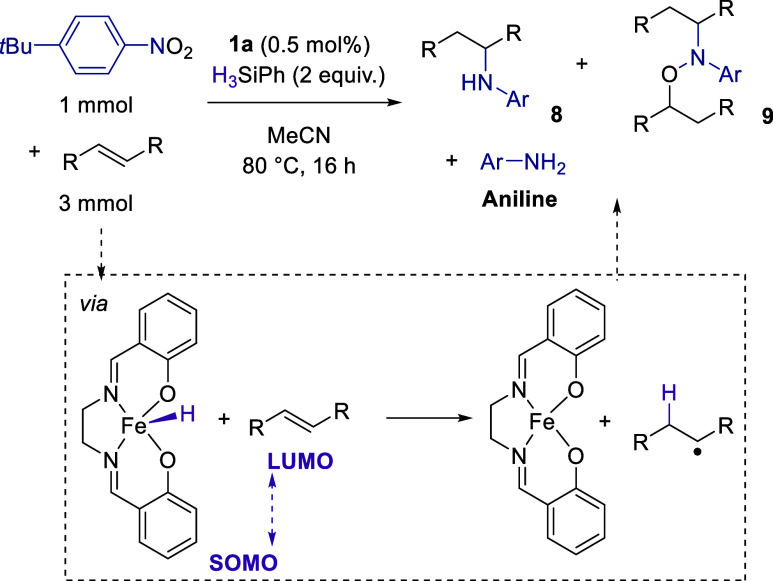

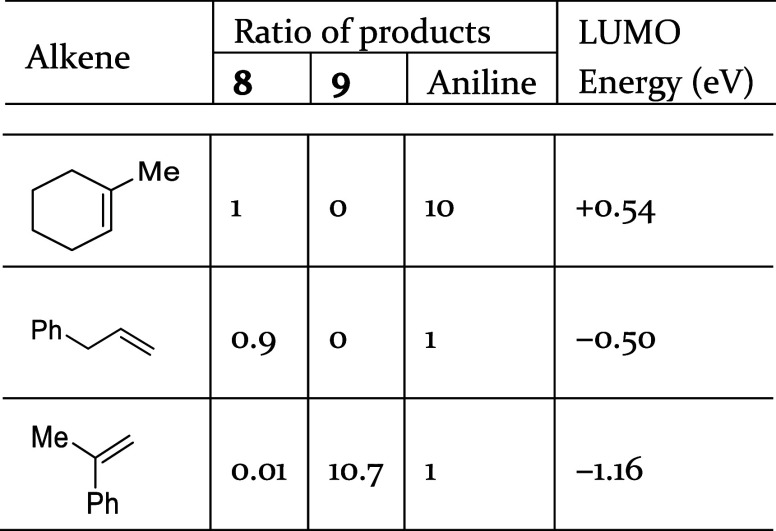
Attempts at HA Optimization Demonstrate Product Distribution Is Linked to Alkene LUMO Energy

**Chart 1:**
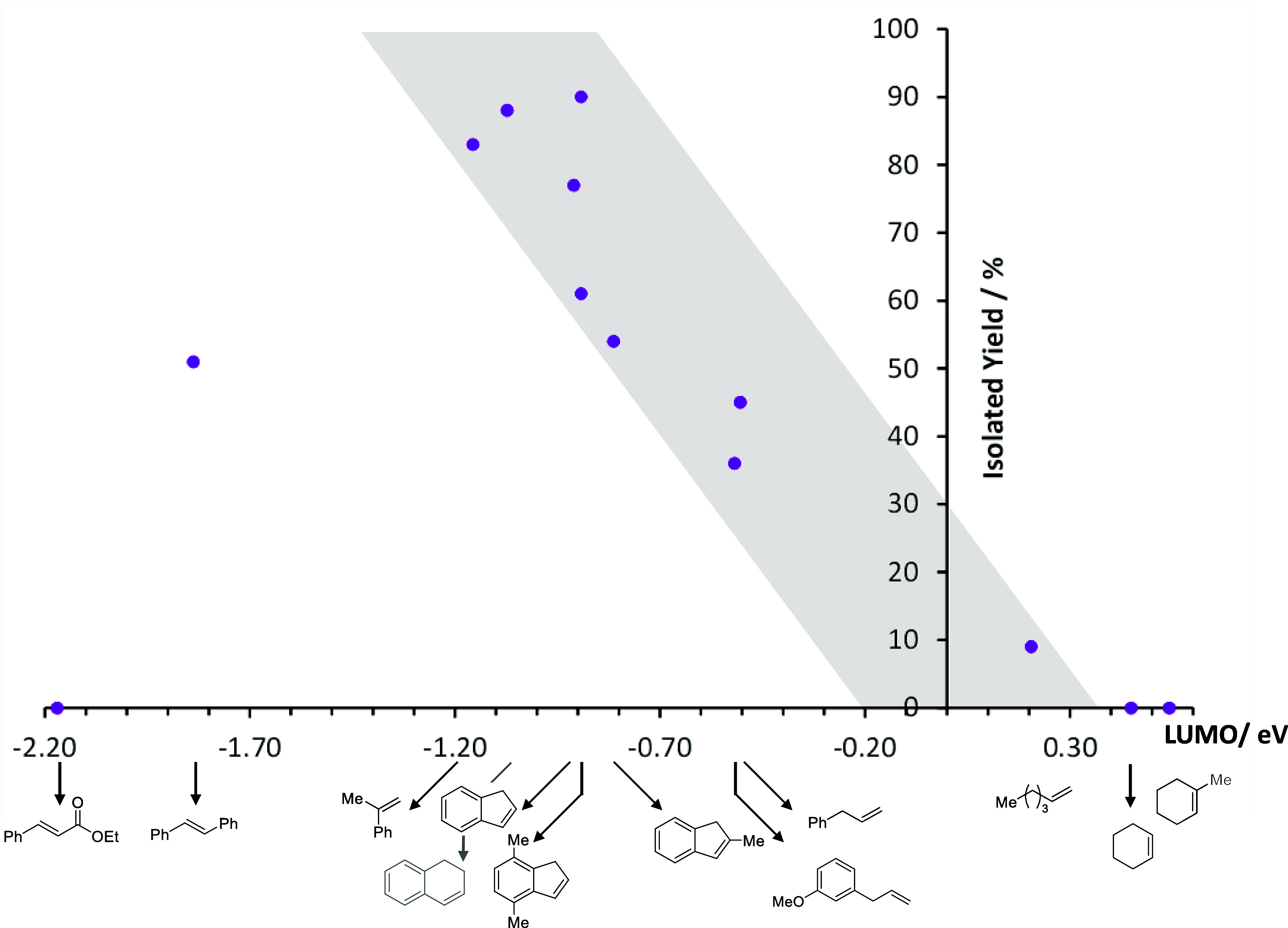
Ability of 1a to undertake HA Catalysis is Inherently Linked to the LUMO Energy of the Alkene Coupling Partner

**Figure 5:**
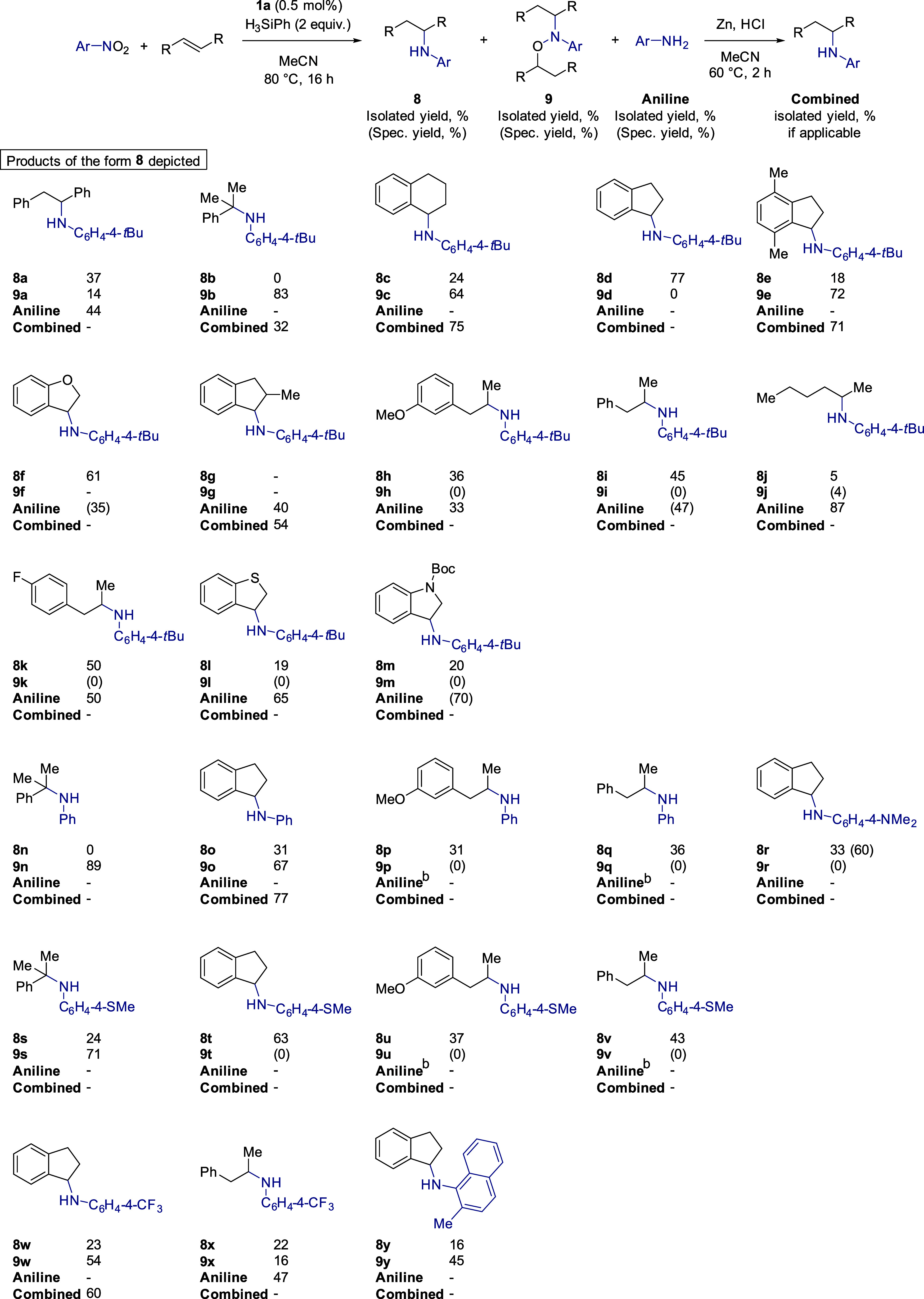
Probing the substrate dependency on the LUMO energy for the HA of nitroarenes. (a) Conditions: 1 mmol of nitro compound, 3 mmol of alkene, 0.5 mol % **1a**, 2 mmol of H_3_SiPh, 0.5 mL of MeCN, 80 °C, 16 h. Isolated yields reported. Spectroscopic yield is provided within parentheses. All spectroscopic yields are based on ultra-high-performance liquid chromatography (UHPLC) data calibrated against an internal standard. If yield is not reported, the mass balance was deemed sufficient without this additional data point. (b) Remainder of the yield was primary amine, identified by ultra-performance liquid chromatography (UPLC), but not isolated.

We therefore wondered if we could use the LUMO energy from a DFT calculation of a given alkene as a simple predictive tool for its competency in HA. We selected a range of alkenes with LUMO energies that span from −2.17 eV (ethyl cinnamate) up to 1-methylcyclohexene at +0.54 eV and applied these in HA using the conditions optimized for allylbenzene. The energy of the SOMO of **1c** is calculated to be −6.08 eV (see the Supporting Information). Gratifyingly, a general trend is observed (Chart 1, isolated yields shown in Figure 5) whereby alkenes with very positive LUMO energies tend not to react and over-reduction to form 4-*tert*-butylaniline dominates (clearly, the SOMO/LUMO gap is too large in this case). In contrast, as we move to more negative LUMO energies, the HA reaction (generating **8** and/or **9**) begins to dominate. However, for ethyl cinnamate, no product is observed and an insoluble precipitate forms; this is likely due to polymerization of alkene as the more stable radical builds up in the system. Therefore, we can conclude that simple LUMO calculations can be used as a qualitative guide to predict reactivity of an alkene. For instance, an alkene with a LUMO energy of 0.00 eV is likely to give only a modest (approximately 15%) amount of **8** and **9**, whereas an alkene with a LUMO energy of −0.95 eV is likely to give approximately 85% of products **8** and **9**. The ability to predict **8** versus **9** is not possible using this simple qualitative measure, but some general trends can be established (see the Supporting Information for a chart showing the LUMO energy versus the yields of **8**, **9**, and aniline data presented in Figure 5).

Clearly the optimum substrates to use for HA have a LUMO energy of approximately −1 eV (indenes, 1,2-dihydronaphthalene, and styrenes). In contrast, nonactivated alkenes are not suitable for catalysis (allyl benzenes through to cyclic and acyclic aliphatic alkenes). This provides a nice level of complementarity to the work of Baran, where HA failed with styrenes but outcompetes our catalyst’s ability to undertake HA on aliphatic substrates. Based on our studies to date, this is likely due to the difference in the electronic nature of the two different iron hydrides being formed (from **1a** versus Fe(acac)_3_^27^ as the precatalysts).

In order to prove the hypothesis that alkenes with LUMO energies of approximately −1 eV will perform well and those with LUMO energies ≥−0.5 eV will perform poorly, we undertook a more detailed substrate scope study (Figure 5).^60^ We further expanded the scope to investigate 4-fluoroallylbenzene, which shows no discernible sign of the *N*,*O*-alkylated product (**9k**) and **8k** is isolated in a 50% yield. Benzo[*b*]thiophene does not behave like benzofuran with the former only giving 19% **8l** and 65% 4-*tert*-butylaniline compared to 61/35% of **8f**/**9f** for benzofuran. Boc-protected indole does allow HA with the protecting group remaining intact, but only 20% **8m** and a 70% spectroscopic yield of the aniline is obtained. As expected, changing to nitrobenzene affords similar HA results as 4-*tert*-butylnitrobenzene (compare products of the form **b**, **d**, **h** and **i** to those of the form **n**, **o**, **p** and **q**). Changing the electronics of the nitro aromatic compound has some effect on the ratio of products formed, but overall, the isolated yield of the HA product is good. For example methyl(4-nitrophenyl)sulfane reacts with indene to afford **8t** in a 63% isolated yield, 1-nitro-4-(trifluoromethyl)benzene reacts with indene to afford **8w** in a 23% isolated yield, **9w** in 54%, and a combined yield following Zn/HCl reduction of 60%. Pleasingly, even sterically challenging nitro aromatics, such as 2-methyl-1-nitronaphthalene, can be applied under our general HA conditions, generating the indenyl product **8y** in a 16% isolated yield and the *N*,*O*-alkylated species, **9y**, in 45%. As expected, across all nitro aromatics, 3-methoxyallylbenzene does not perform well (products **h**, **p** and **u**). It is worth noting that isolated yields are reported, with spectroscopic yields being challenging due to the complex nature of the ^1^H NMR spectra of the crude reaction mixtures.

**Figure 6:**
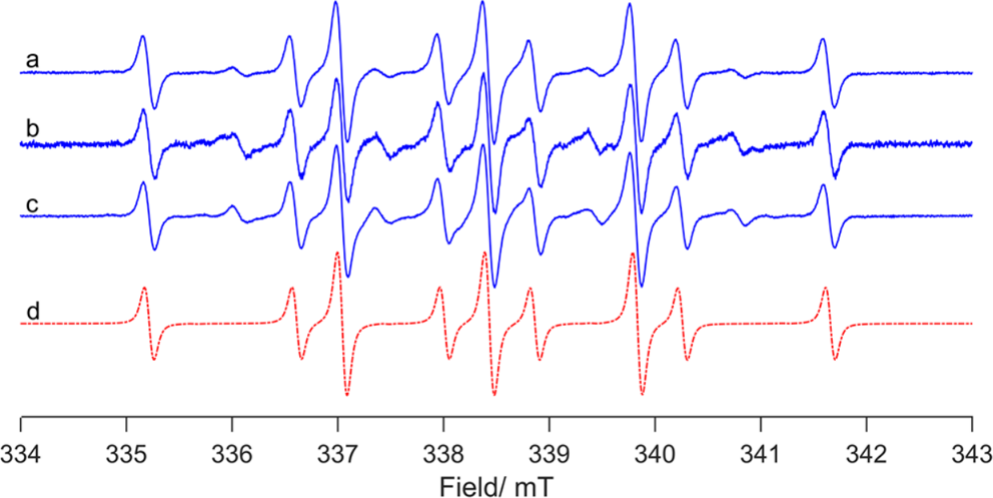
CW EPR spectra (*T* = 298 K) of the reaction of **1a** with PhSiH_3_, *t*Bu_5_NO_2_, and (a) *trans*-stilbene, (b) indene, and (c) hexene in the presence of DMPO. The simulation of a trapped H-DMPO adduct is given in (d).

Similar to the nitro reduction, EPR studies in the presence of **1a** and the spin-trapping agent 5,5-dimethyl-1-pyrroline-*N*-oxide (DMPO) generate the hydrogen-atom trapping product DMPO-H. Unfortunately, no alkyl radicals are detected under catalytic conditions (recorded at 298 K) in the presence of *trans*-stilbene, indene, or 1-hexene (Figure 6). When phenyl *N*-*tert*-butylnitrone (PBN) is employed, there is also some evidence for silane and amine adducts of PBN being formed at 298 K, but again there is no direct evidence for the presence of alkyl radicals (see the Supporting Information). We are pleased to be able to report strong evidence for HAT through these EPR studies, even though no alkyl radicals are detected; we expect these to be short-lived intermediates so their detection is not trivial.

Further control reactions also support a cycle that involves alkyl radical generation and trapping by a nitroso intermediate. For example, reaction of **1a**, 4-*tert*-butylaniline, allylbenzene, and H_3_SiPh does not afford a secondary amine product, but the anti-Markovnikov hydrosilylation product, phenyl(3-phenylpropyl)silane, forms in trace amounts (7%) along with trace propyl benzene (4%). The anti-Markovnikov hydrosilylation product forms quantitatively when 1-hexene is employed in the reaction of **1a**, 4-*tert*-butylaniline, and H_3_SiPh. These are clearly not the product of a radical reaction, and the presence of a nitro/nitroso species is necessary for HA, doing so in a Markovnikov fashion, i.e., via the most stable alkyl radical.

Reaction of **1a** (0.5 mol %), nitrosobenzene (1 mmol), indene (3 mmol), and H_3_SiPh (2 mmol) affords 38% azobenzene (**6a**′), 10% (**8o**), and trace aniline, indicating that nitroso is active in catalysis but must not build up to any extent under our standard HA conditions. In other words, in high concentrations, the nitroso is sequestered in competing side reactions. Reaction of indene (3 mmol), H_3_SiPh (2 mmol), and **1a** (0.5 mol %) affords oligomeric material, as evidenced by DOSY NMR spectroscopy (see the Supporting Information). In the absence of a suitable trapping reagent (nitroso compound), radical polymerization dominates. This, coupled with the observation that allylbenzene generates propyl benzene (*vide infra*) albeit in small quantities, indicates that the transformation could be occurring via HAT.

## Conclusions

In summary, we applied a simple catalytic pairing of **1a** and HBpin to enable the facile reduction of nitro organics. We used reaction tuning through the choice of a reductant to give chemoselectivity in the presence of other reducible functional groups. The mechanism was studied using a range of analytical techniques such as EPR and LIFDI-MS and—coupled with kinetic studies and electronic structure investigations on postulated key intermediates—we can propose a reaction mechanism that proceeds via two catalytic cycles interlinked via a key iron hydride complex. HBpin is too active to allow access to a hydroamination catalytic cycle, but again, using a less active reductant does allow for effective HA. We have shown that a simple MO-based predictor from DFT calculations can be used to assess the suitability of an alkene for use in HA reactions. With the high levels of electronic tuning available to us through salen ligand modification, we should be able to tune the SOMO energy of the precatalyst and thus modify our ability to undertake HA of different alkenes; this is an interesting hypothesis and one that we are currently testing.

## Supporting Information Available

Data relevant to the computational studies reported here, including methodology, benchmarking studies, energetics, and coordinates of characterized stationary points (ZIP)
Analysis data and NMR spectra for all products (PDF)
