# Supplementary material for: Synthetic and Mechanistic Studies into the Reductive Functionalization of Nitro Compounds Catalyzed by an Iron(salen) Complex
Source: J Am Chem Soc. 2024 Jul 12;146(29):19839–51. doi: 10.1021/jacs.4c02797 (PMC11273354; doi:10.1021/jacs.4c02797)
Supplement: Supplementary file 2 — ja4c02797_si_002.pdf [file ja4c02797_si_002.pdf]

Electronic supporting information:

Synthetic and Mechanistic Studies into the Reductive Functionalization of Nitro Compounds Catalyzed by an Iron(salen) Complex

Emily Pocock,[a] Martin Diefenbach,[†b] Thomas M. Hood,[†a] Michael Nunn,[c] Emma Richards,[*d] Vera Krewald[*b] and Ruth L. Webster[*e]

[a] Department of Chemistry, University of Bath, Claverton Down, Bath, United Kingdom, BA2 7AY.

[b] Department of Chemistry, TU Darmstadt, 64287 Darmstadt, Germany.

[c] Early Chemical Development, Pharmaceutical Sciences, Biopharmaceuticals R&D, AstraZeneca, Macclesfield, United Kingdom, SK10 2NA.

[d] School of Chemistry, Cardiff University, Main Building, Park Place, Cardiff, United Kingdom, CF103AT

[e] Yusuf Hamied Department of Chemistry, University of Cambridge, Lensfield Road, Cambridge, United Kingdom, CB2 1EW

Table of Contents

1.	General Considerations	S3
2.	Synthesis of $[Fe(salen)]_2$-μ-oxo Catalyst (1a)	S4
2.1	Procedure for the Synthesis of N,N'-Bis(salicylidene)ethylenediamine Ligand	S4
2.2	Procedure for the Synthesis of the Iron Complex (1a)	S4
3.	Synthesis of the [Fe(II)(salen)] Catalyst (1b)	S5
4.	*In situ* Reduction of Nitro-Compounds	S7
4.1	Optimisation of the synthesis of aromatic primary amines	S7
4.2	General Procedure for the *in situ* Reduction of Aromatic Nitro-compounds	S7
4.3	General Procedure for the *in situ* Reduction of Aliphatic Nitro-compounds	S17
5.	Reduction and Isolation of Nitro-Compounds	S22
5.1	General Procedure for the Reduction of Aromatic Nitro Compounds	S22
5.2	General Procedure for the Reduction of Aliphatic Nitro Compounds	S42
6.	Control Reactions	S49
7.	Radical Clock Experiments	S54
7.1	General Procedure for Radical Clock / Trap Reactions	S54
7.2	Reaction of N,N-dimethyl-4-nitrobenzene with 3 equiv. TEMPO	S66
8.	Kinetic Experiments	S57
8.1	General Notes for Kinetic Analysis	S57
8.2	Order with respect to [Fe]	S58
8.3	Kinetic Isotope Effect	S61
8.3.1	Synthesis of DBpin	S61
8.4	Order in HBpin	S62
8.5	Order in Substrate	S69
8.6	Arrhenius Analysis	S73
8.7	Eyring Analysis	S74
9.	Mass Spectrometry	S75
10.	LIFDI-MS Data	S77
11.	One-Pot Hydroaminations	S78
11.1	Optimisation Table for One-Pot Hydroaminations	S78
11.2	Graphical Evidence to demonstrate that product distribution can be linked to alkene LUMO energy.	S79
11.3	Hydroamination Mechanistic Control Reactions	S79
11.3.1	Subjecting 1-hexene to standard hydroamination conditions	S80
11.3.2	Subjecting allylbenzene to standard hydroamination conditions	S81
11.3.3	Subjecting indene to standard hydroamination conditions	S82
11.3.4	Subjecting t-butyl aniline to standard hydroamination conditions	S84
11.3.5	Subjecting nitroso benzene to standard hydroamination conditions	S85
11.4	General Procedure for One-Pot Hydroaminations	S85
12.	Electron Paramagnetic Resonance	S214
13.	Quantum Chemical Studies	S221
13.1	Computational Methods	S221
13.2	Catalytic Cycles for the Reduction of $^{i}PrNO_2$	S222
13.3	Catalytic Cycles for the Reduction of $PhNO_2$	S233
13.4	Cartesian Coordinates of Optimized Geometries (Å)	S236
14.	References	S281

1. General Considerations

Ligand and pre-catalyst synthesis were performed in air. All other manipulations were carried out using standard Schlenk-line and glovebox techniques under an inert atmosphere of argon (Ar). An MBraun MB200B glovebox was employed operating at <0.1 ppm O_2 and <0.1 ppm H_2O. Acetonitrile was dried over two batches of 3 Å molecular sieves, sparged with Ar and stored under an inert atmosphere. Benzene, toluene, and tetrahydrofuran (THF) were distilled from Na/benzophenone and stored over activated 3 Å molecular sieves. Acetonitirile-d_3 was dried over two batches of 3 Å molecular sieves, sparged with Ar and stored under an inert atmosphere. Benzene-d_6 was degassed and dried over sodium and freeze-pump-thaw degassed. Glassware was dried for 12 hours at 120°C prior to use.

1H, 13C{1H}, 19F{1H} and 11B{1H} NMR spectra were recorded on Bruker Advance or Agilent 500, and 400 MHz NMR spectrometers. In CD_3CN, 1H and 13C{1H} NMR chemical shifts are reported relative to CH_3CN at 1.94 ppm and 118.26 ppm, respectively; in $CDCl_3$, 1H and 13C{1H} NMR chemical shifts are reported relative to $CHCl_3$ at 7.26 ppm and 77.16 ppm, respectively. For the assignment of the 1H and 13C{1H} NMR spectra 2D NMR (COSY, HSQC, HMBC) experiments were also performed. Data were processed in MestReNova. Coupling constants (J) are reported in Hertz (Hz). Multiplicities are indicated by: br s (broad singlet), s(singlet), d (doublet), t (triplet), q (quartet) and m (multiplet) app. (apparent).

Infrared (IR) spectra of neat compounds were recorded at ambient temperature over the range 4000–650 cm^{-1} using a PerkinElmer Spectrum 100 ATR-FTIR spectrometer using a diamond ATR unit. Peaks are reported in cm^{-1}.

UV-Vis spectra were recorded on a Mettler Toledo UV5 spectrometer. LIFDI mass spectrometry were carried out using a Waters GCT Premier MS Agilent 7890A GC performed at the University of York by Mr. Karl Heaton.

For mass spectrometry a microTOF electrospray time-of-flight (ESITOF) mass spectrometer (Bruker Daltonik GmbH, Bremen, Germany) was used. Data are reported in the form of *m/z*. The observed mass and isotope pattern matched the corresponding theoretical values as calculated from the expected molecular formula.

Analytical thin-layer chromatography was performed on Merck silica gel 60 F254 aluminium-backed plates. Visualisation was accomplished with UV light (254 nm), and vanillin stain. Automated flash column chromatography (normal phase) was performed using a CombiFlash NextGen 300+ system equipped with UV and ELSD detectors, using 4 - 40g silica columns.

Unless otherwise stated, all chemicals were purchased from commercial sources, dried over molecular sieves and used as supplied. Pinacolborane was purchased from Sigma Aldrich and distilled and analysed before use. DBpin and $FeCl_2$.THF were prepared according to a literature procedures.[6]

2. Synthesis of $[Fe(salen)]_2$-μ-oxo Catalyst (1a)

2.1. Procedure for the Synthesis of N,N'-Bis(salicylidene)ethylenediamine Ligand[1]

To a 100 mL round bottom flask containing salicylaldehyde (2.49 g, 18.3 mmol, 3.5 equiv.) in 40 mL dry ethanol was added ethylenediamine (0.31 g, 5.2 mmol, 1 equiv.). The vessel was then heated at reflux for 2 hours. The resulting bright yellow solution was then cooled to 0 °C and filtered to yield a bright yellow residue. The resultant yellow residue was washed with cold EtOH and dried in vacuo to give the desired product as a bright yellow solid (1.31 g, 94%).

2.1.1. N,N'-Bis(salicylidene)ethylenediamine ligand[1]

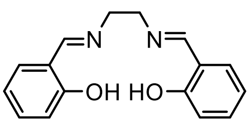

^{1}H NMR ($CDCl_3$, 500 MHz): *δ* 13.19 (s, 2H, Ar-OH), 8.36 (s, 2H, N=CH), 7.31-7.27 (m, 2H, Ar-H), 7.24-7.22 (m, 2H, Ar-H), 6.95-6.93 (m, 2H, Ar-H), 6.87-6.84 (m, 2H, Ar-H), 3.95 (s, 4H, N-CH2-CH2-N). Concordant with literature.

2.2. Procedure for the Synthesis of the Iron Complex (1a)[1]

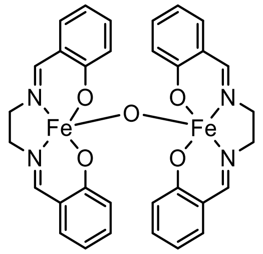

$Fe(OAc)_2$ (0.71 g, 3.9 mmol, 1 equiv.) was weighed into a 250 mL round bottom flask and dissolved in ethanol (50 mL) resulting in a dark brown solution. To this, a solution of N,N'-Bis(salicylidene)ethylenediamine ligand (1.31 g, 4.55 mmol, 1.2 equiv.) in ethanol (90 mL) was added yielding a red solution. The resulting red solution was stirred at 80 °C for 2 hours. The flask was then allowed to cool to room temperature before the resulting red solid was isolated via vacuum filtration to yield a dark red powder (1.39 g, 54%).

3. Synthesis of the [Fe(II)(salen)] Catalyst (1b)

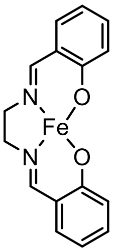

Method A:

A suspension of salenH (0.24 g, 0.89 mmol) and KHMDS (0.35 g, 1.75 mmol) in THF (5 mL) was treated with FeCl2.3/2THF (0.21 g, 0.90 mmol) giving a deep purple solution. The volatiles were removed in vacuo to give a dark bright purple solid which was used without further purification. Yield: 214 mg (74 %).

^{1}H NMR (C_6D_6, 400 MHz): δ 44.7, 28.0, 3.6, 1.4, -4.4, -6.0, -19.4.

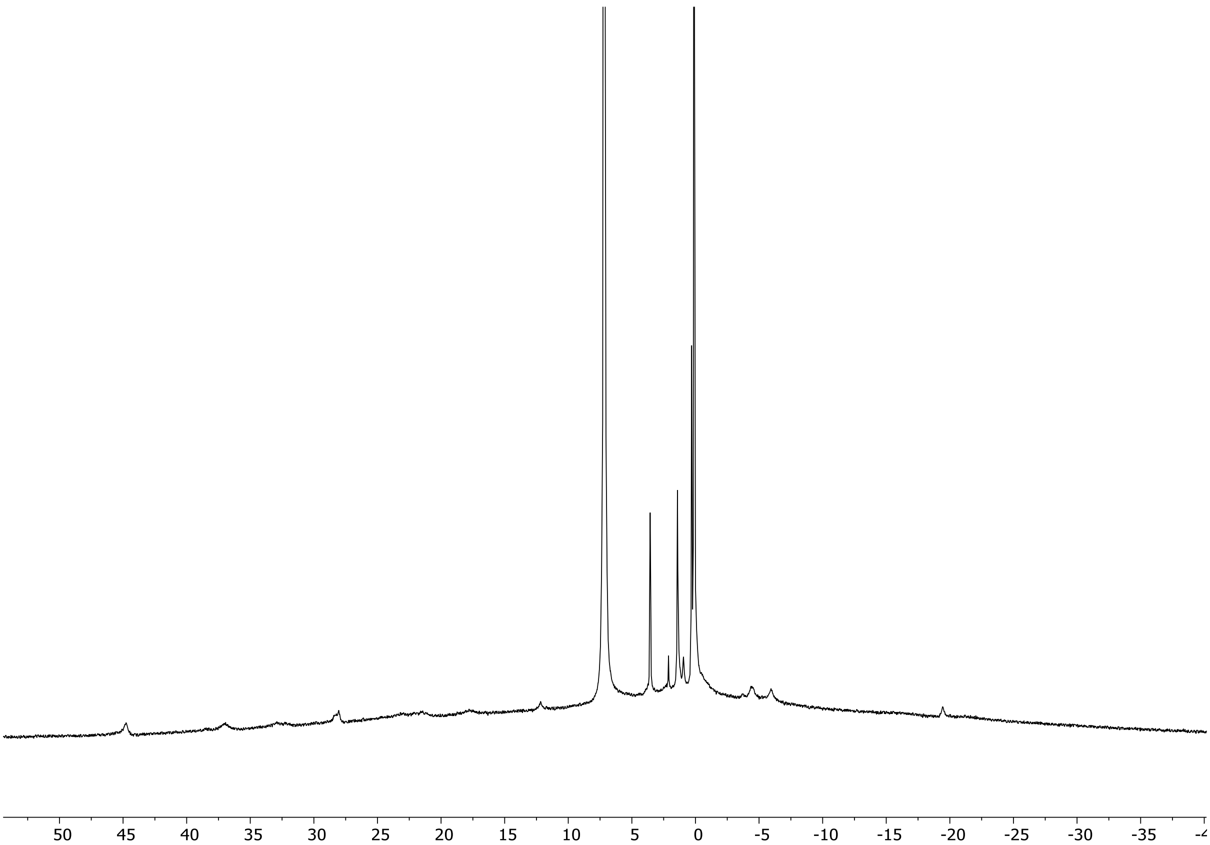

Figure S1: ^{1}H NMR Spectrum of 1b in C_6D_6.

Method B:

A suspension of **1a** (56.0 mg, 84.8 umol) in C_6H_6 (2 mL) was treated with KC_8 (45.9 mg, 340 umol) and mixed for 1 hour to give a dark blue suspension. Excess KC_8 and carbon were removed by filtration to give a purple solution which was dried in vacuo to give a bright purple solid. Yield: 37 mg (66%).

EPR analysis of this sample in the presence of excess KC_8 was consistent with only KC_8 indicating the formation of Fe(II) only.

^{1}H NMR (C_6D_6, 400 MHz): δ 44.7, 28.0, 3.6, 1.4, -4.4, -6.0, -19.4.

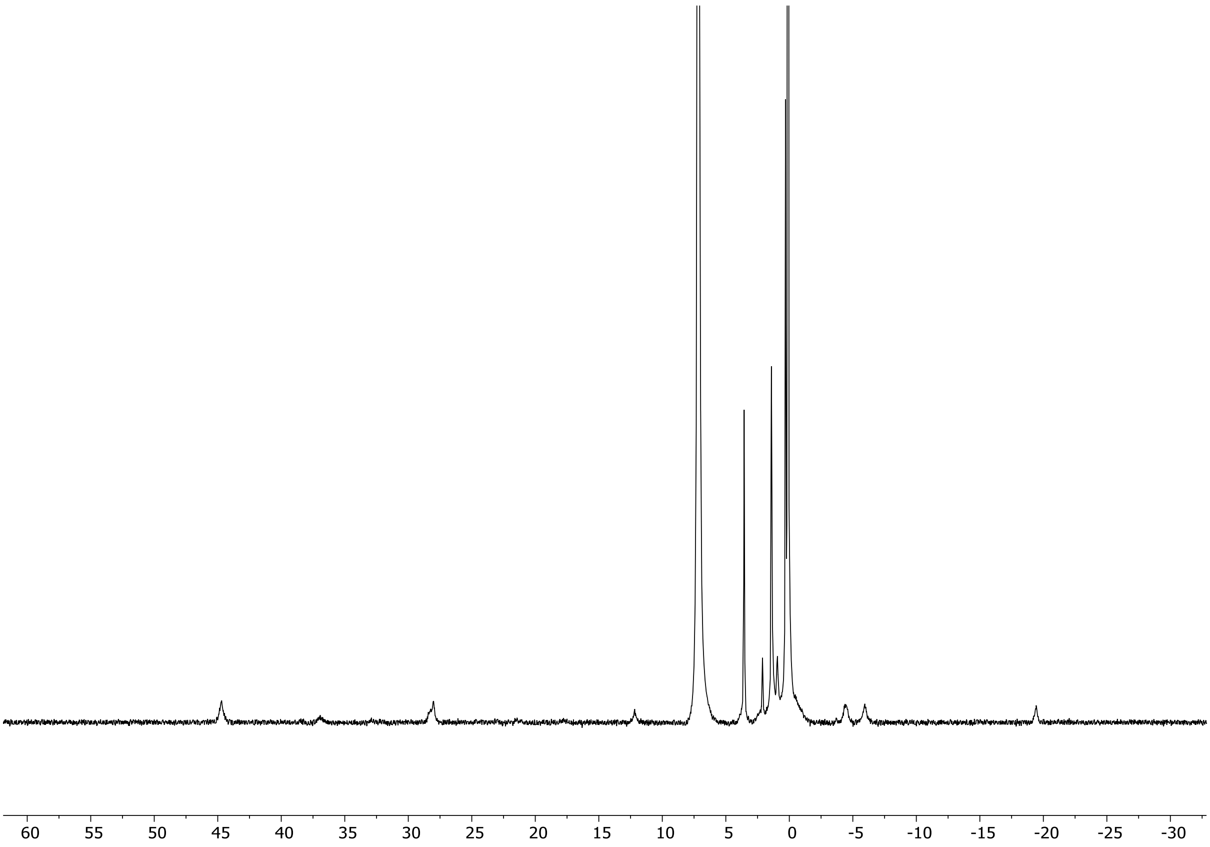

Figure S2: ^{1}H NMR Spectrum of 1b in C_6D_6.

4. *In situ* Reduction of Nitro-Compounds

4.1. Optimisation of the synthesis of aromatic primary amines

Table S1: Optimisation Table for the Synthesis of Aromatic Primary Amines

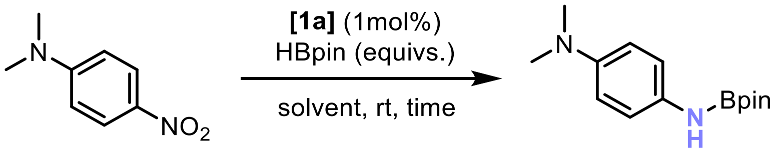

Entry	Solvent	HBpin (equiv.)	[Fe] 1a (mol%)	Spectroscopic Conversion (10 mins)	Spectroscopic Conversion (1h)
1	Pentane	3	1	-	-
2	DCM	3	1	-	-
3	C_6D_6	3	1	42	47
4	MeCN	3	1	22(86)*	22(86)*
5	MeCN	4	1	88	88
6	MeCN	5	1	90	90
7	MeCN	5	2	85	85
8	MeCN	5	0.1	26	26
9	MeCN	5	0	-	-
10	MeCN	2(H_3SiPh)	1	-	77†
11	MeCN	$HSi(OEt)_3$	1	-	86†
12	MeCN	PMHS	1	-	-

*Overall conversion including 'free' amine
†Conversion after 16 h at 50 °C
Reaction Conditions: *N,N*-dimethyl-4-nitrobenzene (0.03 mmol), [**1a**] (1 mol% unless overwise stated), solvent (0.5 mL). Spec yields calculated against 1 equiv. of dimethyl carbonate (internal standard).

4.2. General Procedure for the *in situ* Reduction of Aromatic Nitro-compounds:

To a J-Young NMR tube under argon atmosphere was added a mixture of **1a** (0.003 mmol, 2 mg) and nitro-compound (0.3 mmol, 1 equiv.) in CD_3CN (0.6 mL). To the solution was added pinacolborane (1.5 mmol, 0.22 mL, 5 equiv.) and the reaction mixture was agitated for 10 minutes, NMR spectra were recorded. Spectroscopic yields were calculated by comparison to the internal standard dimethyl carbonate (δ 3.72, 6H). (‡ pinBOBpin byproduct (δ 1.23, 12H)).
In the case of compounds 2i, 23k and 3n spectroscopic yields were not obtained due to peak overlap and broadening.

4.2.1. 4,4,5,5-tetramethyl-N-phenyl-1,3,2-dioxaborolan-2-amine **2a**

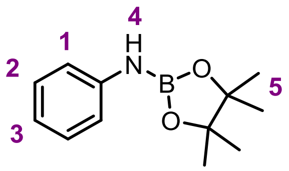

^{1}H NMR (CD_3CN, 400 MHz) δ 7.18- 7.09 (m, 4H, C^1-H, C^2-H), 6.60 (t, J = 7.2 Hz, 1H, C^3-H), 5.34 (*br* s, 1H, N^4-H), 1.28 (s, 12H, C^5-H)

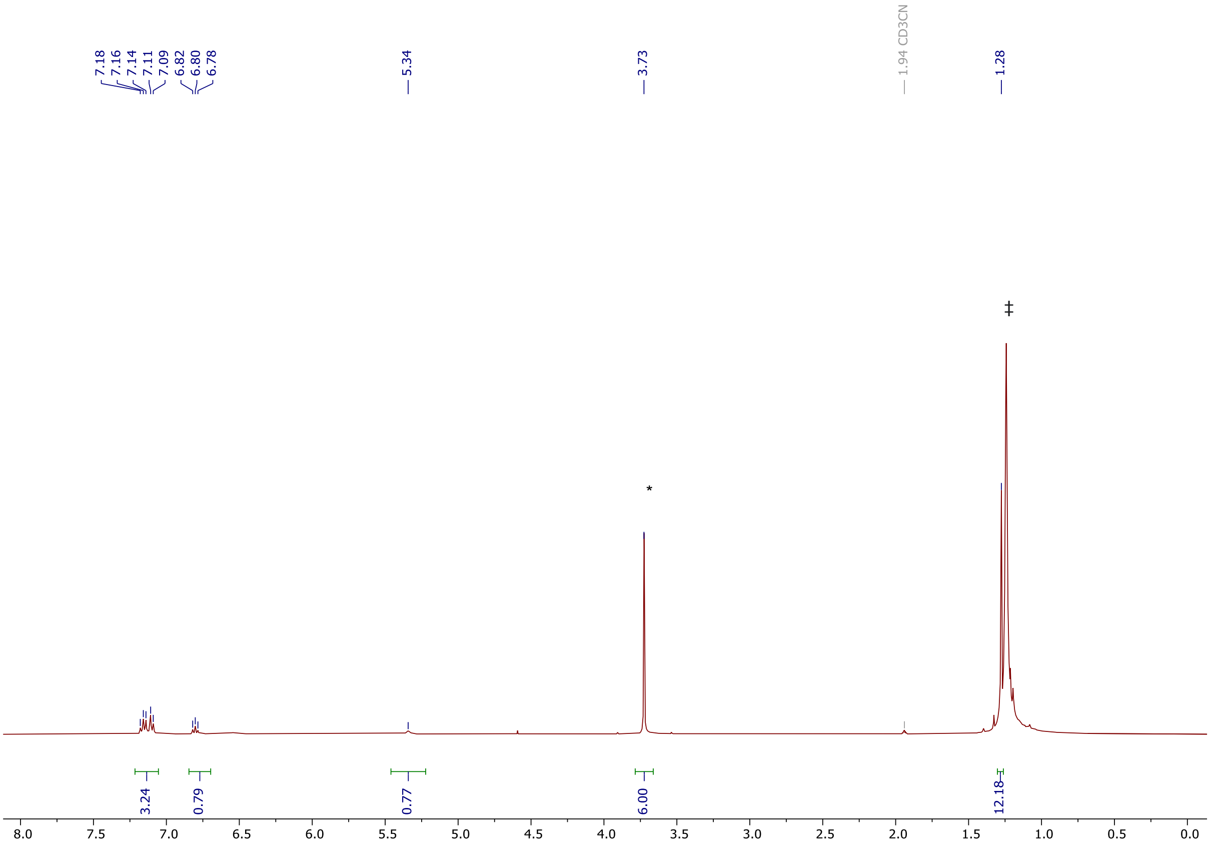

Figure S3: ^{1}H NMR Spectrum of 2a in CD_3CN 10 minutes after the addition of HBpin (singlet at 3.73 corresponds to the dimethyl carbonate internal standard).

4.2.2. N-(4-(tert-butyl)phenyl)-4,4,5,5-tetramethyl-1,3,2-dioxaborolan-2-amine **2b**

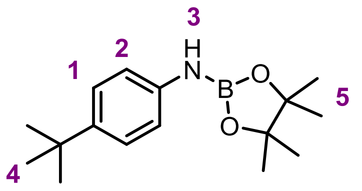

^{1}H NMR (CD_3CN, 400 MHz) *δ* 7.19 (d, J = 8.7 Hz, 2H, C^{1}-H), 7.02 (d, J = 8.7 Hz, 2H, C^{2}-H), 5.22 (*br* s, 1H, N^{3}-H), 1.26 (s, 9H, C^{4}-H), 1.24 (s, 12H, C^{5}-H).

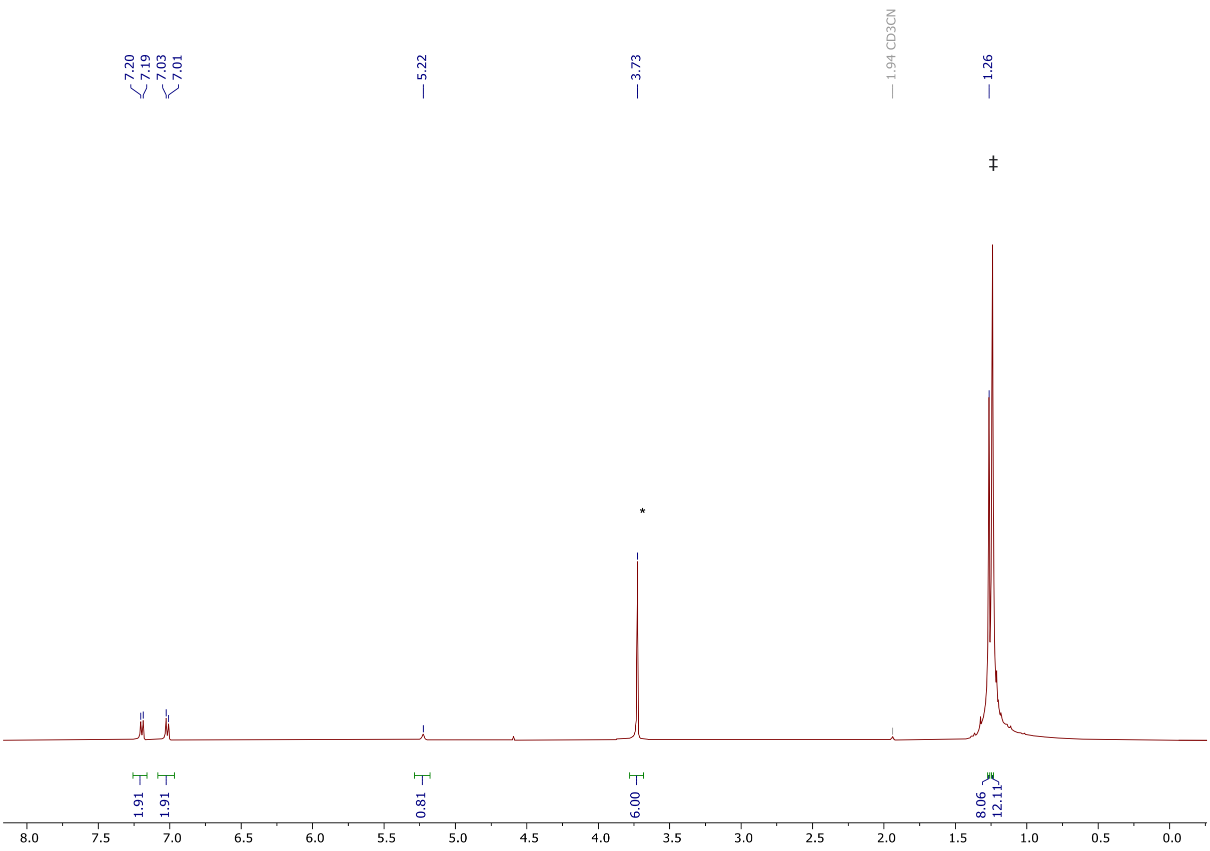

Figure S4: ^{1}H NMR Spectrum of 2b in 10 minutes after the addition of HBpin (singlet at 3.74 corresponds to the dimethyl carbonate internal standard).

4.2.3. N1,N1-dimethyl-N4-(4,4,5,5-tetramethyl-1,3,2-dioxaborolan-2-yl)benzene-1,4-diamine **2c**

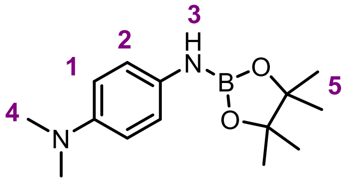

^{1}H NMR (CD_3CN, 400 MHz) δ 6.95 (d, J = 8.4 Hz, 2H, C^2-H), 6.65 (d, J = 8.5 Hz, 2H, C^1-H), 5.00 (*br* s, 1H, N^3-H), 2.79 (s, 6H, C^4-H), 1.25 (s, 12H, C^5-H).

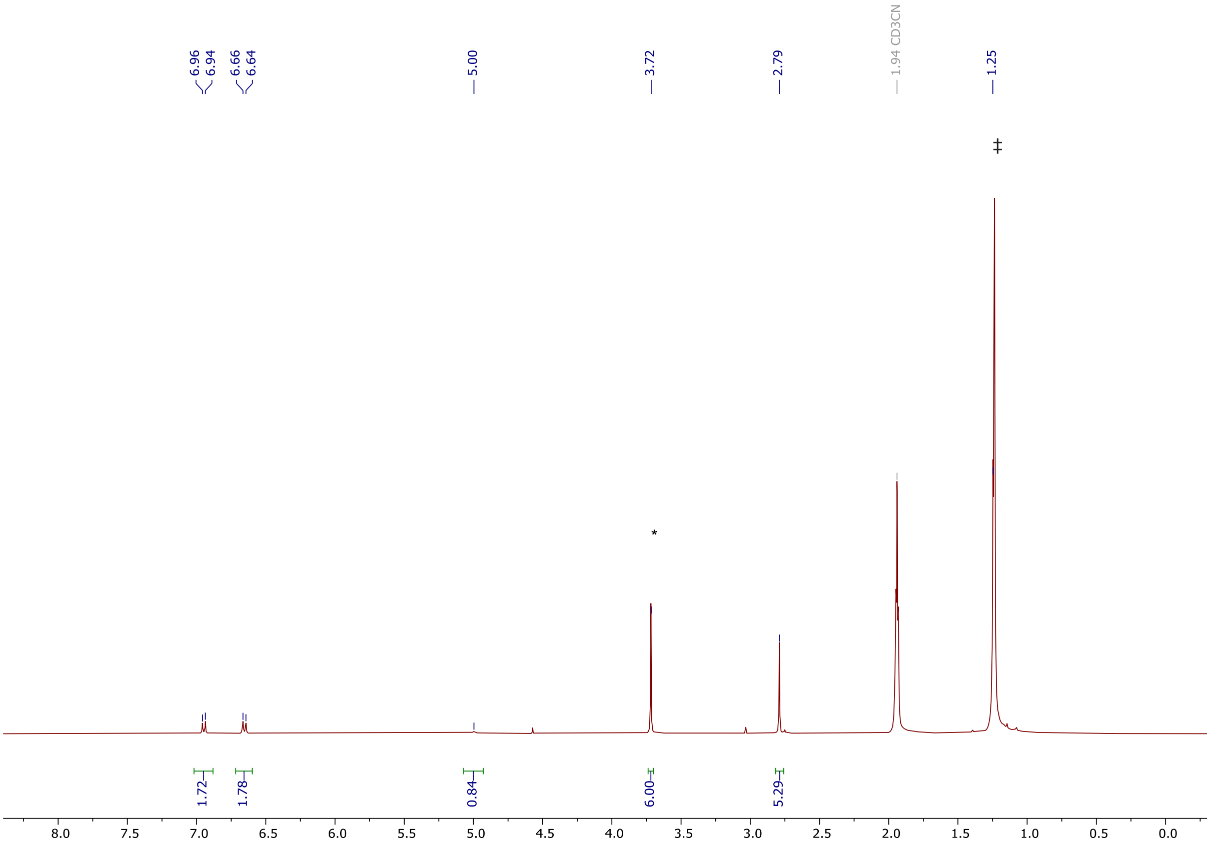

Figure S5: ^{1}H NMR Spectrum of 2c in CD_3CN 10 minutes after the addition of HBpin (singlet at 3.72 corresponds to the dimethyl carbonate internal standard). (Due to overlap with pinBOBpin by-product the C^5-H could not be integrated)

4.2.4. 4,4,5,5-tetramethyl-N-(4-(methylthio)phenyl)-1,3,2-dioxaborolan-2-amine **2d**

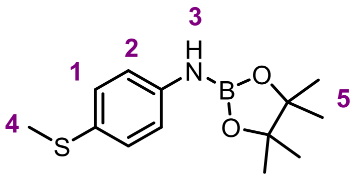

^{1}H NMR (CD_3CN, 400 MHz) *δ* 7.14 (app. *br* s, 2H, C^1-H), 7.06 (app. *br.* s, 2H, C^2-H), 5.36 (*br* s, 1H N^3-H), 2.38 (s, 3H, C^4-H), 1.26 (s, 12H. C^5-H).

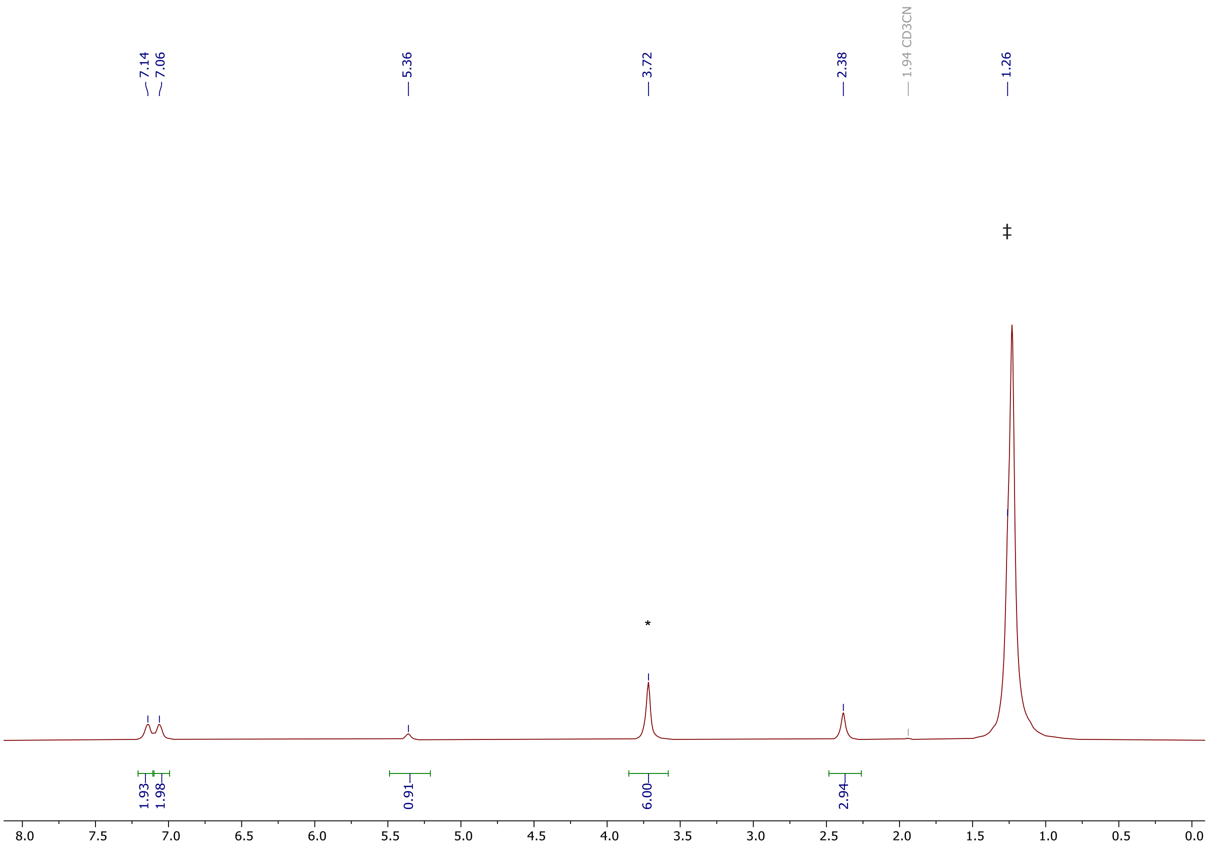

Figure S6: ^{1}H NMR Spectrum of 2d in CD_3CN 10 minutes after the addition of HBpin (singlet at 3.72 corresponds to the dimethyl carbonate internal standard). (Due to overlap with pinBOBpin by-product the C^5-H could not be integrated)

4.2.5. 4-((4,4,5,5-tetramethyl-1,3,2-dioxaborolan-2-yl)amino)benzenethiol **2e**

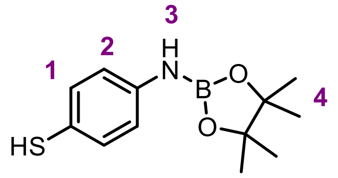

^{1}H NMR (CD_3CN, 400 MHz) *δ* 7.27 (d, J = 8.7 Hz, 2H, C^1-H), 7.08 (d, J = 8.8 Hz, 2H, C^1-H), 5.52 (*br* s, 1H, N^3-H), 1.26 (s, 12H, C^4-H).

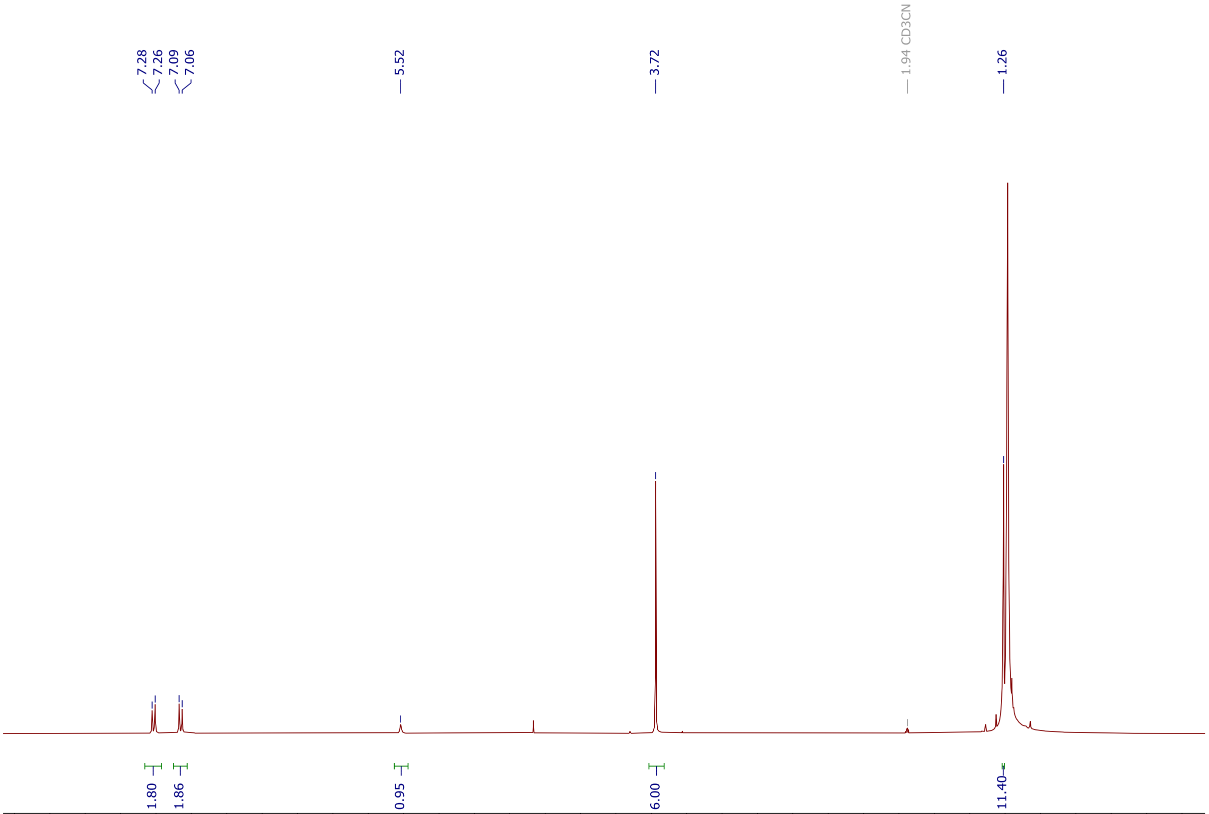

Figure S7: ^{1}H NMR Spectrum of 2e in CD_3CN 12h after the addition of HBpin (singlet at 3.72 corresponds to the dimethyl carbonate internal standard).

4.2.6. N-(4-bromophenyl)-4,4,5,5-tetramethyl-1,3,2-dioxaborolan-2-amine **2f**

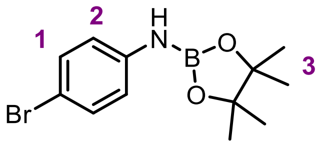

^{1}H NMR (CD_3CN, 400 MHz) *δ* 7.37 - 6.95 (*br* m, 4H, C^2-H and C^1-H), NH (not observed due to spectra broadening), 1.23 (s, 12H, C^3-H).

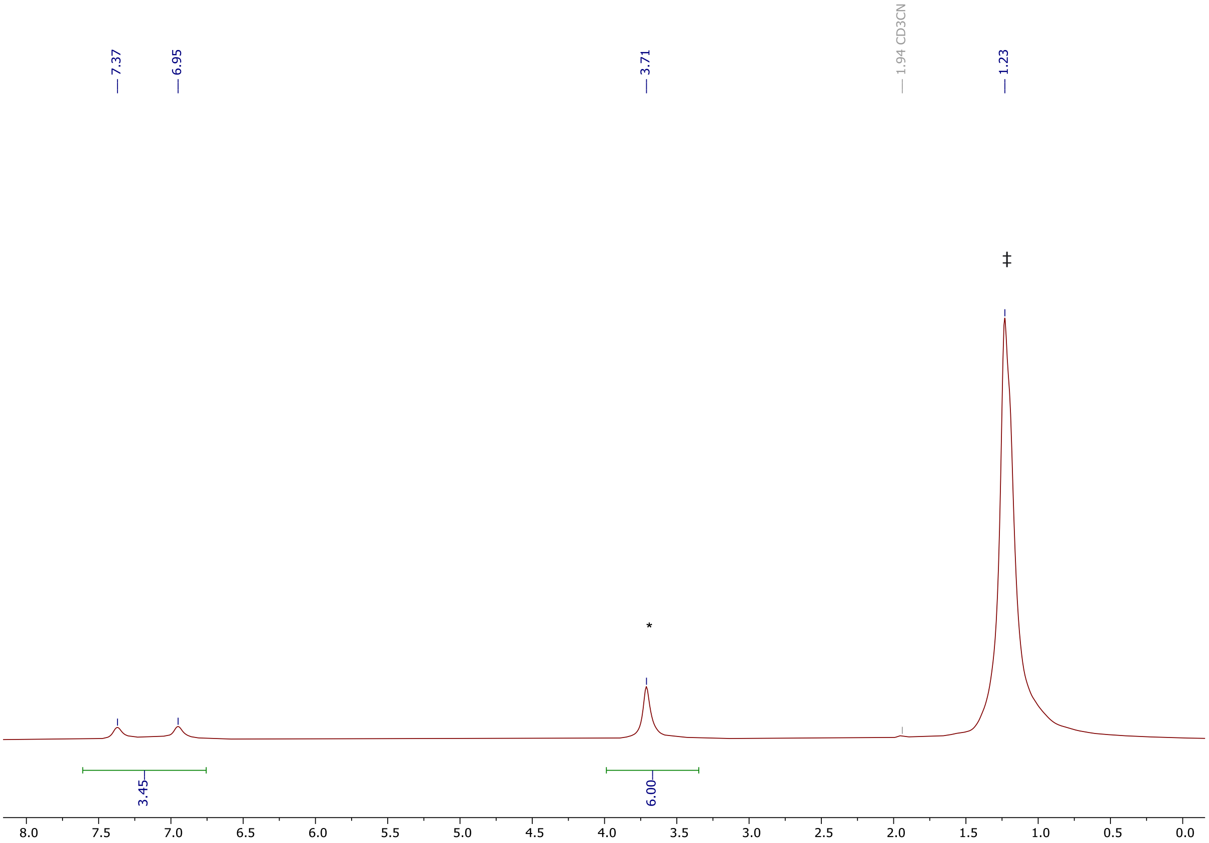

Figure S8: ^{1}H NMR Spectrum of 2f in CD_3CN three days after the addition of HBpin (singlet at 3.71 corresponds to the dimethyl carbonate internal standard). (Due to overlap with pinBOBpin by-product the C^3-H could not be integrated)

4.2.7. 1-(4-((4,4,5,5-tetramethyl-1,3,2-dioxaborolan-2-yl)amino)phenyl)ethan-1-one **2g**

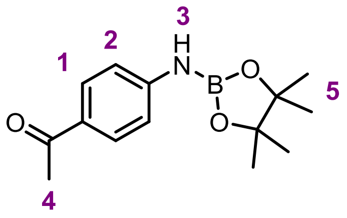

^{1}H NMR (CD_3CN, 400 MHz) *δ* 7.80 (d, J = 8.7 Hz, 2H, C^1-H), 7.18 (d, J = 8.7 Hz, 2H, C^2-H), 5.92 (*br* s, 1H, N^3-H), 2.46 (s, 3H, C^4-H), 1.28 (s, 12H, C^5-H).

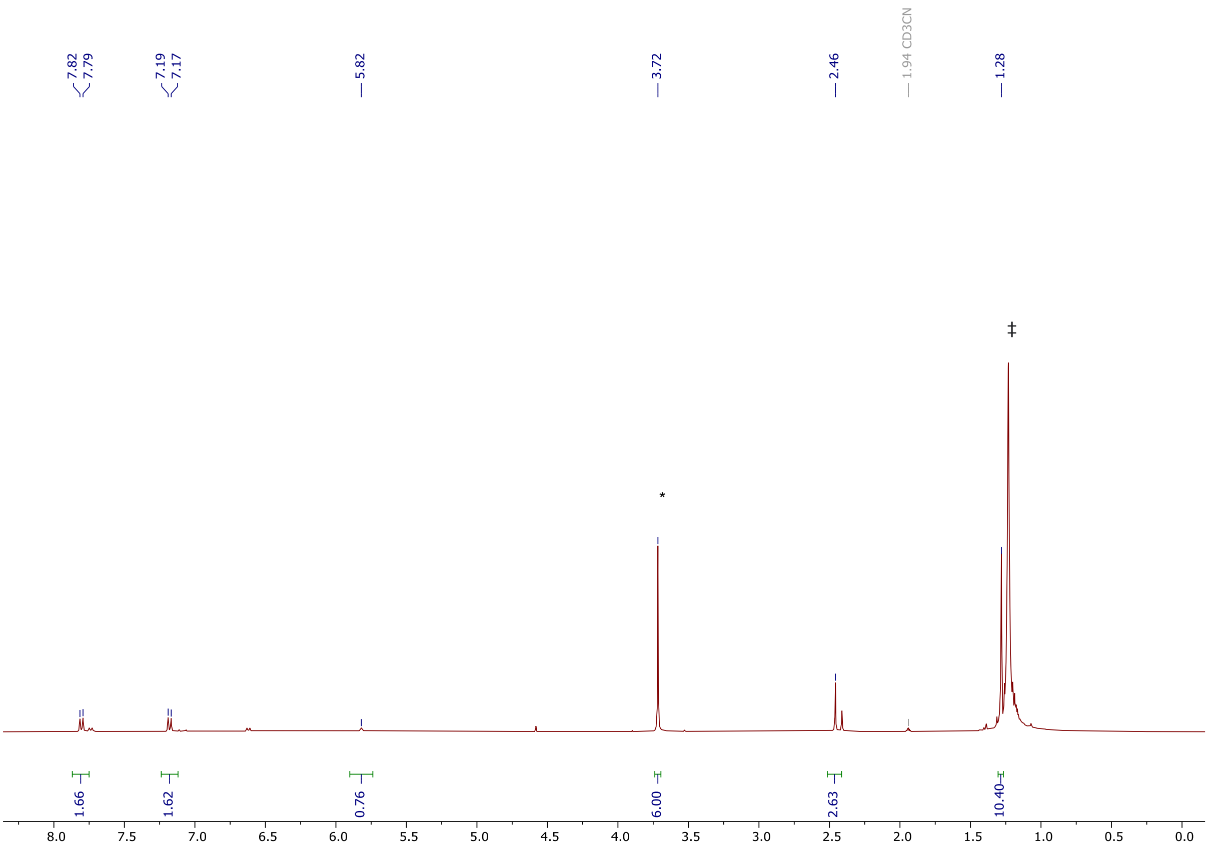

*Figure S9: ^{1}H NMR Spectrum of 2g in CD_3CN 10 minutes after the addition of HBpin (singlet at 3.72 corresponds to the dimethyl carbonate internal standard). *Other component present in the spectra corresponds to the fully reduced product.*

4.2.8. 4,4,5,5-tetramethyl-N-(4-(((4,4,5,5-tetramethyl-1,3,2-dioxaborolan-2-yl)oxy)methyl)phenyl)-1,3,2-dioxaborolan-2-amine **2h**

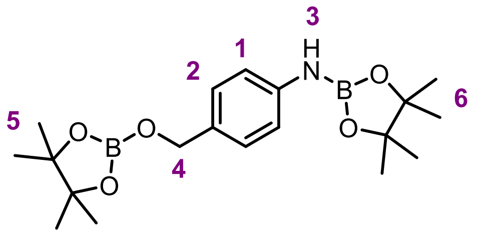

^{1}H NMR (CD_3CN, 400 MHz) δ 7.13 (d, J = 8.4 Hz, 2H, C^1-H), 7.08 (d, J = 8.4 Hz, 2H, C^2-H), 5.38 (*br* s, 1H, N^3-H), 4.73 (s, 2H, C^4-H), 1.27 (s, 12H, C^5-H/ C^6-H), 1.23 (s, 12H, C^5-H/ C^6-H).

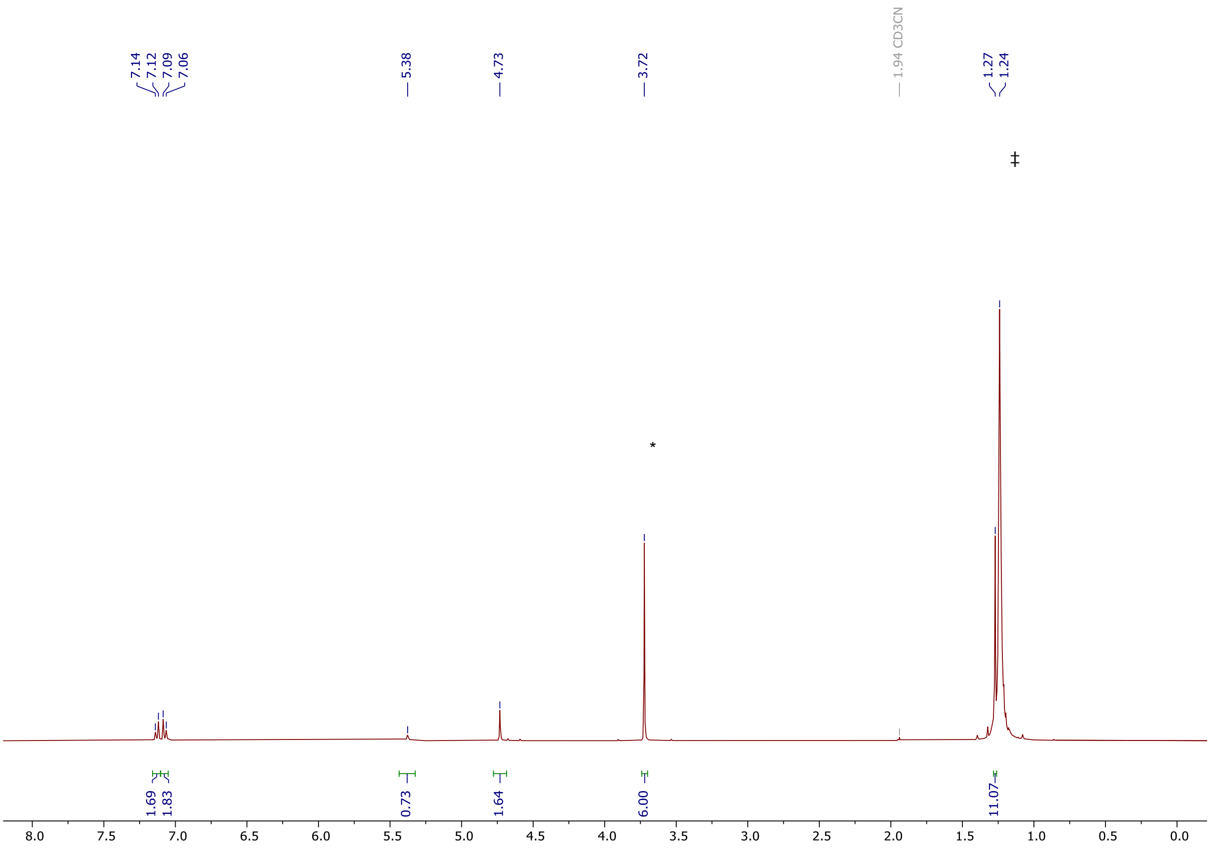

Figure S10: ^{1}H NMR Spectrum of 2h in CD_3CN 10 minutes after the addition of HBpin (singlet at 3.72 corresponds to the dimethyl carbonate internal standard). (Due to overlap with pinBOBpin by-product the $C^{5/6}$-H could not be integrated)

4.2.9. 4,4,5,5-tetramethyl-N-(3-((4,4,5,5-tetramethyl-1,3,2-dioxaborolan-2-yl)oxy)phenyl)-1,3,2-dioxaborolan-2-amine **2j**

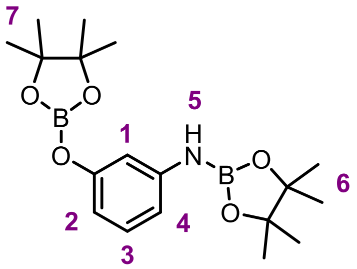

^{1}H NMR (CD_3CN, 400 MHz) *δ* 7.05 (app. t, J = 8.1 Hz, 1H, C^3-H), 6.90 (app. t, J = 2.2 Hz, 1H C^1-H), 6.75 (dd, J = 8.1, 1.3 Hz, 1H, C^2-H), 6.49 (dd, J = 8.1, 1.5 Hz, 1H, C^4-H), 5.40 (*br* s, 1H, N^5-H), 1.29 (s, 12H, C^6-H/ C^7-H), 1.27 (s, 12H, C^6-H/ C^7-H).

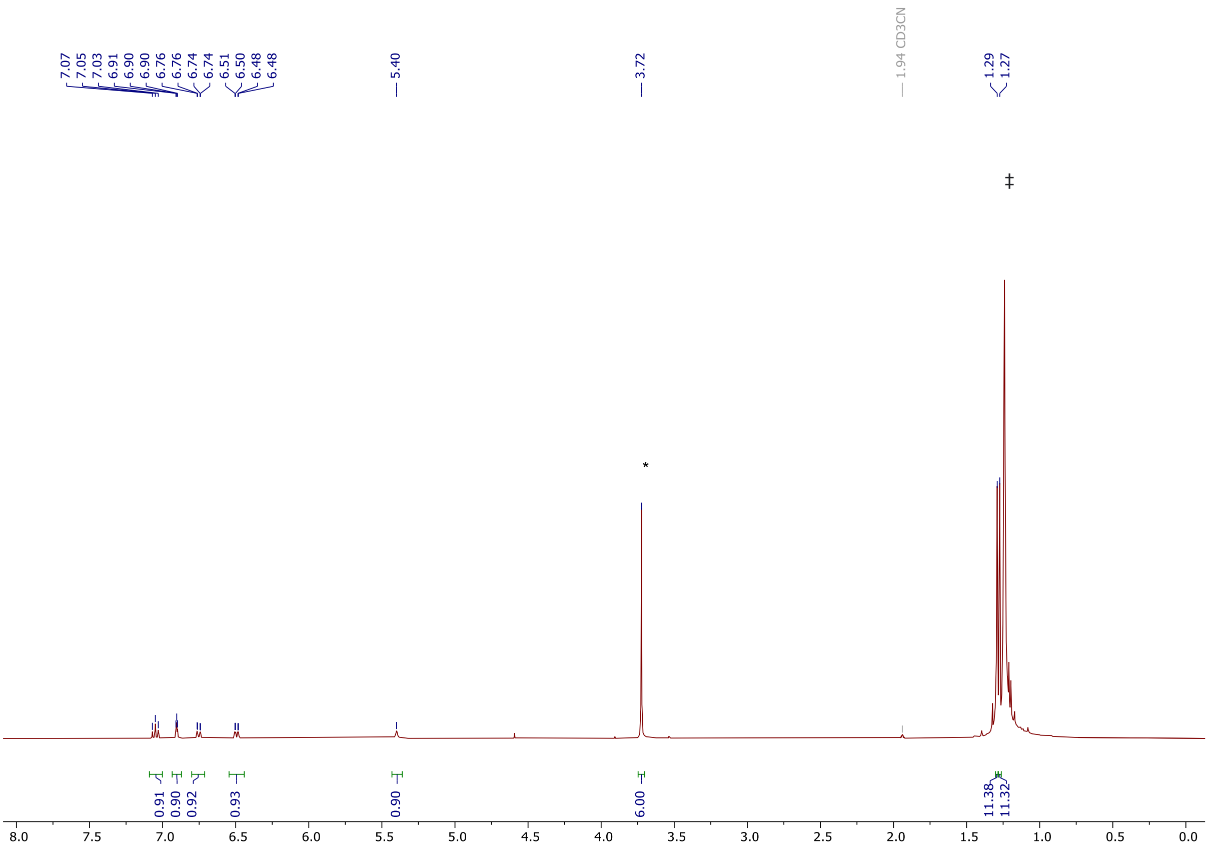

Figure S11: ^{1}H NMR Spectrum of 2j in CD_3CN 12h after the addition of HBpin (singlet at 3.72 corresponds to the dimethyl carbonate internal standard).

4.3. General Procedure for the *in situ* Reduction of Aliphatic Nitro-compounds:

To a J-Young NMR tube under argon atmosphere was added a mixture of **1a** (0.003 mmol, 2 mg) and nitro-compound (0.3 mmol, 1 equiv.) in CD_3CN (0.6 mL). To the solution was added pinacolborane (1.5 mmol, 0.22 mL, 5 equiv.) and the reaction mixture was agitated for 16h at room temperature, NMR spectra were recorded. Spectroscopic yields were calculated by comparison to the internal standard dimethyl carbonate * (δ = 3.72, 6H).
(pinBOBpin byproduct ‡ (δ = 1.23, 12H)).

4.3.1. N,4,4,5,5-pentamethyl-1,3,2-dioxaborolan-2-amine **2l**

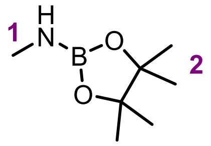

^{1}H NMR (CD_3CN, 400 MHz) δ 2.48 (s, 3H, C^1-H), 1.16 (s, 12H, C^2-H).

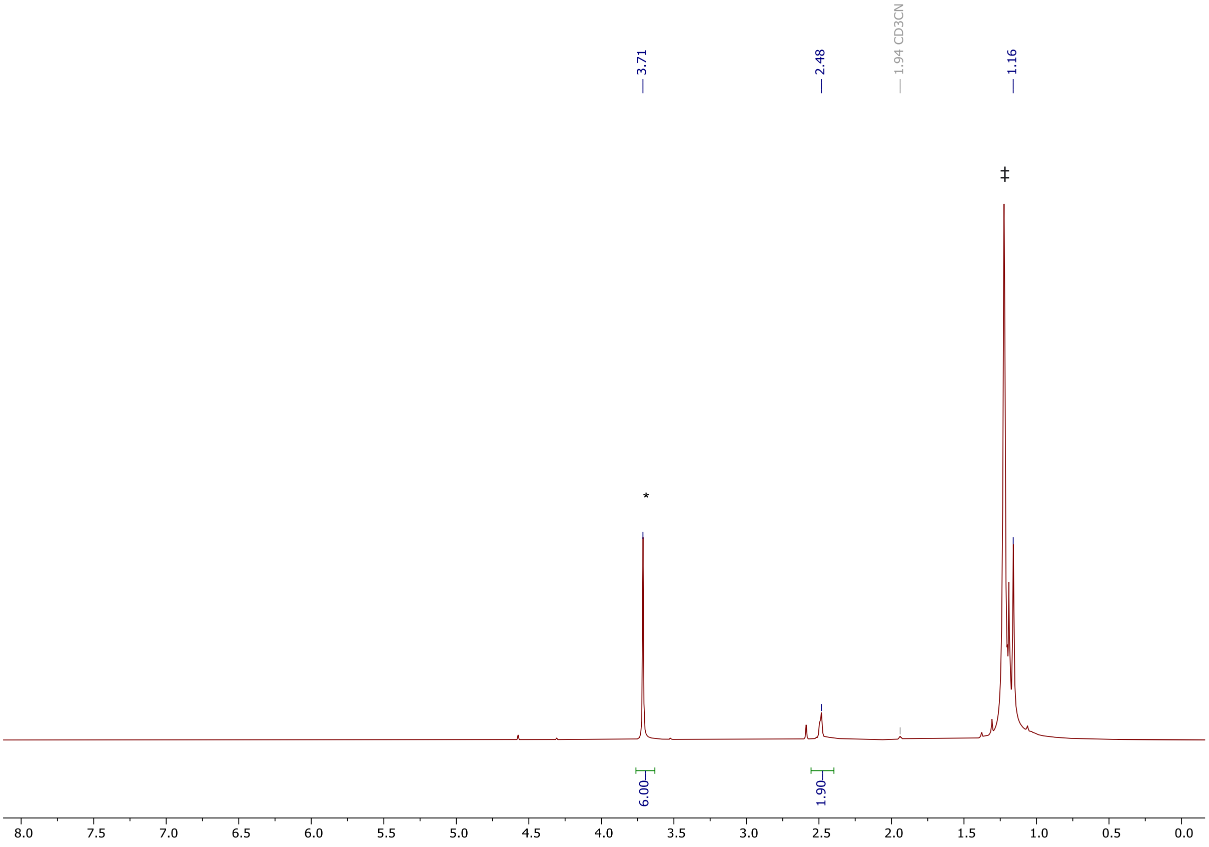

Figure S12: ^{1}H NMR Spectrum of 2l in CD_3CN 16h after the addition of HBpin (singlet at 3.71 corresponds to the dimethyl carbonate internal standard). (Due to overlap with pinBOBpin by-product the C^2-H could not be integrated)

4.3.2. N-ethyl-4,4,5,5-tetramethyl-1,3,2-dioxaborolan-2-amine **2m**

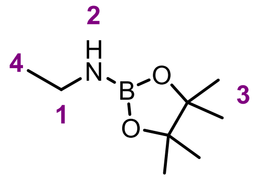

^{1}H NMR (CD_3CN, 400 MHz) δ 2.852 (*app.* pent, J = 7.1 Hz, 2H, C^1-H), 2.60 (*br* s, 1H, N^2-H), 1.16 (s, 12H, C^3-H), 1.00 (t, J = 7.1 Hz, 3H, C^4-H).

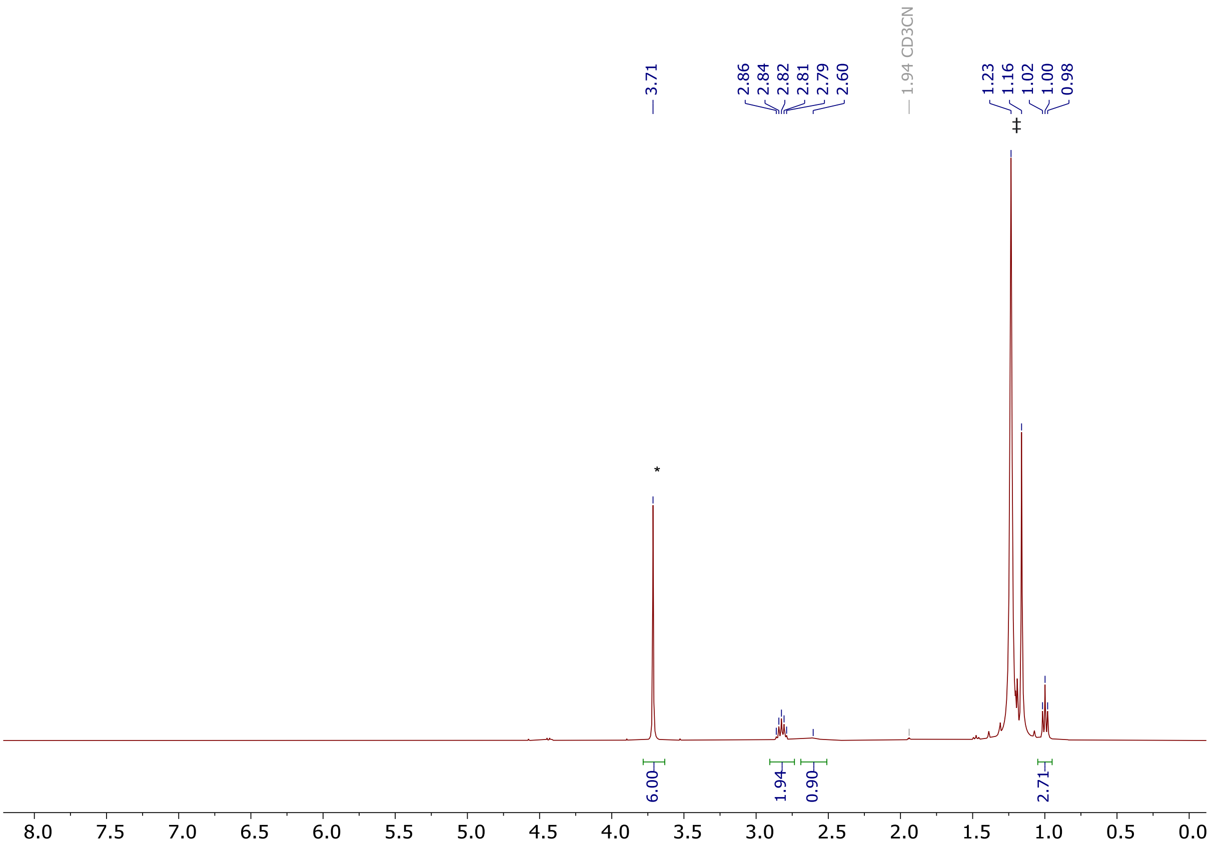

Figure S13: H NMR Spectrum of 2m in CD_3CN 16h after the addition of HBpin (singlet at 3.71 corresponds to the dimethyl carbonate internal standard). (Due to overlap with pinBOBpin by-product the C^3-H (1.16 ppm) could not be integrated)

4.3.3. N-isopropyl-4,4,5,5-tetramethyl-1,3,2-dioxaborolan-2-amine **2n**

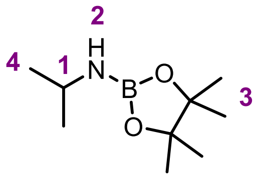

^{1}H NMR (CD_3CN, 400 MHz) δ 3.29 – 3.17 (m, 1H, C^1-H), 2.49 (*br* s, 1H, N^2-H), 1.16 (s, 12H, C^3-H), 1.03 (d, J = 6.5 Hz, C^4-H).

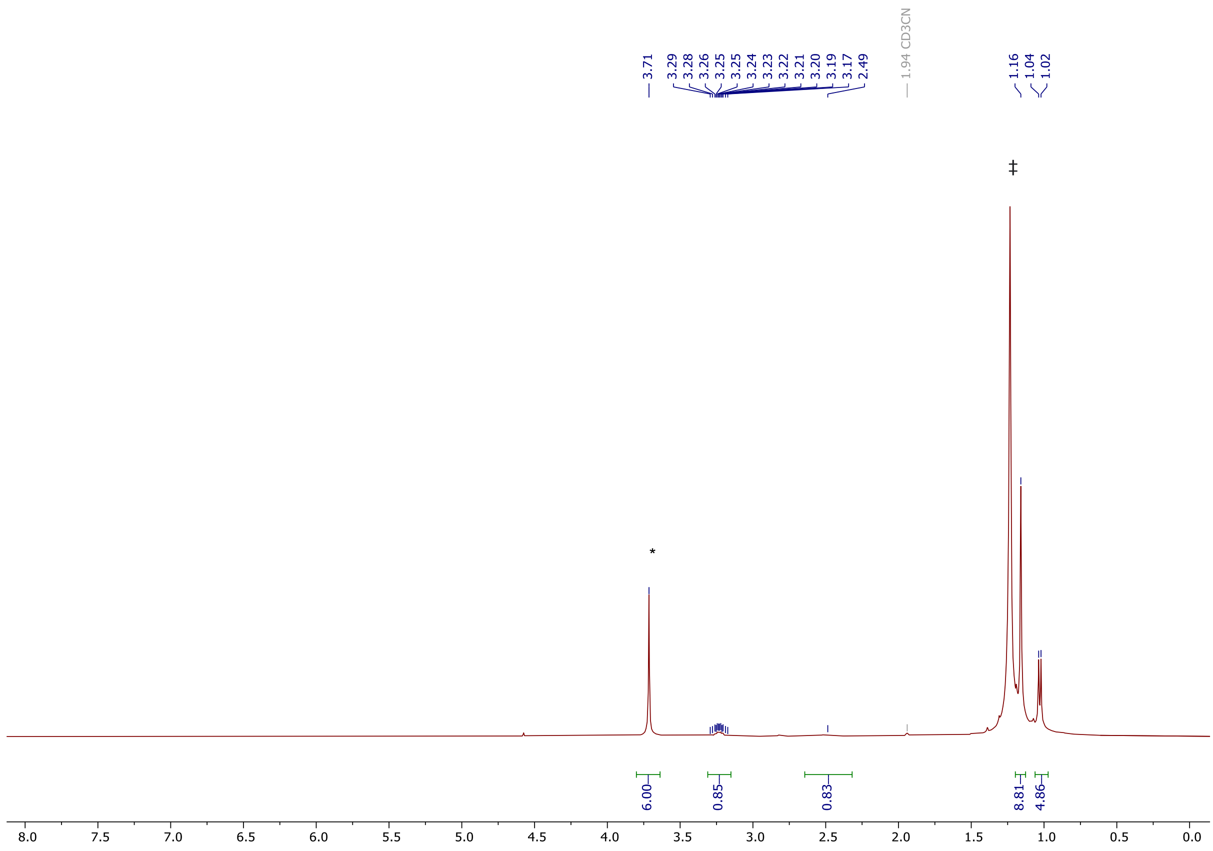

Figure S14: ^{1}H NMR Spectrum of 2n in CD_3CN 16h after the addition of HBpin (singlet at 3.71 corresponds to the dimethyl carbonate internal standard).

4.3.4. 4,4,5,5-tetramethyl-N-propyl-1,3,2-dioxaborolan-2-amine **2o**

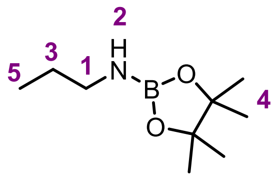

^{1}H NMR (CD_3CN, 400 MHz) *δ* 2.76 (app. q, *J* = 7.1 Hz, 2H, C^1-H), 2.59 (*br* s, 1H, N^2-H), 1.39 – 1.31 (m, 2H, C^3-H), 1.17 (s, 12H, C^4-H), 0.85 (t, *J* = 7.4 Hz, 3H, C^5-H).

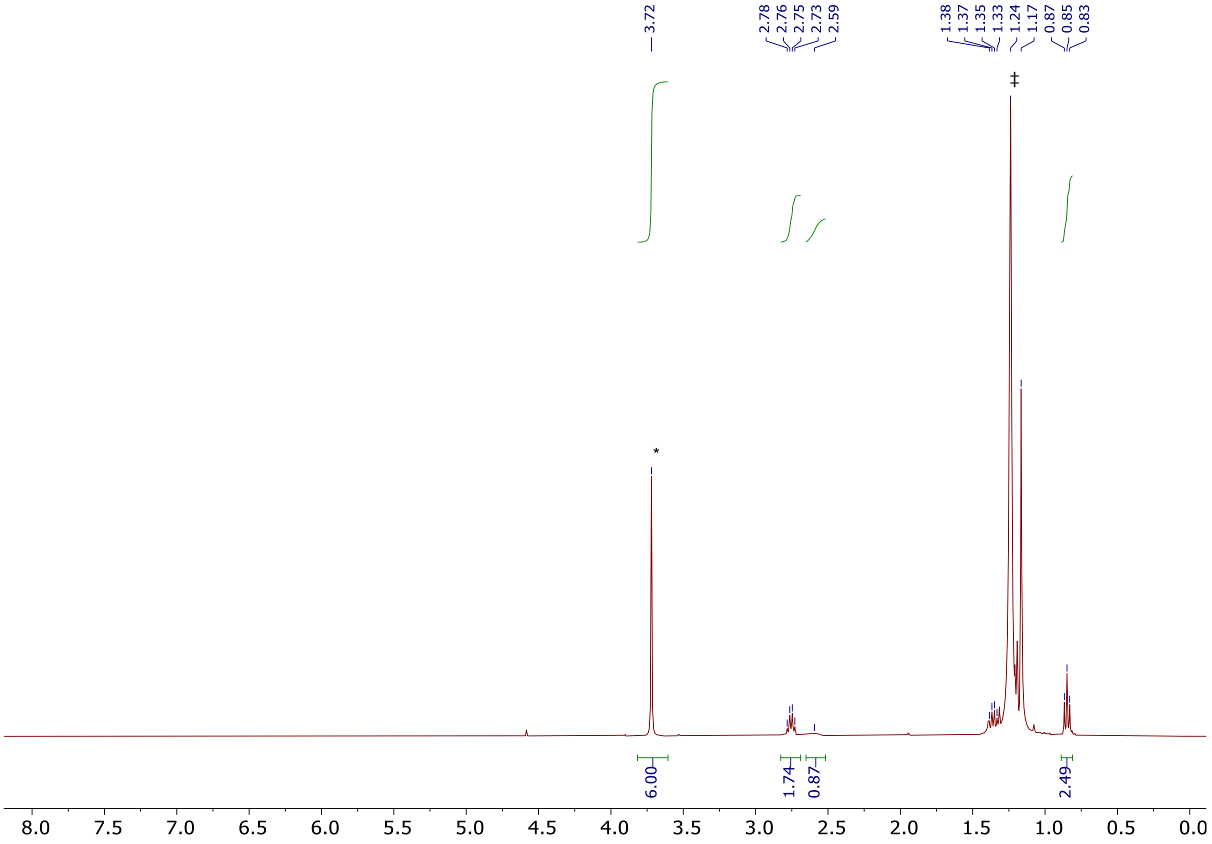

Figure S15: ^{1}H NMR Spectrum of 2o in CD_3CN 16h after the addition of HBpin (singlet at 3.71 corresponds to the dimethyl carbonate internal standard). (Due to overlap with pinBOBpin by-product the C^3-H (1.39-1.31 ppm) and C^4-H (1.17 ppm) could not be integrated).

4.3.5. 4,4,5,5-tetramethyl-N-pentyl-1,3,2-dioxaborolan-2-amine **2q**

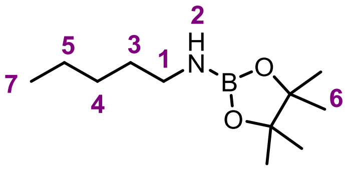

^{1}H NMR (CD_3CN, 400 MHz) δ 2.78 (dd, J = 7.5, 6.7 Hz, 2H, C^1-H), 2.56 (s, 1H, N^2-H), 1.39 – 1.16 (m, 6H, C^3-H/C^4-H/C^5-H), 1.16 (s, 12H, C^6-H), 0.89 (t, J = 6.9 Hz, 3H, C^7-H).

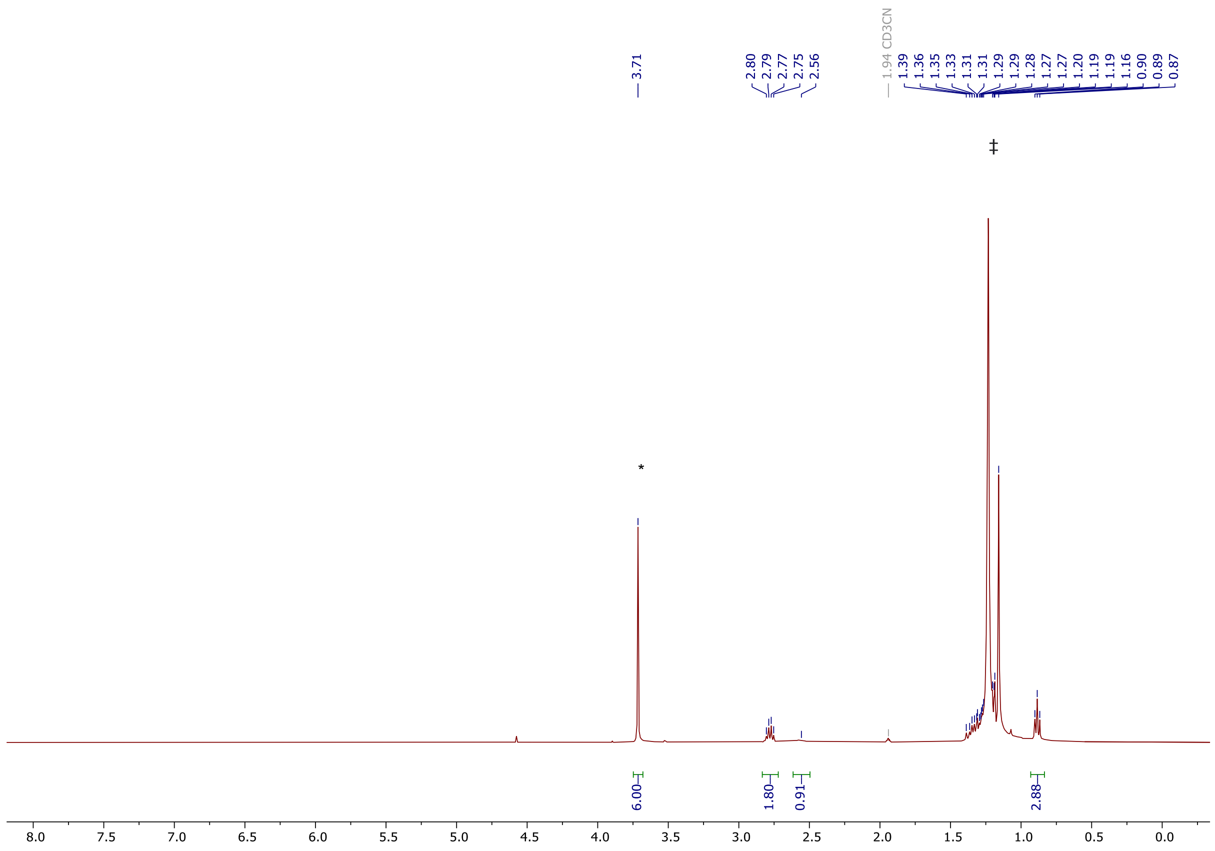

Figure S16: ^{1}H NMR Spectrum of 2q in CD_3CN 16h after the addition of HBpin (singlet at 3.71 corresponds to the dimethyl carbonate internal standard). (Due to overlap with pinBOBpin by-product the C^6-H could not be integrated)

4.3.6. N-cyclopentyl-4,4,5,5-tetramethyl-1,3,2-dioxaborolan-2-amine **2r**

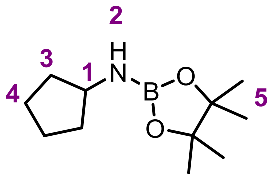

^{1}H NMR (CD_3CN, 400 MHz) *δ 3*.43 (app. sept, *J* = 6.7 Hz, 2H, C^1-H), 2.58 (*br* s, 1H, N^2-H), 1.81 – 1.49 (m, 8H, C^3-H/ C^4-H), 1.17 (s, 12H, C^5-H).

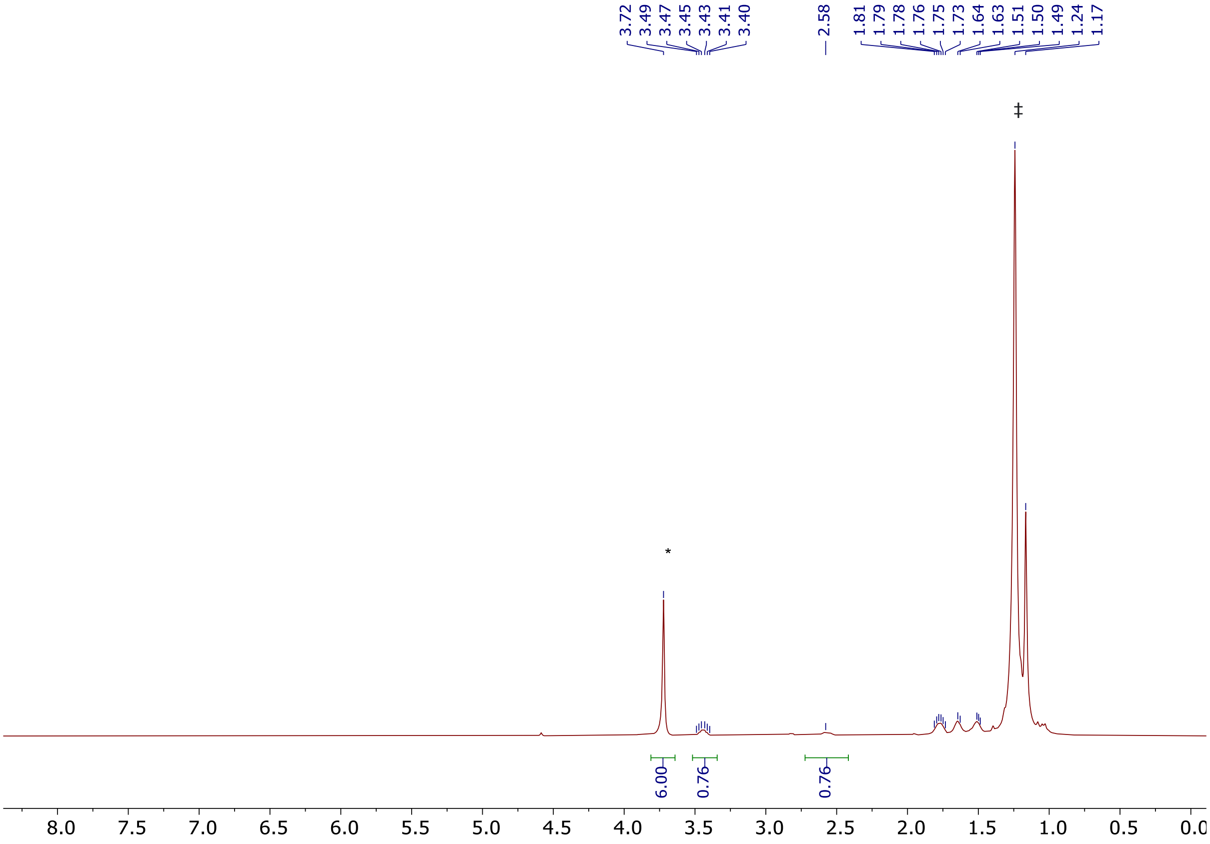

Figure S17: ^{1}H NMR Spectrum of 2r in CD_3CN 16h after the addition of HBpin (singlet at 3.71 corresponds to the dimethyl carbonate internal standard). (Due to overlap with pinBOBpin / broadening certain peaks could not be integrated).

5. Reduction and Isolation of Nitro-Compounds

5.1. General Procedure for the Reduction of Aromatic Nitro Compounds:

To a J-young NMR tube under argon atmosphere was added a mixture of **1a** (0.003 mmol, 2 mg) and nitro-compound (0.3 mmol, 1 equiv.) in CD_3CN (0.6 mL). To the solution was added pinacolborane (1.5 mmol, 0.22 mL, 5 equiv.). The reaction mixture was then left for 10 minutes at room temperature. After the reaction has gone to completion, the reaction mixture was diluted with diethyl ether (2 mL) and quenched with aqueous HCl (0.2 mL, 2M) at 0 °C. The mixture was then left in an ice bath for 30 minutes. The resulting precipitate was filtered and washed with cold dichloromethane to afford the related ammonium salt.

5.1.1. Aniline hydrochloride **3a**[2]

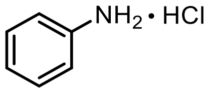

Off white solid (24 mg, 61%)

Analytic data is in accordance with those reported in literature.

^{1}H NMR (D_2O, 400 MHz) δ 7.53 – 7.48 (m. 3H, *Ar*-H), 7.40 – 7.38 (m, 2H, *Ar*-H).

^{13}C{^{1}H} NMR (D_2O, 101 MHz) δ 130.1, 129.6, 129.2, 122.9.

HRMS (ESI+): calcd for $[M, C_6H_7N]^+$ 94.0652, found 94.0653.

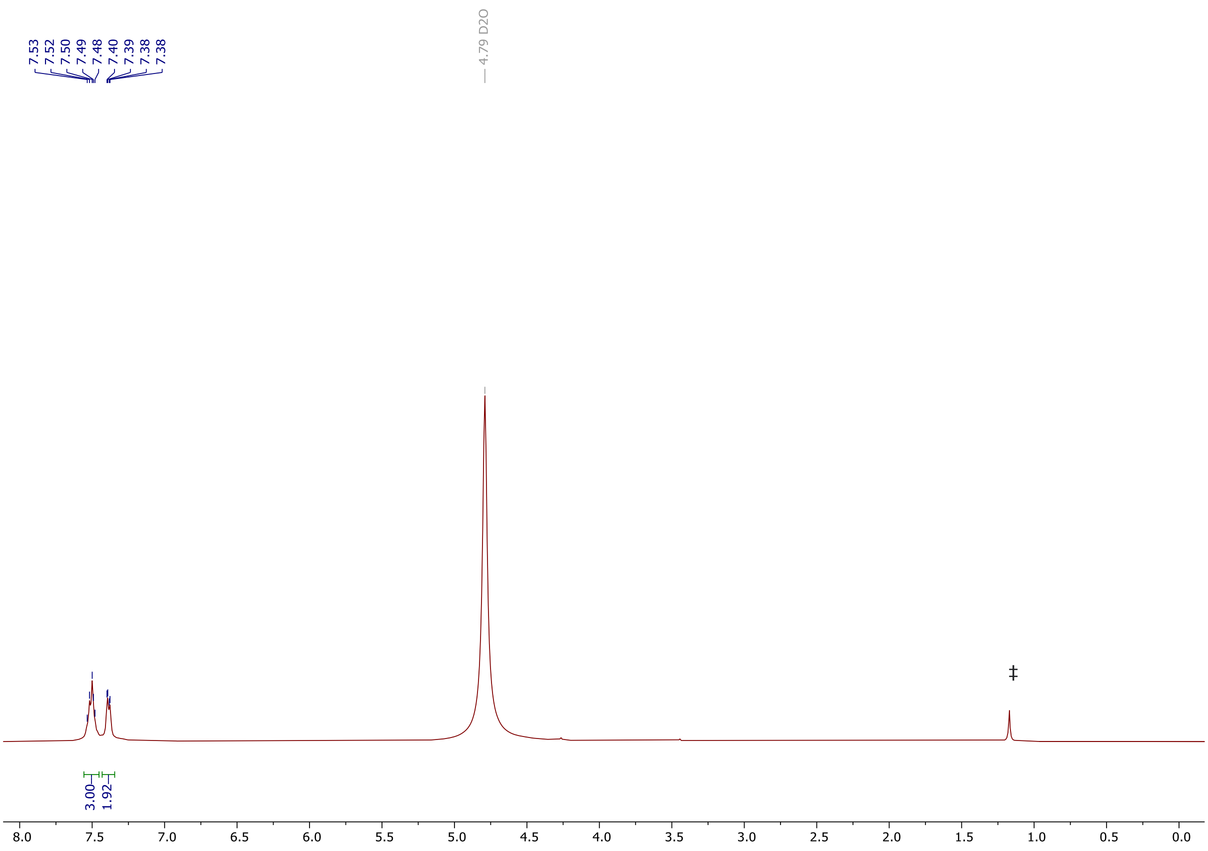

Figure S18: ^{1}H NMR Spectrum of 3a in D_2O after salt formation.

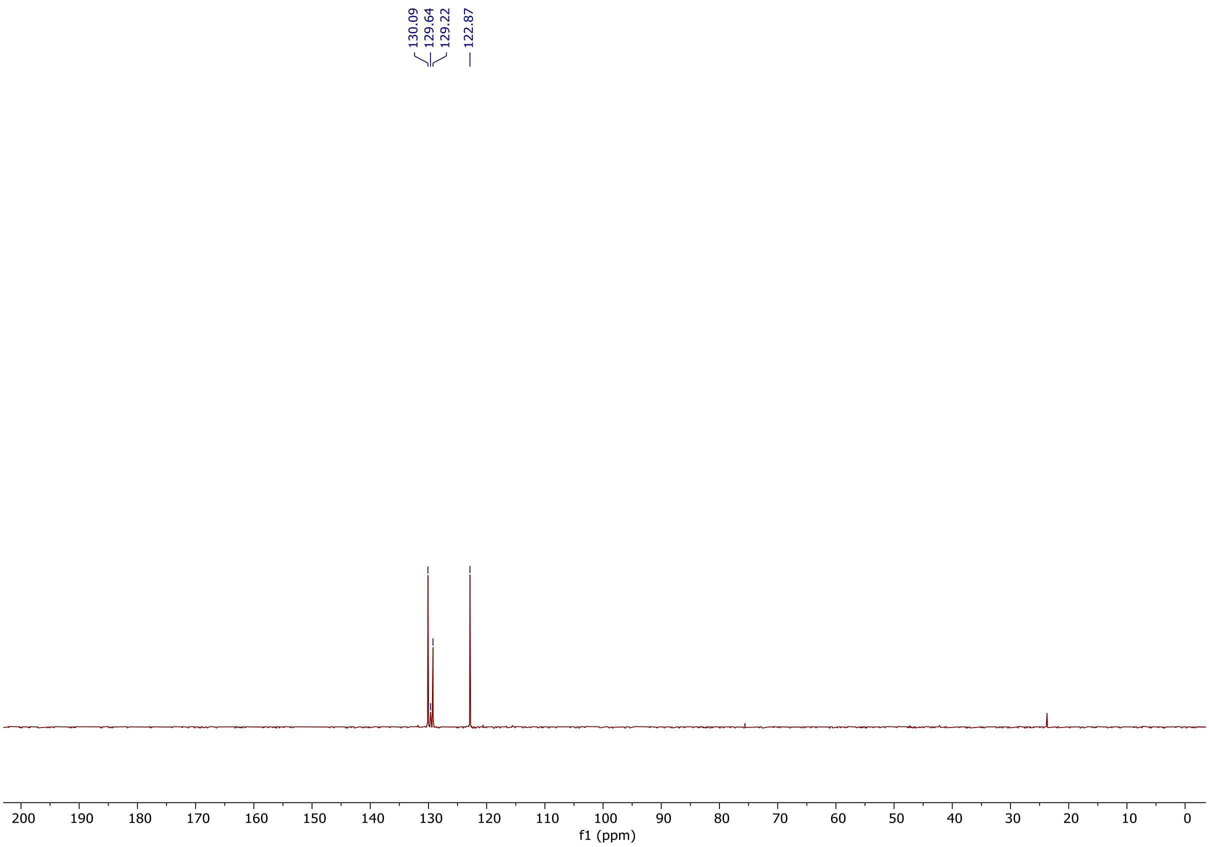

Figure S19: ^{13}C NMR Spectrum of 3a in D_2O after salt formation.

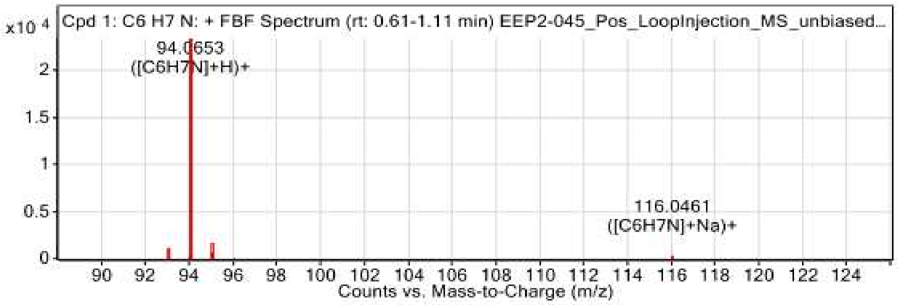

Figure S20: HRMS spectra for compound 3a.

5.1.2. *p*-tert-Butylaniline hydrochloride **3b**

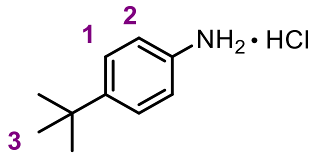

Off white solid (38 mg, 68%)

^{1}H NMR (D_2O, 400 MHz) δ 7.62 (d, J = 8.0 Hz, 2H, C^1-H), 7.36 (d, J = 8.8 Hz, 2H, C^2-H), 1.31 (s, 9H, C^3-H).

^{13}C{^{1}H} NMR (D_2O, 101 MHz) δ 152.9, 127.1, 127.1, 122.5, 34.1, 30.1.

HRMS (ESI+): calcd for [M, $C_{10}H_{16}N$]$^+$ 150.1277, found 150.1281.

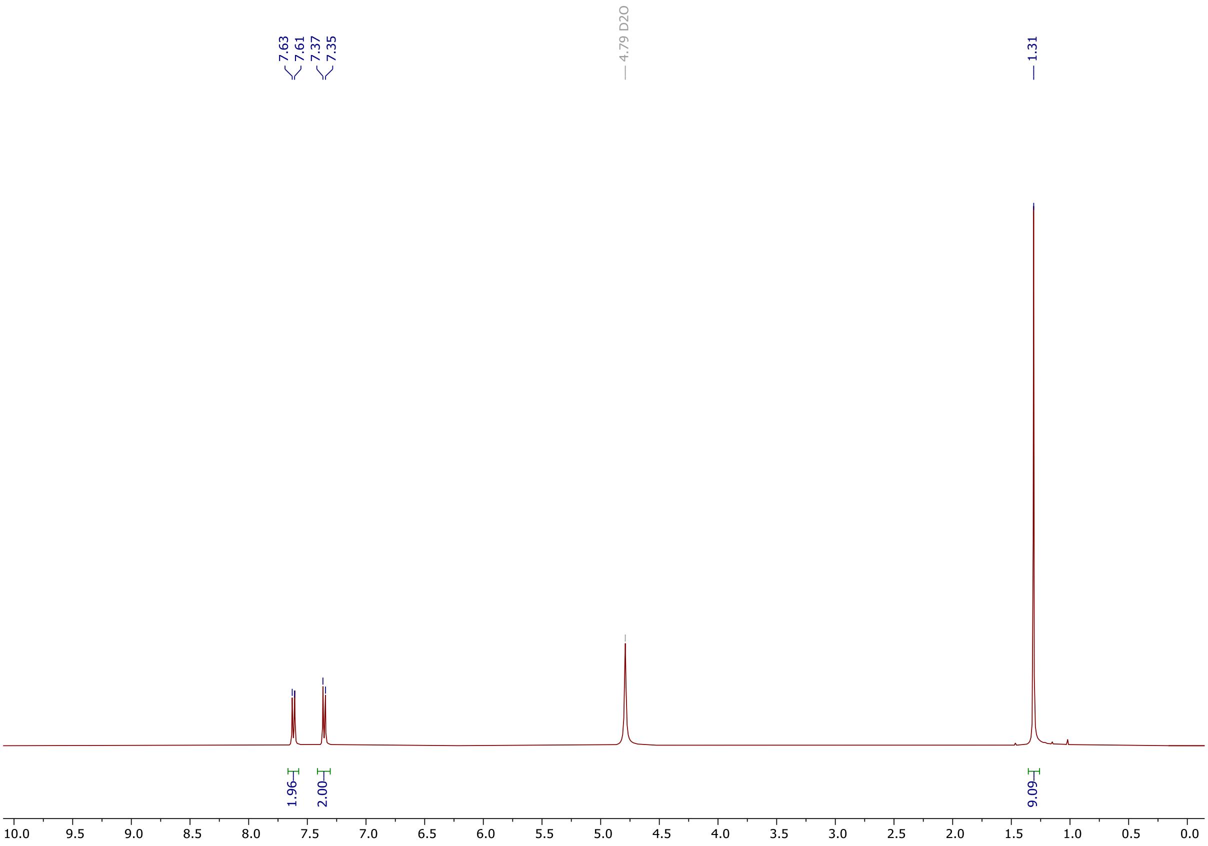

Figure S21: ^{1}H NMR Spectrum of 3b in D_2O after salt formation.

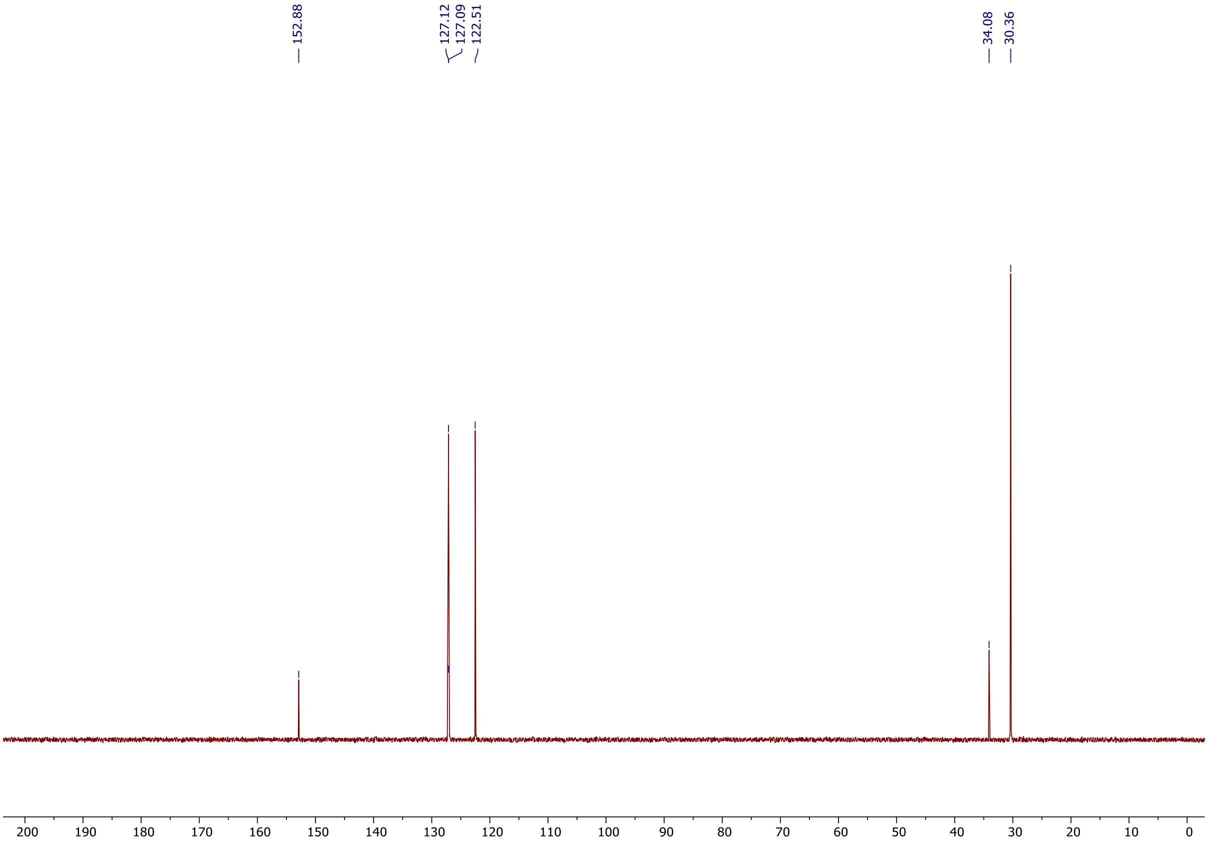

Figure S22: ^{13}C NMR Spectrum of 3b in D_2O after salt formation.

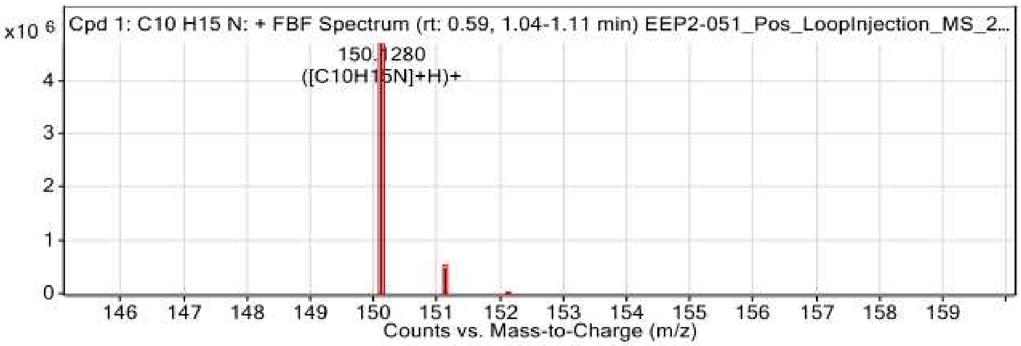

Figure S23: HRMS spectra for compound 3b.

5.1.3. 4-dimethylaminophenylamine dihydrochloride **3c**

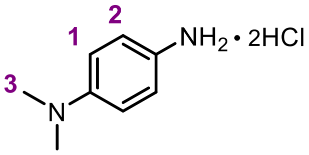

Dark purple solid (21 mg, 41%)

^{1}H NMR (D_2O, 400 MHz) δ 7.71 (d, J = 9.1, 2H, C^1-H), 7.53 (d, J = 9.1, 2H, C^2-H), 3.28 (s, 6H, C^3-H).

^{13}C{^{1}H} NMR (D_2O, 101 MHz) δ 141.4, 124.4, 122.3, 46.3, 23.7.

HRMS (ESI+): calcd for [M, $C_8H_{12}N_2$]$^+$ 137.1074, found 137.1083.

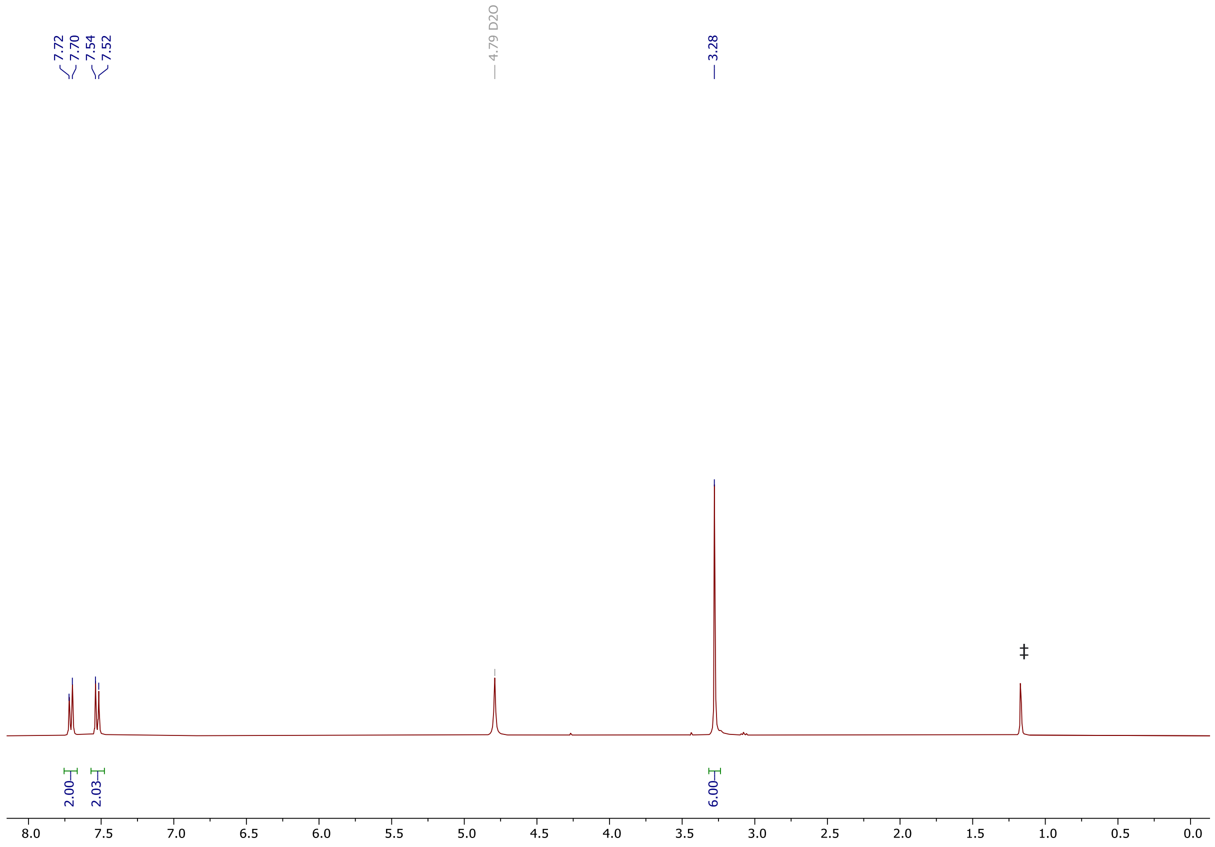

Figure S24: ^{1}H NMR Spectrum of 3c in D_2O after salt formation.

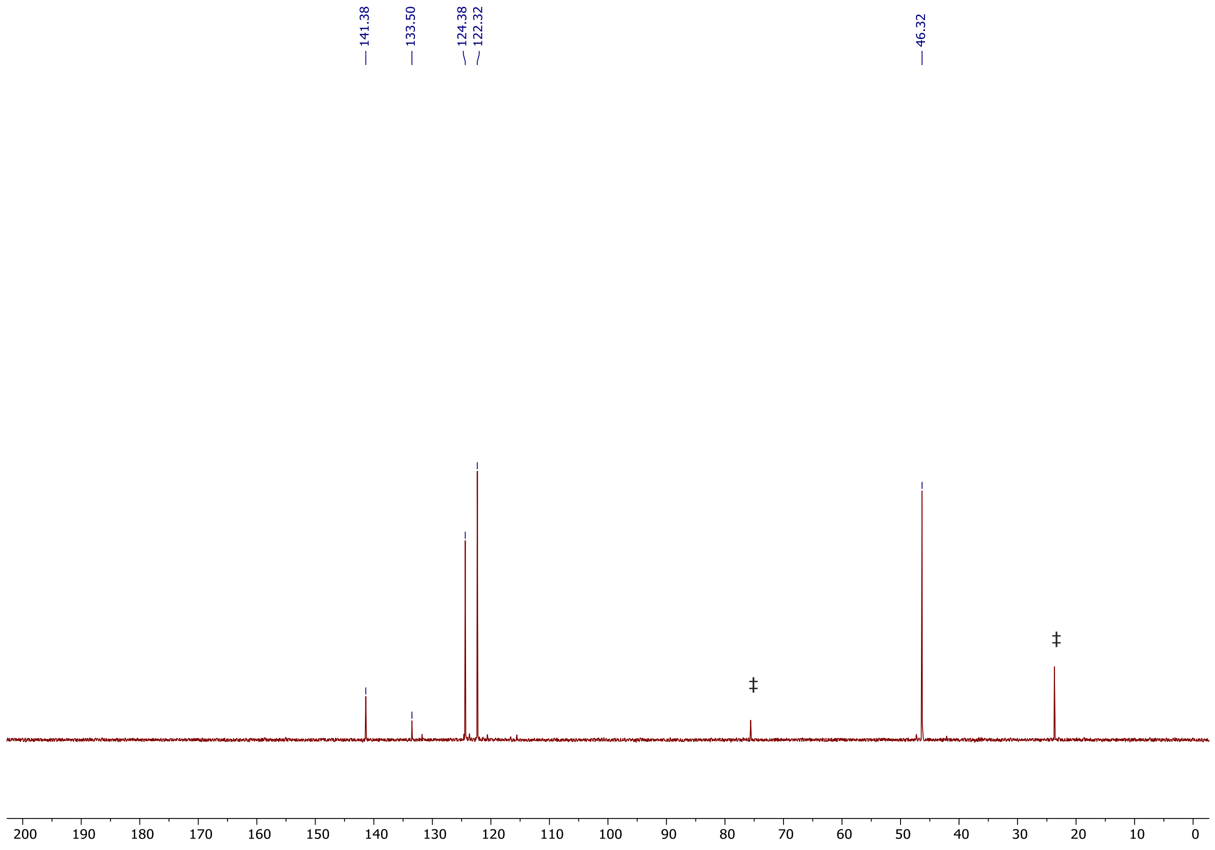

*Figure S25: ^{13}*C NMR Spectrum of 3c in D_2O after salt formation.

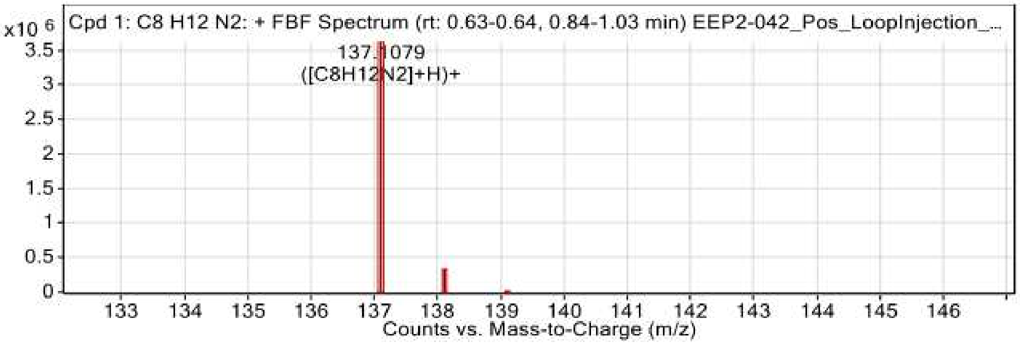

Figure S26: HRMS spectra for compound 3c.

5.1.4. 4-(methylthio)aniline hydrochloride **3d**

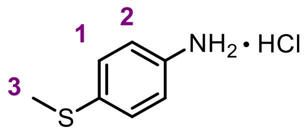

Pale yellow solid (42 mg, 79%)

^{1}H NMR (D_2O, 400 MHz) δ 7.44 (d J = 8.8 Hz, 2H, C^1-H), 7.35 (d J = 8.8, 2H, C^2-H), 2.53 (s, 3H, C^3-H).

^{13}C{^{1}H} NMR (D_2O, 101 MHz) δ 139.3, 127.3, 127.1, 123.3, 14.4.

HRMS (ESI+): calcd for [M, C_7H_9NS]$^+$ 140.0528, found 140.0532.

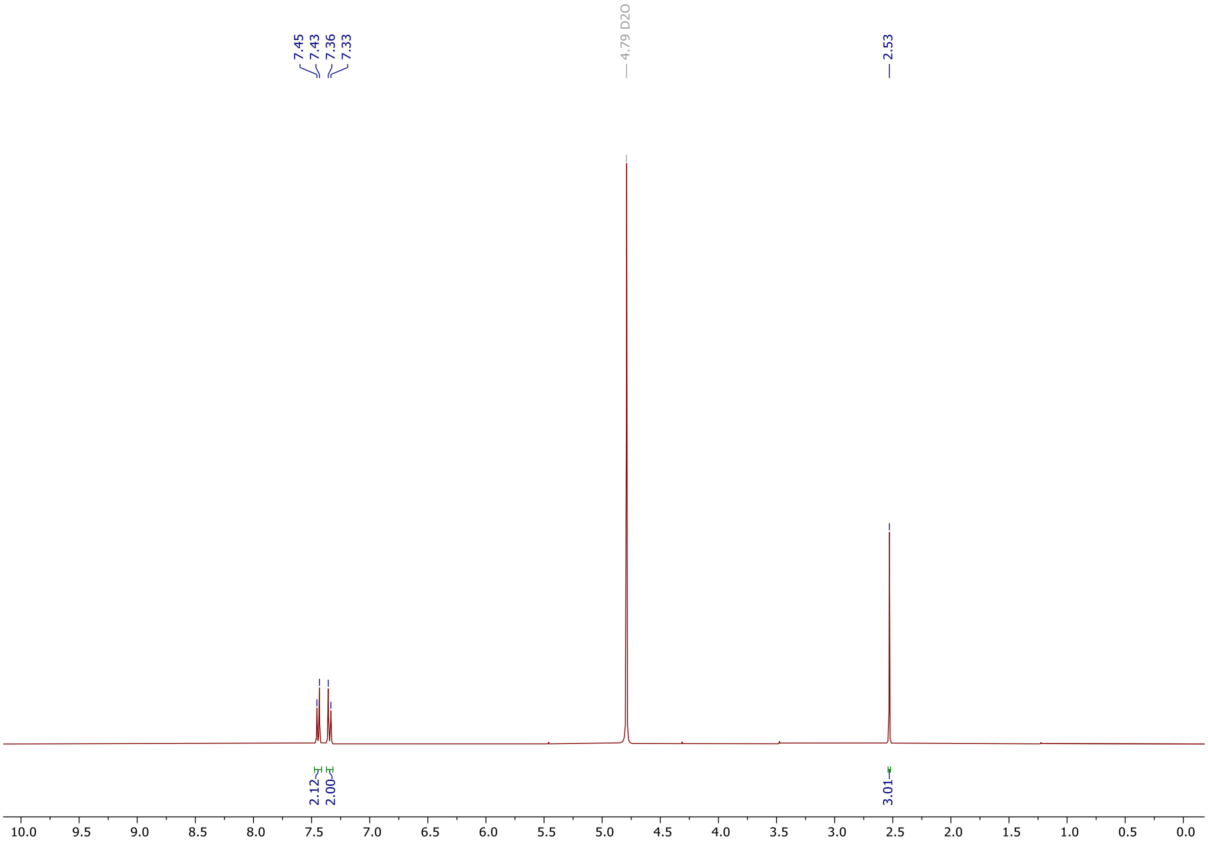

Figure S27: ^{1}H NMR Spectrum of 3d in D_2O after salt formation.

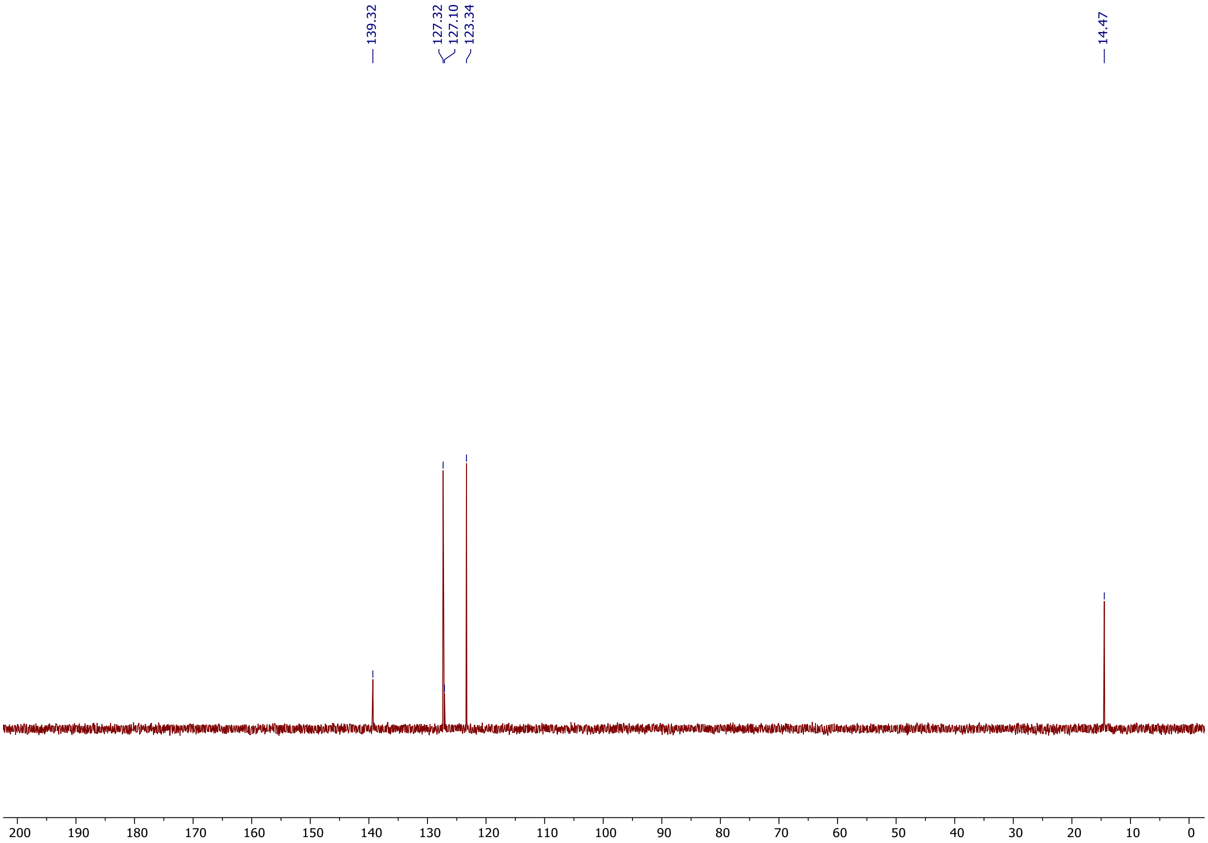

Figure S28: ^{13}C NMR Spectrum of 3d in D_2O after salt formation.

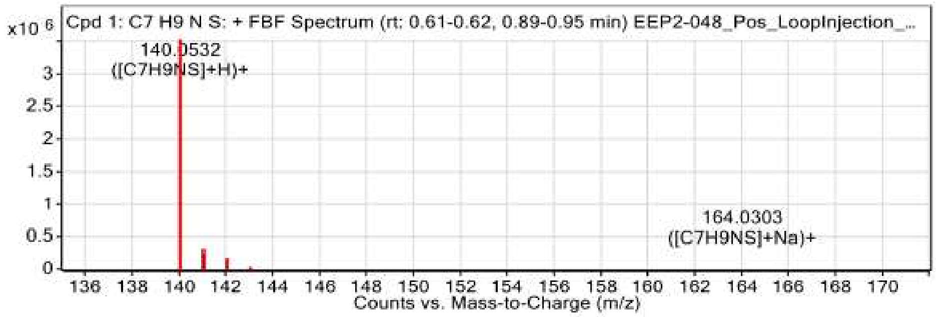

Figure S29: HRMS spectra for compound 3d.

5.1.5. 4-amino-thiophenol hydrochloride **3e**

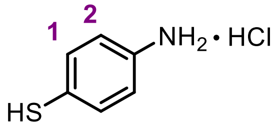

Required 6 equivalents of HBpin and the reaction to be heated at 50 °C for 16h.

white solid (43 mg, 88%)

^{1}H NMR (D_2O, 400 MHz) δ 7.69 (d, J = 8.7 Hz, 2H, C^1-H), 7.35 (d, J = 8.9 Hz, 2H, C^2-H).

^{13}C{^{1}H} NMR (D_2O, 101 MHz) δ 136.8, 130.0, 129.1, 123.6.

HRMS (ESI+): calcd for [M, C_6H_7NS]$^+$ 126.0372, found 126.0371.

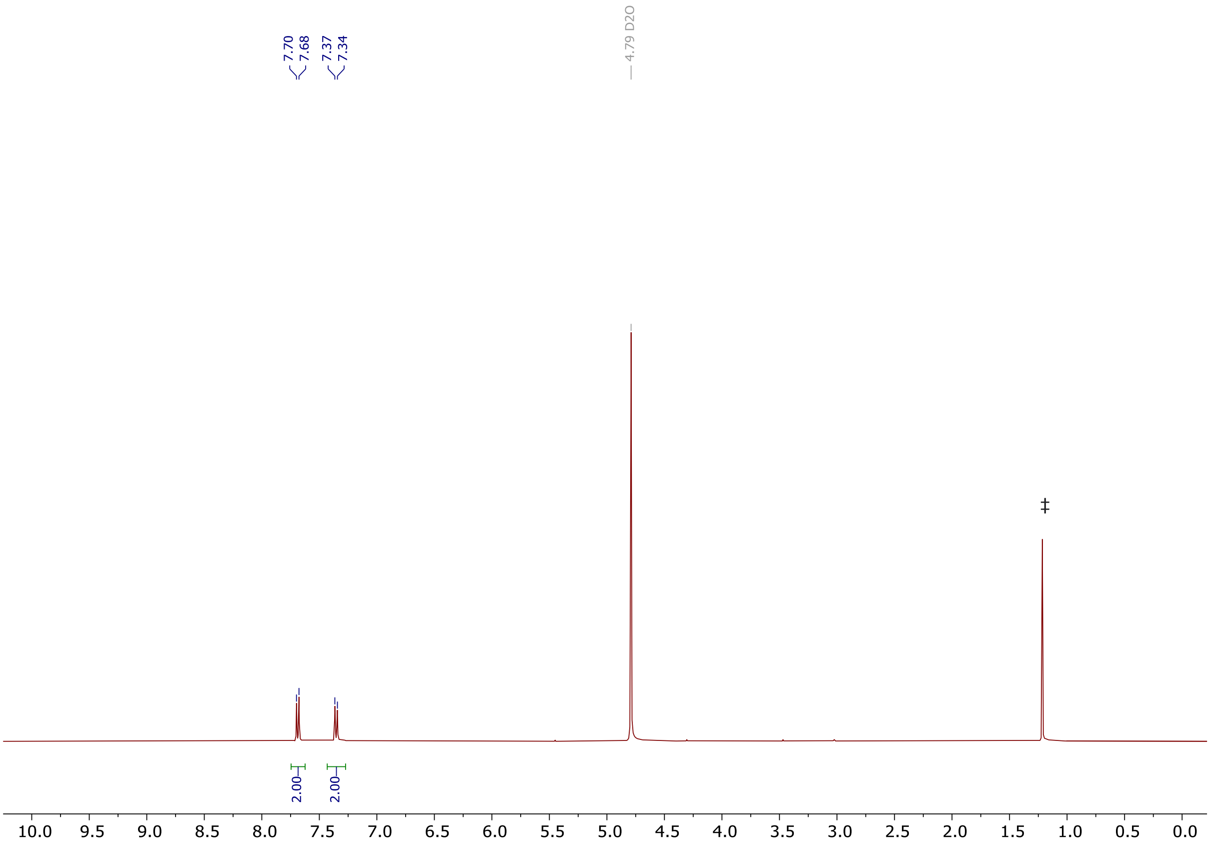

Figure S30: ^{1}H NMR Spectrum of 3e in D_2O after salt formation.

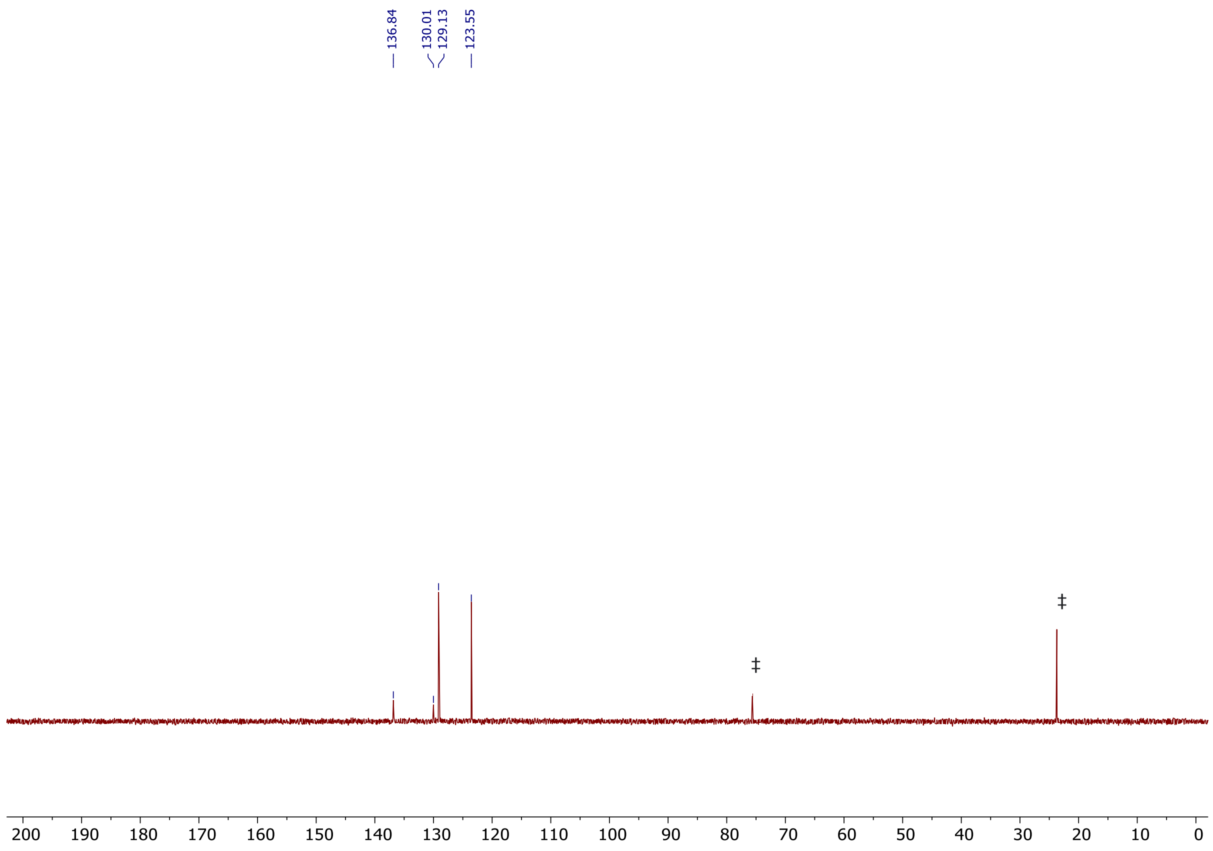

Figure S31: ^{13}C NMR Spectrum of 3e in D_2O after salt formation.

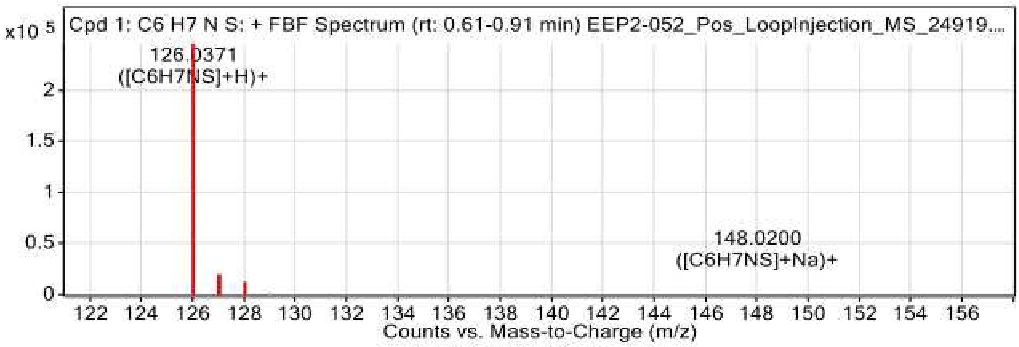

Figure S32: HRMS spectra for compound 3e.

5.1.6. 4-bromoaniline hydrochloride **3f**[2]

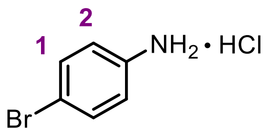

6 equivalents of HBpin were required along with 2 mol% **1a**, the reaction mixture was maintained at 80 °C for 3 days.

Off white solid (39 mg, 63%)

Analytic data is in accordance with those reported in literature.

^{1}H NMR (D_2O, 400 MHz) δ 7.72 (d, J = 8.9 Hz, 2H, C^1-H), 7.32 (d, J = 8.9 Hz, 2H, C^2-H).

^{13}C{^{1}H} NMR (D_2O, 101 MHz) δ 133.1, 129.6, 124.6, 122.0.

HRMS (ESI+): calcd for [M, C_6H_7NBr]$^+$ 171.9756, found 171.9760.

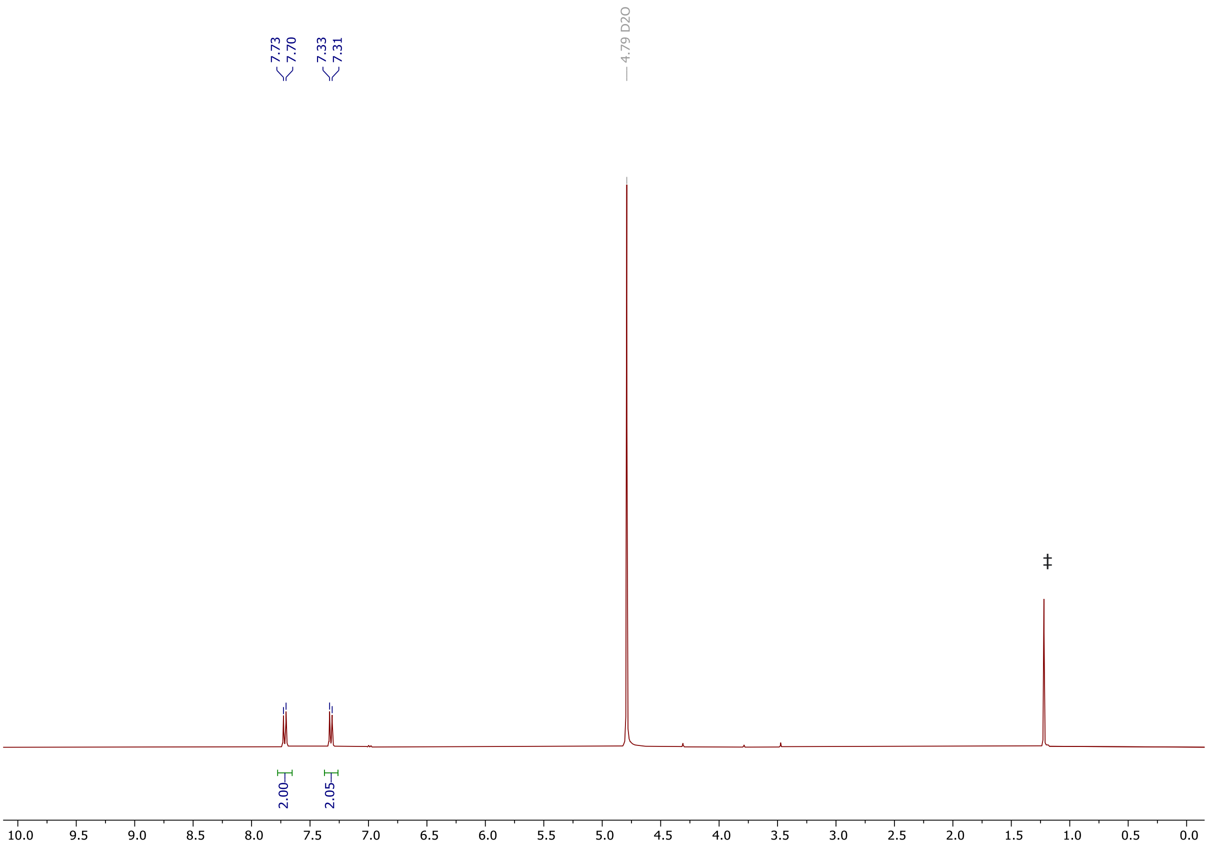

Figure S33: ^{1}H NMR Spectrum of 3f in D_2O after salt formation.

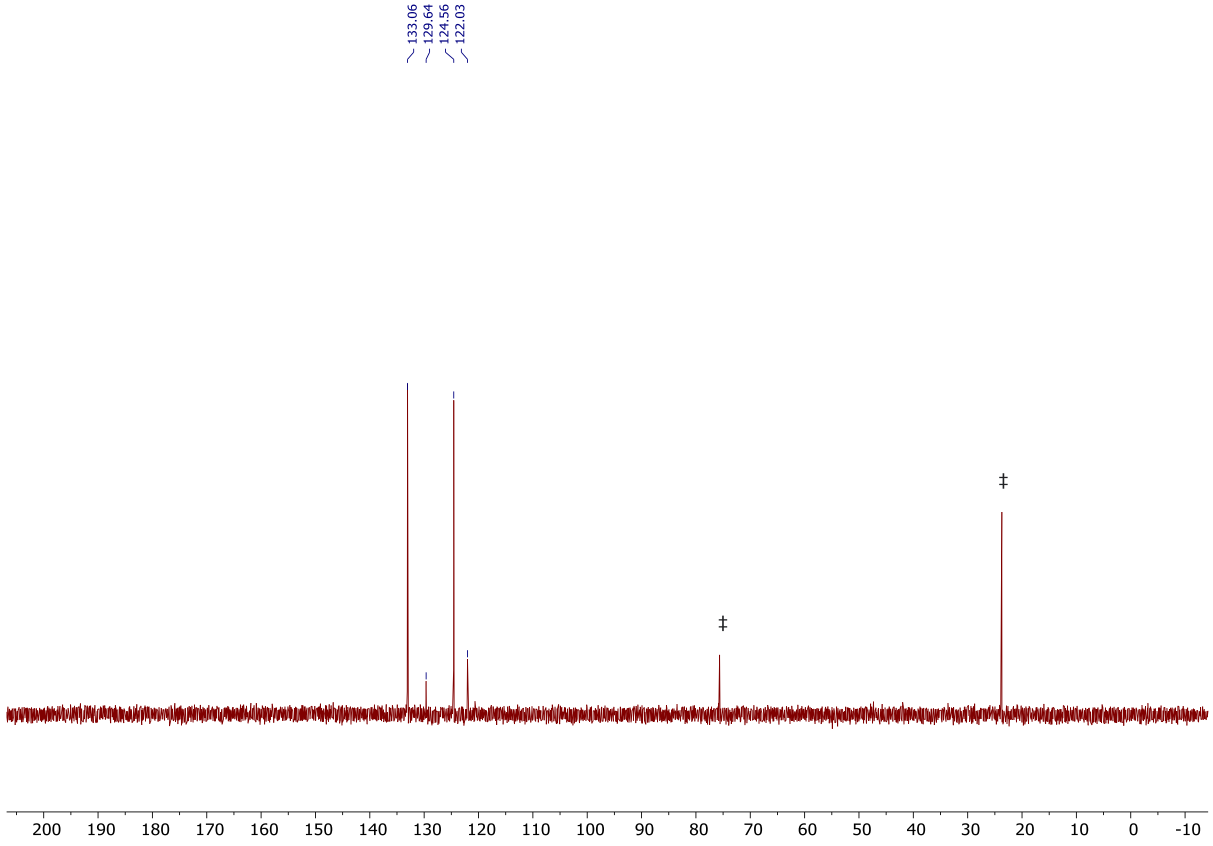

Figure S34: ^{13}C NMR Spectrum of 3f in D_2O after salt formation.

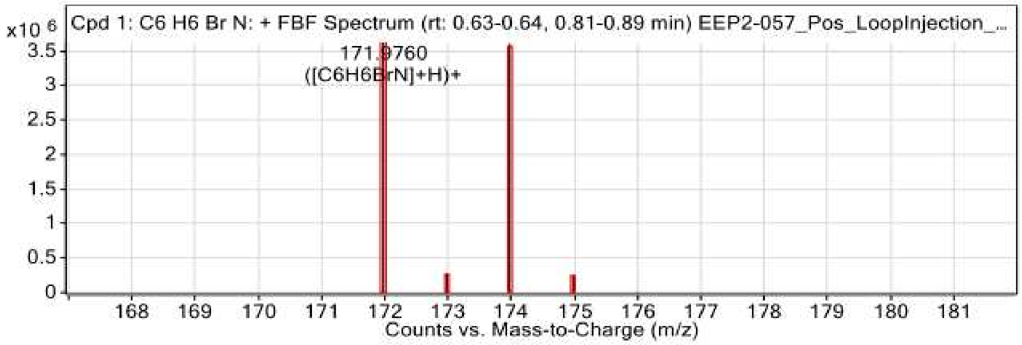

Figure S35: HRMS spectra for compound 3f.

5.1.7. 4'-aminocetophenone hydrochloride **3g**[2]

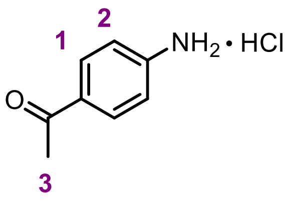

Required 6 equivalents of HBpin for reaction to go to completion.

Bright yellow solid (34 mg, 65%)

Analytic data is in accordance with those reported in literature.

^{1}H NMR (D_2O, 400 MHz) δ 8.07 (d, J = 8.9 Hz, 2H, C^1-H), 7.41 (d, J = 8.7 Hz, 2H, C^2-H), 2.65 (s, 3H, C^3-H).

$^{13}C\{^1H\}$ NMR (D_2O, 101 MHz) δ 202.4, 138.2, 134.9, 130.6, 121.6 26.1.

HRMS (ESI+): calcd for [M, C_8H_9NO]$^+$ 136.0757, found 136.0759.

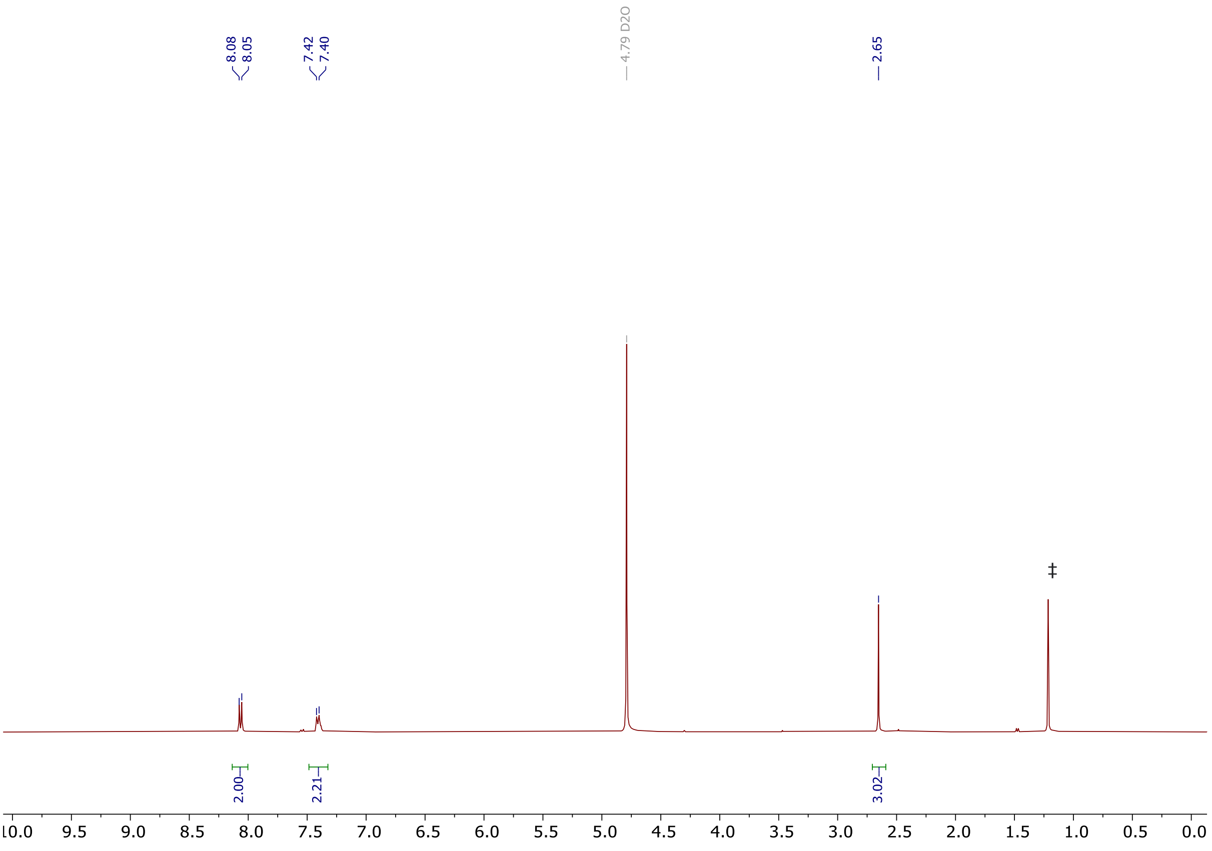

Figure S36: ^{1}H NMR Spectrum of 3g in D_2O after salt formation.

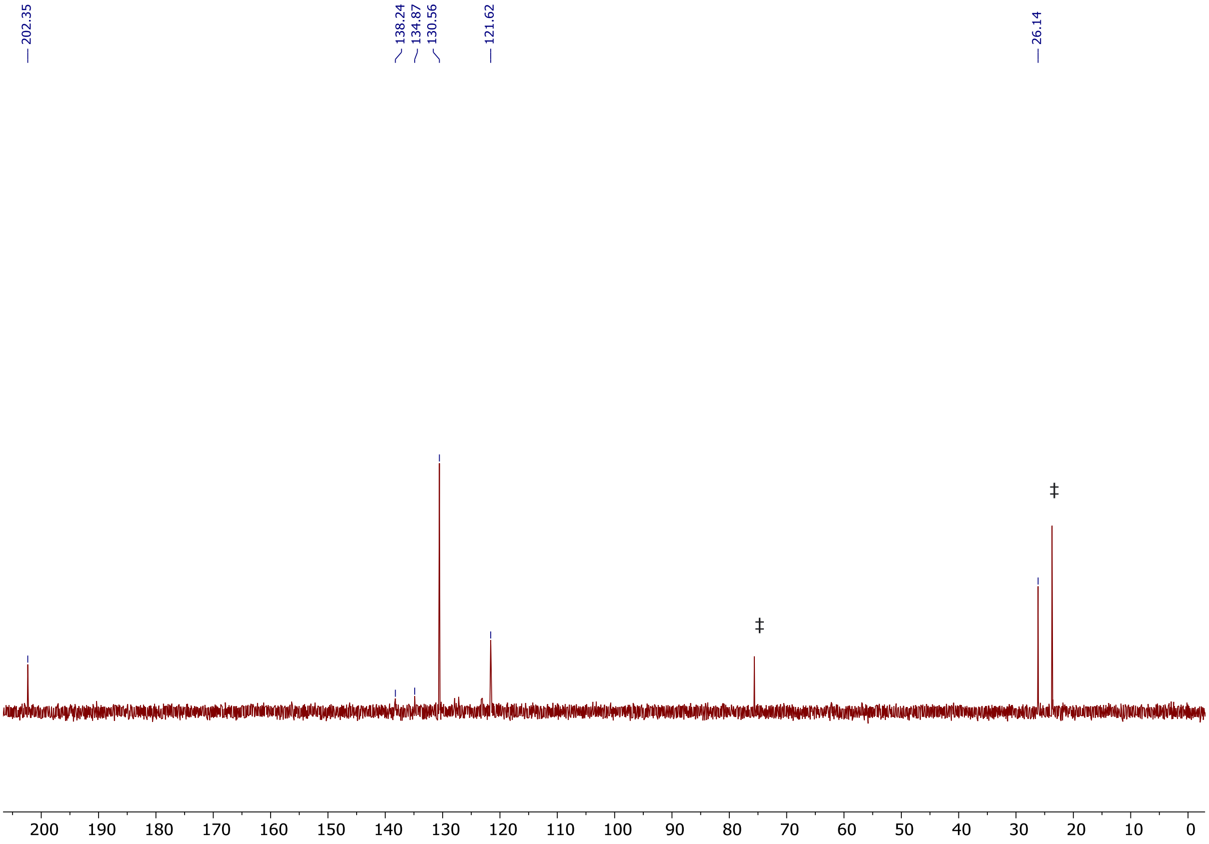

Figure S37: ^{13}C NMR Spectrum of 3g in D_2O after salt formation.

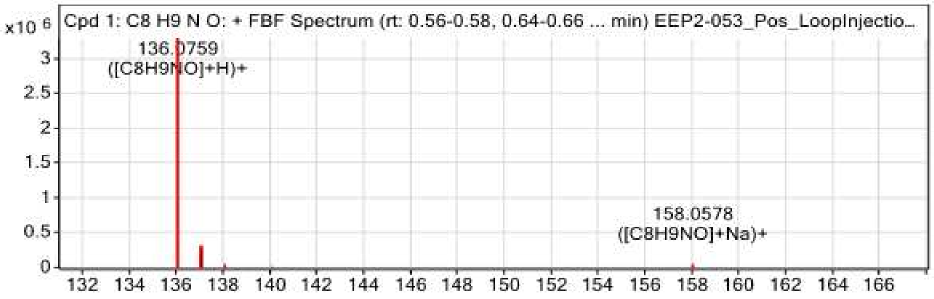

Figure S38: HRMS spectra for compound 3g.

5.1.8. (4-aminophenyl)-methanol hydrochloride **3h**

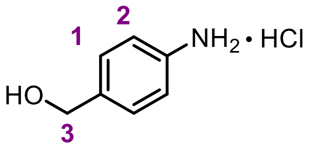

6 equivalents of HBpin were required for the reaction to go to completion.

Yellow solid (28 mg, 75%)

^{1}H NMR (D_2O, 400 MHz) δ 7.51 (d, J = 8.6 Hz, 2H, C^1-H), 7.40 (d, J = 8.6 Hz, 2H, C^2-H), 4.66 (s, 2H, C^3-H).

^{13}C{^{1}H} NMR (D_2O, 101 MHz) δ 131.5, 128.8, 123.5, 123.1, 63.0.

HRMS (ESI+): calcd for [M, $C_7H_{10}NO$]$^+$ 124.0757, found 124.0759.

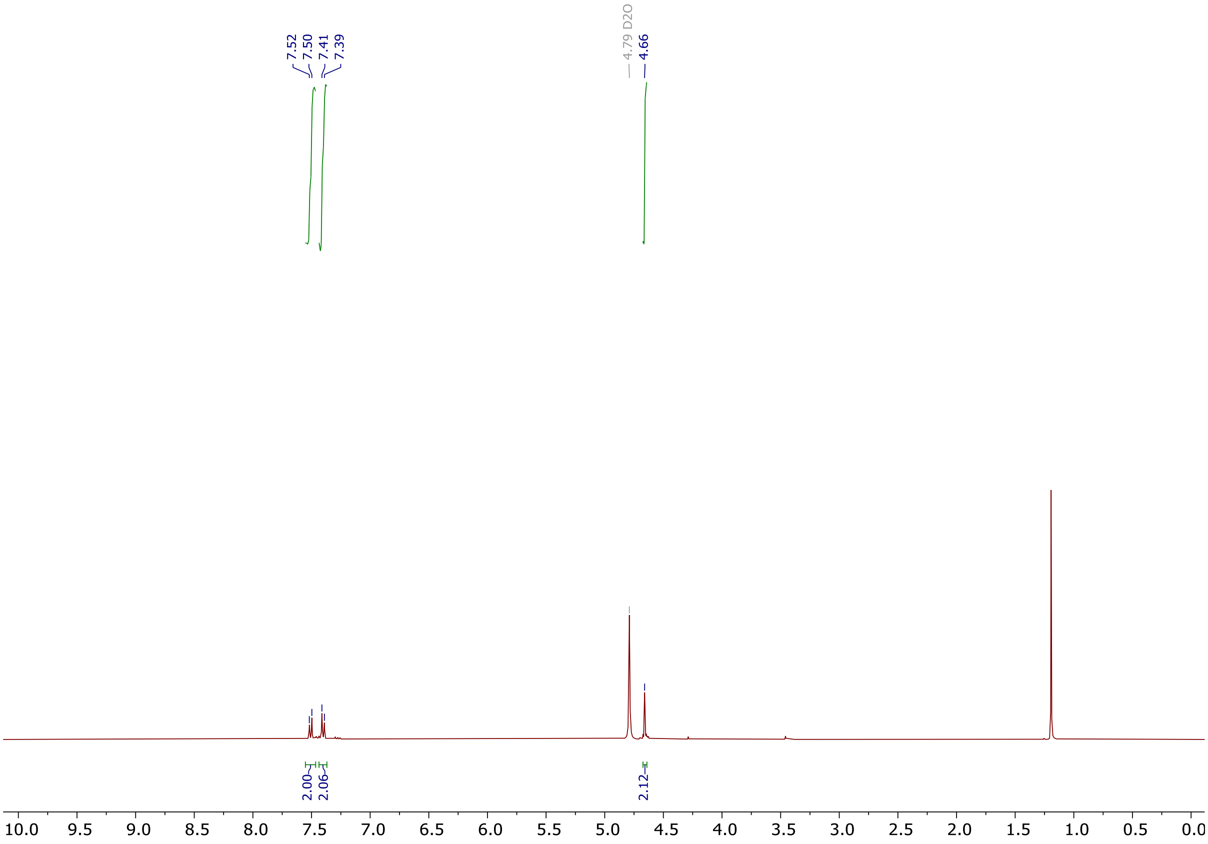

Figure S39: ^{1}H NMR Spectrum of 3h in D_2O after salt formation.

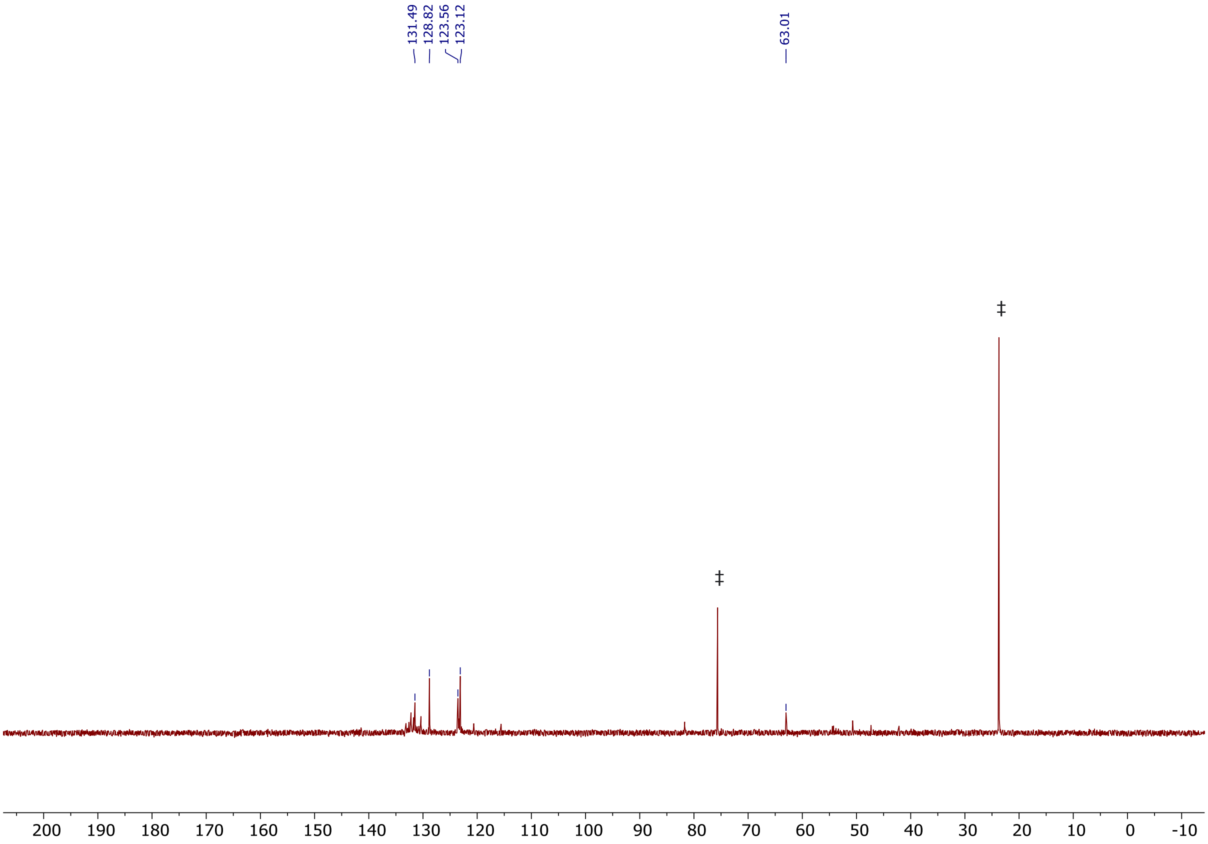

Figure S40: ^{13}C NMR Spectrum of 3h in D_2O after salt formation.

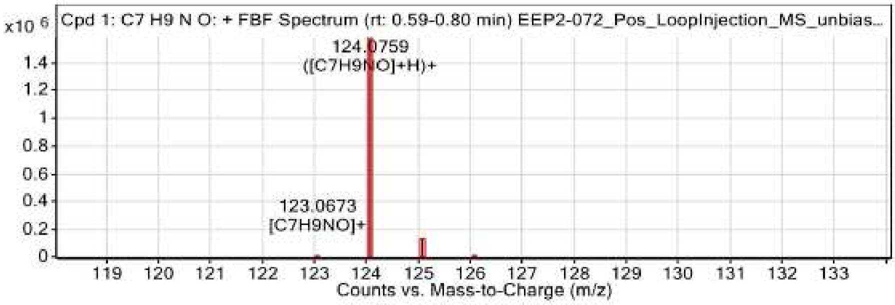

Figure S41: HRMS spectra for compound 3h.

5.1.9. *m*-anisidine hydrochloride **3i**

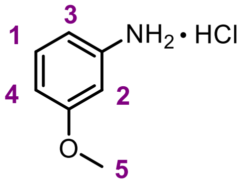

Off white solid (36 mg, 75%)

^{1}H NMR (D_2O, 400 MHz) δ 7.49 (app. t, J = 8.2 Hz, 1H, C^1-H), 7.11 (dd, J = 8.1, J =2.9 Hz, 1H, C^3-H), 7.03-6.98 (m, 2H, C^2-H/ C^4-H), 3.88 (s, 3H, C^5-H).

^{13}C{^{1}H} NMR (D_2O, 101 MHz) δ 160.0, 131.3, 131.2, 115.1, 114.1, 108.9, 55.6.

HRMS (ESI+): calcd for [M, C_7H_9NO]$^+$ 124.0757, found 124.0759.

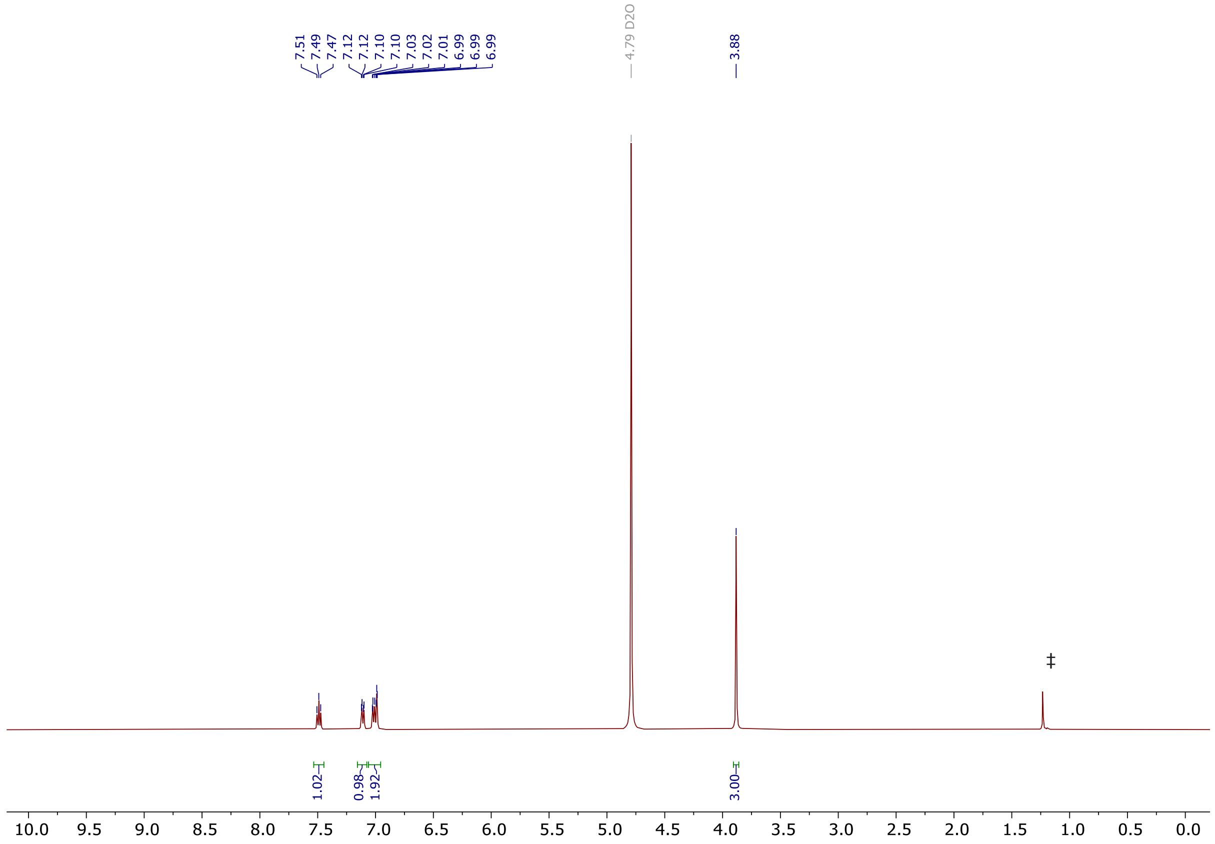

Figure S42: ^{1}H NMR Spectrum of 3i in D_2O after salt formation.

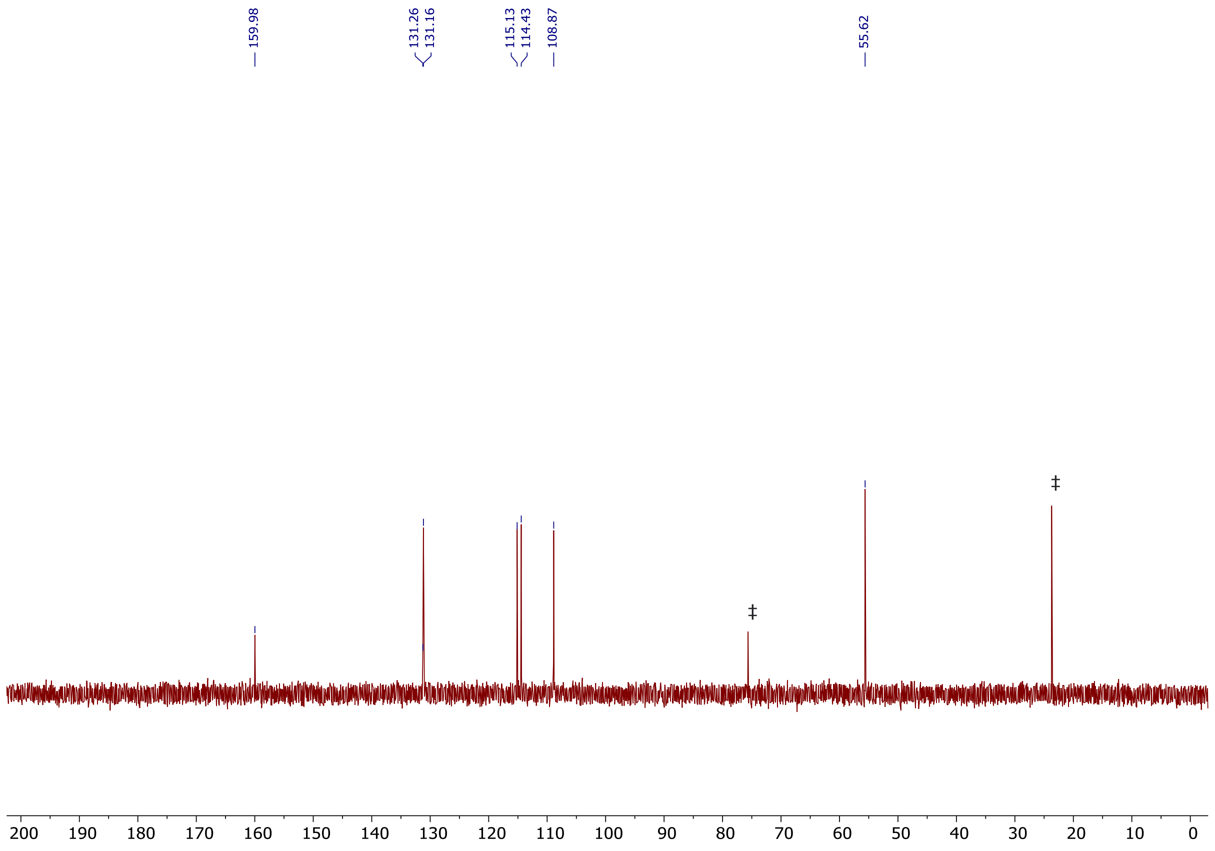

Figure S43: ^{13}C NMR Spectrum of 3i in D_2O after salt formation.

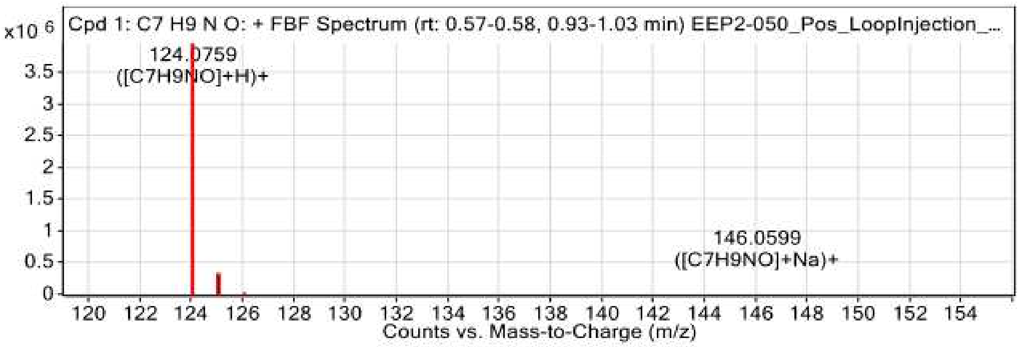

Figure S44: HRMS spectra for compound 3i.

5.1.10. 3-aminophenol hydrochloride **3j**[2]

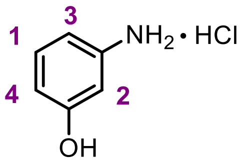

Required 6 equivalents of HBpin and the reaction to be heated at 50 °C for 16h.

white solid (31 mg, 71%)

Analytic data is in accordance with those reported in literature.

^{1}H NMR (D_2O, 400 MHz) δ 7.41 (app. t, J = 8.1 Hz, 1H, C^1-H), 6.99 (app. d, J = 8.3 Hz, 1H, C^3-H), 6.94 (d, J = 7.9 Hz, 1H, C^4-H), 6.89 (app. t, J = 2.2 Hz, 1H, C^2-H).

$^{13}C\{^1H\}$ NMR (D_2O, 101 MHz) δ 156.8, 131.3, 131.1, 116.0, 114.5, 110.0.

HRMS (ESI+): calcd for [M, C_6H_7NO]$^+$ 110.0601, found 110.0605.

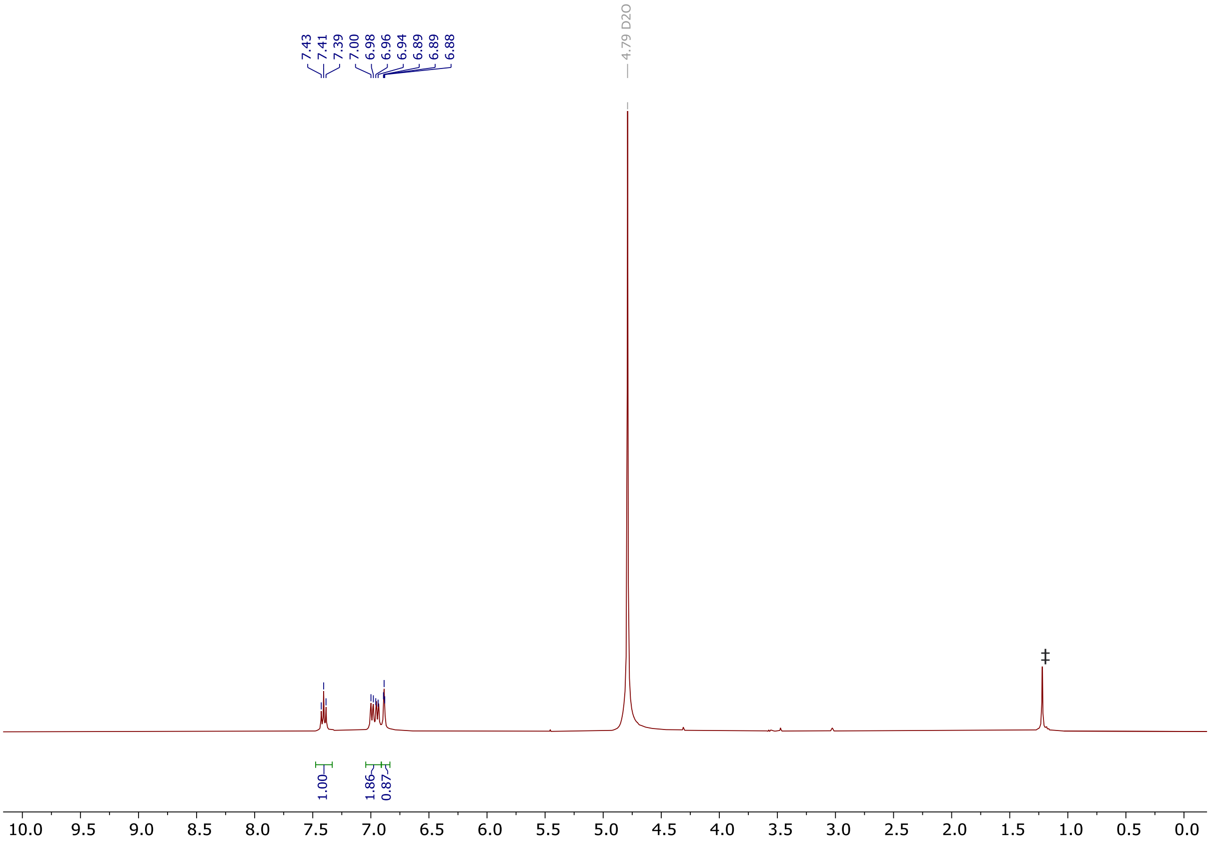

Figure S45: ^{1}H NMR Spectrum of 3j in D_2O after salt formation.

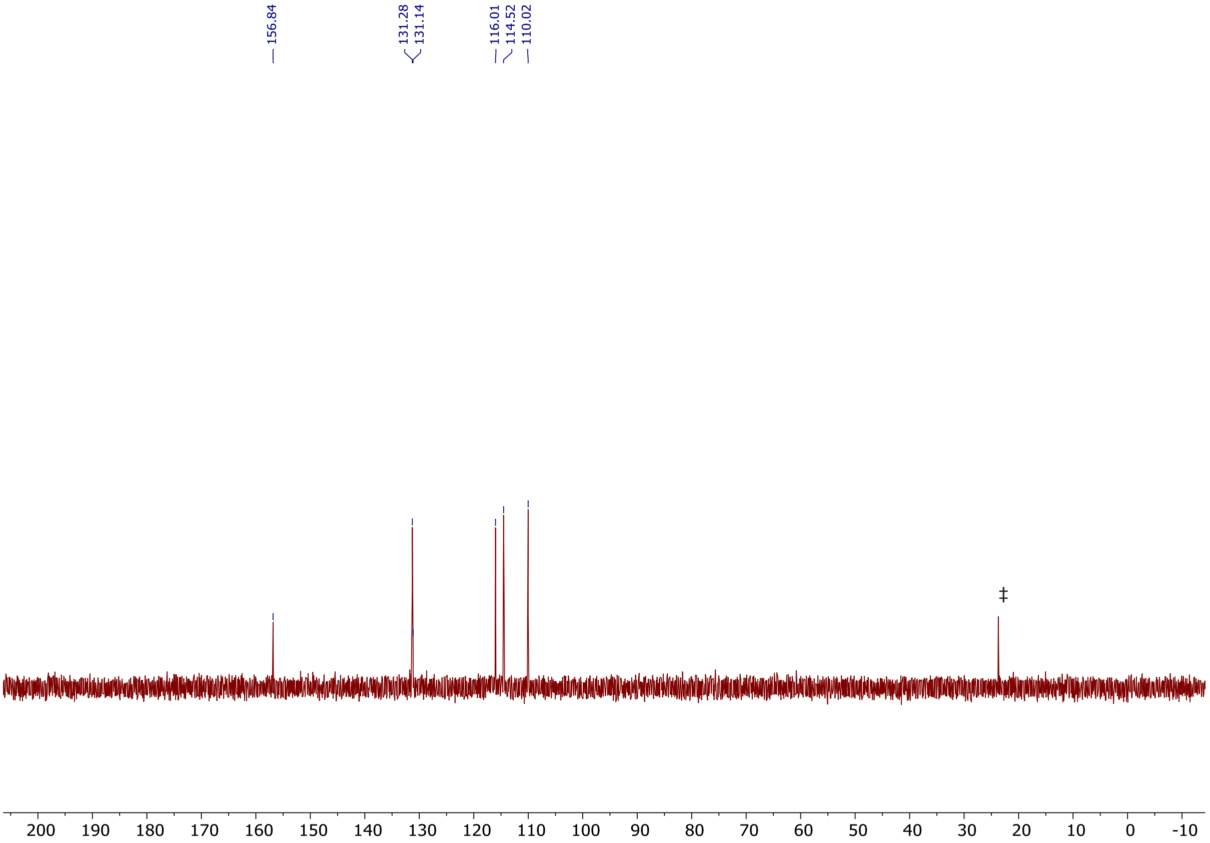

Figure S46: ^{13}C NMR Spectrum of 3j in D_2O after salt formation.

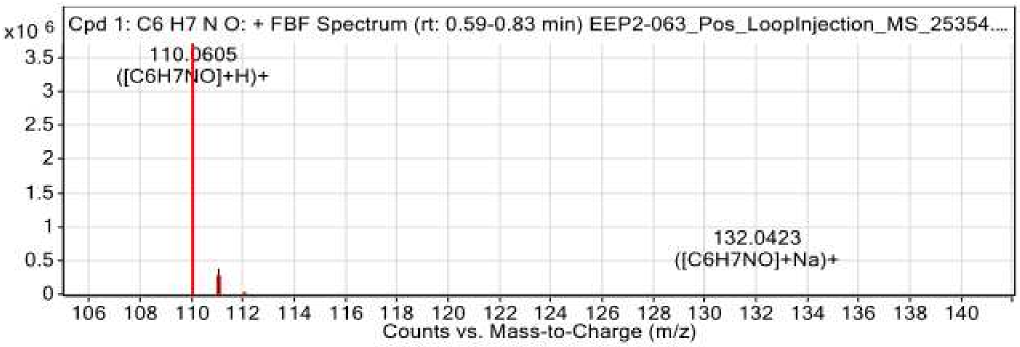

Figure S47: HRMS spectra for compound 3j.

5.1.11. 5-amino-1H-indole **3k**[3]

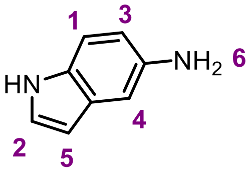

6 equivalents of HBpin were required for the reaction to go to completion and the product was isolated via column chromatography on basified alumina (2:8 hexane: chloroform).

brown solid (17 mg, 42%)

Analytic data is in accordance with those reported in literature.

^{1}H NMR (CDCl$_3$, 400 MHz) δ 7.97 (*br* s, 1H, NH), 7.20 (d, J = 8.5 Hz, 1H, C^1-H), 7.13 (app. t, J = 2.8 Hz, 1H, C^2-H), 6.95 (d, J = 2.9 Hz, 1H, C^4-H), 6.67 (dd, J = 8.54, J = 2.4 Hz, 1H, C^3-H), 6.38 (app. t, J = 2.7 Hz, 1H, C^5-H), 3.51 (*br* s, 2H, N-H^6).

^{13}C{^{1}H} NMR (CDCl$_3$, 101 MHz) δ 139.7, 130.8, 128.9, 124.8, 113.1, 111.6, 105.7, 101.7.

HRMS (ESI+): calcd for [M, $C_8H_9N_2$]$^+$ 133.0760, found 133.0765.

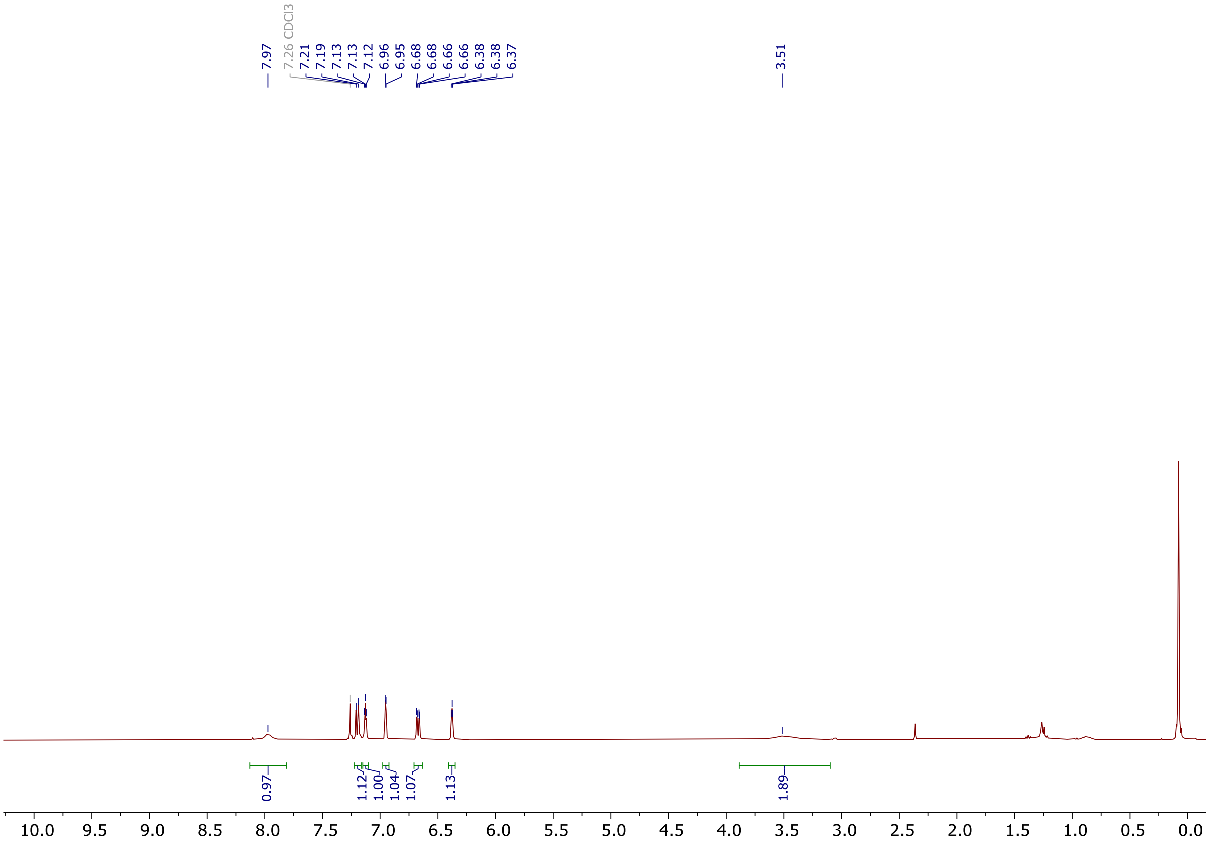

Figure S48: ^{1}H NMR Spectrum of 3k in $CDCl_3$ after isolation via column chromatography.

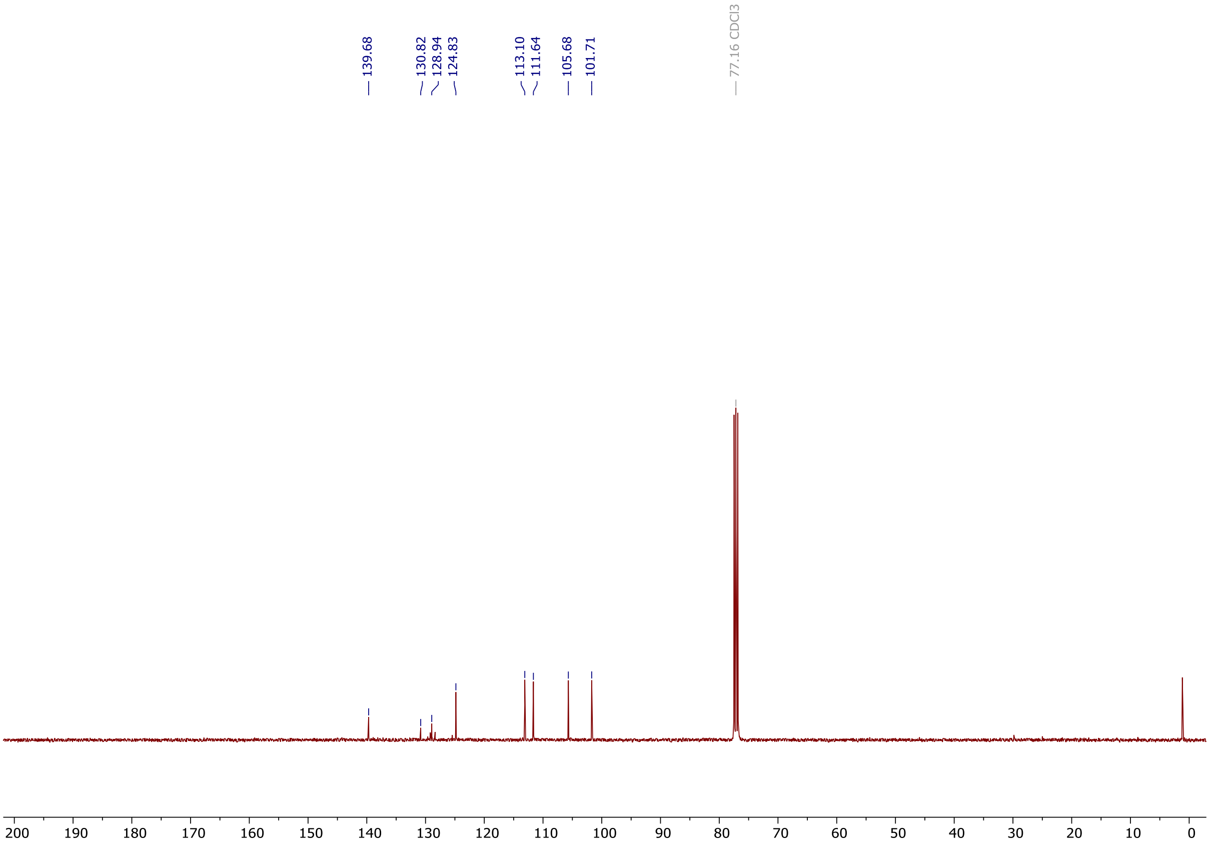

Figure S 49: ^{13}C NMR Spectrum of 3k in $CDCl_3$ after isolation via column chromatography.

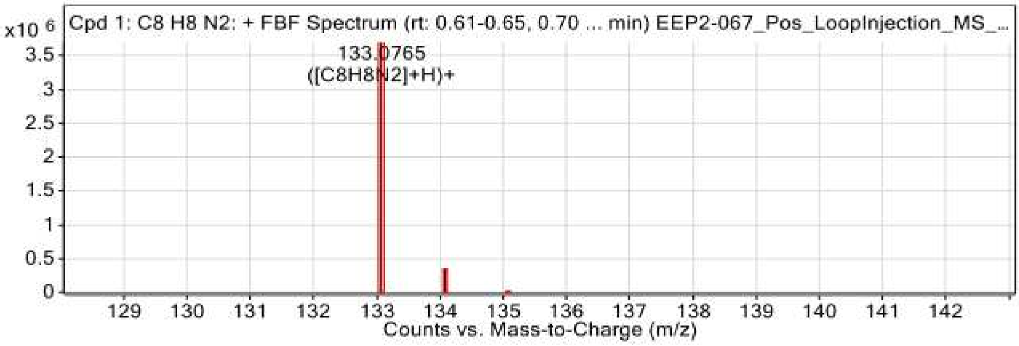

Figure S50: HRMS spectra for compound 3k.

5.2. General Procedure for the Reduction of Aliphatic Nitro Compounds:

To a J-Young NMR tube under argon atmosphere was added a mixture of **1a** (0.003 mmol, 2 mg) and nitro-compound (0.3 mmol, 1 equiv.) in deuterated acetonitrile (0.6 mL). To the solution was added pinacolborane (1.5 mmol, 0.22 mL, 5 equiv.). The reaction mixture was then left for 12h at room temperature. After the reaction has gone to completion, the reaction mixture was diluted with diethyl ether (2 mL) and quenched with aqueous HCl (0.2 mL, 2M) at 0 °C. The mixture was then left in an ice bath for 30 minutes. The resulting precipitate was filtered and washed with cold dichloromethane to afford the related ammonium salt.

5.2.1. methylamine hydrochloride 3l[4]

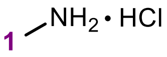

white solid (14 mg, 68%)

Analytic data is in accordance with those reported in literature.

^{1}H NMR (D_2O, 400 MHz) δ 2.59 (s, 3H, C^1-H).

$^{13}C\{^1H\}$ NMR (D_2O, 101MHz) δ 24.5.

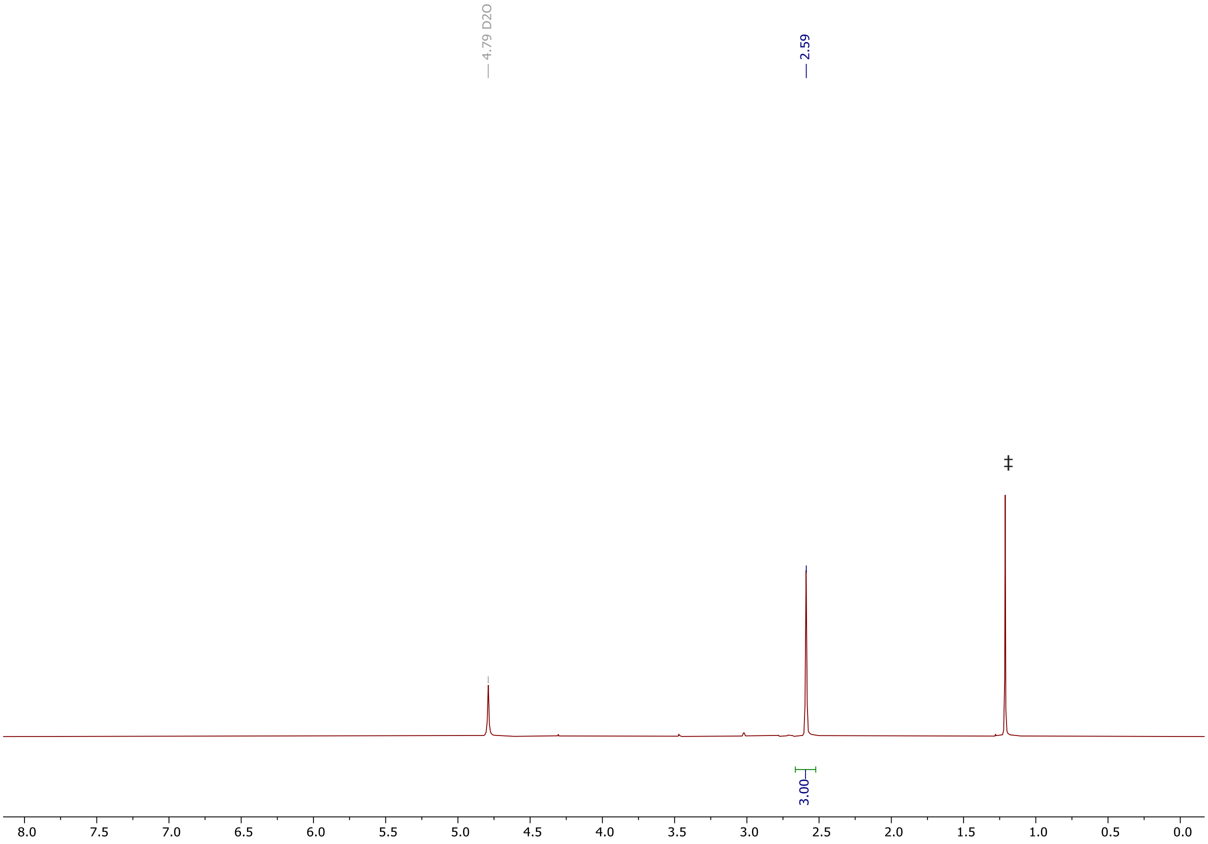

Figure S51: ^{1}H NMR Spectrum of 3l in D_2O after salt formation.

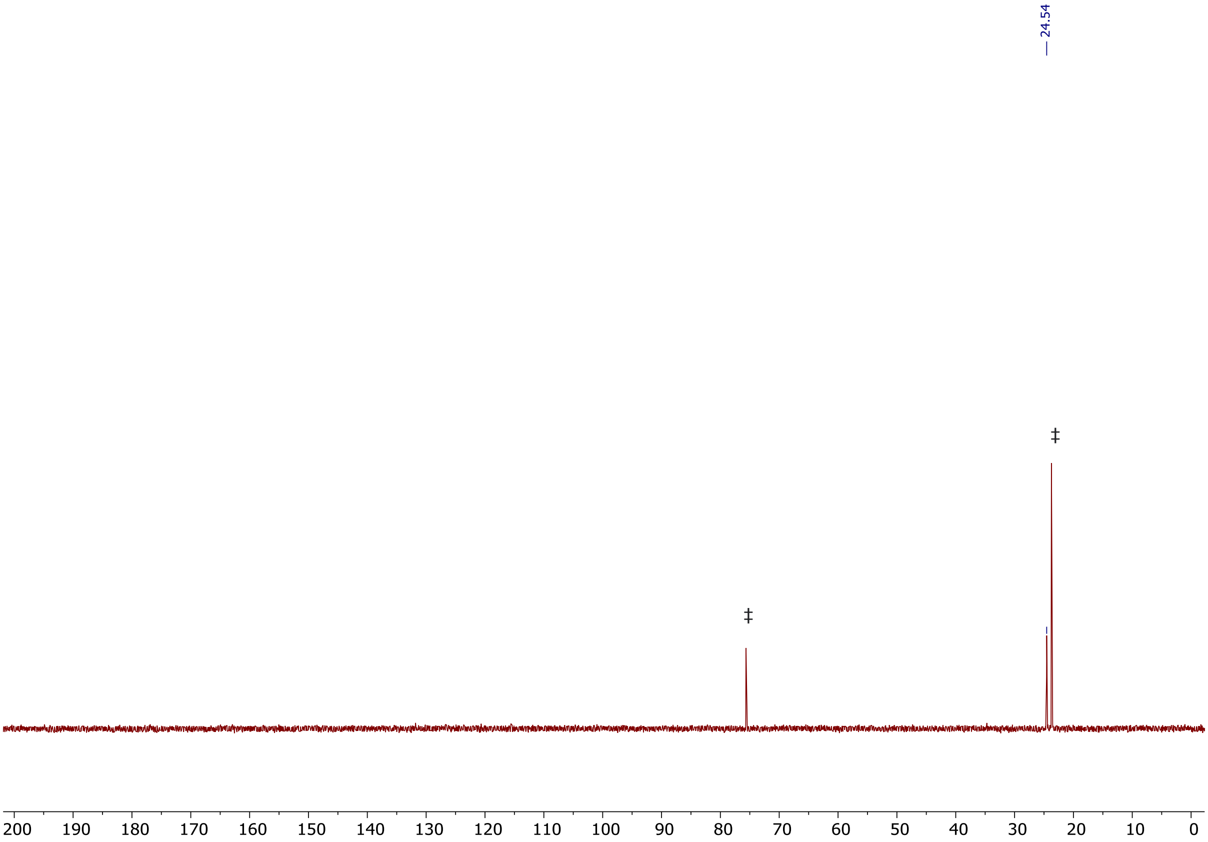

Figure S52: ^{13}C NMR Spectrum of 3l in D_2O after salt formation.

5.2.2. isopropylamine hydrochloride **3n**[5]

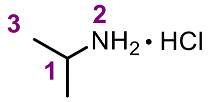

white solid (17 mg, 60%)

Analytic data is in accordance with those reported in literature.

^{1}H NMR (D_2O, 400 MHz) δ 3.49 (sept, J = 6.7 Hz, 1H, C^1-H), 3.02, (*br* s, 2H, N^2-H), 1.29 (d, J = 6.6 Hz, 6H, C^3-H).

$^{13}C\{^1H\}$ NMR (D_2O, 101 MHz) δ 44.0, 19.8.

HRMS (ESI+): calcd for [M, $C_3H_{10}N]^+$ 60.0808, found 60.0810.

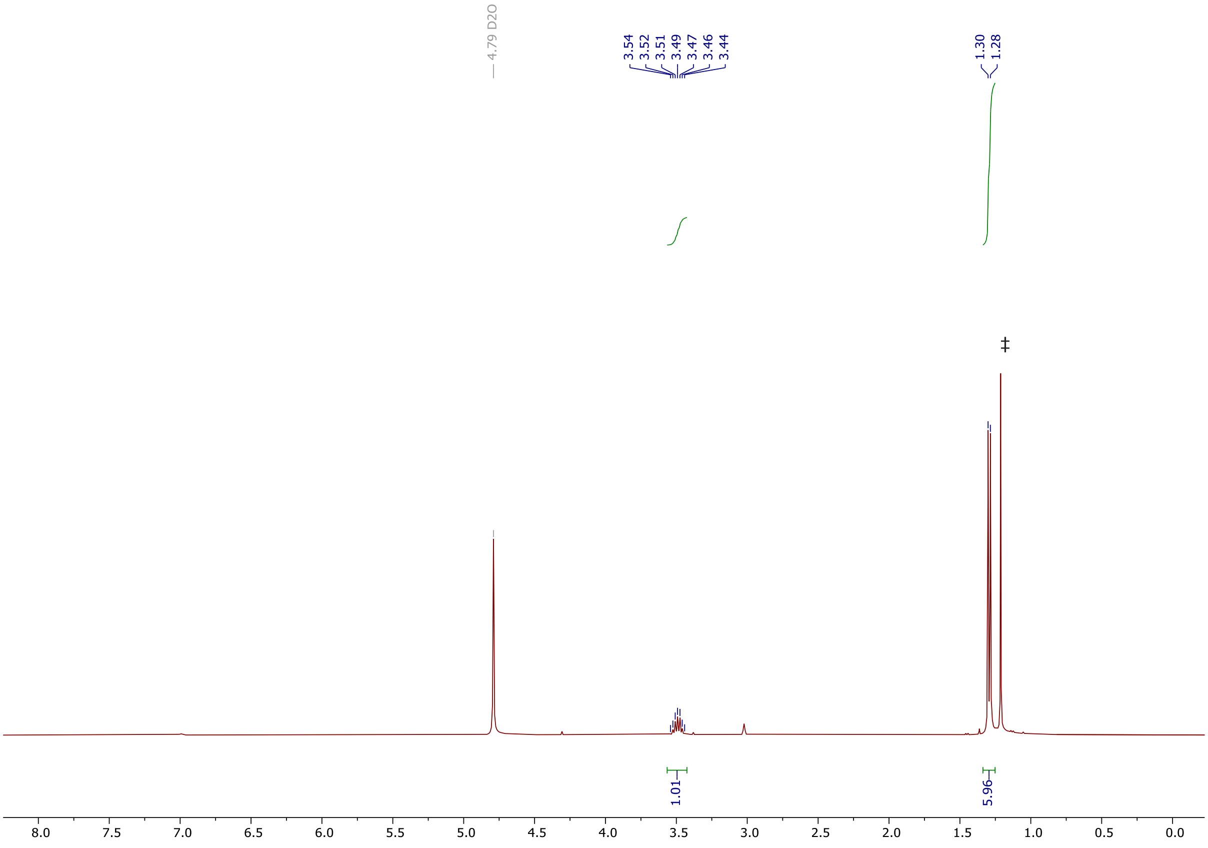

Figure S53: ^{1}H NMR Spectrum of 3n in D_2O after salt formation.

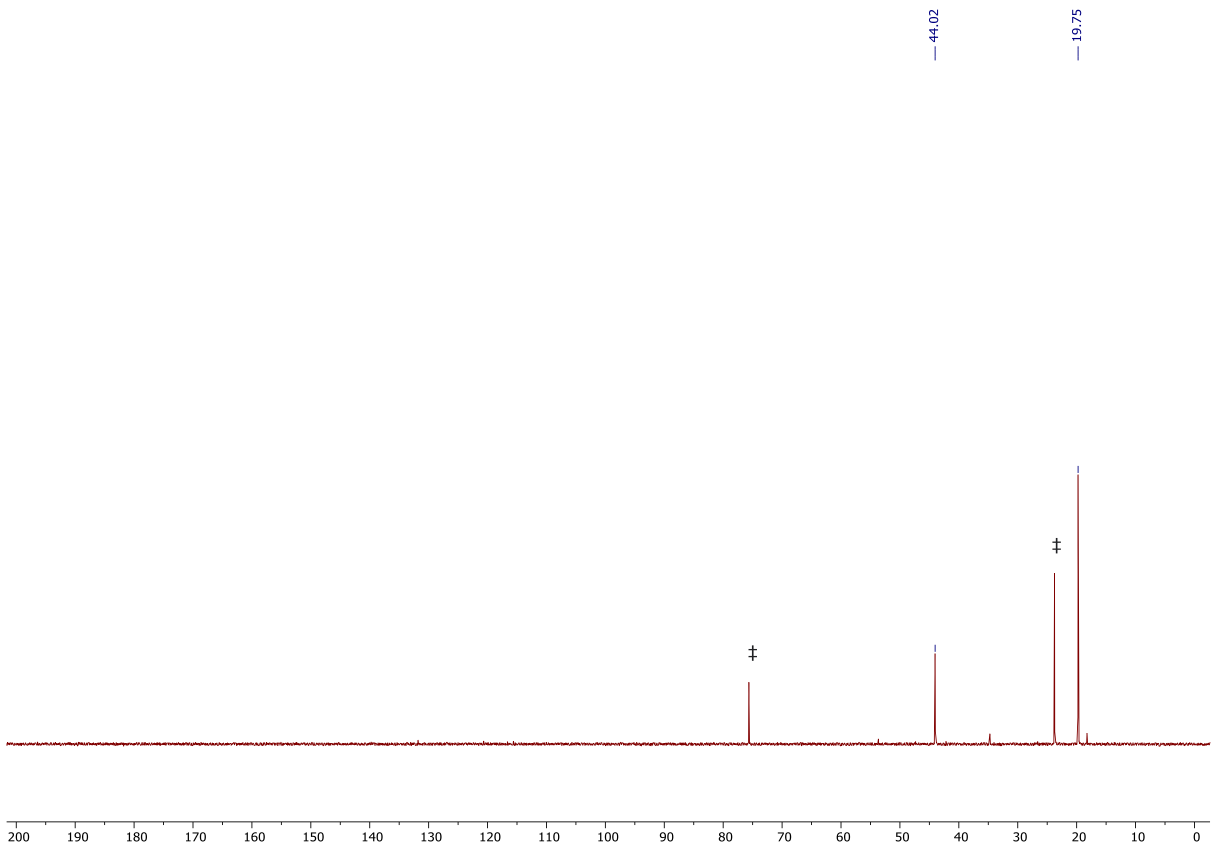

Figure S54: ^{13}C NMR Spectrum of 3n in D_2O after salt formation.

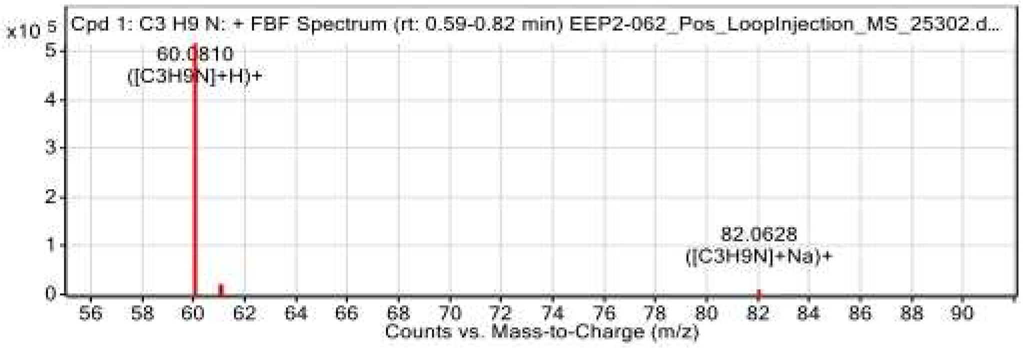

Figure S55: HRMS spectra for compound 3n.

5.2.3. *tert*-butylamine hydrochloride **3p**[5]

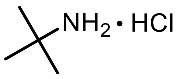

white solid (18 mg, 54%)

Analytic data is in accordance with those reported in literature.

^{1}H NMR (D_2O, 400 MHz) δ 1.37 (s, 9H, $(CH_3)_3$).

^{13}C{^{1}H} NMR (D_2O, 101 MHz) δ 51.9, 26.6.

HRMS (ESI+): calcd for [M, $C_4H_{12}N$]$^+$ 74.0964, found 74.0967.

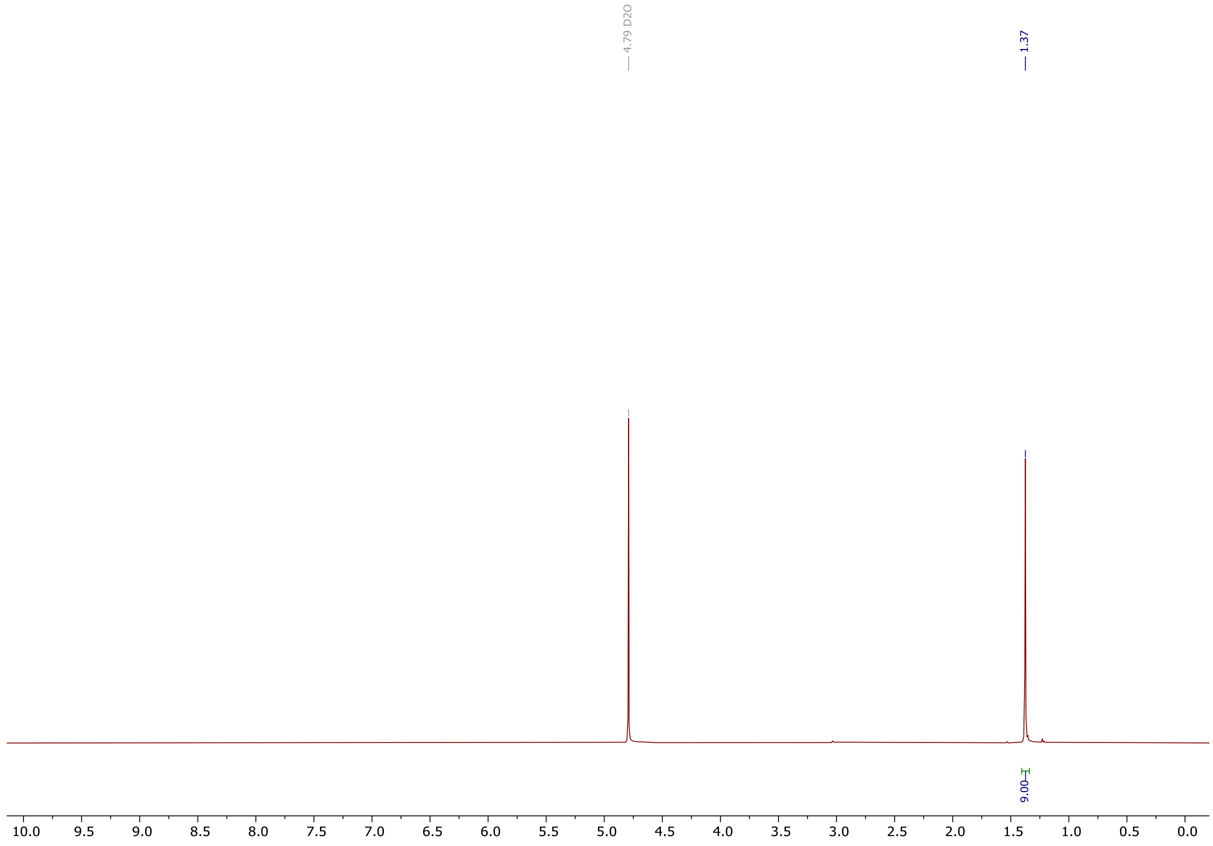

Figure S56: ^{1}H NMR Spectrum of 3p in D_2O after salt formation.

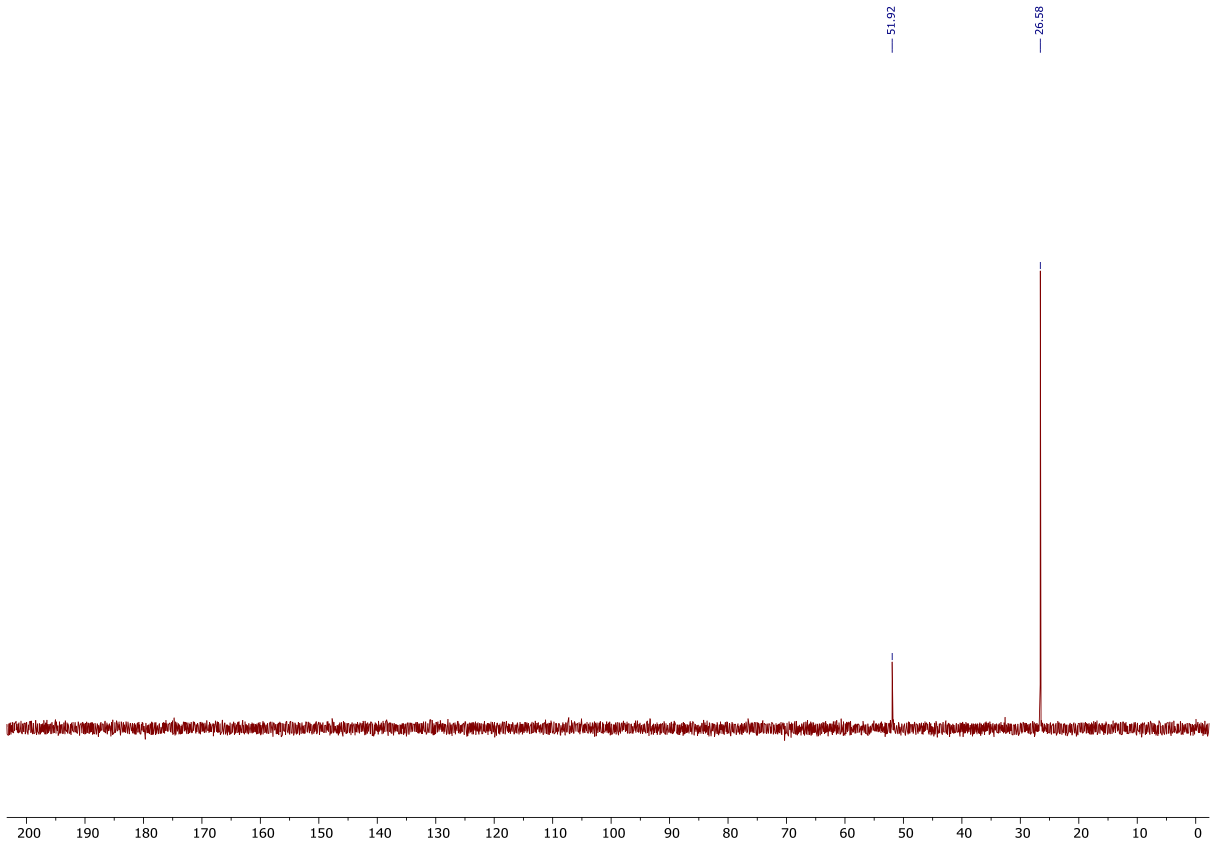

Figure S57: ^{13}C NMR Spectrum of 3p in D_2O after salt formation.

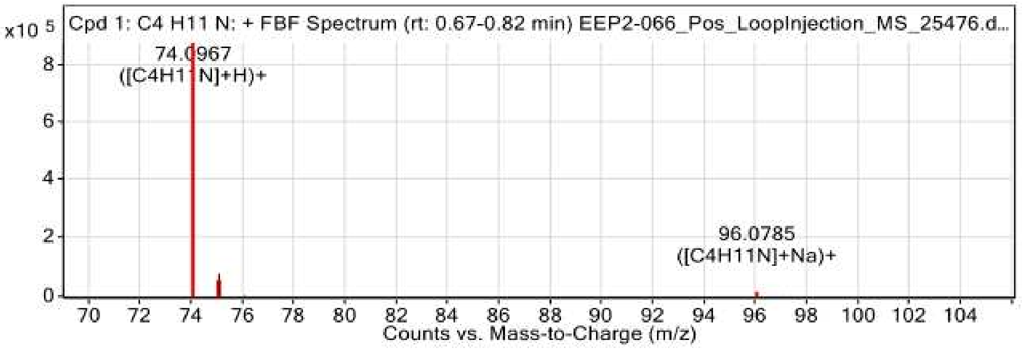

Figure S58: HRMS spectra for compound 3p.

5.2.4. *n*-pentylamine hydrochloride **3q**[5]

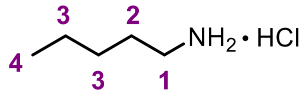

white solid (21 mg, 56%)

Analytic data is in accordance with those reported in literature.

^{1}H NMR (D_2O, 400 MHz) δ 3.00 (t, J = 7.6 Hz, 2H, C^1-H), 1.66 (app. q, J = 7.4 Hz, 2H, C^2-H), 1.39 – 1.32 (m, 4H, C^3-H), 0.90 (t, J = 6.9 Hz, 3H, C^4-H).

^{13}C{^{1}H} NMR (D_2O, 101 MHz) δ 39.5, 27.7, 26.3, 21.4, 13.0.

HRMS (ESI+): calcd for [M, $C_5H_{14}N$]$^+$ 88.1121, found 88.1123.

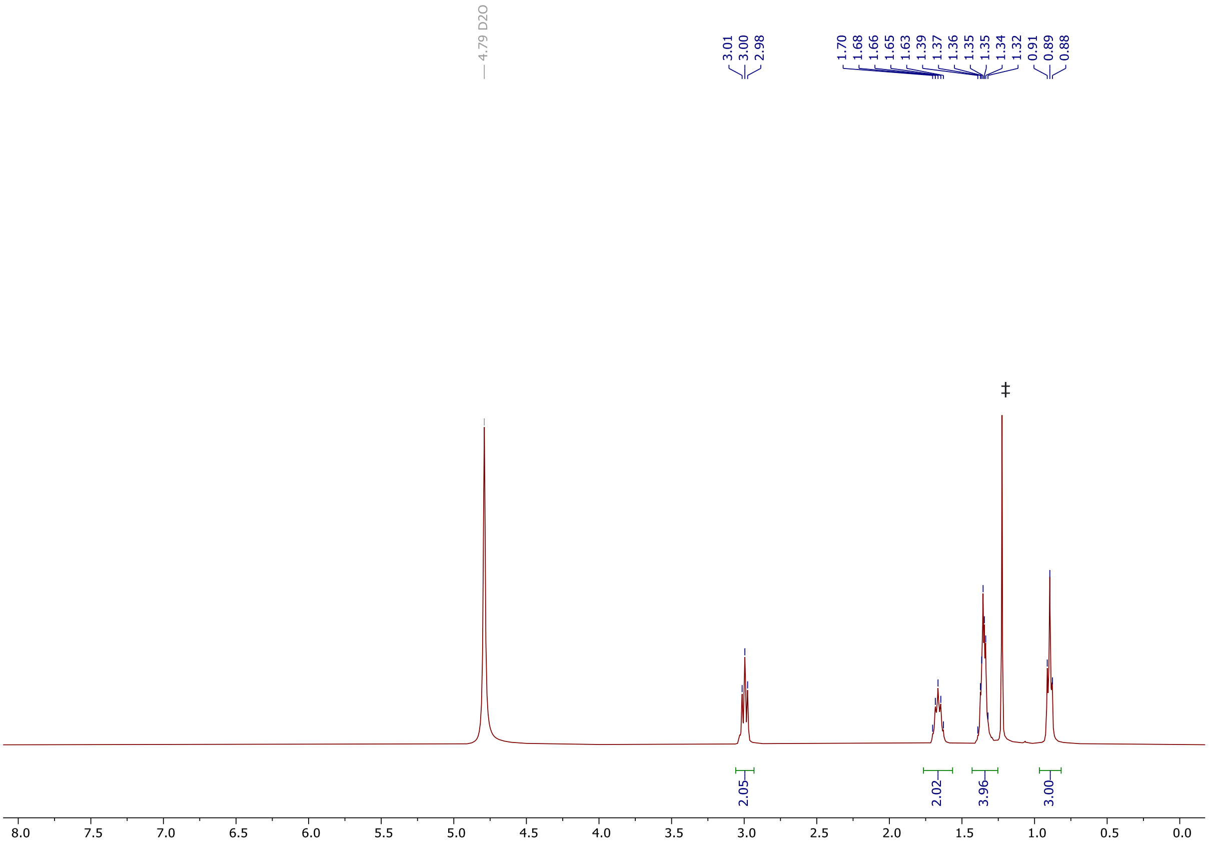

Figure S59: ^{1}H NMR Spectrum of 3q in D_2O after salt formation.

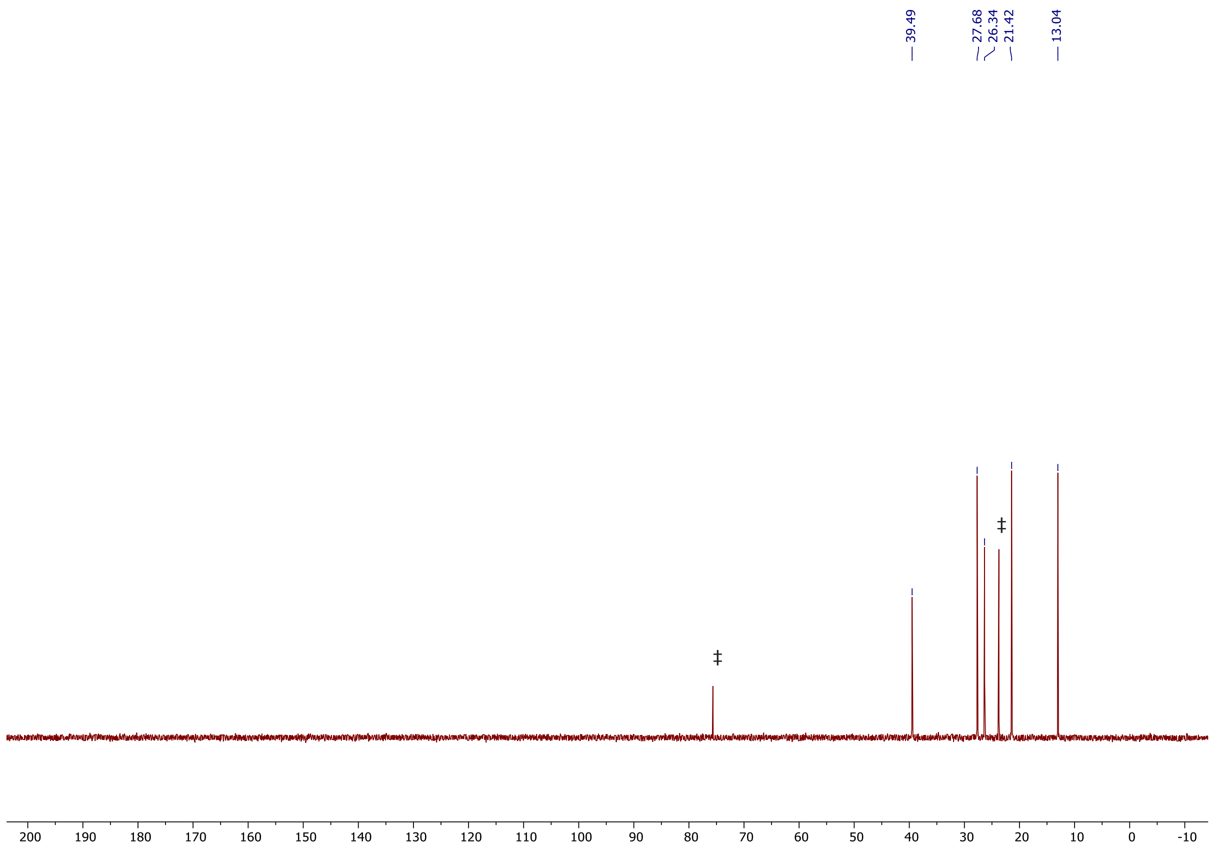

Figure S60: ^{13}C NMR Spectrum of 3q in D_2O after salt formation.

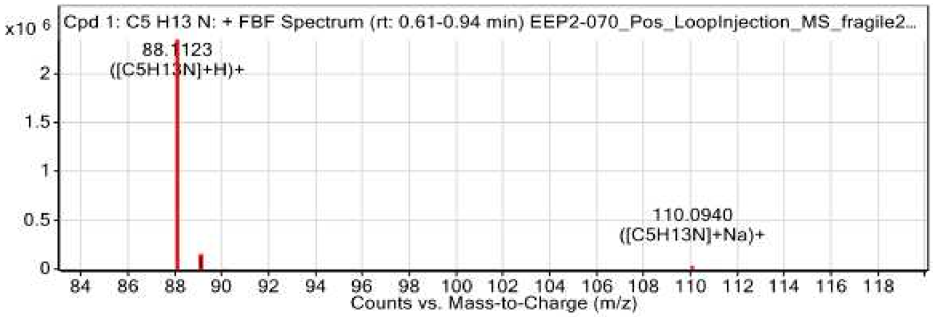

Figure S61: HRMS spectra for compound 3q.

6. Control Reactions

Phenyl hydroxylamine (0.3 mmol) was treated with 1a (1mol%) and pinacolborane 217.6 uL, 1.5 mmol) in MeCN-d3 (0.5 mL) at rt. The reaction was found to proceed via azoxybenzene (6a)[6] and azobenzene (6a’) intermediates which over time gets converted to the desired product (2a).

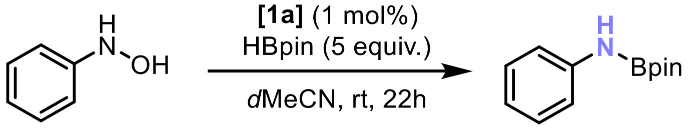

Figure S62: Reaction of hydroxyl amine under the optimised reduction conditions.

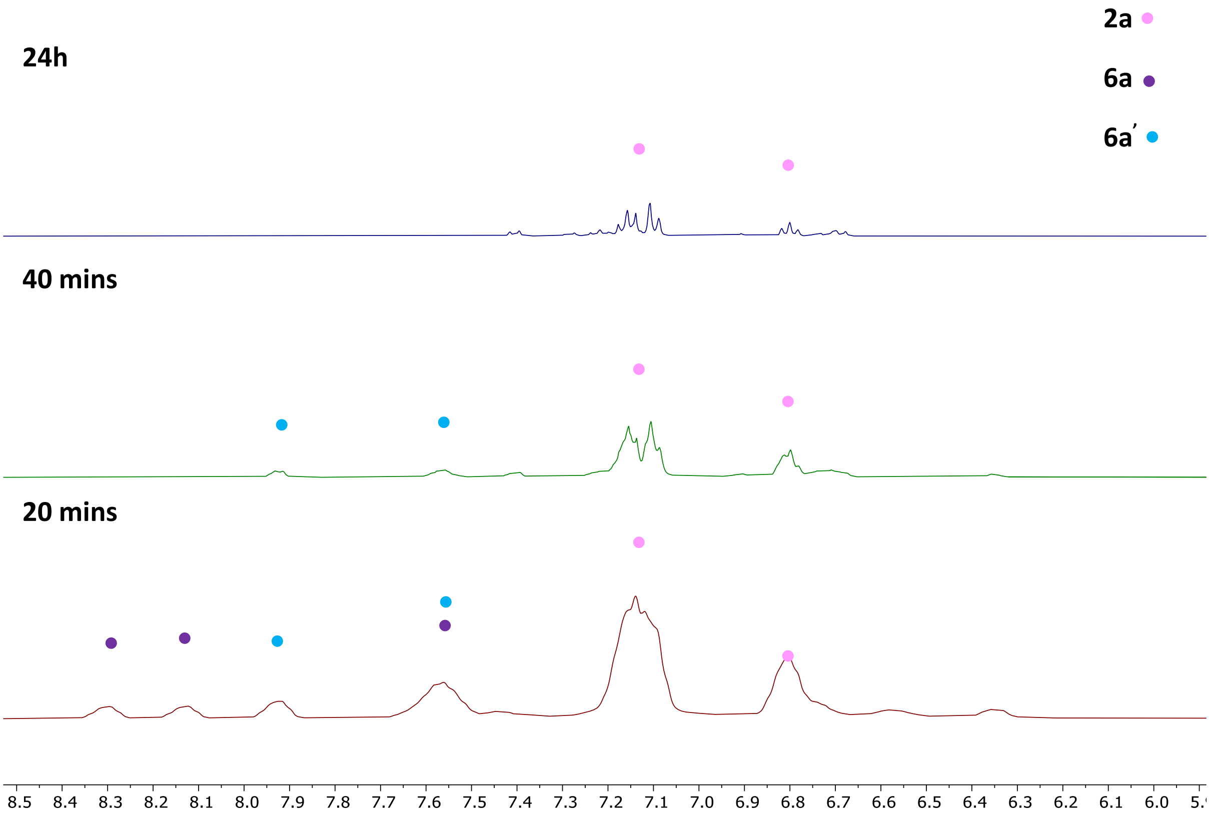

Figure S63: ^{1}H NMR spectrum in CD_3CN of hydroxylamine subjected to our standard reduction conditions at varying time points.

The reaction mixture NMR spectrum appeared to suffer from poor resolution, in light of this phenyl hydroxylamine (0.15 mmol) was treated with 1a (1 mol%) and pinacolborane triethoxysilane 83.1uL, 0.45 mmol) in MeCN-d3 (0.5 mL) at rt for 20 mins. A similar product distribution was observed when using this alternative reductant with the reaction initially forming a large amount of azoxybenzene[6], aniline and some azobenzene which overtime was converted to the desired aniline product.

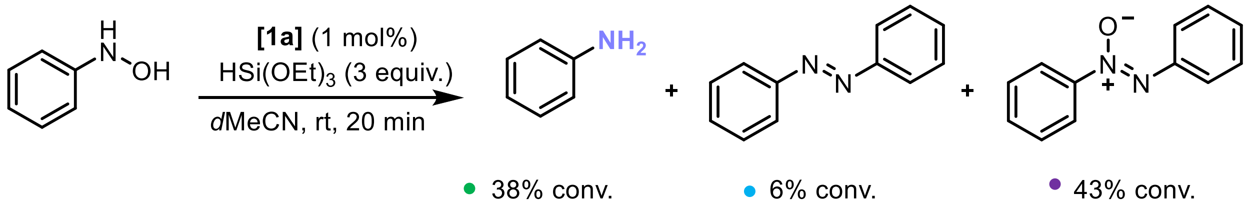

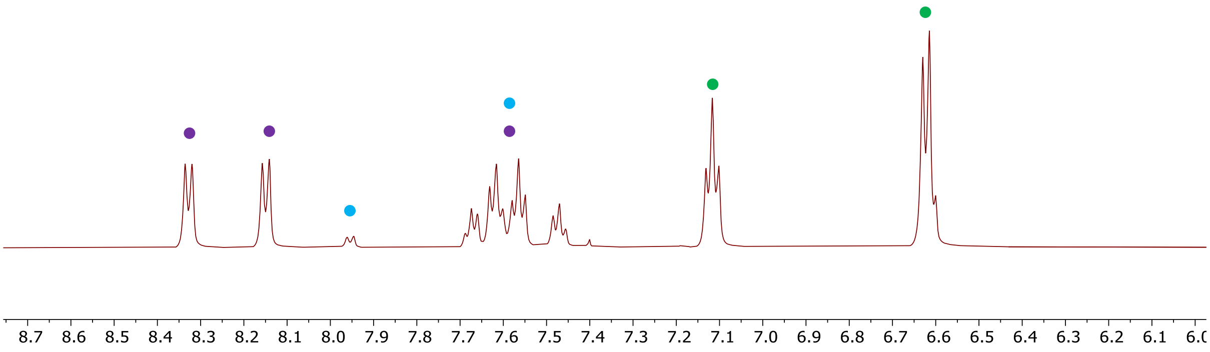

Figure S64: ^{1}H NMR spectrum in CD_3CN of hydroxylamine (1 equiv.), 1a (1mol%) and $HSi(OEt)_3$ (3 equiv.) after 20 mins at rt.

nitrosobenzene (0.15 mmol) was treated with 1a (1mol%) and pinacolborane (0.75 mmol) in MeCN-d3 (0.5 mL) at rt. The reaction instantly formed 87% of the corresponding aniline showing it is highly likely our mechanism proceeds via a nitroso intermediate.

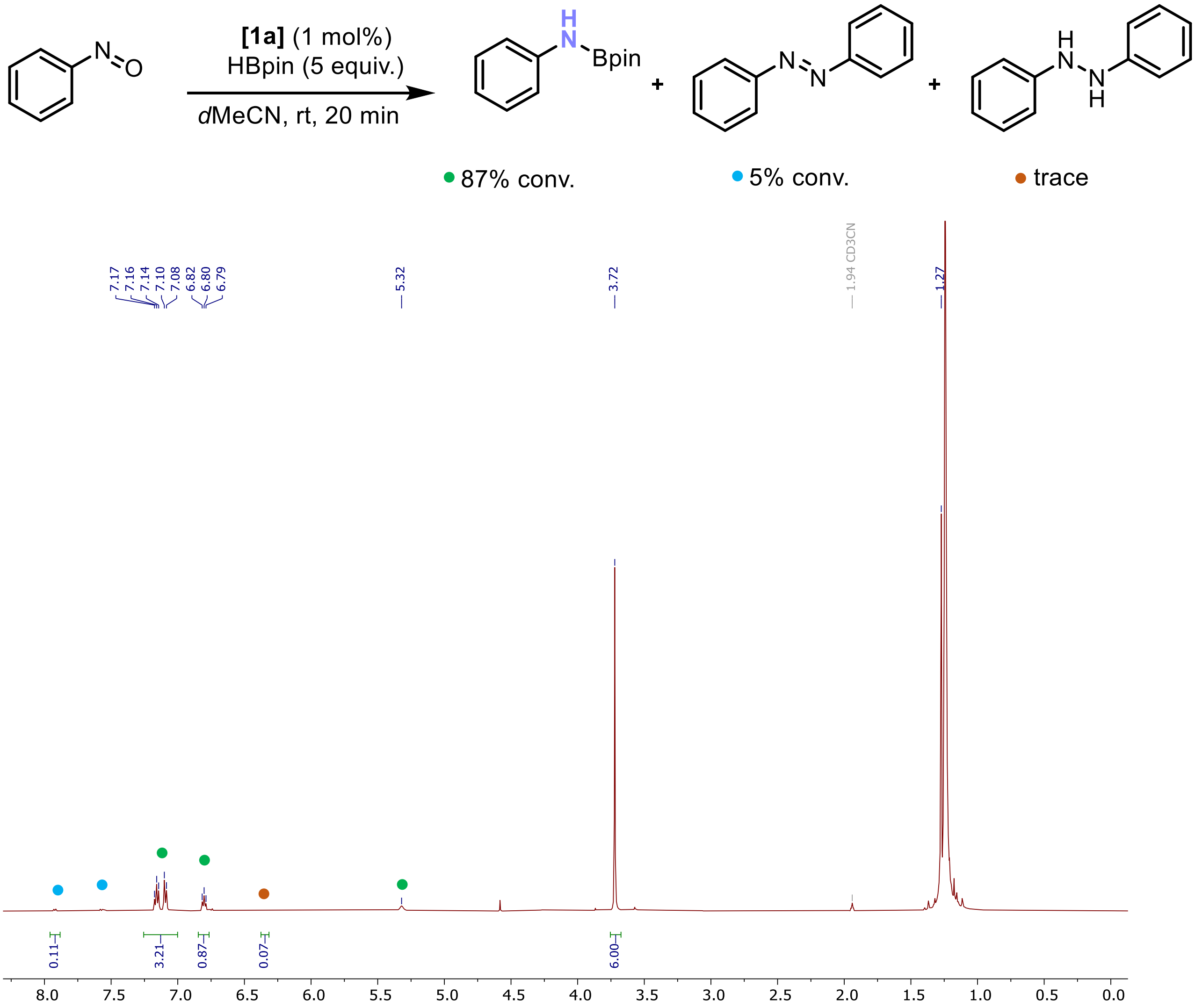

Figure S65: ^{1}H NMR spectrum in CD_3CN of nitrosobenzene subjected to our standard reduction conditions after 20 mins at rt.

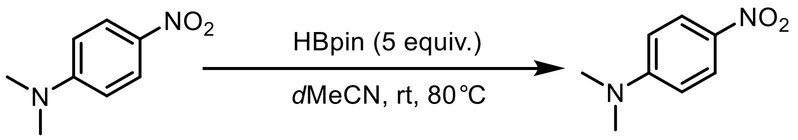

Figure S66: Reaction of N,N-dimethyl-4-nitrobenzene with pinacolborane at 80 ℃ overnight.

N,*N*-dimethyl-4-nitrobenzene (3.3 mg, 10 umol) was treated with pinacolborane (8.7 uL, 30 umol) in MeCN-d_3 (0.5 mL). No reduction was observed at 80 °C overnight.

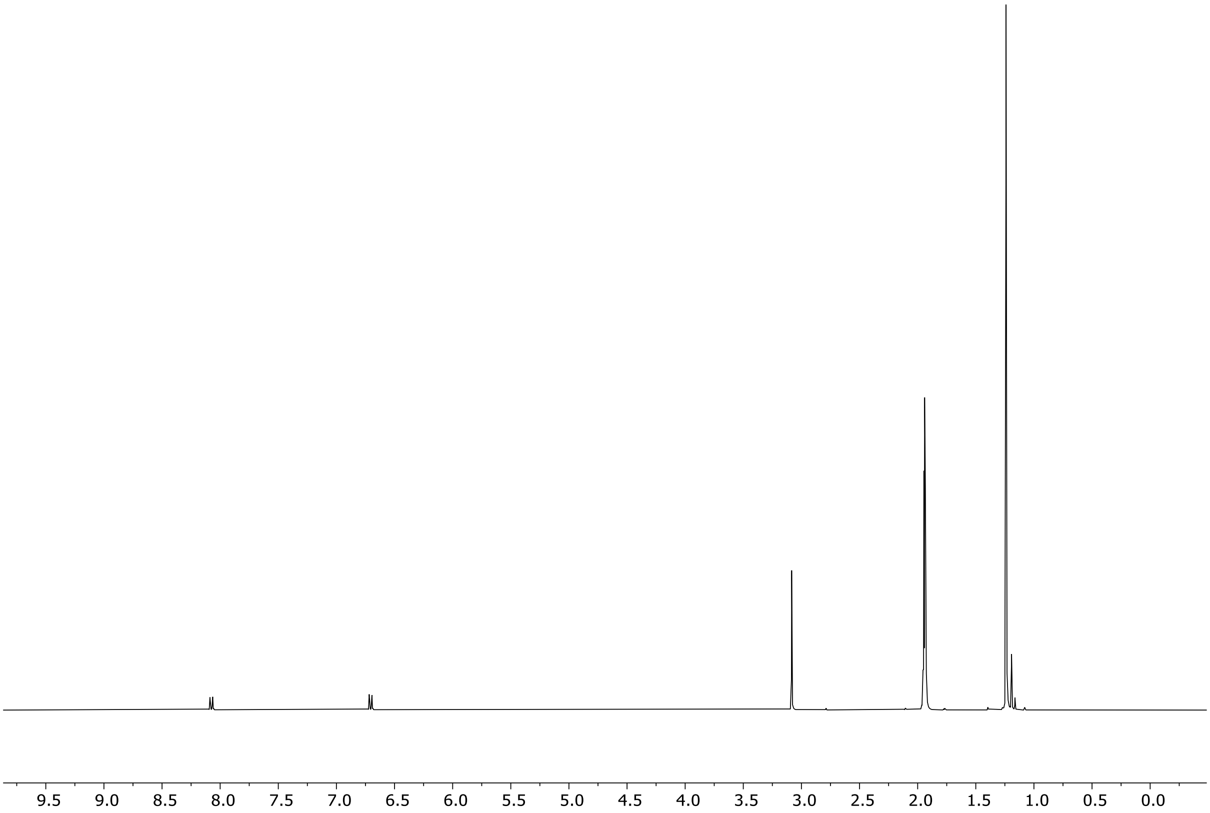

Figure S67: ^{1}H NMR spectrum in CD_3CN of N,N-dimethyl-4-nitrobenzene and pinacolborane after 16 hours at 80 °C.

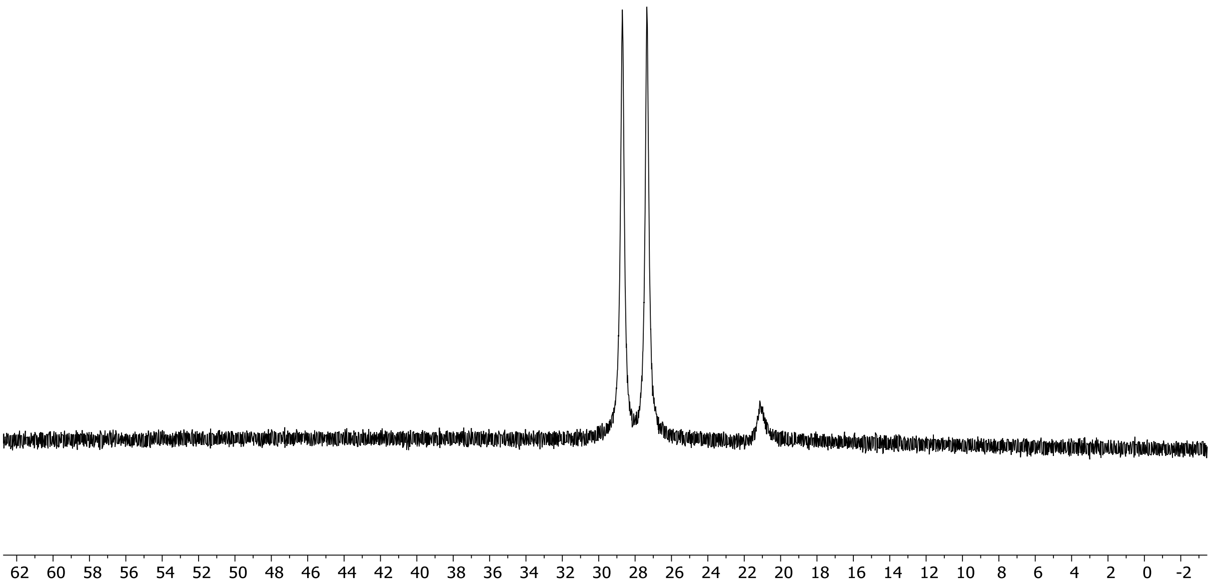

Figure S68: $^{11}B\{^{1}H\}$ NMR spectrum of N,N-dimethyl-4-nitrobenzene and pinacolborane after 16 hours at 80 °C.

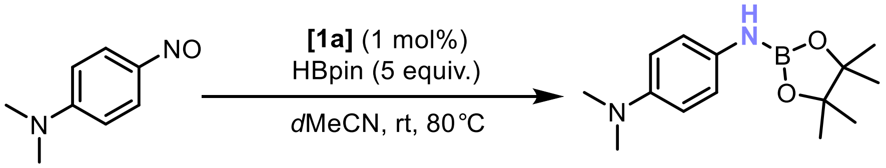

Figure S69: Reaction of N,N-dimethyl-4-nitrosobenzene with pinacolborane at 80 ℃ overnight.

N,*N*-dimethyl-4-nitrosobenzene (3.0 mg, 10 umol) was treated with pinacolborane (8.7 uL, 30 umol) in MeCN-d_3 (0.5 mL). Complete reduction was observed after 80 °C overnight. The nitroso can be reduced to aniline using HBpin in the absence of 1a, but requires 18 h at 80 °C to do so, therefore reduction of the nitroso intermediate must also be iron catalysed.

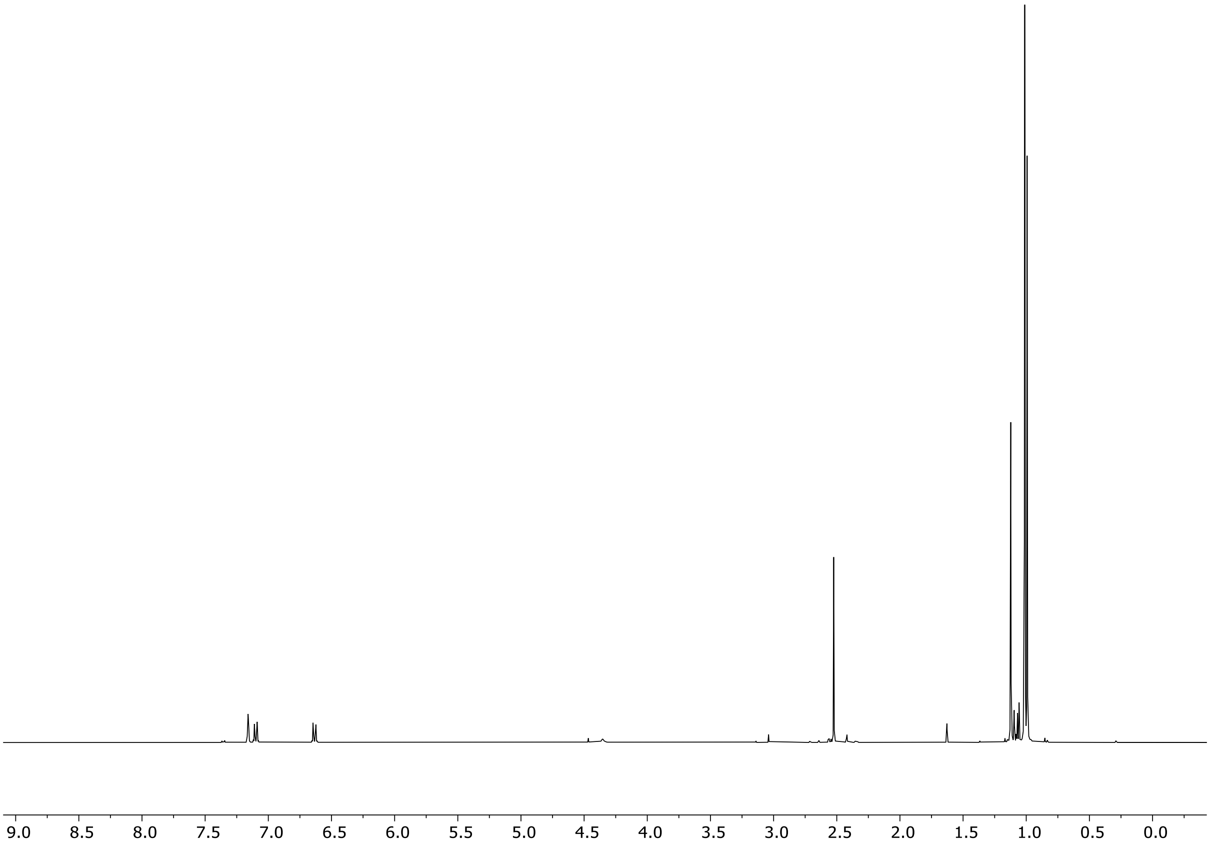

Figure S70: ¹H NMR spectrum of N,N-dimethyl-4-nitrosobenzene and pinacolborane after 16 hours at 80 °C.

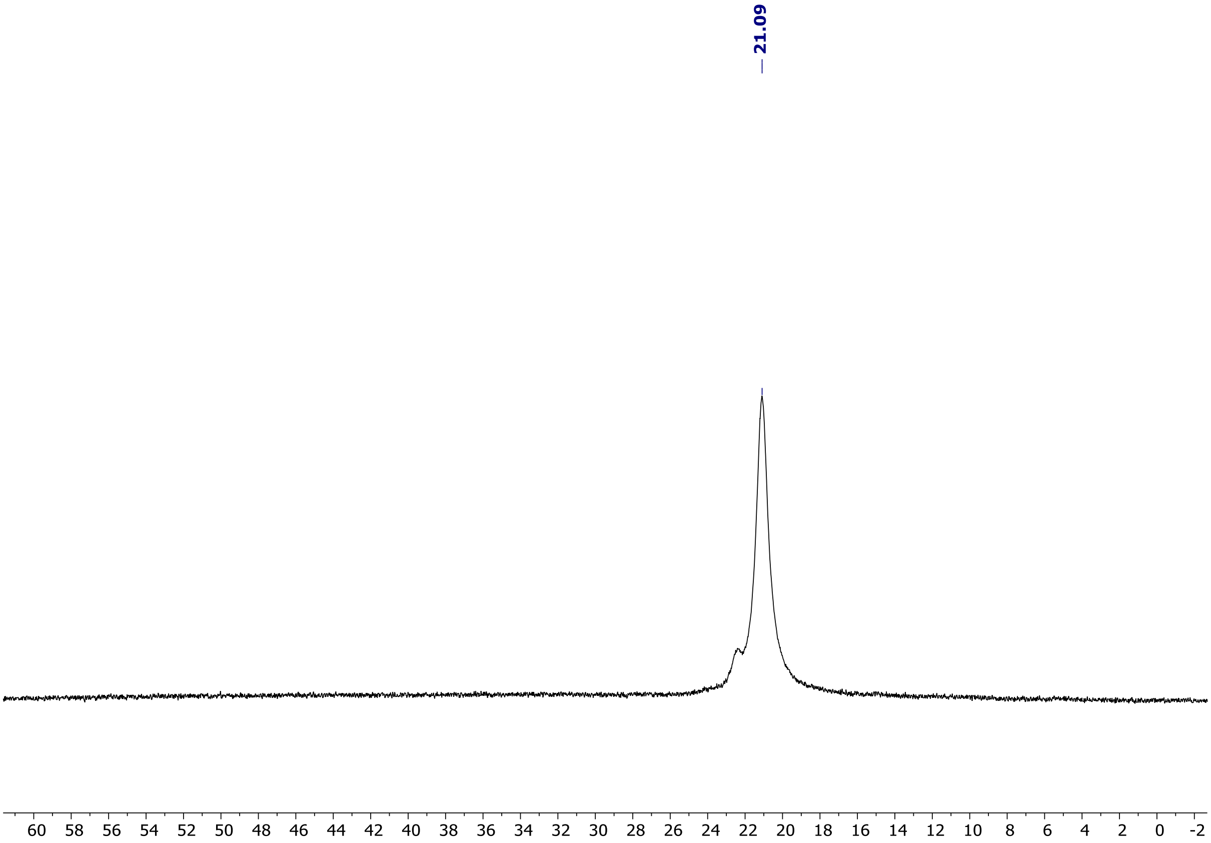

Figure S71. $^{11}B\{^1H\}$ NMR spectrum of N,N-dimethyl-4-nitrosobenzene and pinacolborane after 16 hours at 80 °C.

7. Radical Clock Experiments

7.1. General Procedure for Radical Clock / Trap Reactions

To a J-young NMR tube under argon atmosphere was added a mixture of **1a** (0.0015 mmol, 1 mg) and nitro-compound (0.15 mmol, 1 equiv.) in CD_3CN (0.5 mL). To the solution was added the radical clock / radical trap (1 equiv. – 5 equiv.) Finally, to the solution was added pinacolborane (1.5 mmol, 0.22 mL, 5 equiv.) and the reaction mixture was agitated for 16h at room temperature, NMR spectra were recorded. Spectroscopic yields were calculated by comparison to the internal standard dimethyl carbonate (0.15 mmol, 1 equiv.).

Table S2: Data collected from test reactions using a variety of radical traps / clocks added in 1 – 5 equiv.

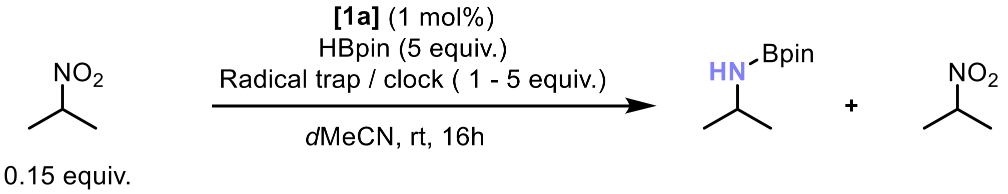

Radical Trap / Clock	Amount of Radical Trap / Clock	Yield of Product after 16h at rt / %	Amount of Unreacted Nitro Left over / %
**None**	-	82	0
**TEMPO**	1 equiv.	60	19
**TEMPO**	5 equiv.	0	100
**(chloromethyl)cyclopropane**	1 equiv.	76	5
**PBN**	1 equiv.	59	25
**PBN**	5 equiv.	9	84

7.2. Reaction of N,N-dimethyl-4-nitrobenzene with 3 equiv. TEMPO

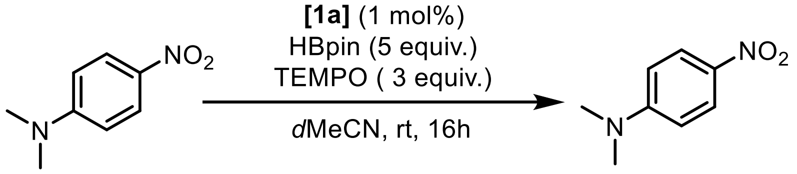

To a J-young NMR tube under argon atmosphere was added a mixture of **1a** (0.0015 mmol, 1 mg) and N,N-dimethyl-4-nitrobenzene (0.15 mmol, 1 equiv.) in CD_3CN (0.5 mL). To the solution was added the TEMPO (3 equiv.) Finally, to the solution was added pinacolborane (1.5 mmol, 0.22 mL, 5 equiv.) and the reaction mixture was agitated for 16h at room temperature, NMR spectra were recorded. Using the general procedure in the presence of 3 equivalents of TEMPO results in no conversion to the corresponding amine product. Under these conditions complete consumption of HBpin was observed with TEMPO-Bpin observed as the only ^{11}B-containing product.

nb. TEMPO-Bpin is not formed by the reaction of TEMPO and HBpin in the absence of **2a**. However, TEMPO-Bpin can be formed by the via a dehydrocoupling reaction between TEMPOL and HBpin.

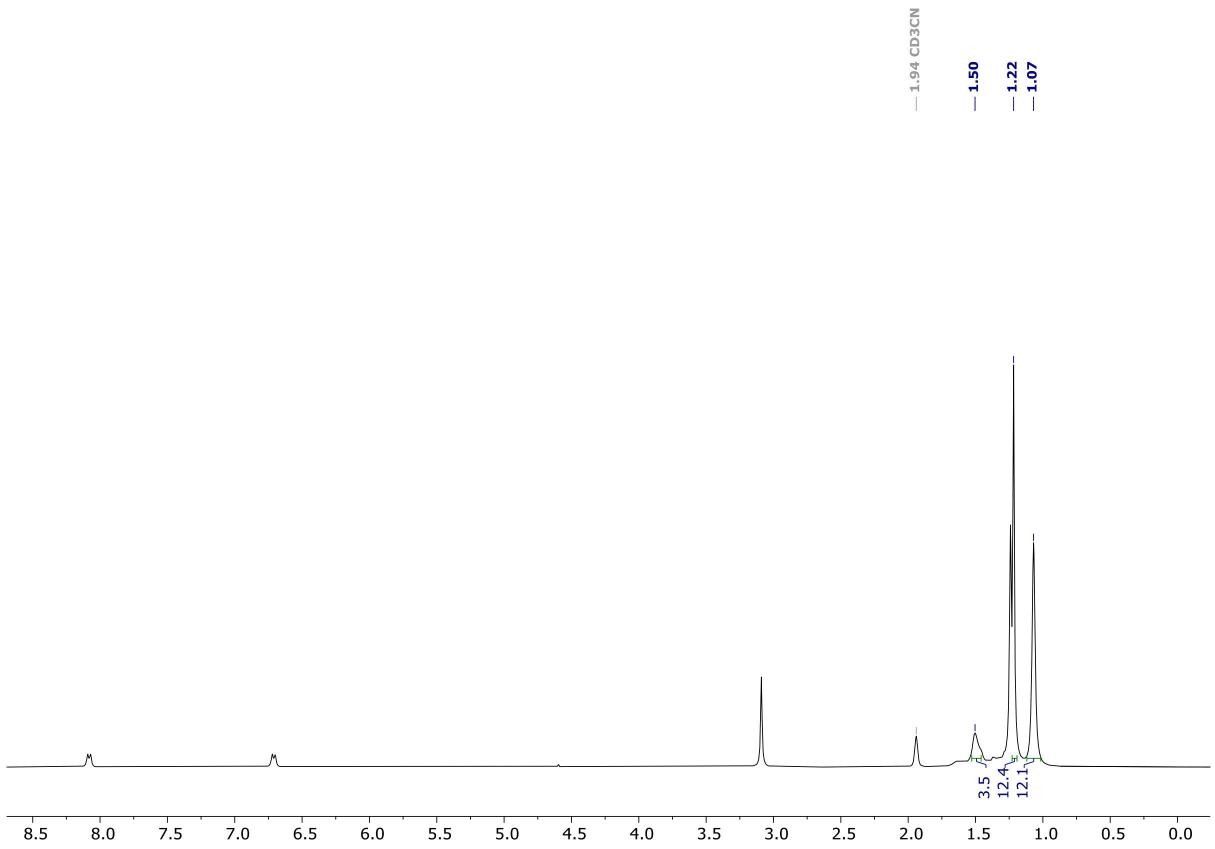

Figure S72: ^{1}H NMR spectrum in CD_3CN of the attempted reduction of N,N-dimethyl-4-nitrobenzene in the presence of 3 equivalents of TEMPO .

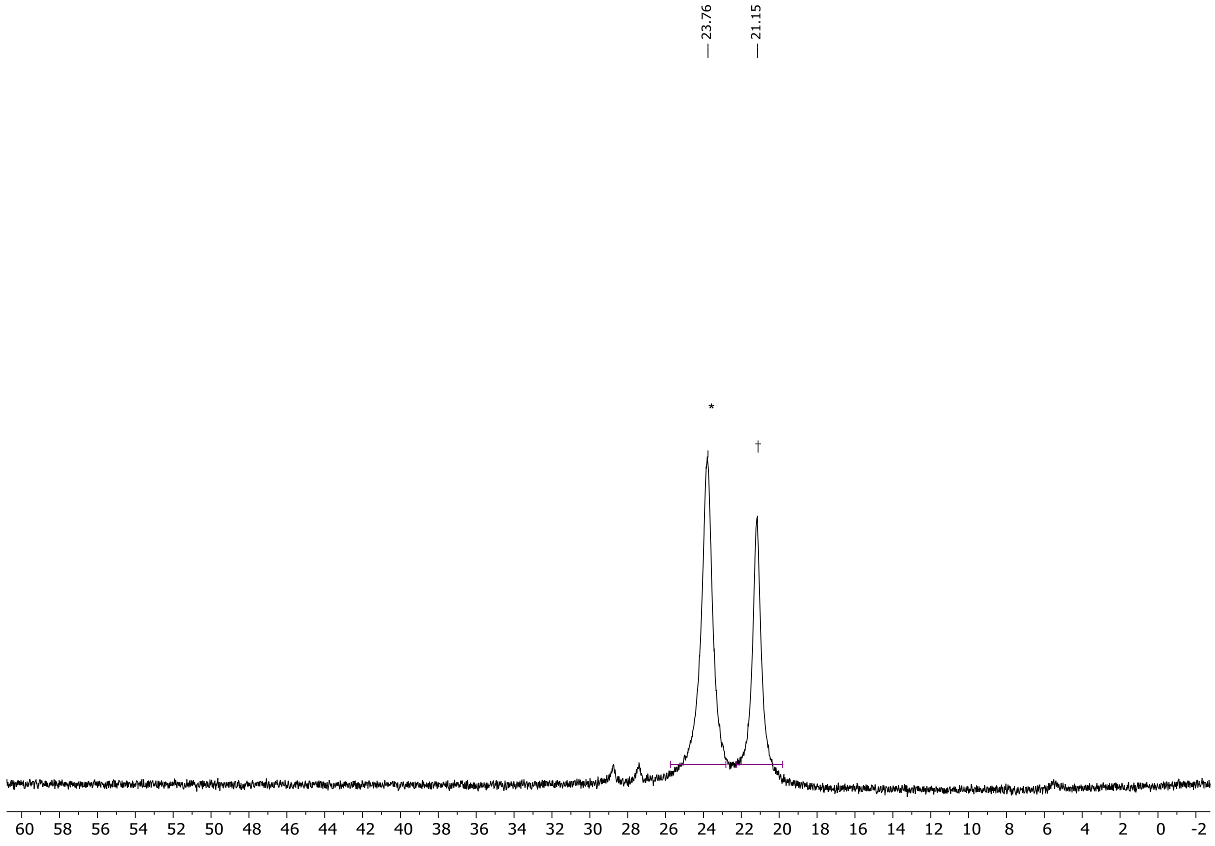

*Figure S73: $^{11}B\{^{1}H\}$ NMR spectrum in CD_3CN of the attempted reduction of N,N-dimethyl-4-nitrobenzene in the presence of 3 equivalents of TEMPO. *TEMPO-Bpin and †O(Bpin)$_2$.*

8. Kinetic Experiments

8.1. General Notes for Kinetic Analysis

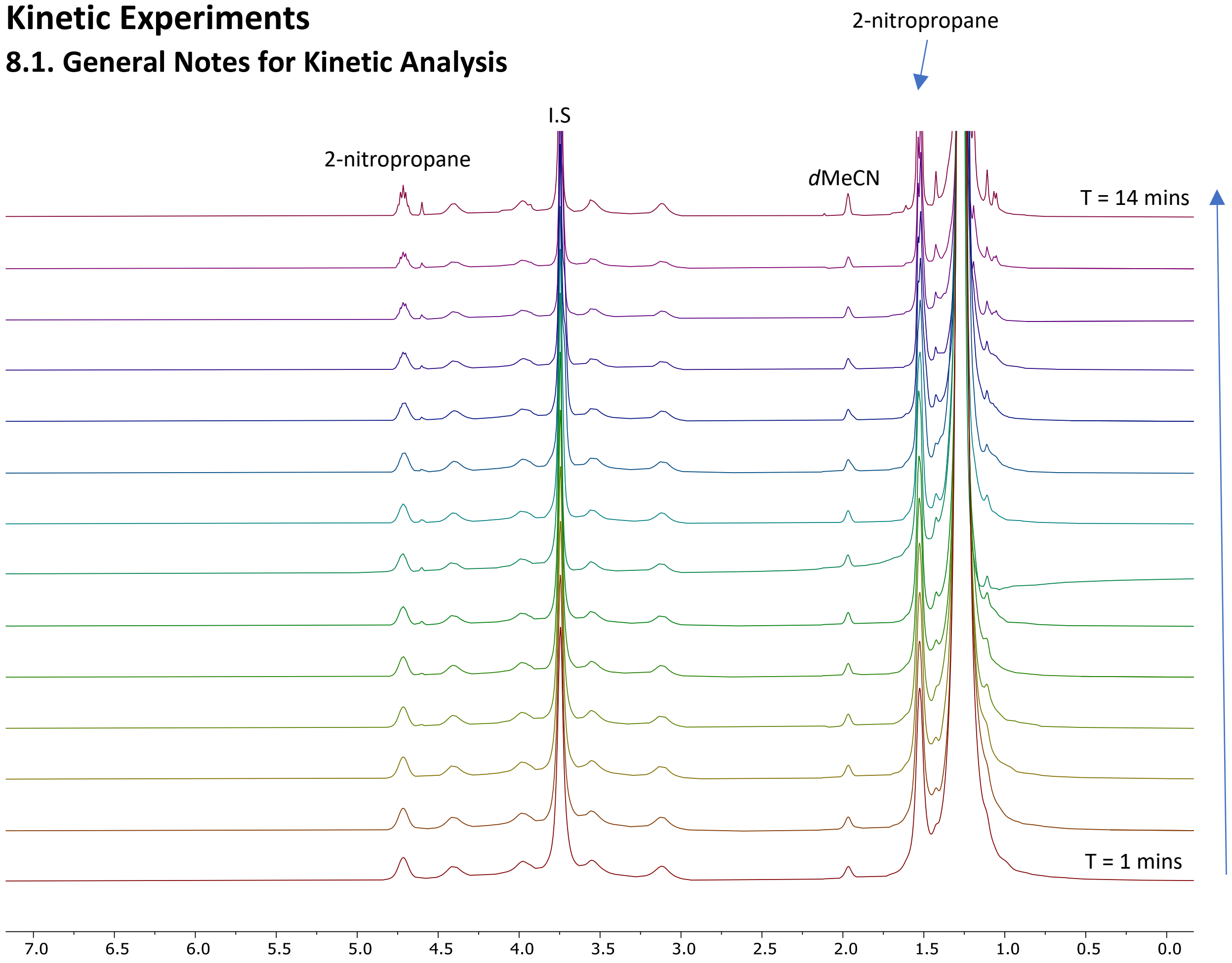

Figure S74: Reaction profile for kinetic run using 2-nitropropane and HBpin highlighting the issues with Para-magnetism observed in the first 10-15 minutes of the kinetic run.

Due to the paramagnetic nature of **1a** and the fact it isn't fully soluble at the start of the reaction the reaction suffers from Para-magnetism as observed in figure S74. Due to this where possible data was taken from around 10-15 minutes after the shimming / line broadening had significantly improved.

8.1.1. VTNA plots Information

For VTNA, calculations for order in substrate/catalyst were performed as follows: X-axis data point = y + ([*substrate*]$_x$ + [*substrate*]$_y$)/2 ^Z * (time$_y$ – time$_x$) where a given cell is X or Y and Z is the computed 'order-in'. For example, to generate an X-axis entry in cell C2 = C1 + (B1 + B2)/2 ^1 * (A2 – A1), where B is the concentration and A is the unit of time and the order is 1st order. For order in iron, it was assumed that the concentration of iron did not change as a function of time and thus [Fe]$_x$ = [Fe]$_y$.

8.2. Order with respect to [Fe]

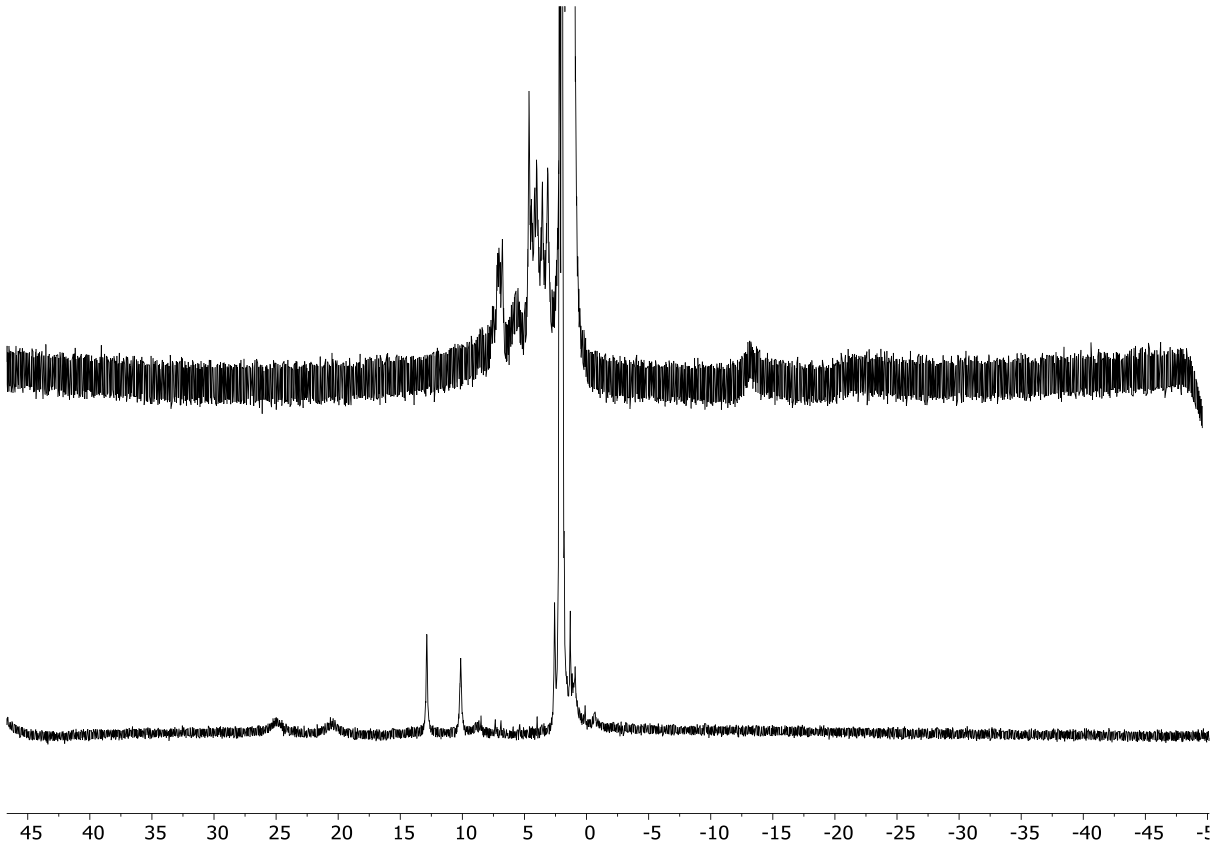

*Figure S75: ^{1}H NMR spectrum in CD_3CN of **1a** (bottom) and ^{1}H NMR spectrum in CD_3CN of the reaction of **1a** with HBpin after 5 minutes at rt (top).*

Reaction of **1a** with HBpin results in complete loss of **1a** within 5 minutes at room temperature.

We have not been able to obtain order in **1a** based on initial rates data using HBpin as the reductant based on the fast activation process that brings **1a** on-cycle. However, by using $HSi(OEt)_3$ to reduce $PhNO_2$ and measuring the initial induction period, we can see an approximate half order relationship in **1a**, indicating that this splits into two equally active monomers i.e. two equivalents of **1c**.

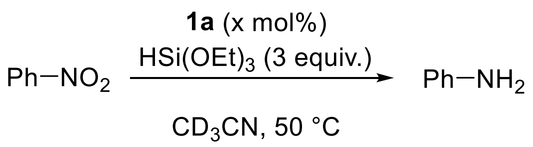

Scheme S1: Kinetics studying the order in [Fe] using a silane reductant.

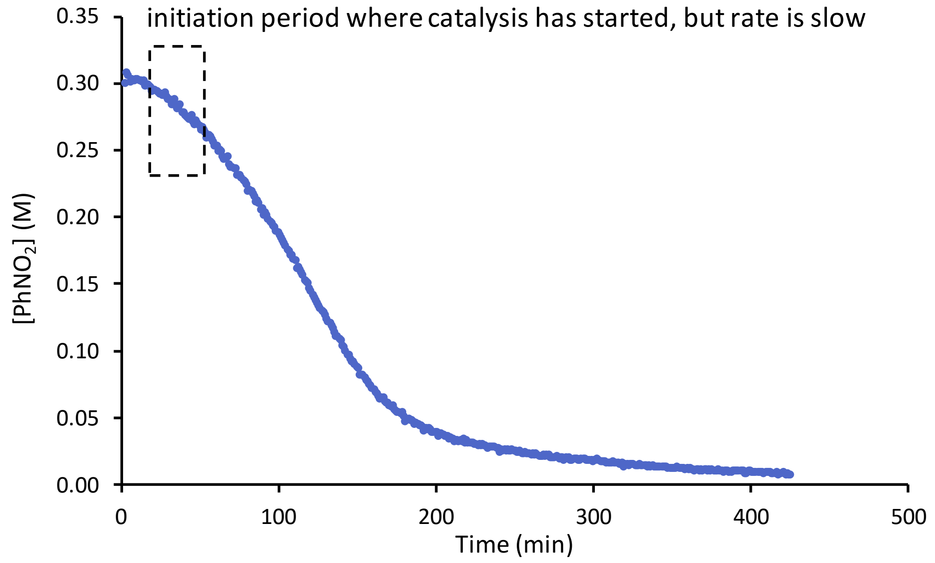

Figure S76: Uptake of $PhNO_2$ versus time using $HSi(OEt)_3$ as the reducing agent, showing initial area where initial rate data was extracted from. 0 to 5 mins shows no conversion of $PhNO_2$, so not included in LN plot. The same approach was taken for all loadings of 1a (0.5, 0.75, 1.0, 1.25 mol%).

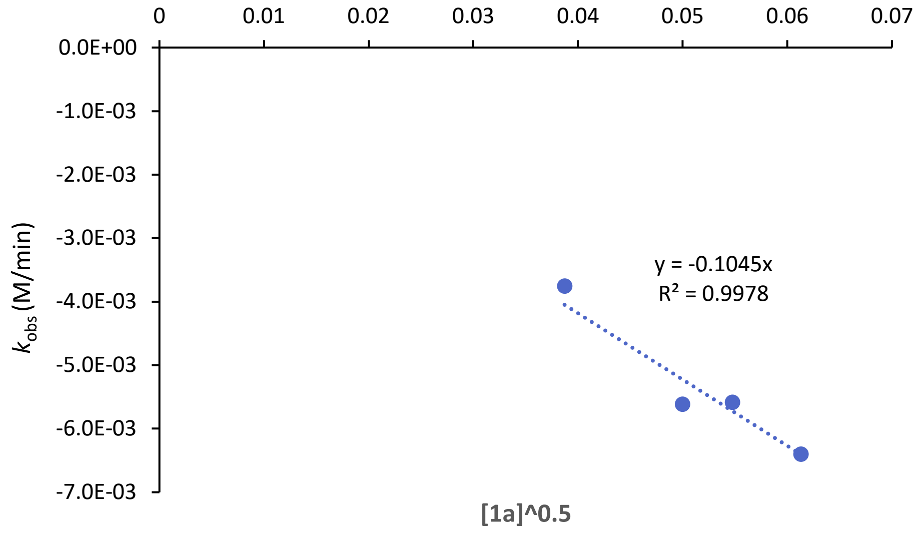

*Figure S77: Reaction rate (M/min) versus [**1a**]$^{0.5}$, using $HSi(OEt)_3$ as the reducing agent.*

Given that activation of **1a** to on-cycle species is almost instantaneous, we investigated the overall order in catalyst. Both a VTNA and LN plots using HBpin to reduce *i*$PrNO_2$ gives a 1st order relationship in iron. We link this to the fact that once on-cycle the mononuclear iron species (e.g. **1c**) behaves in a first order manner throughout and no competitive re-dimerisation takes place.

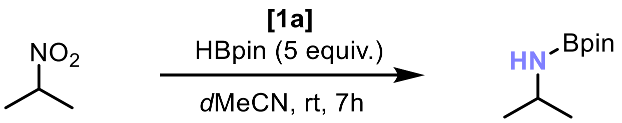

Scheme S2: Kinetics studying the order in [Fe].

Kinetic studies were undertaken using the general method for the reduction of aliphatic nitro-compounds (Section 4.3) using varied amounts of [**1a**], pinacol borane (5 equiv.) with 1 equiv. 2-nitropropane staring material in 0.5 mL CD_3CN at RT. Reactions were monitored by consumption of substrate vs. a dimethyl carbonate internal standard using in-situ NMR analysis (^{1}H NMR, CD_3CN, 400 MHz).

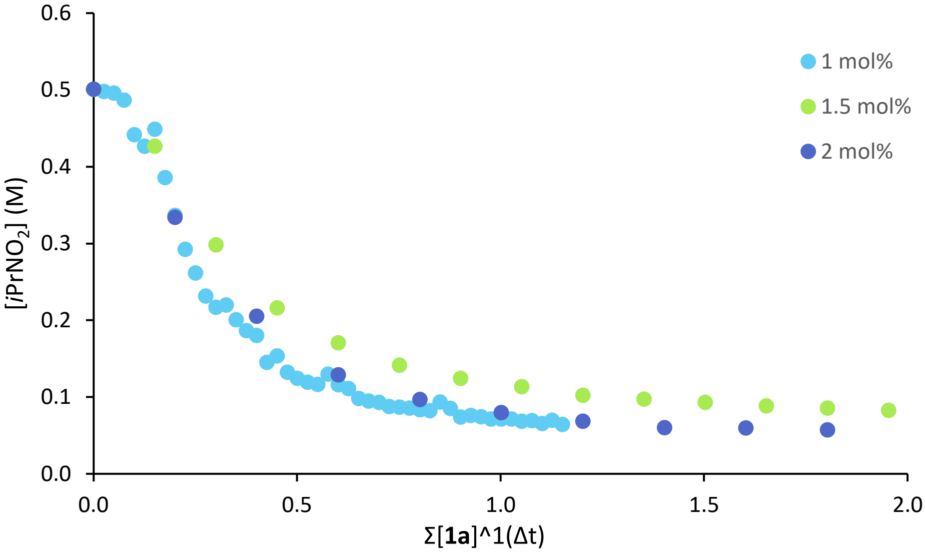

*Figure S78: 1st order VTNA plot showing the loss of iPrNO$_2$ at 1, 1.5 and 2 mol% **1a**. NB. 0.5 mol% **1a** gives a poor correlation over the main time period of catalysis (100 to 400 min).*

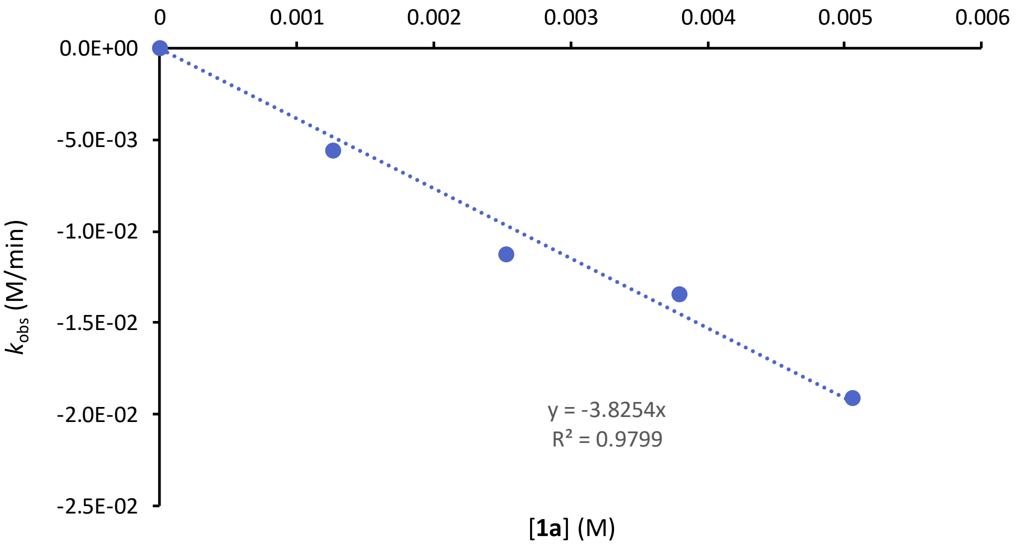

*Figure S79: Reaction rate (M/min) versus [**1a**], using HBpin as the reducing agent. Data collected using 0.5, 1, 1.5 and 2 mol% **1a**.*

8.2.1. Summary of Order in Iron Data

Our data suggests that the reaction is half order in pre-catalyst **1a**. This is based on data acquired using silane as the reducing agent. The HBpin data suggests that almost instantaneous and complete catalyst activation occurs, and when these data sets are collected we are measuring the order in on-cycle iron species. We believe our on-cycle iron is mononuclear in nature. The reason for this being the weight of combined data supported by EPR and DFT calculations.

We do not believe that on-cycle iron-hydride **1c** exists as a dimer because our previous studies[7,8] indicate that if we were to form an iron-hydride dimer this would instantaneously decompose, releasing hydrogen gas and forming the iron(II) species **1b**. We have carefully undertaken several studies on **1b**, specifically by EPR, which indicate that it does not react in a manner that is analogous to the reactivity of **1a**.

It is also worth noting that if **1c** dimerised and thus formed **1b**, then over the course of 10 to 30 minutes, **1b** would undergo ligand-based reduction to form iron(salan) complexes.[7] In the case of aryl nitro substrates in particular, the reaction is complete within 10 minutes at room temperature. This gives no opportunity for this onward ligand or catalyst activation process to take place. For alkyl nitro substrates, we have no evidence for the presence of iron(salan) complex formation. Therefore, we believe we activate from **1a**, form a mononuclear iron-hydride (**1c**) which is then intercepted by the highly reactive nitro compound, as per the interlocked catalytic cycles.

8.3. Kinetic Isotope Effect

8.3.1. Synthesis of DBpin

DBpin was synthesized by a modified literature procedure.[9] B_2pin_2 (1.00 g, 3.94 mmol) and Raney Nickel (5 mol%) were placed under an atmosphere of D_2 gas and the solids were agitated at 80 °C for 16 h. The resulting suspension was allowed to cool to room temperature and the flask was backfilled

with D_2 again. The suspension was stirred at 80 °C for another 16 h, and the neat DBpin was collected by filtration. Yield: 0.92 g (91%).

^{1}H NMR (C_6D_6, 400 MHz): δ 0.99 (s, 12H).

^{11}B NMR (C_6D_6, 128 MHz): δ 28.4 (t, 1 JBD = 21). Data are consistent with the literature.[10]

8.3.2. KIE NMR Reactions and Results

A comparison of concentration versus time for the reduction of $iPrNO_2$ in the presence of HBpin/DBpin shows almost identical reactivity:

KIE(H/D) = 1.13 ± 0.09

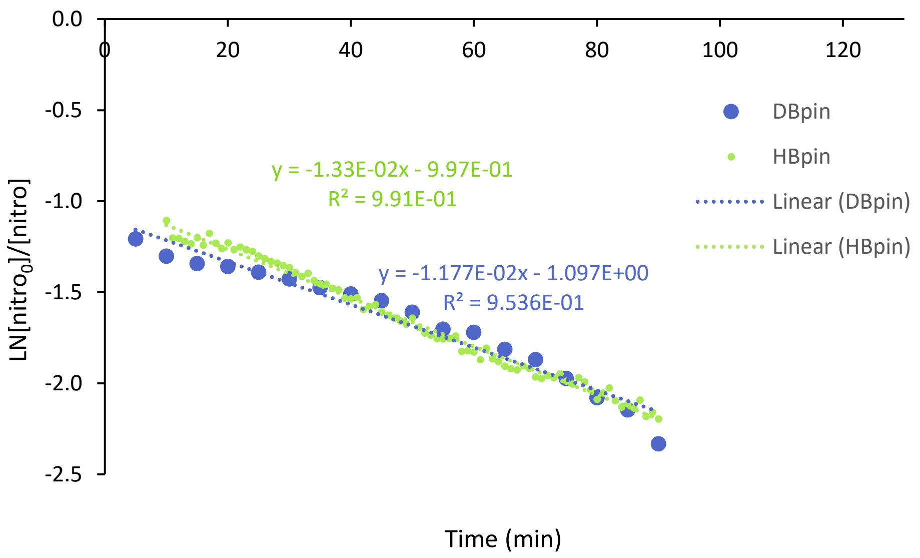

Figure S80: LN[nitro$_0$]/[nitro] plotted against time (min) for HBpin and DBpin giving KIE(H/D) = 1.13 ± 0.09.

8.4. Order with respect to HBpin

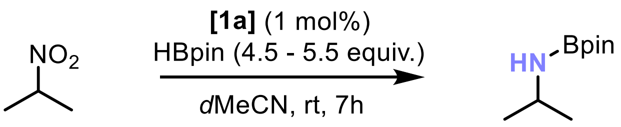

Kinetic studies were undertaken using the general method for the reduction of aliphatic nitro-compounds (Section 4.3) using [**1a**] (1 mol%), 2-nitropropane (1 equiv.) with different amounts of the pinacol borane in 0.5 mL CD_3CN at RT. Reactions were monitored by consumption of substrate vs. a dimethyl carbonate internal standard using in-situ NMR analysis (^{1}H NMR, CD_3CN, 400 MHz).

VTNA using a zero-order dependency shows a reasonable fit for the 4.5 and 5.0 equiv. loadings (See figure S81).

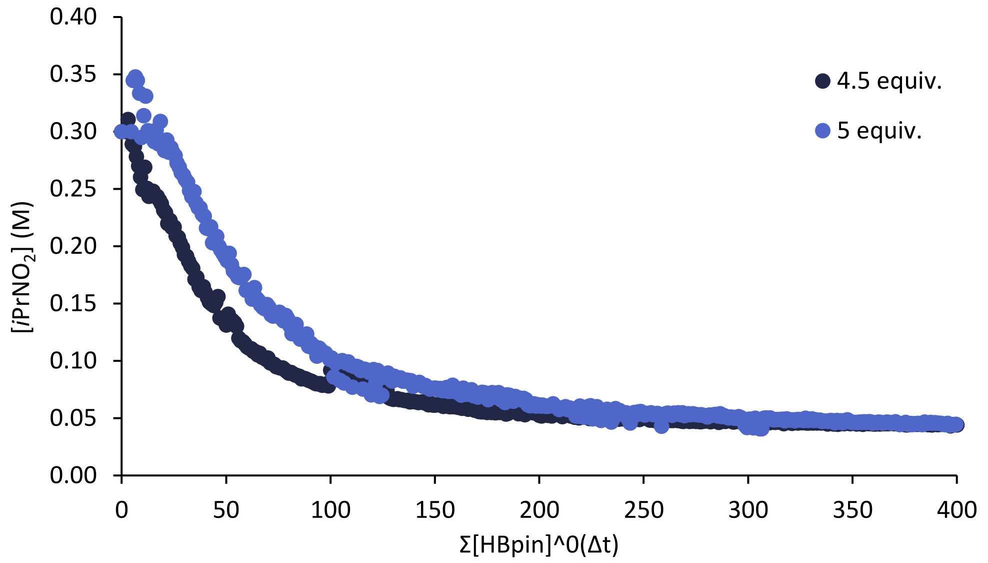

Figure S81: Zero order VTNA plot showing the loss of iPrNO2 at 4.5 equiv. HBpin (dark blue plot) and 5.0 equiv. HBpin (light blue plot).

When a 1st order plot is attempted, a poorer fit is obtained:

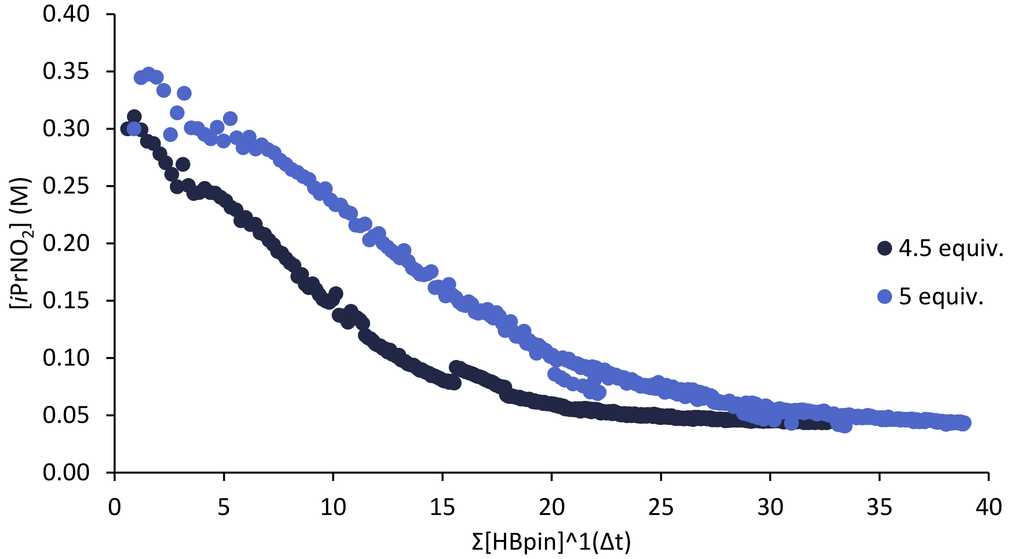

Figure S82: 1st order VTNA plot showing the loss of iPrNO2 at 4.5 equiv. HBpin (dark blue plot) and 5.0 equiv. HBpin (light blue plot).

Since VTNA should not require pseudo 1st order conditions to give an accurate output, we believe the closest approximation (zero order in HBpin) is the best possible fit. The lack of *exact* fit at zero order could be linked to the complexities of our interlocked cycles in that they do not conform to an idealised A + B → C reaction.

A zero order plot at LN(rate) for 4.5, 5.0 and 5.5 equiv. gives a decent line that is ostensibly flat (i.e. gradientless). We had to remove the initial data points from our concentration versus time plots due to paramagentism impeding line-fitting (the effect of paramagnetism can be seen in the data previously in Figures S74 and S82, particularly the 5.0 equiv. plot).

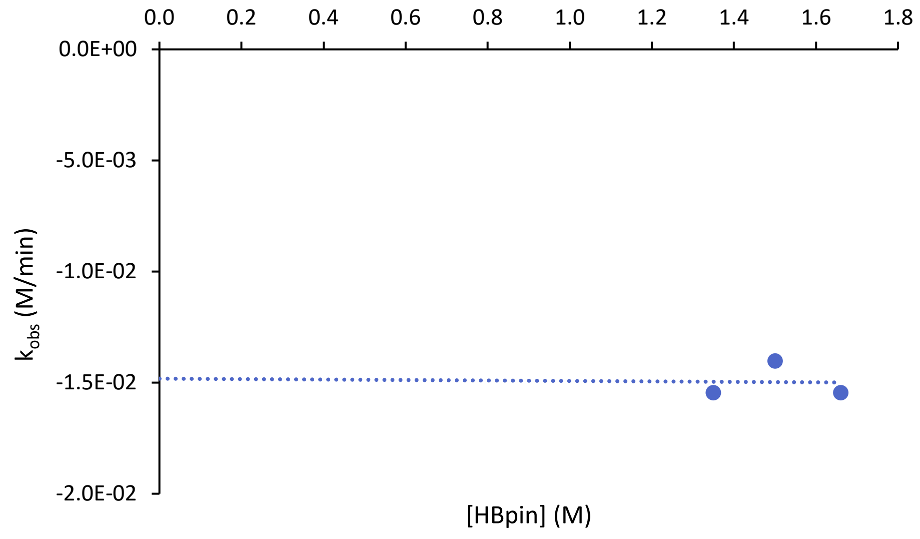

Figure S83: Reaction rate (M/min) versus HBpin concentration (M)

For completeness, the LN[nitro0/nitro] versus time plots are as follows:

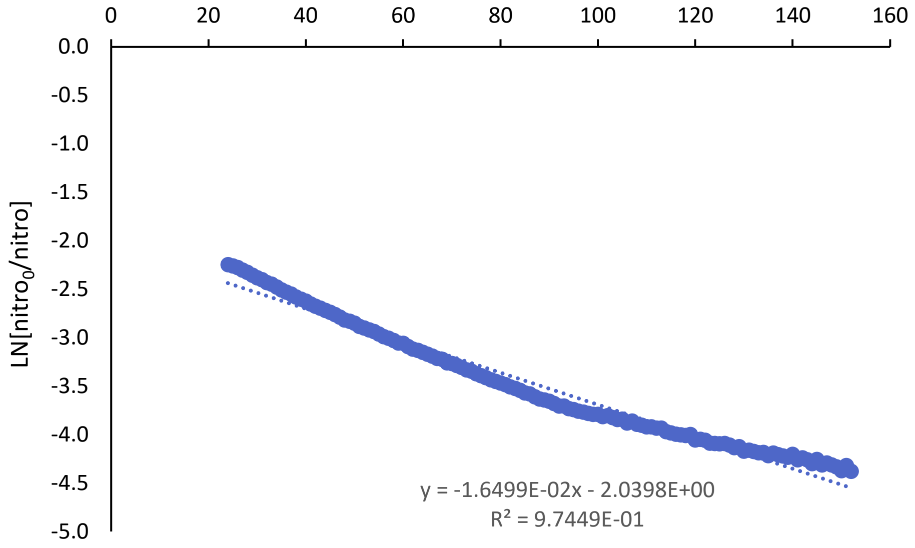

Figure S84: LN plot for uptake iPrNO2 at 5.5 equiv. HBpin. Initial data points lost due to paramagnetism causing extreme line broadening. Also note the plot may be starting to deviate from pseudo 1st order behaviour, as the loading of HBpin is higher in this instance.

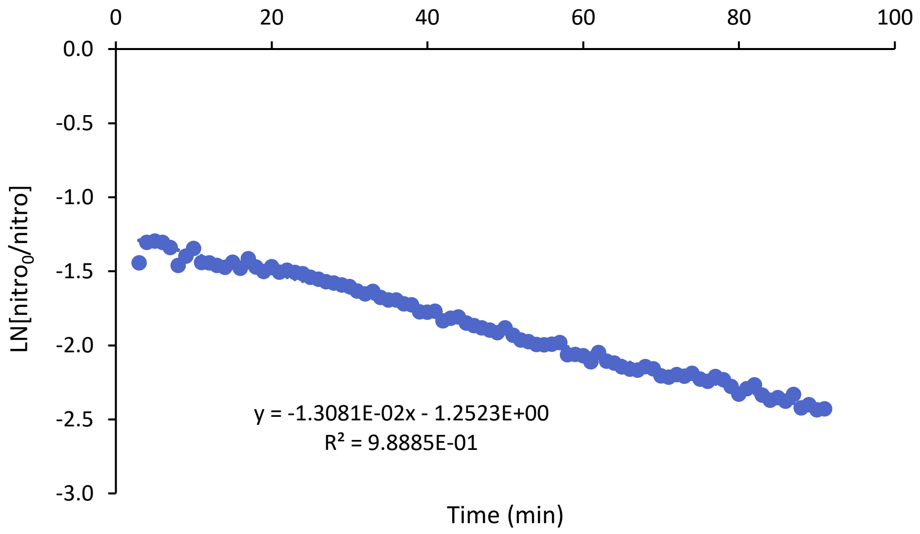

Figure S85: LN plot for uptake iPrNO2 at 5.0 equiv. HBpin.

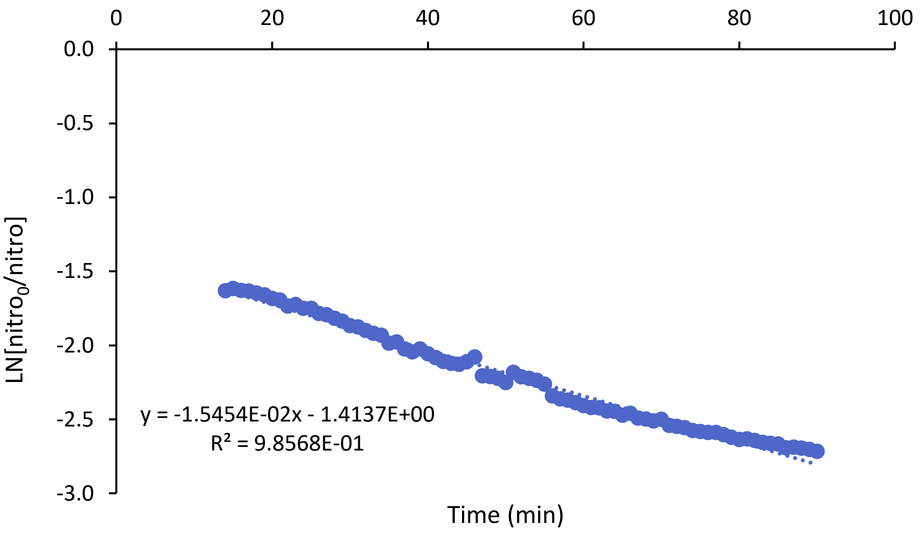

Figure S86: LN plot for uptake iPrNO2 at 4.5 equiv. HBpin. Initial data points lost due to paramagnetism causing extreme line broadening.

To further interrogate the data, we have attempted VTNA fits using pseudo 1st order conditions i.e. we have used high loadings of HBpin (6.75, 7.5 and 10 equiv.). As expected, the reaction is not zero order in HBpin at the higher loadings:

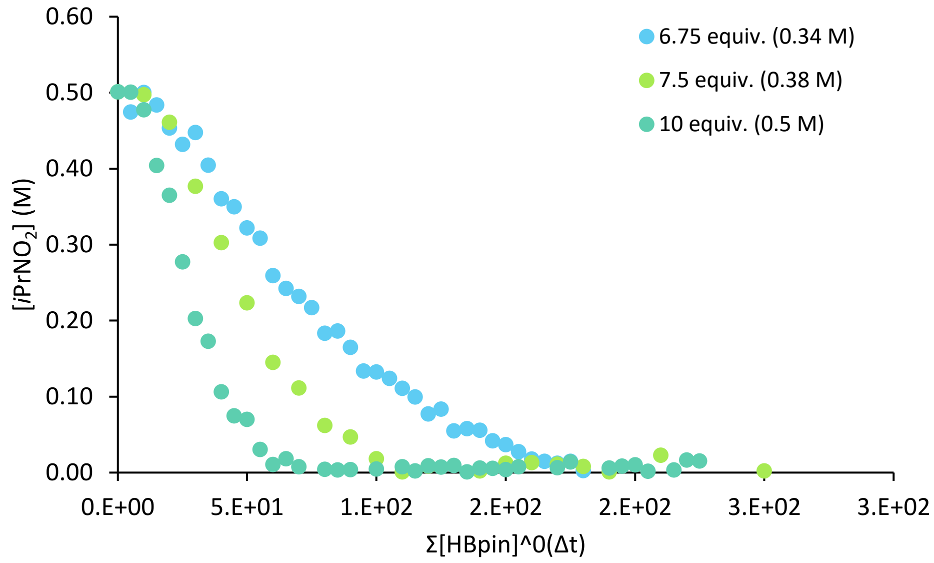

Figure S87: Zero order VTNA plot showing the loss of iPrNO2 at 6.75 equiv. HBpin, 7.5 equiv. HBpin and 10 equiv. HBpin.

If we take the high loading data and replot as a VTNA 1st order plot, then there is still no convincing overlap of the data, possibly indicting that the reaction is more complex than a simplified VTNA approach is able to deal with:

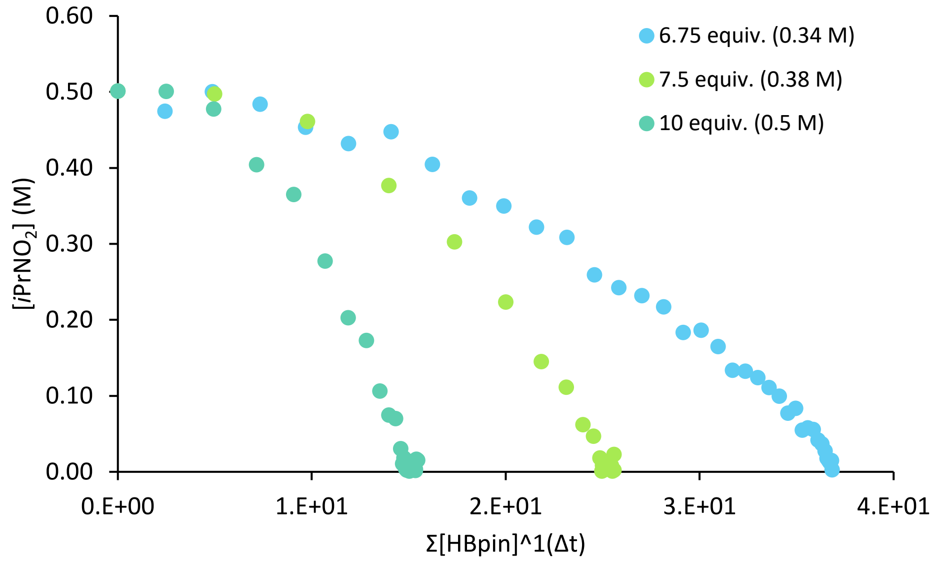

Figure S88: 1st order VTNA plot showing the loss of iPrNO2 at 6.75 equiv. HBpin, 7.5 equiv. HBpin and 10 equiv. HBpin.

For the 5.0 equiv. and 6.75 equiv. loadings, there is a reasonable overlap between zero and 1st order data, but zero order data provides a better visual match across the whole data set. It could be argued that as we move away from the optimised catalytic conditions that the interlocked cycles are based on, we are moving towards a different catalytic regime.

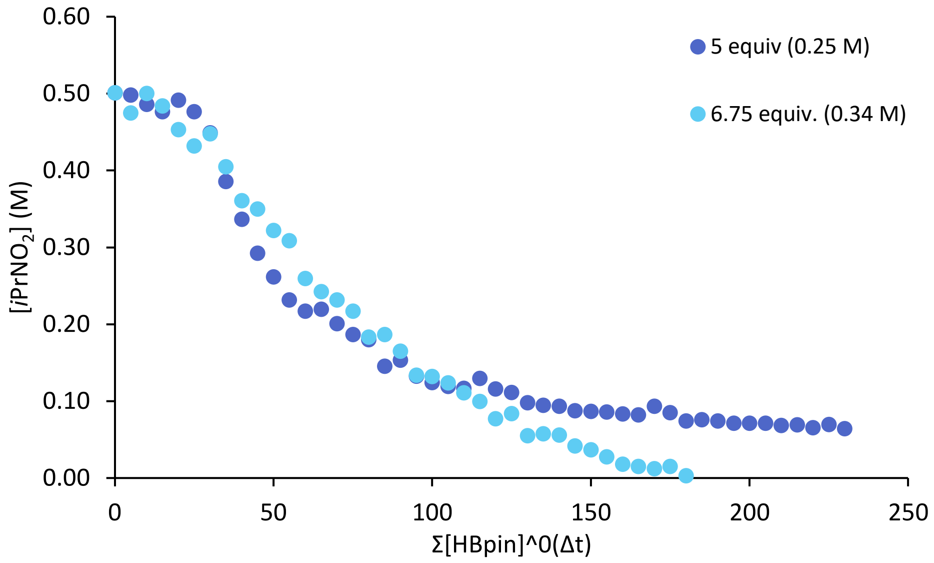

Figure S89: Zero order VTNA plot showing the loss of iPrNO2 at 5 equiv. HBpin and 6.75 equiv. HBpin.

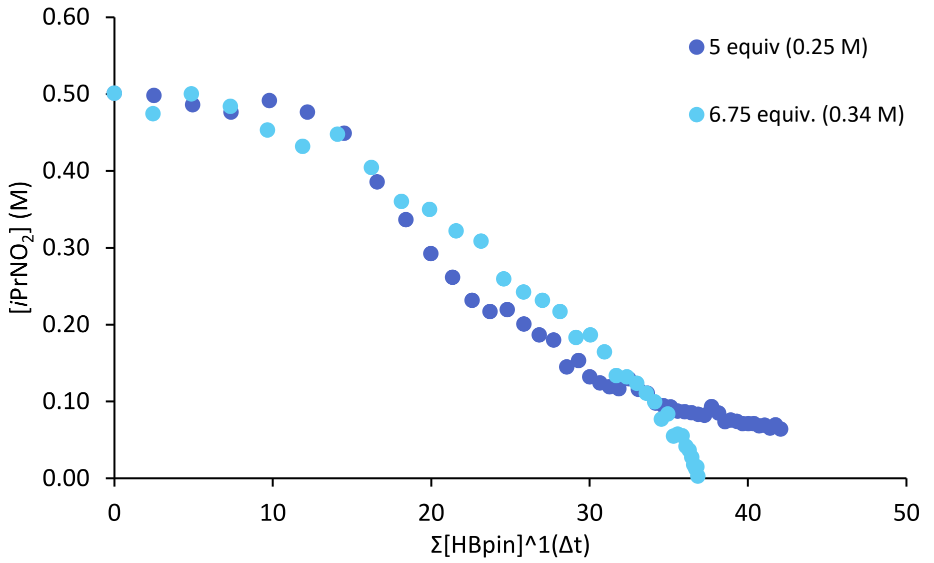

Figure S90: 1st order VTNA plot showing the loss of iPrNO2 at 5 equiv. HBpin and 6.75 equiv. HBpin.

Finally, treating all high HBpin loading data as second order gives the following charts- VTNA fails to give a match whereas LN plots give a reasonable second order fit. Since our standard reaction is not run with ≥ 6.76 equivalents HBpin the reason for these data sets and explaining the discrepancy between VTNA and integrated rate law data is beyond the scope of this study.

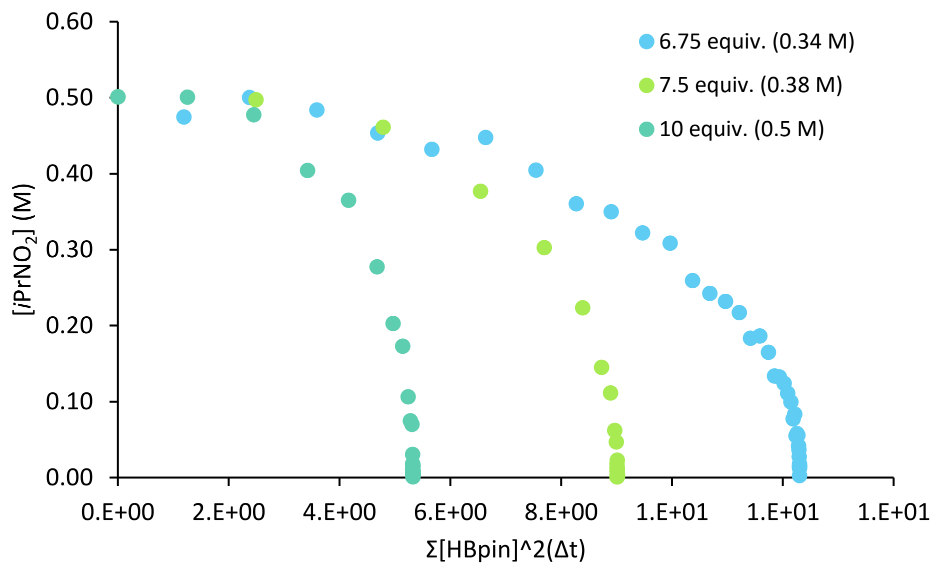

Figure S91: 2nd order VTNA plot showing the loss of iPrNO2 at 6.75 equiv. HBpin, 7.5 equiv. HBpin and 10 equiv. HBpin.

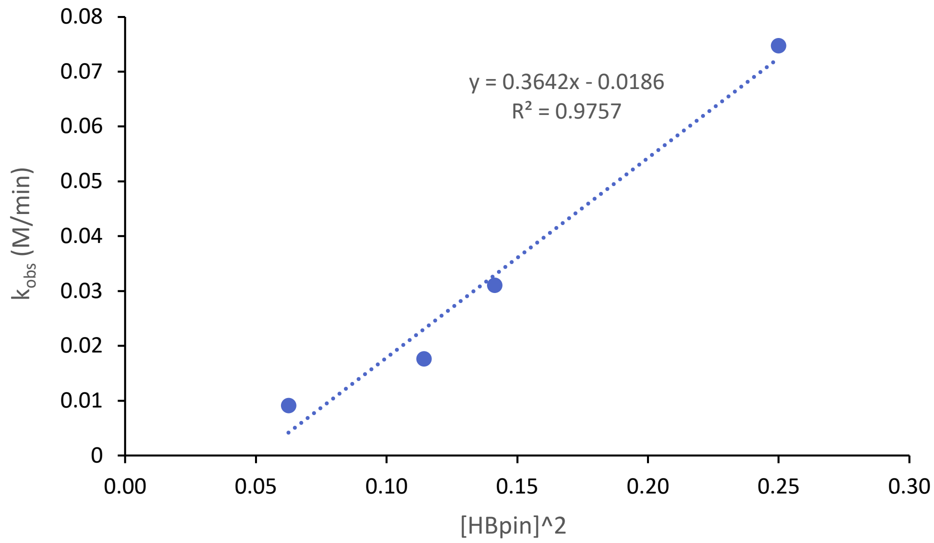

Figure S92: Second order plot of reaction rate (M/min) versus HBpin concentration (M)^2.

8.4.1. Summary of HBpin loading data

Our approximation of the reaction being zero order in HBpin under our standard catalytic conditions, we believe, is the best fit based on the combined VTNA and integrated rate law kinetics, EPR and DFT data. This also provides a good match to the overall appearance of the raw concentration versus time data, which is approximately (pseudo) 1st order: therefore, the reaction is likely to be 1st order in one substrate (nitro, vide infra) and zero order in the other (HBpin). We have combined several analytical and computational techniques to postulate a set of interlocked cycles that are not, as far as we are aware, covered by textbook-based data. In short, at higher loadings of HBpin we believe we start to change the mechanism and thus the rate-limiting step of the reaction changes.

8.5. Order with respect to Substrate

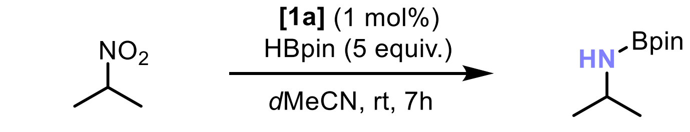

Scheme S3: Kinetics studying the order in nitro-compound.

Kinetic studies were undertaken using the general method for the reduction of aliphatic nitro-compounds (Section 4.3) using [**1a**] (1 mol%), pinacol borane (5 equiv.) with different amounts of the 2-nitropropane staring material in 0.5 mL CD_3CN at RT. Reactions were monitored by consumption of substrate vs. a dimethyl carbonate internal standard using in-situ NMR analysis (^{1}H NMR, CD_3CN, 400 MHz).

Taking 0.34 M and 0.38 M nitro data, normalising to allow for the use of nitro uptake data to generate order in nitro data, and subjecting them to a 1st order VTNA plot gives a good overlap. Note that initial data points were lost due to excessive line broadening.

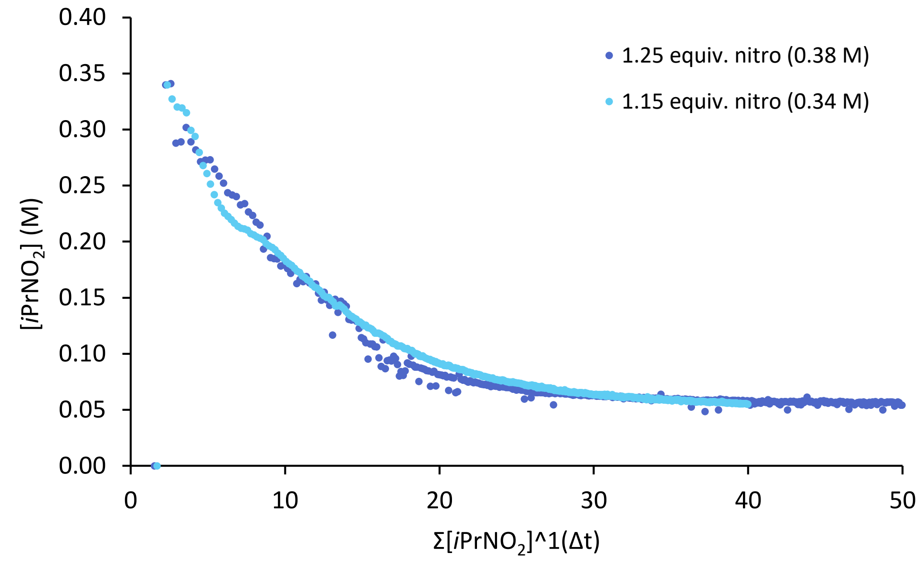

Figure S93: 1st order VTNA plot showing the loss of $iPrNO_2$ at 0.34 and 0.34 M $iPrNO_2$ (data normalised to 0.34 M nitro to generate VTNA plot). Y-axis: ● = [$iPrNO_2$] – 0.04 M; ● = [$iPrNO_2$].

A traditional LN plot supports the 1st order VTNA plot. Across both initial (fast, first regime) rate data and later (slower, second regime):

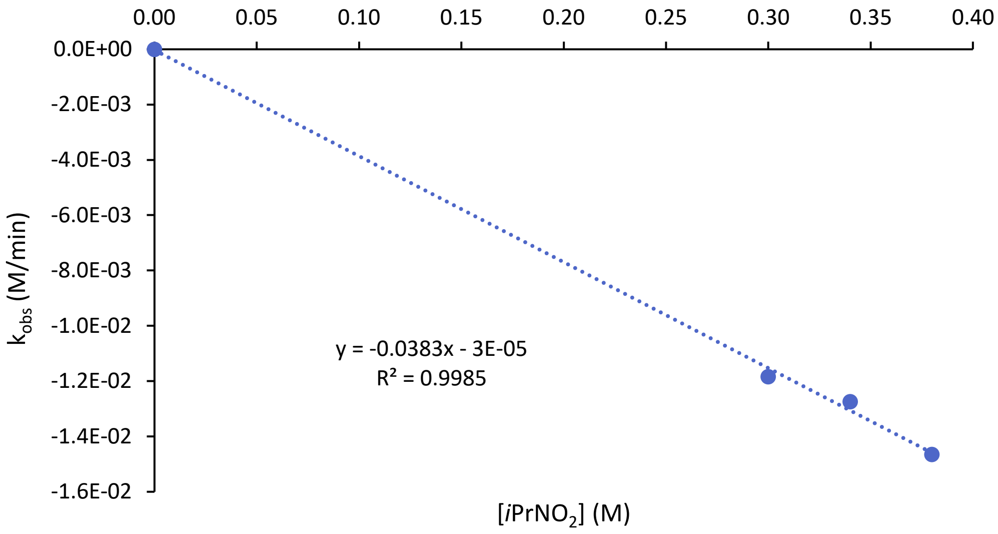

Figure S94: Reaction rate (M/min) versus iPrNO2 concentration (M) (taken from approximately t = 0 to 100 mins, depending on length of 'fast' reaction rate).

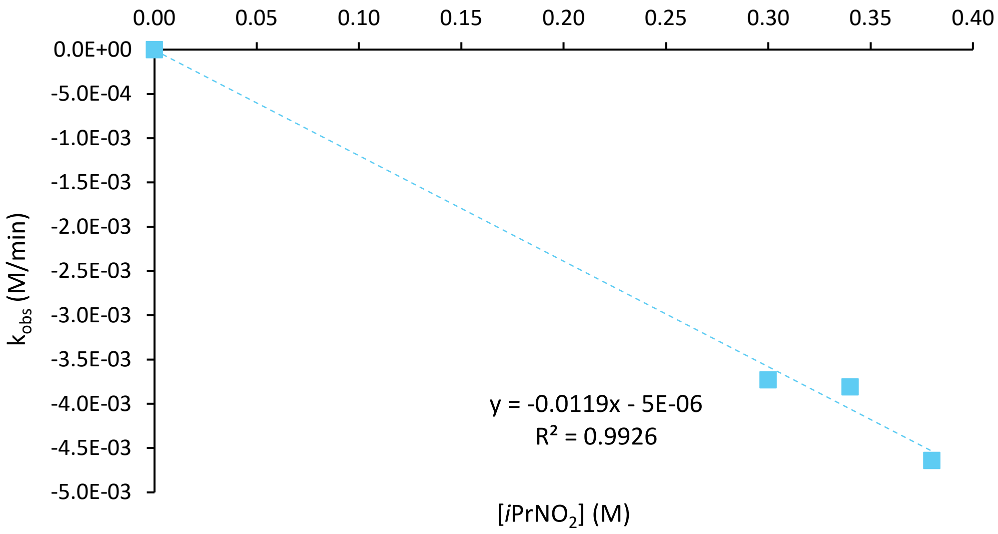

Figure S95: Reaction rate (M/min) versus iPrNO2 concentration (M) (taken from approximately t = 100 up to 250 mins, depending on length of 'slow' reaction rate).

For completeness, the concentration versus time plots highlighting the regions that were extracted for LN plots are as follows:

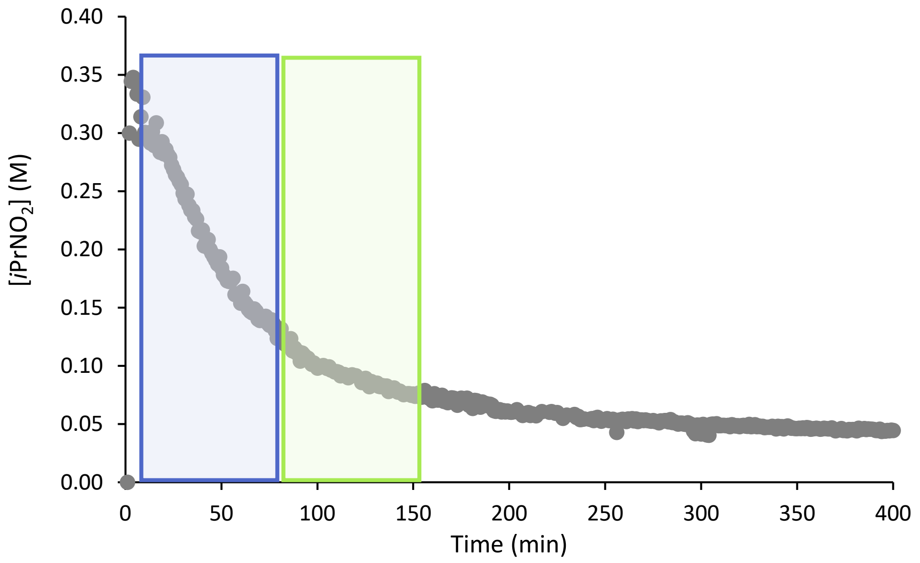

Figure S96: Raw data plot for iPrNO2 at 0.38 M. Initial data points were removed due to paramagnetism in 1H NMR spectrum.

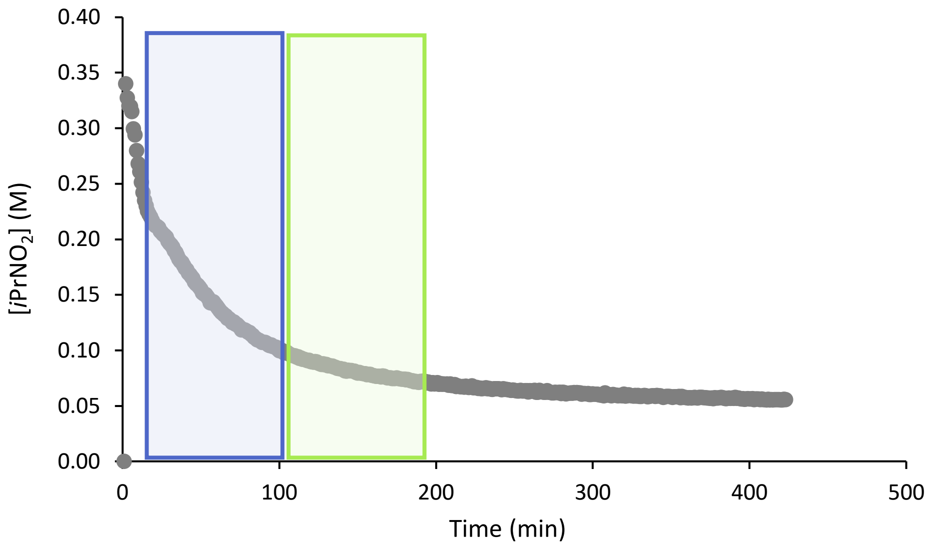

Figure S97: Raw data plot for iPrNO2 at 0.34 M. Initial data points were removed due to paramagnetism in 1H NMR spectrum.

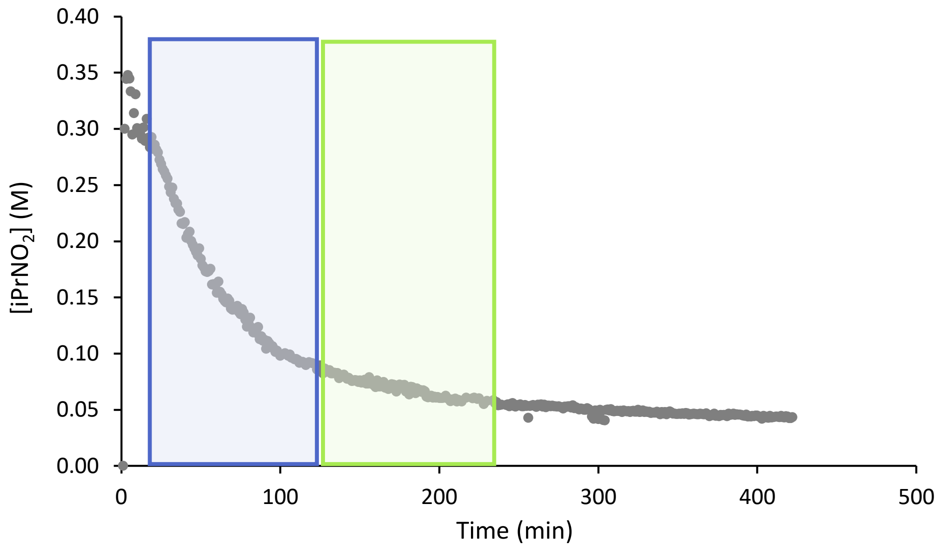

Figure S98: Raw data plot for iPrNO2 at 0.30 M. Initial data points were removed due to paramagnetism in 1H NMR spectrum.

This resulted in LN plots as follows:

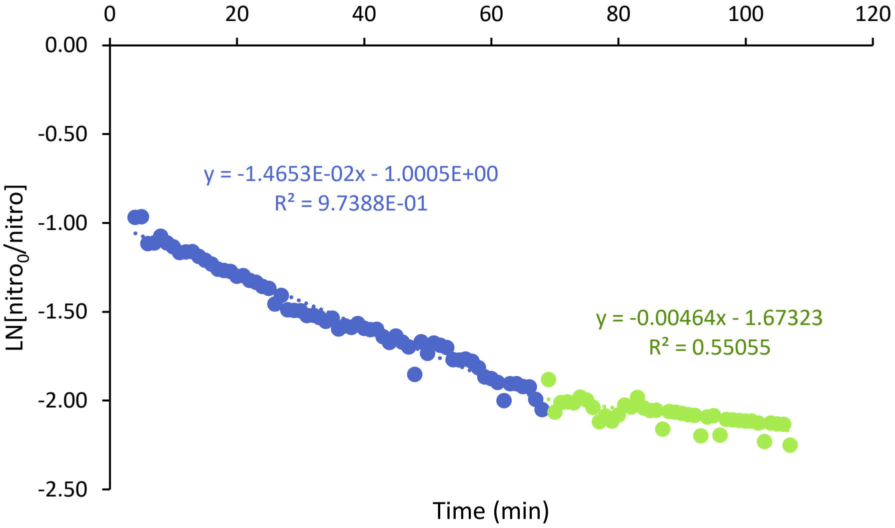

Figure S99: LN plot for iPrNO2 at 0.38 M. Note low R2 value for the slower (green) reaction regime is linked to intermittent loss of shimming (due to paramagnetism) over the data collection period.

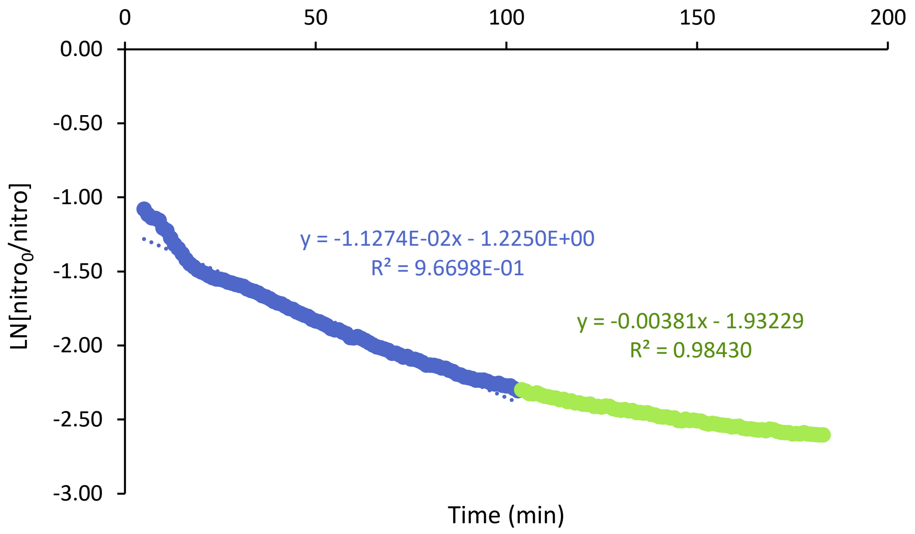

Figure S100: LN plot for iPrNO2 at 0.34 M

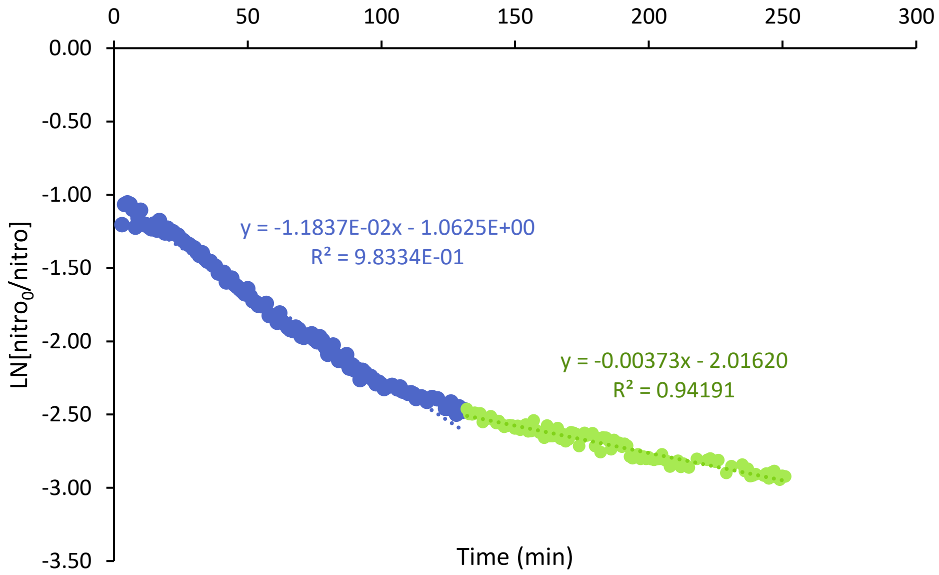

Figure S101: LN plot for iPrNO2 at 0.30 M

8.6. Arrhenius Analysis

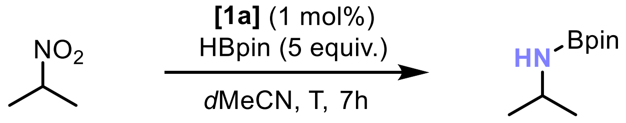

Arrhenius analysis was carried out by performing the optimized reaction for the reduction of aliphatic nitro-compounds (Section 4.3) using [**1a**] (1 mol%), 2-nitropropane (1 equiv.) with the pinacol borane (5 equiv.) in 0.5 mL CD_3CN at different temperatures in the range of 283K – 308K. Reactions were monitored by consumption of substrate vs. a dimethyl carbonate internal standard using in-situ NMR analysis (^{1}H NMR, CD_3CN, 400 MHz). The E_a was calculated using the equation $\ln(k) = -(E_a/RT) + \ln(A)$.

E_a= 78.8 kJ mol^{-1} ± 4.7

Table 3: Data obtained from reaction of 2-nitropropane with HBpin at varying temperatures used for Arrhenius Analysis.

T	k	k (s^{-1})	ln(k)	1/T
308	0.0355	0.000592	-7.43257	0.003247
298	0.01511	0.000252	-8.28674	0.003356
289	0.00446	7.43E-05	-9.50695	0.00346
283	0.00253	4.22E-05	-10.0739	0.003534

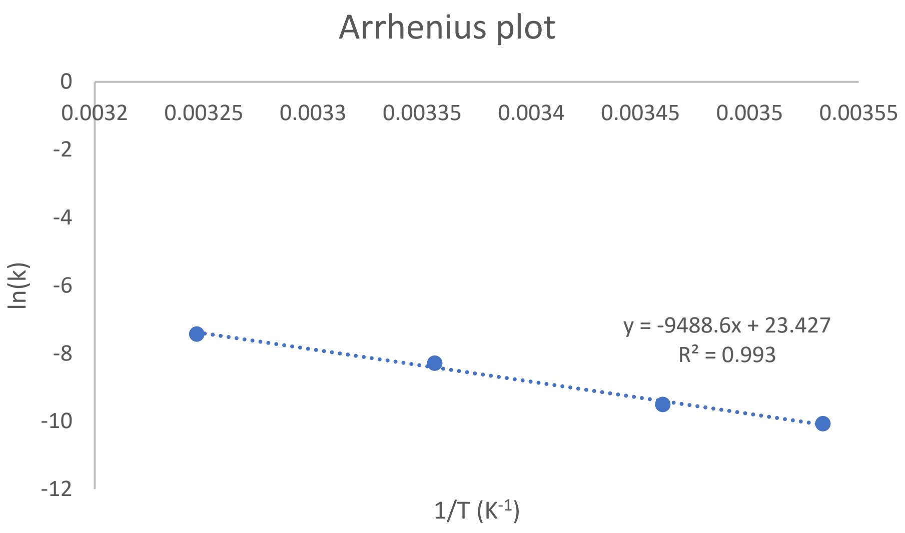

*Figure S102: Arrhenius plot for the direct reduction of 2-nitrtopropane using HBpin catalysed by **1a**.*

8.7. Eyring Analysis

Eyring analysis was carried out by performing the optimized reaction for the reduction of aliphatic nitro-compounds (Section 4.3) using [**1a**] (1 mol%), 2-nitropropane (1 equiv.) with the pinacol borane (5 equiv.) in 0.5 mL CD_3CN at different temperatures in the range of 283K – 308K. Reactions were monitored by consumption of substrate vs. a dimethyl carbonate internal standard using in-situ NMR analysis (^{1}H NMR, CD_3CN, 400 MHz). The entropy, enthalpy and Gibbs energy of activation were calculated using the equation $ln\ (kh/k_B)T = \frac{\Delta H^{\ddagger}}{RT} + \Delta S^{\ddagger}$ thus $\Delta G_{298}{}^{\ddagger} = \Delta H^{\ddagger} - T\Delta S^{\ddagger}$; with $\Delta H^{\ddagger}$ 76.36 kJ mol^{-1} ± 4.7, $\Delta S^{\ddagger}$ - 58.6 kJ mol^{-1} ± 16.0 and $\Delta G^{\ddagger}$ 93.8 ± 16.8 kJ mol^{-1}

Table 4: Data obtained from reaction of 2-nitropropane with HBpin at varying temperatures used for Eyring Analysis.

T	k	k (s^{-1})	ln(k)	ln(kh/k_bT)	1/T
313.2	0.004871	8.11883E-05	-9.41874	-38.9256	0.003192848
318.2	0.008429	0.000140477	-8.87047	-38.3931	0.003142678
323.2	0.011756	0.000195927	-8.53777	-38.076	0.003094059
328.2	0.017273	0.00028788	-8.15297	-37.7066	0.003046923

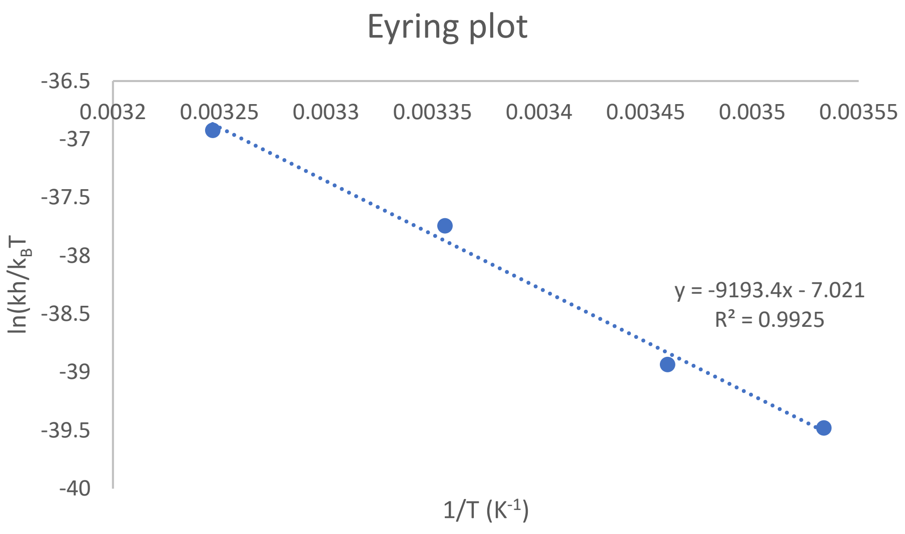

*Figure S103: Eyring plot for direct reduction of 2-nitropropane using HBpin catalysed by **1a**.*

9. Mass Spectrometry

In a nitrogen foiled glovebox, a sample of **1a** (13 mg, 0.02 mmol equivalent to 0.04 mmol of iron centres) was dissolved in THF (0.45 mL), then 50 µL (0.068 mmol) of a DBpin solution was added (4.08 mmol in 3 mL MeCN). The solution was shaken then 100 L was removed and added to 0.9 mL THF in a mass spectrometry vial. The sample was sealed and run using a Thermofisher Q Exactive within 20 minutes of sample preparation. Excess DBpin and a small quantity of HBpin impurity have led to small amount of **1b** and proteo-**1c** being observed in the mass spectrum. The major isotope observed belongs to deutero-**1c**. Data carried errors due to isotopic scrambling from the contamination from **1b** and proteo-**1c** that could not be deconvoluted.

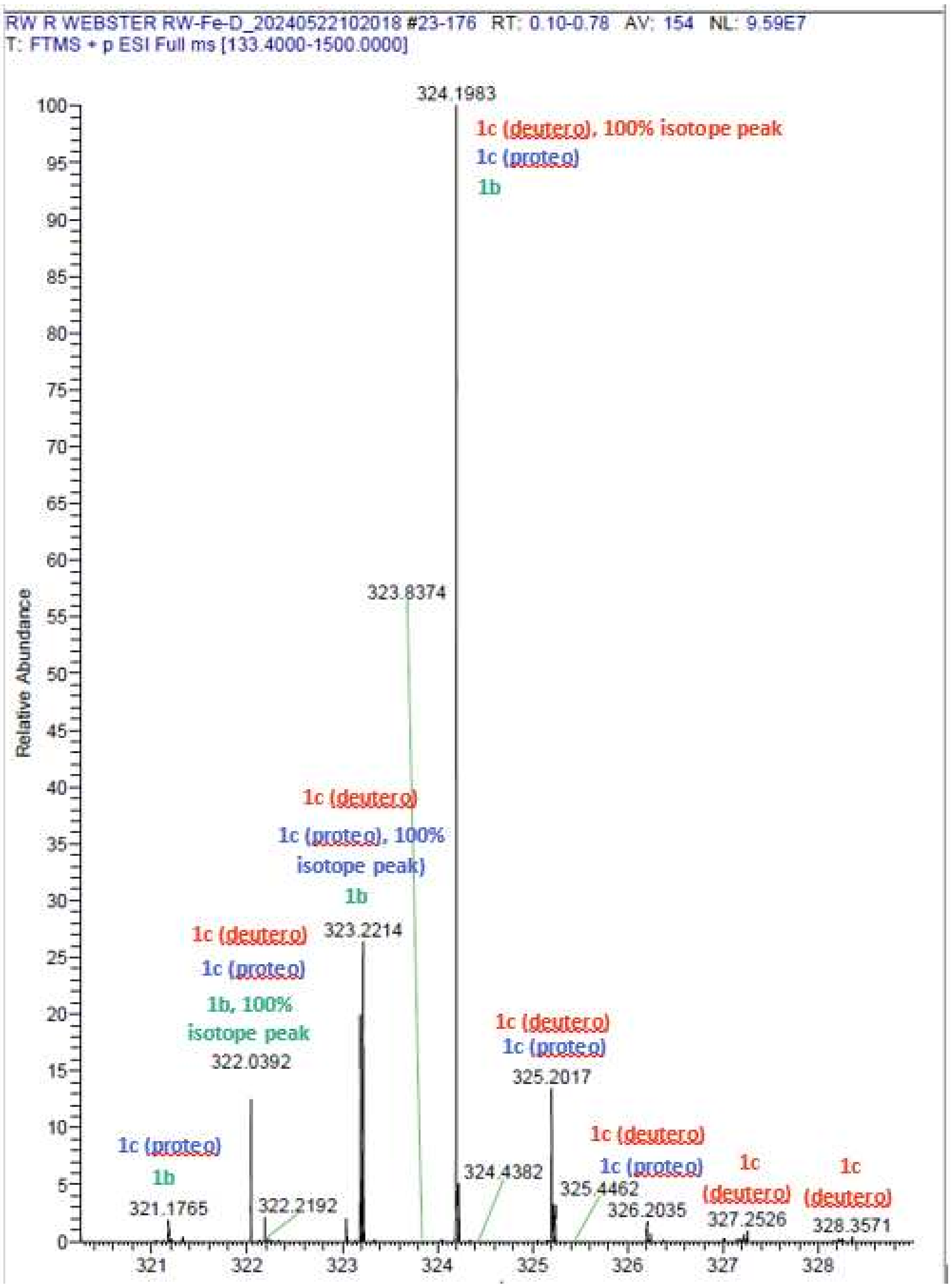

*Figure S104: Mass Spectrometry data to show the evidence of deutero-**1c** contaminated with **1b** and proteo-**1c.***

10. LIFDI-MS Data

CH_3CN is not compatible with LIFDI-MS, therefore the stoichiometric reactions were conducted in PhCN and THF (500 µL) at ambient temperature in an inert atmosphere glove box. Aliquots were taken periodically, and the LIFDI-MS recorded.

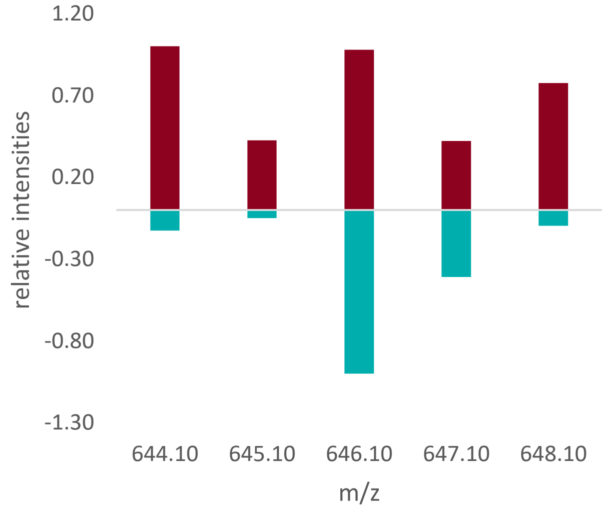

Figure S105: LIFDI-MS signal about m/z = 646 (top) and the simulated MS signal of Fe-H (bottom).

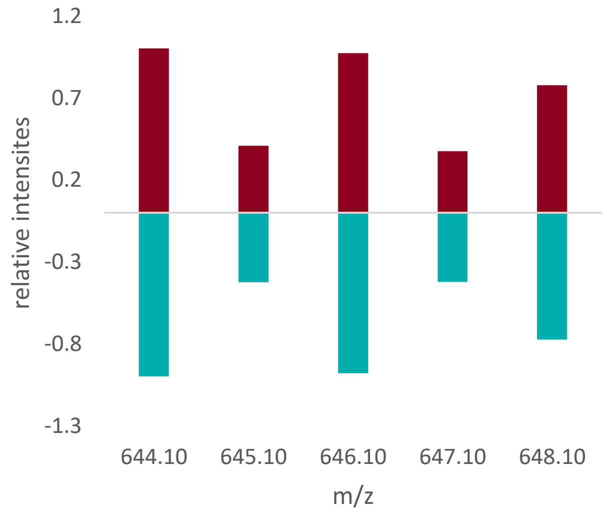

Figure S106: LIFDI-MS signal about m/z = 646 (top) and a satisfactory simulation of a mixture of molecular ions (m/z = 644, 646 and 648). The signals could not be confidently deconvoluted but without a molecular ion of m/z = 646 (Fe-H) the isotope pattern could not be simulated.

11. One-Pot Hydroaminations

11.1. Optimisation Table for One-Pot Hydroaminations

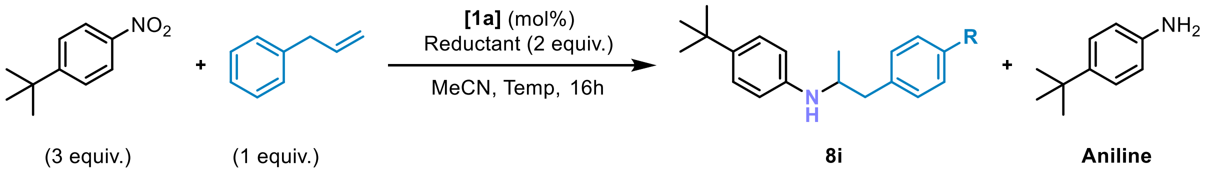

[1a] (mol%)	Concentration (M)	Temperature (°C)	silane	reaction time (h)	Conversion to aniline (%)	Conversion to 8i (%)	reaction time (h)	Conversion to aniline (%)	Conversion to 8i (%)
**1**	1	50	phenyl silane	6	8.5	0.0	22	25.5	1.0
**2**	1	50	phenyl silane	6	18.7	2.0	22	41.4	1.0
**1**	1	80	phenyl silane	6	41.9	5.0	22	40.0	31.0
**0.5**	1	80	phenyl silane	6	N/A	N/A	22	34.0	36.0
**1**	1	80	diphenyl silane	6	7.6	11.0	22	17.0	24.0
**1 Ar-Fe**	1	80	phenyl silane	6	65.6	11.0	22	95.0	25.0
**1 Ar-f-Fe**	1	80	phenyl silane	4	28.2	4.0	22	81.3	16.0
**1 *t* butyl-Fe**	1	80	phenyl silane	4	2.7	1.0	22	12.6	12.0
**1**	1	80	triethyl silane	6	0.0	0.0	22	0.0	0.0
**0.5**	0.2	50	phenyl silane	6	4.5	0.0	22	4.7	0.0
**0.5**	2	50	phenyl silane	6	5.6	0.0	22	12.2	1.0
**2.5**	0.2	50	phenyl silane	6	22.8	0.0	22	37.4	1.0
**2.5**	2	50	phenyl silane	6	23.8	1.0	22	56.3	9.0
**0.5**	0.2	80	phenyl silane	6	8.2	0.0	22	31.3	6.0
**0.5**	2	80	phenyl silane	6	27.8	11.0	22	49.0	47.0
**2.5**	0.2	80	phenyl silane	6	46.0	4.0	22	32.0	25.0
**2.5**	2	80	phenyl silane	6	34.6	22.0	22	23.0	28.0
**1.5**	1.1	65	phenyl silane	6	23.6	1.0	22	32.0	21.0
**1.5**	1.1	65	phenyl silane	6	24.6	1.0	22	43.0	17.0
**0**	2	80	Phenyl silane	-	-	-	22	0.0	0.0
**0.5**	2	80	None	-	-	-	22	0.0	0.0

Reaction Conditions: *t-butyl*nitrobenzene (1 mmol), allylbenzene (3 equiv.), Spec yields calculated from UPLC calibrated against anisole (0.5 equiv.) (internal standard).

11.2. Graphical Evidence to demonstrate that product distribution can be linked to alkene LUMO energy.

*Chart S1: The ability of **1a** to undertake HA catalysis is inherently linked to the LUMO energy of the alkene coupling partner.*

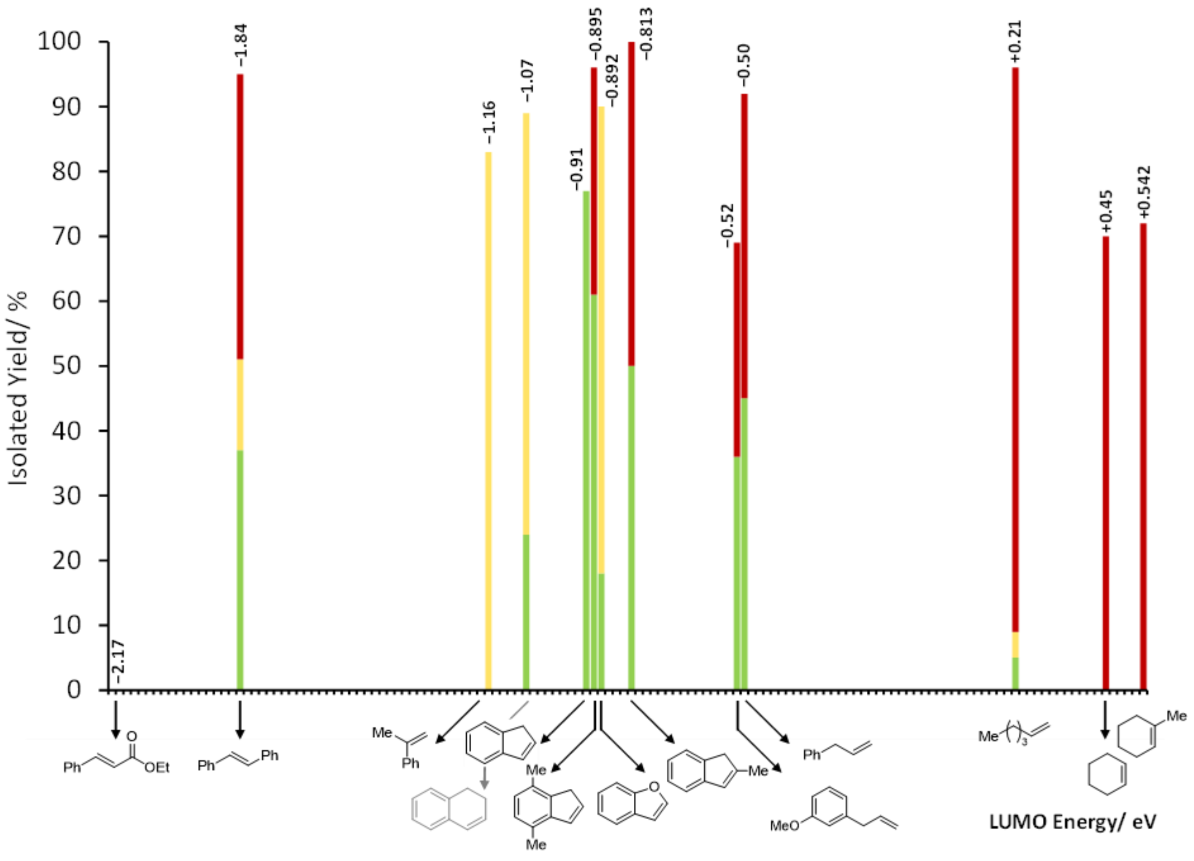

Chart S2: Highest singly occupied molecular orbital (α spin SOMO) of sextet high-spin 1C plotted at an isovalue of ±0.05 $a_0^{-3/2}$ (left). Spin density plot at 0.01 a_0^{-3} (right).

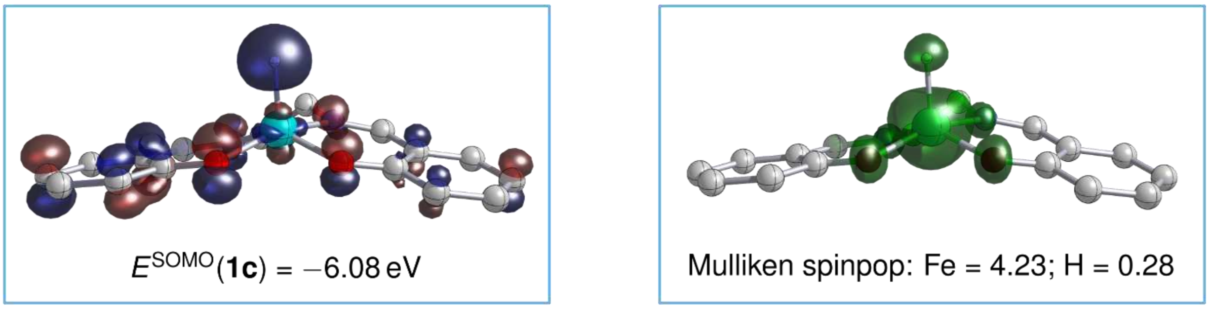

11.3. Hydroamination Mechanistic Control Reactions

To an ampule fitted with a magnetic stirrer bar under nitrogen was added a mixture of **1a** (0.005 mmol, 3 mg) and donor olefin (3 mmol) in dry acetonitrile (0.5 mL). To the solution was then added phenyl silane (2 mmol, 2 equiv.) The reaction mixture was then stirred at 80 °C under a positive flow of nitrogen for 22h. The solvent was then removed *in vacuo* and an NMR was run of the reaction mixture in $CDCl_3$.

11.3.1. Subjecting 1-hexene to standard hydroamination conditions

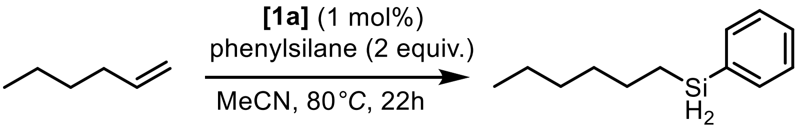

Figure S107: subjecting 1-hexene to standard hydroamination reaction conditions in the absence of nitro-compound.

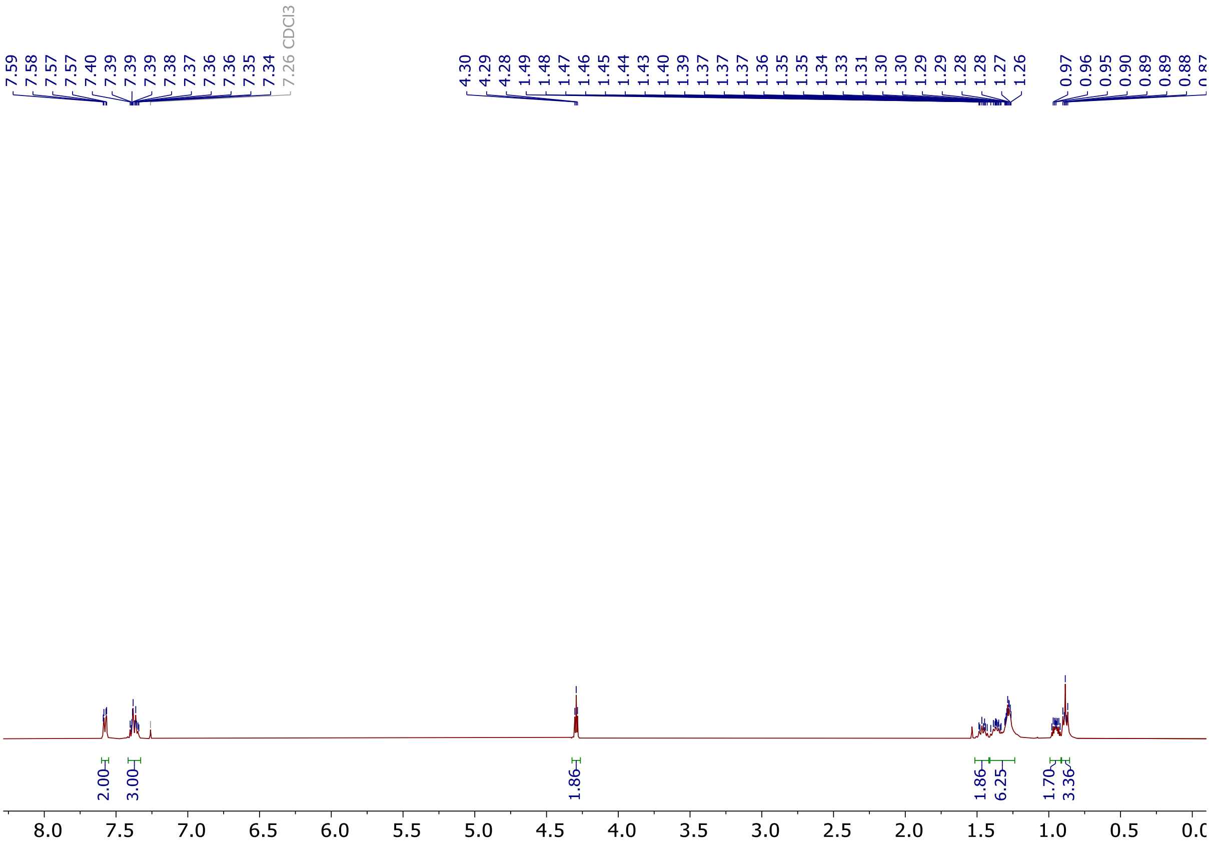

Figure S108: ^{1}H NMR Spectrum of reaction of 1-hexene under standard reaction conditions in $CDCl_3$.

Without the presence of a nitro compound reactivity switched to show a new hydrosilyation reaction taking place giving rise to quantitative conversion of 1-hexene to the anti-Markovnikov hydrosilyation product (in good agreement with the literature).[11]

^{1}H NMR (400 MHz, $CDCl_3$) δ 7.58 (dd, *J* = 7.6, 1.8 Hz, 2H), 7.37 (qd, *J* = 6.3, 4.3 Hz, 3H), 4.29 (t, *J* = 3.7 Hz, 2H), 1.51 – 1.42 (m, 2H), 1.41 – 1.33 (m, 2H), 1.29 (tt, *J* = 6.2, 2.1 Hz, 4H), 0.98 – 0.92 (m, 2H), 0.88 (t, *J* = 6.8 Hz, 4H).

11.3.2. Subjecting allylbenzene to standard hydroamination conditions

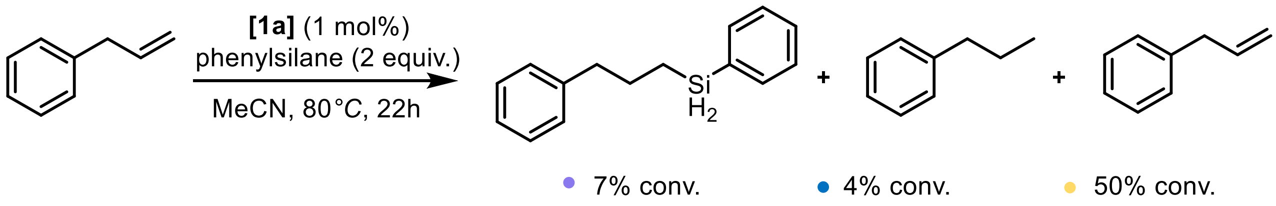

Figure S109: subjecting allylbenzene to standard hydroamination reaction conditions in the absence of nitro-compound.

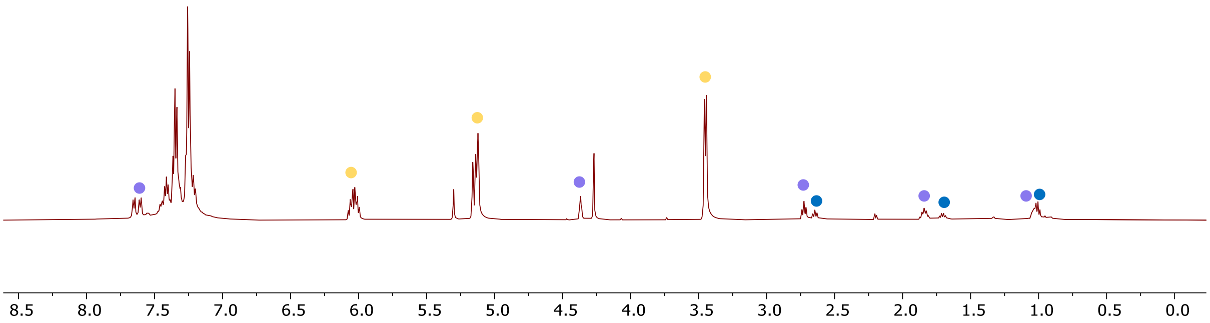

Figure S110: ^{1}H NMR Spectrum of reaction of allylbenzene under standard reaction conditions in $CDCl_3$.

Without the presence of nitro compound a mixture of products formed including unreacted allylbenzene, the anti-Markovnikov hydrosilyation product and some propyl benzene which is assumed to form by a HAT event.

11.3.3. Subjecting indene to standard hydroamination conditions

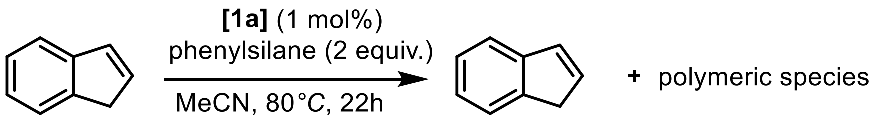

Figure S111: subjecting indene to standard hydroamination reaction conditions in the absence of nitro-compound.

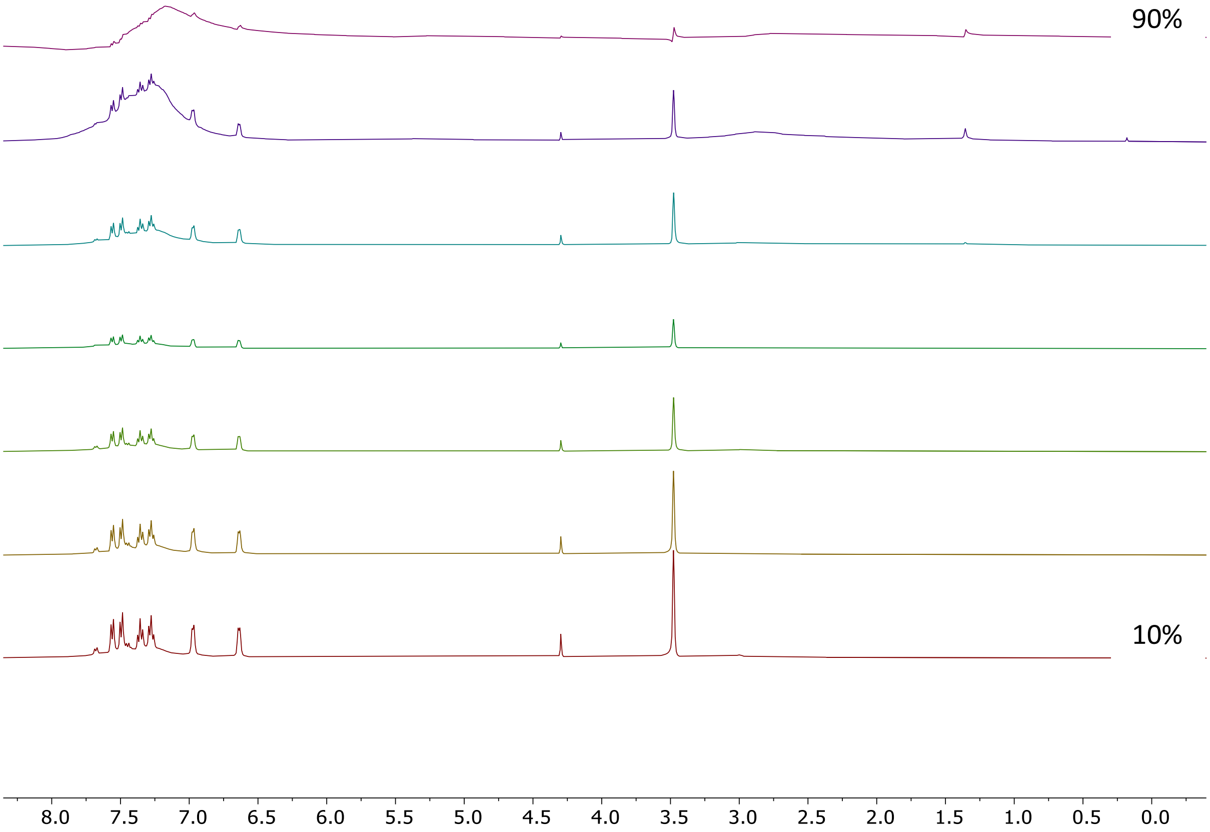

Figure S112: Individual traces from the DOSY spectrum of reaction of indene under standard reaction conditions in $CDCl_3$*, showing signal intensity as a function of gradient strength (increasing linearly from 10% to 90%).*

The ^{1}H NMR data suggest the presence of unreacted indene (giving rise to sharp signals) and polymer (giving broad signals), with significant overlap of the resonances for monomer and polymer. Evidence for this was provided by a DOSY spectrum: the individual ^{1}H data sets showed relatively fast decay of the sharp resonances (indene) with increasing gradient strength, whilst the underlying broad signals (polymer) were affected to a much lower extent.

The severe peak overlap of sharp and broad signals made obtaining a good quality 2D DOSY plot difficult, but reasonable fits were obtained using a two-component model, again consistent with the presence of monomer and polymer signals.

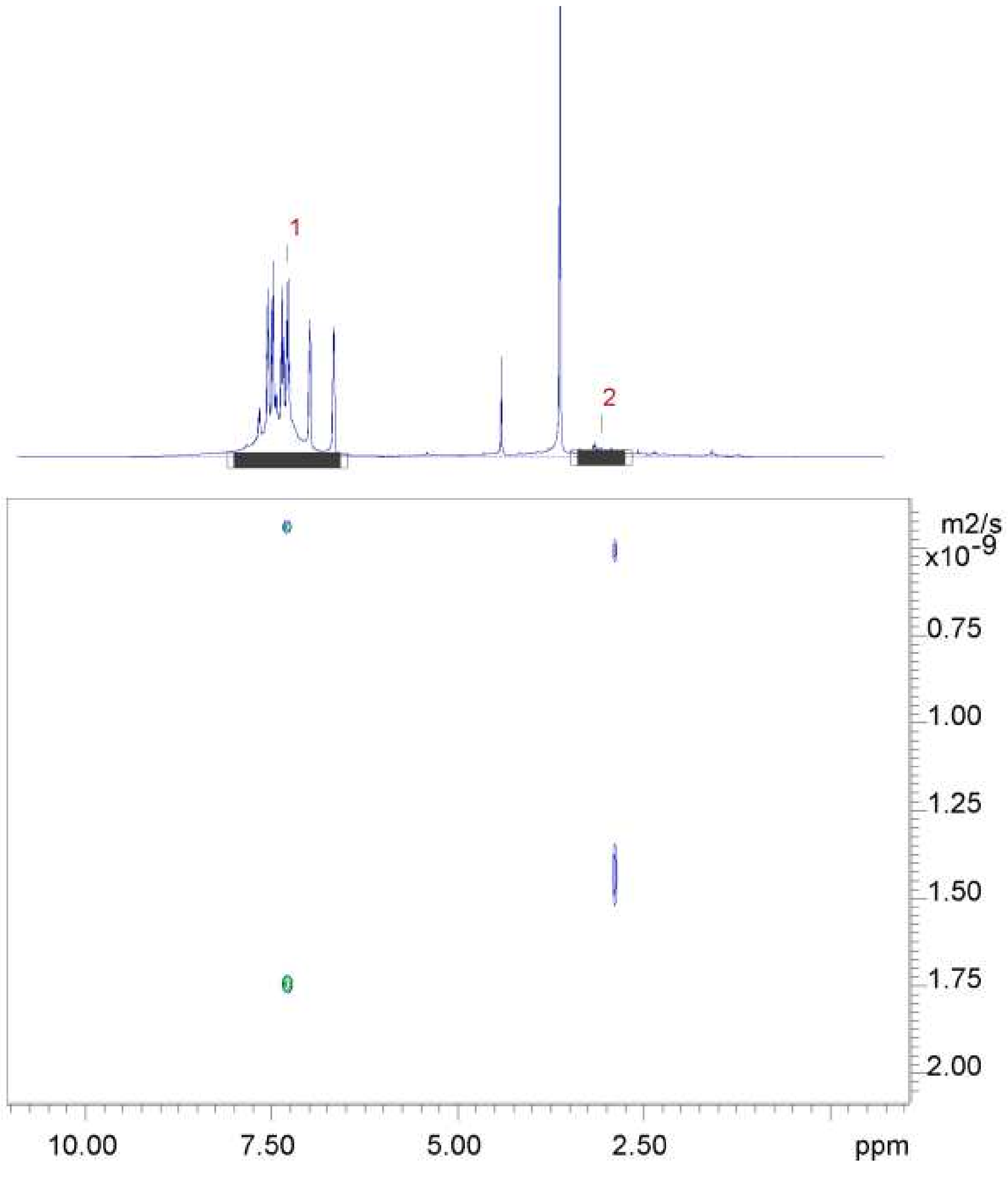

Figure S113: 2D DOSY NMR spectra of the reaction of indene under standard reaction conditions in $CDCl_3$ showing the presence of both monomeric and polymeric species.

A rough molecular weight was predicted using the SEGWE calculator: Diffusion coefficient and molecular weight estimation.[12]

With a diffusion coefficient of 4.48E-10 m^2s^{-1} a molecular weight of 2404 g mol^{-1} was predicted indicative of an oligomeric species.[12]

11.3.4. Subjecting *t*-butyl aniline to standard hydroamination conditions

To an ampule fitted with a magnetic stirrer bar under nitrogen was added a mixture of **1a** (0.005 mmol, 3 mg) and t-butyl aniline (1.0 mmol, 1 equiv.) in dry acetonitrile (0.5 mL). To the solution was then added allylbenzene (3 mmol, 3 equiv.) followed by phenyl silane (2 mmol, 2 equiv.) The reaction mixture was then stirred at 80 °C under a positive flow of nitrogen for 22h. After the reaction had gone to completion the solvents were removed under vacuum. The resulting crude products were then analysed by proton NMR spectroscopy.

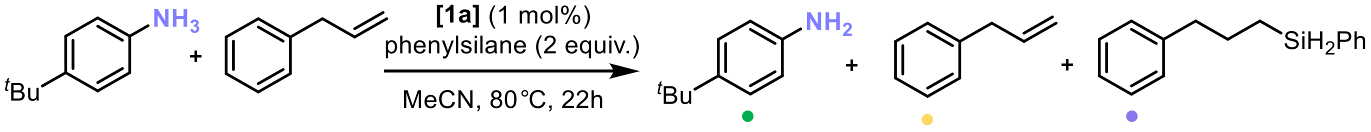

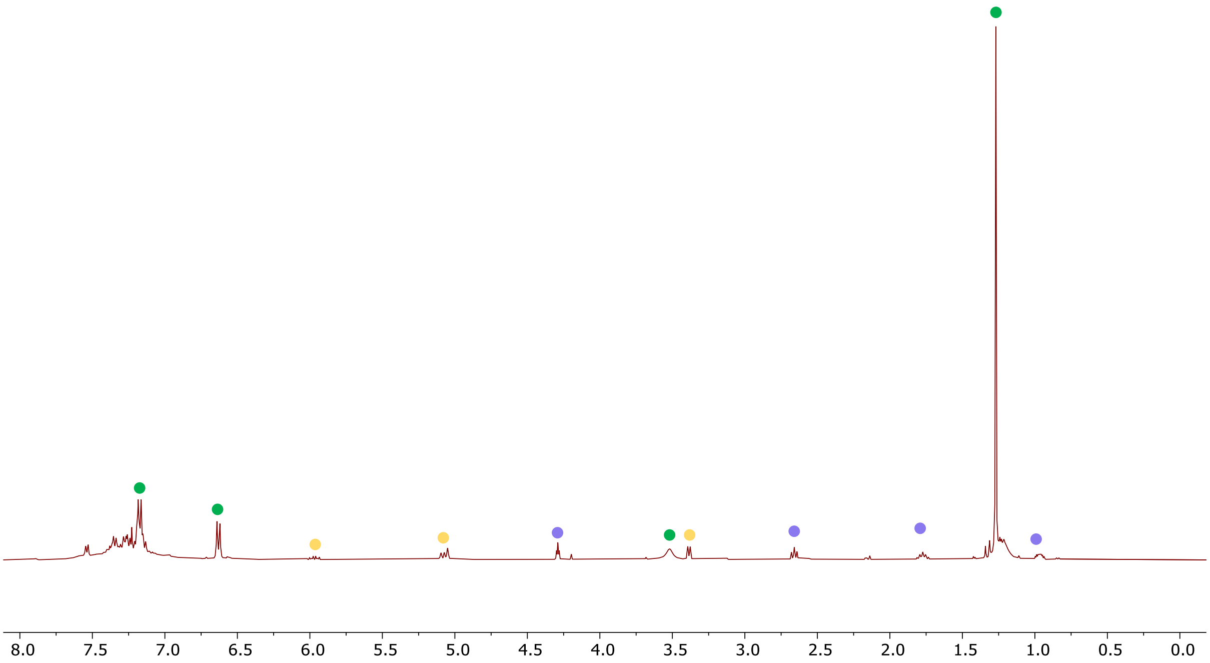

Figure S114: ^{1}H NMR Spectrum of reaction of t-butyl aniline under standard reaction conditions in $CDCl_3$.

This control was done to determine if aniline was an intermediate in the reaction pathway. However, subjection of aniline to the reaction conditions with allylbenzene did not give rise to the formation of any desired hydroamination product. This suggests that aniline is not involved in a productive reaction pathway indicating that nitro or nitroso-arene are more likely involved in the C-N bond forming event.

Data in agreement with literature.[13]

11.3.5. Subjecting nitroso benzene to standard hydroamination conditions

To an ampule fitted with a magnetic stirrer bar under nitrogen was added a mixture of **1a** (0.005 mmol, 3 mg) and nitroso benzene (1.0 mmol, 1 equiv.) in dry acetonitrile (0.5 mL). To the solution was then added indene (3 mmol, 3 equiv.) followed by phenyl silane (2 mmol, 2 equiv.) The reaction mixture was then stirred at 80 °C under a positive flow of nitrogen for 22h. After the reaction had gone to completion the solvents were removed under vacuum. The resulting crude product was then purified by flash column chromatography on silica using *iso*-hexane/dichloromethane.

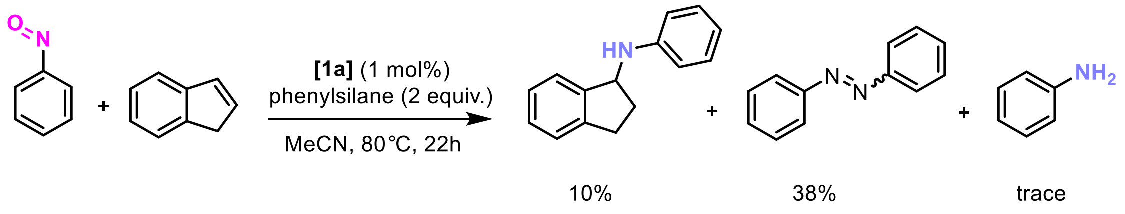

Figure S115: Reaction of nitroso benzene with indene under the standard hydroamination conditions.

Using nitroso benzene instead of nitrobenzene did give rise to some hydroamination product indicating a nitroso species could be an intermediate on route to the desired hydroamination product. However, it is worth noting that the product distribution is different to that of the corresponding reaction using nitro benzene support our theory that nitroso is a highly reactive intermediate and therefore is unlikely to build up in the reaction mixture.

11.4. General Procedure for the One-Pot Hydroaminations

To an ampule fitted with a magnetic stirrer bar under nitrogen was added a mixture of **1a** (0.005 mmol, 3 mg) and nitro-compound (1.0 mmol, 1 equiv.) in dry acetonitrile (0.5 mL). To the solution was then added donor olefin (3 mmol, 3 equiv.) followed by phenyl silane (2 mmol, 2 equiv.) The reaction mixture was then stirred at 80 °C under a positive flow of nitrogen for 22h. After the reaction had gone to completion the solvents were removed under vacuum. The resulting crude product was then purified by flash column chromatography on silica using *iso*-hexane/dichloromethane.

11.4.1. *Rac* 4-(tert-butyl)-N-(1,2-diphenylethyl)aniline **8a**

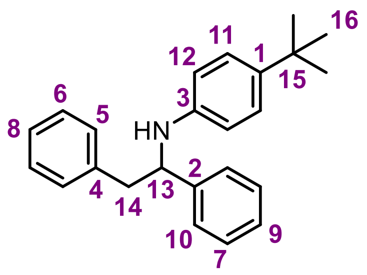

Yellow oil (86.6 mg, 37%)

R_f = 0.33 (30% DCM / 70% hexane)

^{1}H NMR (CDCl$_3$, 400 MHz) δ 7.29 – 7.16 (m, 8H, C$^{5-10}$-H), 7.07 (dd, J = 8.3, 1.6 Hz, 2H, C$^{6/7}$-H), 7.02 (d, J = 8.7 Hz, 2H, C^{11}-H), 6.35 (d, J = 8.7 Hz, 2H, C^{12}-H), 4.47 (dd, J = 8.5, 5.6 Hz, 1H, C^{13}-H), 3.98 (*br* s, 1H, NH), 3.05 (dd, J = 13.9, 5.6 Hz, 1H, C^{14}-H), 2.92 (dd, J = 13.9, 8.5 Hz, 1H, C^{14}-H), 1.15 (s, 9H, C^{16}-H).

^{13}C{^{1}H} NMR (CDCl$_3$, 101 MHz) δ 145.2 (C^{1}), 144.0 (C^{2}), 140.3 (C^{3}), 138.0 (C^{4}), 129.3 (C^{5}), 128.7 (C^{6}), 128.7 (C^{7}), 127.2 (C^{8}), 126.8 (C^{9}), 126.6 (C^{10}), 125.9 (C^{11}), 113.5 (C^{12}), 59.8 (C^{13}), 45.5 (C^{14}), 33.9 (C^{15}) 31.6 (C^{16})

HRMS (ESI+): calcd for [M, $C_{24}H_{27}N$]$^+$ 330.2216, found 330.2229.

IR (Neat): 3406, 2959, 1893, 1615, 1517, 818, 697 cm^{-1}.

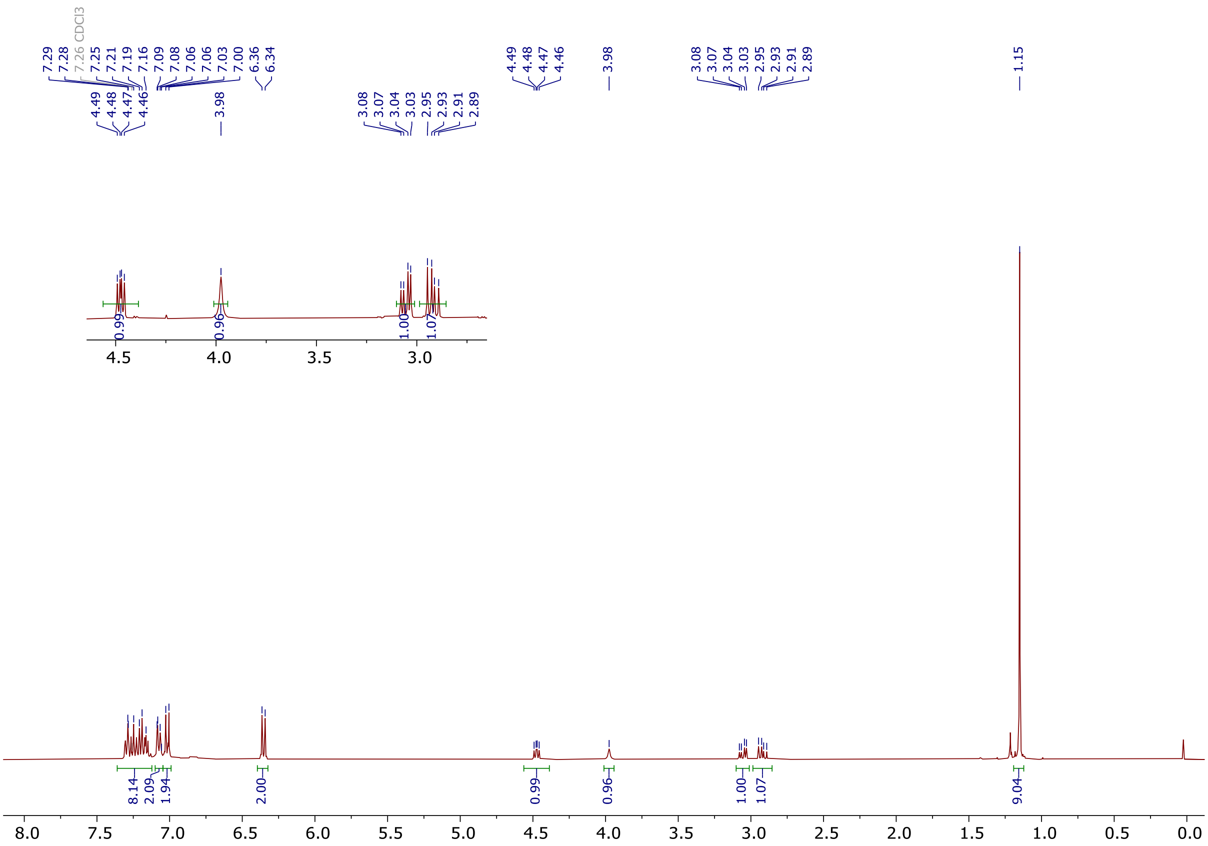

Figure S116: ^{1}H NMR Spectrum of 8a in CDCl$_3$ after isolation via column chromatography.

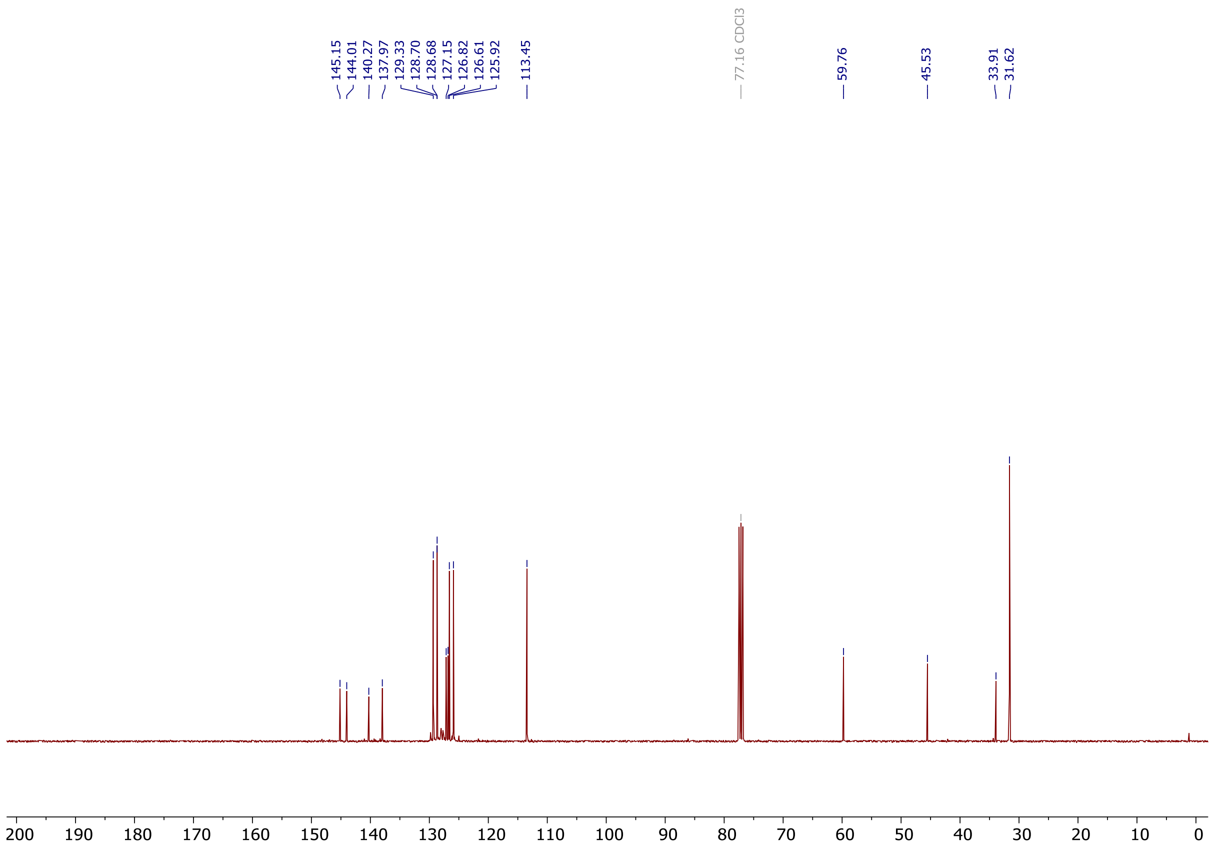

Figure S117: ^{13}C NMR Spectrum of 8a in $CDCl_3$ after isolation via column chromatography.

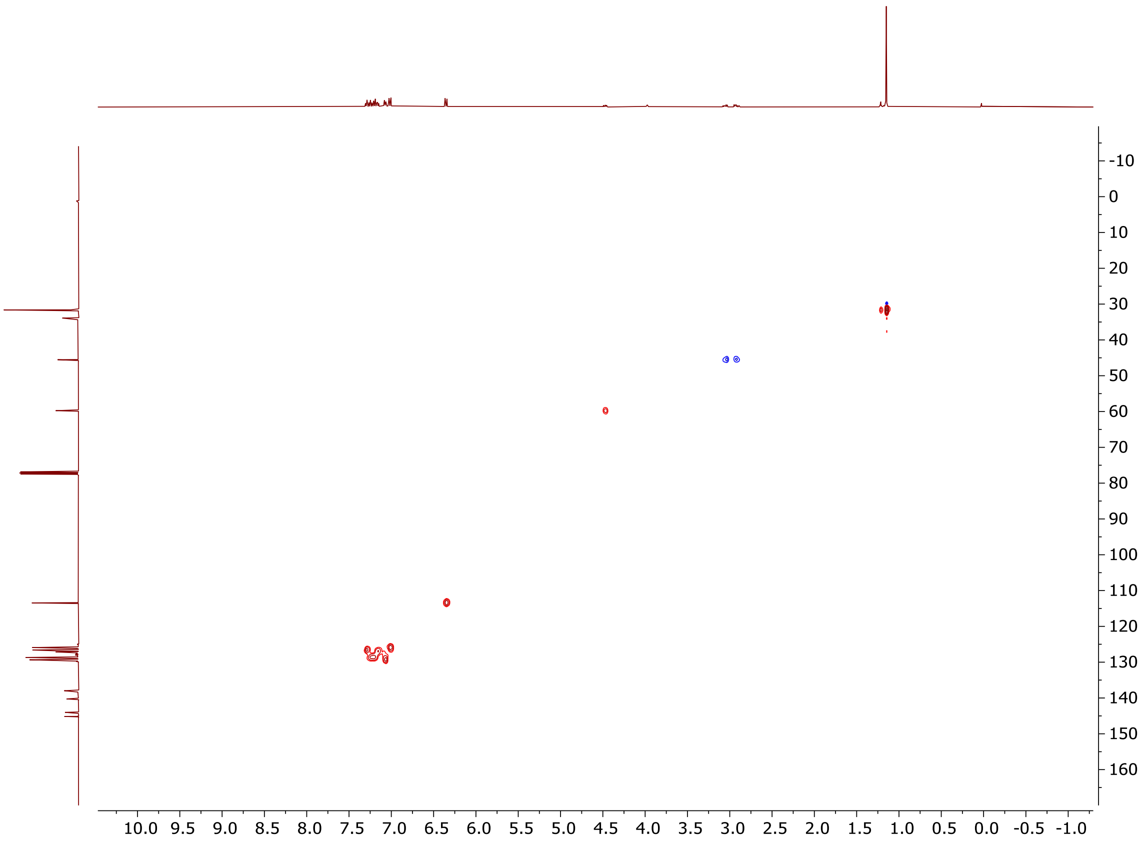

Figure S118: ^{1}*H*-^{13}*C HSQC* NMR Spectrum of 8a in $CDCl_3$ after isolation via column chromatography.

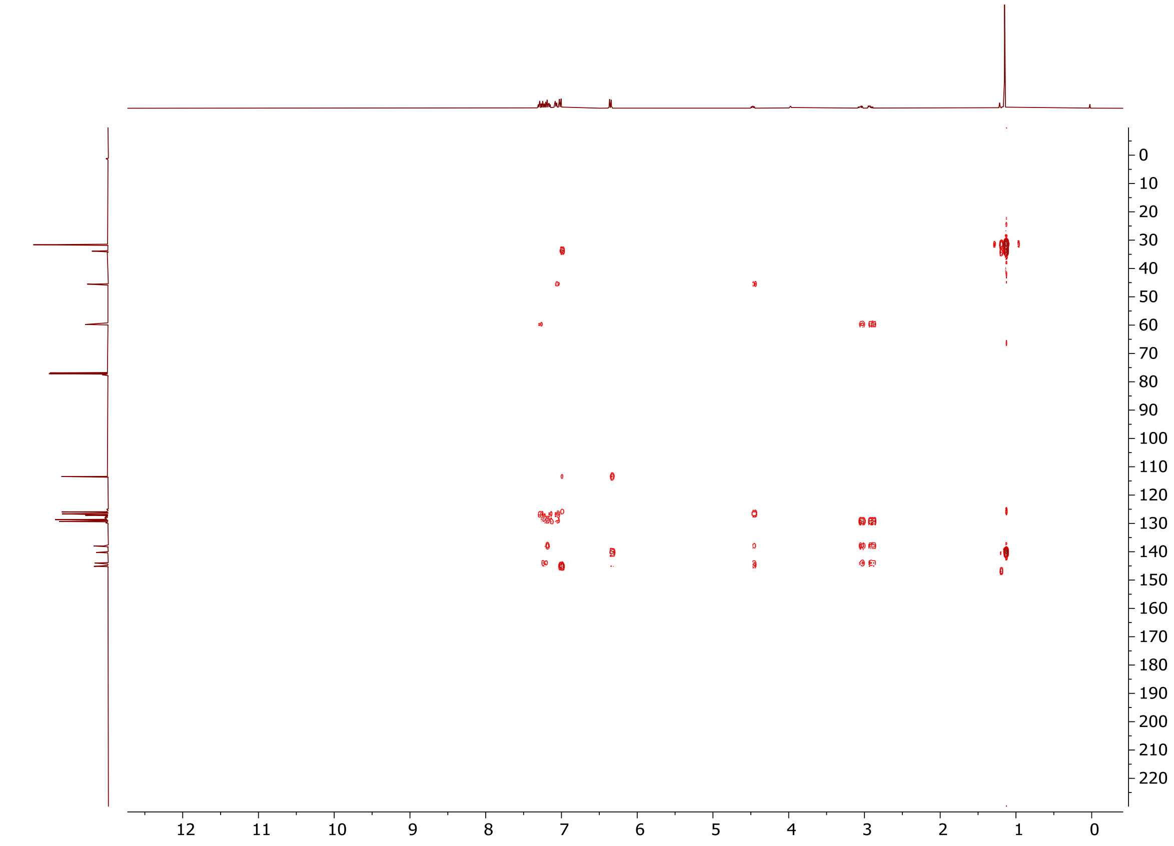

Figure S119: ^{1}H-^{13}C HMBC NMR Spectrum of 8a in $CDCl_3$ after isolation via column chromatography.

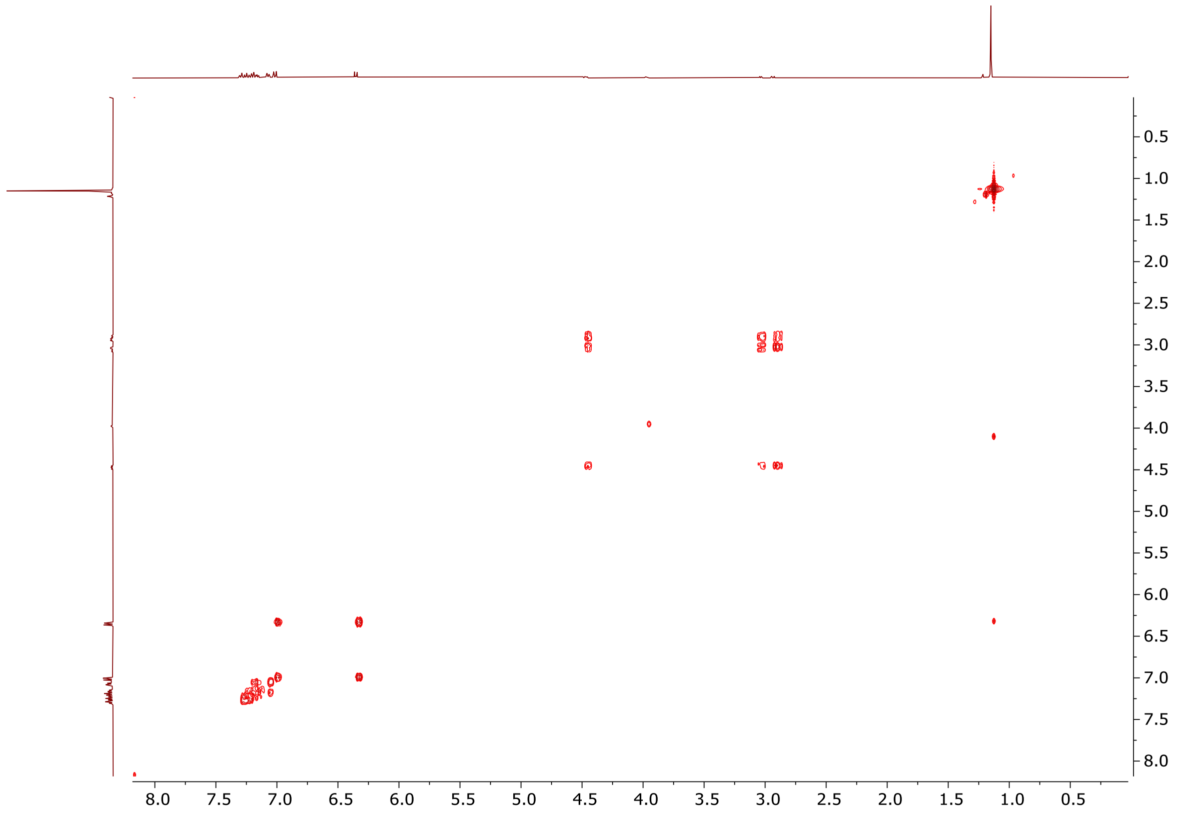

Figure S120: ^{1}H-^{1}H COSY NMR Spectrum of 8a in $CDCl_3$ after isolation via column chromatography.

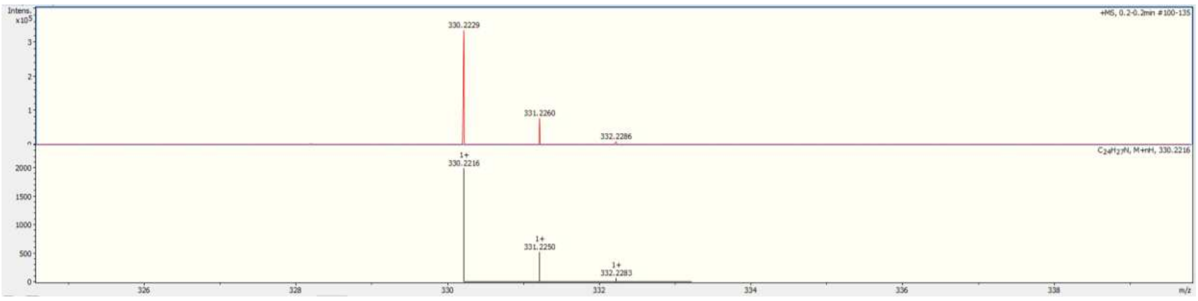

Figure S121: HRMS spectra for compound 8a.

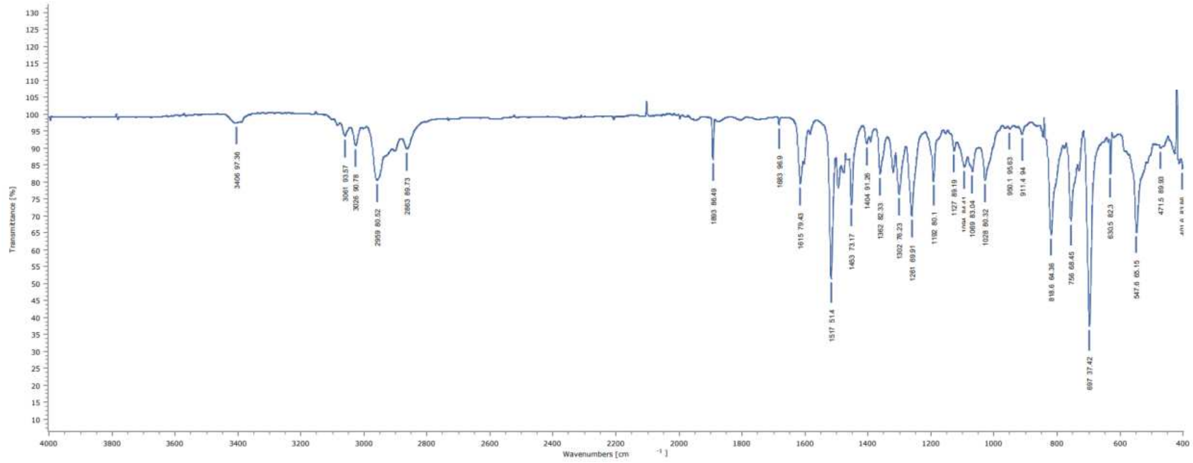

Figure S122: IR spectra for compound 8a.

11.4.2. 4-(tert-butyl)-N-(2-phenylpropan-2-yl)aniline **8b**[14]

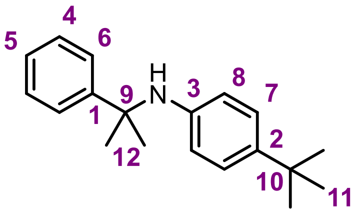

Required an additional zinc reduction step.

White oil (85 mg, 32%)

R_f = 0.28 (30% DCM / 70% hexane)

Analytic data is in accordance with those reported in literature.

^{1}H NMR ($CDCl_3$, 500 MHz) δ 7.60 (d, J = 7.9 Hz, 2H, C^6-H), 7.39 (app. t, J = 7.6 Hz, 2H, C^4-H), 7.29 (app. t, J = 7.3 Hz, 1H, C^5-H), 7.09 (d, J = 8.4 Hz, 2H, C^7-H), 6.35 (d, J = 8.4 Hz, 2H, C^8-H), 3.98 (*br* s, 1H, NH), 1.69 (s, 6H, C^{12}-H), 1.29 (s, 9H, C^{11}-H).

^{13}C{^{1}H} NMR ($CDCl_3$, 126 MHz) δ 147.9 (C^1), 143.8 (C^3), 139.8 (C^2), 128.6 (C^4), 126.4 (C^5), 125.8 (C^6), 125.6 (C^7), 115.2 (C^8), 55.9 (C^9), 33.9 (C^{10}), 31.6 (C^{11}), 30.9 (C^{12}).

HRMS (ESI+): calcd for $[M, C_{19}H_{25}N]^+$ 268.2060, found 268.2063.

IR (Neat): 3411, 2960, 1893, 1617, 1517, 814, 697 cm^{-1}.

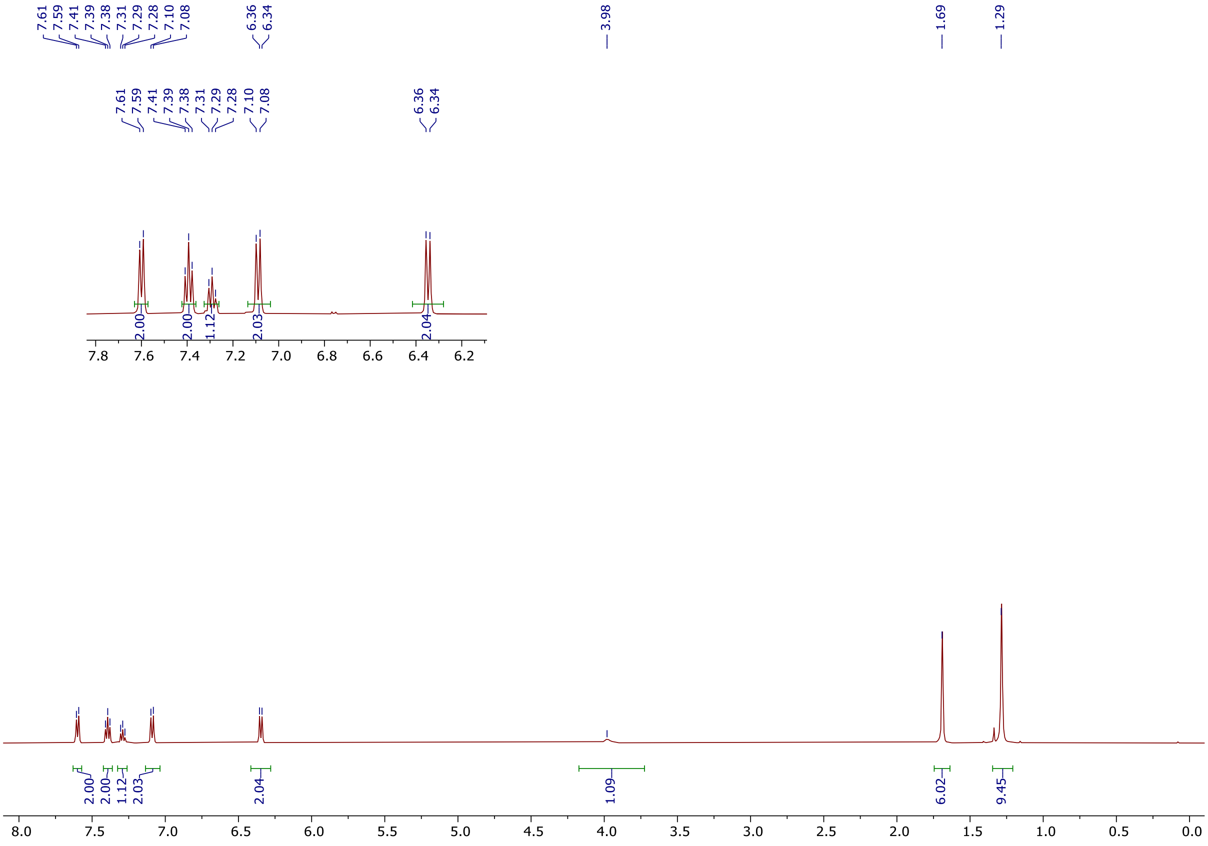

Figure S123: ^{1}H NMR Spectrum of 8b in $CDCl_3$ after isolation via column chromatography.

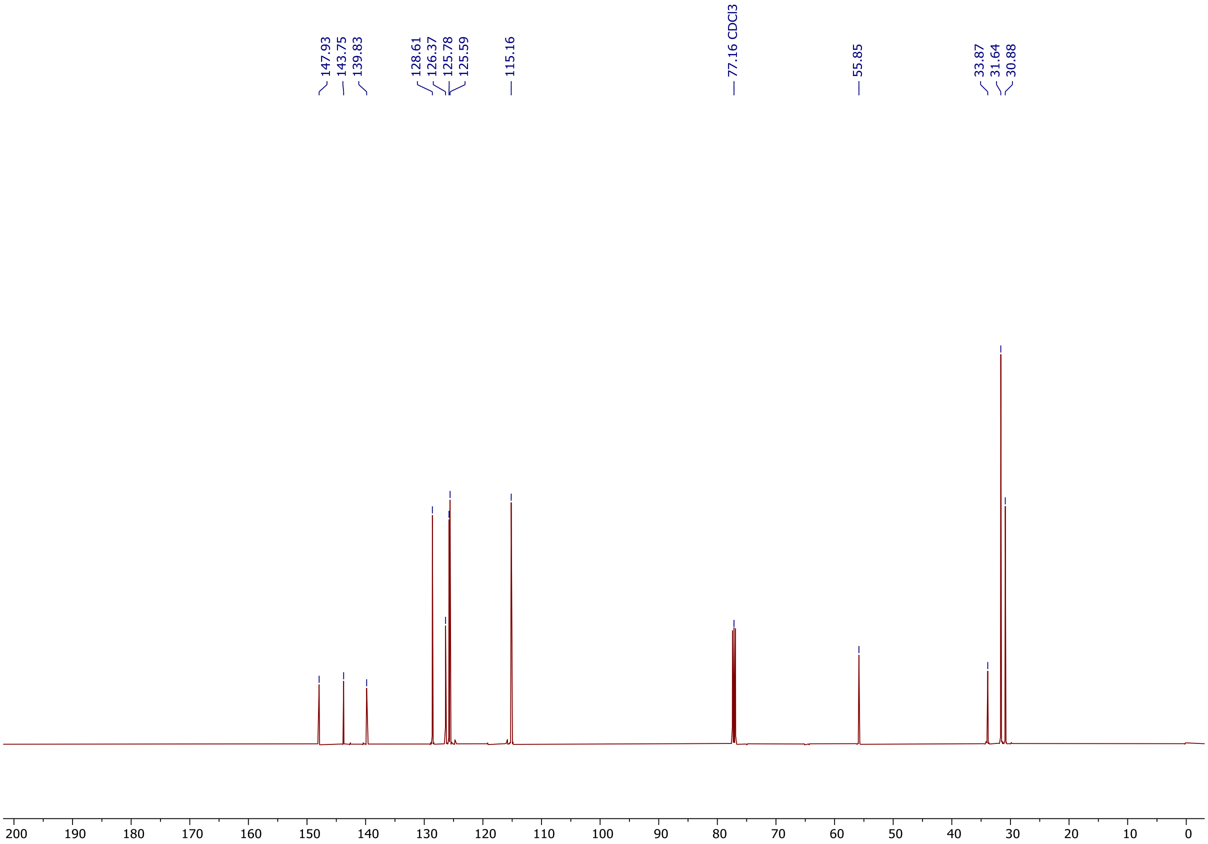

Figure S124: ^{13}C NMR Spectrum of 8b in $CDCl_3$ after isolation via column chromatography.

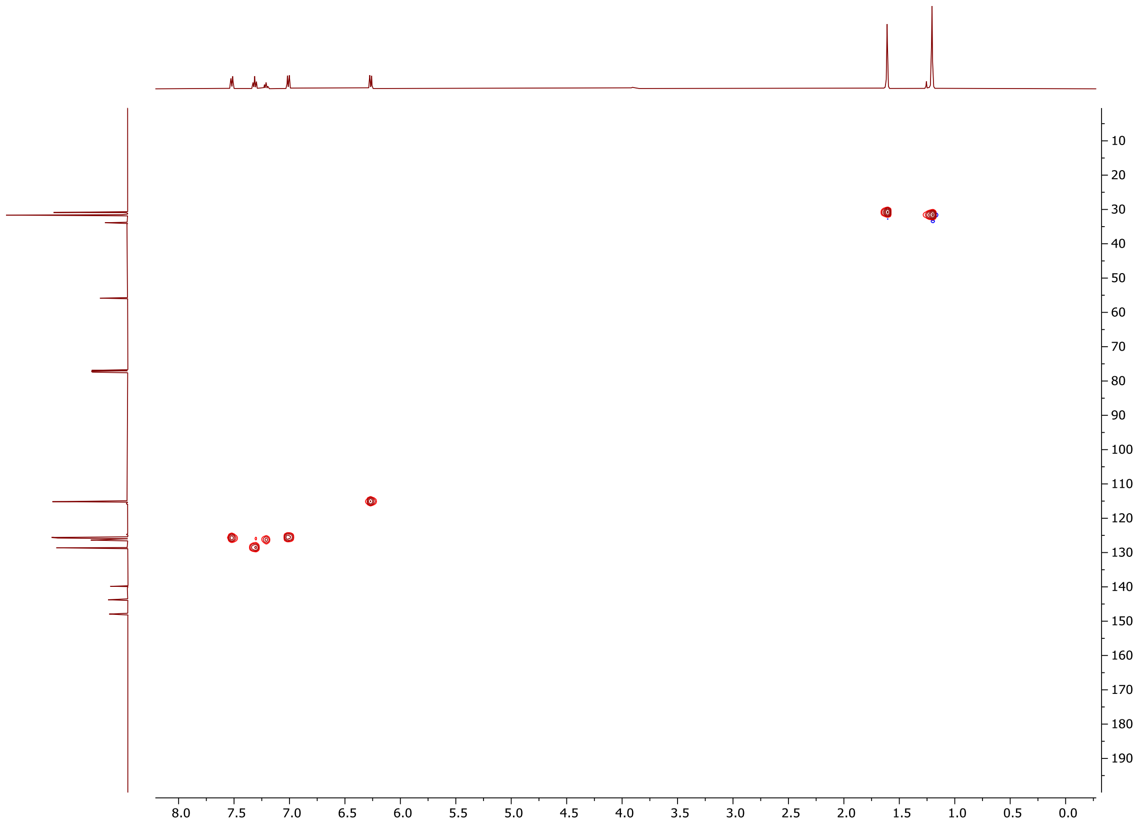

Figure S125: ^{1}H-^{13}C HSQC NMR Spectrum of 8b in $CDCl_3$ after isolation via column chromatography.

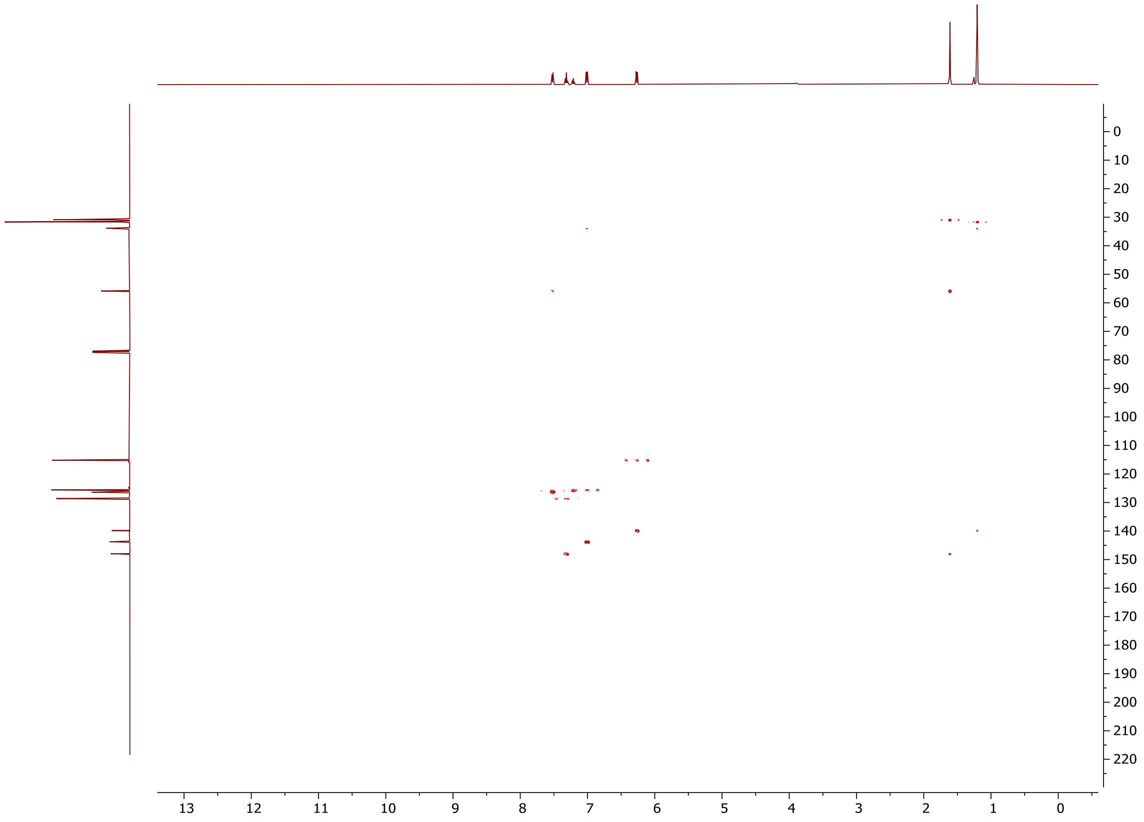

Figure S126: ^{1}H-^{13}C HMBC NMR Spectrum of 8b in $CDCl_3$ after isolation via column chromatography.

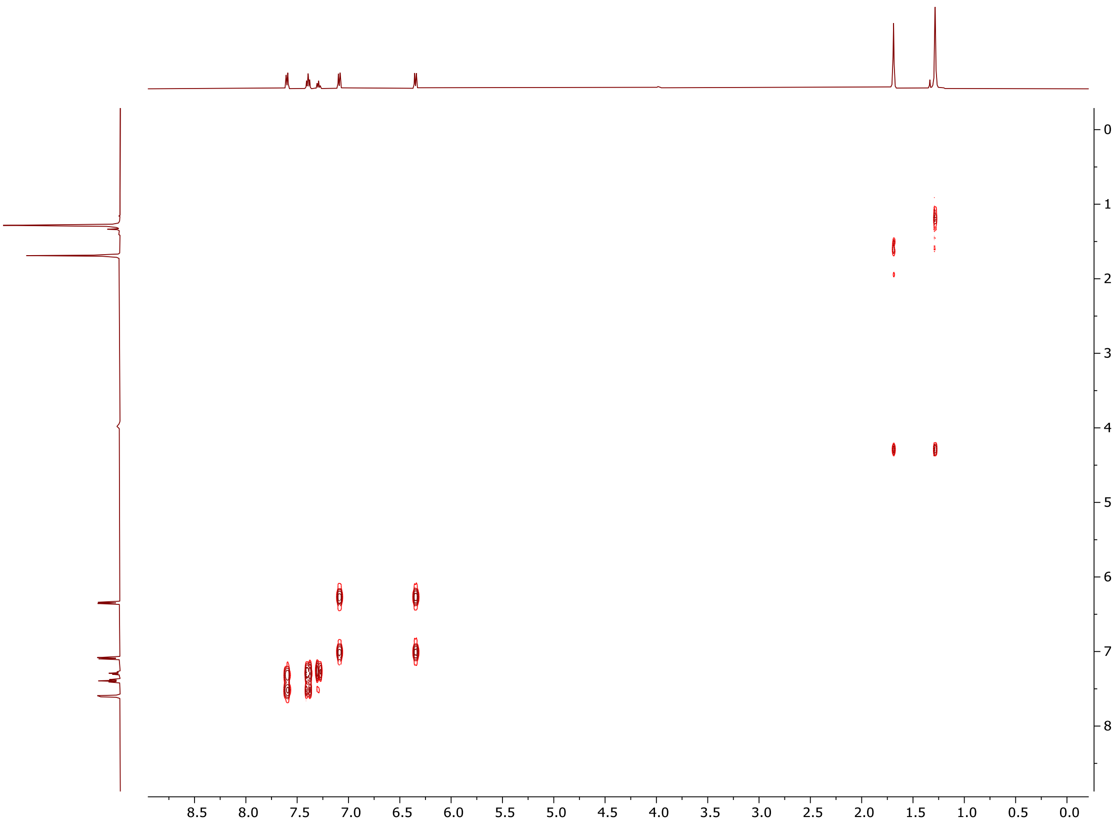

Figure S127: ^1H-^1H COSY NMR Spectrum of 8b in $CDCl_3$ after isolation via column chromatography.

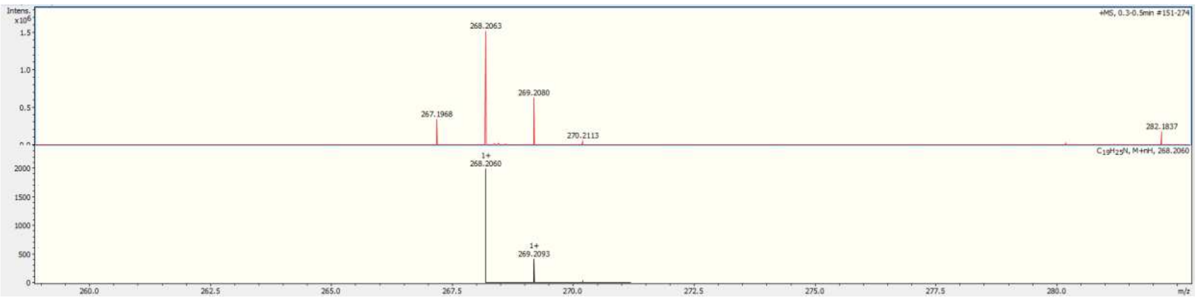

Figure S128: HRMS spectra for compound 8b.

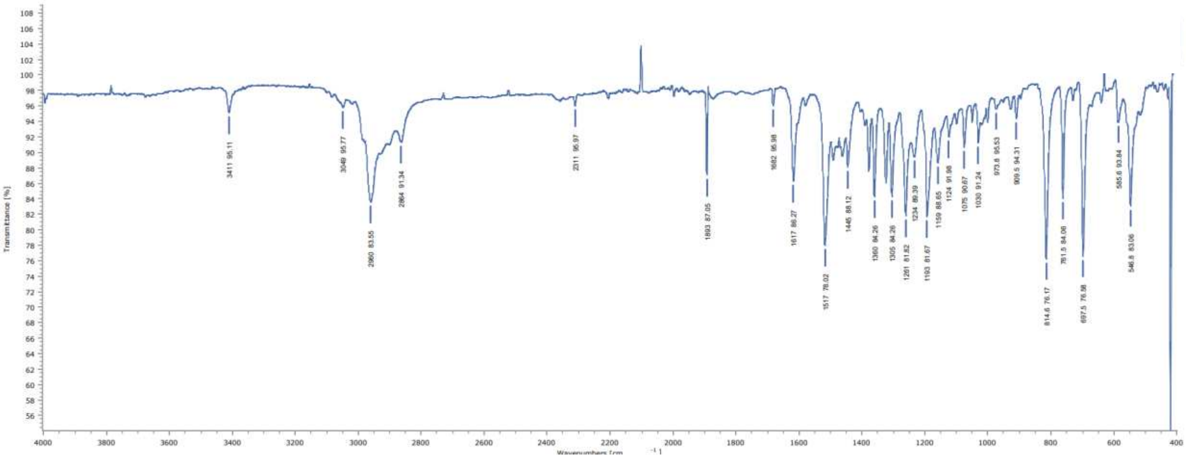

Figure S129: IR spectra for compound 8b.

11.4.3. *Rac* N-(4-(tert-butyl)phenyl)-1,2,3,4-tetrahydronaphthalen-1-amine **8c**[15]

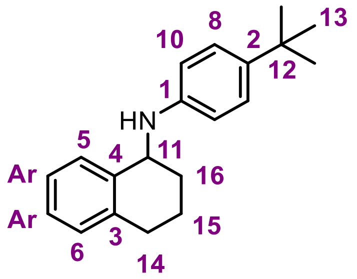

Required an additional zinc reduction step.

Colourless oil (210 mg, 75%)

R_f = 0.23 (30% DCM / 70% hexane)

Analytic data is in accordance with those reported in literature.

^{1}H NMR ($CDCl_3$, 500 MHz) δ 7.46 (dd, J = 6.8, 1.8 Hz, 1H, C^5-H), 7.29 (app. d, J = 8.7 Hz, 2H, C^8-H), 7.27 – 7.20 (m, 2H, Ar), 7.17 (d, J = 7.5 Hz, 1H, C^6-H), 6.69 (app. d, J = 8.7 Hz, 2H, C^{10}-H), 4.66 (app. t, J = 4.8 Hz, 1H, C^{11}-H), 3.84 (*br* s, 1H, NH), 2.89 (app. dt, J = 16.6, 5.4 Hz, 1H, C^{14}-H), 2.84 – 2.78 (m, 1H, C^{14}-H), 2.08 – 2.01 (m, 2H, C^{15}-H), 1.99 – 1.91 (m, 1H, C^{16}-H), 1.84 (app. ddd, J = 13.4, 9.7, 4.8 Hz, 1H, C^{16}-H), 1.36 (s, 9H, C^{13}-H).

^{13}C{^{1}H} NMR ($CDCl_3$, 126 MHz) δ 145.2 (C^1), 139.9 (C^2), 138.5 (C^3), 137.7 (C^4), 129.5 (C^5), 129.1 (C^6), 127.2 (Ar), 126.3 (C^8), 126.2 (Ar), 112.6 (C^{10}), 51.3 (C^{11}), 34.0 (C^{12}), 31.7 (C^{13}), 29.5 (C^{14}), 28.9 (C^{15}), 19.5 (C^{16}).

HRMS (ESI+): calcd for [M, $C_{20}H_{25}N$]$^+$ 280.2060, found 280.2076.

IR (Neat): 3411, 2959, 1615, 1516, 908, 816, 729 cm^{-1}.

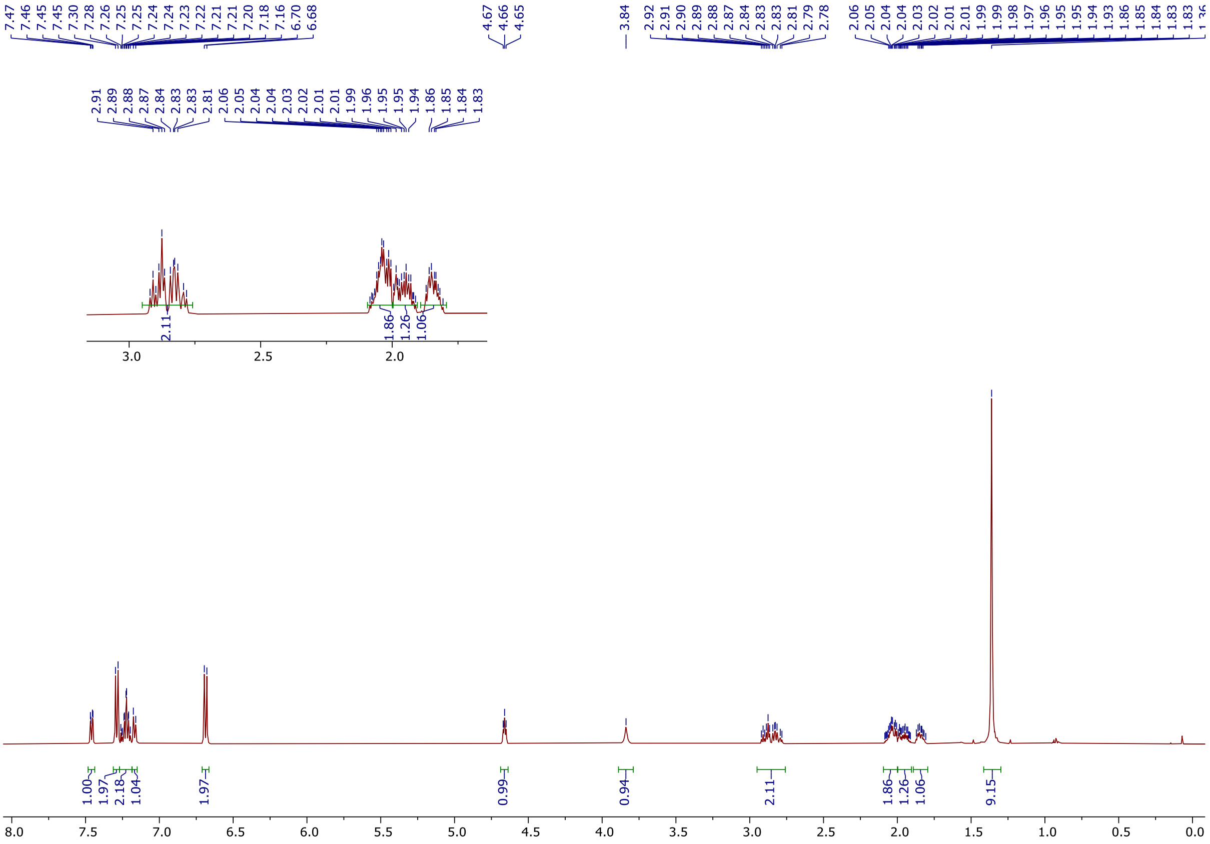

Figure S130: ^{1}H NMR Spectrum of 8c in $CDCl_3$ after isolation via column chromatography.

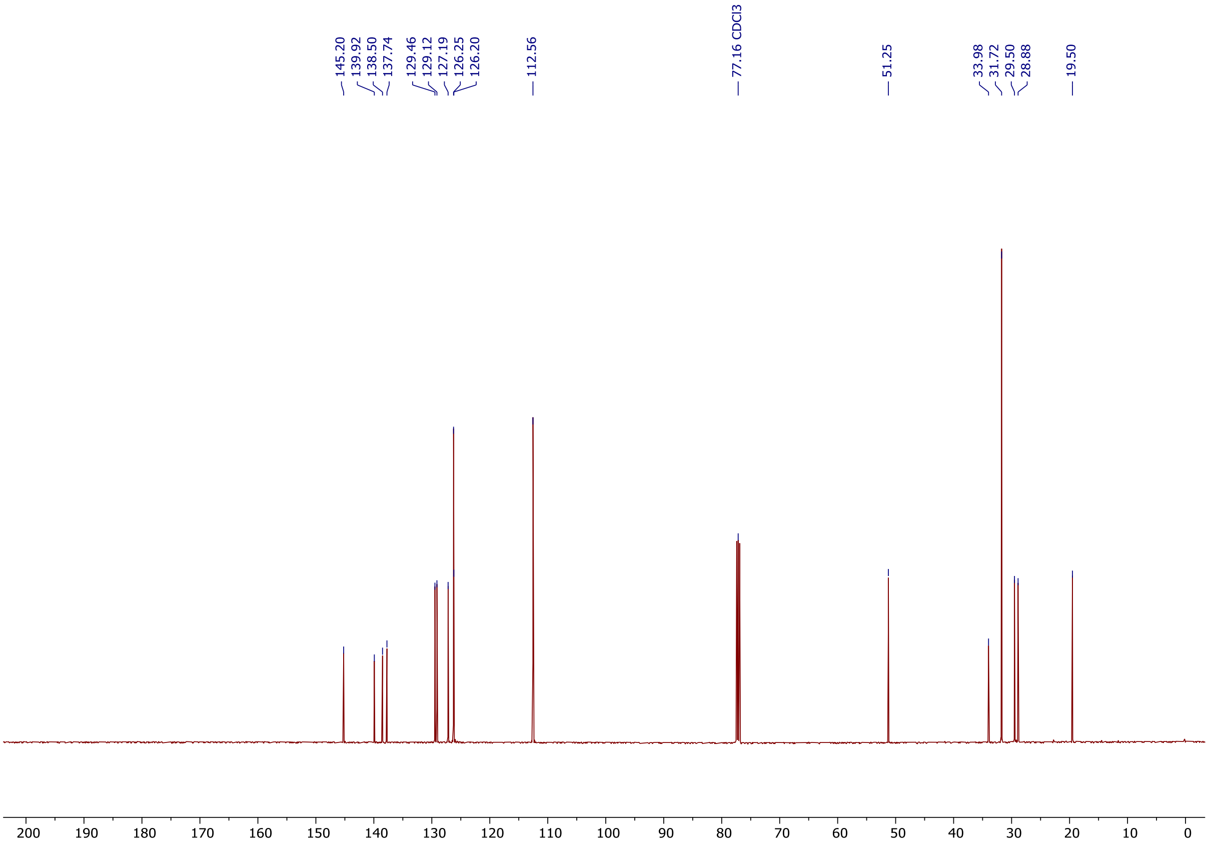

Figure S131: ^{13}C NMR Spectrum of 8c in $CDCl_3$ after isolation via column chromatography.

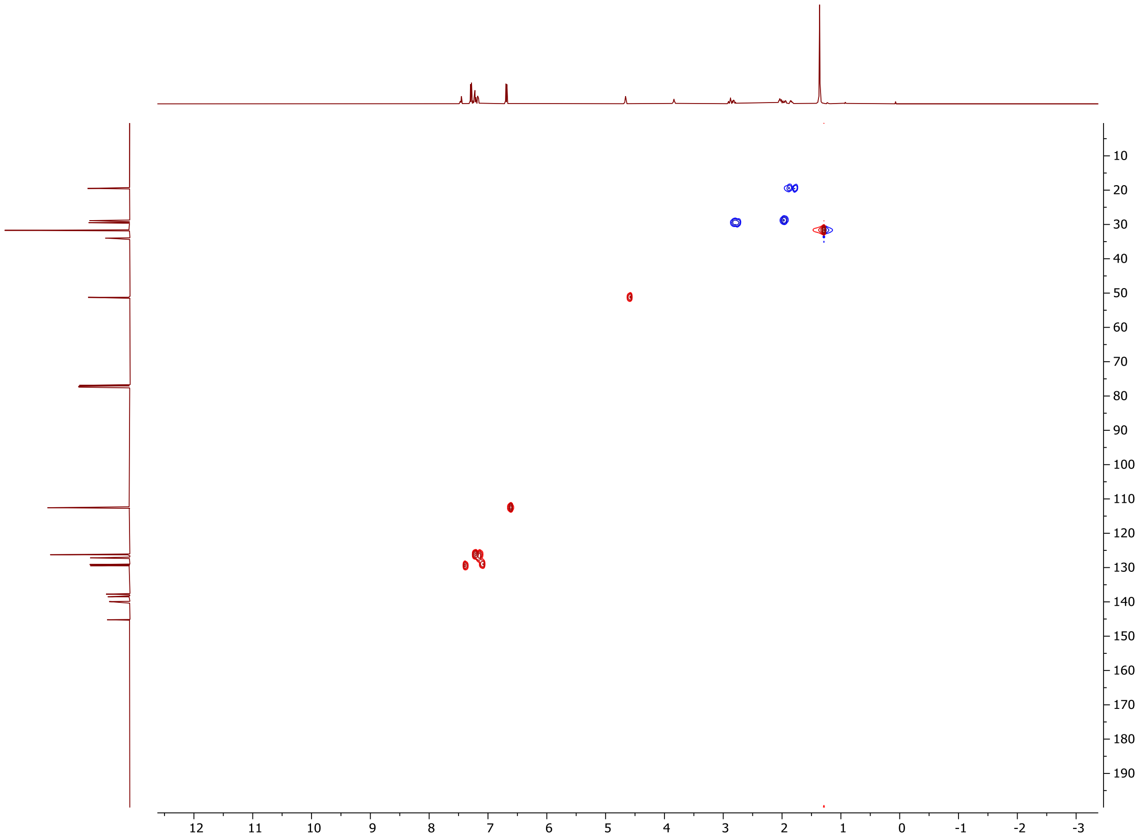

Figure S132: ^{1}H-^{13}C HSQC NMR Spectrum of 8c in $CDCl_3$ after isolation via column chromatography.

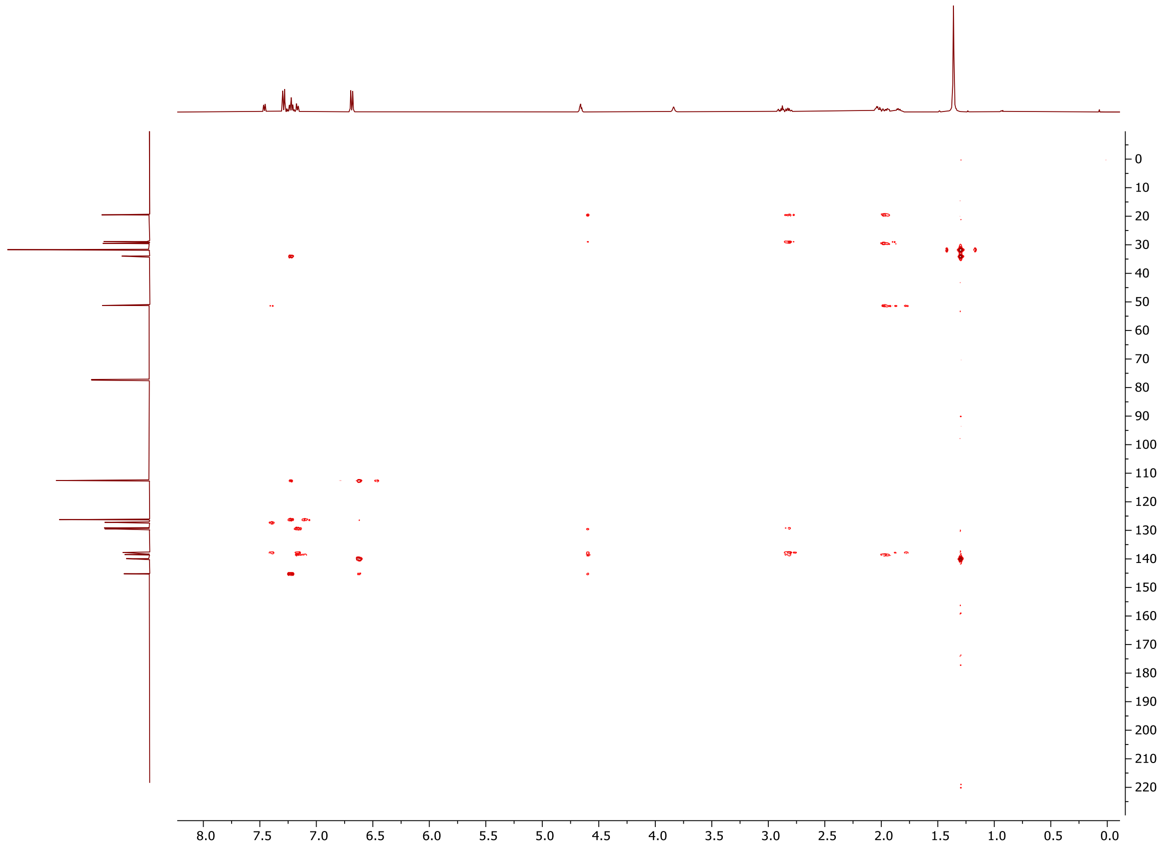

Figure S133: ^{1}H-^{13}C HMBC NMR Spectrum of 8c in $CDCl_3$ after isolation via column chromatography.

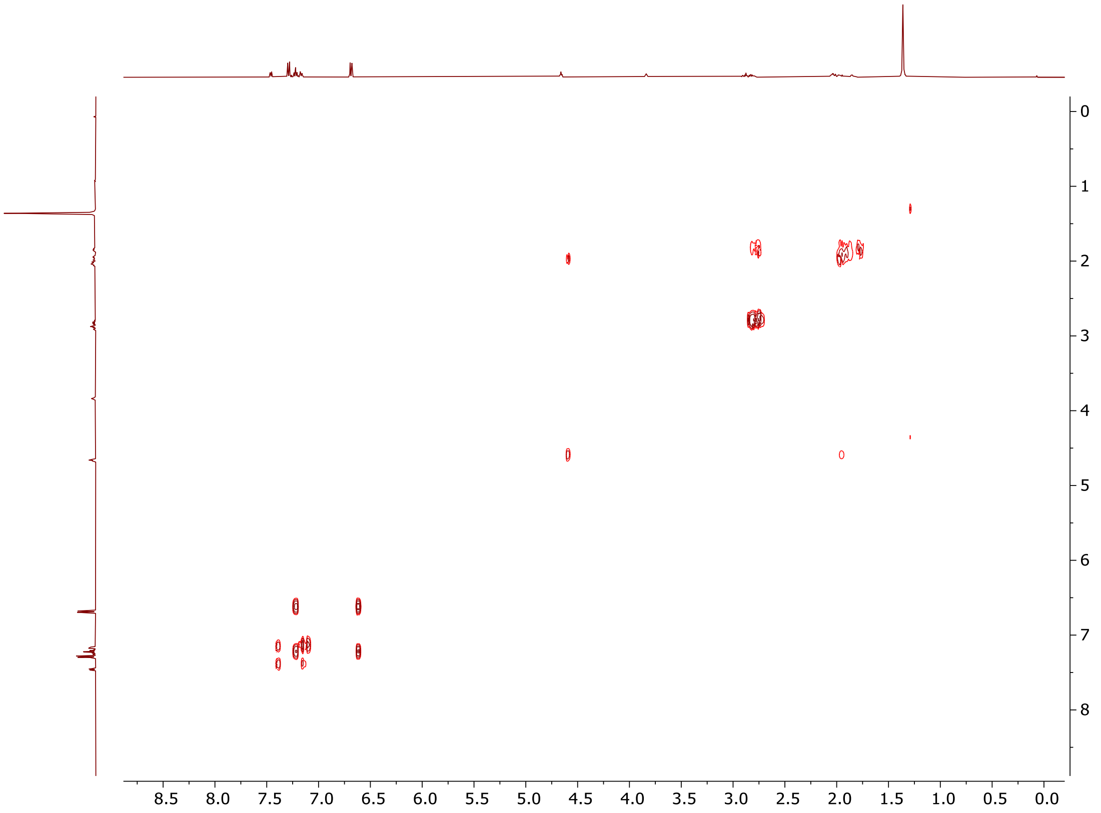

Figure S134: ^{1}H-^{1}H COSY NMR Spectrum of 8c in $CDCl_3$ after isolation via column chromatography.

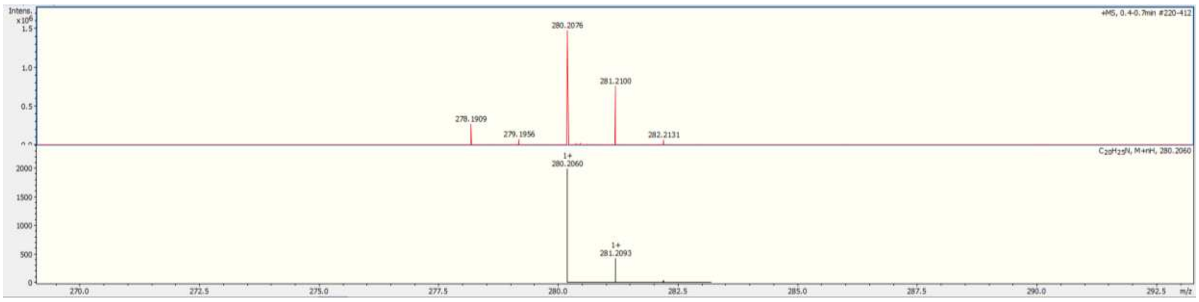

Figure S135: HRMS spectra for compound 8c.

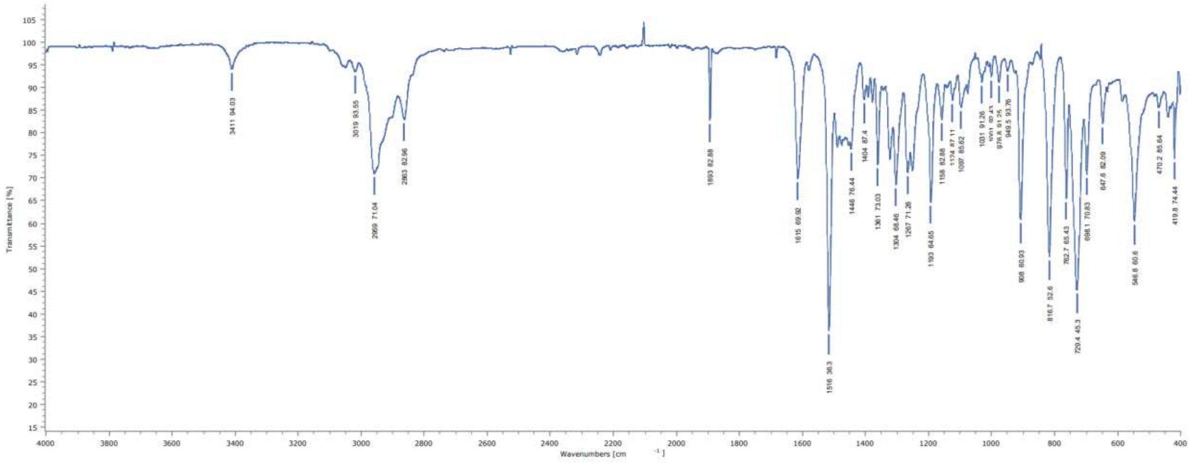

Figure S136: IR spectra for compound 8c.

11.4.4. N-(4-(tert-butyl)phenyl)-2,3-dihydro-1H-inden-1-amine **8d**

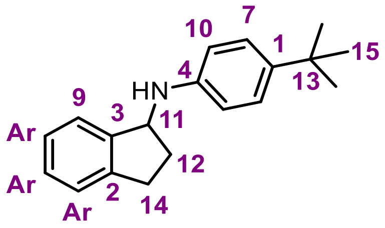

yellow oil (204 mg, 77%)

R_f = 0.54 (30% DCM / 70% hexane)

^{1}H NMR (CDCl$_3$, 500 MHz) δ 7.43 (d, J = 7.2 Hz, 1H, C^9-H), 7.32 – 7.24 (m, 3H, Ar), 7.30 (app. d, J = 8.7 Hz, 2H, C^7-H), 6.72 (app. d, J = 8.7 Hz, 2H, C^{10}-H), 5.04 (app. t, J = 6.8 Hz, 1H, C^{11}-H), 3.85 (*br* s, 1H, NH), 3.06 (ddd, J = 15.9, 8.7, 4.3 Hz, 1H, C^{14}-H), 2.93 (dt, J = 15.9, 7.9 Hz, 1H, C^{14}-H), 2.65 – 2.59 (m, 1H, C^{12}-H), 2.00 – 1.92 (m, 1H, C^{12}-H), 1.37 (s, 9H, C^{15}-H).

^{13}C{^{1}H} NMR (CDCl$_3$, 126 MHz) δ 145.5 (C^4), 144.9 (C^2), 143.7 (C^3), 140.2 (C^1), 127.9 (Ar), 126.7 (Ar), 126.2 (C^7), 124.9 (Ar), 124.4 (C^9), 112.9 (C^{10}), 58.9 (C^{11}), 34.1 (C^{12}), 34.0 (C^{13}), 31.7 (C^{14}), 30.4 (C^{15}).

HRMS (ESI+): calcd for [M, $C_{19}H_{23}N$]$^+$ 266.1903, found 266.1902.

IR (Neat): 3397, 2957, 1614, 1517, 817, 740 cm^{-1}.

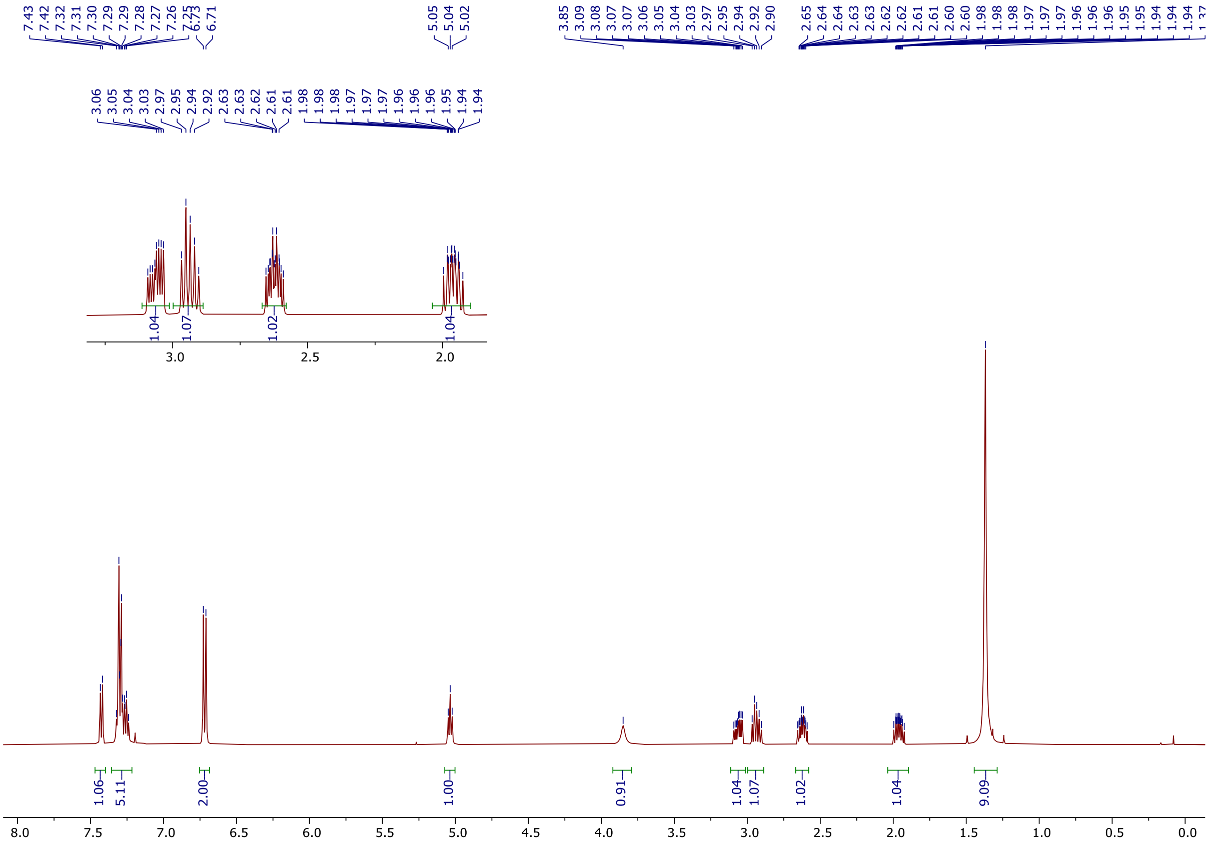

Figure S137: ^{1}H NMR Spectrum of 8d in CDCl$_3$ after isolation via column chromatography.

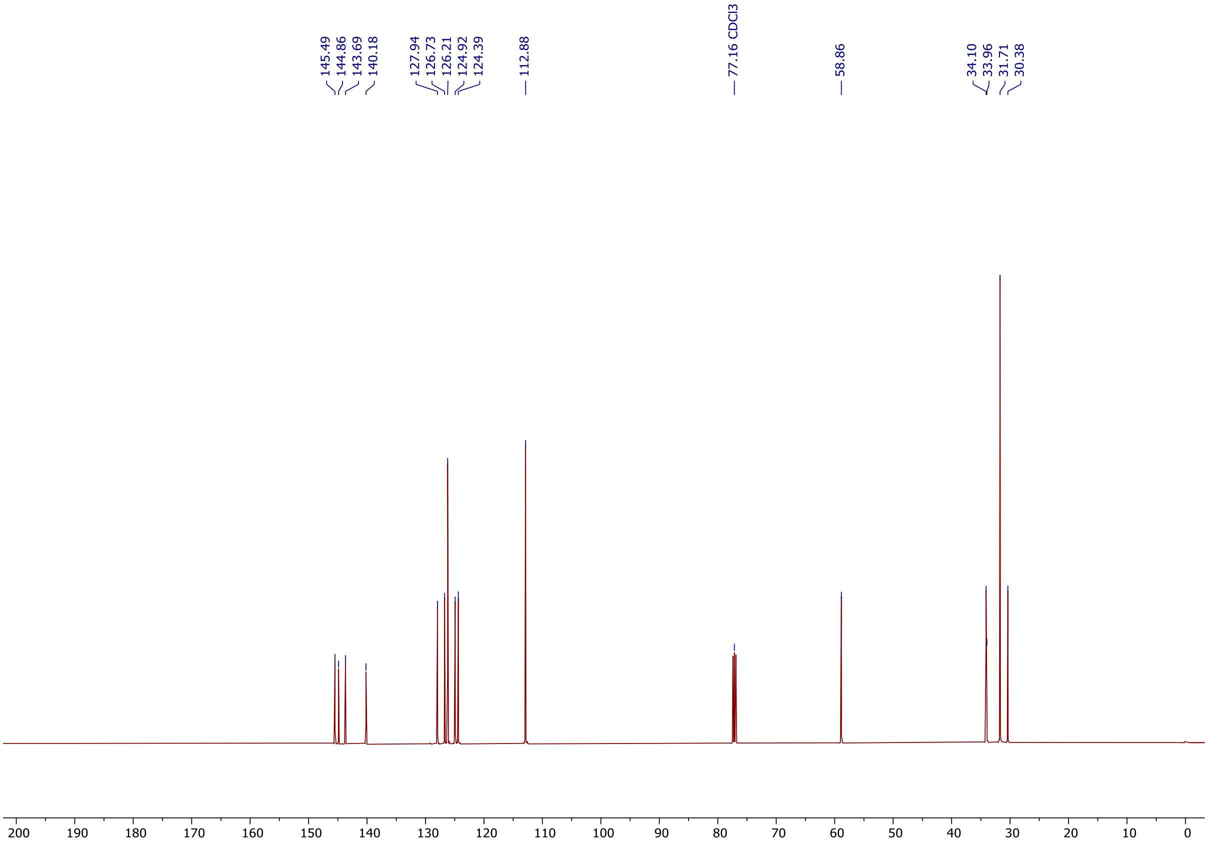

Figure S138: ^{13}C NMR Spectrum of 8d in $CDCl_3$ after isolation via column chromatography.

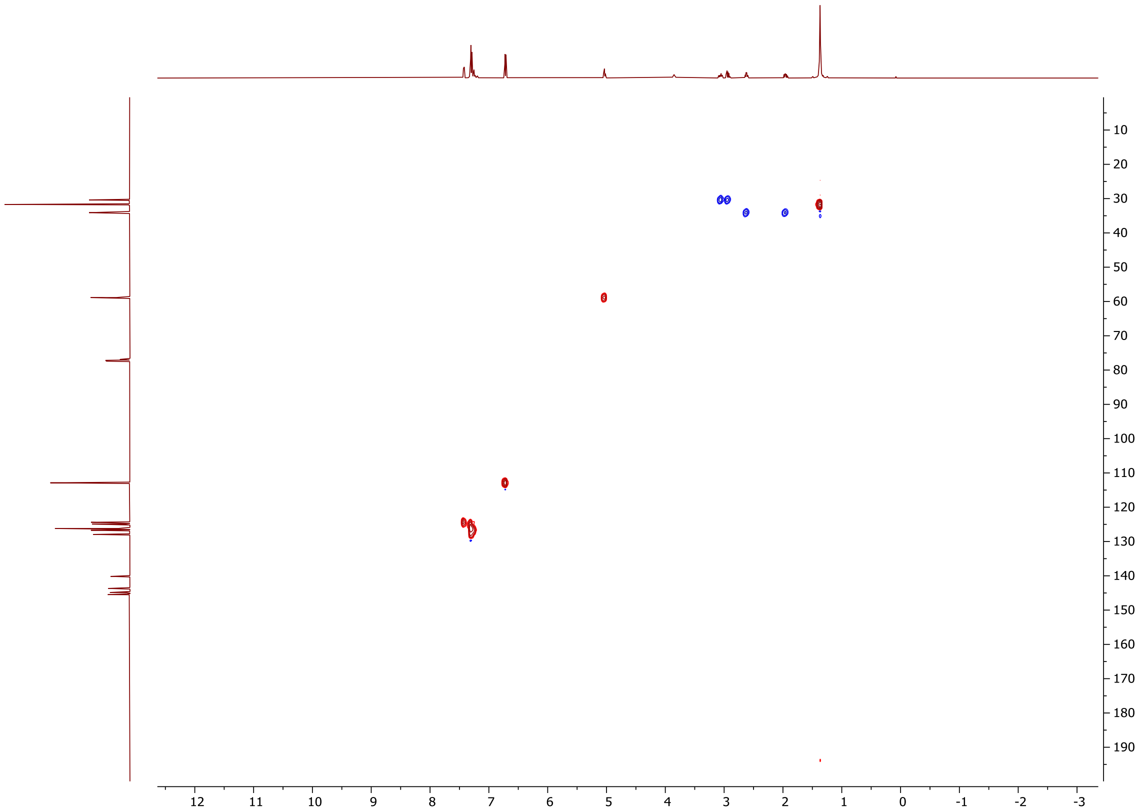

Figure S139: [1]*H*-[13]*C HSQC* NMR Spectrum of 8d in $CDCl_3$ after isolation via column chromatography.

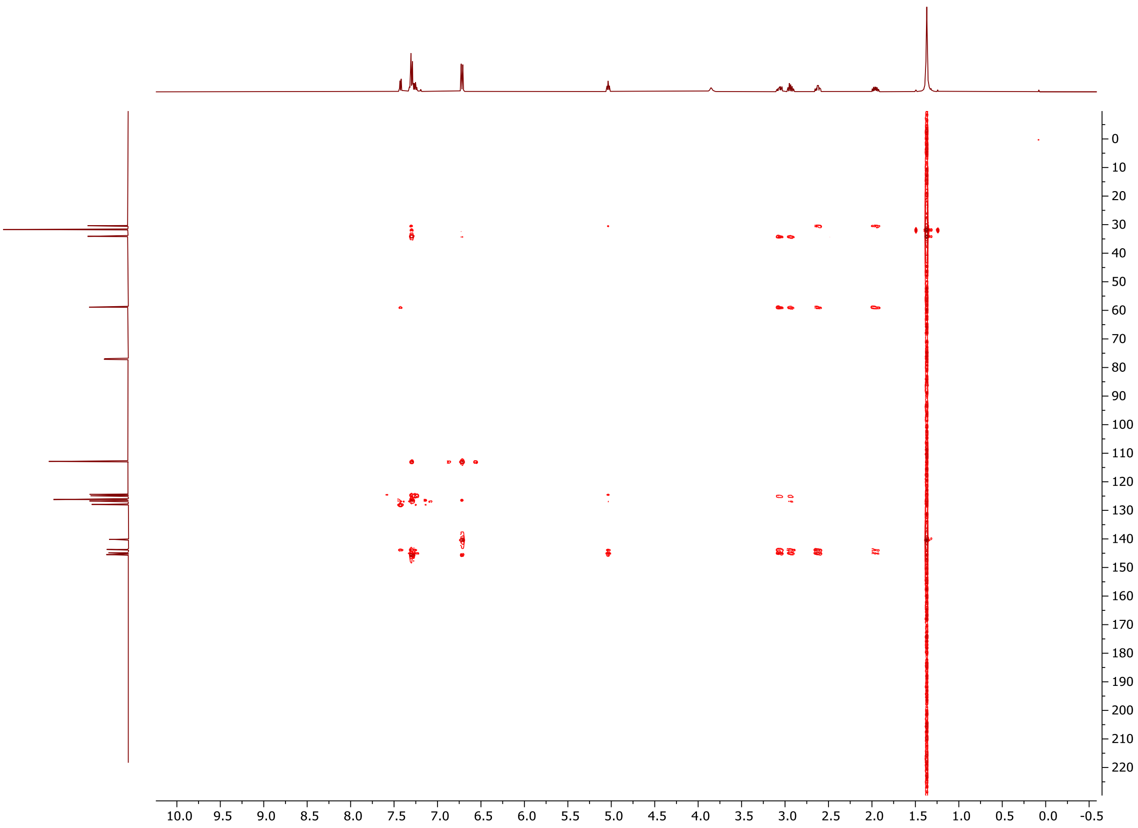

Figure S140: ^{1}H-^{13}C HMBC NMR Spectrum of 8d in $CDCl_3$ after isolation via column chromatography.

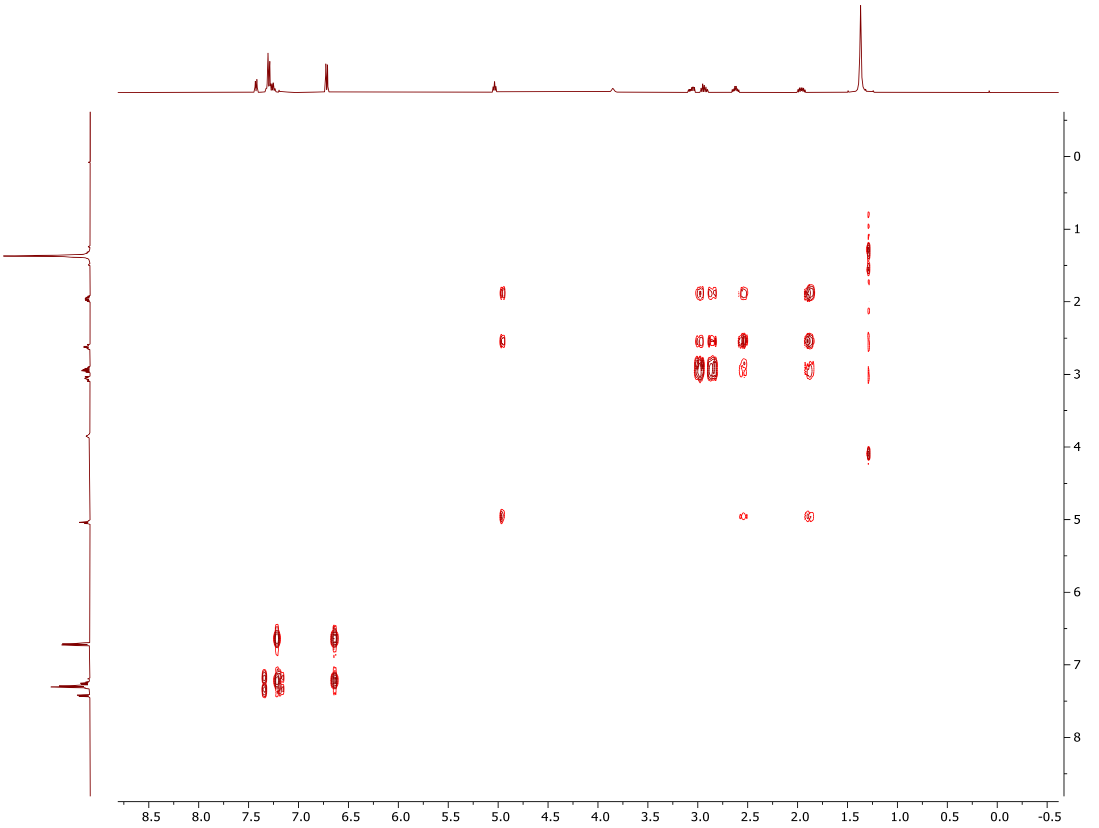

Figure S141: ^{1}H-^{1}H COSY NMR Spectrum of 8d in $CDCl_3$ after isolation via column chromatography.

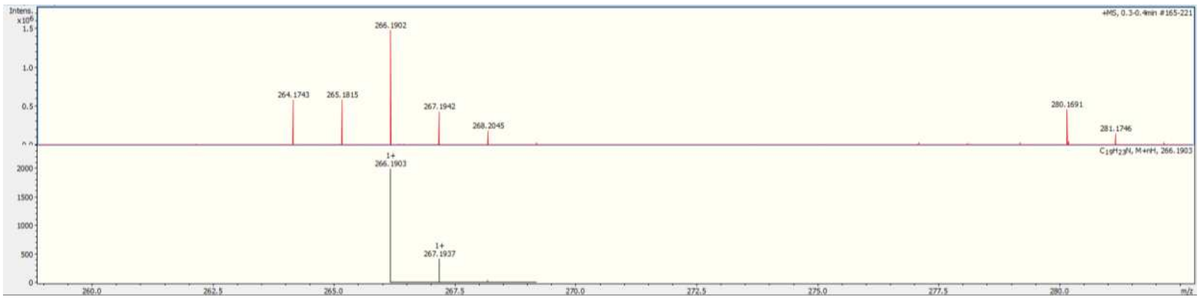

Figure S142: HRMS spectra for compound 8d.

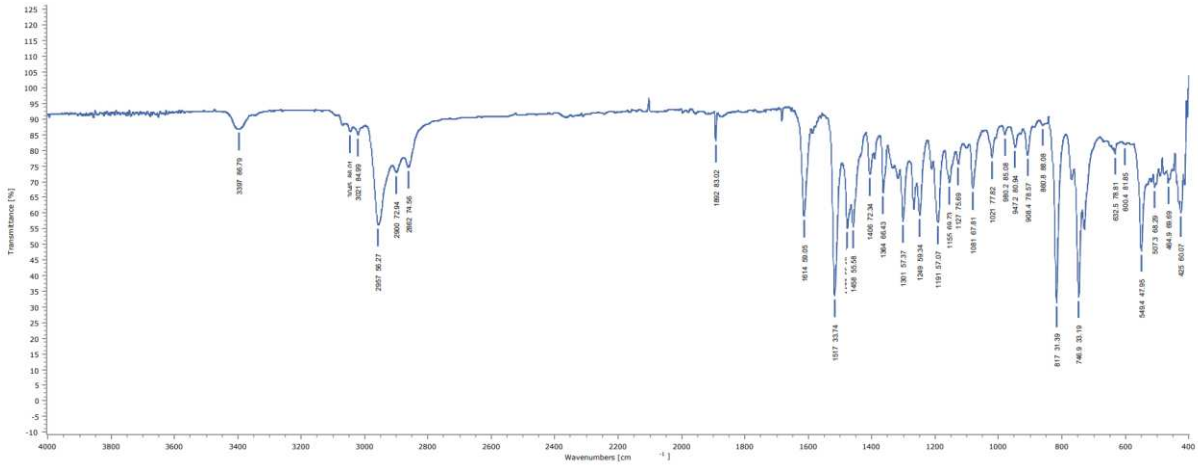

Figure S143: IR spectra for compound 8d.

11.4.5. N-(4-(tert-butyl)phenyl)-4,7-dimethyl-2,3-dihydro-1H-inden-1-amine **8e**

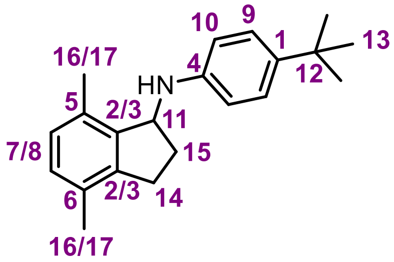

Required Zinc Reduction Step

Colourless oil (209.4 mg, 71%)

R_f = 0.48 (30% DCM / 70% hexane)

^{1}H NMR ($CDCl_3$, 500 MHz) δ 7.30 (d, J = 8.8 Hz, 2H, C^9-H), 7.08 (d, J = 7.6 Hz, 1H, $C^{7/8}$-H), 7.02 (d, J = 7.6 Hz, 1H, $C^{7/8}$-H), 6.67 (d, J = 8.6 Hz, 2H, C^{10}-H), 5.03 (dd, J = 5.2, 3.01 Hz, 1H, C^{11}-H), 3.69 (*br* s, 1H , NH), 3.03 (dt, J = 16.7, 8.5 Hz, 1H, C^{15}-H), 2.88 (ddd, J = 16.3, 7.2, 4.2 Hz, 1H, C^{15}-H), 2.37 (s, 3H, $C^{16/17}$-H), 2.34 – 2.29 (m, 2H, C^{14}-H), 2.32 (s, 3H, $C^{16/17}$-H), 1.37 (s, 9H, C^{13}-H).

^{13}C{^{1}H} NMR ($CDCl_3$, 126 MHz) δ 145.4 (C^1), 143.5 (C^2), 142.2 (C^3), 139.8 (C^4), 132.5 (C^5), 131.6 (C^6), 129.3 (C^7), 128.3 (C^8), 126.2 (C^9), 112.7 (C^{10}), 57.9 (C^{11}), 34.0 (C^{12}), 31.7 (C^{13}), 31.4 (C^{14}), 29.5 (C^{15}), 18.9 (C^{16}), 18.3(C^{17}).

HRMS (ESI+): calcd for [M, $C_{21}H_{27}N$]$^+$ 294.2216, found 294.2220.

IR (Neat): 3398, 2959, 1612, 1516, 816 cm^{-1}

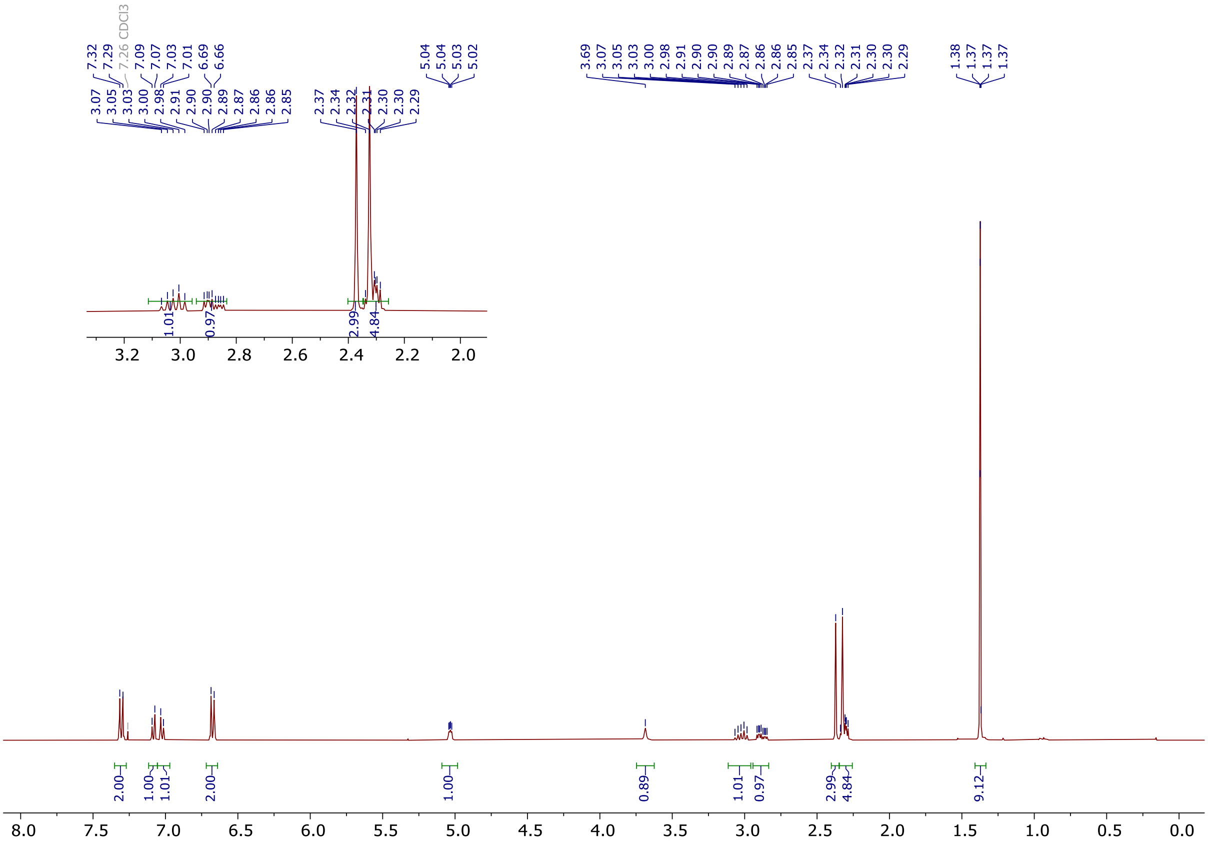

Figure S144: ^{1}H NMR Spectrum of 8e in $CDCl_3$ after isolation via column chromatography.

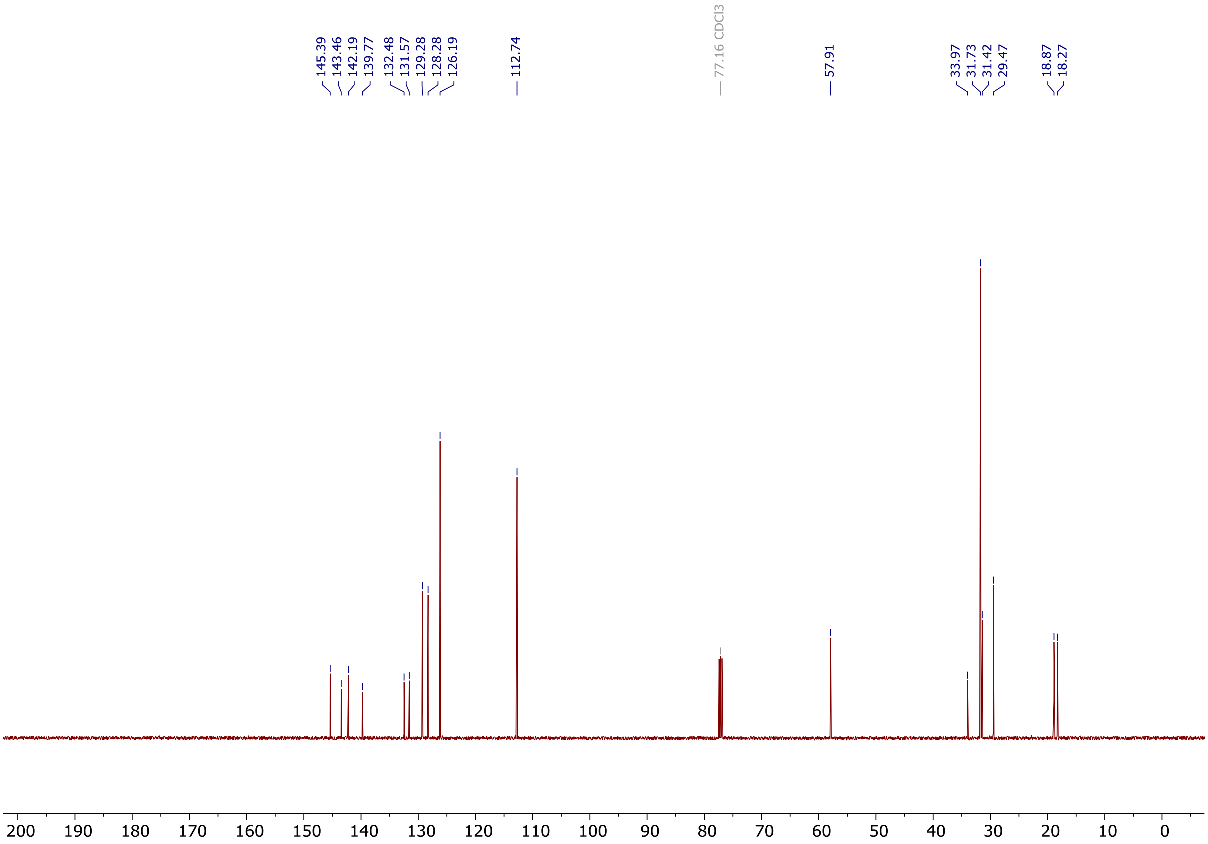

Figure S145: ^{13}C NMR Spectrum of 8e in $CDCl_3$ after isolation via column chromatography.

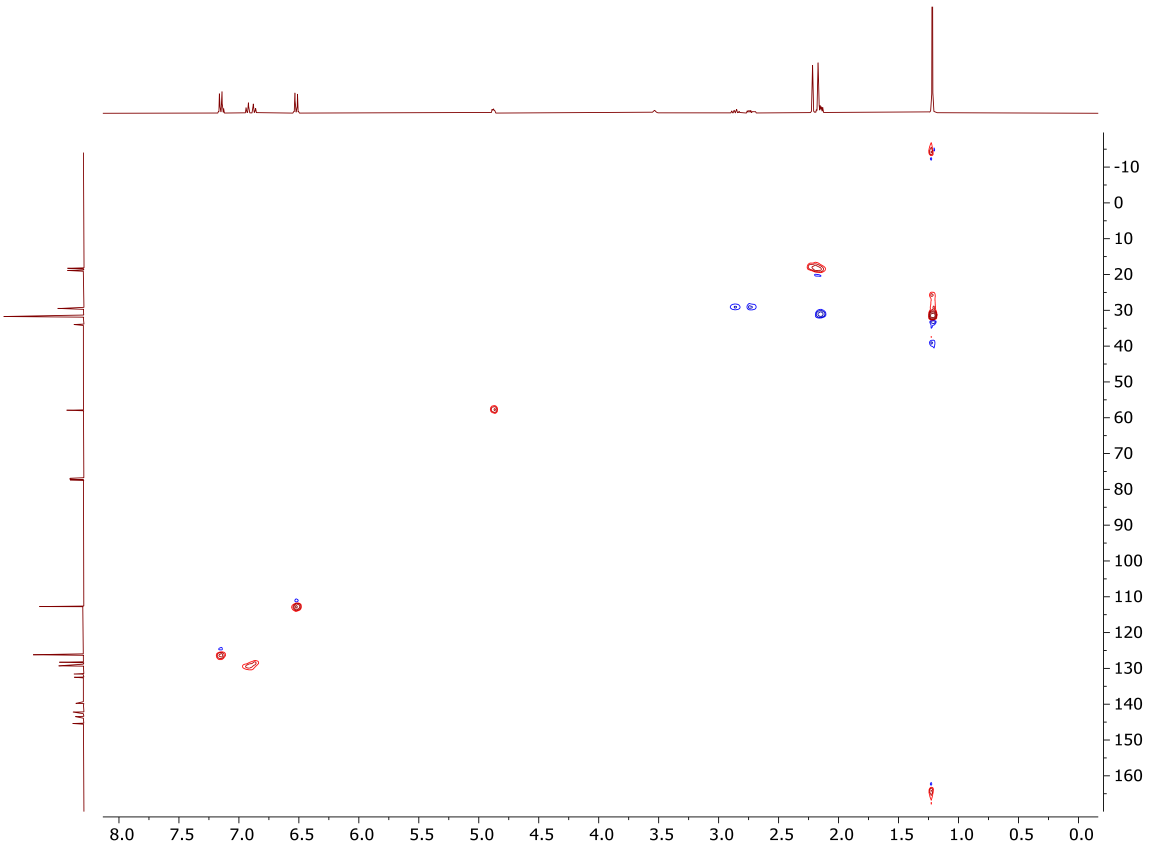

Figure S146: ^{1}H-^{13}C HSQC NMR Spectrum of 8e in $CDCl_3$ after isolation via column chromatography.

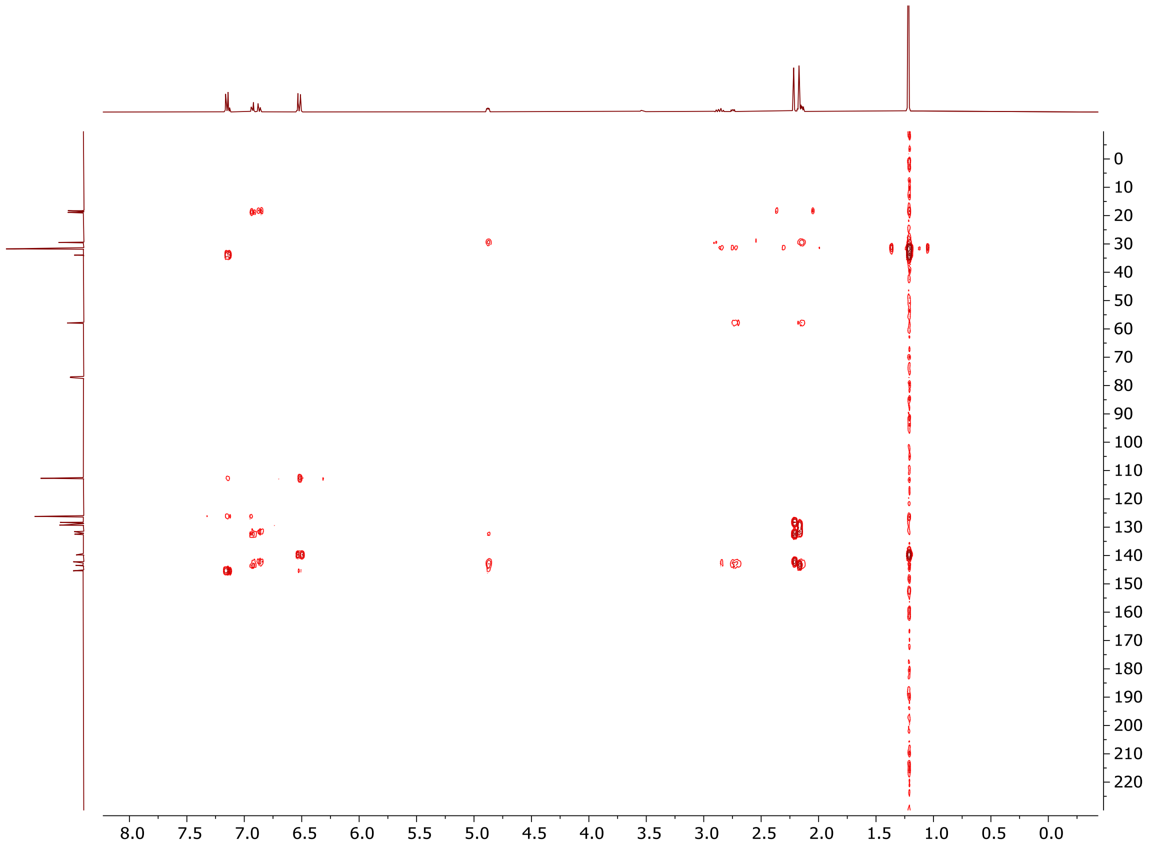

Figure S147: ^{1}H-^{13}C HMBC NMR Spectrum of 8e in $CDCl_3$ after isolation via column chromatography.

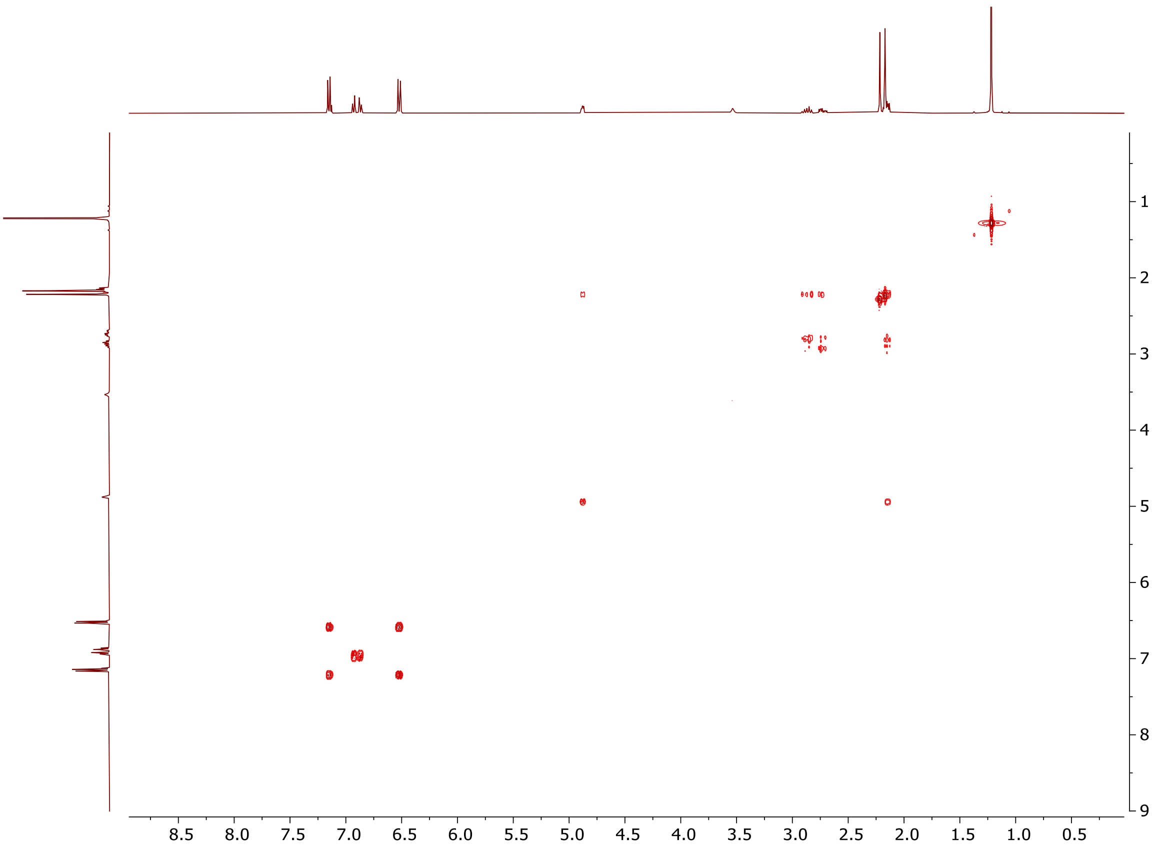

Figure S148: ^{1}H-^{1}H COSY NMR Spectrum of 8e in $CDCl_3$ after isolation via column chromatography.

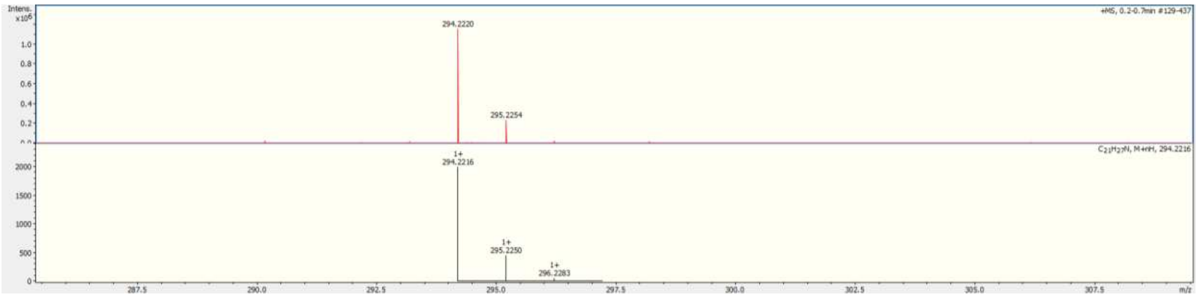

Figure S149: HRMS spectra for compound 8e.

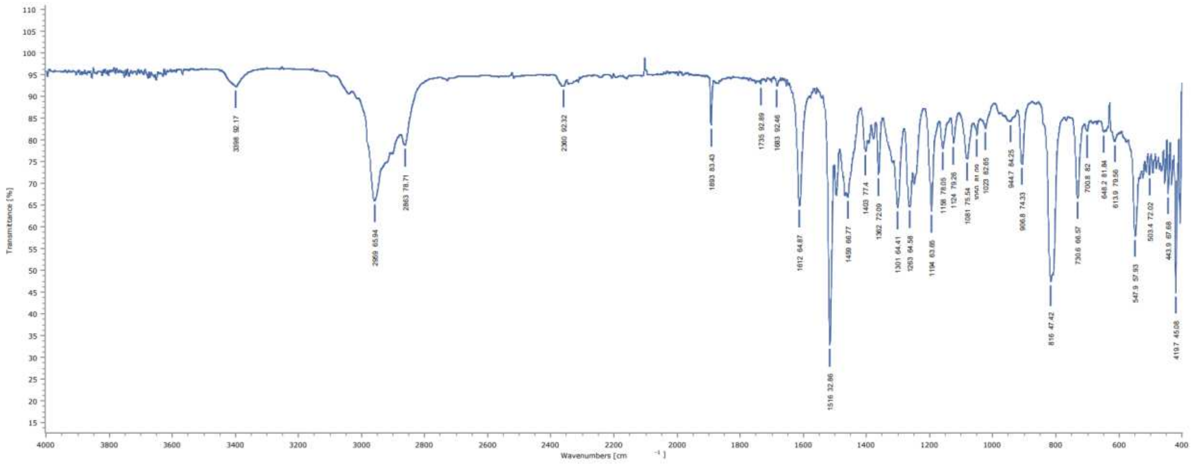

Figure S150: IR spectra for compound 8e.

11.4.6. N-(4-(tert-butyl)phenyl)-2,3-dihydrobenzofuran-3-amine **8f**[16]

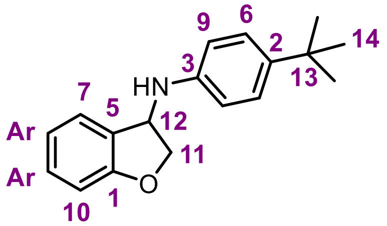

Pale yellow oil (164 mg, 61%)

R_f = 0.26 (30% DCM / 70% hexane)

Analytic data is in accordance with those reported in literature.

^{1}H NMR ($CDCl_3$, 500 MHz) δ 7.39 (d, J = 7.4 Hz, 1H, C^7-H), 7.31 – 7.26 (m, 1H, Ar-H), 7.30 (d, J = 8.6 Hz, 2H, C^6-H), 6.96 (app. t, J = 7.4 Hz, 1H, Ar-H), 6.92 (d, J = 8.1 Hz, 1H, C^{10}), 6.62 (d, J = 8.6 Hz, 2H, C^9), 5.23 – 5.19 (m, 1H, C^{12}-H), 4.74 (dd, J = 9.5, 7.3 Hz, 1H, C^{11}-H), 4.44 (dd, J = 9.5, 4.1 Hz, 1H, C^{11}-H), 3.92 (*br* s, 1H, NH), 1.36 (s, 9H, C^{14}-H).

^{13}C{^{1}H} NMR ($CDCl_3$, 126 MHz) δ 160.3 (C^1), 144.2 (C^3), 141.1 (C^2), 130.2 (Ar), 127.7 (C^5), 126.4 (C^6), 125.4 (C^7), 121.0 (Ar), 113.0 (C^9), 110.4 (C^{10}), 77.9 (C^{11}), 55.7 (C^{12}), 34.0 (C^{13}), 31.6 (C^{14}).

HRMS (ESI+): calcd for [M, $C_{18}H_{21}NO$]$^{+Na}$ 290.1515, found 290.1518.

IR (Neat): 3387, 2949, 1611, 1516, 958, 822, 749 cm^{-1}.

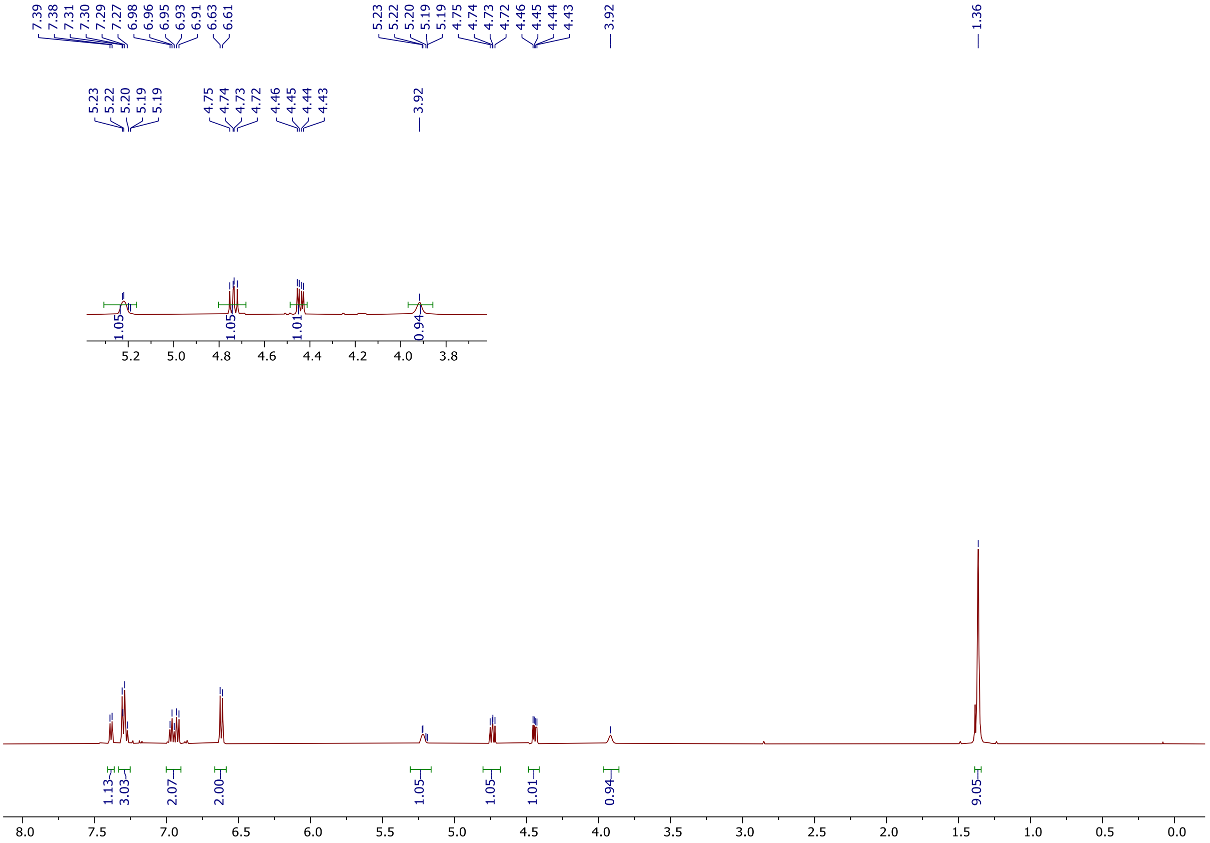

Figure S151: ^{1}H NMR Spectrum of 8f in $CDCl_3$ after isolation via column chromatography.

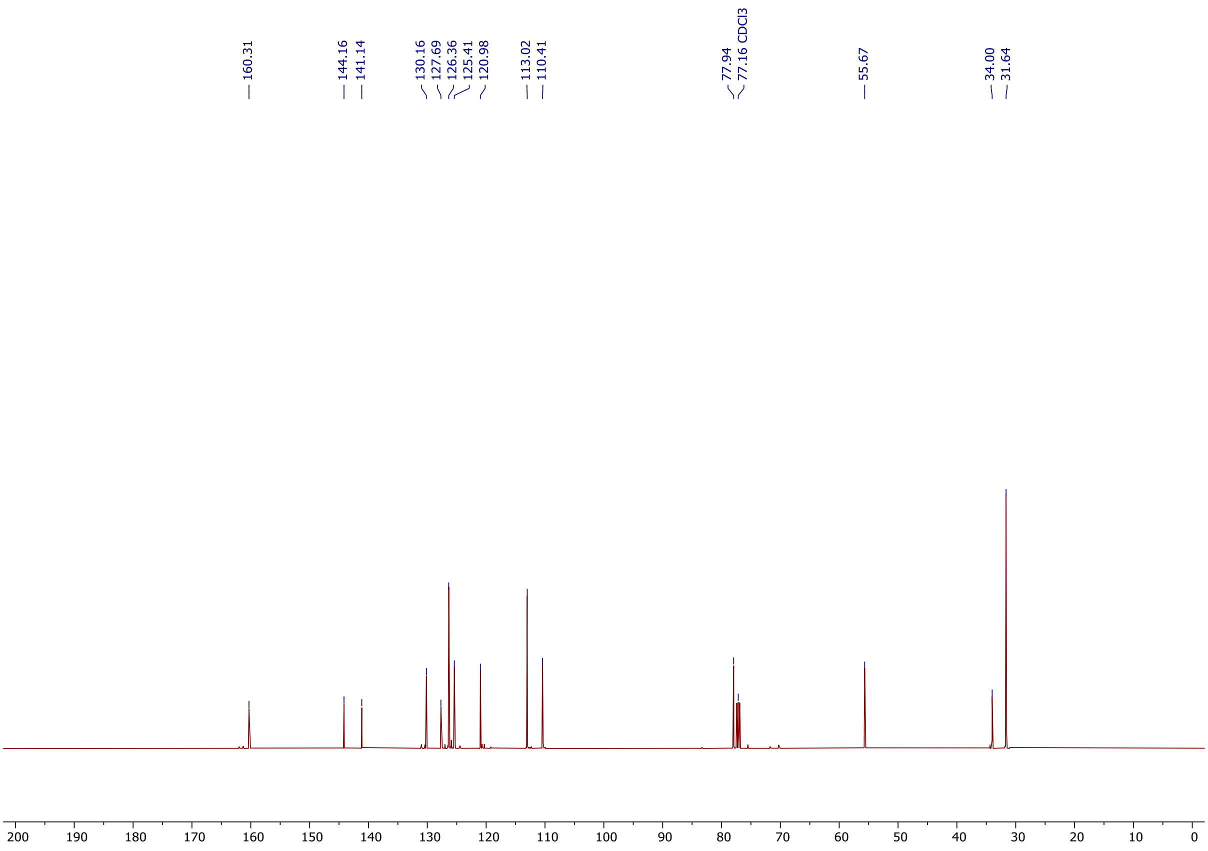

Figure S 152: ^{13}C NMR Spectrum of 8f in $CDCl_3$ after isolation via column chromatography.

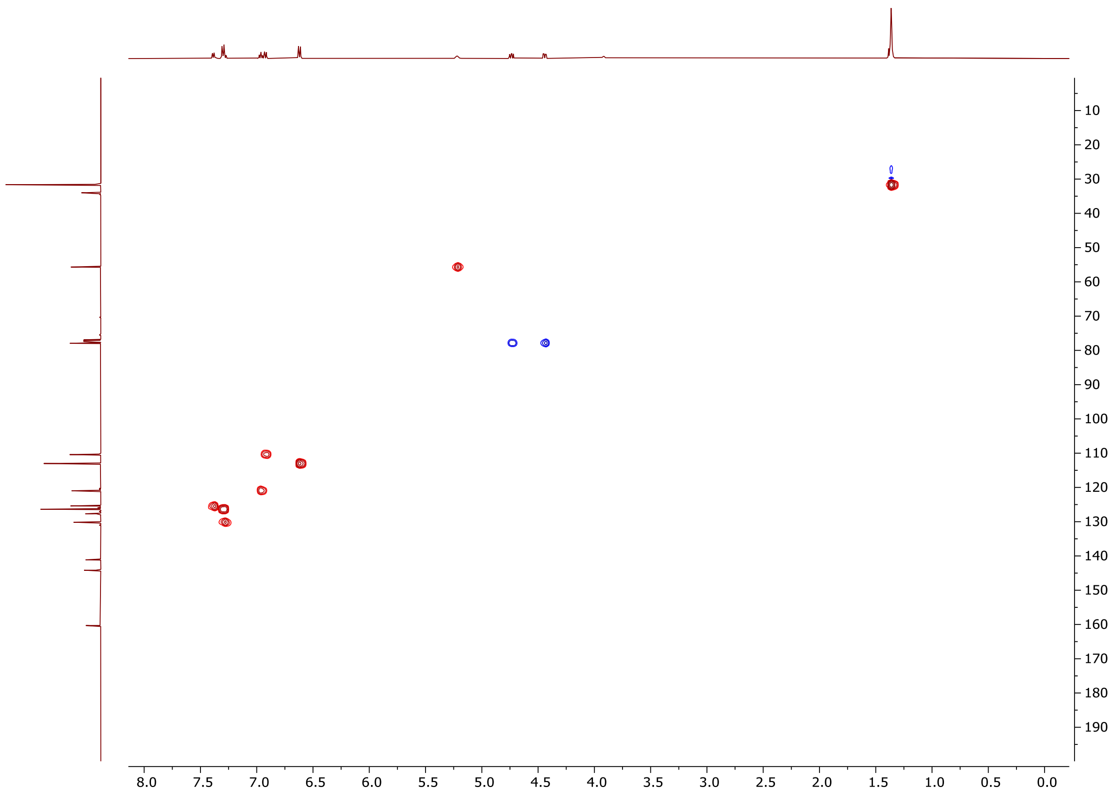

Figure S153: *^{1}H-^{13}C HSQC* NMR Spectrum of 8f in $CDCl_3$ after isolation via column chromatography.

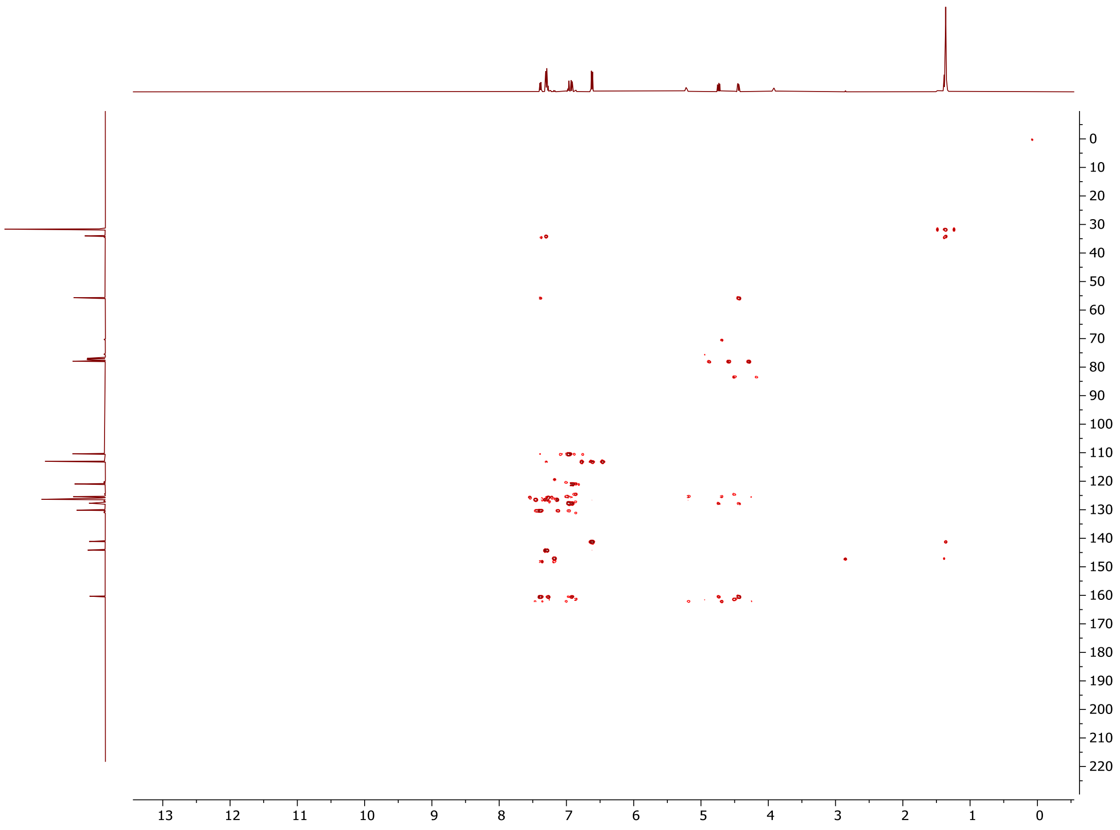

Figure S154: ^{1}H-^{13}C HMBC NMR Spectrum of 8f in $CDCl_3$ after isolation via column chromatography.

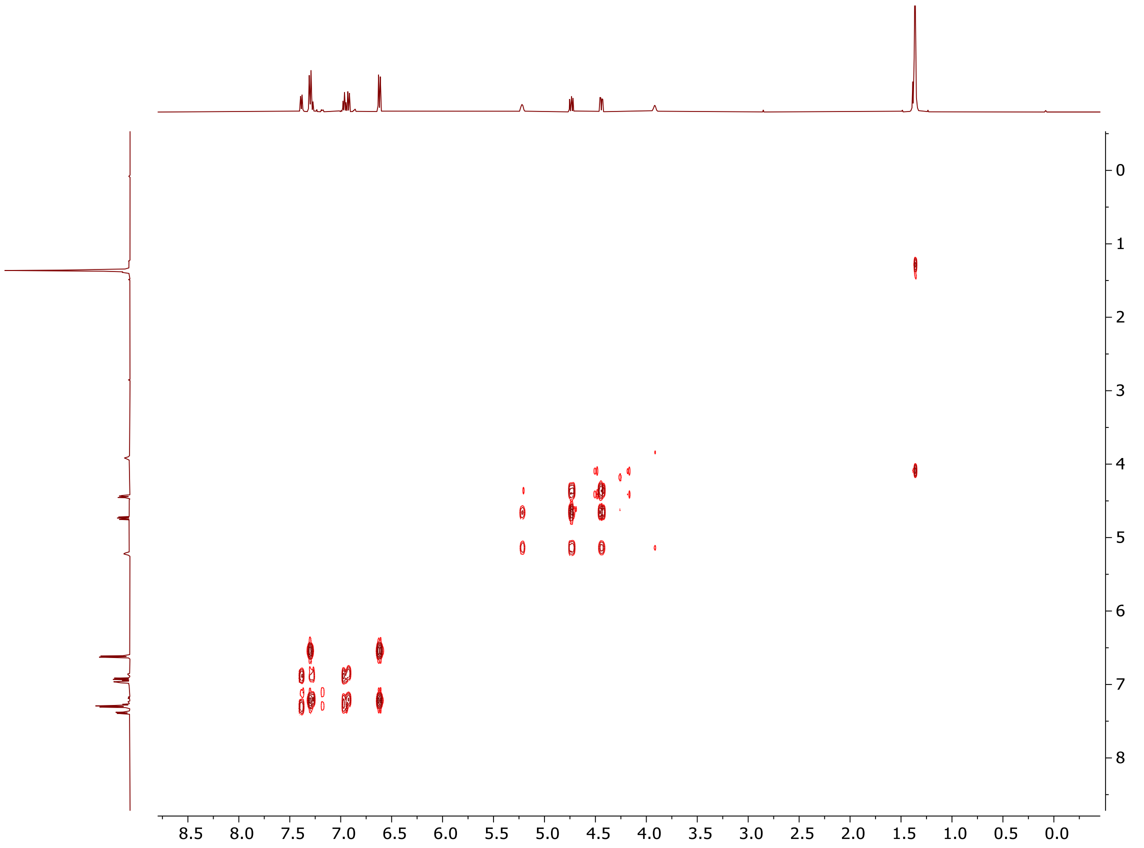

Figure S155: ^{1}H-^{1}H COSY NMR Spectrum of 8f in $CDCl_3$ after isolation via column chromatography.

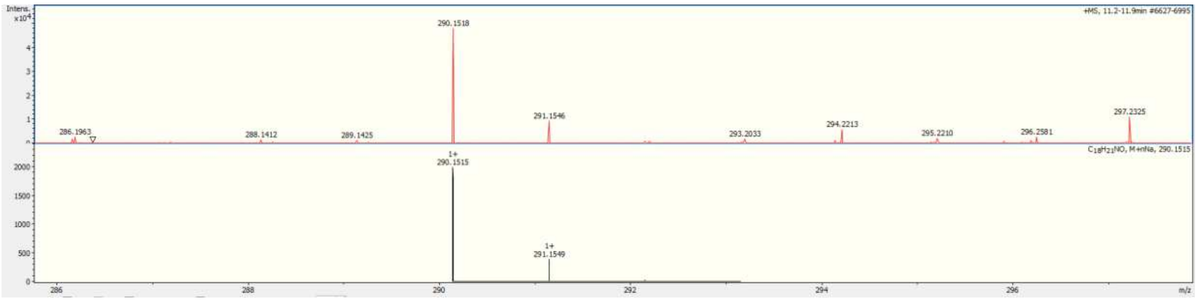

Figure S156: HRMS spectra for compound 8f.

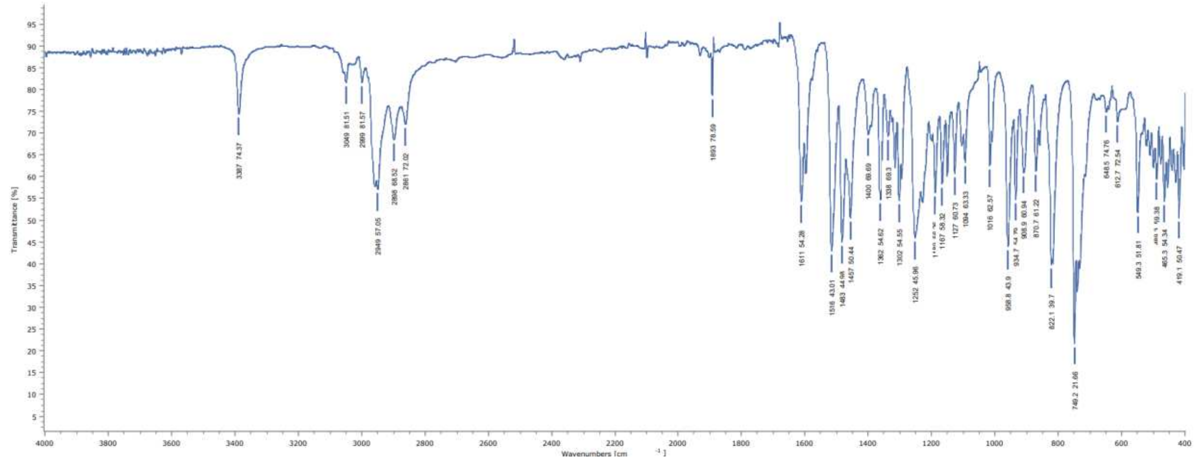

Figure S157: IR spectra for compound 8f.

11.4.7. tert-butyl 3-((4-(tert-butyl)phenyl)amino)indoline-1-carboxylate **8g**

Ratio of diastereoisomers 1:0.1

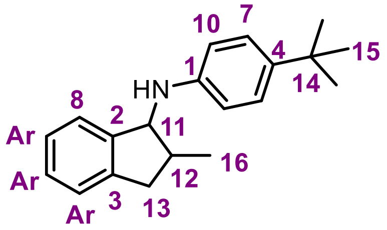

Required an additional zinc reduction step.

Colourless oil (252 mg, 54%)

R_f = 0.56 (30% DCM / 70% hexane)

<u>Data Reported for Major Diastereoisomer</u>

^{1}H NMR (CDCl$_3$, 500 MHz) δ 7.22 (app. d, J = 7.5 Hz, 1H, C^8-H), 7.16 – 7.12 (m, 4H, C^7-H and 2Ar-H), 7.10 -7.05 (m, 1H, Ar-H), 6.58 (d, J = 8.7 Hz, 2H, C^{10}-H), 4.43 (d, J = 6.8 Hz, 1H, C^{11}-H), 3.70 (*br* s, 1H, NH), 3.04 (dd, J = 15.5, 7.5 Hz, 1H, C^{13}-H), 2.47 (dd, J = 15.6, 7.6 Hz, 1H, C^{13}-H), 2.31 – 2.20 (m ,1H, C^{12}-H), 2.11 (s, 9H, C^{15}-H), 1.17 (d, J = 6.8 Hz, 3H, C^{16}-H).

^{13}C{^{1}H} NMR (CDCl$_3$, 126 MHz) δ 146.0 (C^1), 145.3 (C^2), 142.8 (C^3), 140.1 (C^4), 127.8 (Ar), 126.7 (Ar), 126.2 (C^7), 124.9 (C^8), 124.6 (Ar), 112.7 (C^{10}), 65.7 (C^{11}), 43.2 (C^{12}), 38.6 (C^{13}), 34.0 (C^{14}), 31.7 (C^{15}), 18.8 (C^{16}).

HRMS (ESI+): calcd for [M, $C_{20}H_{25}N$]$^+$ 280.2060, found 280.2070.

IR (Neat): 2952, 2863, 2109, 1613, 1516, 1300, 817 cm^{-1}.

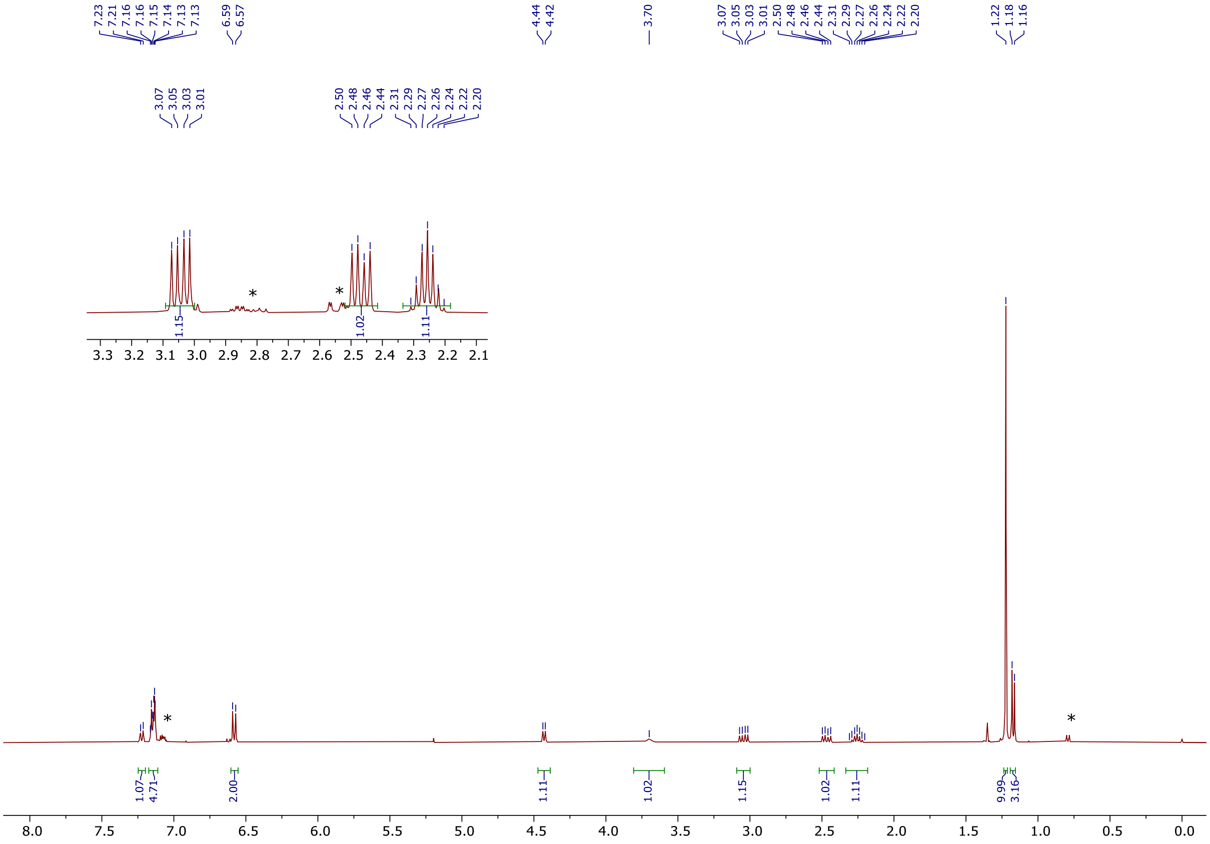

Figure S 158: ^{1}H NMR Spectrum of 8g in $CDCl_3$ after isolation via column chromatography.

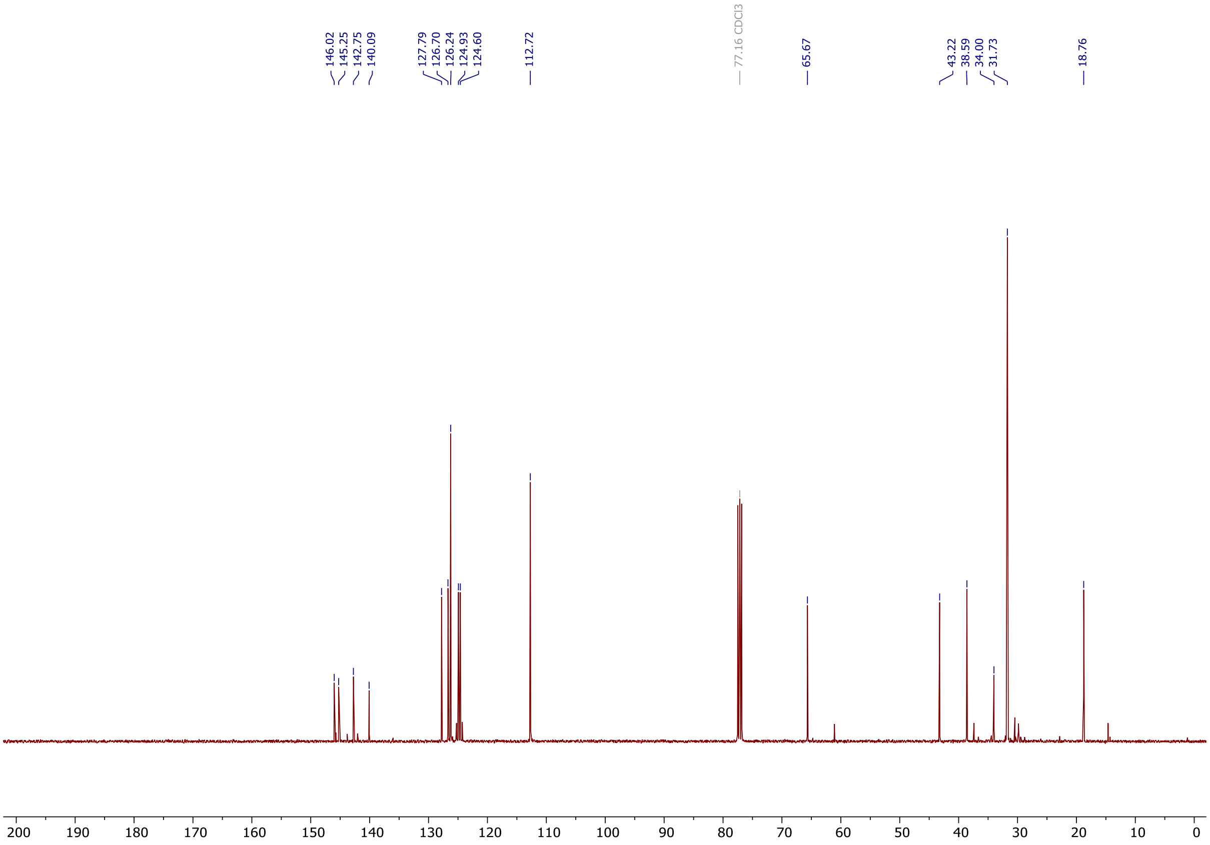

Figure S159: ^{13}C NMR Spectrum of 8g in $CDCl_3$ after isolation via column chromatography.

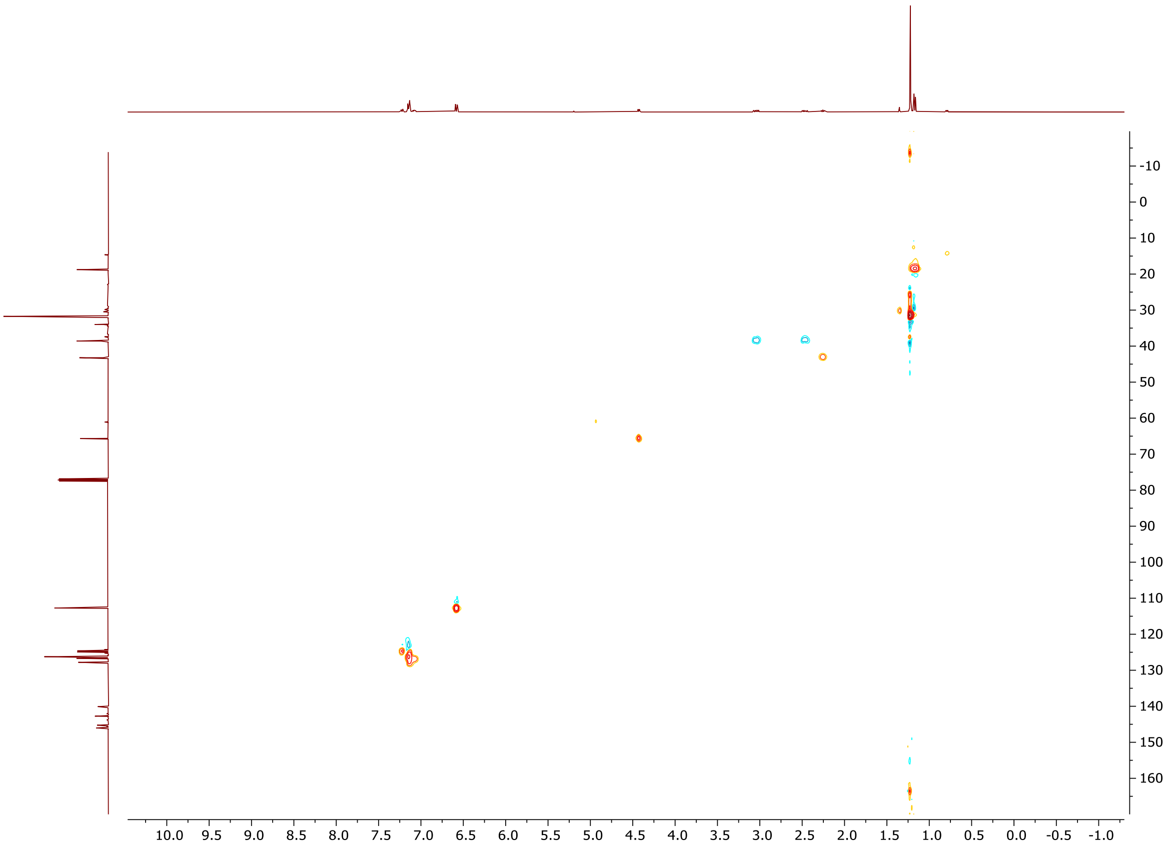

Figure S160: [1]*H-*[13]*C HSQC* NMR Spectrum of 8g in $CDCl_3$ after isolation via column chromatography.

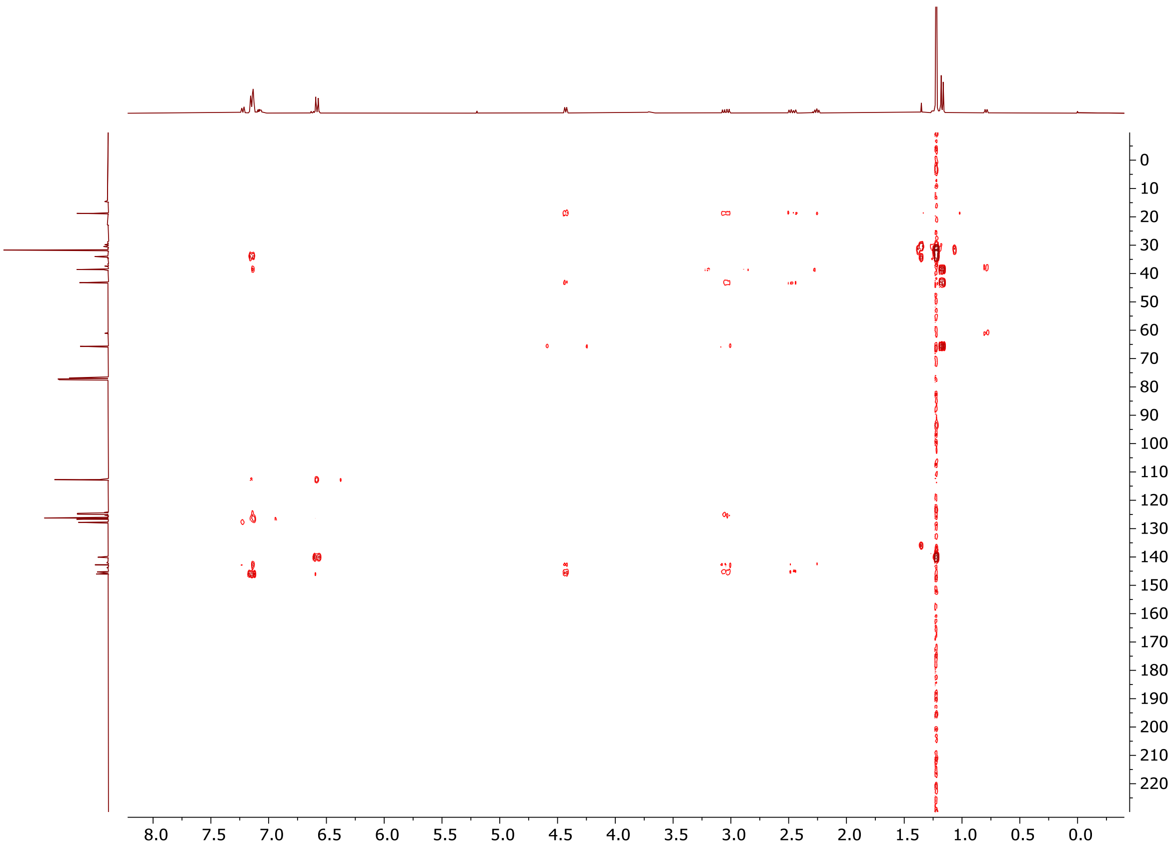

Figure S161: ¹H-¹³C HMBC NMR Spectrum of 8g in $CDCl_3$ after isolation via column *chromatography*.

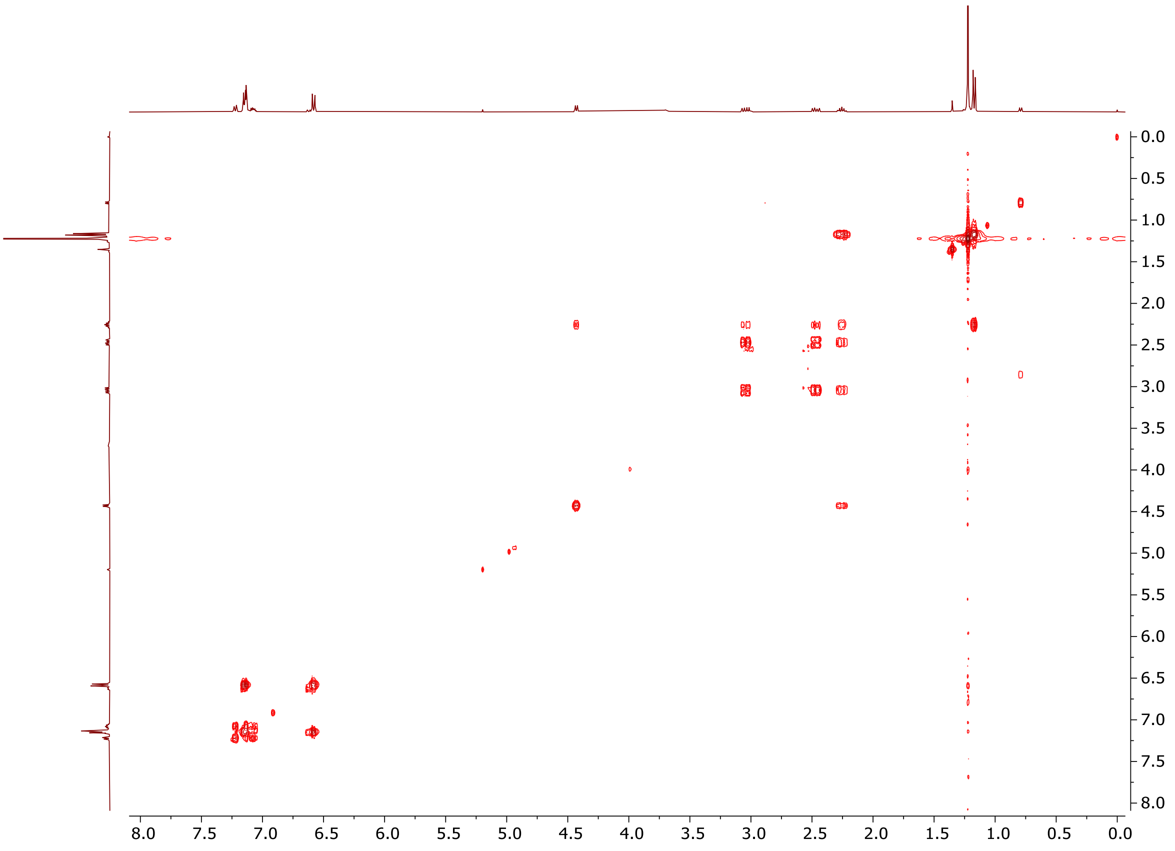

Figure S162: ¹H-¹H COSY NMR Spectrum of 8g in $CDCl_3$ after isolation via column *chromatography*.

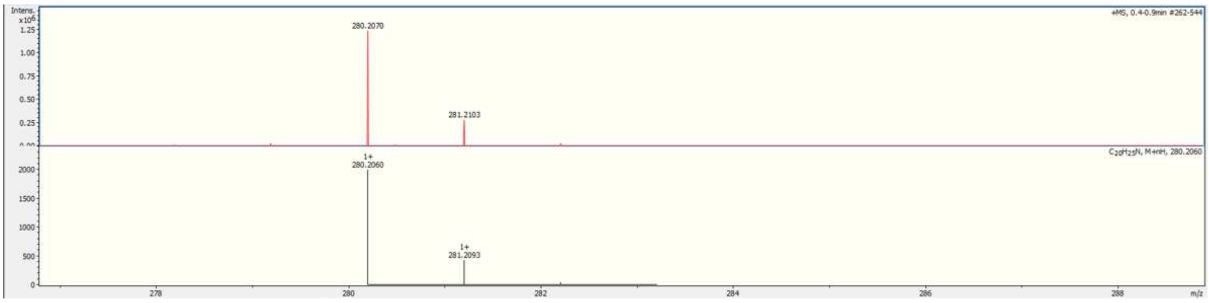

Figure S163: HRMS spectra for compound 8g.

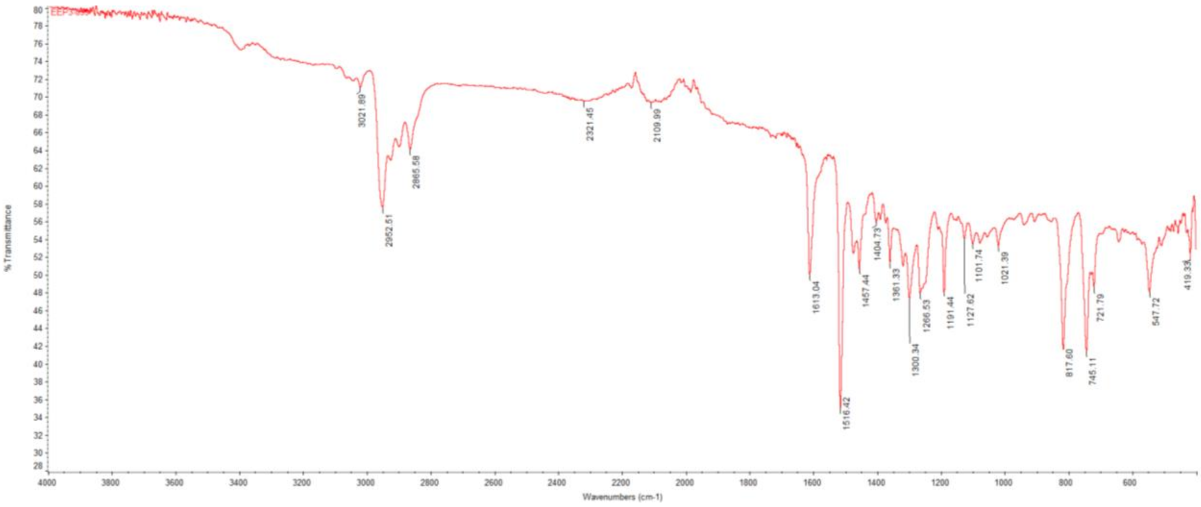

Figure S164: IR spectra for compound 8g.

11.4.8. 4-(tert-butyl)-N-(1-(2-methoxyphenyl)propan-2-yl)aniline **8h**

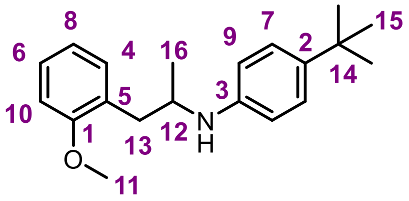

Light yellow oil (105 mg, 36%)

R_f = 0.15 (30% DCM / 70% hexane)

^{1}H NMR ($CDCl_3$, 500 MHz) δ 7.24 (app. d, J = 8.8 Hz, 2H, C^7-H), 7.25 – 7.21 (m, 1H, C^6-H), 7.17 (dd, J = 7.4, 1.6 Hz, 1H, C^4-H), 6.94 – 6.89 (m, 2H, C^8-H and C^{10}-H), 6.66 (app d, J = 8.8 Hz, 2H, C^9-H), 3.90 (s, 3H, C^{11}-H), 3.77 (app. ddq, J = 7.4, 7.4, 6.4, 1H, C^{12}-H), 3.18 (dd, J = 13.3, 7.4, 1H, C^{13}-H), 2.56 (dd, J = 13.3, 7.4 Hz, 1H, C^{13}-H), 2.19 (*br* s, 1H, NH), 1.33 (3, 9H, C^{15}-H), 1.19 (d, J = 6.4 Hz, 3H, C^{16}-H).

^{13}C{^{1}H} NMR ($CDCl_3$, 126 MHz) δ 157.8 (C^1), 145.4 (C^2), 139.4 (C^3), 131.3 (C^4), 127.8 (C^5), 127.6 (C^6), 126.1 (C^7), 120.6 (Ar), 112.8 (C^9), 110.4 (Ar), 55.3 (C^{11}), 49.1 (C^{12}), 37.9 (C^{13}), 34.0 (C^{14}), 31.7 (C^{15}), 20.8 (C^{16}).

HRMS (ESI+): calcd for [M, $C_{20}H_{27}NO$]$^+$ 298.2165, found 298.2176.

IR (Neat): 3407, 2958, 1614, 1518, 1241, 818, 749 cm^{-1}.

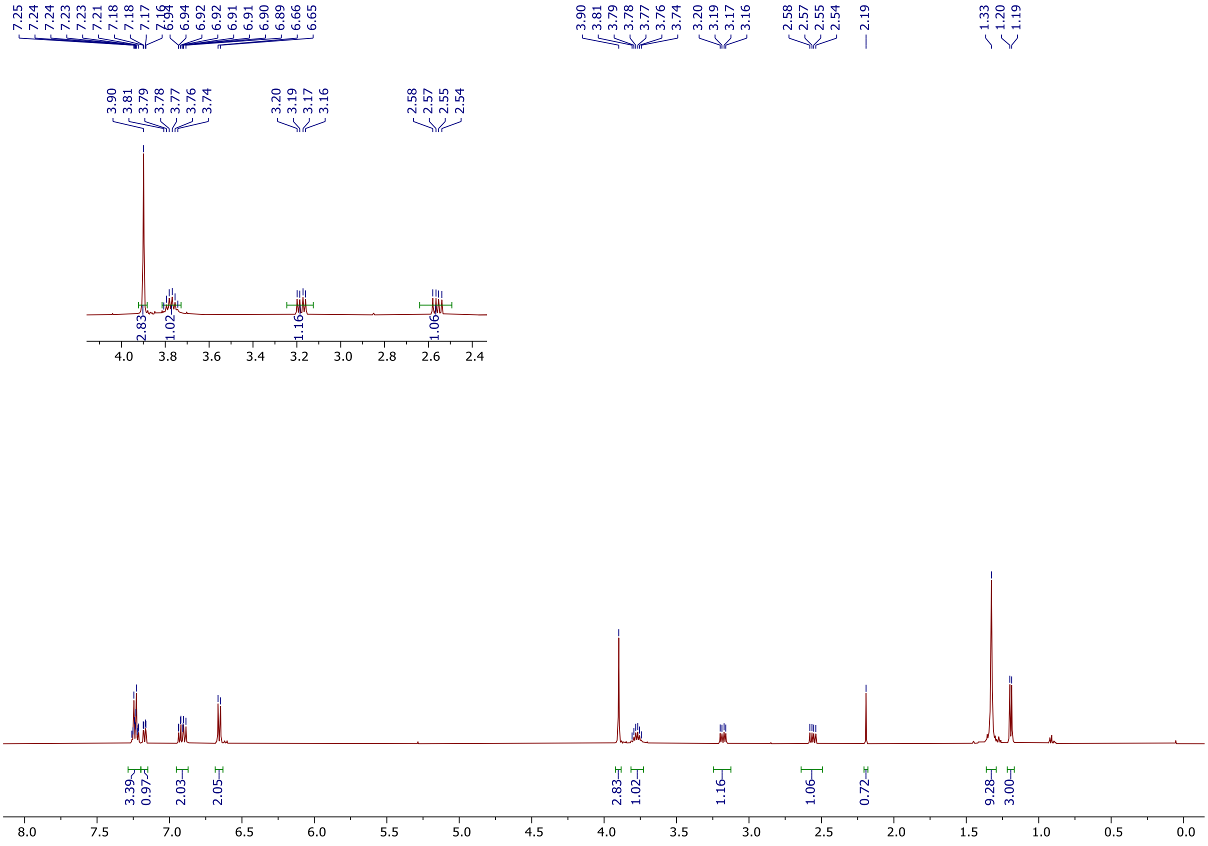

Figure S165: ^{1}H NMR Spectrum of 8h in $CDCl_3$ after isolation via column chromatography.

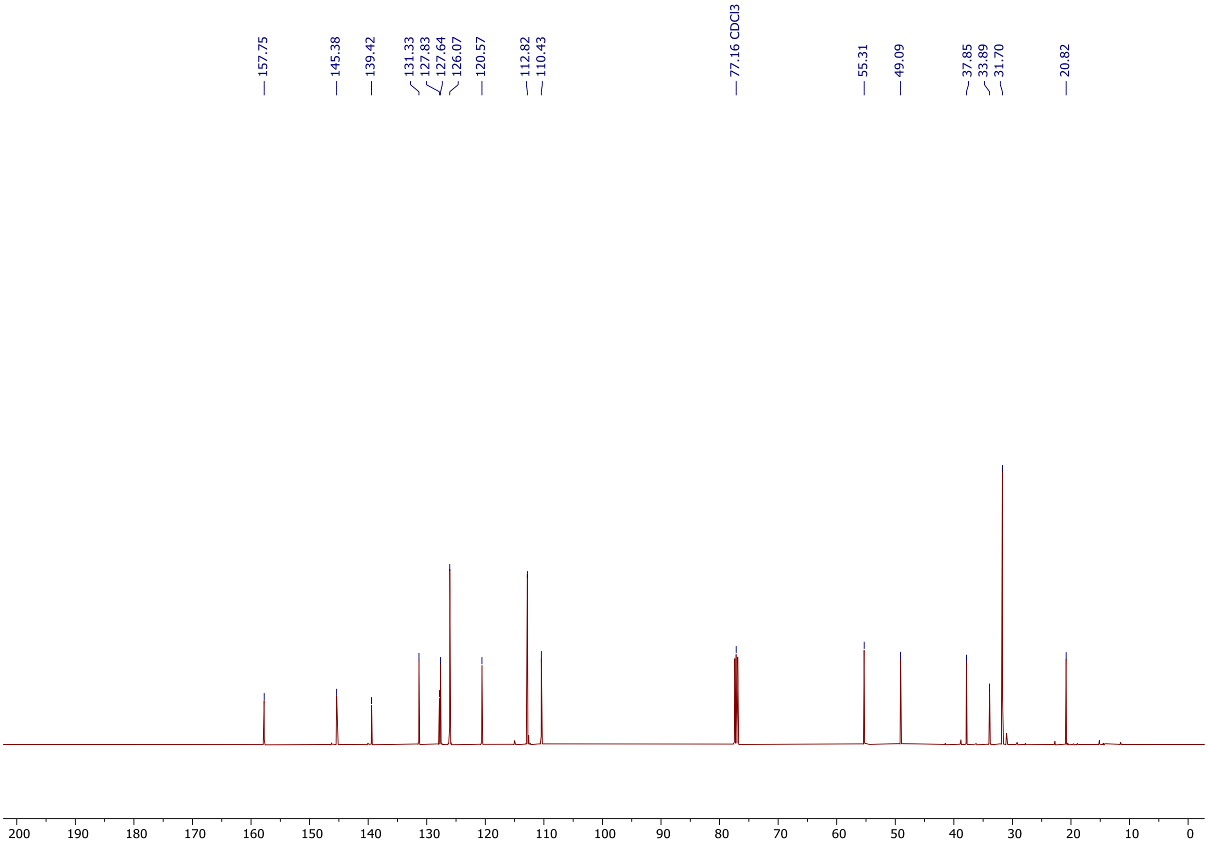

Figure S166: ^{13}C NMR Spectrum of 8h in $CDCl_3$ after isolation via column chromatography.

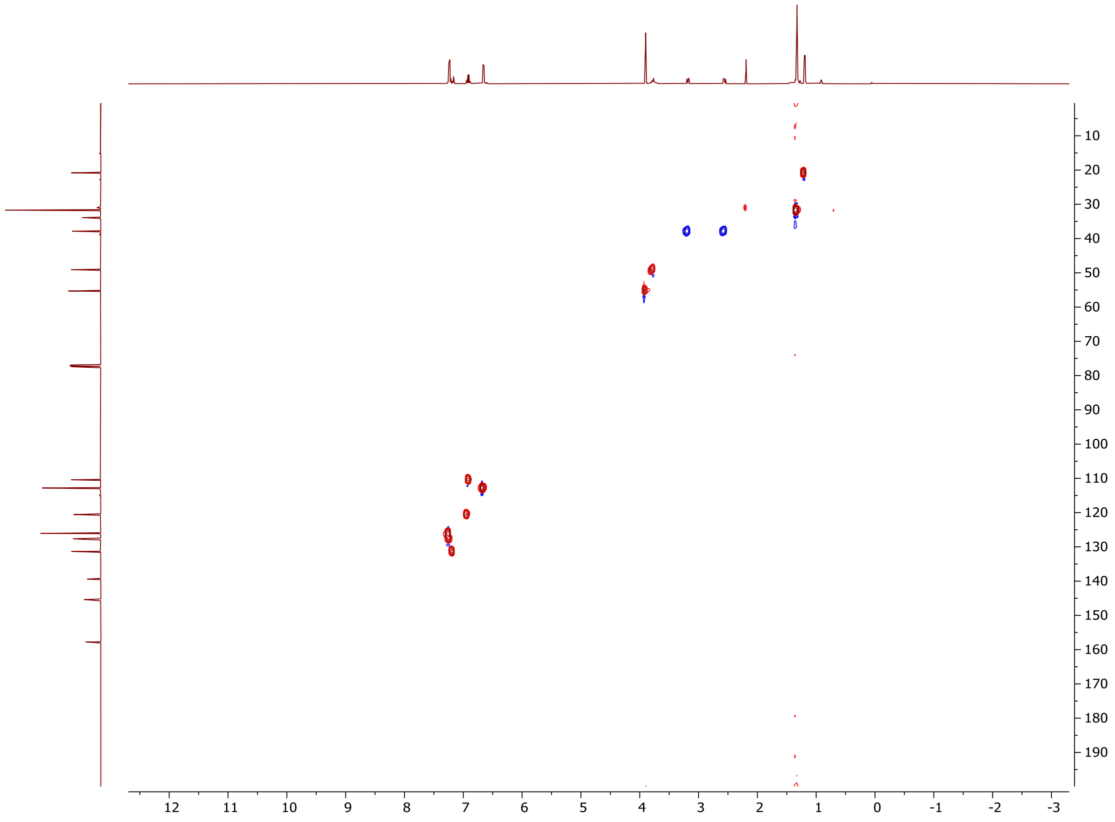

Figure S167: ¹H-¹³C HSQC NMR spectrum of 8h in $CDCl_3$ after isolation via column chromatography.

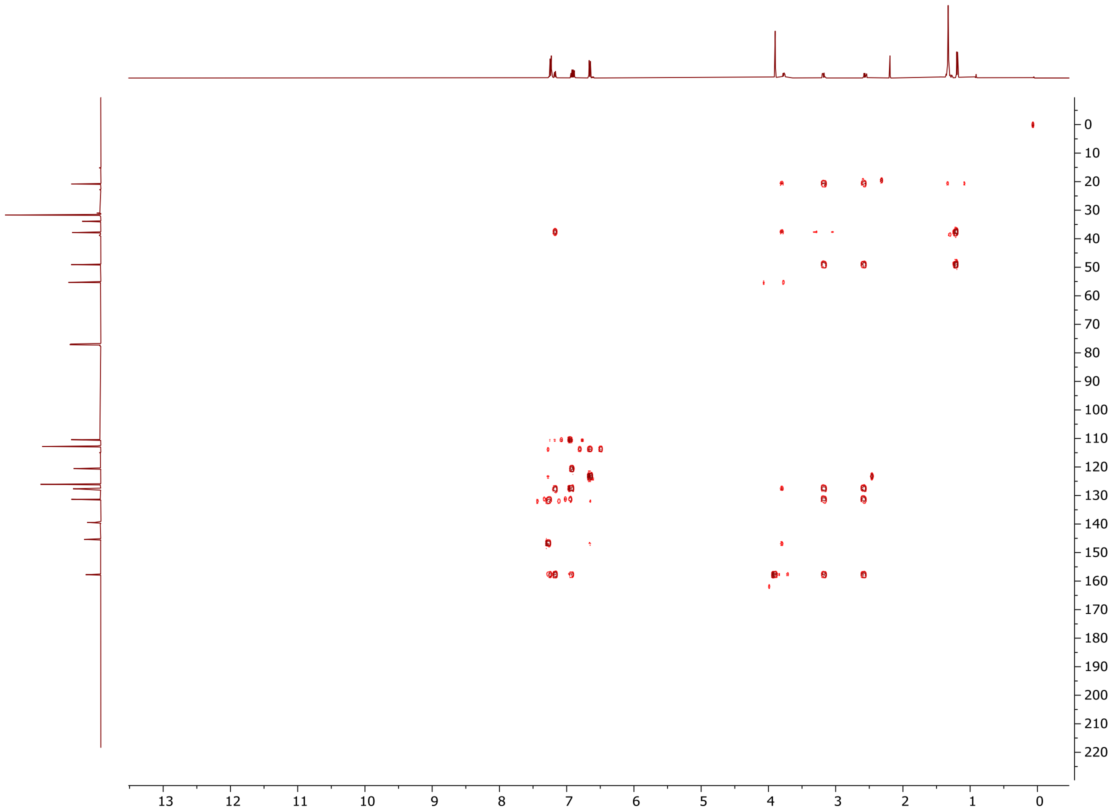

Figure S168: ¹H-¹³C HMBC NMR spectrum of 8h in $CDCl_3$ after isolation via column chromatography.

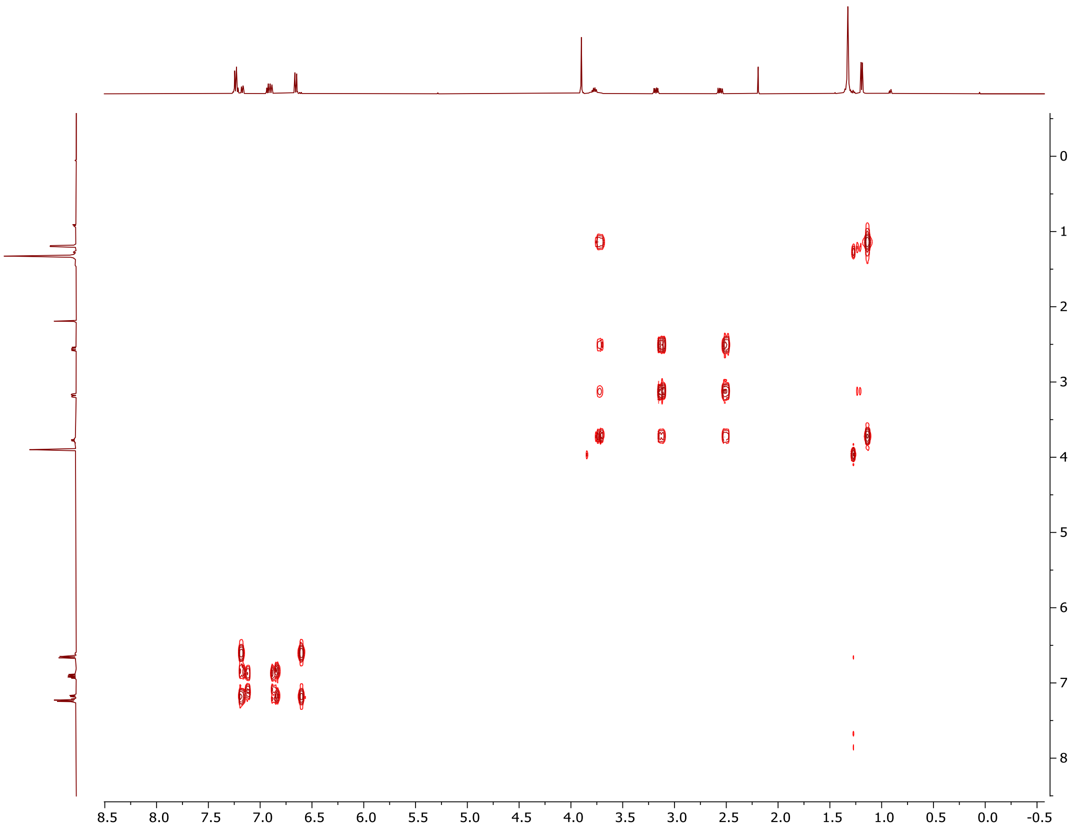

Figure S169: ^{1}H-^{1}H COSY NMR spectrum of 8h in $CDCl_3$ after isolation via column chromatography.

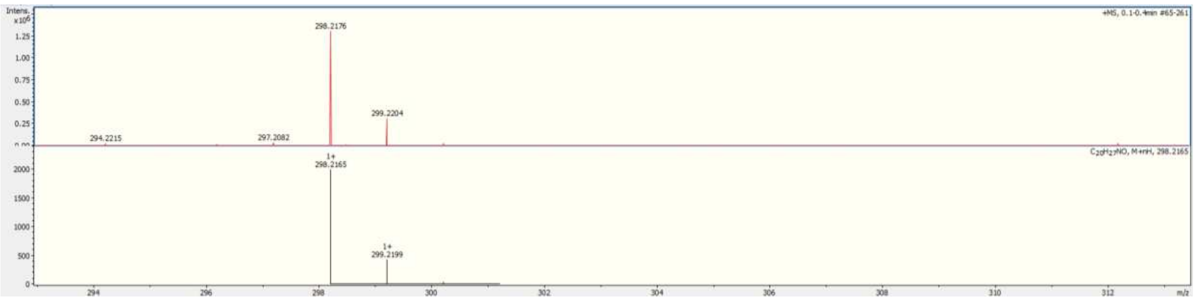

Figure S170: HRMS spectra for compound 8h.

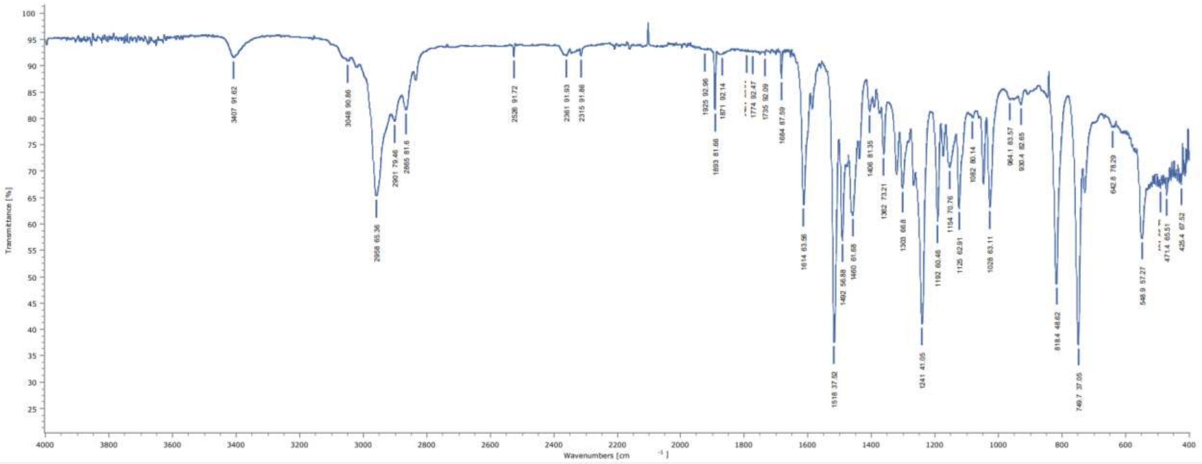

Figure S171: IR spectra for compound 8h.

11.4.9. 4-(tert-butyl)-N-(1-phenylpropan-2-yl)aniline **8i**

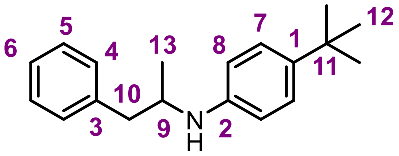

Light yellow oil (119 mg, 45%)

R_f = 0.20 (30% DCM / 70% hexane)

^{1}H NMR (CDCl$_3$, 500 MHz) δ 7.36 (app. t, J= 7.3 Hz, 2H, C^5-H), 7.29 – 7.23 (m, 5H, C^4,C^6,C^7-H), 6.66 (app. d, J = 8.6 Hz, 2H, C^8-H), 3-83 – 3.77 (m, 1H, C^9-H), 3.52 (*br* s, 1H, NH), 3.02 (dd, J = 13.4, 4.6 Hz, 1H, C^{10}-H), 2.74 (dd, J = 13.4, 7.3 Hz, 1H, C^{10}-H), 1.36 (s, 9H, C^{12}-H), 1.21 (d, J = 6.2 Hz, 3H, C^{13}-H).

^{13}C{^{1}H} NMR (CDCl$_3$, 126 MHz) δ 145.0 (C^1), 140.0 (C^2), 138.9 (C^3), 129.6 (C^4), 128.4 (C^5), 126.4 (C^6), 126.2 (C^7), 113.1 (C^8), 49.7 (C^9), 42.3 (C^{10}), 34.0 (C^{11}), 31.7 (C^{12}), 20.5 (C^{13}).

HRMS (ESI+): calcd for for [M, $C_{19}H_{25}N$]$^+$ 268.2060, found 286.2072.

IR (Neat): 3060, 2961, 1613, 1517 817 698 cm^{-1}.

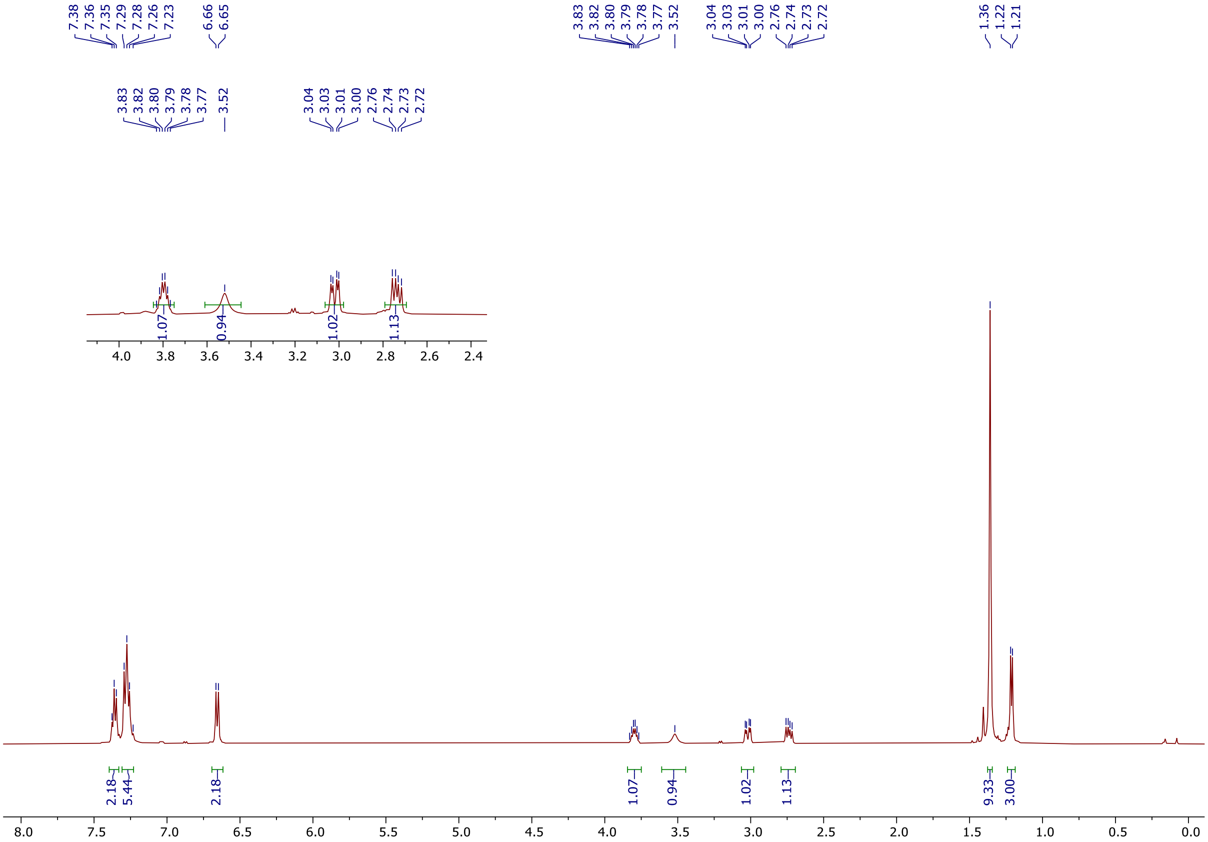

Figure S172: ^{1}H NMR Spectrum of 8i in CDCl$_3$ after isolation via column chromatography.

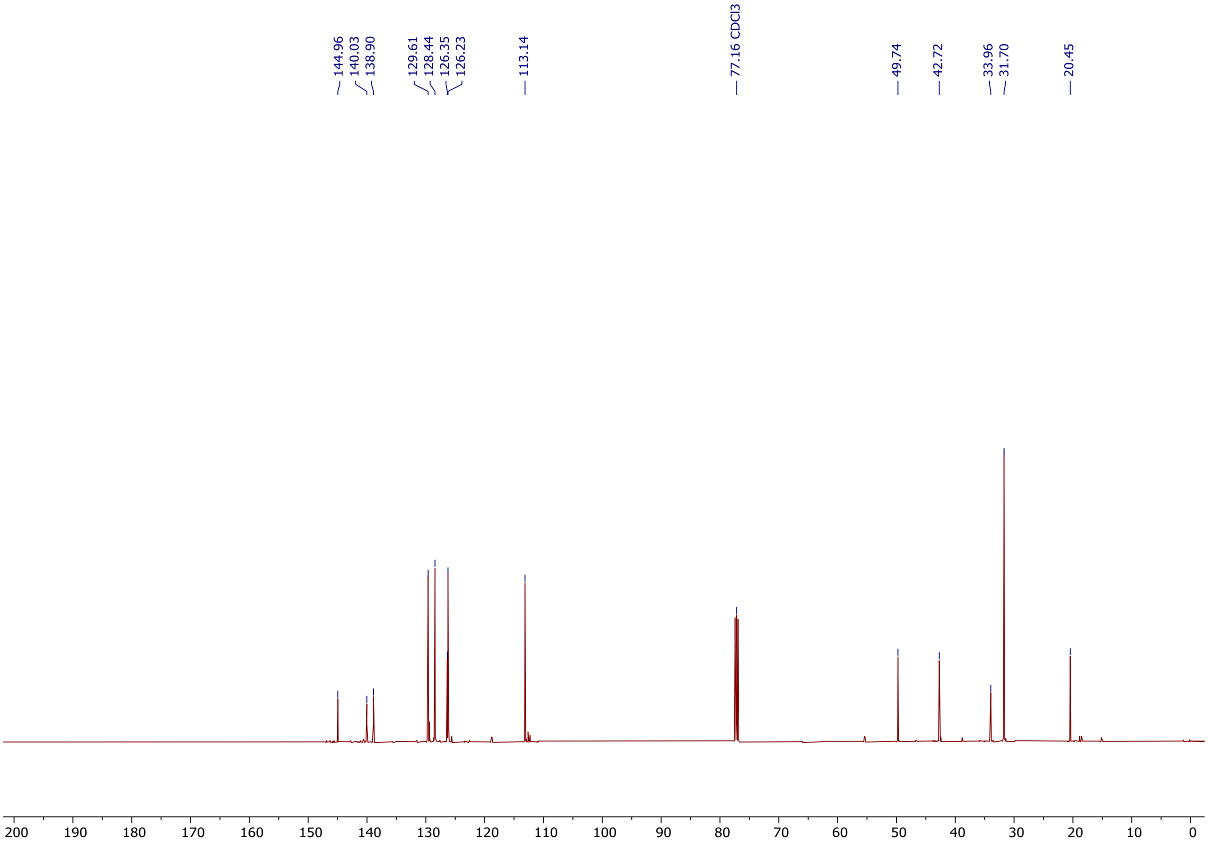

Figure S173: ^{13}C NMR Spectrum of 8i in $CDCl_3$ after isolation via column chromatography.

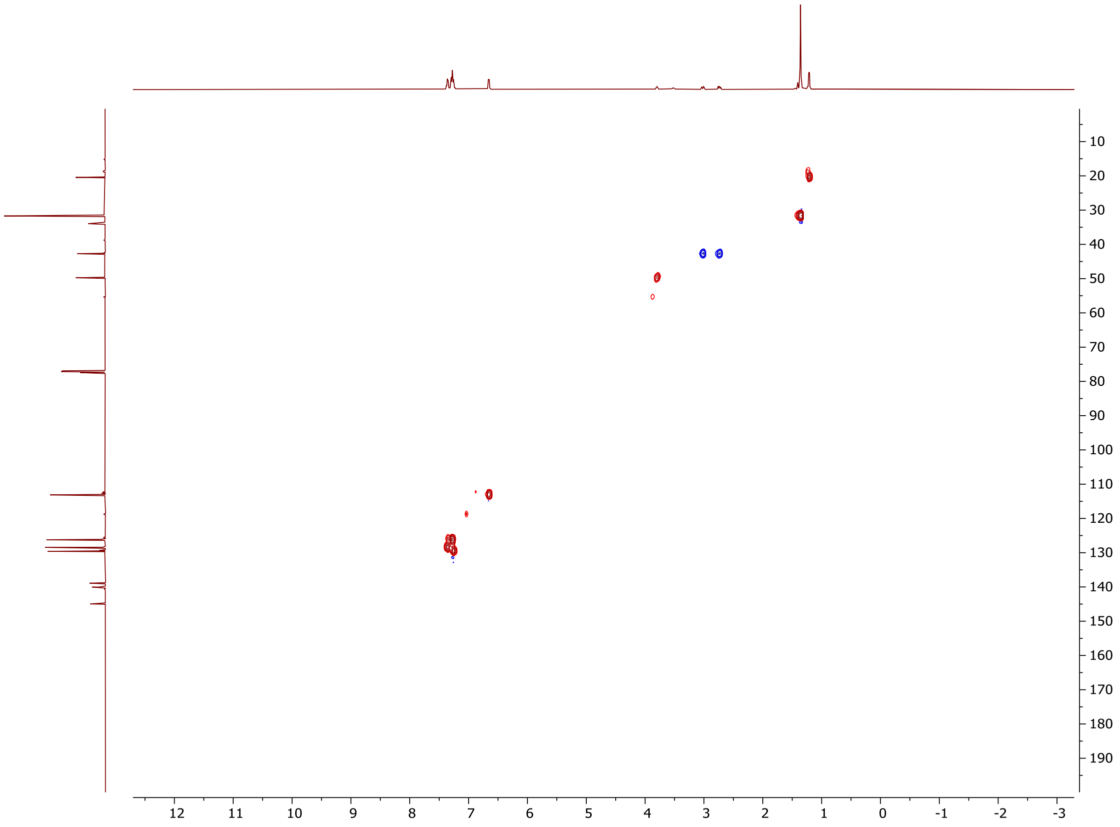

Figure S174: [1]*H-*[13]*C HSQC NMR spectrum of* 8i in $CDCl_3$ after isolation via column chromatography.

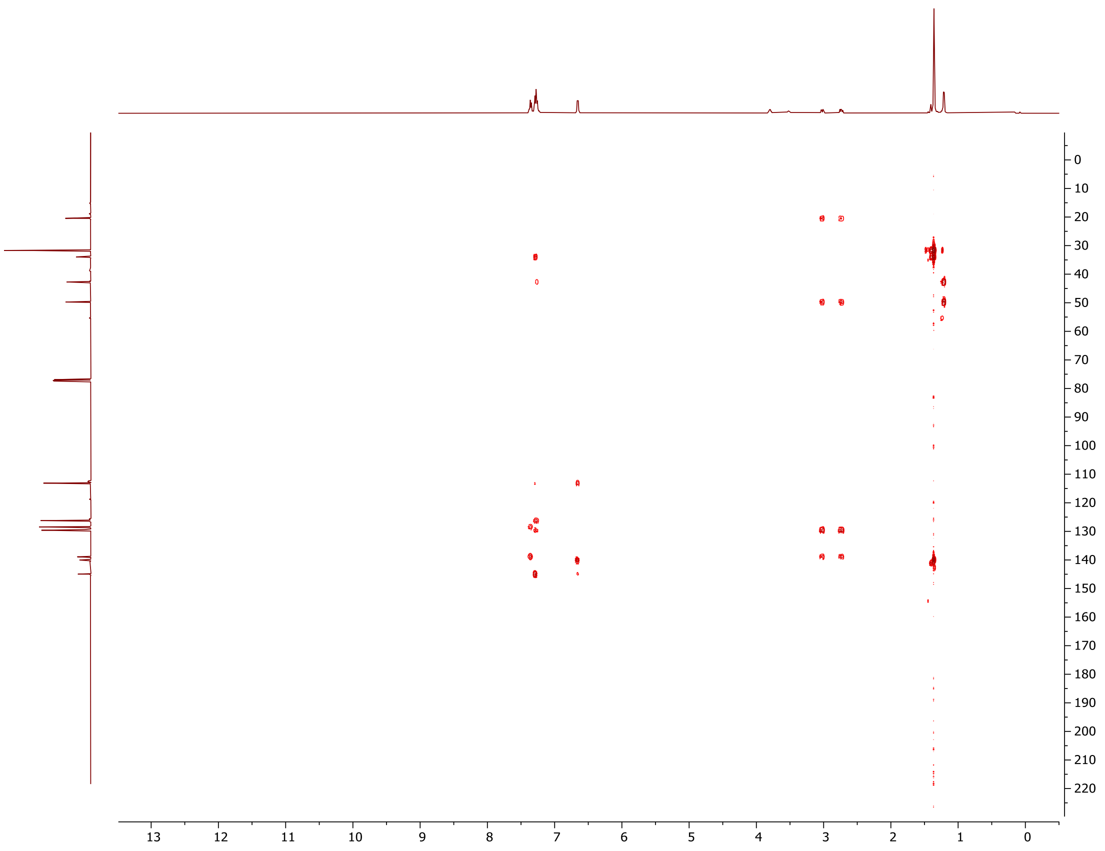

Figure S175: ¹H-¹³C HMBC NMR spectrum of 8i in $CDCl_3$ after isolation via column chromatography.

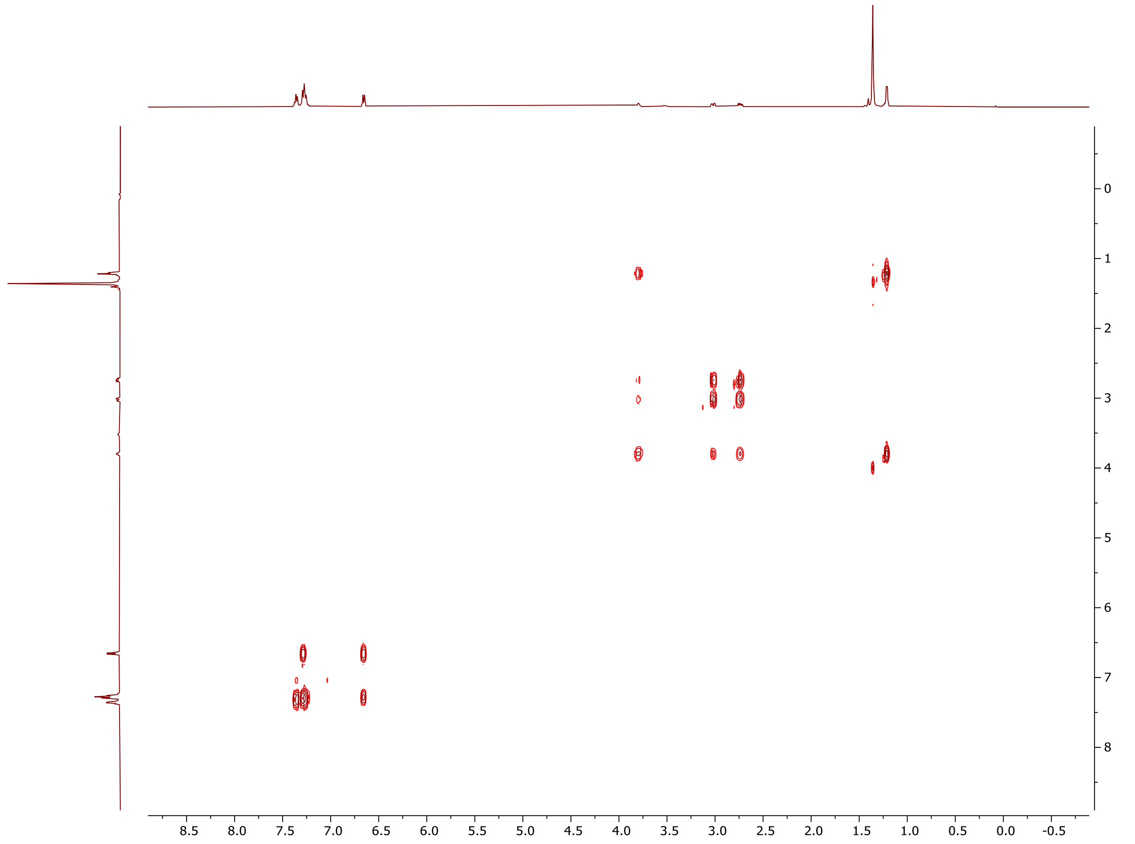

Figure S176: ¹H-¹H COSY NMR spectrum of 8i in $CDCl_3$ after isolation via column chromatography.

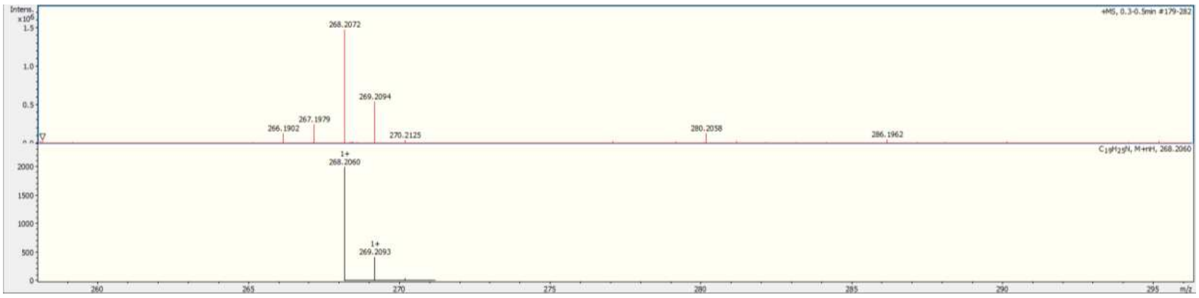

Figure S177: HRMS spectra for compound 8i.

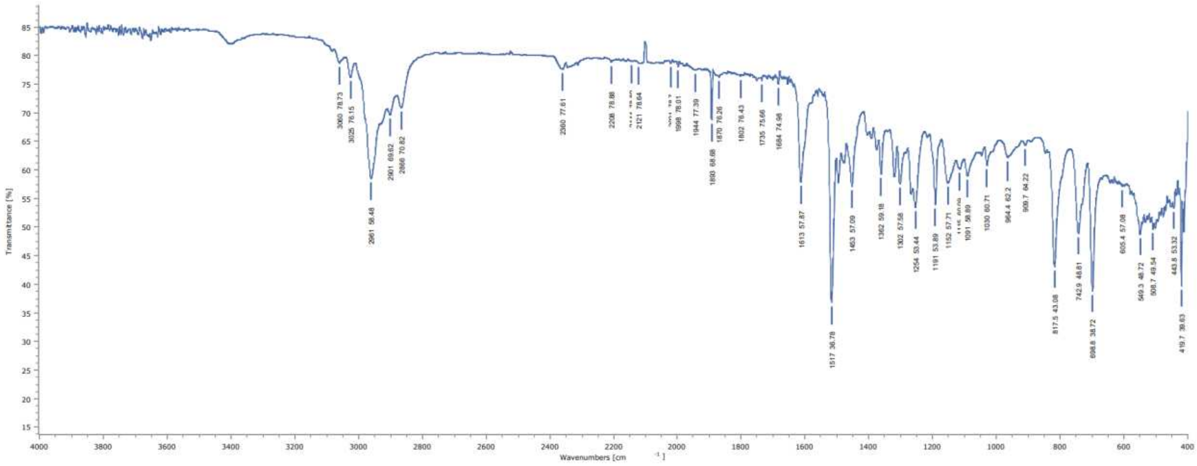

Figure S178: IR spectra for compound 8i.

11.4.10. 4-(tert-butyl)-N-(hexan-2-yl)aniline **8j**

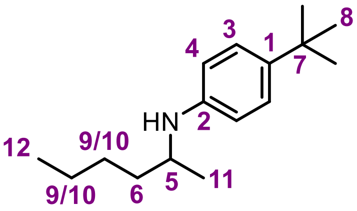

Light yellow oil (11.7 mg, 5%)

R_f = 0.22 (30% DCM / 70% hexane)

^{1}H NMR ($CDCl_3$, 400 MHz) δ 7.19 (d, J = 8.8 Hz, 2H, C^3-H), 6.53 (d, J = 8.8 Hz, 2H, C^4-H), 3.42 (app. p, J = 6.22, 1H, C^5-H), 3.35 (*br* s, 1H, NH), 1.56 – 1.53 (m, 2H, C^6-H), 1.43 – 1.32 (m, 4H, $C^{9/10}$-H), 1.28 (s, 9H, C^8-H), 1.17 (d, J = 6.3 Hz, 3H, C^{11}-H), 0.91 (t, J = 6.9 Hz, 3H, C^{12}-H).

^{13}C{^{1}H} NMR ($CDCl_3$, 126 MHz) δ 145.5 (C^2), 139.6 (C^1), 126.2 (C^3), 112.8 (C^4), 48.7 (C^5) 37.2 (C^6), 33.9 (C^7), 31.7 (C^8), 28.6 (C^9), 22.9 (C^{10}), 21.1 (C^{11}), 14.3 (C^{12}).

HRMS (ESI+): calcd for [M, $C_{16}H_{28}N$]$^+$ 234.2216, found 234.2228.

IR (Neat): 2958, 2929, 1893, 1614, 1518, 1074, 817, 697 cm^{-1}.

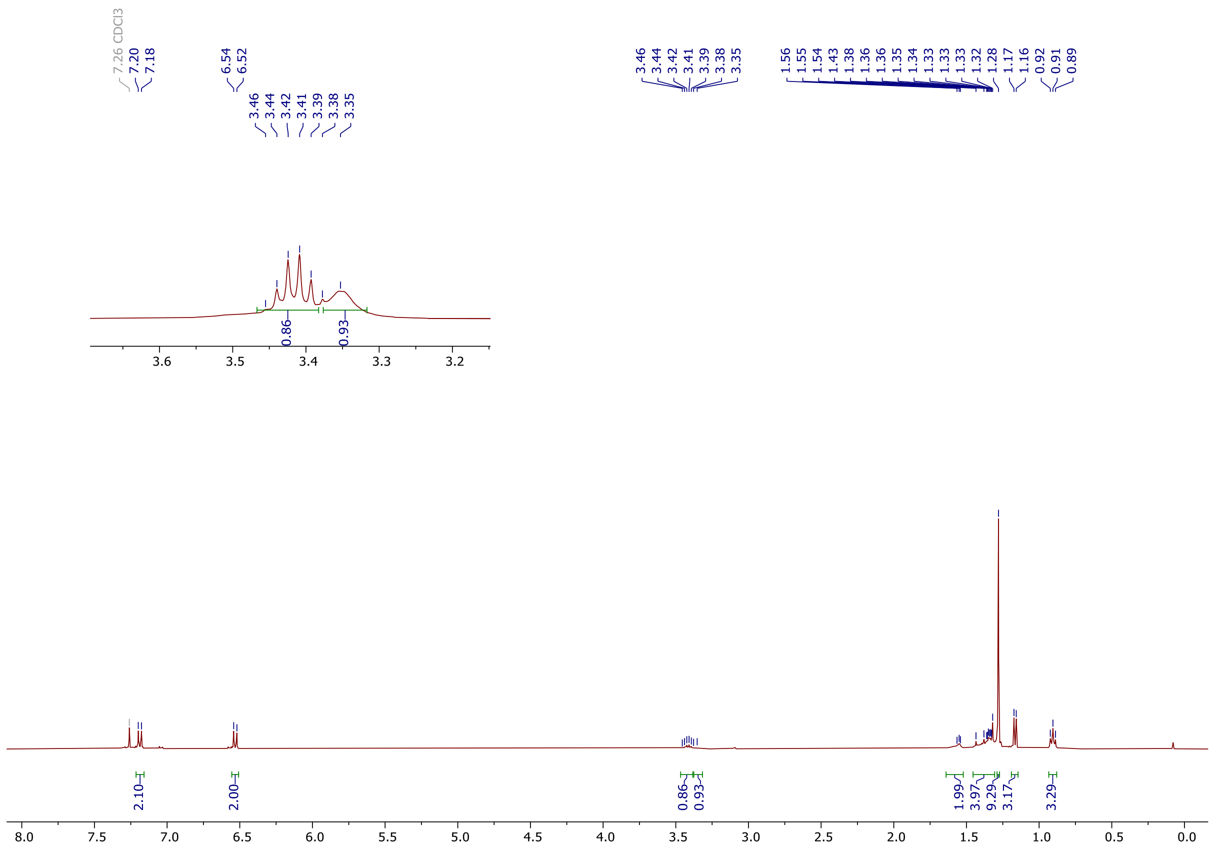

Figure S179: ^{1}H NMR Spectrum of 8j in $CDCl_3$ after isolation via column chromatography.

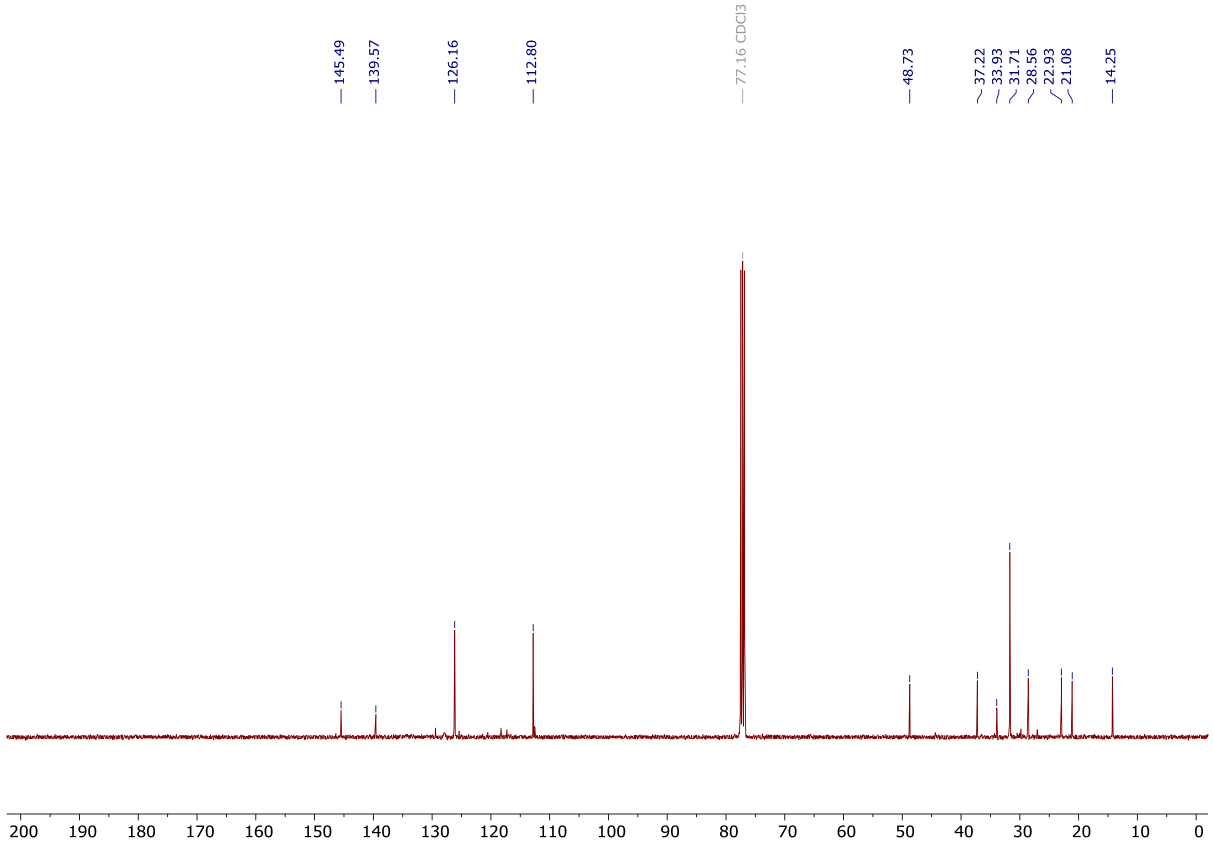

Figure S180: ^{13}C NMR Spectrum of 8j in $CDCl_3$ after isolation via column chromatography.

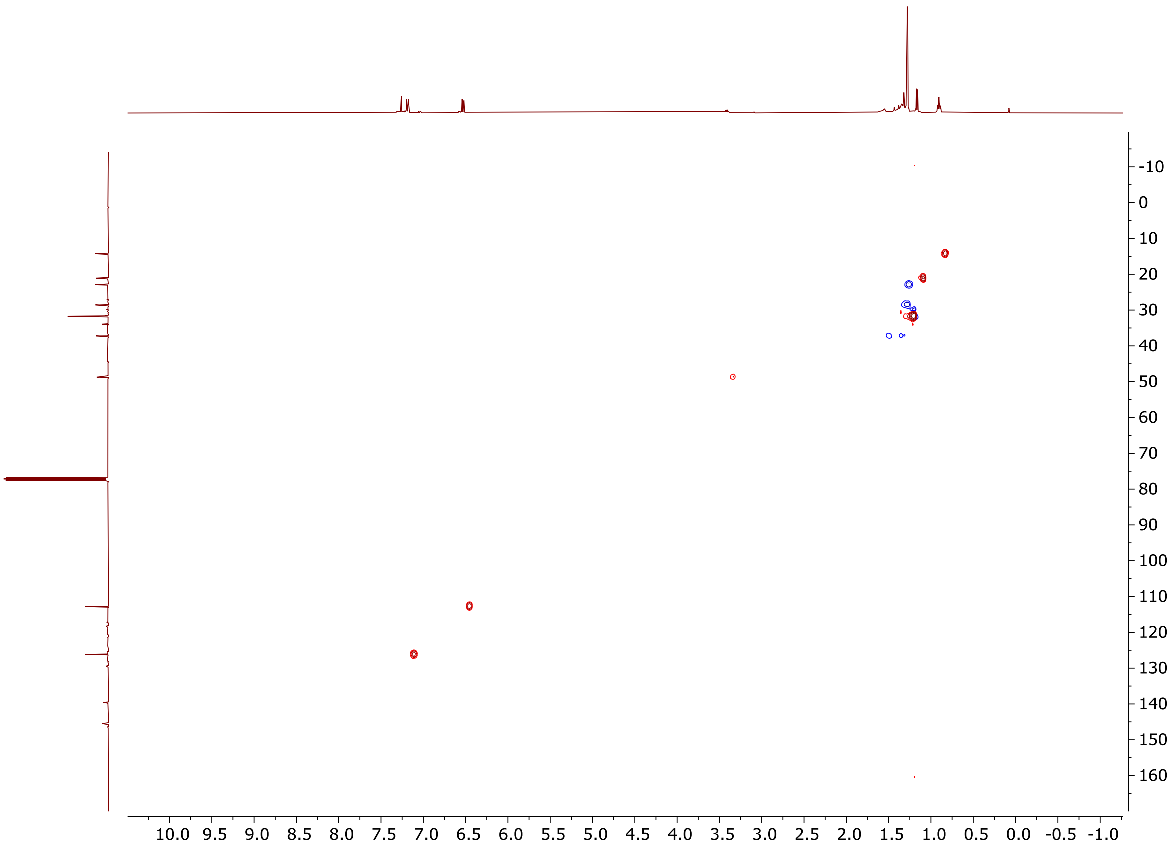

Figure S181: ¹H-¹³C HSQC NMR Spectrum of 8j in $CDCl_3$ after isolation via column chromatography.

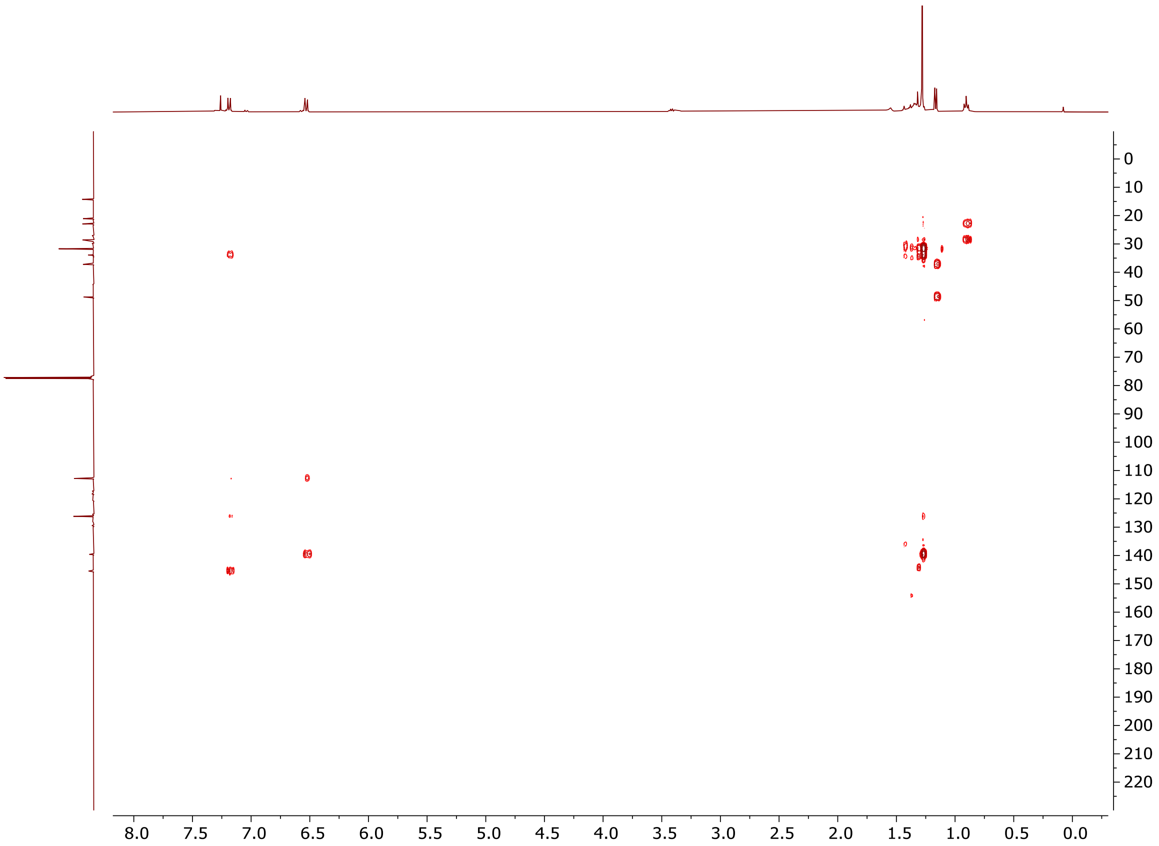

Figure S182: ¹H-¹³C HMBC NMR Spectrum of 8j in $CDCl_3$ after isolation via column chromatography.

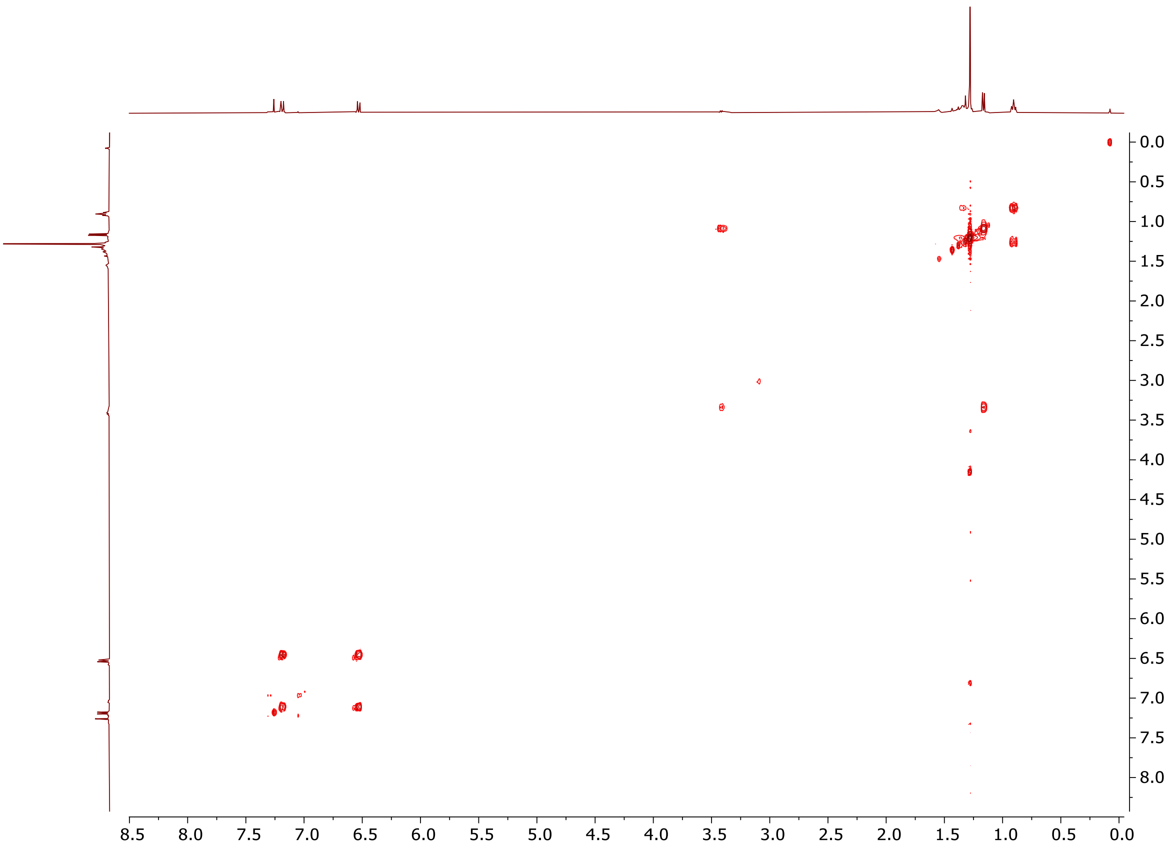

Figure S183: ^{1}H-^{1}H COSY NMR Spectrum of 8j in $CDCl_3$ after isolation via column chromatography.

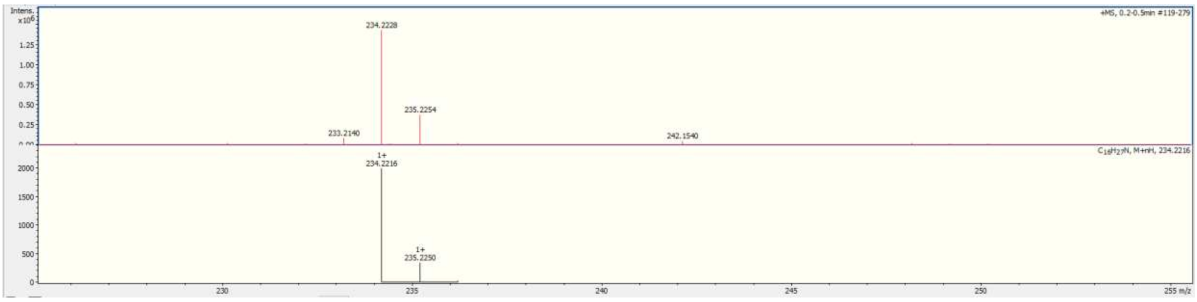

Figure S184: HRMS spectra for compound 8j.

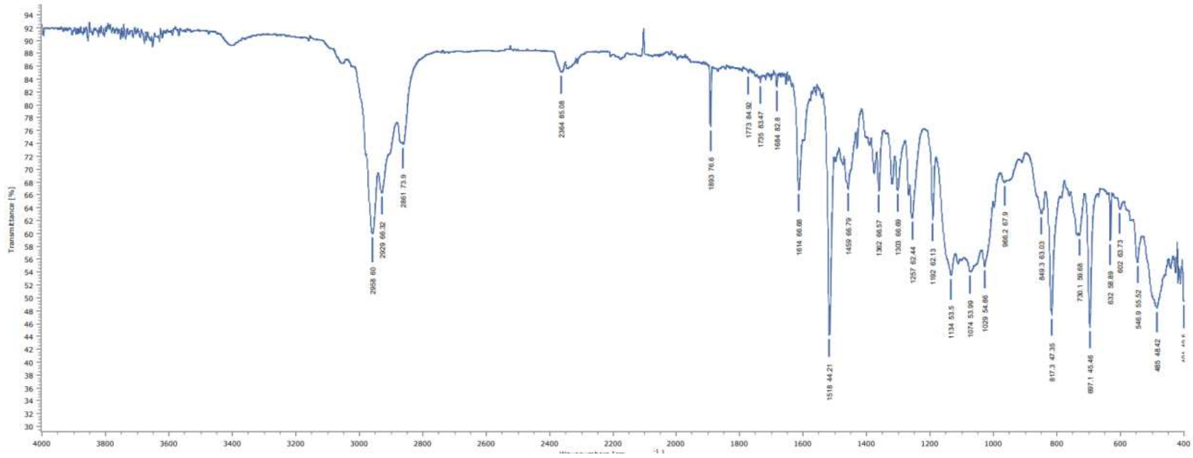

Figure S185: IR spectra for compound 8j.

11.4.11. 4-(tert-butyl)-N-(1-(4-fluorophenyl)propan-2-yl)aniline **8k**

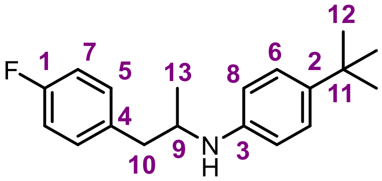

yellow oil (144 mg, 50%)

R_f = 0.29 (30% DCM / 70% hexane)

^{1}H NMR ($CDCl_3$, 500 MHz) δ 7.29 (app. d, J= 8.6 Hz, 2H, C^6-H), 7.20 (dd, J = 8.6, 5.9 Hz, 2H, C^5-H), 7.04 (app. t, J = 8.7 Hz, 2H, C^7-H), 6.64 (app. d, J = 8.5 Hz, 2H, C^8-H), 3-80 – 3.74 (m, 1H, C^9-H), 2.47 (*br* s, 1H, NH), 2.96 (dd, J = 13.6, 4.9 Hz, 1H, C^{10}-H), 2.73 (dd, J = 13.6, 6.7 Hz, 1H, C^{10}-H), 1.36 (s, 9H, C^{12}-H), 1.20 (d, J = 6.4 Hz, 3H, C^{13}-H).

^{13}C{^{1}H} NMR ($CDCl_3$, 126 MHz) δ 161.7 (d, J = 244.8 Hz, C^1), 144.9 (C^3), 140.2 (C^2), 134.5 (d, J = 3.3 Hz, C^4), 131.0 (d, 7.7 Hz, C^5), 126.3 (C^6), 115.2 (d, J = 20.9 Hz, C^7), 113.1 (C^8), 49.7 (C^9), 41.7 (C^{10}), 34.0 (C^{11}), 31.7 (C^{12}), 20.4 (C^{13}).

HRMS (ESI+): calcd for [M, $C_{19}H_{24}NF$]$^+$ 286.1966, found 286.1985.

IR (Neat): 2961, 2865, 1614, 1518, 1482, 1319 cm^{-1}.

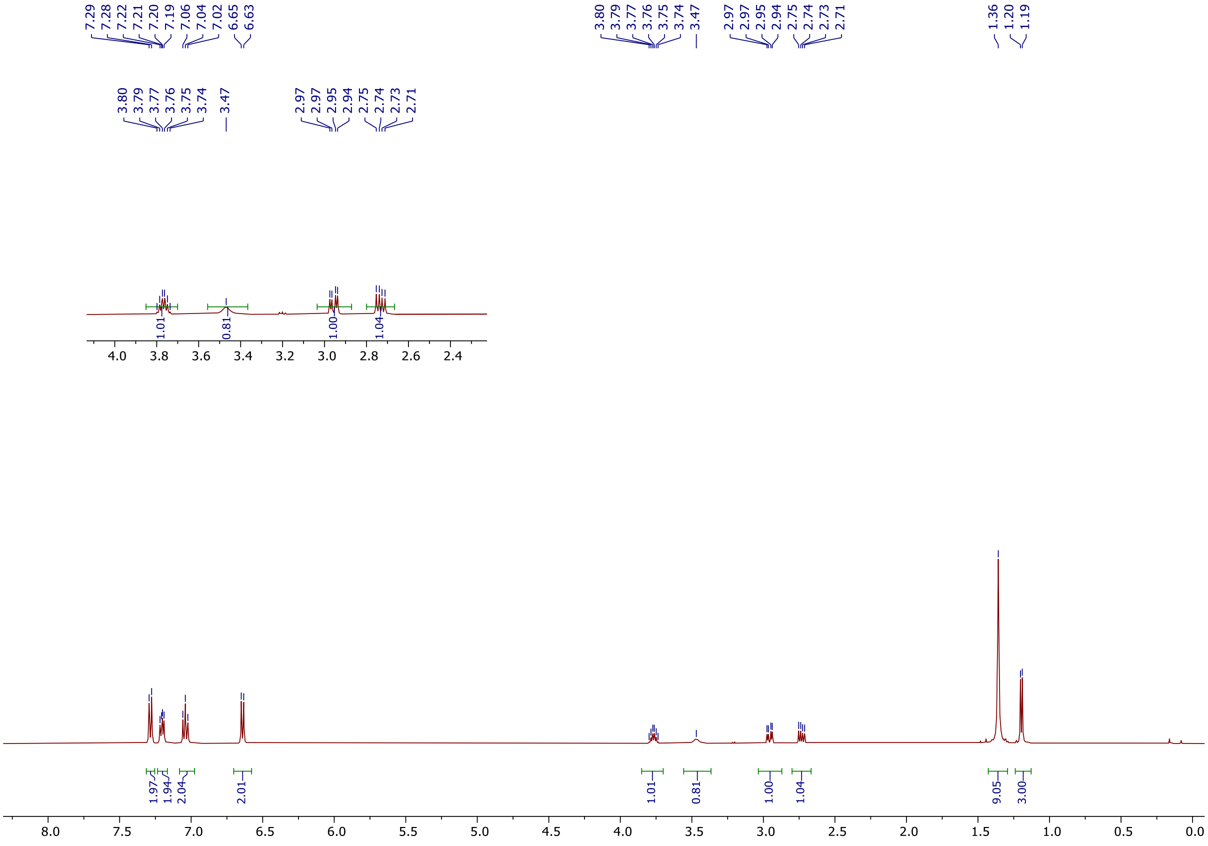

Figure S186: ^{1}H NMR Spectrum of 8k in $CDCl_3$ after isolation via column chromatography.

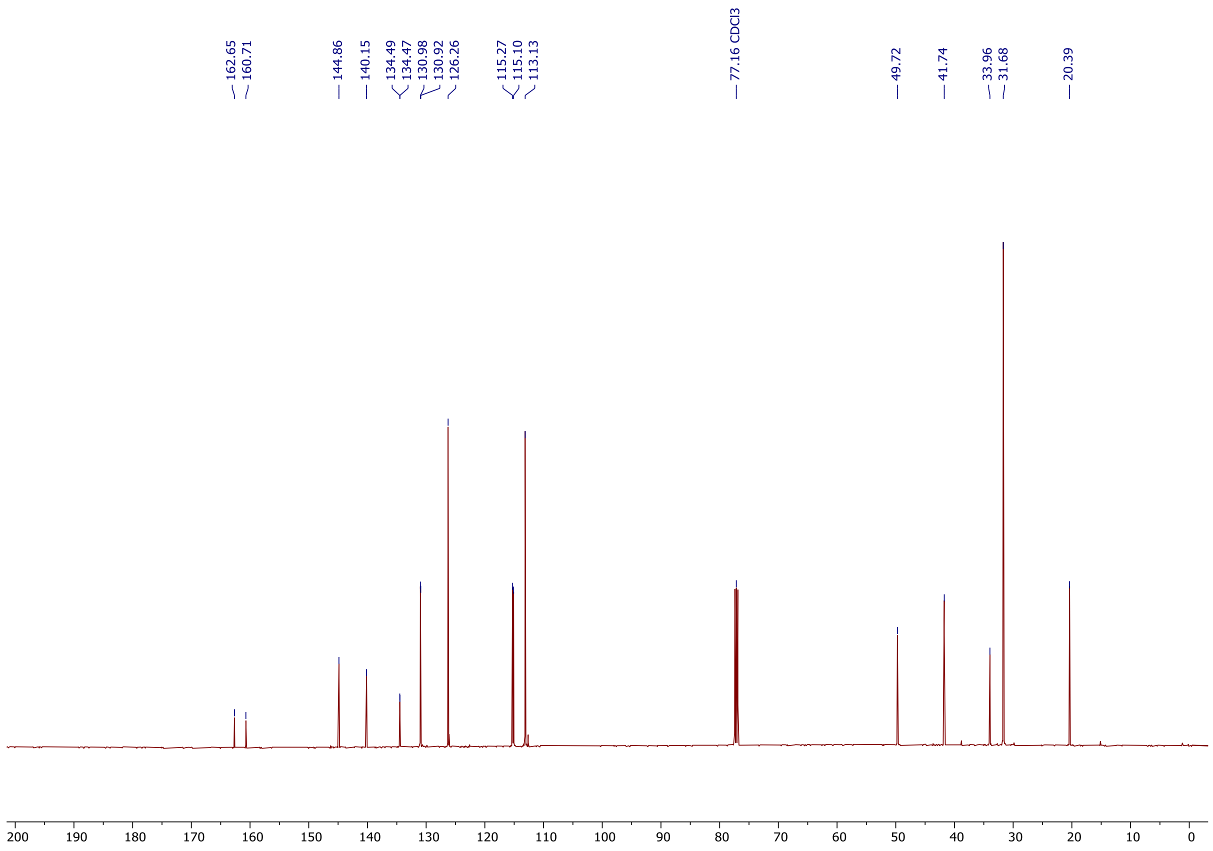

Figure S187: ^{13}C NMR Spectrum of 8k in $CDCl_3$ after isolation via column chromatography.

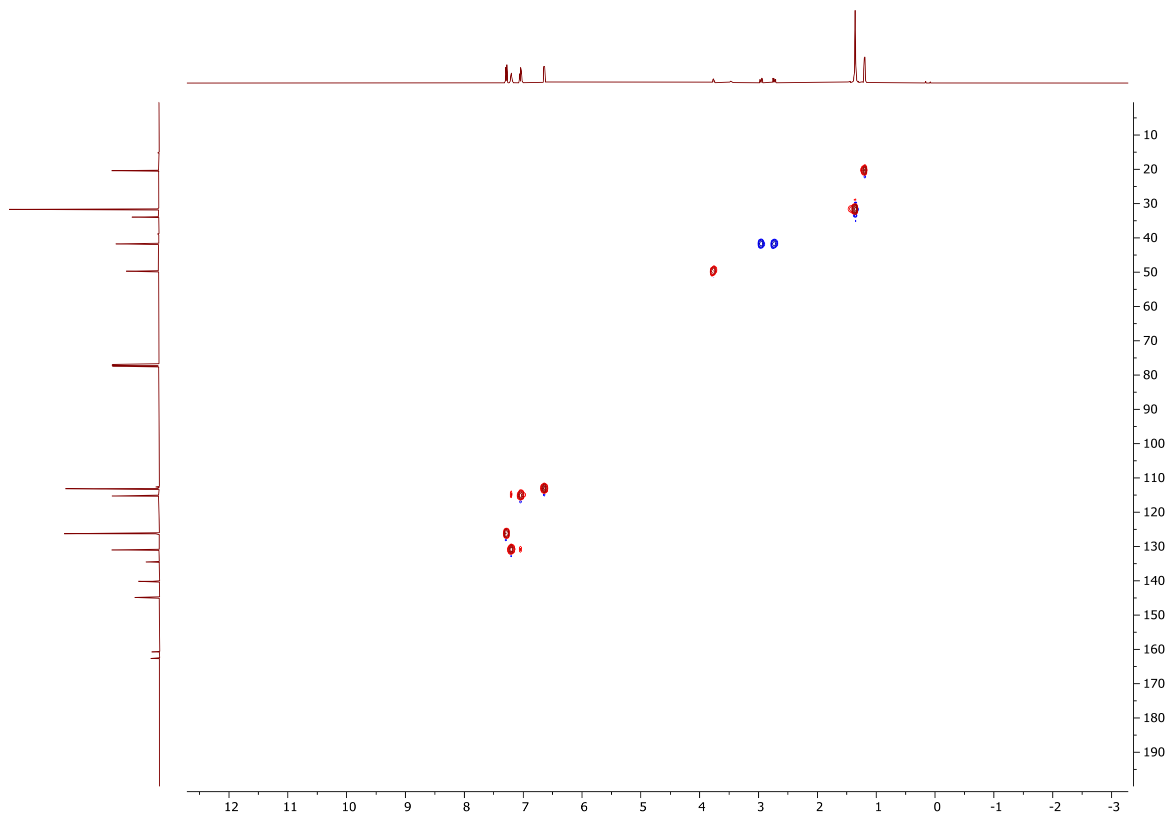

Figure S188: ^{1}H-^{13}C HSQC NMR spectrum of 8k in $CDCl_3$ after isolation via column chromatography.

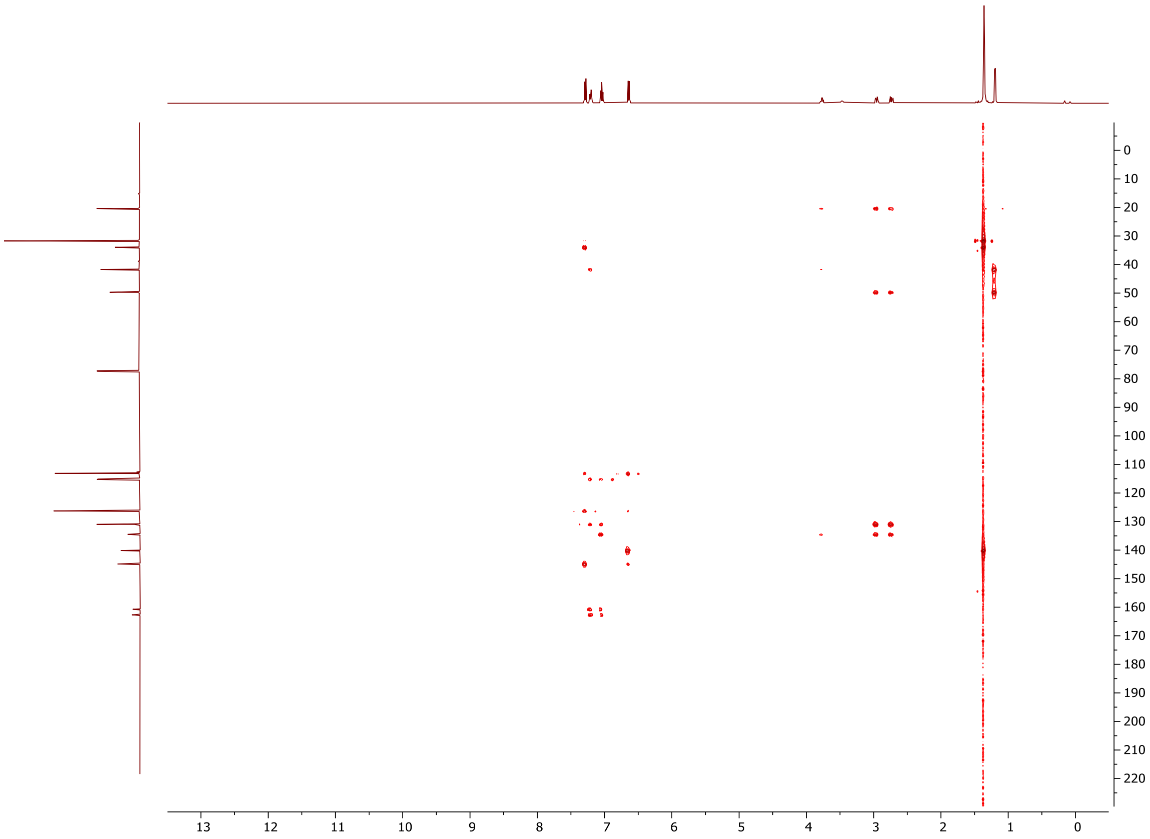

Figure S189: ¹H-¹³C HMBC NMR spectrum of 8k in $CDCl_3$ after isolation via column chromatography.

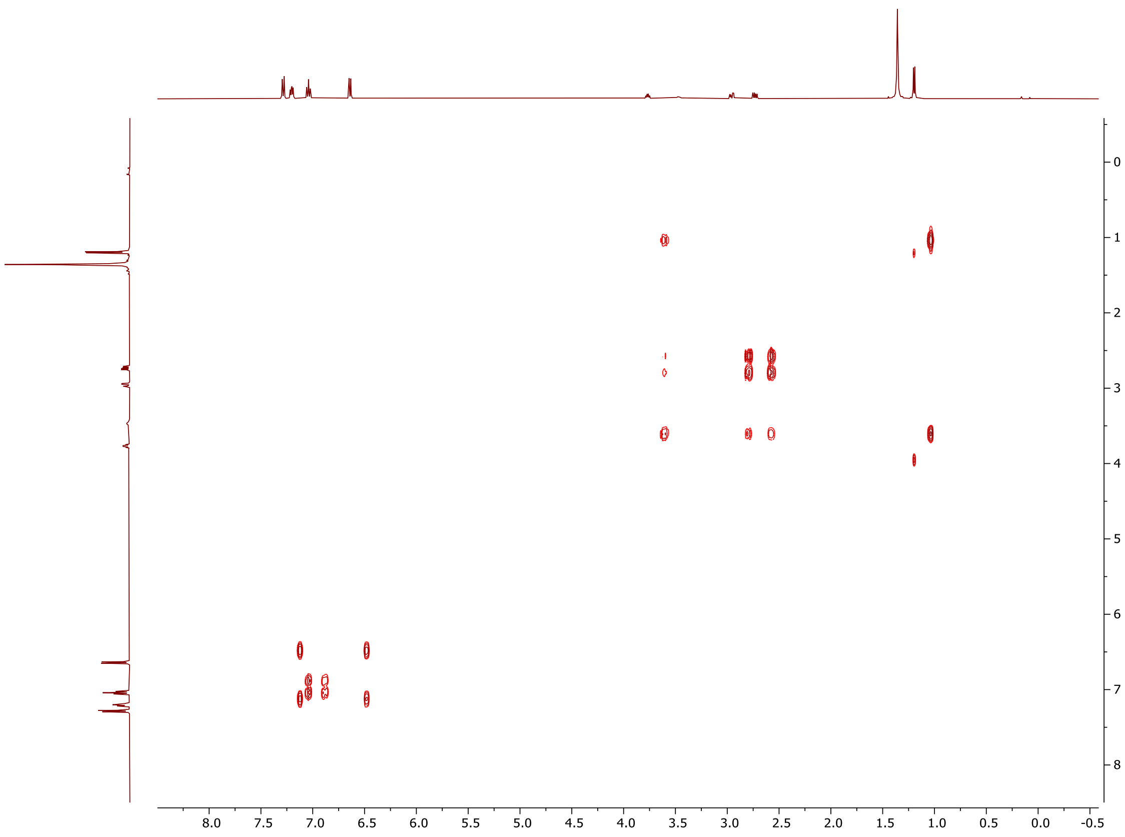

Figure S191: ^1H-^1H COSY NMR spectrum of 8k in $CDCl_3$ after isolation via column chromatography.

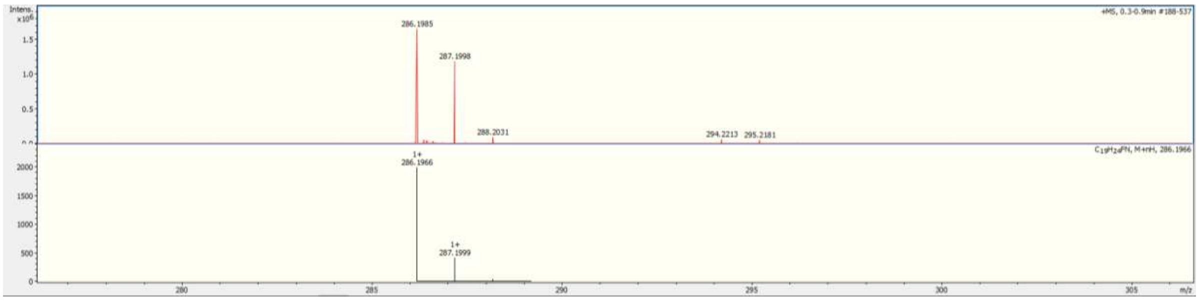

Figure S190: HRMS spectra for compound 8k.

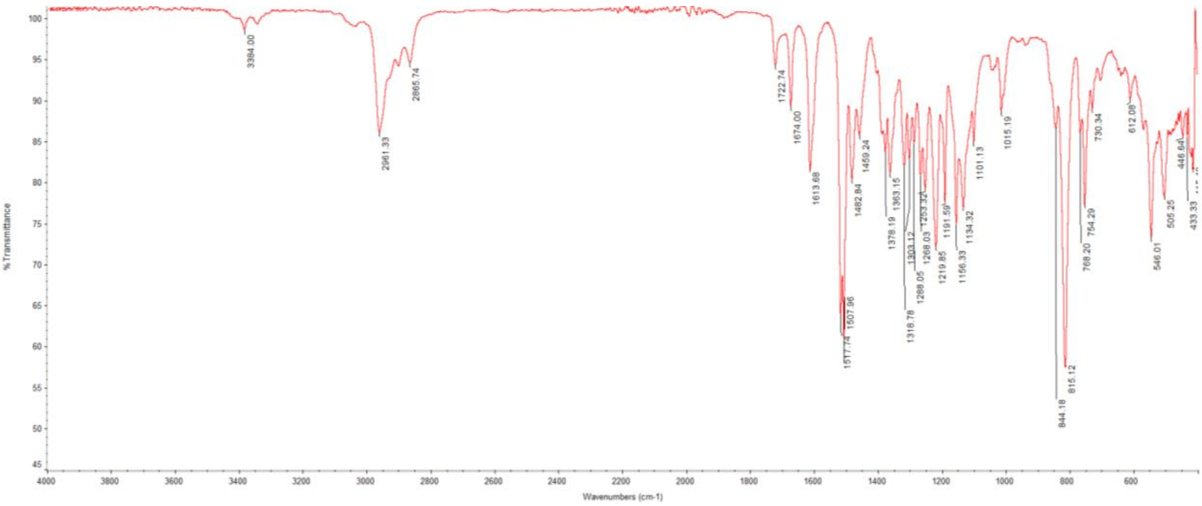

Figure S192: IR spectra for compound 8k.

11.4.12. N-(4-(tert-butyl)phenyl)-2,3-dihydrobenzo[b]thiophen-3-amine **8l**

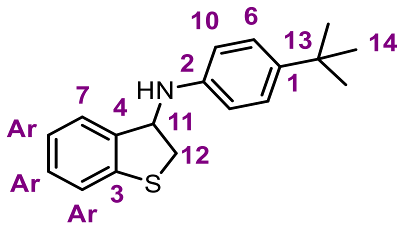

Colourless oil (54 mg, 19%)

R_f = 0.31 (30% DCM / 70% hexane)

^{1}H NMR ($CDCl_3$, 500 MHz) δ 7.38 (d, J = 7.5 Hz, 1H, C^{7}-H), 7.32 (app. d, J = 8.7 Hz, 2H, C^{6}-H), 7.33 – 7.27 (m, 2H, Ar-H), 7.15 (app. td, J = 7.5, 1.6 Hz, 1H, Ar-H), 6.73 (app. d, J = 8.6 Hz, 2H, C^{10}-H), 5.35 (app. q, J = 5.5 Hz, 1H, C^{11}-H), 4.00 (*br* s, 1H, NH), 3.71 (dd, J = 11.3, 6.7 Hz, 1H, C^{12}-H), 3.33 (dd, J = 11.3, 5.8 Hz, 1H, C^{12}-H), 1.37 (s, 9H, C^{14}-H).

$^{13}C\{^{1}H\}$ NMR ($CDCl_3$, 126 MHz) δ 144.3 (C^{2}), 141.2 (C^{1}), 141.2 (C^{3}), 140.3 (C^{4}), 129.2 (Ar), 126.4 (C^{6}), 125.3 (C^{7}), 124.8 (Ar), 122.9 (Ar), 113.3 (C^{10}), 61.1 (C^{11}), 39.2 (C^{12}), 34.1 (C^{13}), 31.7 (C^{14}).

HRMS (EI): calcd for $[M, C_{18}H_{21}NS]^{+}$ 284.1467, found 284.1465.

IR (Neat): 3390, 2957, 1614, 1517, 817, 747 cm^{-1}.

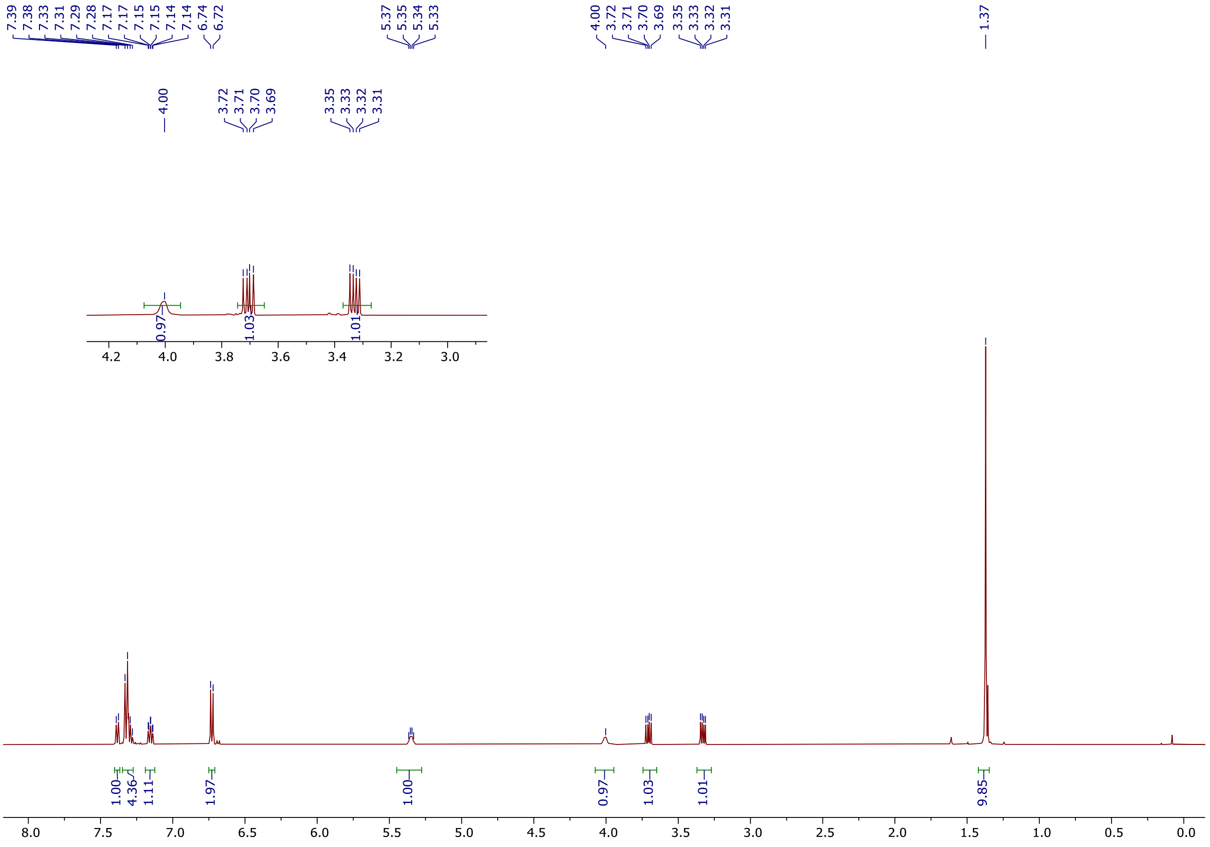

Figure S193: ^{1}H NMR Spectrum of 8l in $CDCl_3$ after isolation via column chromatography.

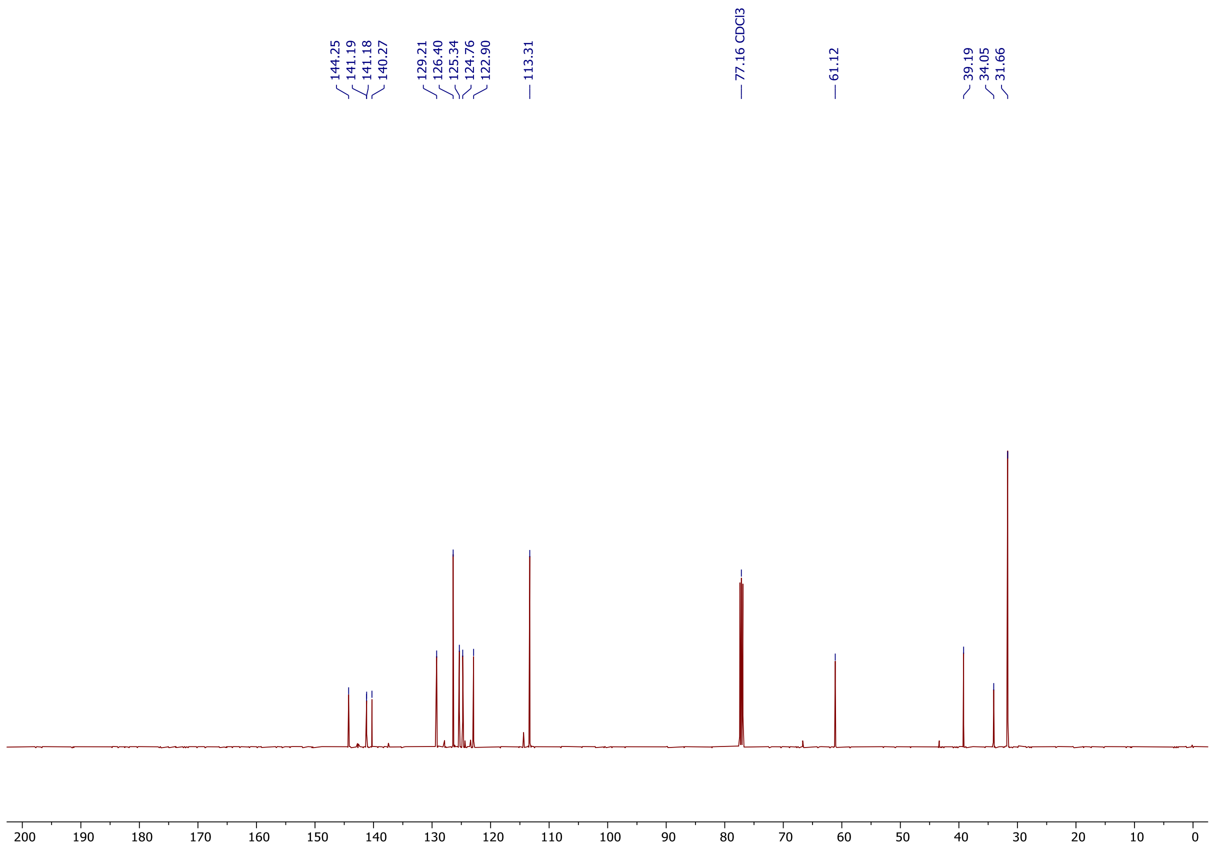

Figure S194: ^{13}C NMR Spectrum of 8l in $CDCl_3$ after isolation via column chromatography.

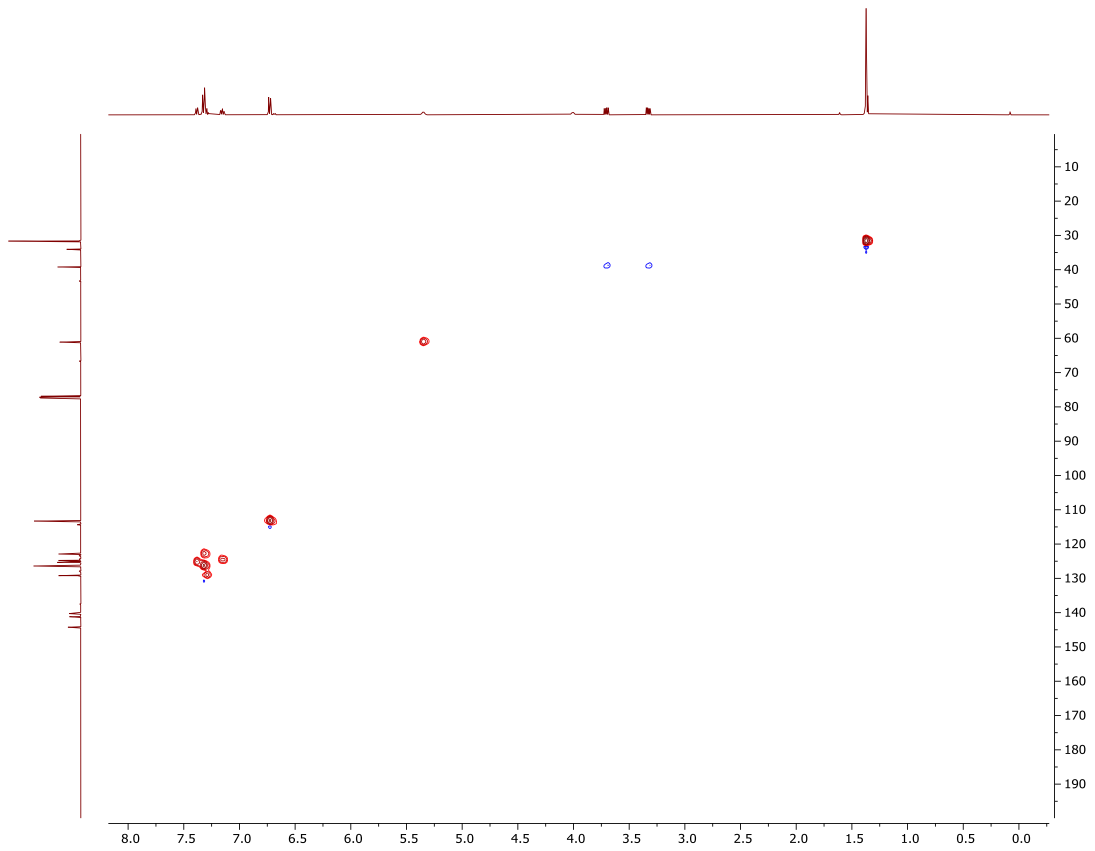

Figure S195: ^{1}H-^{13}C *HSQC* NMR Spectrum of 8l in $CDCl_3$ after isolation via column chromatography.

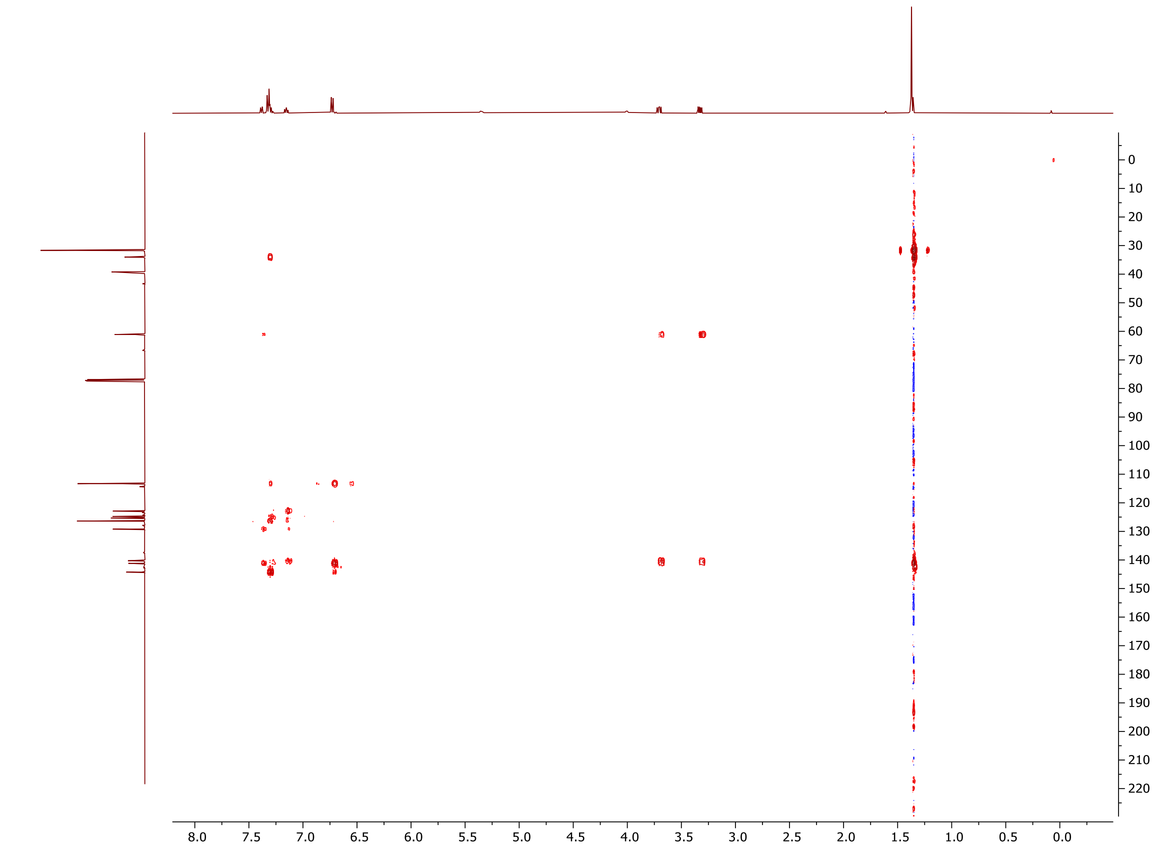

Figure S196: ^{1}H-^{13}C HMBC NMR Spectrum of 8l in $CDCl_3$ after isolation via column chromatography.

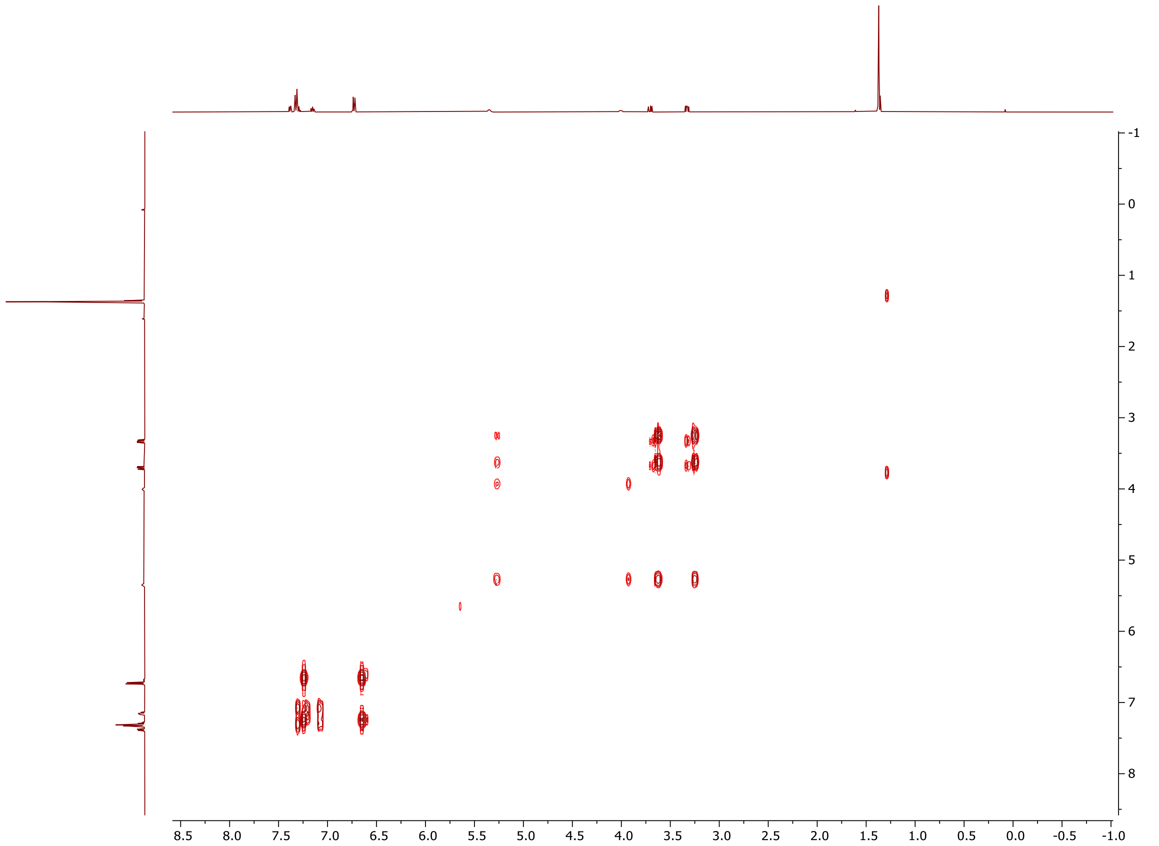

Figure S197: ^{1}H-^{1}H COSY NMR Spectrum of 8l in $CDCl_3$ after isolation via column chromatography.

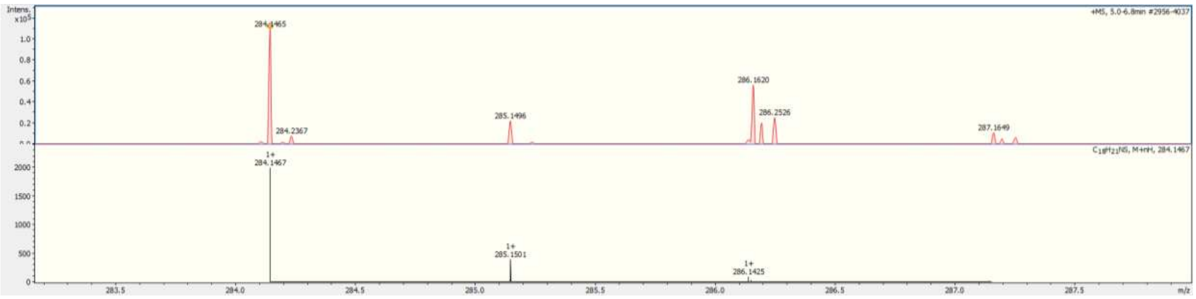

Figure S198: HRMS spectra for compound 8l.

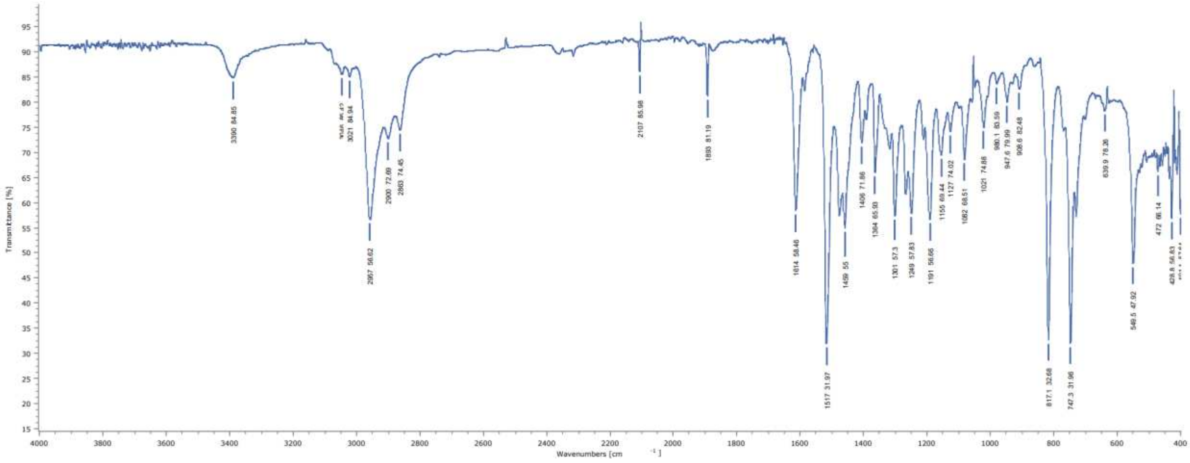

Figure S199: IR spectra for compound 8l.

11.4.13. tert-butyl 3-((4-(tert-butyl)phenyl)amino)indoline-1-carboxylate **8m**

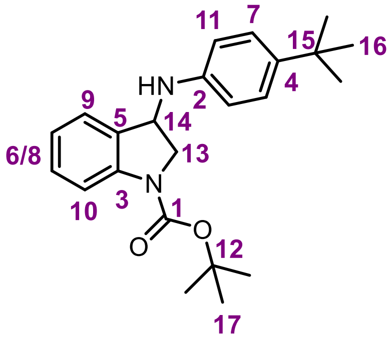

red oil (74 mg, 20%)

R_f = 0.27 (50% DCM / 50% hexane)

^{1}H NMR (CDCl$_3$, 500 MHz) δ 7.94 (app. *br* s, 1H, C^{10}-H), 7.32 (app. t, J = 6.8 Hz, 2H, $C^{6/8}$-H), 7.27 (d, J = 8.7 Hz, 2H, C^7-H), 7.01 (td, J = 7.5, 1.0 Hz, 1H, C^9-H), 6.62 (d, J = 8.7 Hz, 2H, C^{11}-H), 5.05 (dd, J = 8.1, 4.1 Hz, 1H, C^{14}-H), 4.21 (dd, J = 11.8, 8.1 Hz, 1H, C^{13}-H), 3.89 (app. *br* s, 1H, C^{13}-H), 3.86 (br s, 1H, NH), 1.56 (s, 9H, C^{17}-H), 1.32 (s, 9H, C^{16}-H).

$^{13}C\{^1H\}$ NMR ($CDCl_3$, 126 MHz) δ 152.5 (C^1), 144.2 (C^2), 143.1 (C^3), 141.1 (C^4), 131.4 (C^5), 129.7 (C^6), 126.4 (C^7), 125.4 (C^8), 122.7 (C^9), 115.2 (C^{10}), 112.9 (C^{11}), 81.1 (C^{12}), 55.7 (C^{13}), 53.1 (C^{14}), 34.1 (C^{15}), 31.7 (C^{16}), 28.6 (C^{17}).

HRMS (ESI+): calcd for [M, $C_{23}H_{31}N_2O_2$]$^+$ 367.2383, found 367.2380.

IR (Neat): 3380, 2965, 2507, 1722, 1673, 1518, 1389 cm^{-1}.

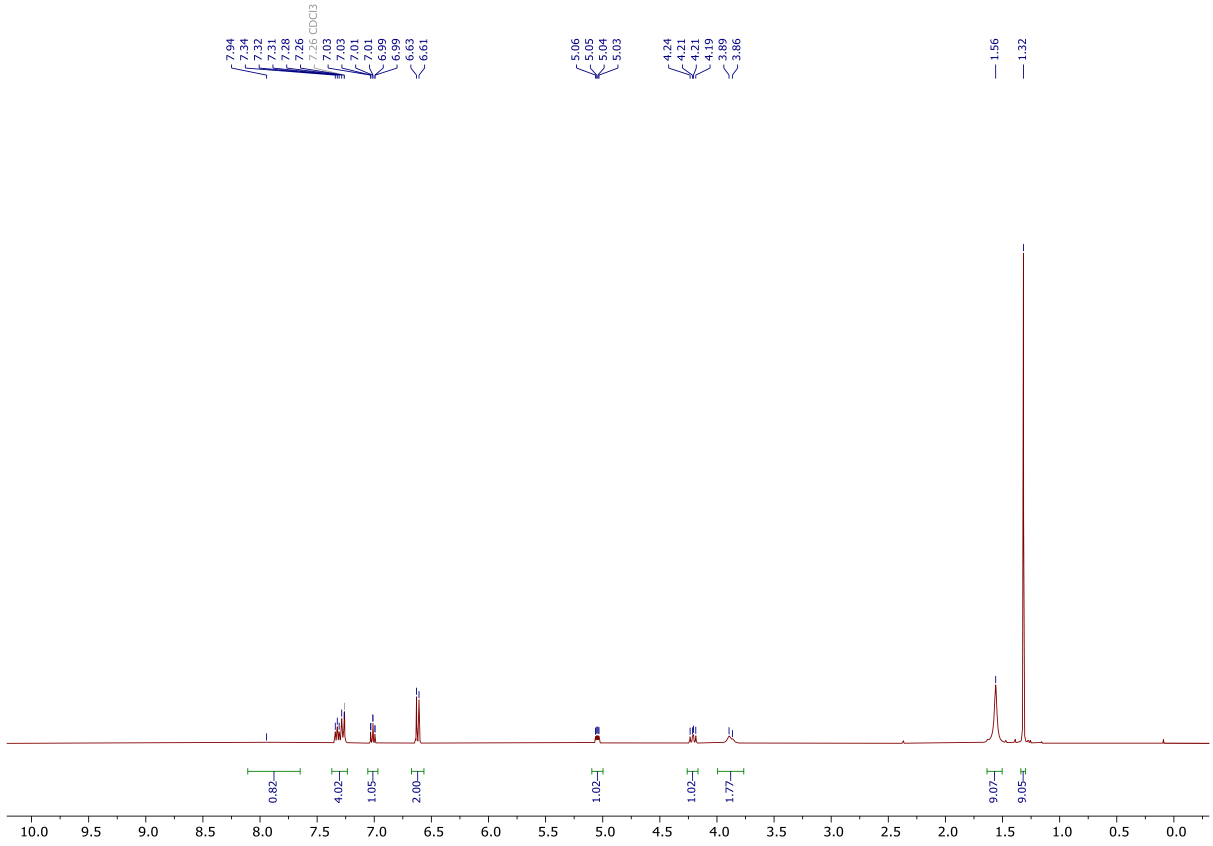

Figure S200: ^{1}H NMR Spectrum of 8m in $CDCl_3$ after isolation via column chromatography.

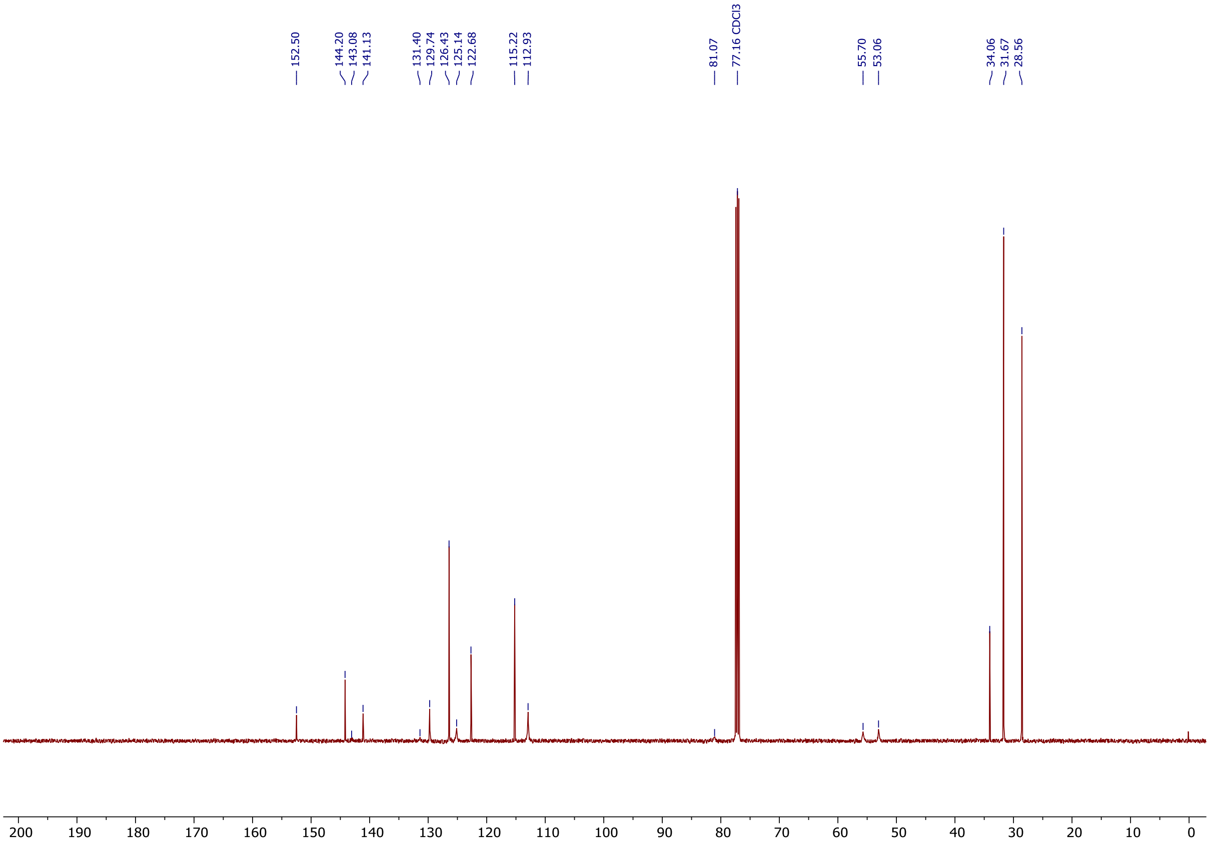

Figure S201: ^{13}C NMR Spectrum of 8m in $CDCl_3$ after isolation via column chromatography.

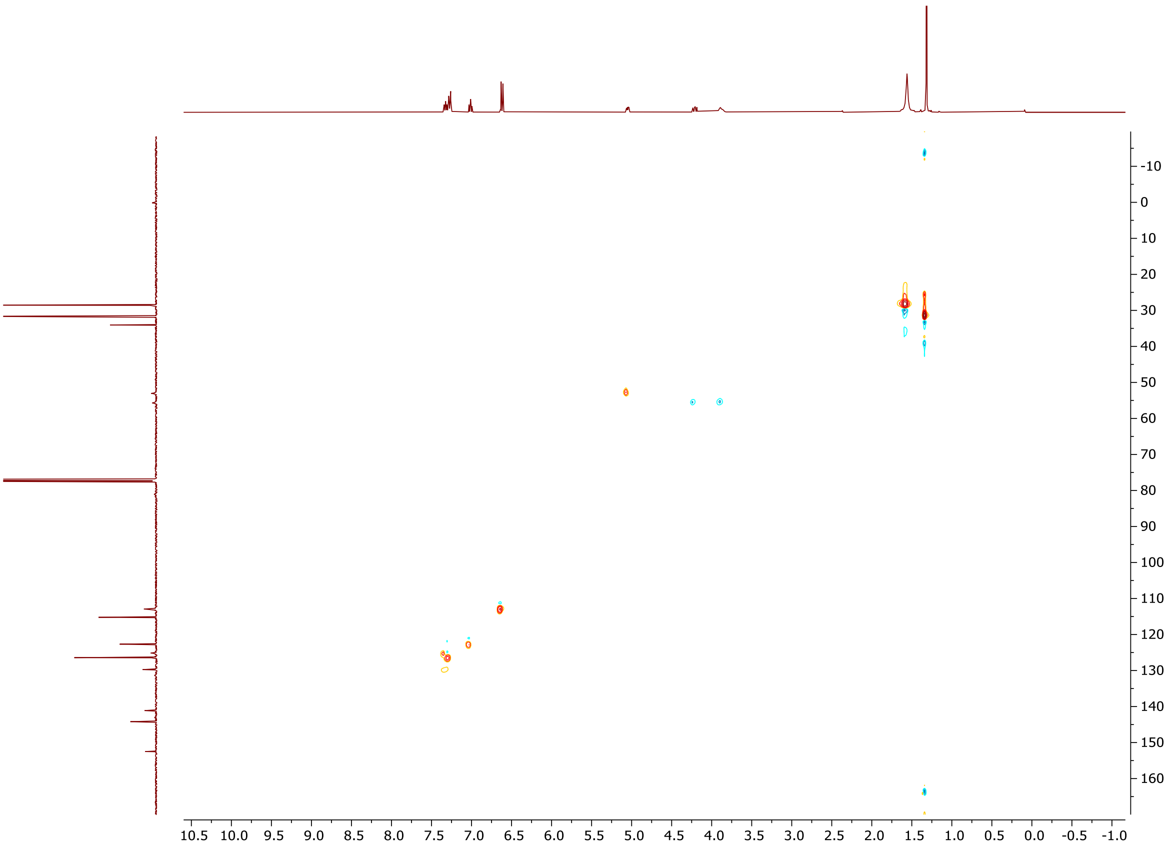

Figure S202: ^{1}*H-*^{13}*C HSQC* NMR Spectrum of 8m in $CDCl_3$ after isolation via column chromatography.

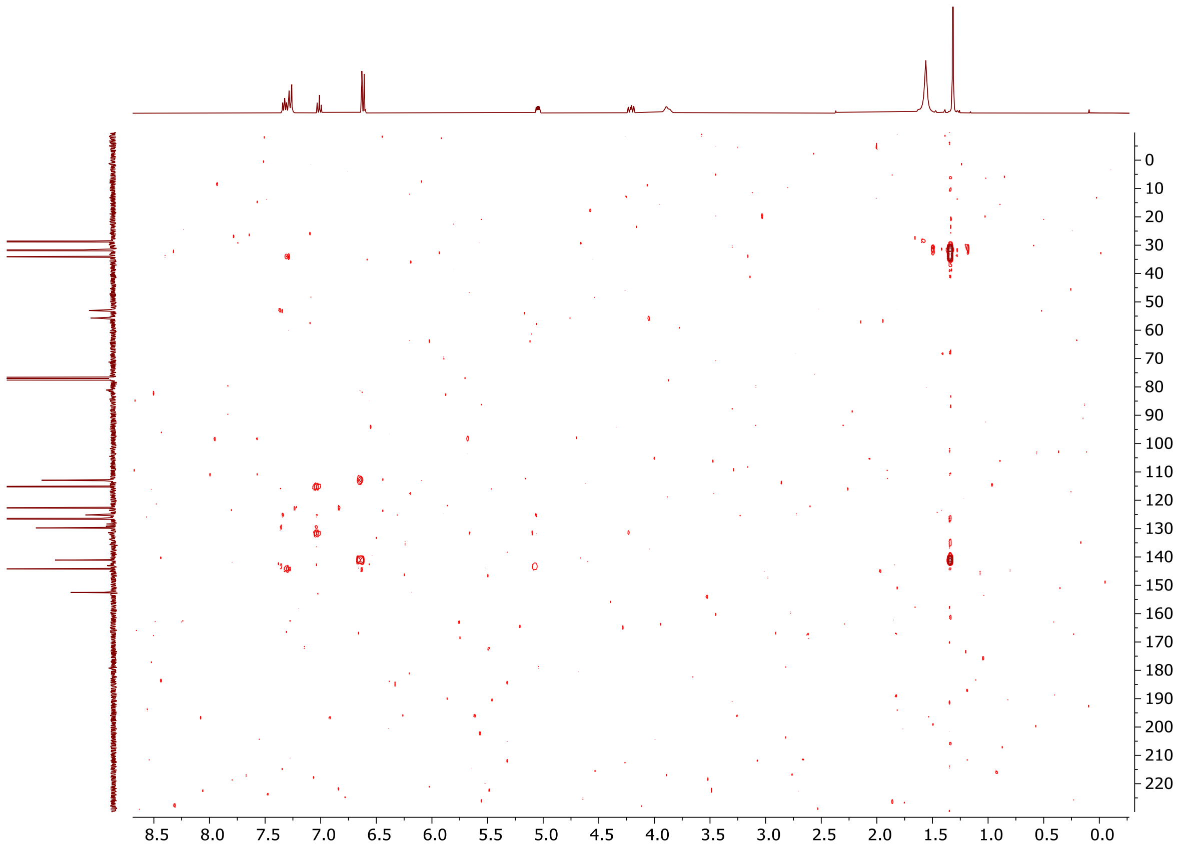

Figure S203: ^{1}H-^{13}C HMBC NMR Spectrum of 8m in $CDCl_3$ after isolation via column chromatography.

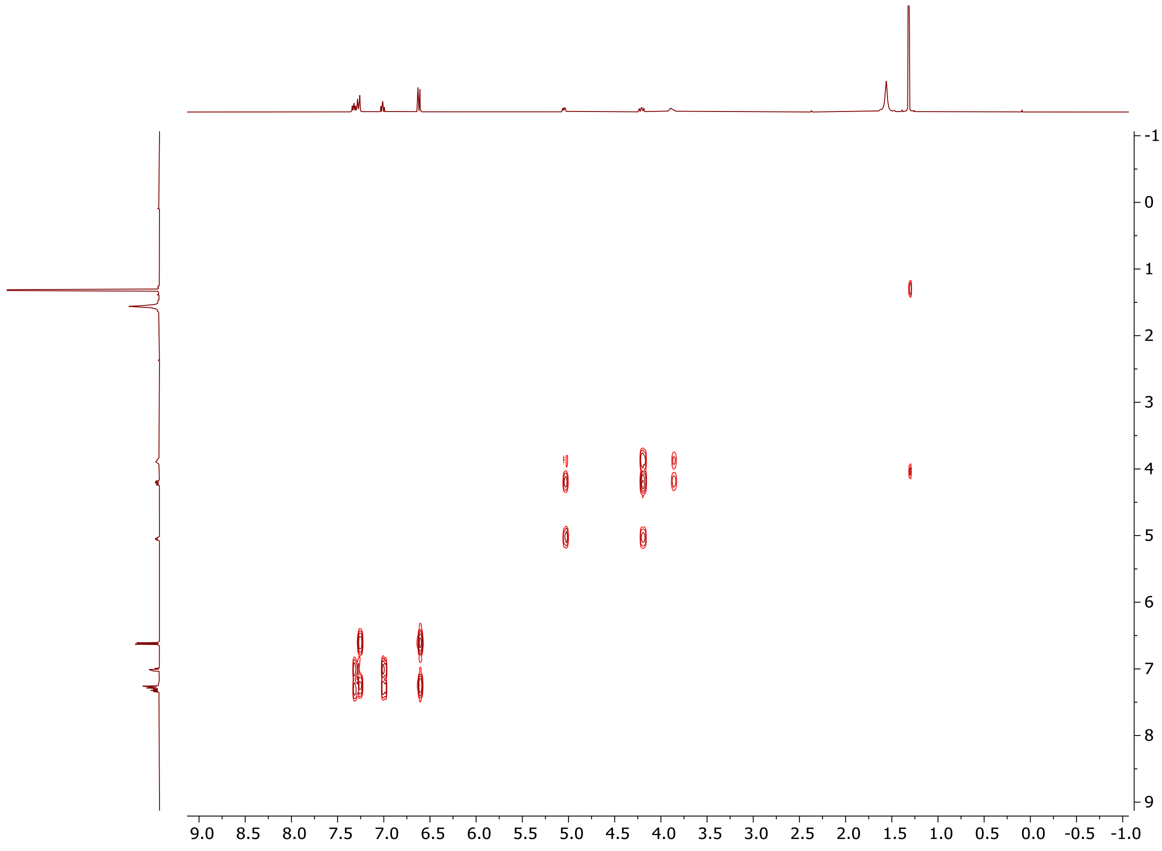

Figure S204: ^{1}H-^{1}H COSY NMR Spectrum of 8m in $CDCl_3$ after isolation via column chromatography.

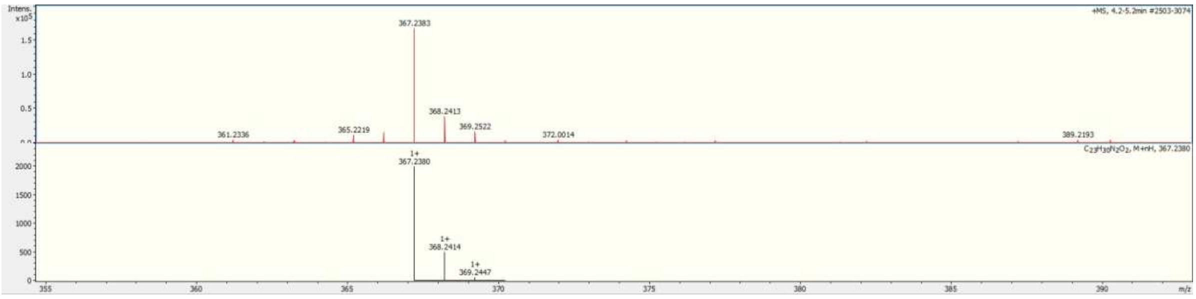

Figure S205: HRMS spectra for compound 8m.

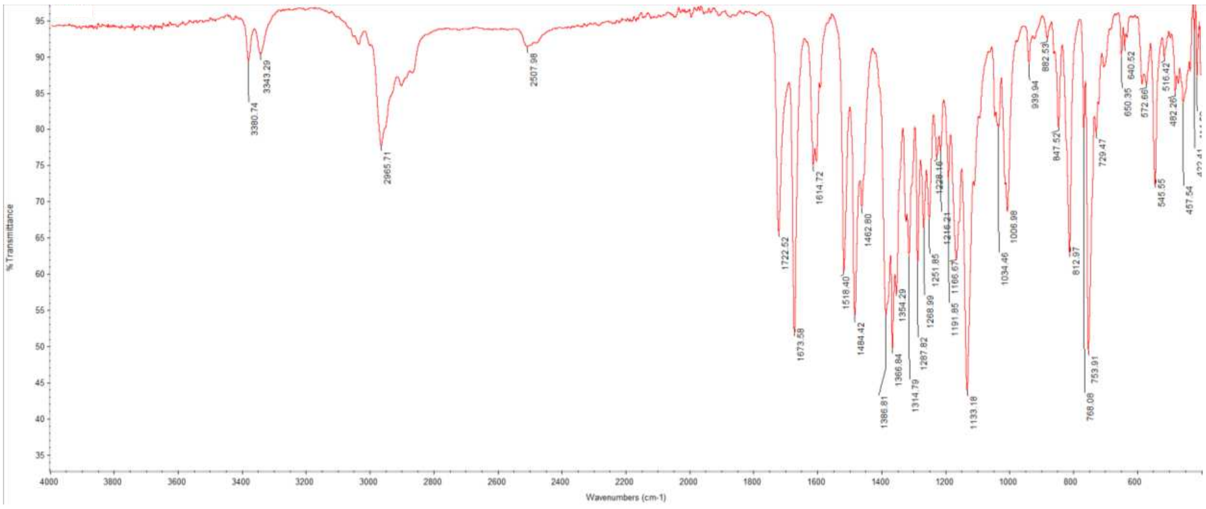

Figure S206: IR spectra for compound 8m.

11.4.14. N-phenyl-2,3-dihydro-1H-inden-1-amine **8o**[17]

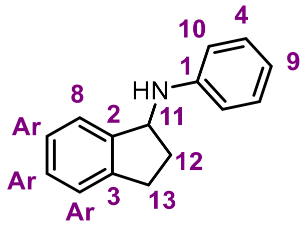

Required an additional zinc reduction step.

Light yellow oil (161 mg, 77%)

R_f = 0.26 (30% DCM / 70% hexane)

Analytic data is in accordance with those reported in literature.

^{1}H NMR ($CDCl_3$, 500 MHz) δ 7.44 (d, J = 7.4 Hz, 1H, C^8-H), 7.34 – 7.25 (m, 3H, Ar-H), 7.27 (app. td, J = 7.4, 2.1 Hz, 2H, C^4-H), 6.81 – 6.77 (m, 3H, C^9, C^{10}-H), 5.08 (app. t, J = 6.7 Hz, 1H, C^{11}-H), 3.94 (*br* s, 1H, NH), 3.08 (ddd, J = 15.9, 8.6, 4.3 Hz, 1H, C^{13}-H), 2.96 (app. dt, J = 15.9, 7.9 Hz, 1H, C^{13}-H), 2.70 – 2.62 (m, 1H, C^{12}-H), 1.99 – 1.94 (m, 1H, C^{12}-H).

^{13}C{^{1}H} NMR ($CDCl_3$, 126 MHz) δ 147.9 (C^1), 144.7 (C^2), 143.7 (C^3), 129.5 (C^4), 129.5 (Ar), 128.0 (Ar), 126.8 (Ar), 125.0 (C^8), 124.4 (C^9), 113.3 (C^{10}), 58.7 (C^{11}), 34.0 (C^{12}), 30.4 (C^{13}).

HRMS (ESI+): calcd for [M, $C_{15}H_{15}N$]$^+$ 210.1277, found 210.1290.

IR (Neat): 3410, 2960, 1601, 1501, 746, 693 cm^{-1}.

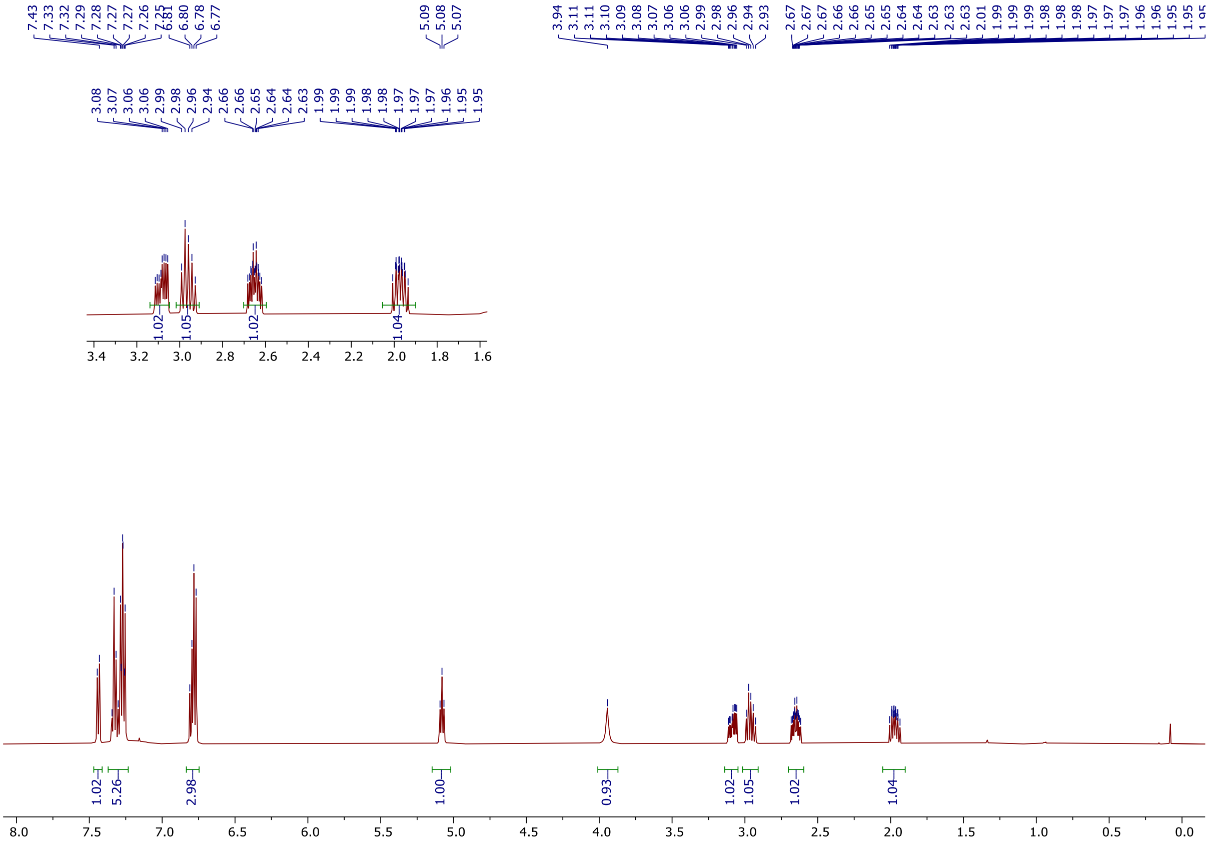

Figure S207: ^{1}H NMR Spectrum of 8o in $CDCl_3$ after isolation via column chromatography.

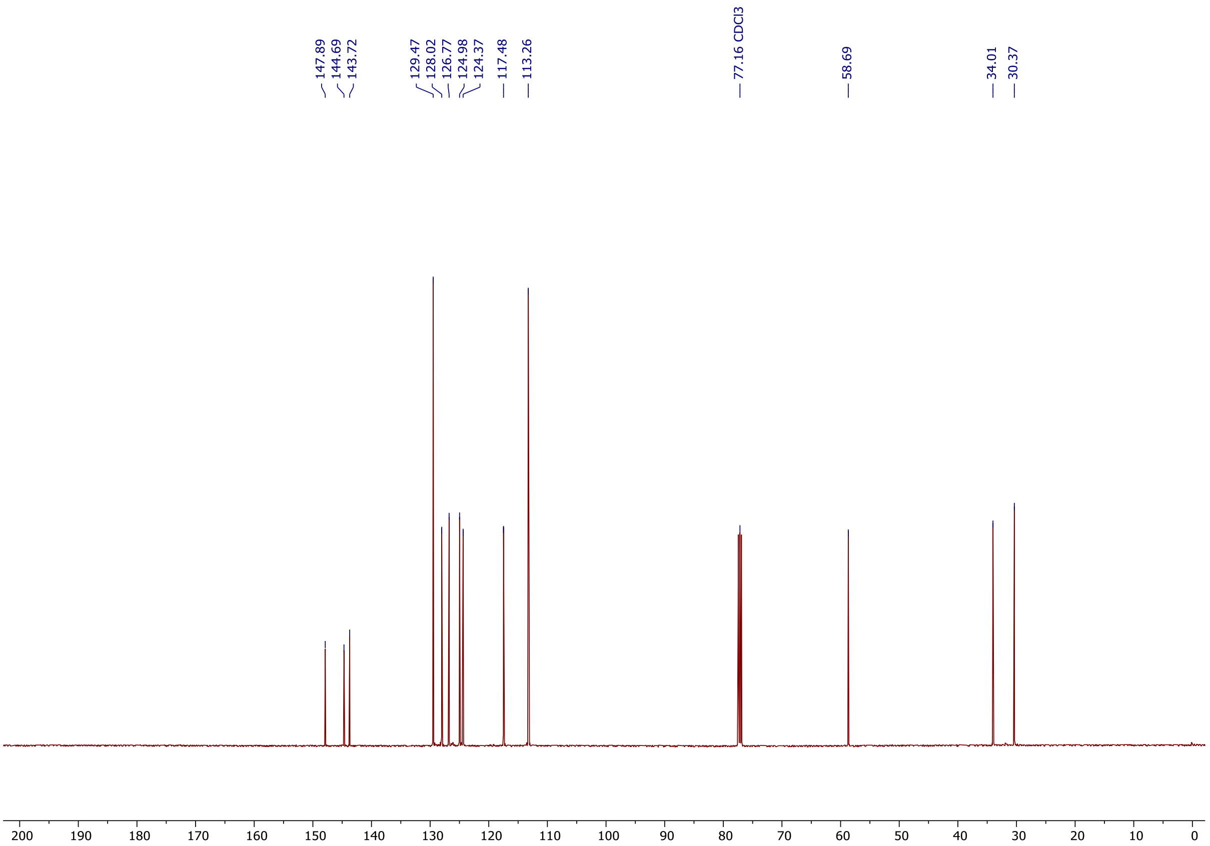

Figure S208: ^{13}C NMR Spectrum of 8o in $CDCl_3$ after isolation via column chromatography.

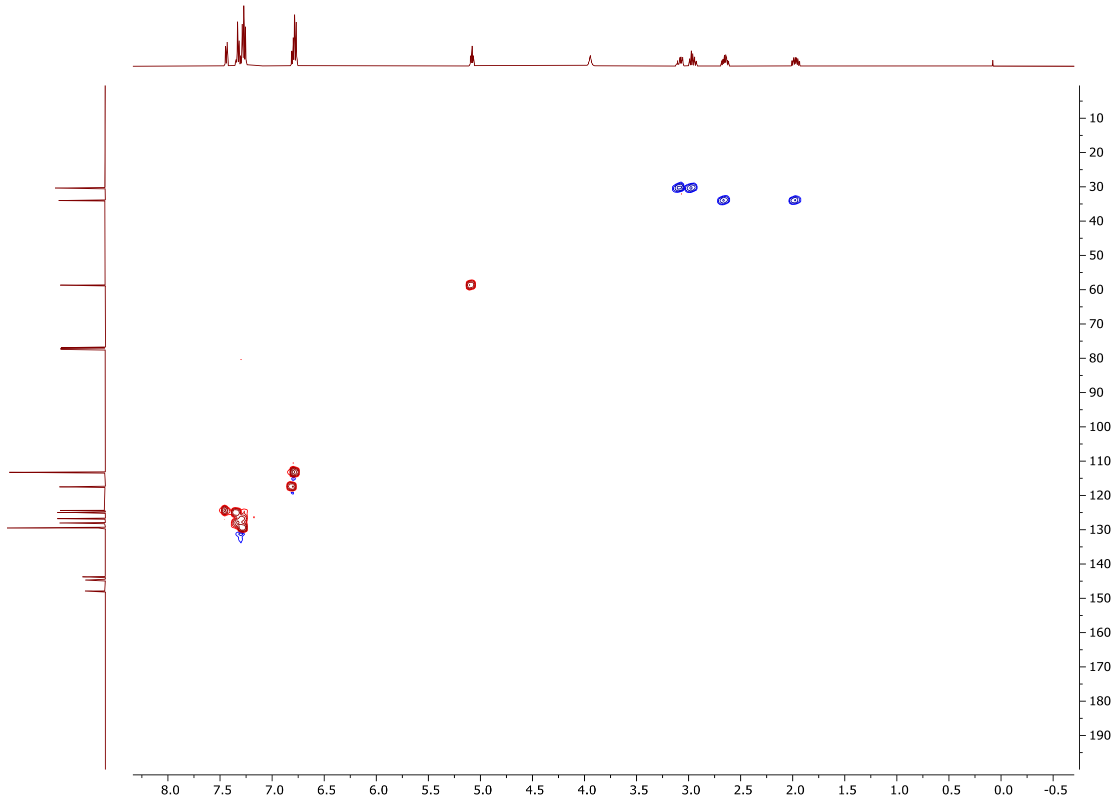

Figure S209: ^{1}H-^{13}C HSQC NMR Spectrum of 8o in $CDCl_3$ after isolation via column chromatography.

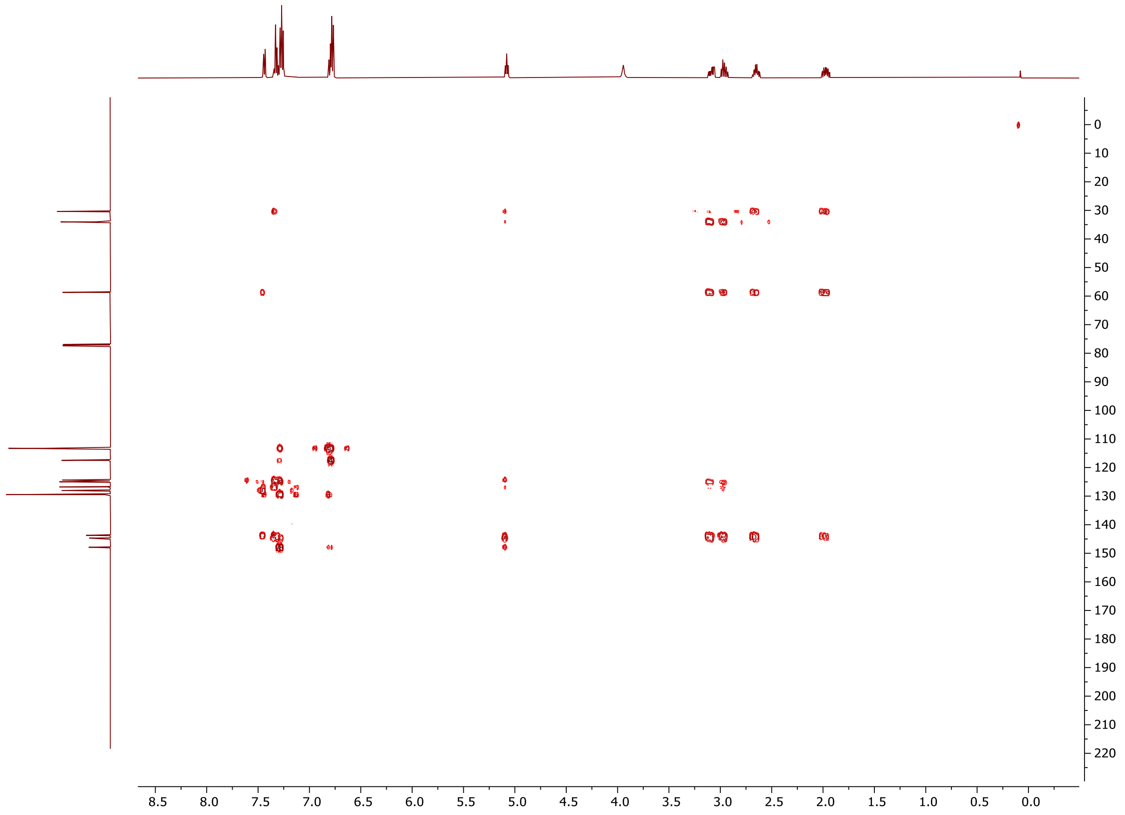

Figure S210: ^{1}H-^{13}C HMBC NMR Spectrum of 8o in $CDCl_3$ after isolation via column chromatography.

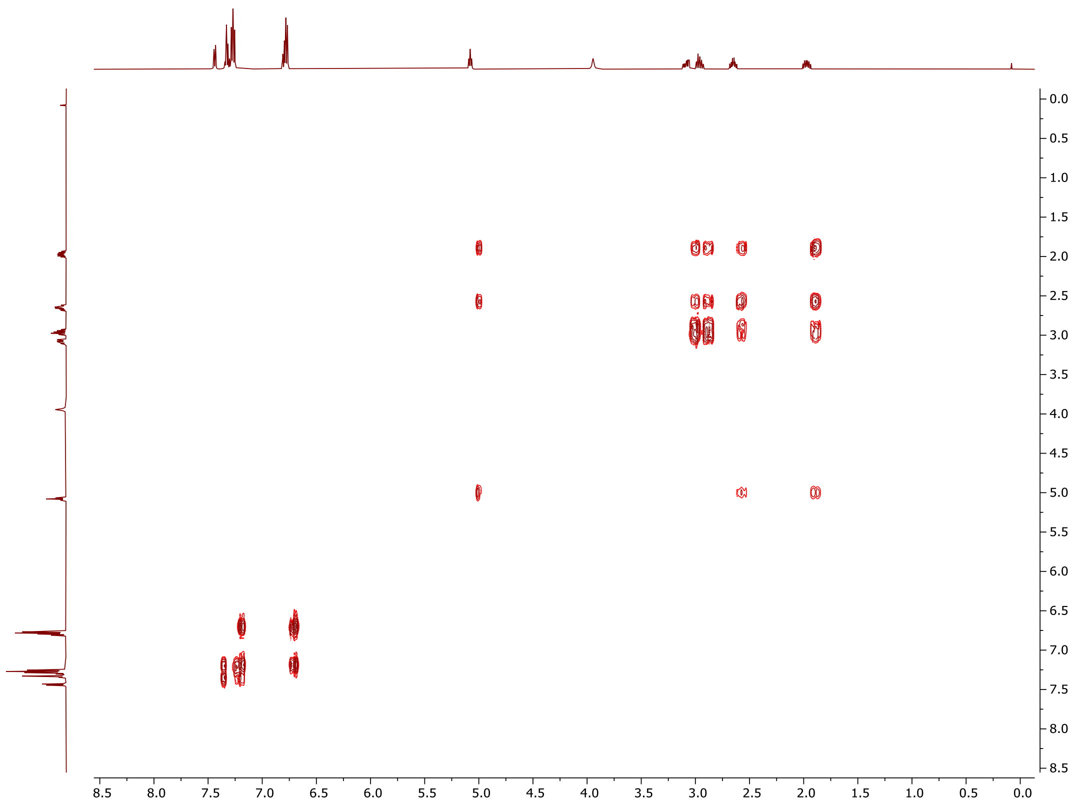

Figure S211: ^1H-^1H COSY NMR Spectrum of 8o in $CDCl_3$ after isolation via column chromatography.

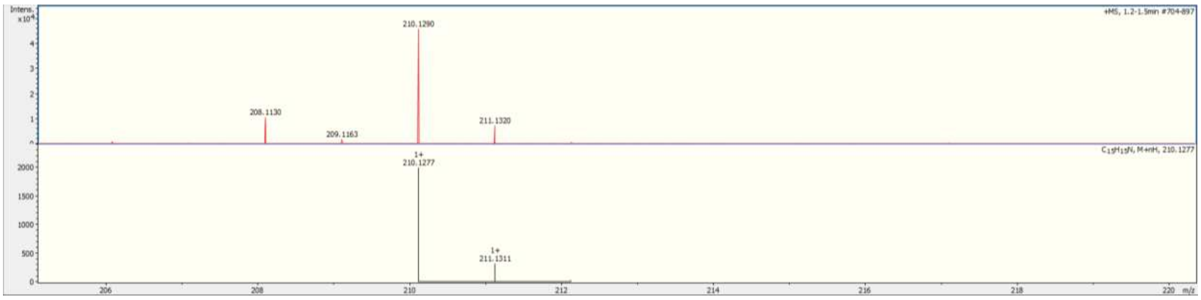

Figure S212: HRMS spectra for compound 8o.

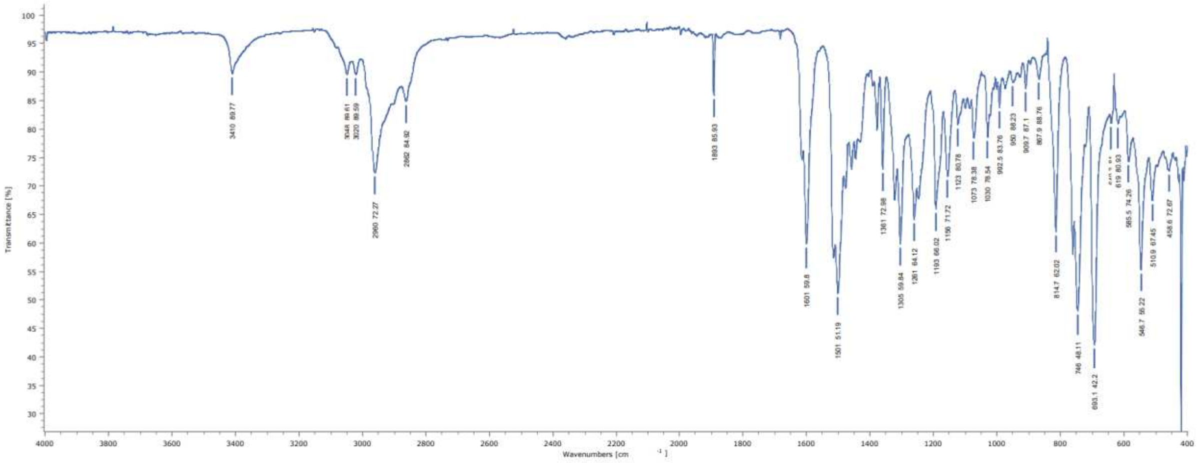

Figure S213: IR spectra for compound 8o.

11.4.15. N-(1-(2-methoxyphenyl)propan-2-yl)aniline **8p**

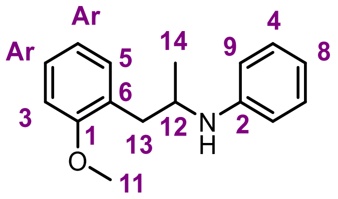

Light yellow oil (76 mg, 31%)

R_f = 0.15 (30% DCM / 70% hexane)

^{1}H NMR ($CDCl_3$, 500 MHz) δ 7.24 – 7.14 (m, 4H Ar-H,C^5-H), 7.19 (app. t, J = 7.6 Hz, 2H, C^4-H), 6.92 (dd, J = 7.5, 1.0 Hz, 1H, Ar-H), 6.90 (app. t, J = 7.6 Hz, 1H, C^3-H), 6.69 – 6.67 (m, 3H, C^8-H, C^9-H), 3.89 (s, 3H, C^{11}-H), 3.85 – 3.77 (m, 1H, C^{12}-H), 3.16 (dd, J = 13.3, 5.6 Hz, 1H, C^{13}-H), 2.56 (dd, J = 13.3, 7.3 Hz, 1H, C^{13}-H), 1.19 (d, J = 6.3 Hz, 3H, C^{14}-H).

$^{13}C\{^1H\}$ NMR ($CDCl_3$, 126 MHz) δ 157.8 (C^1), 147.8 (C^2), 131.4 (Ar), 129.3 (C^4), 127.7 (C^5), 127.7 (C^6), 120.6 (Ar), 116.8 (C^8), 113.2 (C^9), 110.5 (C^3), 55.3 (C^{11}), 48.9 (C^{12}), 37.7 (C^{13}), 20.7 (C^{14}).

HRMS (ESI+): calcd for [M, $C_{16}H_{19}NO$]$^+$ 242.1539, found 242.1556.

IR (Neat): 3047, 3050, 2961, 1600, 1492, 1240, 746 cm^{-1}.

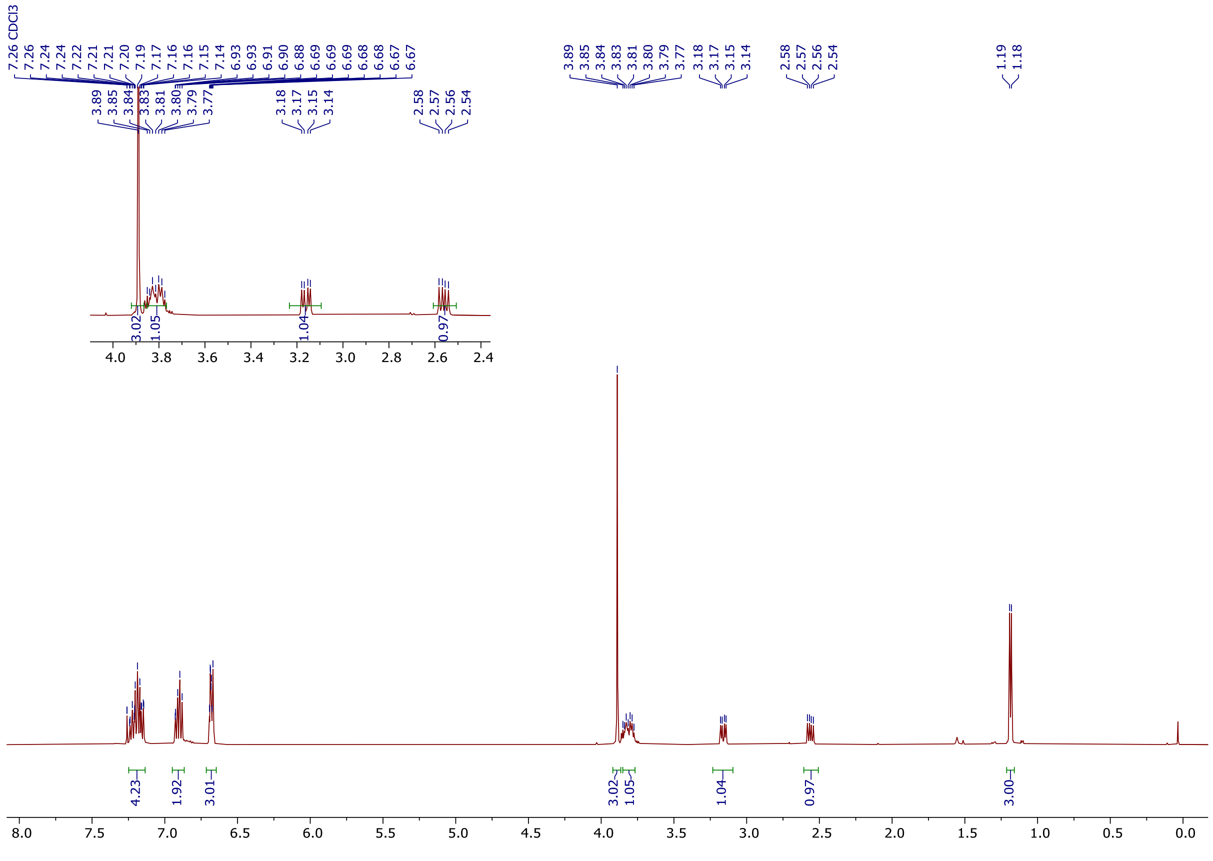

Figure S214: ^{1}H NMR Spectrum of 8p in $CDCl_3$ after isolation via column chromatography.

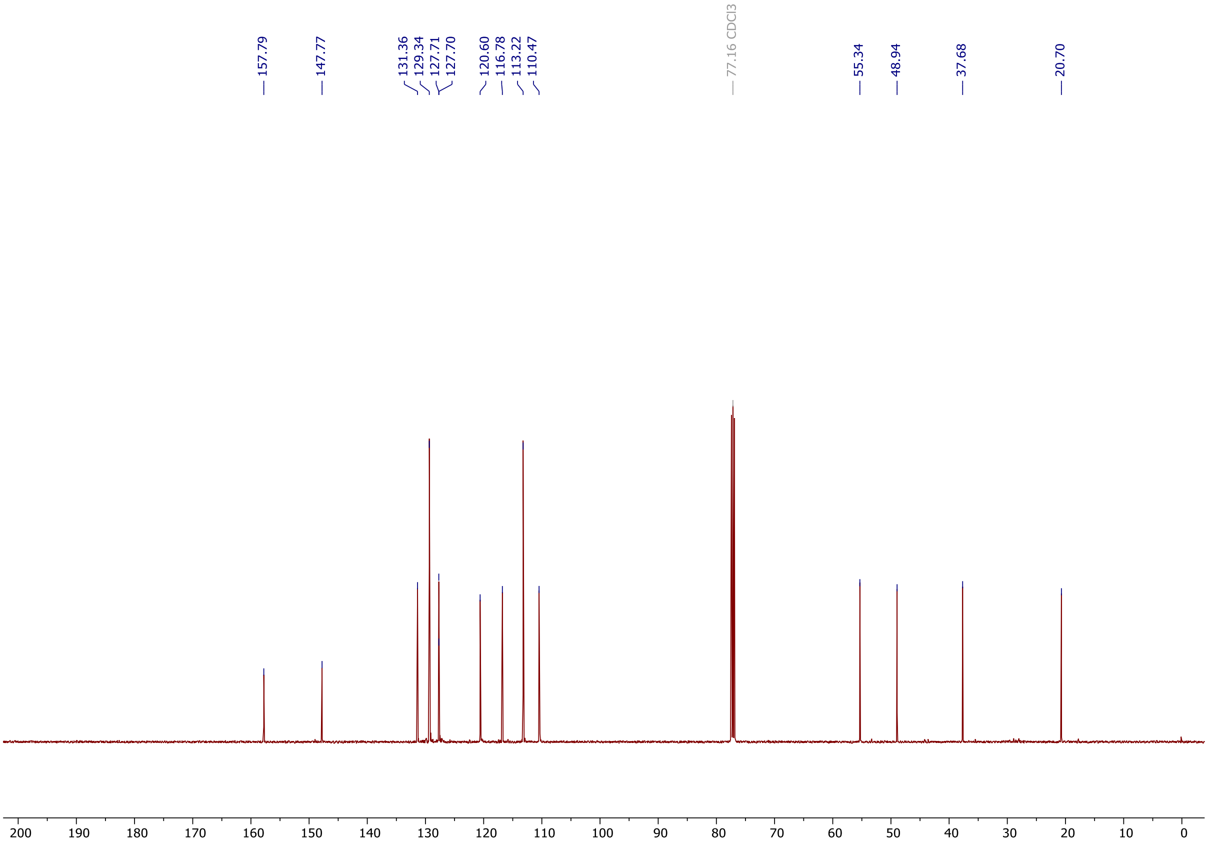

Figure S 215: ^{13}C NMR Spectrum of 8p in $CDCl_3$ after isolation via column chromatography.

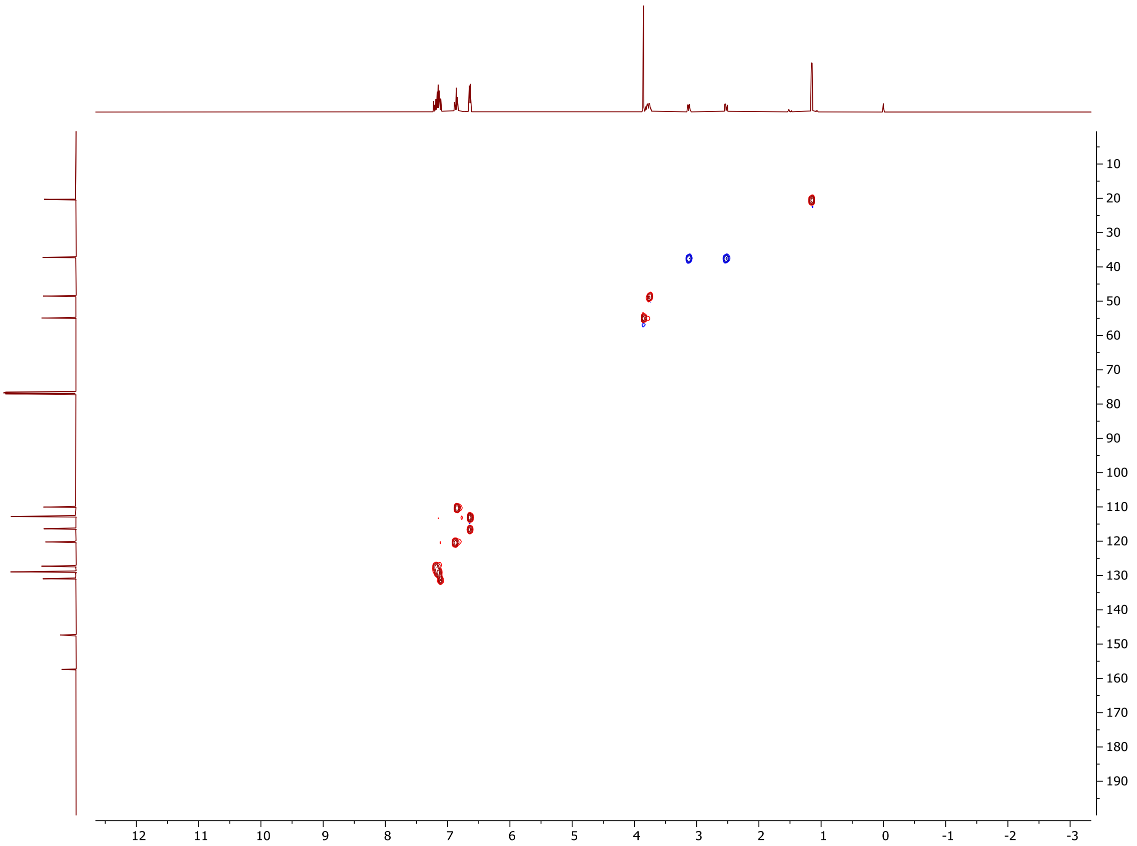

Figure S216: ^{1}H-^{13}C HSQC NMR Spectrum of 8p in $CDCl_3$ after isolation via column chromatography.

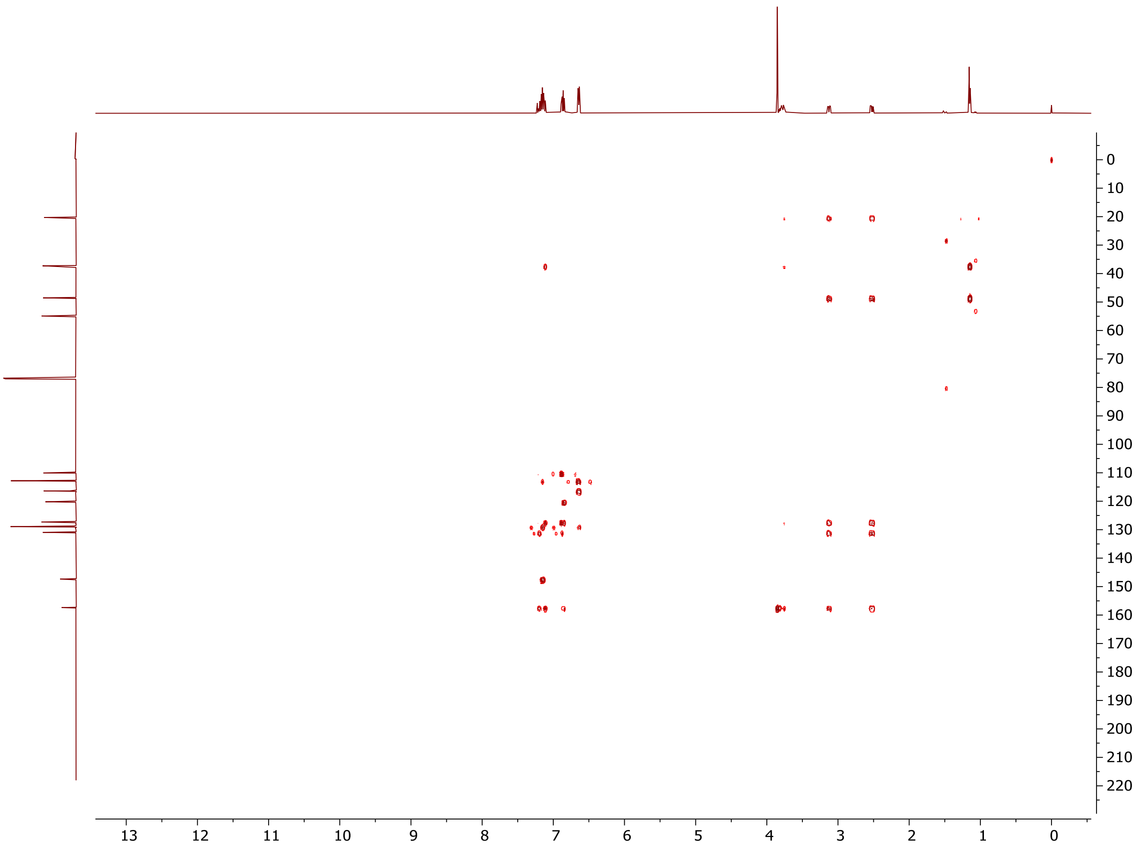

Figure S217: ^{1}H-^{13}C HMBC NMR Spectrum of 8p in $CDCl_3$ after isolation via column chromatography.

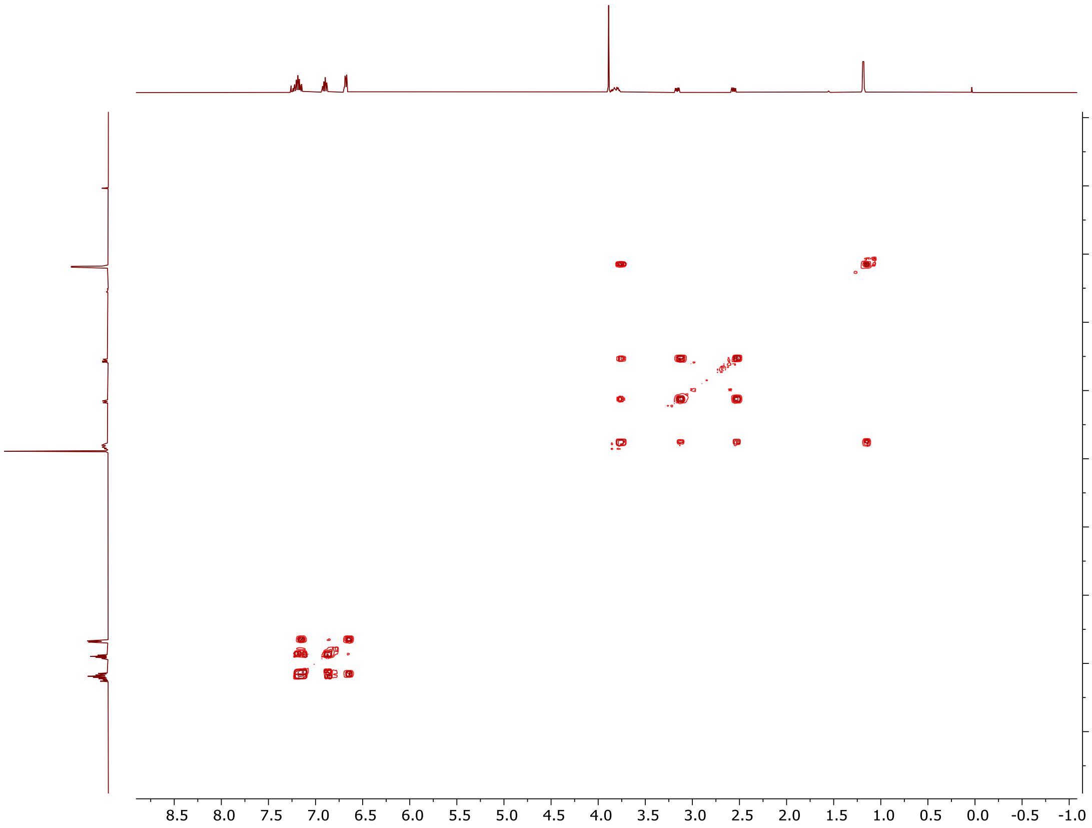

Figure S218: ^{1}H-^{1}H COSY NMR Spectrum of 8p in $CDCl_3$ after isolation via column chromatography.

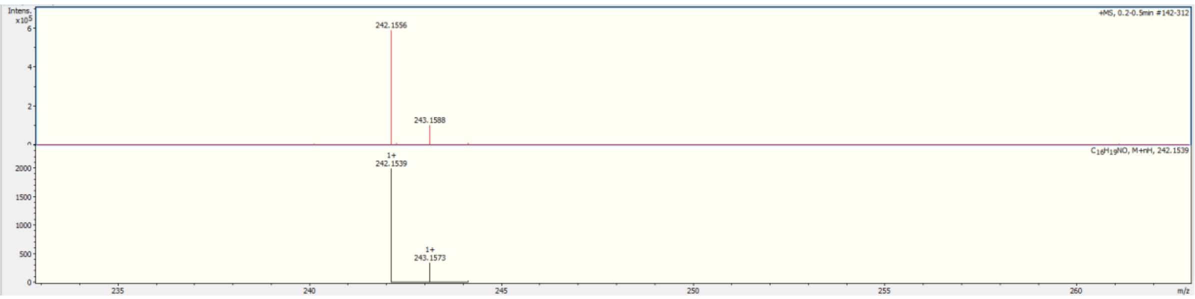

Figure S219: HRMS spectra for compound 8p.

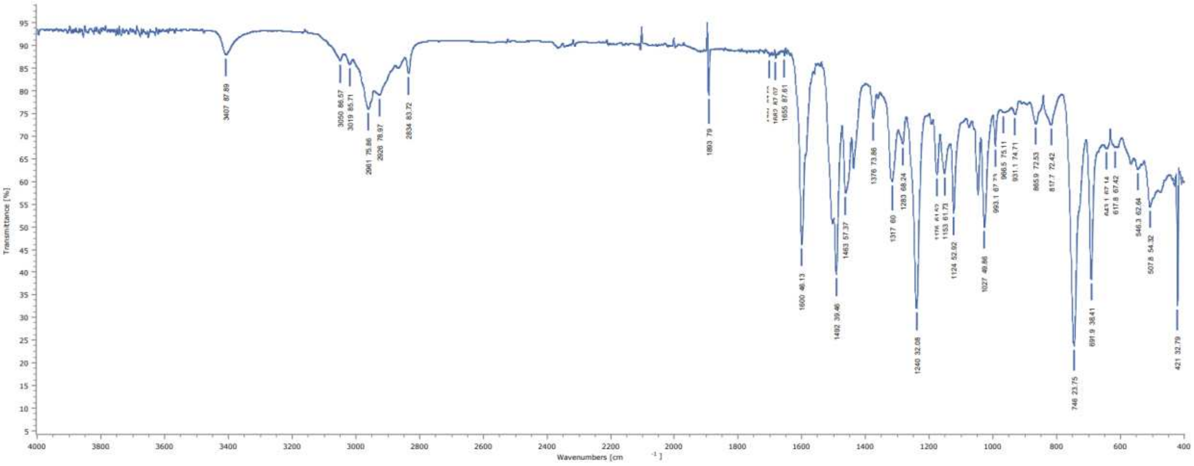

Figure S220: IR spectra for compound 8p.

11.4.16. N-(1-phenylpropan-2-yl)aniline **8q**[18]

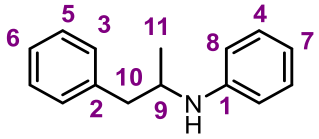

Light yellow oil (75 mg, 36%)

R_f = 0.21 (30% DCM / 70% hexane)

Analytic data is in accordance with those reported in literature.

^{1}H NMR (CDCl$_3$, 500 MHz) δ 7.37 (app. t, J = 7.1 Hz, 2H, C^5-H), 7.30 – 7.22 (m, 5H, C^3, C^4, C^6), 6.77 (app. t, J = 7.3 Hz, 1H, C^7), 6.70 (d, J = 7.9 Hz, 2H, C^8), 3.87 – 3.81 (m, 1H, C^9), 3.59 (*br* s, 1H, NH), 3.01 (dd, J = 13.4, 4.4 Hz, 1H, C^{10}-H), 2.77 (dd, J = 13.4, 7.3 Hz, 1H, C^{10}-H), 1.22 (d, J = 6.4 Hz, 3H, C^{11}-H).

^{13}C{^{1}H} NMR (CDCl$_3$, 126 MHz) δ 147.4 (C^1), 138.7 (C^2), 129.6 (C^3), 129.5 (C^4), 128.5 (C^5), 126.4 (C^6), 117.3 (C^7), 113.5 (C^8), 49.5 (C^9), 42.4 (C^{10}), 20.3 (C^{11}).

HRMS (ESI+): calcd for [M, $C_{15}H_{17}N$]$^+$ 212.1434, found 212.1435.

IR (Neat): 3410, 2962 1600, 1504, 744, 697 cm^{-1}.

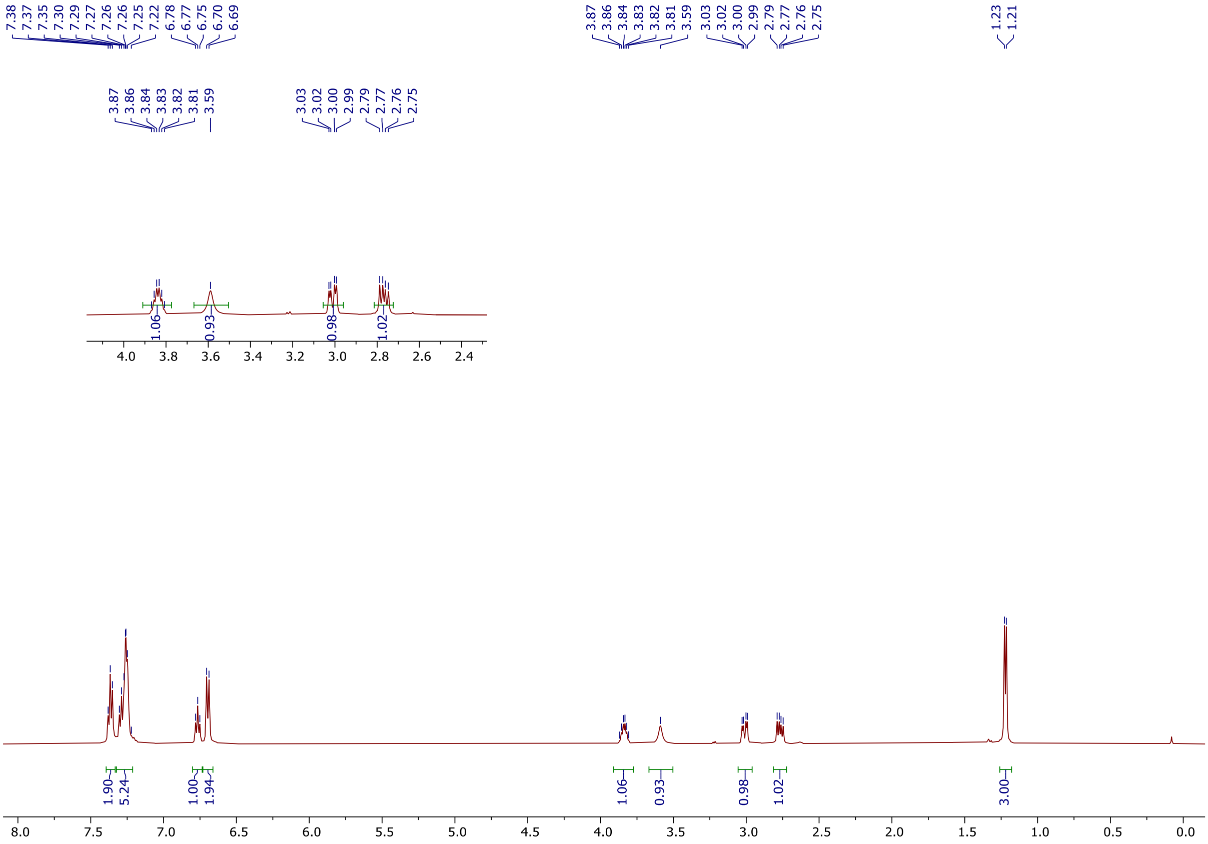

Figure S221: ^{1}H NMR Spectrum of 8q in $CDCl_3$ after isolation via column chromatography.

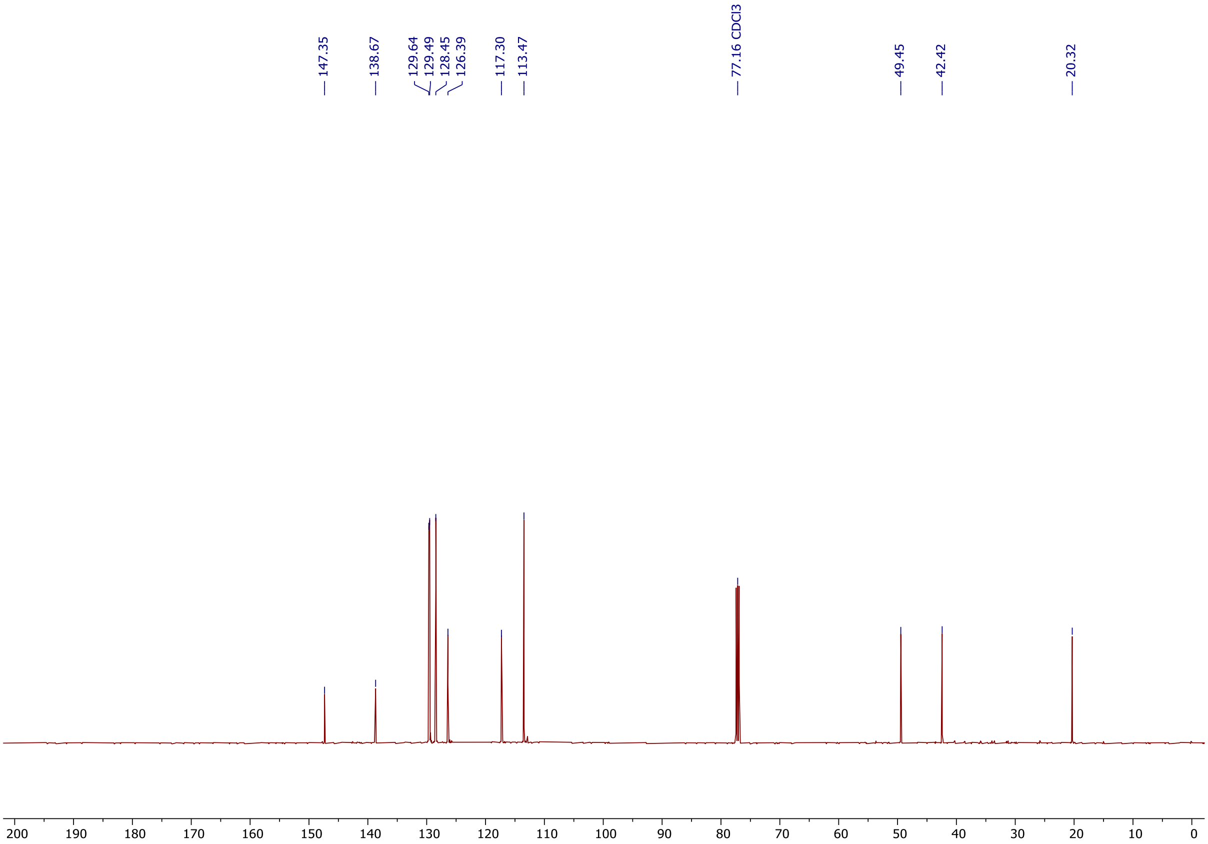

Figure S222: ^{13}C NMR Spectrum of 8q in $CDCl_3$ after isolation via column chromatography.

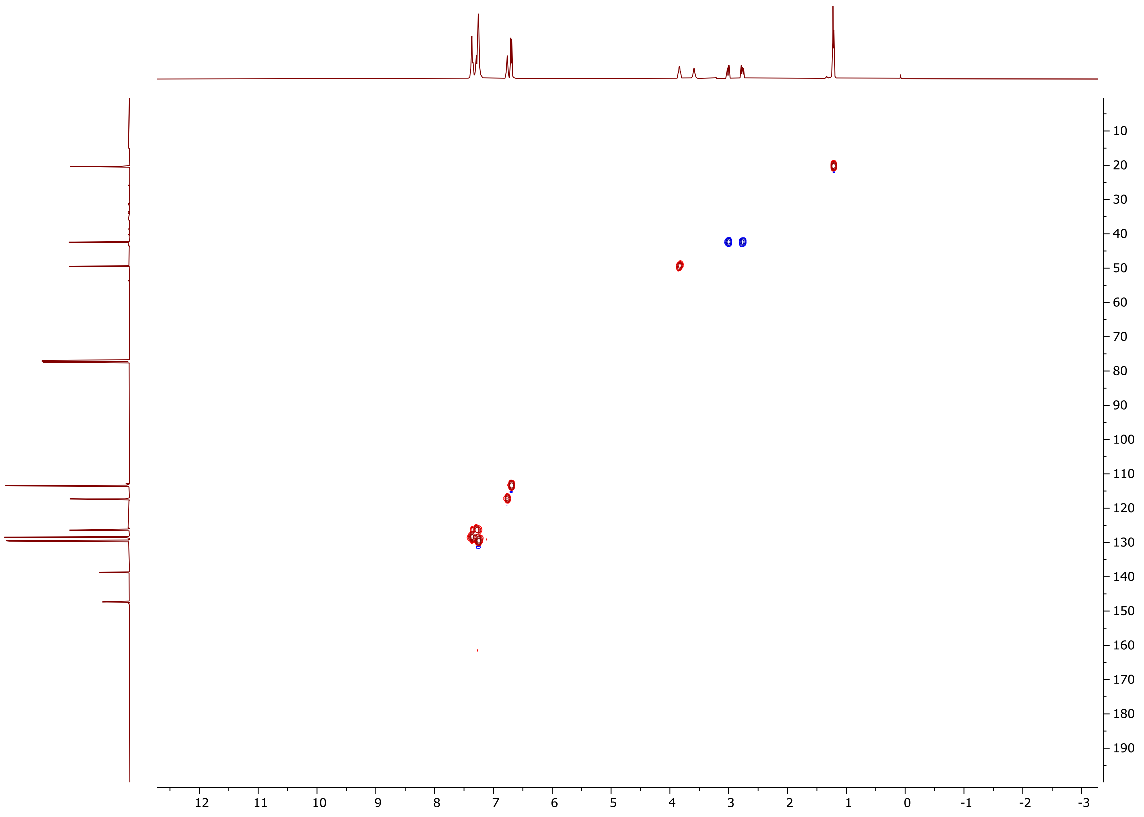

Figure S223: ¹H-¹³C HSQC NMR spectrum of 8q in $CDCl_3$ after isolation via column chromatography.

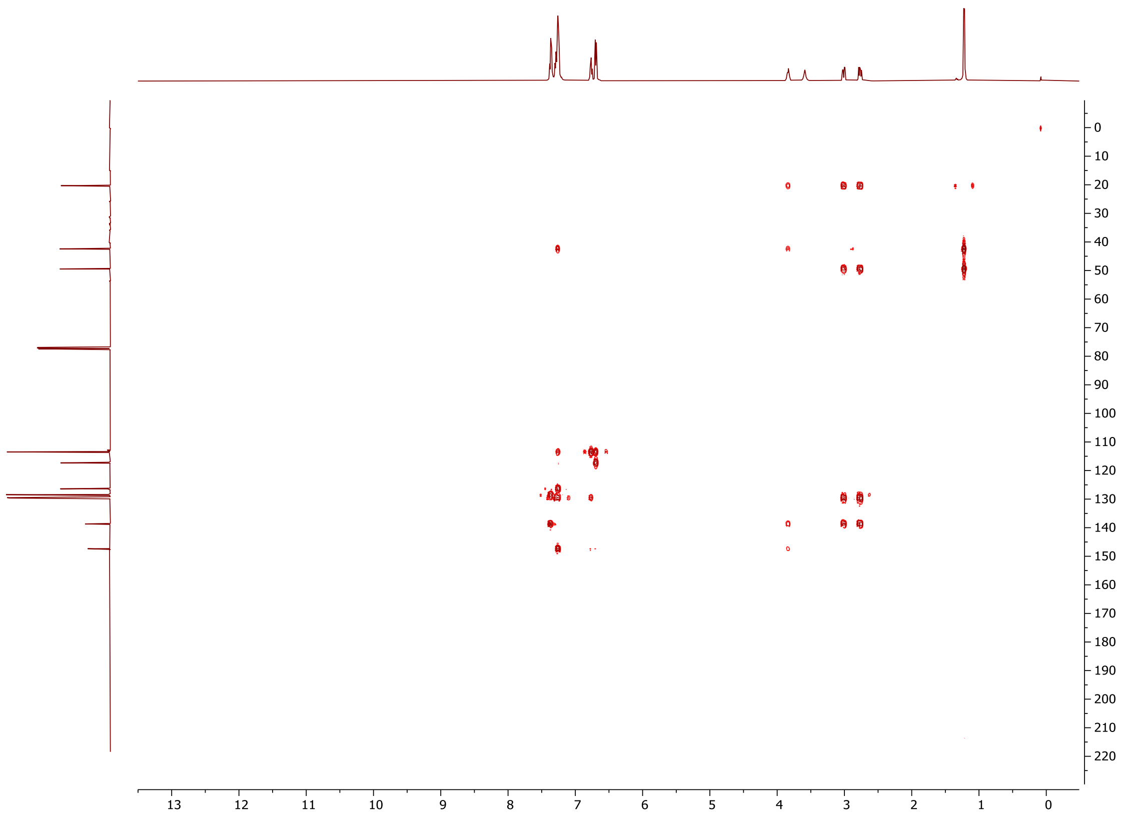

Figure S224: ¹H-¹³C HMBC NMR spectrum of 8q in $CDCl_3$ after isolation via column chromatography.

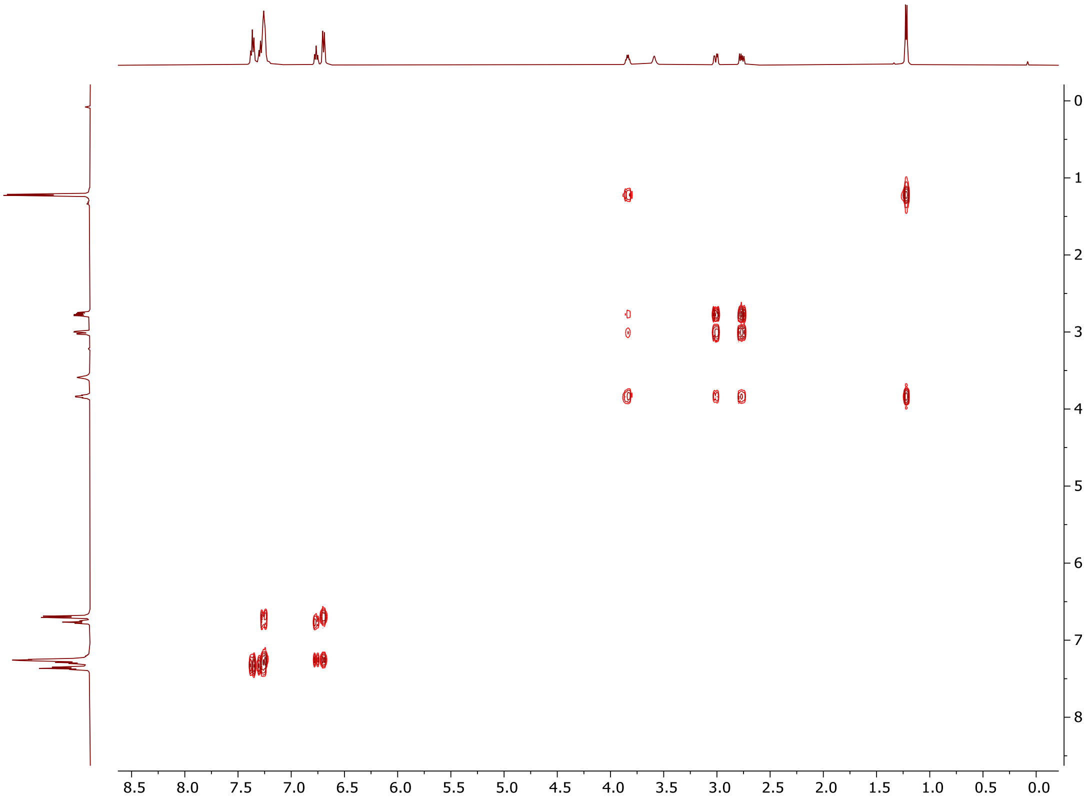

Figure S225: ¹H-¹H COSY NMR spectrum of 8q in $CDCl_3$ after isolation via column chromatography.

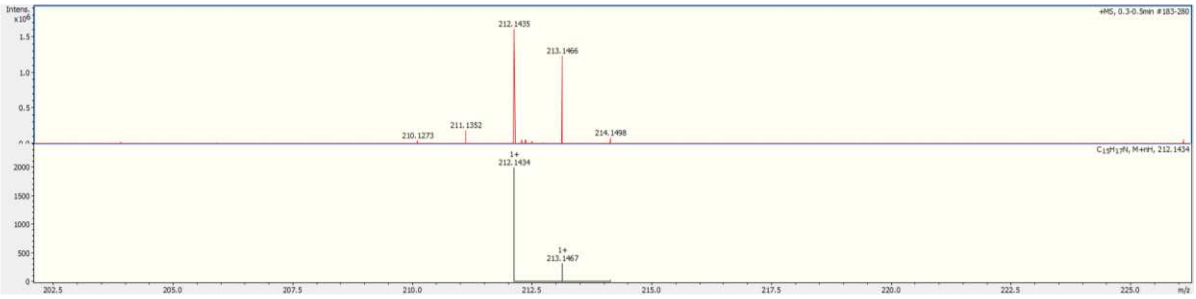

Figure S226: HRMS spectra for compound 8q.

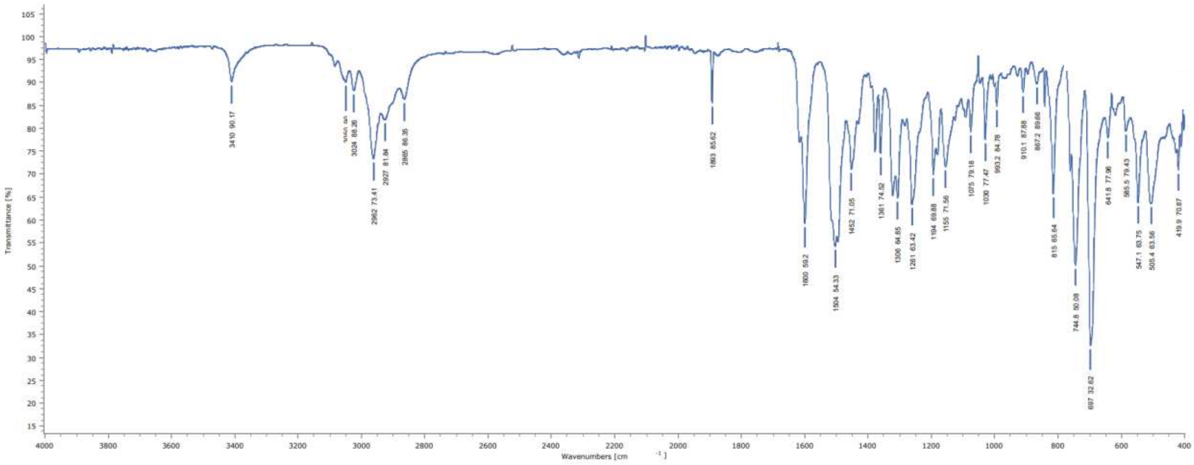

Figure S227: IR spectra for compound 8q.

11.4.17. N[1]-(2,3-dihydro-1H-inden-1-yl)-N[4],N[4]-dimethylbenzene-1,4-diamine **8r**

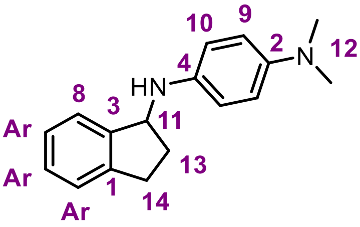

Dark yellow oil (84 mg, 33% (60% conversion compared to I.S))

R_f = 0.20 (50% DCM / 50% hexane)

^{1}H NMR ($CDCl_3$, 500 MHz) δ 7.38 (d, J = 7.3 Hz, 1H, C^8-H), 7.27 – 7.19 (m, 3H, Ar-H), 6.78 (app. d, J = 8.8 Hz, 2H, C^9-H), 6.71 (app. d, J = 8.9 Hz, 2H, C^{10}-H), 4.95 (app. t, J = 6.7 Hz, 1H, C^{11}-H), 3.01 (ddd, J = 15.9, 8.7, 4.3 Hz, 1H, C^{14}-H), 2.91 – 2.85 (m, 1H, C^{14}-H), 2.85 (s 6H, C^{12}-H), 2.60 – 2.54 (m, 1H, C^{13}-H), 1.89 (dddd, J = 12.9, 8.7, 7.6, 6.7 Hz, 1H, C^{13}-H).

$^{13}C\{^1H\}$ NMR ($CDCl_3$, 126 MHz) δ 145.2 (C^1), 144.2 (C^2), 143.7 (C^3), 140.6 (C^4), 127.9 (Ar), 126.7 (Ar), 125.0 (Ar), 124.5 (C^8), 116.2 (C^9), 115.0 (C^{10}), 59.8 (C^{11}), 42.50 (C^{12}), 34.2 (C^{13}), 30.4 (C^{14}).

HRMS (ESI+): calcd for [M, $C_{17}H_{20}N_2$]$^+$ 253.1699, found 253.1715.

IR (Neat): 3351, 2929, 1608, 1513, 944, 811, 748 cm^{-1}.

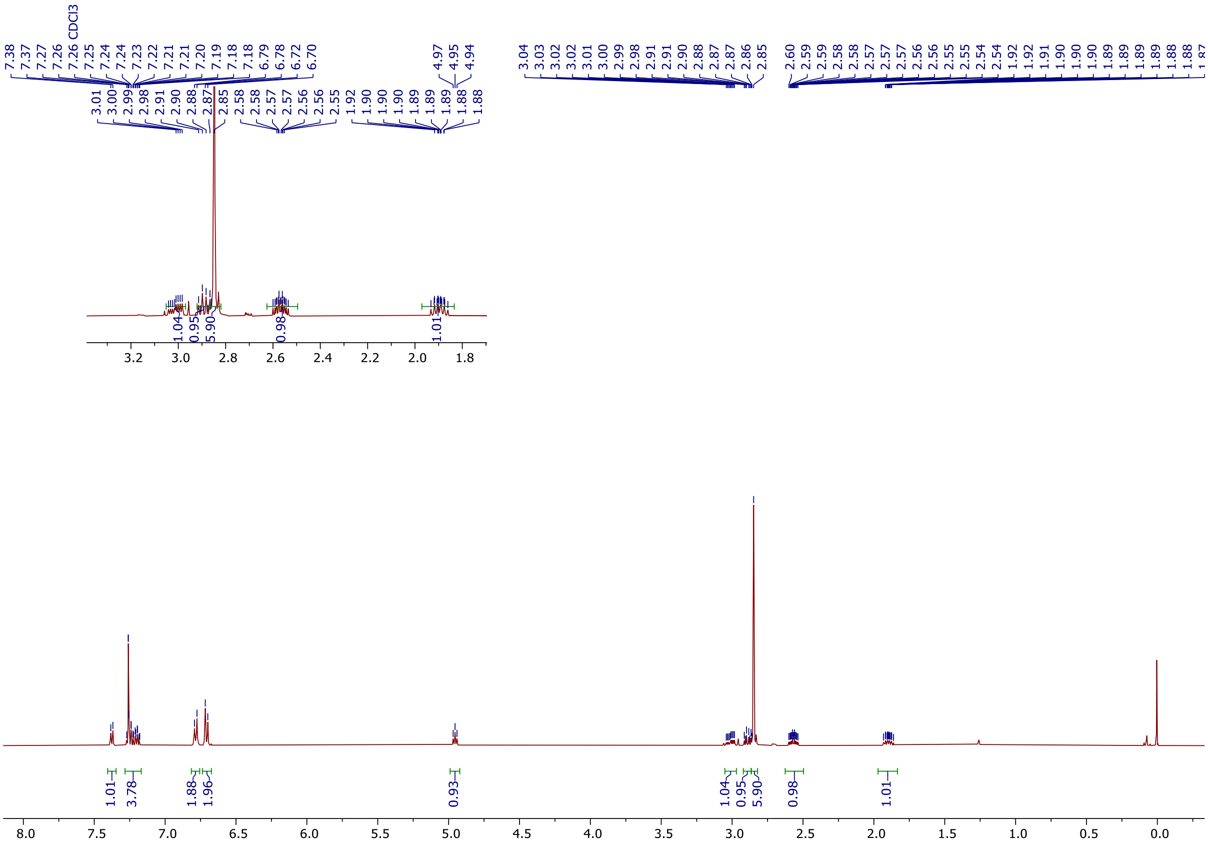

Figure S 228: ^{1}H NMR Spectrum of 8r in $CDCl_3$ after isolation via column chromatography.

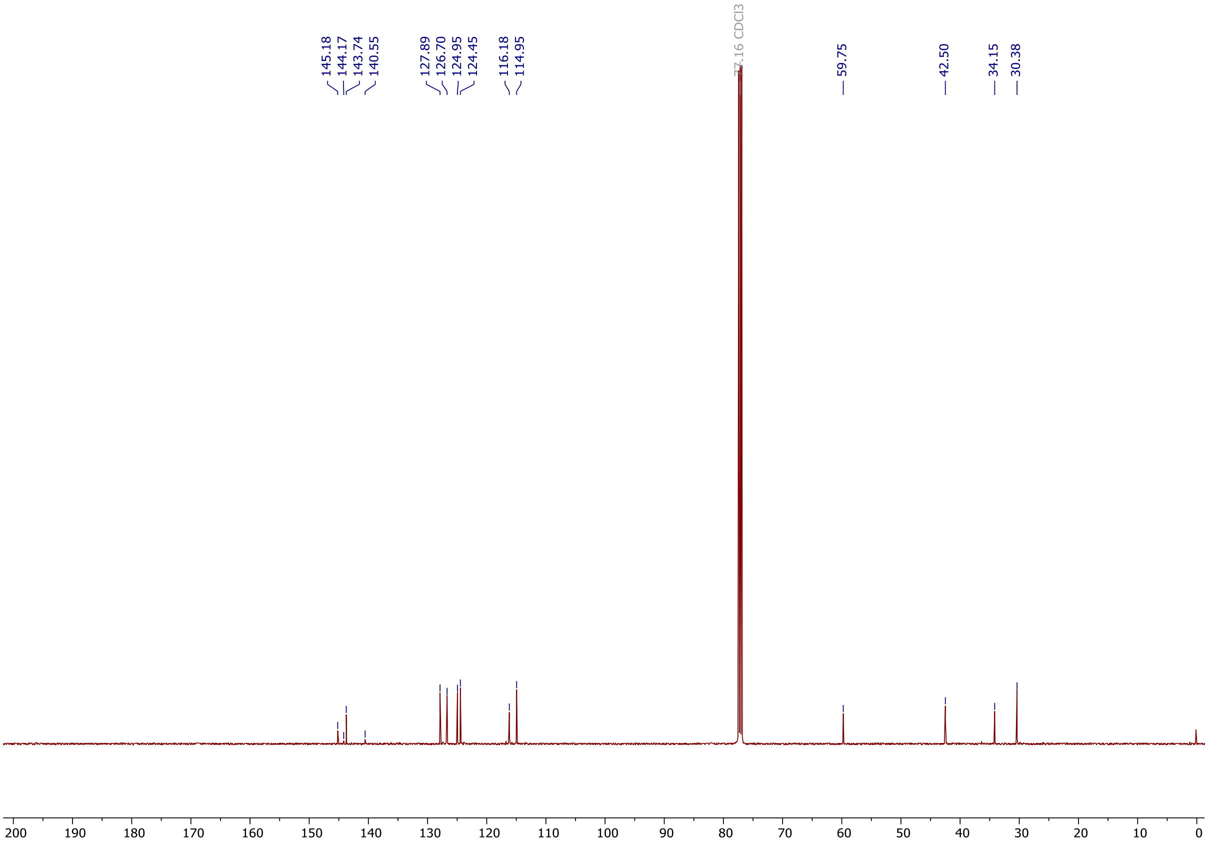

Figure S229: ^{13}C NMR Spectrum of 8r in $CDCl_3$ after isolation via column chromatography.

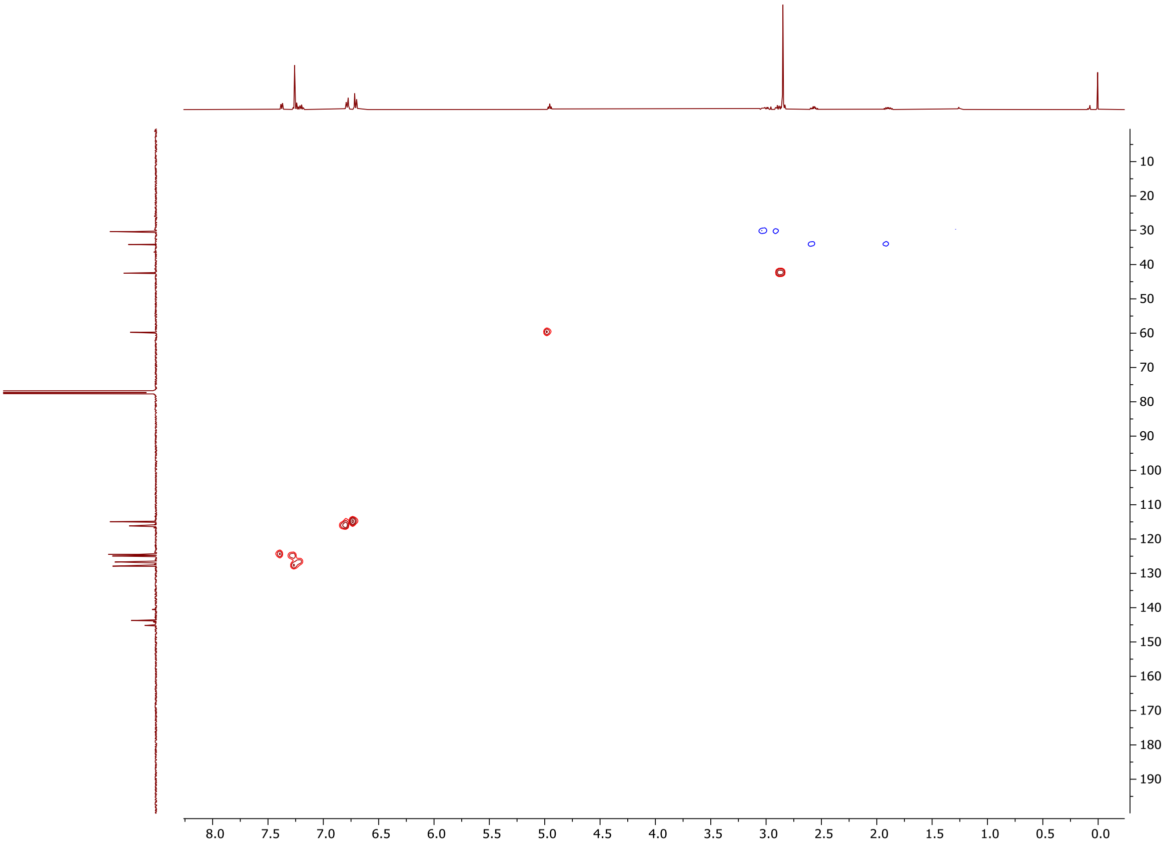

Figure S230: ^{1}*H*-^{13}*C HSQC* NMR Spectrum of 8r in $CDCl_3$ after isolation via column chromatography.

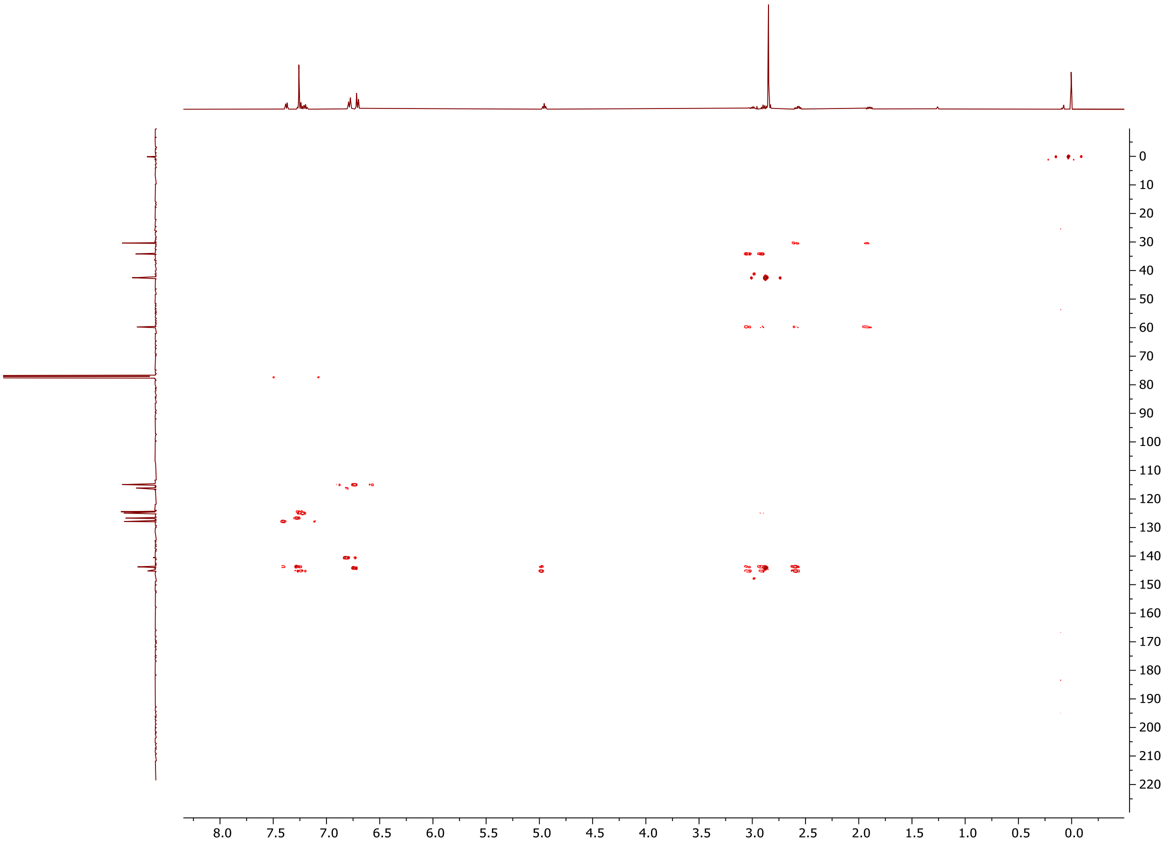

Figure S231: ^{1}H-^{13}C HMBC NMR Spectrum of 8r in $CDCl_3$ after isolation via column chromatography.

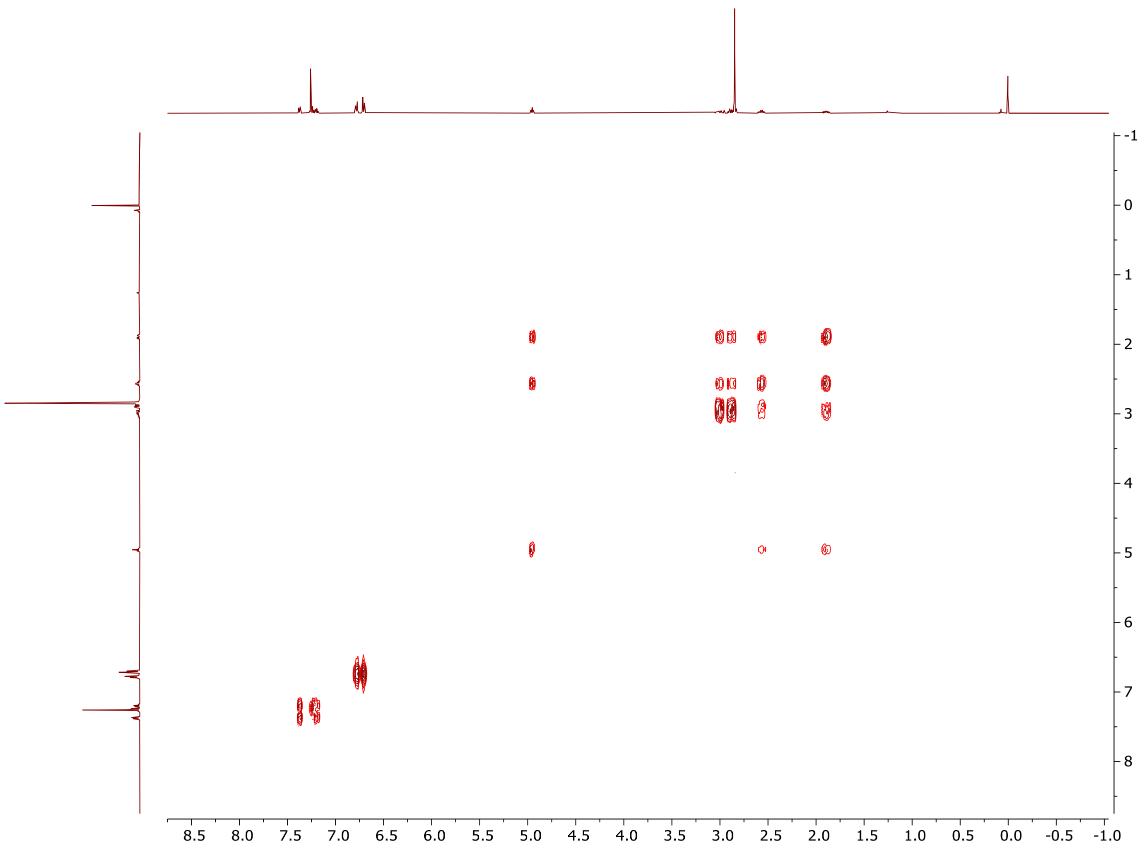

Figure S233: ^{1}H-^{1}H COSY NMR Spectrum of 8r in $CDCl_3$ after isolation via column chromatography.

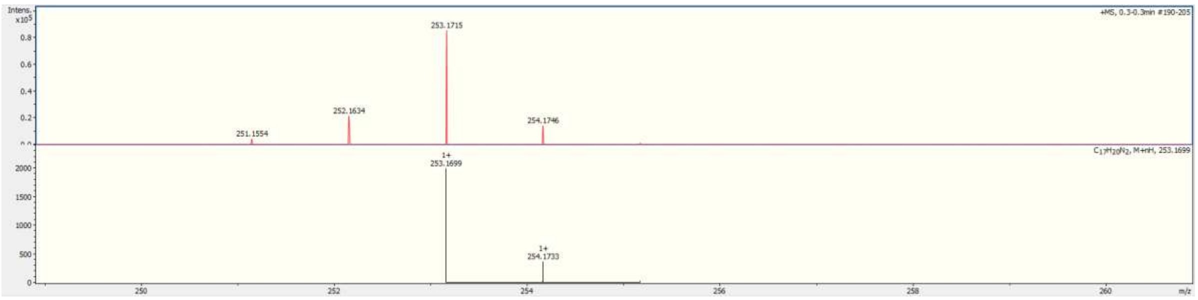

Figure S232: HRMS spectra for compound 8r.

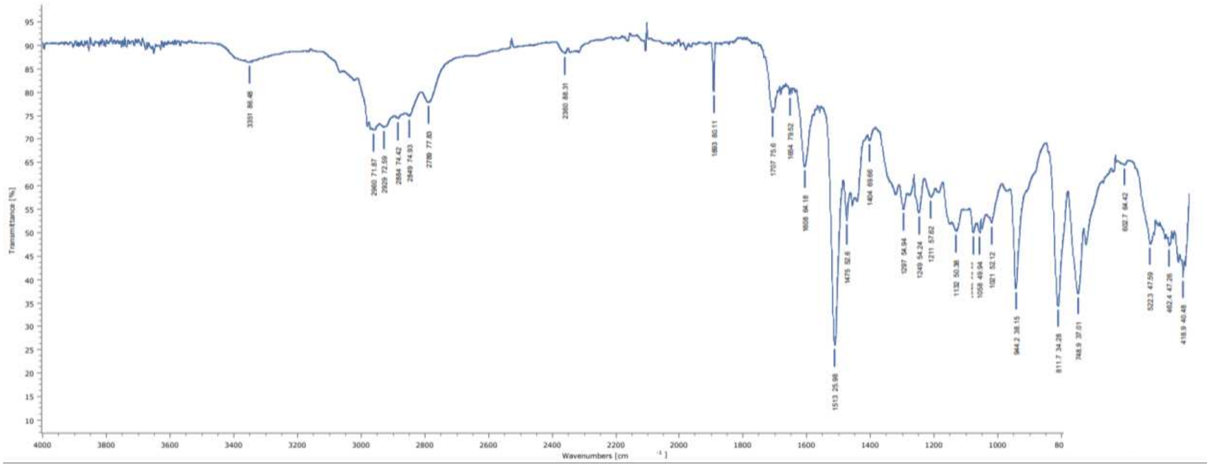

Figure S234: IR spectra for compound 8r.

11.4.18. 4-(methylthio)-N-(2-phenylpropan-2-yl)aniline **8s**

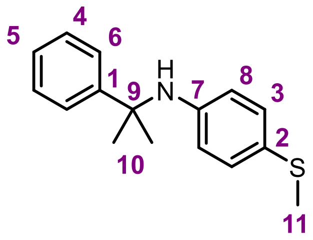

Bright yellow oil (63 mg, 24%)

R_f = 0.23 (30% DCM / 70% hexane)

^{1}H NMR ($CDCl_3$, 500 MHz) δ 7.53 (d, J = 7.5 Hz, 2H, C^6-H), 7.37 (app.t, J = 7.7 Hz, 2H, C^4-H), 7.28 (d, J = 6.5 Hz, 1H, C^5-H), 7.07 (app. d, J = 8.6 Hz, 2H, C^3-H), 6.32 (app. d, J = 8.6 Hz, 2H, C^8-H), 4.11 (*br* s, 1H, NH), 2.39 (s, 3H, C^{11}-H), 1.67 (s, 6H, C^{10}-H).

^{13}C{^{1}H} NMR ($CDCl_3$, 126 MHz) δ 147.3 (C^1), 145.0 (C^7), 130.8 (C^8), 128.7 (C^4), 126.5 (C^5), 125.7 (C^6), 124.1 (C^2), 116.0 (C^3), 56.0 (C^9), 30.8 (C^{10}), 19.0 (C^{11}).

HRMS (EI): calcd for [M, $C_{16}H_{19}NS$]$^+$ 258.1311, found 258.1309.

IR (Neat): 3437, 3081, 2963, 1597, 1441, 577 cm^{-1}.

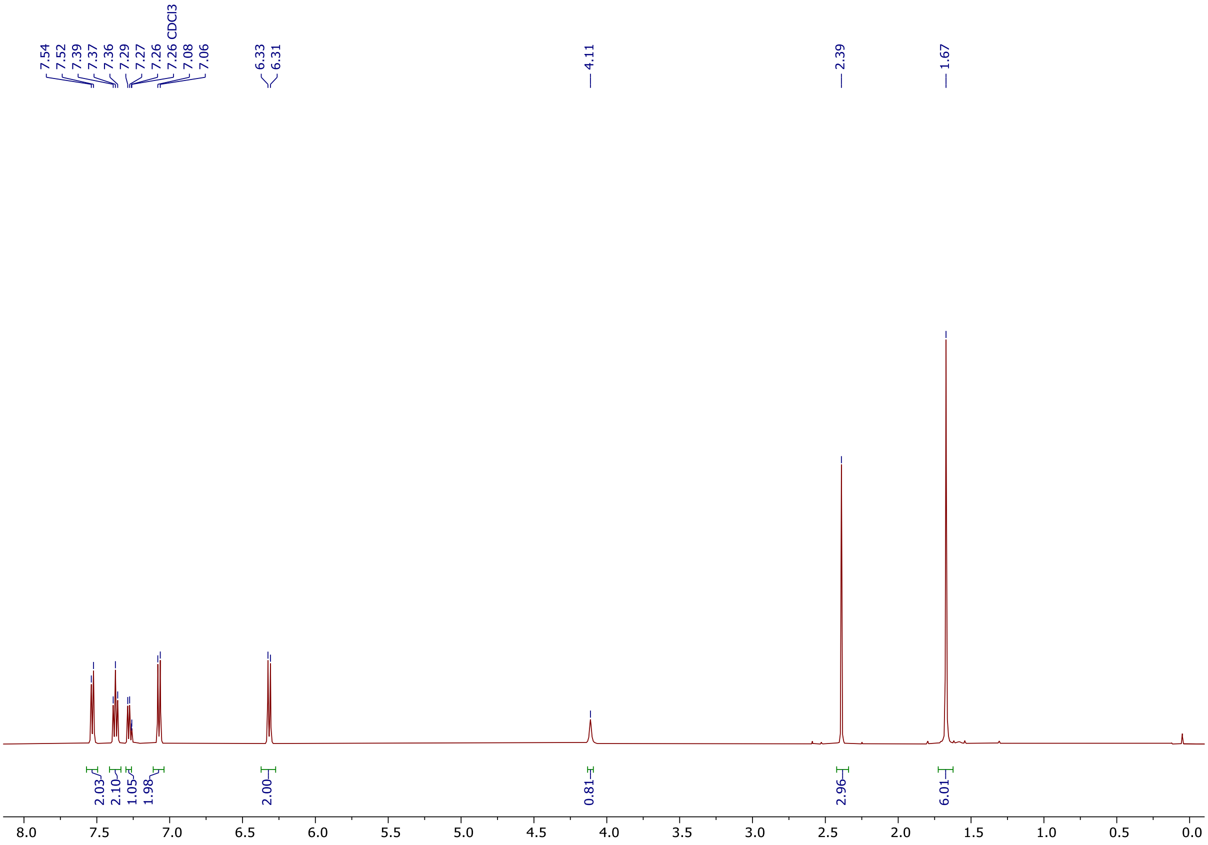

Figure S235: ^{1}H NMR Spectrum of 8s in $CDCl_3$ after isolation via column chromatography.

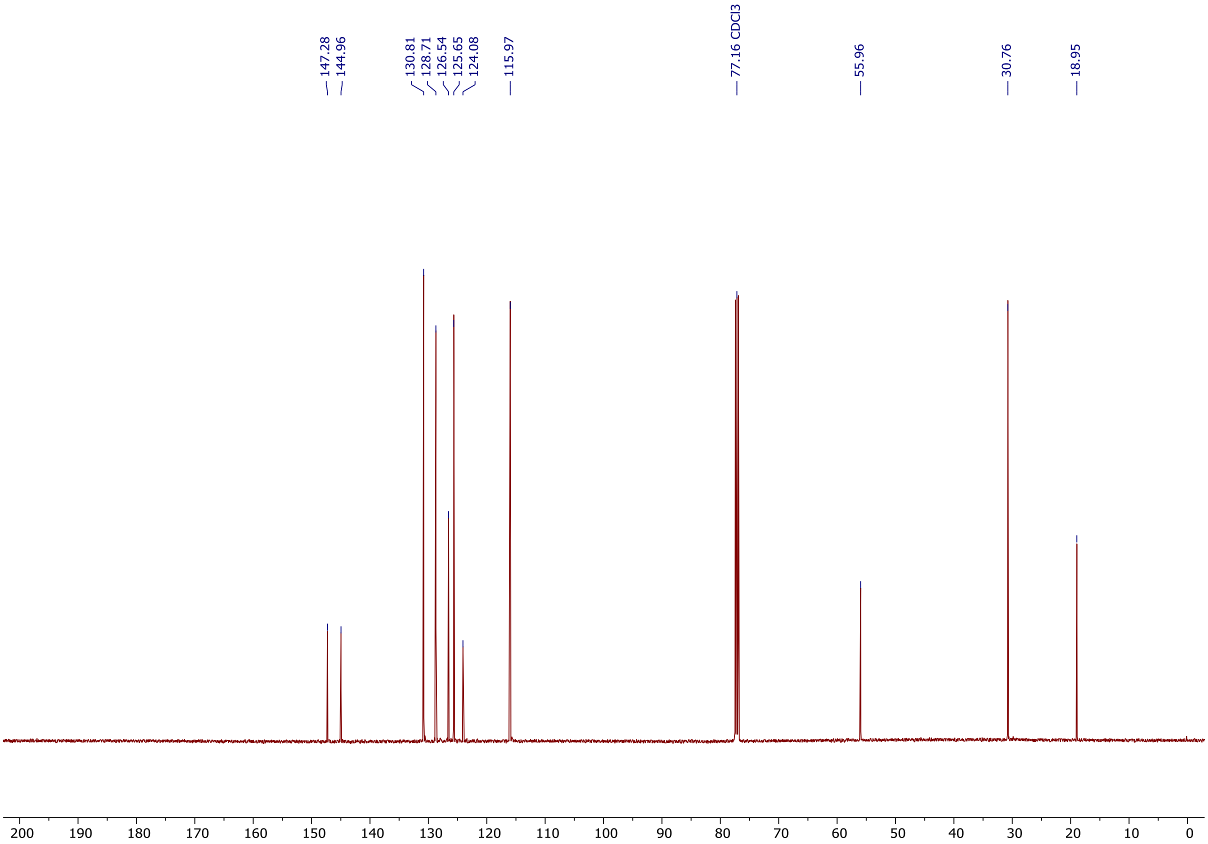

Figure S236: ^{13}C NMR Spectrum of 8s in $CDCl_3$ after isolation via column chromatography.

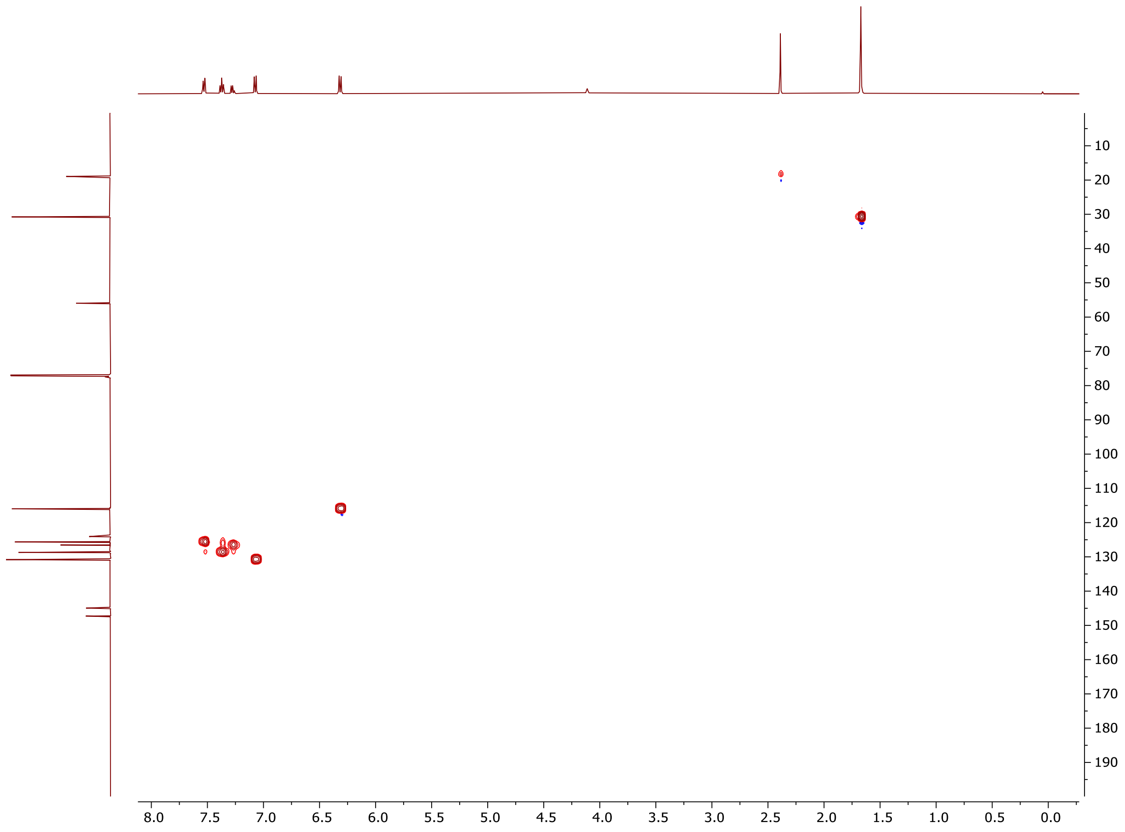

Figure S237: ^{1}H-^{13}C HSQC NMR Spectrum of 8s in $CDCl_3$ after isolation via column chromatography.

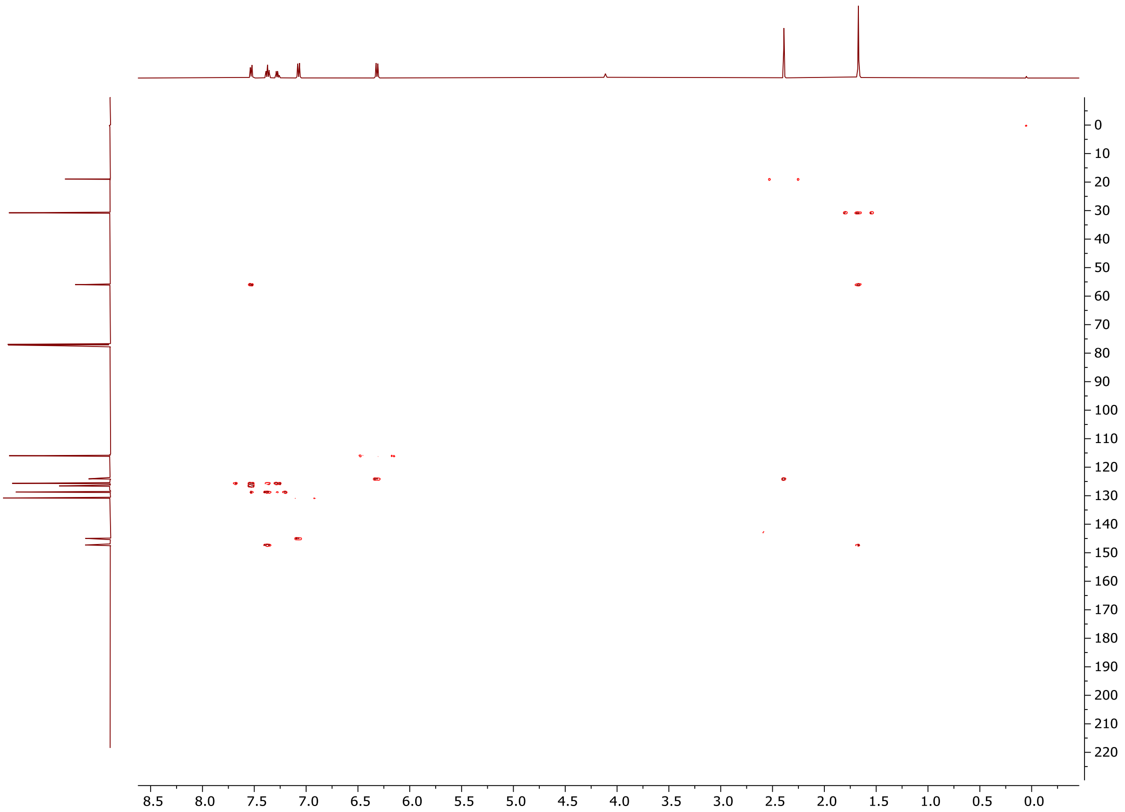

Figure S238: ^{1}H-^{13}C HMBC NMR Spectrum of 8s in $CDCl_3$ after isolation via column chromatography.

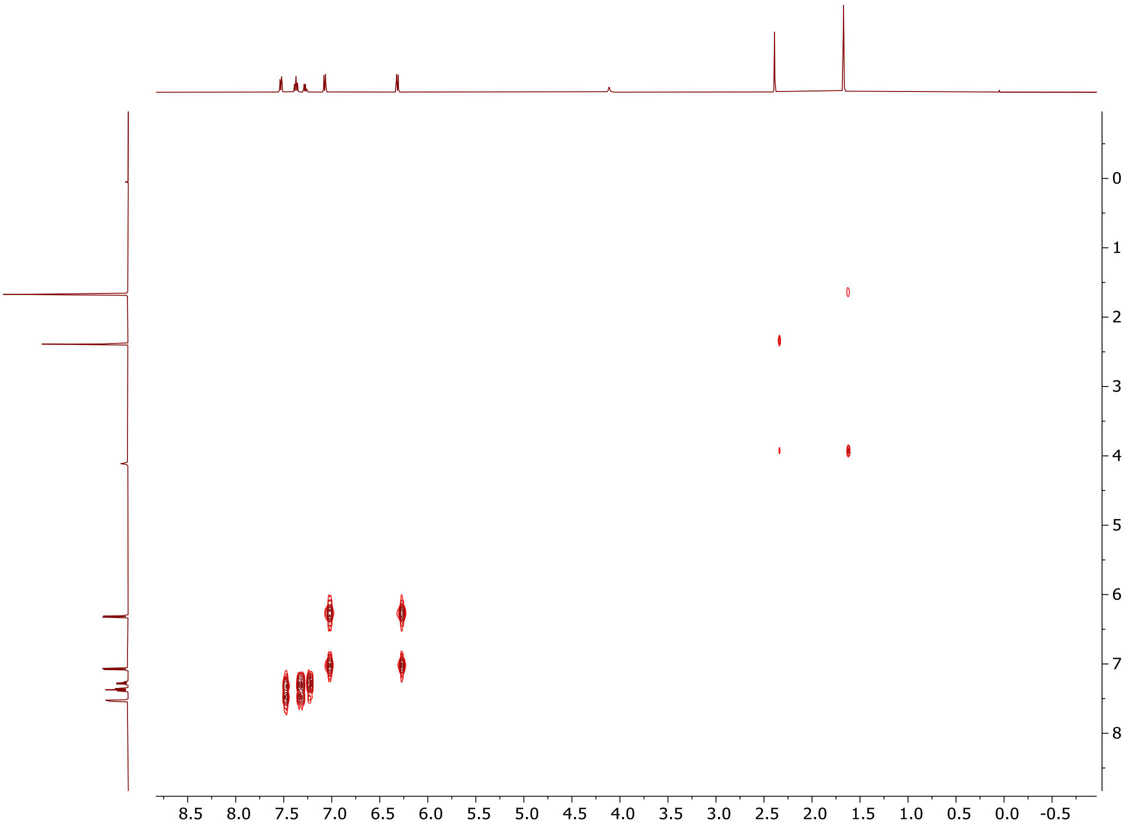

Figure S239: ^{1}H-^{1}H COSY NMR Spectrum of 8s in $CDCl_3$ after isolation via column chromatography.

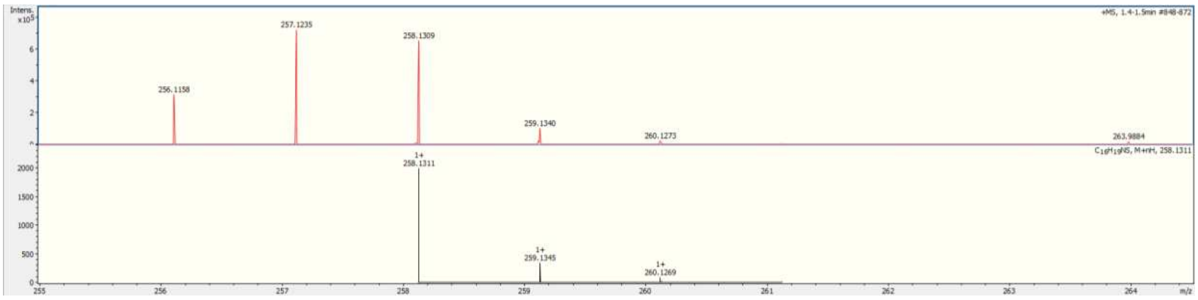

Figure S240: HRMS spectra for compound 8s.

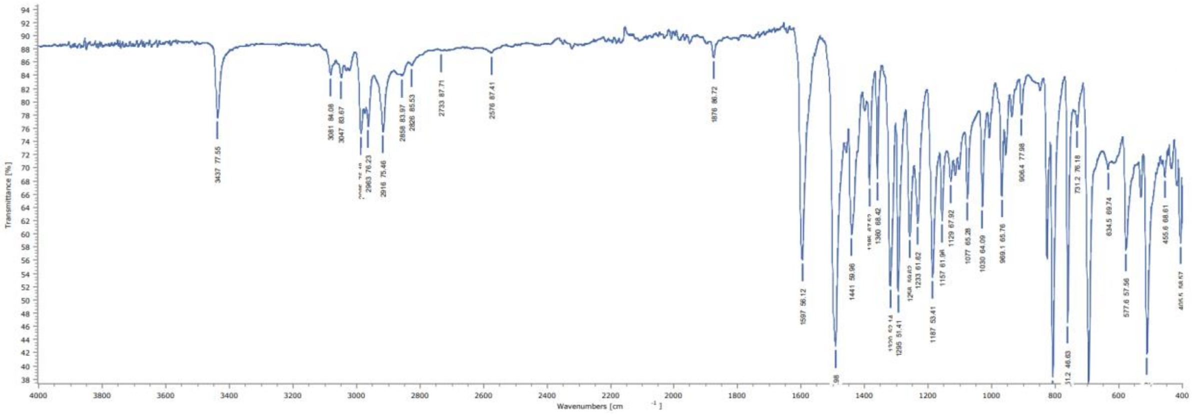

Figure S241: IR spectra for compound 8s.

11.4.19. N-(4-(methylthio)phenyl)-2,3-dihydro-1H-inden-1-amine **8t**

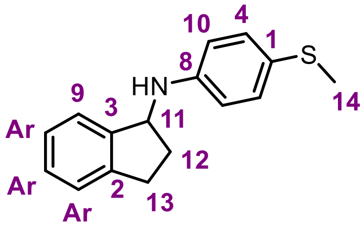

Bright yellow oil (140 mg, 63%)

R_f = 0.21 (30% DCM / 70% hexane)

^{1}H NMR ($CDCl_3$, 500 MHz) δ 7.42 (d, J = 7.4 Hz, 1H, C^9-H), 7.34 – 7.25 (m, 3H, Ar-H), 7.31 (app. d, J = 8.7 Hz, 2H, C^4-H), 6.72 (app. d, J = 8.7 Hz, 2H, C^{10}-H), 5.05 (app. t, J = 6.7 Hz, 1H, C^{11}-H), 3.99 (*br* s, 1H, NH), 3.08 (ddd, J = 15.9, 8.6, 4.3 Hz, 1H, C^{13}-H), 2.96 (app. dt, J = 15.9, 7.9 Hz, 1H, C^{13}-H), 2.67 – 2.61 (m, 1H, C^{12}-H), 2.99 (s, 3H, C^{14}-H), 1.96 (dddd, J = 12.9, 8.6, 7.6, 6.7 Hz, 1H, C^{12}-H).

^{13}C{^{1}H} NMR ($CDCl_3$, 126 MHz) δ 146.7 (C^8), 144.4 (C^2), 143.7 (C^3), 131.8 (C^4), 128.1 (Ar), 126.8 (Ar), 125.0 (Ar), 124.3 (C^1), 124.3 (C^9), 113.8 (C^{10}), 58.7 (C^{11}), 33.9 (C^{12}), 30.3 (C^{13}), 19.3 (C^{14}).

HRMS (ESI+): calcd for [M, $C_{16}H_{18}NS$]$^+$ 256.1154, found 256.1156.

IR (Neat): 2961, 2916, 1596, 1497, 813, 749 cm^{-1}.

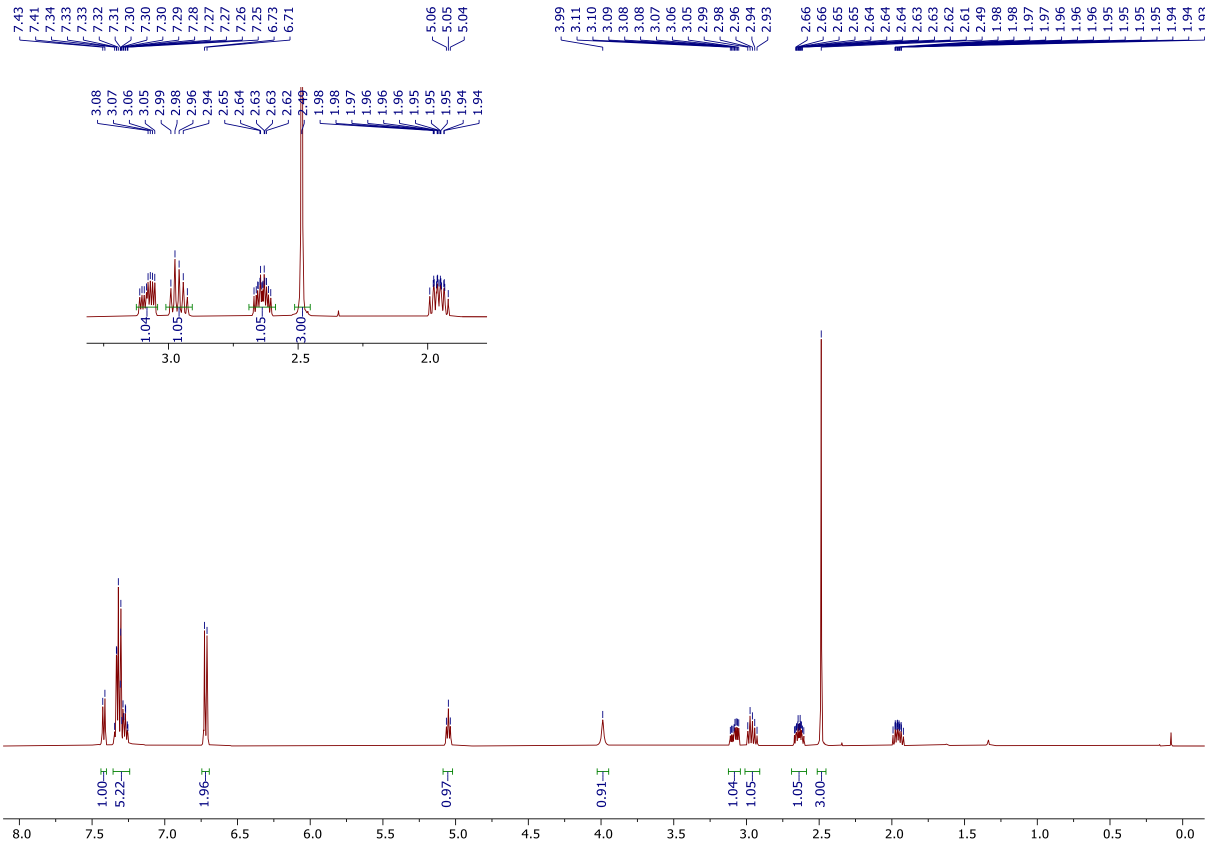

Figure S242: ^{1}H NMR Spectrum of 8t in $CDCl_3$ after isolation via column chromatography.

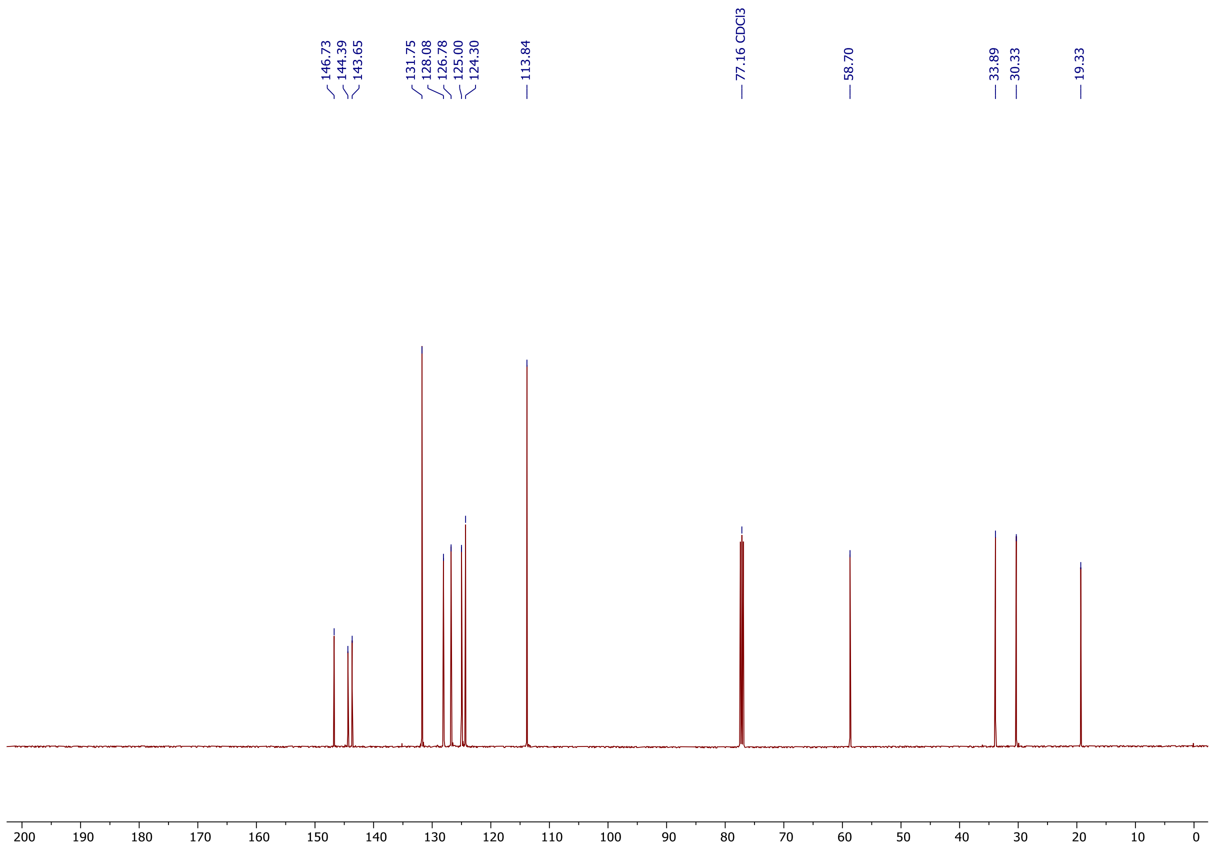

Figure S243: ^{13}C NMR Spectrum of 8t in $CDCl_3$ after isolation via column chromatography.

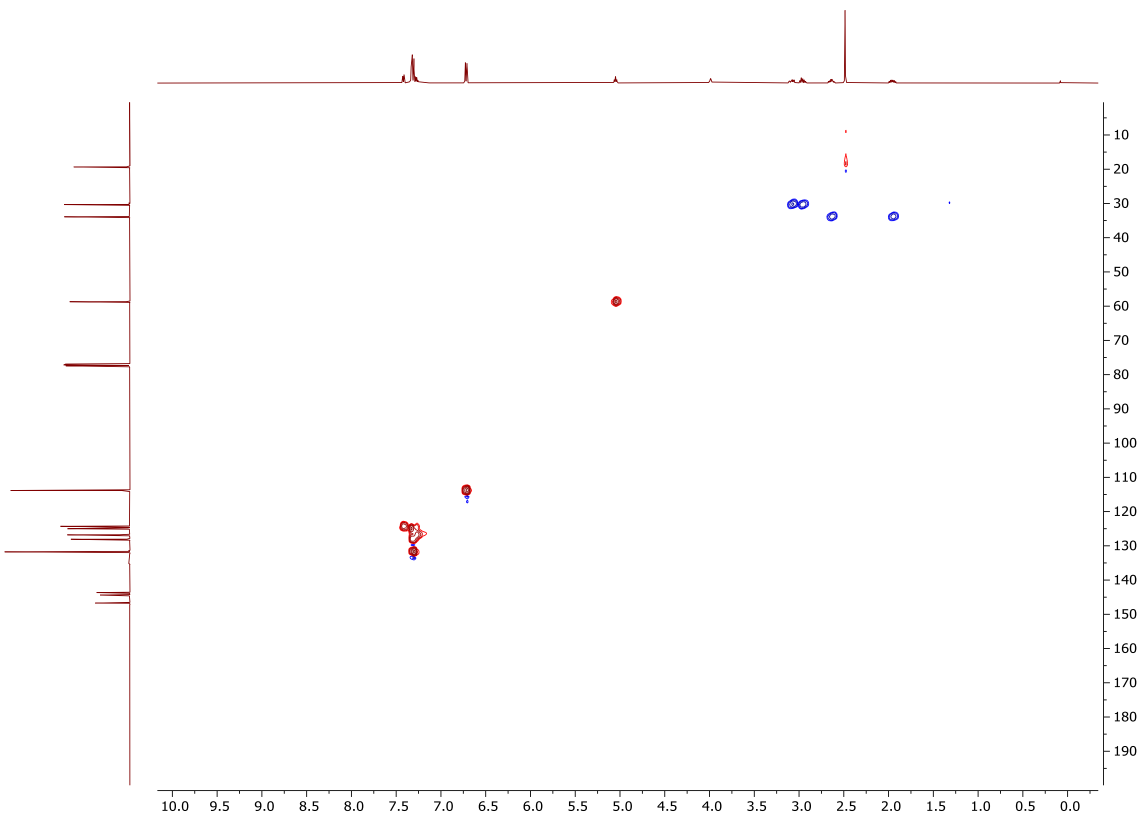

Figure S244: ^{1}H-^{13}C HSQC NMR Spectrum of 8t in $CDCl_3$ after isolation via column chromatography.

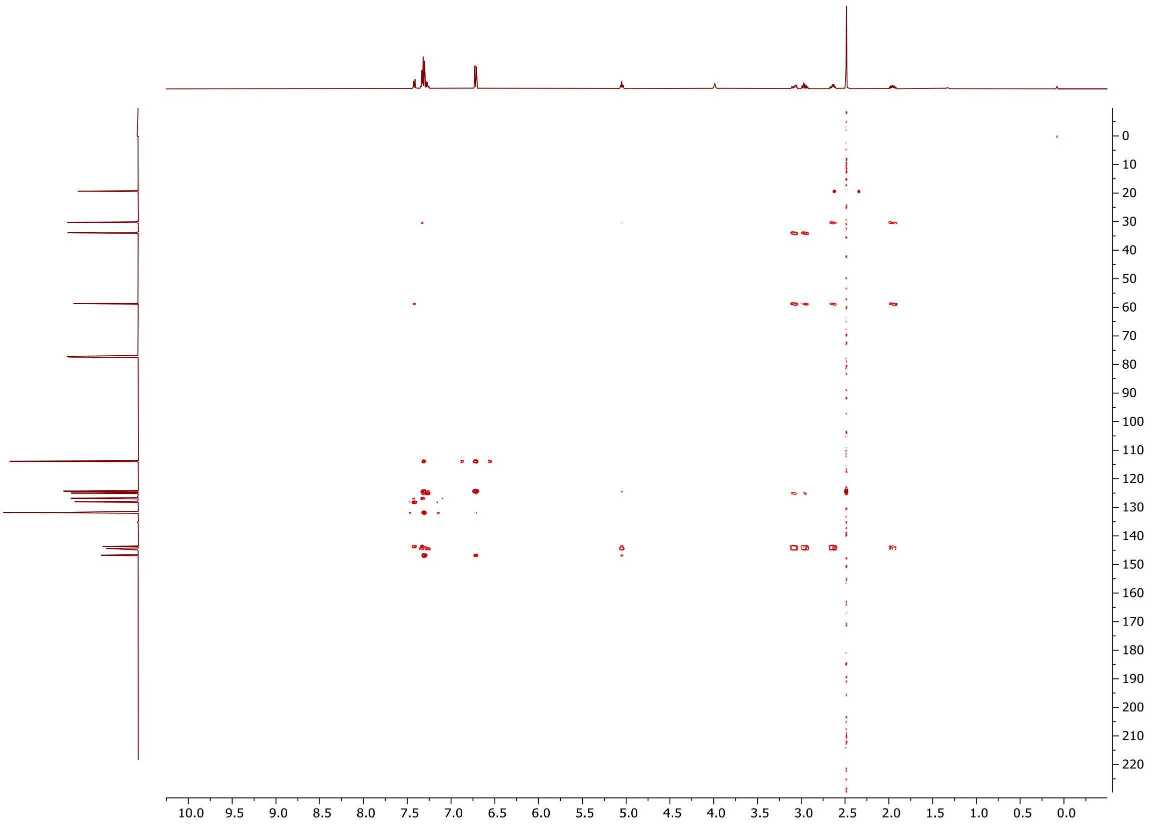

Figure S245: ^{1}H-^{13}C HMBC NMR Spectrum of 8t in $CDCl_3$ after isolation via column chromatography.

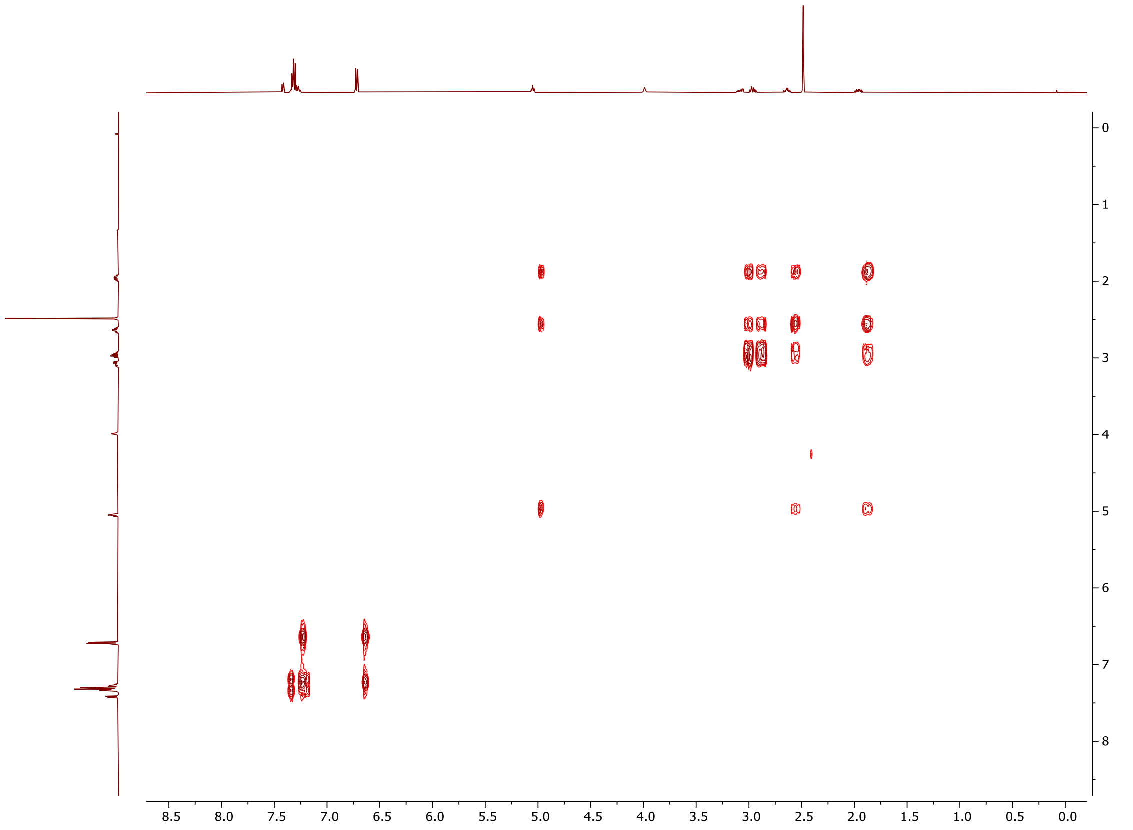

Figure S246: ^{1}H-^{1}H COSY NMR Spectrum of 8t in $CDCl_3$ after isolation via column chromatography.

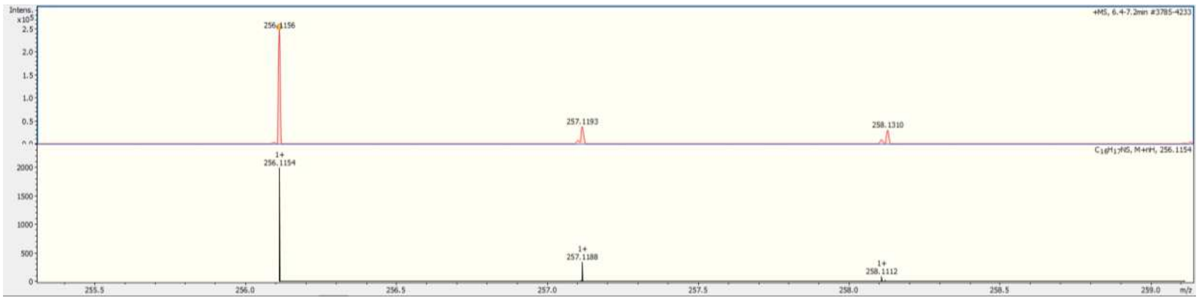

Figure S247: HRMS spectra for compound 8t.

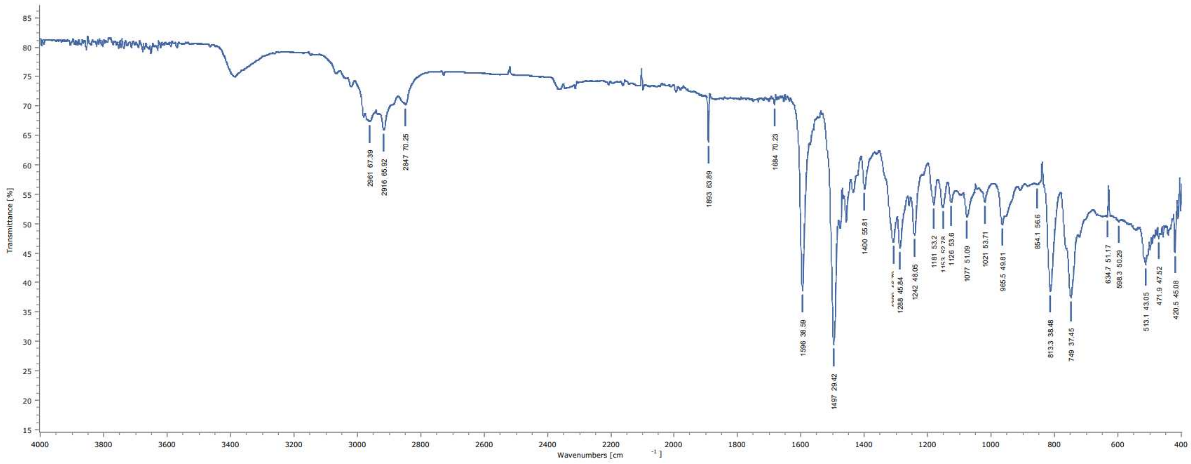

Figure S248: IR spectra for compound 8t.

11.4.20. N-(1-(2-methoxyphenyl)propan-2-yl)-4-(methylthio)aniline **8u**

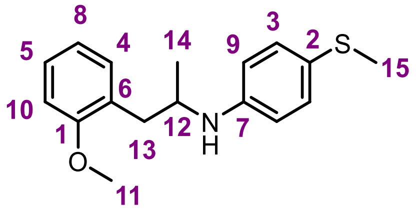

Dark yellow oil (106 mg, 37%)

R_f = 0.40 (50% DCM / 50% hexane)

^{1}H NMR ($CDCl_3$, 500 MHz) δ 7.24 – 7.18 (m, 1H, C^5-H), 7.21 (d, J = 8.3 Hz, 2H, C^3-H), 7.11 (d, J = 7.0 Hz, 1H, C^4-H), 6.89 – 6.85 (m, 2H, C^8-H, C^{10}-H), 6.59 (d, J = 8.3 Hz, 2H, C^9-H), 3.86 (s, 2H, C^{11}-H), 3.77 – 3.71 (m, 1H, C^{12}-H), 3.11 (dd, J = 13.2, 5.5 Hz, 1H, C^{13}-H), 2.52 (dd, J = 13.2, 7.4 Hz, 1H, C^{13}-H), 2.39 (s, 3H, C^{15}-H), 1.15 (d, J = 5.9 Hz, 3H, C^{14}-H).

^{13}C{^{1}H} NMR ($CDCl_3$, 126 MHz) δ 157.7 (C^1), 146.8 (C^7), 132.0 (C^3), 131.4 (C^4), 127.8 (C^5), 127.5 (C^6), 123.3 (C^2), 120.6 (C^8), 113.8 (C^9), 110.5 (C^{10}), 55.3 (C^{11}), 49.0 (C^{12}), 37.7 (C^{13}), 20.6 (C^{14}), 19.6 (C^{15}).

HRMS (ESI+): calcd for [M, $C_{17}H_{21}NOS$]$^+$ 288.1417, found 288.1422.

IR (Neat): 3401, 2961, 2917, 1597, 1492, 1240, 751 cm^{-1}.

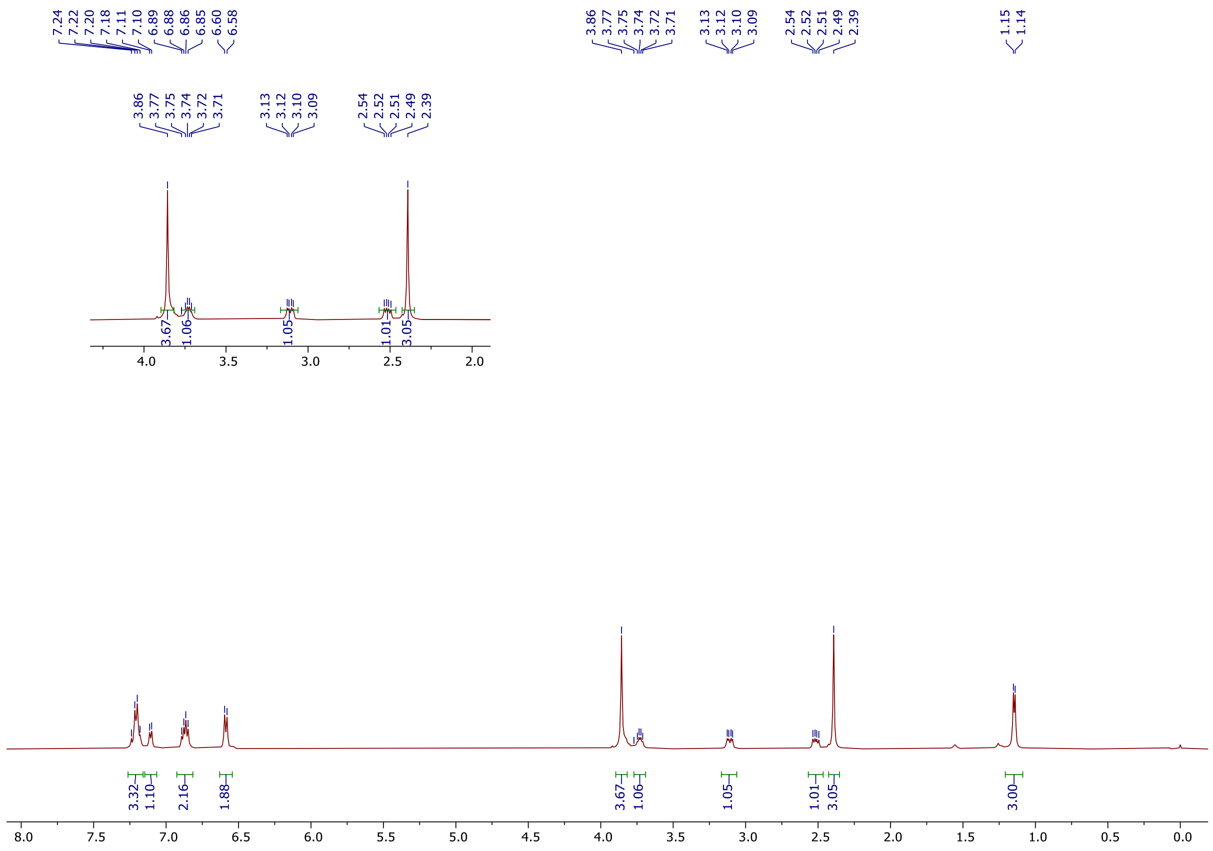

Figure S249: ^{1}H NMR Spectrum of 8u in $CDCl_3$ after isolation via column chromatography.

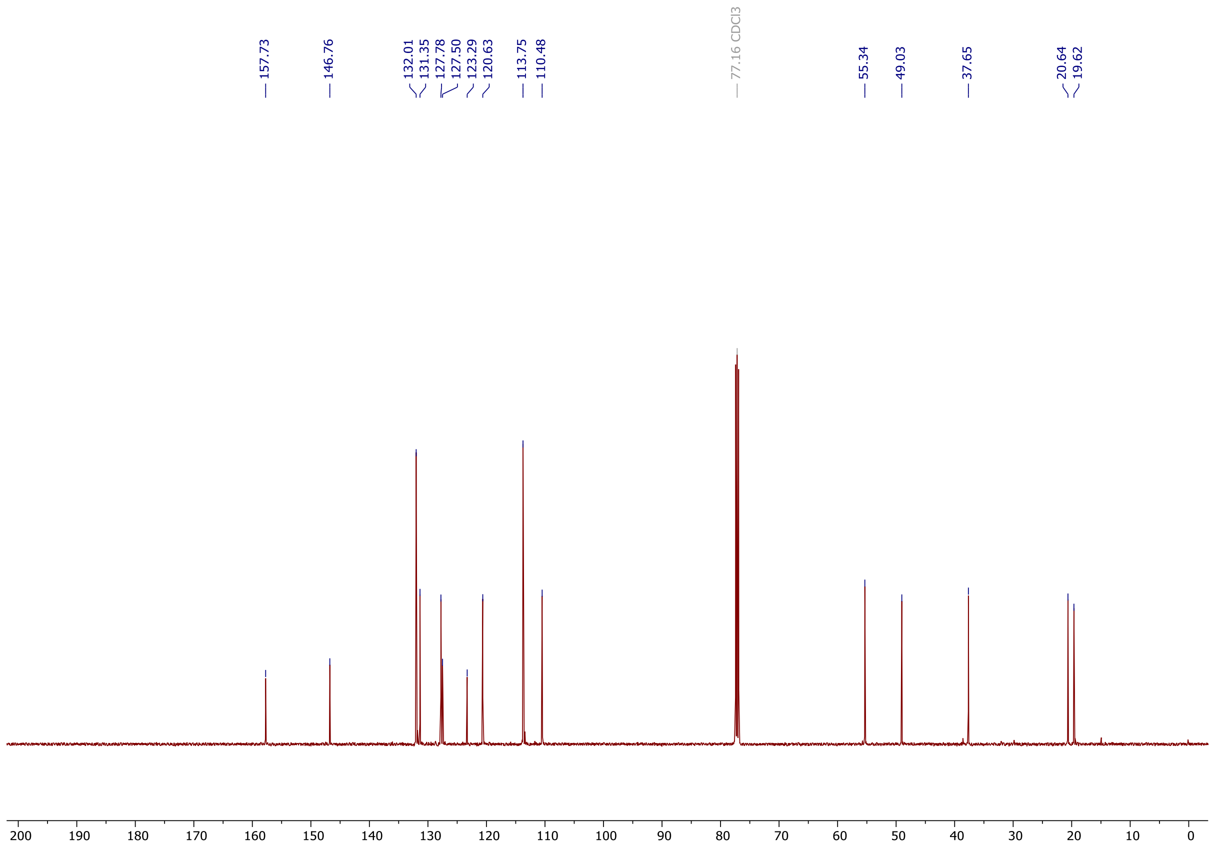

Figure S250: ^{13}C NMR Spectrum of 8u in $CDCl_3$ after isolation via column chromatography.

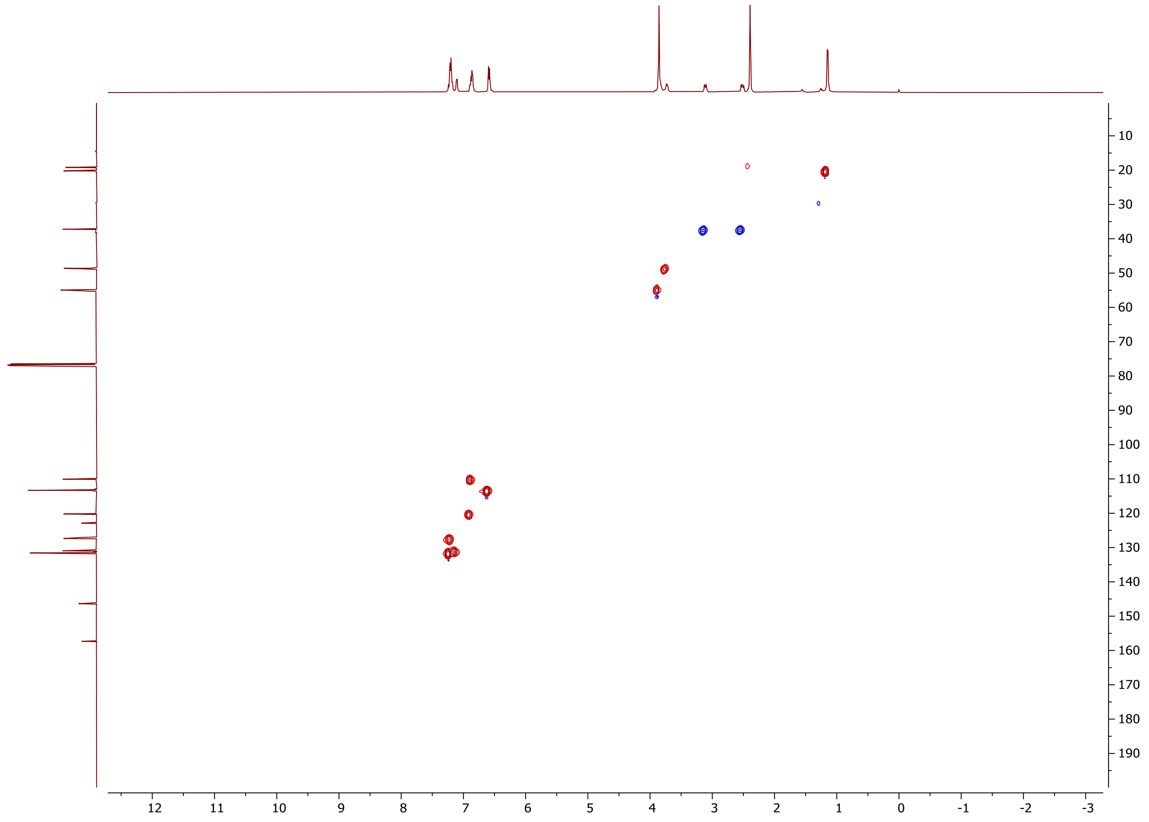

Figure S251: ^{1}H-^{13}C HSQC NMR Spectrum of 8u in $CDCl_3$ after isolation via column chromatography.

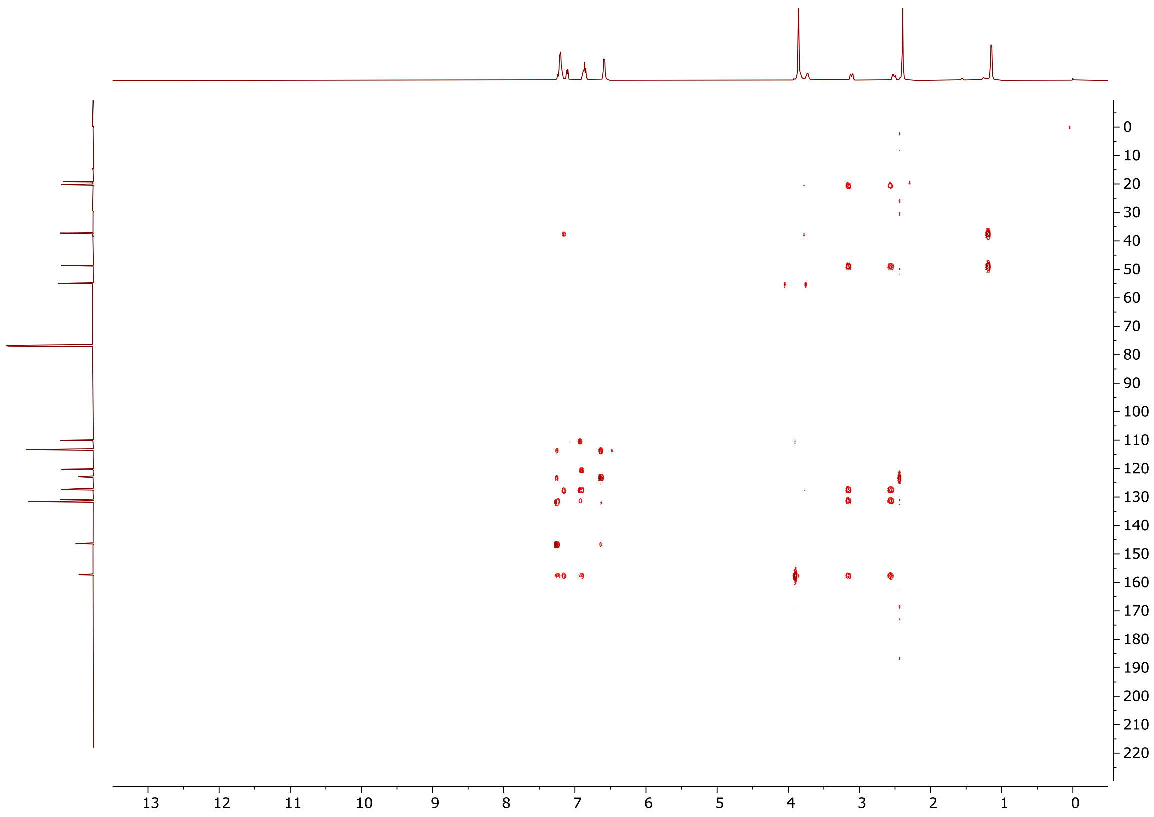

Figure S252: ^{1}H-^{13}C HMBC NMR Spectrum of 8u in $CDCl_3$ after isolation via column chromatography.

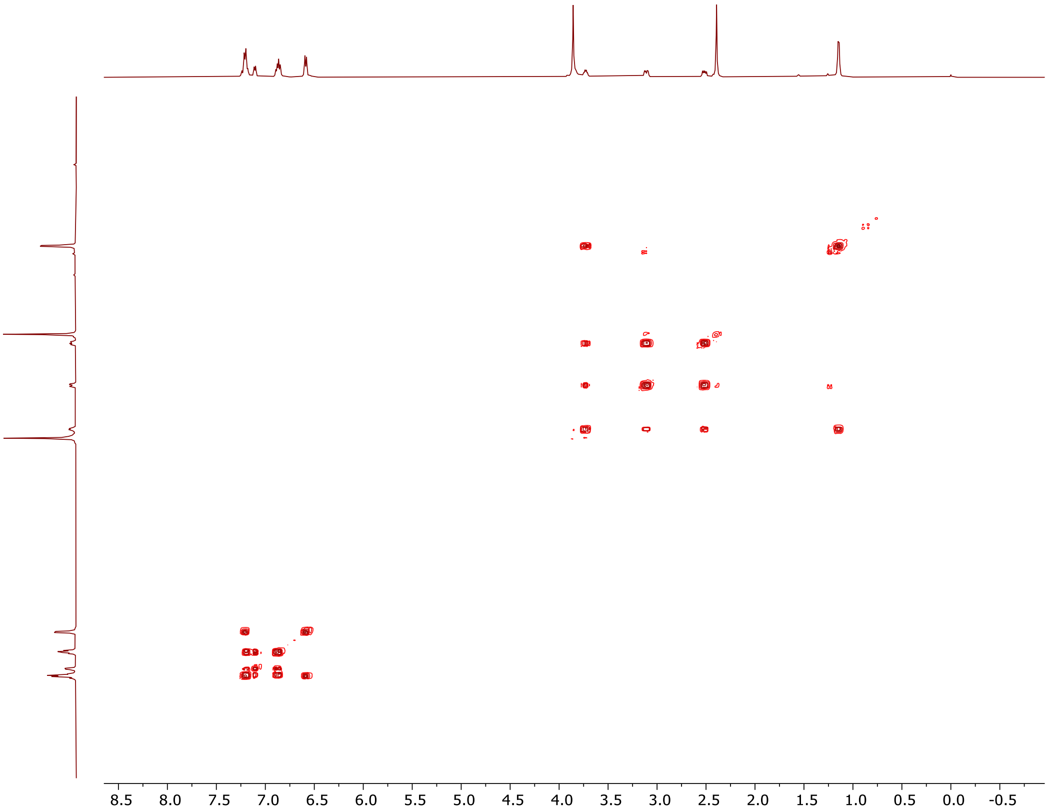

Figure S253: ^1H-^1H COSY NMR Spectrum of 8u in $CDCl_3$ after isolation via column chromatography.

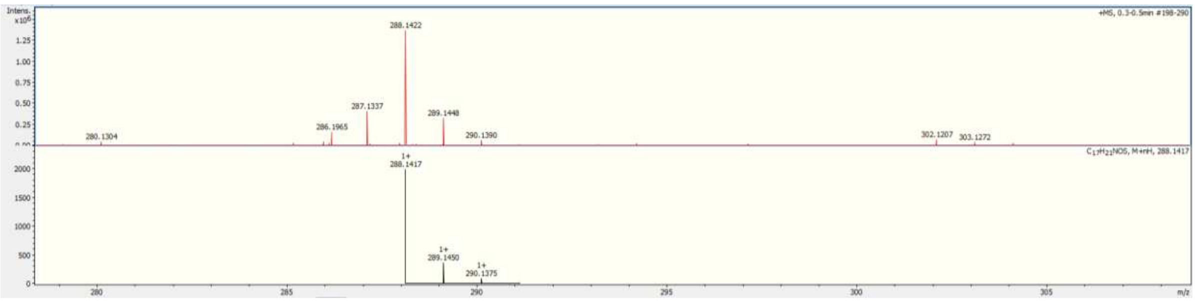

Figure S254: HRMS spectra for compound 8u.

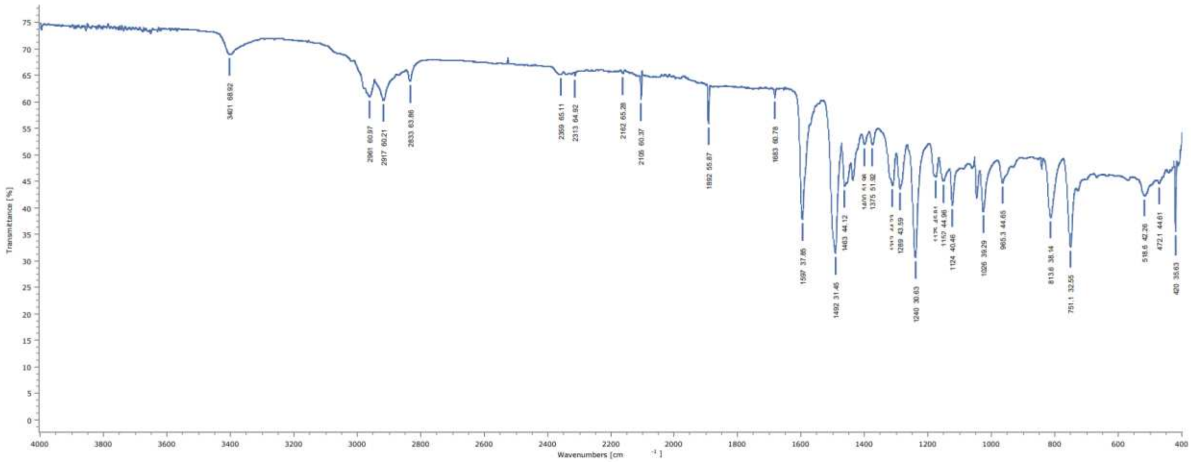

Figure S255: IR spectra for compound 8u.

11.4.21. 4-(methylthio)-N-(1-phenylpropan-2-yl)aniline **8v**

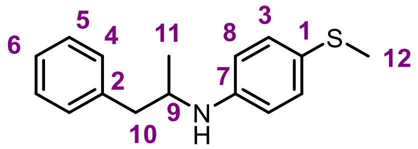

Light yellow oil (110 mg, 43%)

R_f = 0.40 (50% DCM / 50% hexane)

^{1}H NMR (CDCl$_3$, 500 MHz) δ 7.37 (app. t, J = 7.4 Hz, 2H, C^5-H), 7.32 – 7.28 (m, 1H, C^6-H), 7.30 (d, J = 8.6 Hz, C^3), 7.25 (d, J = 7.2 Hz, C^4-H), 6.64 (d, J = 8.6 Hz, C^8-H), 3.85 – 3.79 (app. hex, J = 6.3 Hz, 1H, C^9-H), 3.83 (br s, 1H, NH), 2.99 (dd, J = 13.4, 4.7 Hz, 1H, C^{10}-H), 2.78 (dd, J = 13.4, 7.2 Hz, 1H, C^{10}-H), 2.49 (s, 3H, C^{12}-H), 1.22 (d, J = 6.4 Hz, 3H, C^{11}-H).

^{13}C{^{1}H} NMR (CDCl$_3$, 126 MHz) δ 146.2 (C^1), 138.5 (C^2), 131.8 (C^3), 129.6 (C^4), 128.5 (C^5), 126.5 (C^6), 124.1 (C^7), 114.0 (C^8), 49.5 (C^9), 42.4 (C^{10}), 20.3 (C^{11}), 19.4 (C^{12}).

HRMS (ESI+): calcd for [M, $C_{16}H_{19}NS$]$^+$ 258.1311, found 258.1309.

IR (Neat): 3399, 3025, 1596, 1495, 812, 698 cm^{-1}.

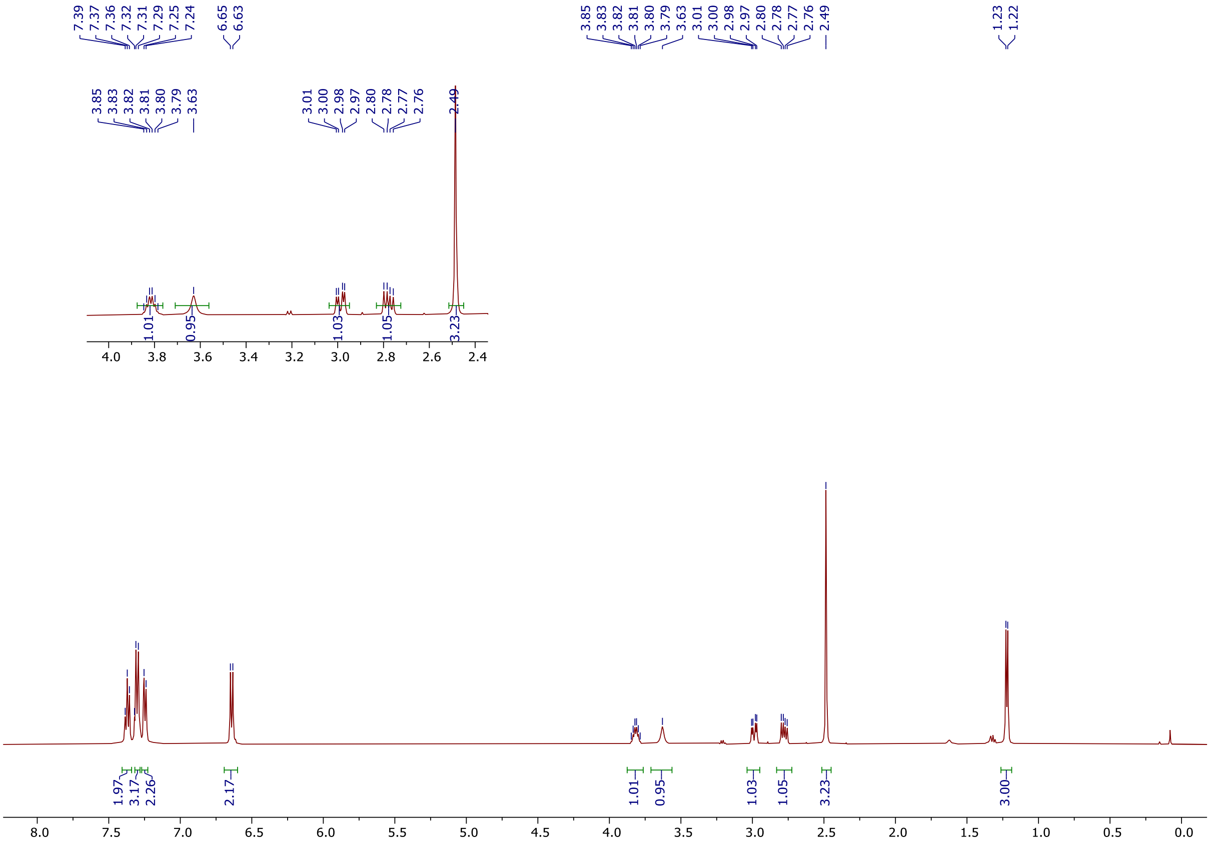

Figure S256: ^{1}H NMR Spectrum of 8v in CDCl$_3$ after isolation via column chromatography.

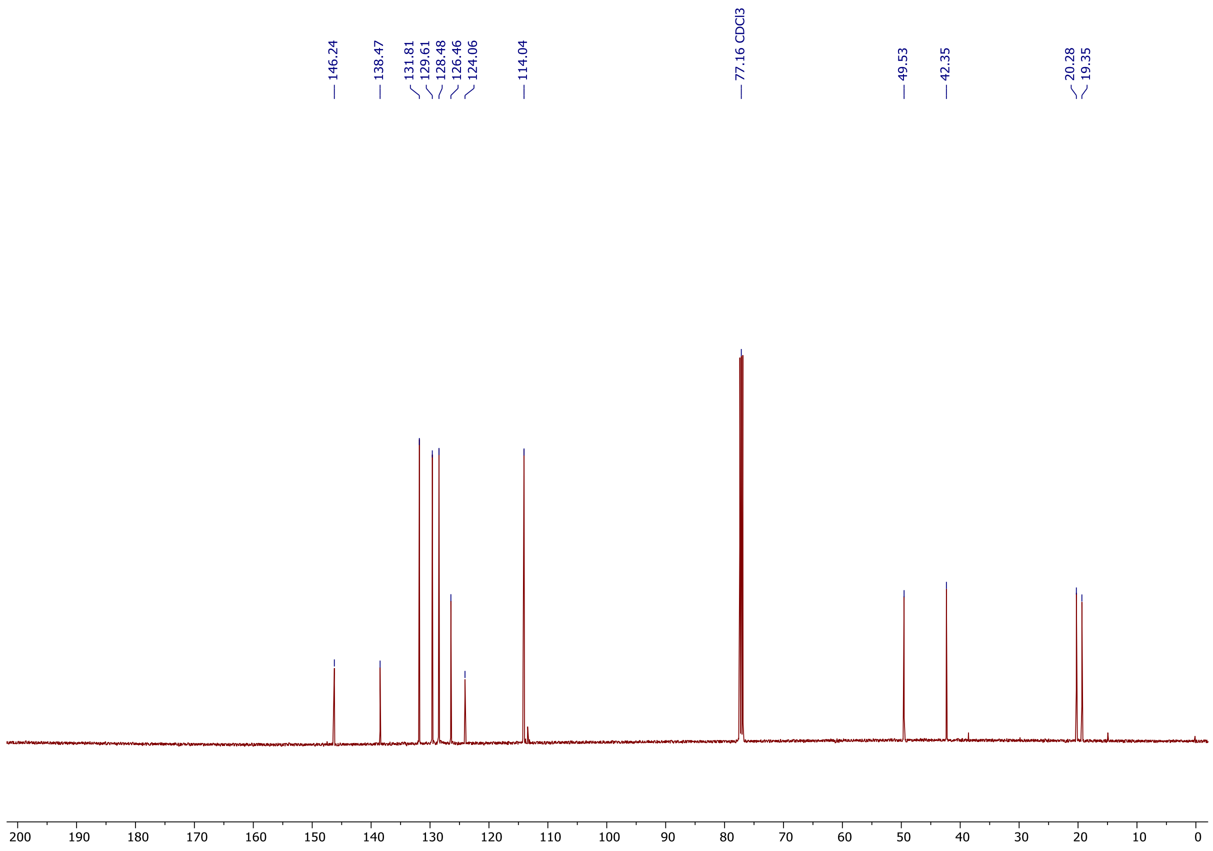

*Figure S257: [13]*C NMR Spectrum of 8v in $CDCl_3$ after isolation via column chromatography.

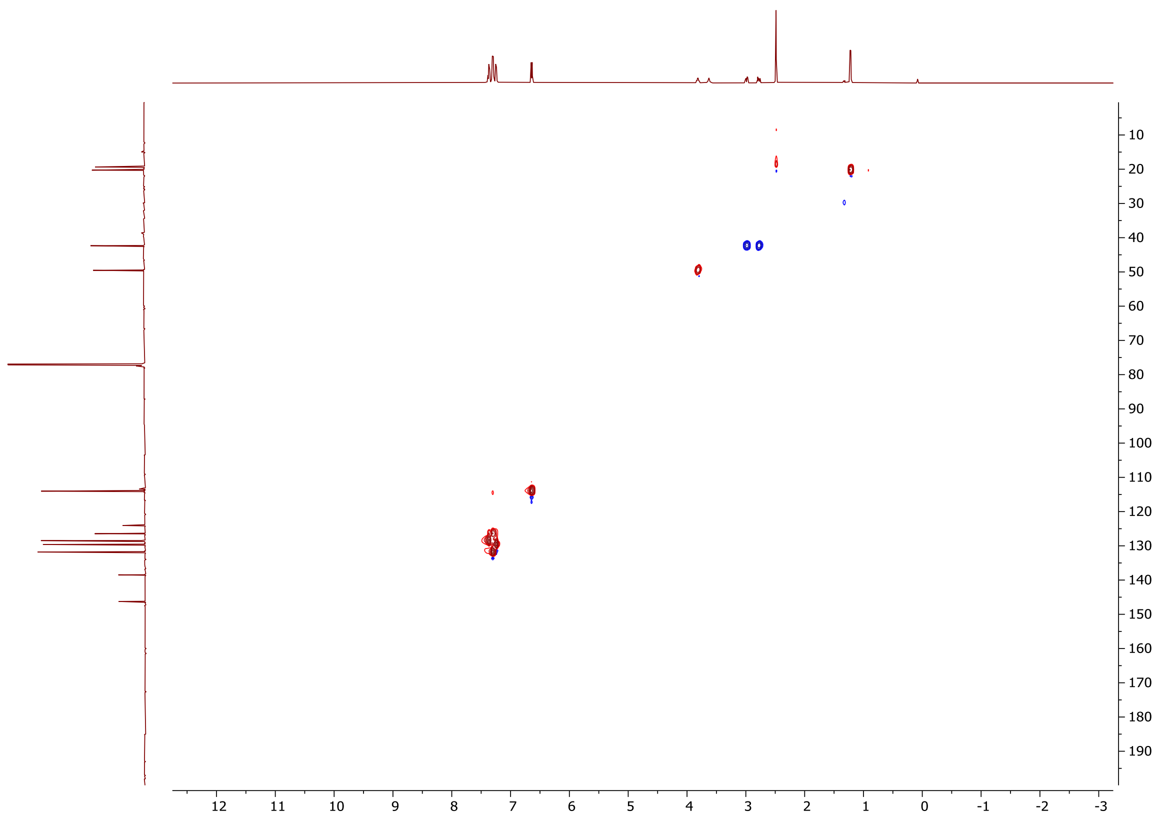

*Figure S258: ^{1}H-^{13}C HSQ*C NMR Spectrum of 8v in $CDCl_3$ after isolation via column chromatography.

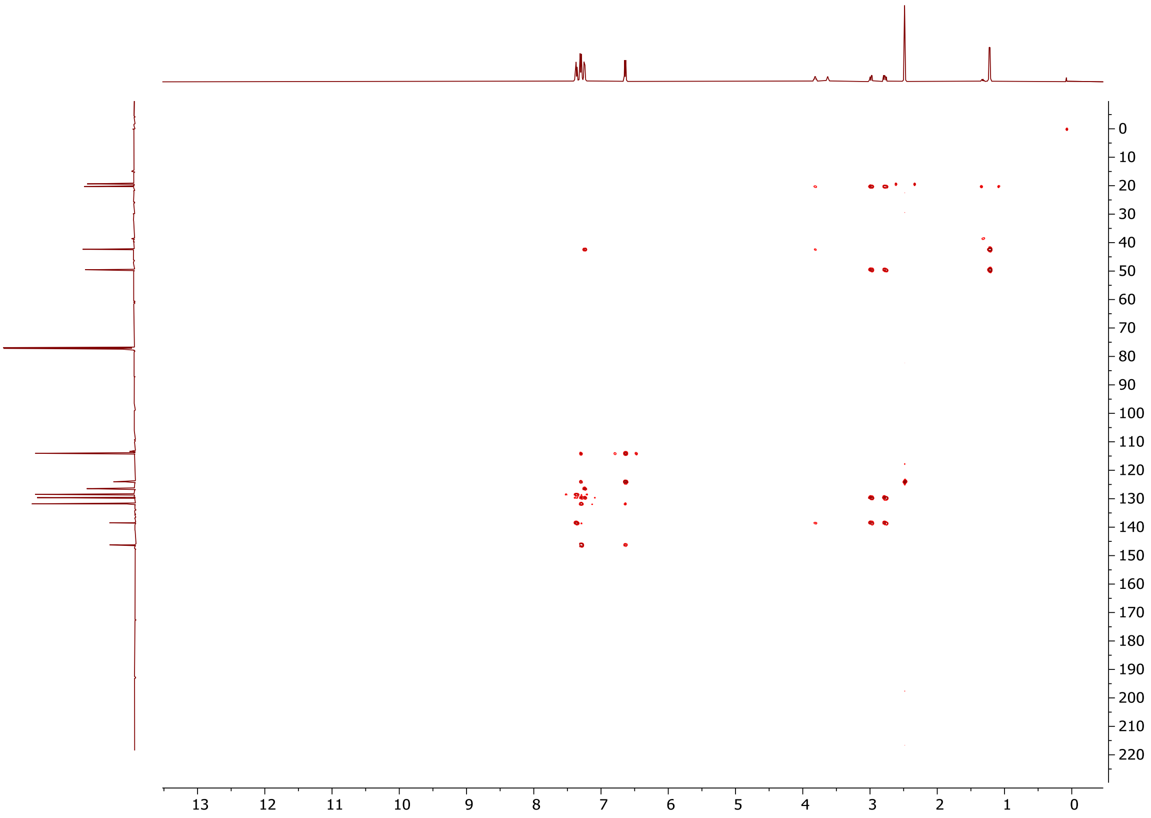

Figure S259: ^{1}H-^{13}C HMBC NMR Spectrum of 8v in $CDCl_3$ after isolation via column chromatography.

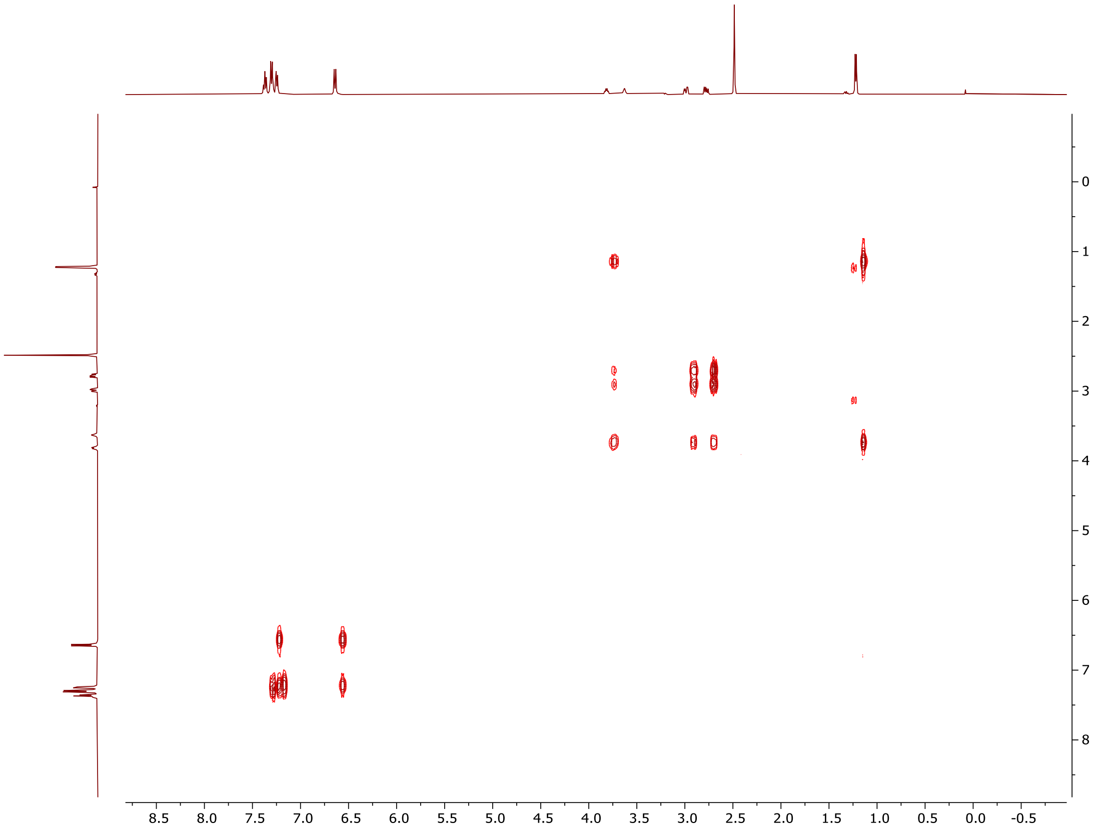

Figure S260: ^{1}H-^{1}H COSY NMR Spectrum of 8v in $CDCl_3$ after isolation via column chromatography.

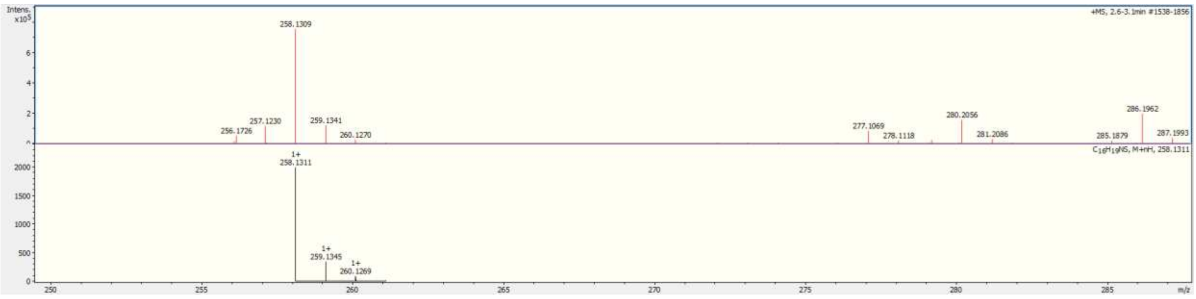

Figure S261: HRMS spectra for compound 8v.

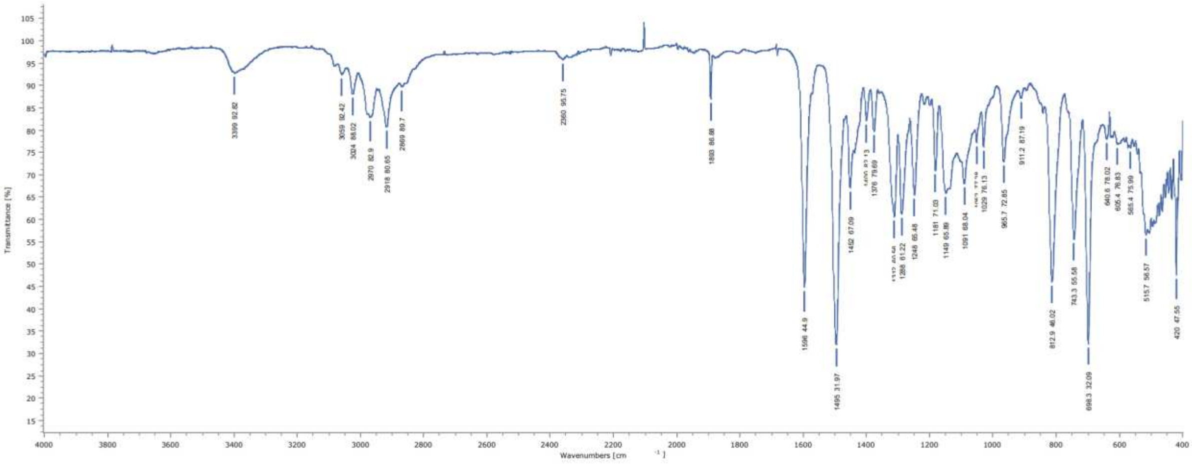

Figure S262: IR spectra for compound 8v.

11.4.22. N-(4-(trifluoromethyl)phenyl)-2,3-dihydro-1H-inden-1-amine **8w**[18]

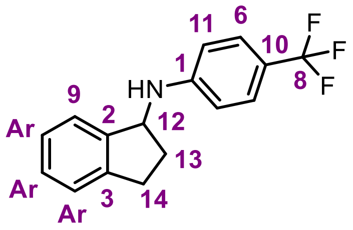

Required an additional zinc reduction step.

Colourless oil (165 mg, 60%)

R_f = 0.30 (30% DCM / 70% hexane)

Analytic data is in accordance with those reported in literature.

^{1}H NMR ($CDCl_3$, 500 MHz) δ 7.51 (app. d, J = 8.6 Hz, 2H, C^6-H), 7.42 (d, J = 7.4 Hz, 1H, C^9-H), 7.37 – 7.27 (m, 3H, Ar-H), 6.78 (app. d, J = 8.6 Hz, 2H, C^{11}-H), 5.12 (app. q, J = 7.1 Hz, 1H, C^{12}-H), 4.31 (*br* d, J = 7.3 Hz, 1H, NH), 3.12 (ddd, J = 15.9, 8.6, 4.4 Hz, 1H, C^{14}-H), 3.00 (app. dt, J = 15.9, 7.8 Hz, 1H, C^{14}-H), 2.71 – 2.65 (m, 1H, C^{13}-H), 2.03 – 1.96 (m, 1H, C^{13}-H).

^{13}C{^{1}H} NMR ($CDCl_3$, 126 MHz) δ 150.3 (C^1), 143.8 (C^2), 143.7 (C^3), 128.4 (Ar), 127.0 (Ar), 126.9 (q, J = 3.6 Hz, C^6), 125.2 (Ar), 125.1 (q, J = 270.3 Hz, C^8), 124.3 (C^9), 119.0 (q, 32.4 Hz, C^{10}), 112.3 (C^{11}), 58.4 (C^{12}), 33.8 (C^{13}), 30.4 (C^{14}).

^{19}F{^{1}H} NMR ($CDCl_3$, 470 MHz) δ -61.0

HRMS (ESI+): calcd for [M, $C_{16}H_{14}NF_3$]$^+$ 278.1151, found 278.1149.

IR (Neat): 3404, 2941, 1614, 1317, 1100, 1061, 822, 747 cm^{-1}.

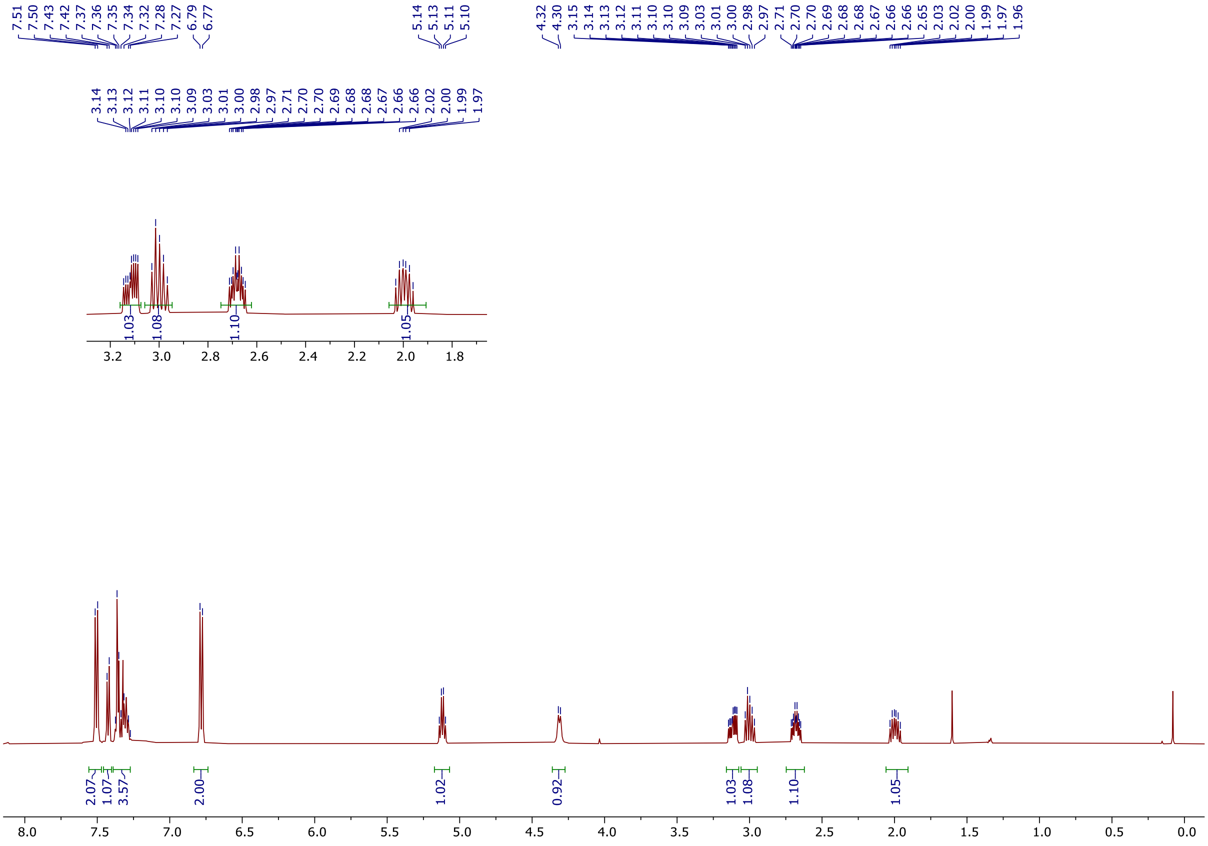

Figure S263: ^{1}H NMR Spectrum of 8w in $CDCl_3$ after isolation via column chromatography.

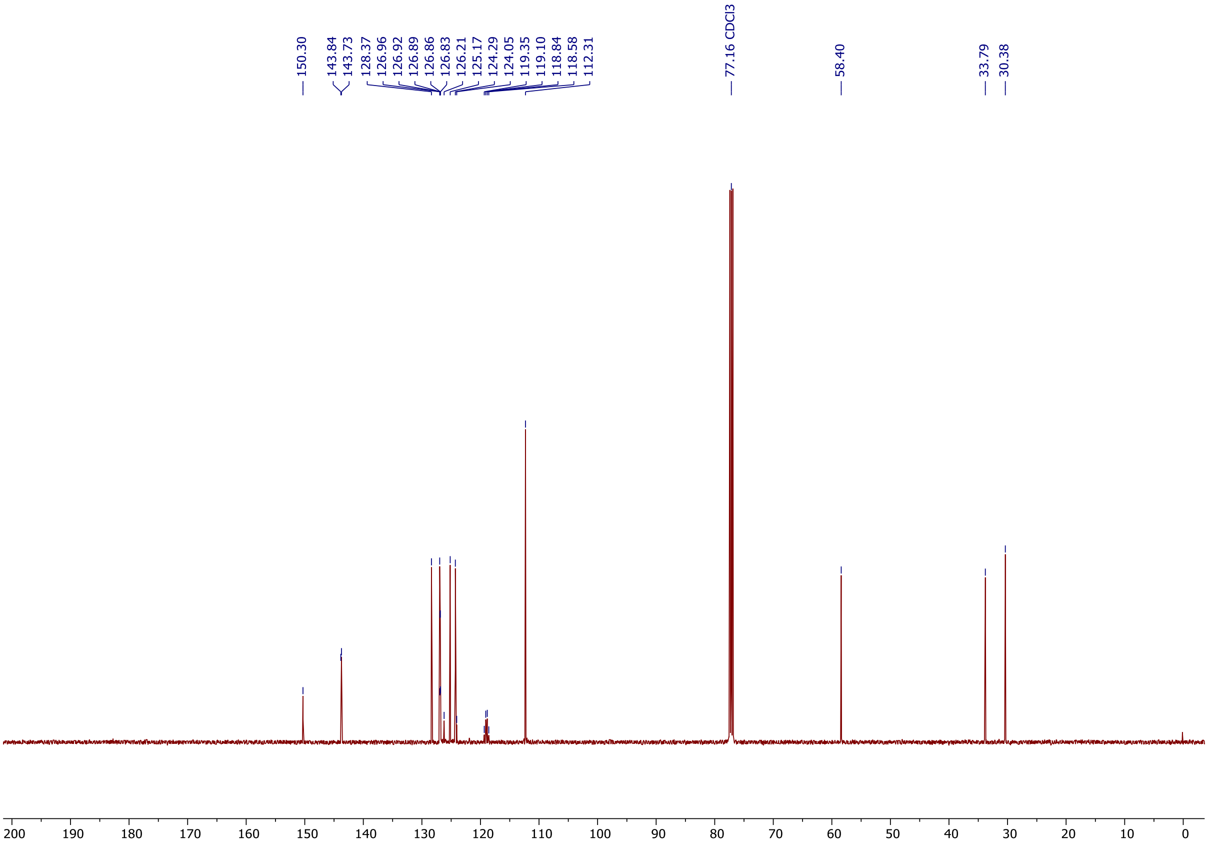

Figure S264: ^{13}C NMR Spectrum of 8w in $CDCl_3$ after isolation via column chromatography.

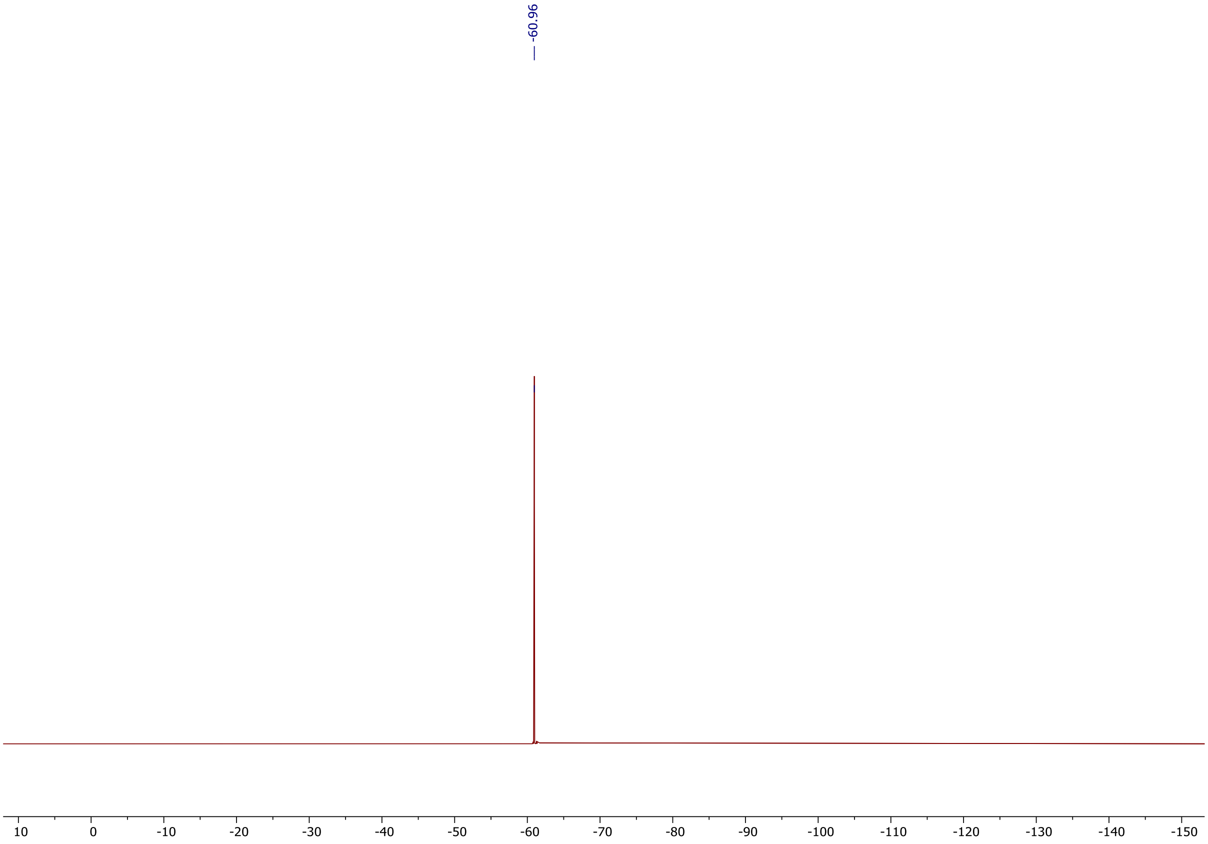

Figure S265: ^{19}F NMR Spectrum of 8w in $CDCl_3$ after isolation via column chromatography.

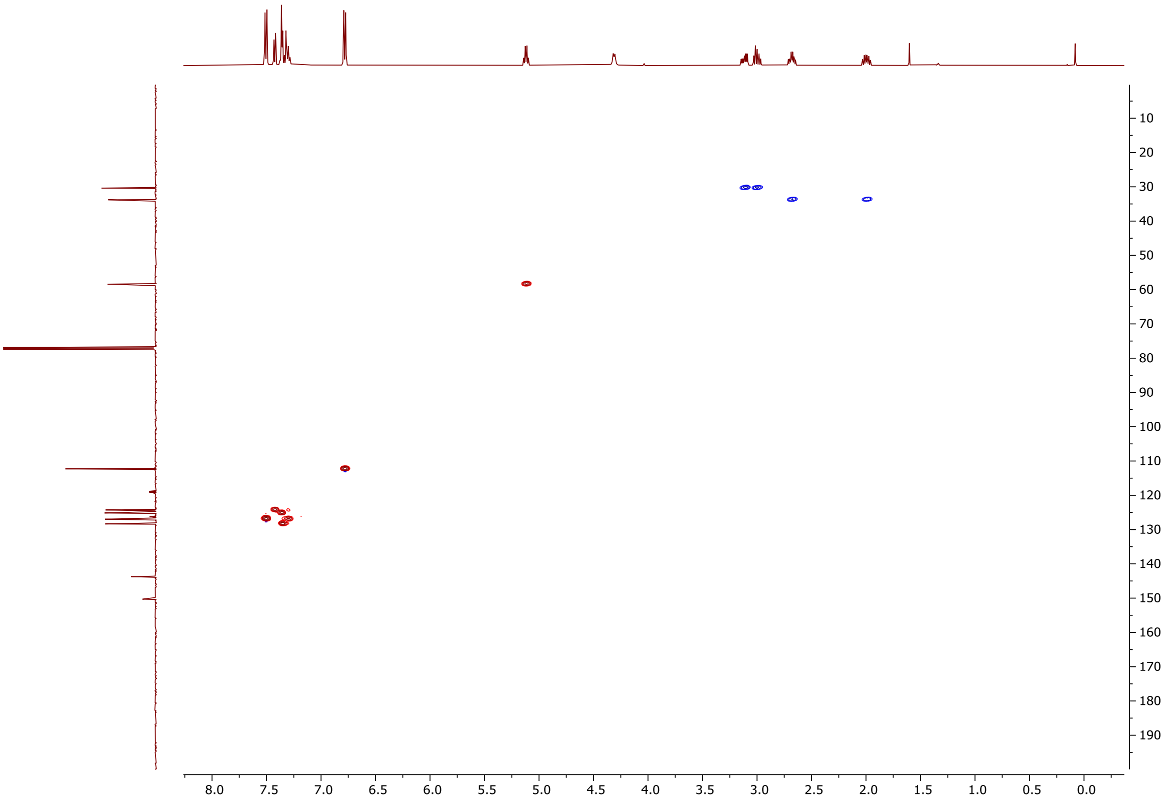

Figure S266: ^{1}H-^{13}C *HSQC* NMR Spectrum of 8w in $CDCl_3$ after isolation via column chromatography.

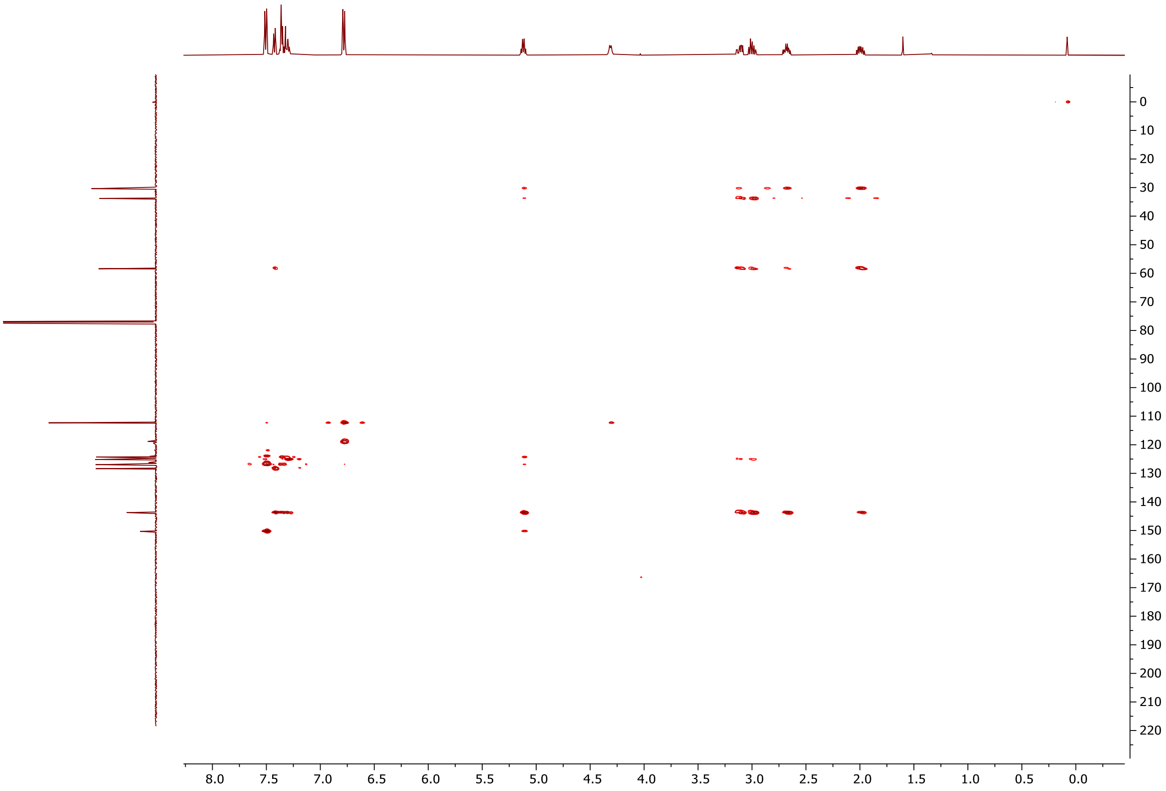

Figure S267: ^{1}H-^{13}C HMBC NMR Spectrum of 8w in $CDCl_3$ after isolation via column chromatography.

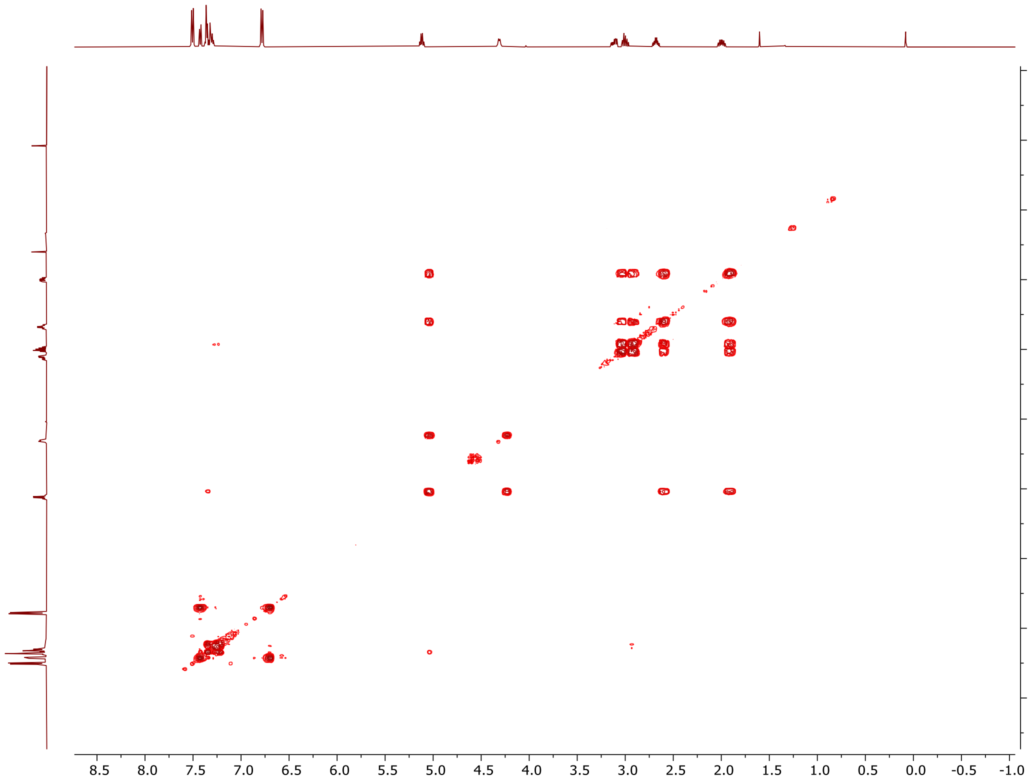

Figure S268: ^{1}H-^{1}H COSY NMR Spectrum of 8w in $CDCl_3$ after isolation via column chromatography.

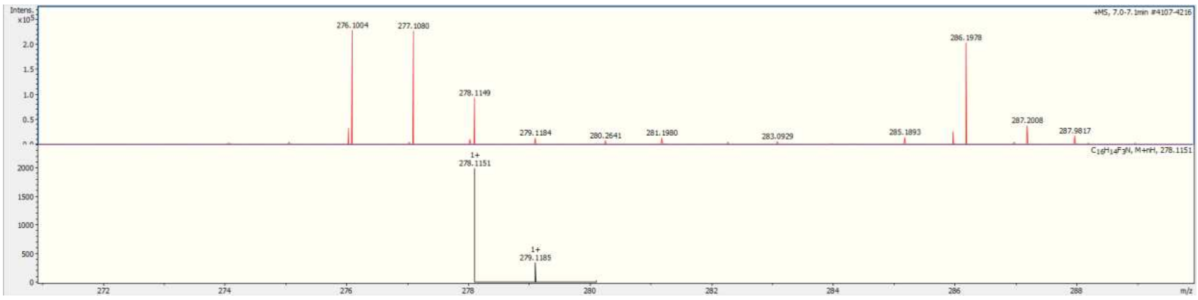

Figure S269: HRMS spectra for compound 8w.

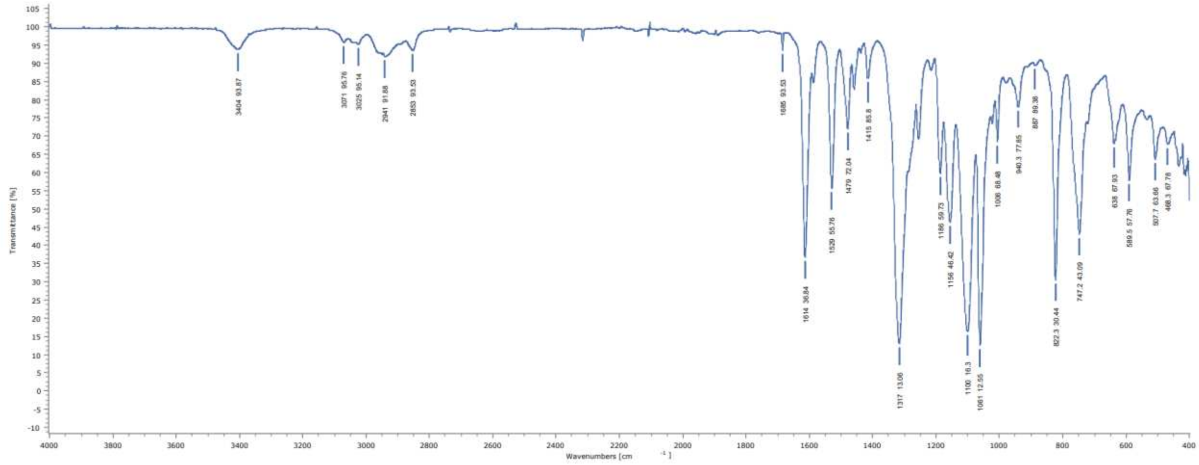

Figure S270: IR spectra for compound 8w.

11.4.23. N-(1-phenylpropan-2-yl)-4-(trifluoromethyl)aniline **8x**

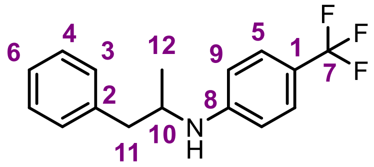

Colourless oil (59 mg, 22%)

R_f = 0.63 (50% DCM / 50% hexane)

^{1}H NMR ($CDCl_3$, 500 MHz) δ 7.40 (d, J = 8.5 Hz, 2H, C^5-H), 7.31 (t, J = 7.5 Hz, 2H, C^4-H), 7.24 (app. t, J = 7.3 Hz, 1H, C^6-H), 7.17 (d, J = 6.8 Hz, 2H, C^3-H), 6.60 (d, J = 8.5 Hz, 2H, C^9-H), 3.85 (br s, 1H, NH), 3.84 – 3.78 (m, 1H, C^{10}-H), 2.91 (dd, J = 13.6, 4.8 Hz, 1H, C^{11}-H), 2.76 (dd, J = 13.6, 6.8 Hz, 1H, C^{11}-H), 1.18 (d, J = 6.2 Hz, 3H, C^{12}-H).

^{13}C{^{1}H}{^{19}F} NMR ($CDCl_3$, 126 MHz) δ 149.8 (C^8), 138.1 (C^2), 129.6 (C^3), 128.6 (C^4), 126.9 (C^5), 126.7 (C^6), 125.1 (C^7), 118.7 (C^1), 112.4 (C^9), 49.2 (C^{10}), 42.3 (C^{11}), 20.2 (C^{12}).

^{19}F{^{1}H} NMR ($CDCl_3$, 470 MHz) δ -61.0.

HRMS (ESI+): calcd for [M, $C_{16}H_{16}NF_3$]$^+$ 280.1308, found 280.1320.

IR (Neat): 3063, 2980, 1615, 1318, 1103, 822 cm^{-1}.

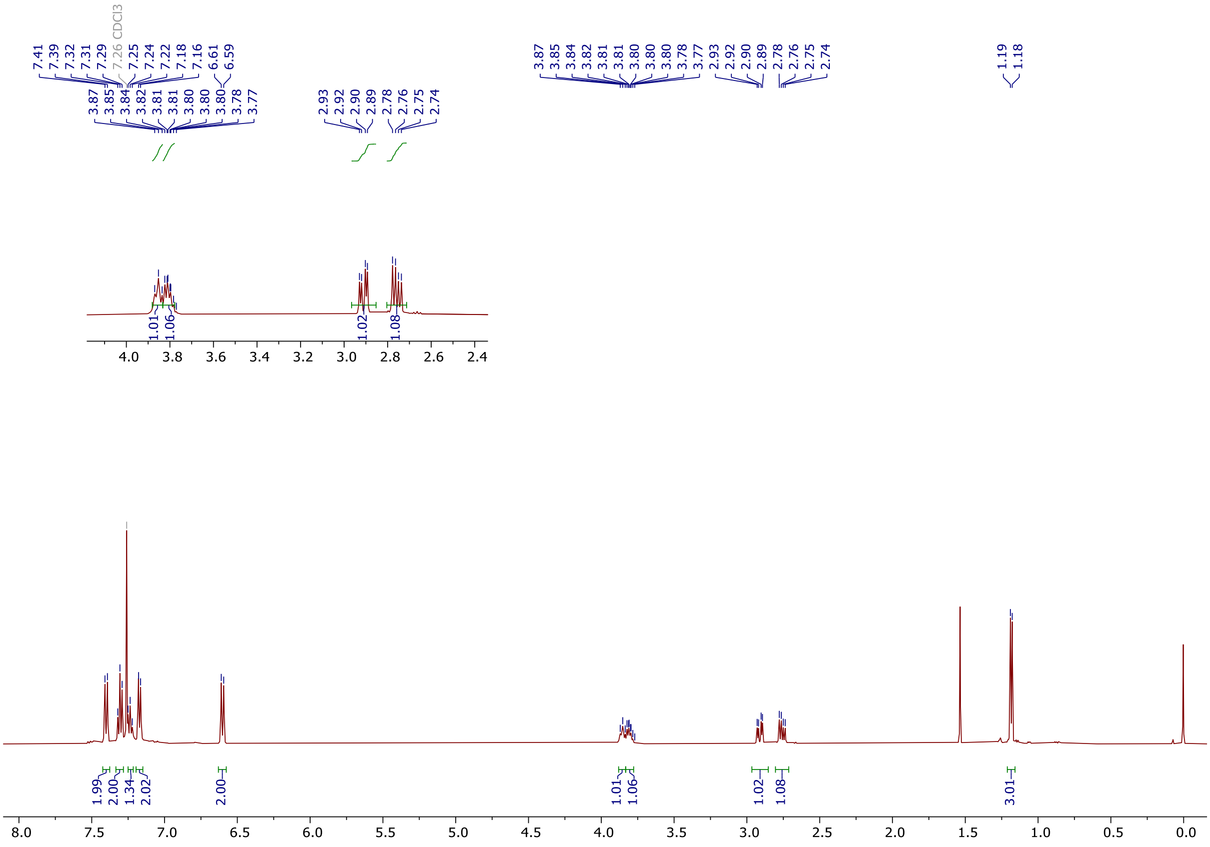

Figure S271: ^{1}H NMR Spectrum of 8x in $CDCl_3$ after isolation via column chromatography.

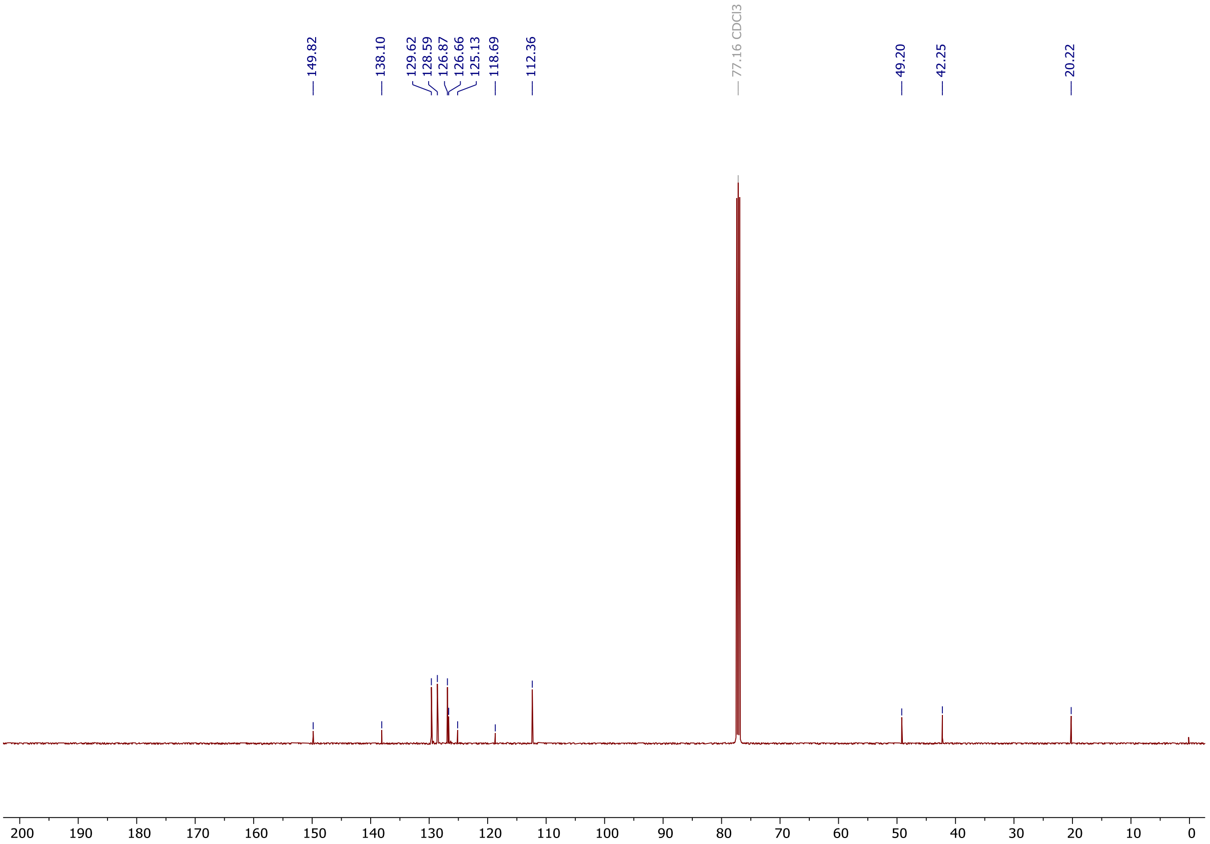

Figure S272: ^{13}C NMR Spectrum of 8x in $CDCl_3$ after isolation via column chromatography.

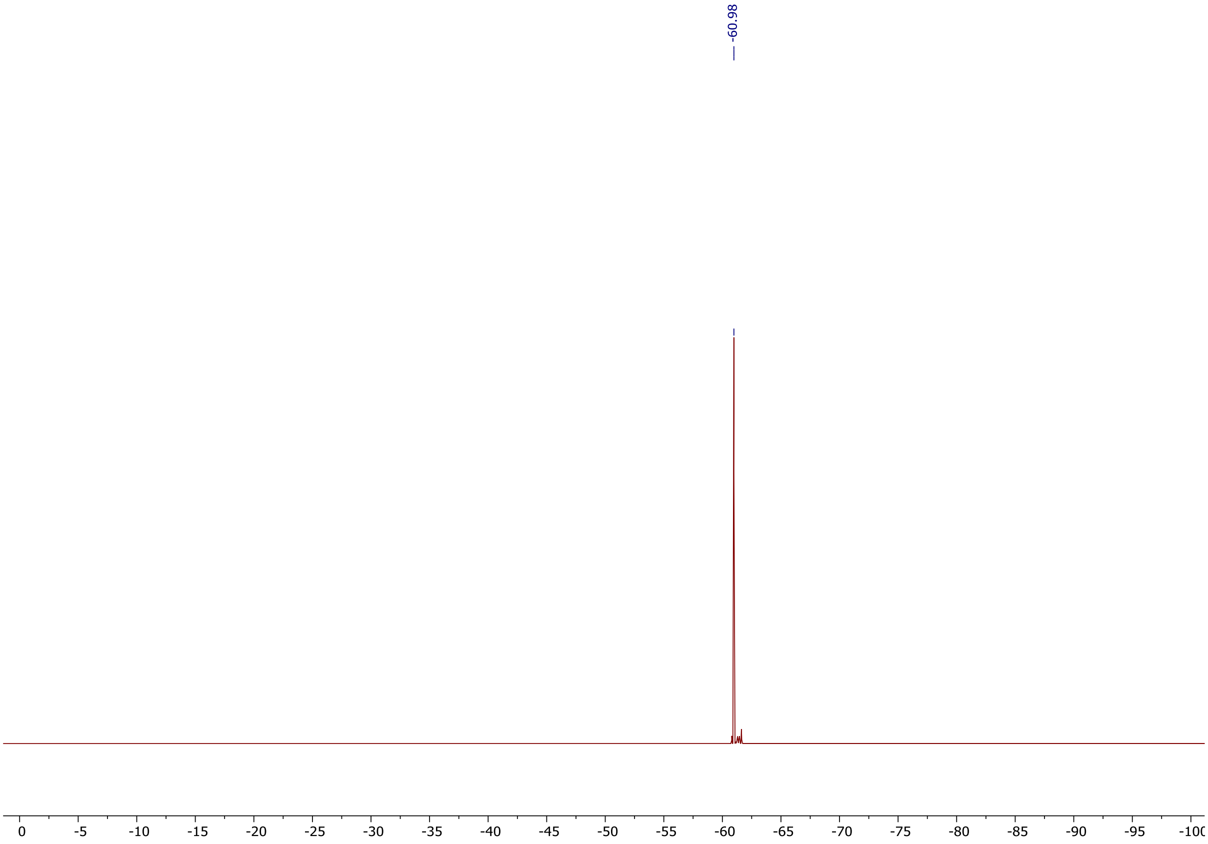

Figure S273: ^{19}F NMR Spectrum of 8x in $CDCl_3$ after isolation via column chromatography.

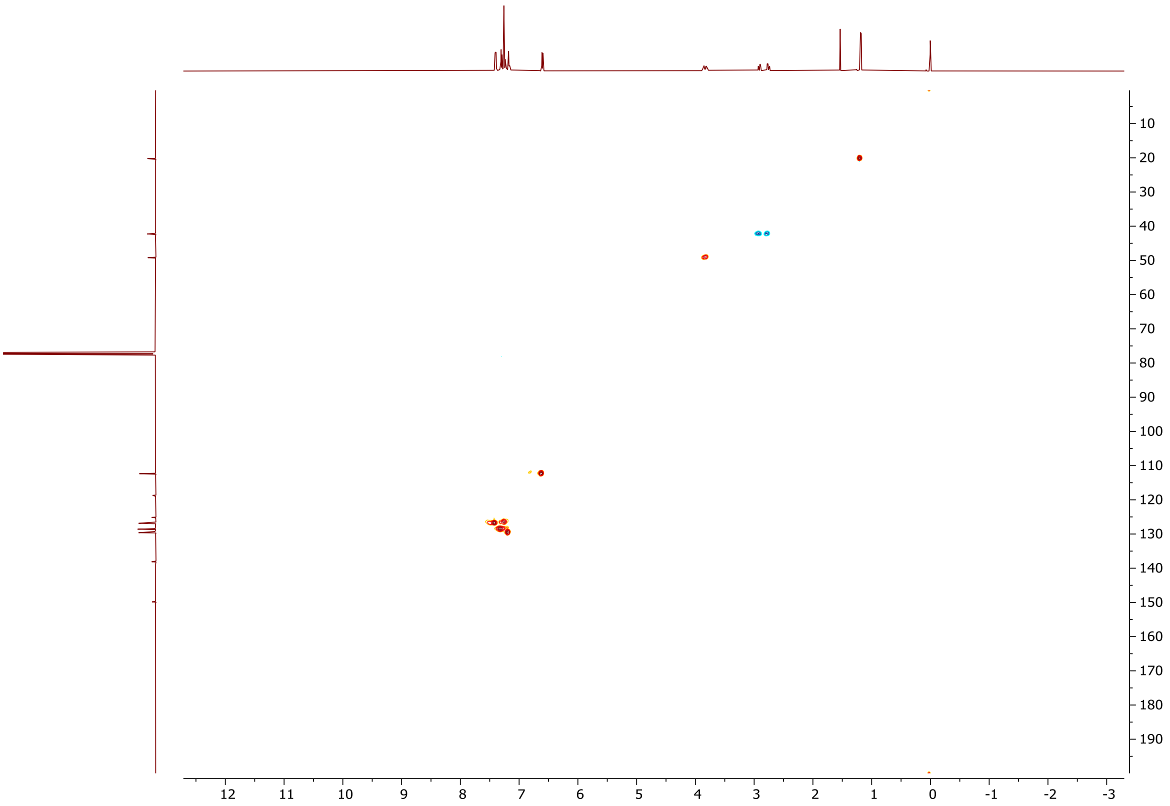

Figure S274: ^{1}H-^{13}C *HSQC NMR spectrum of* 8x in $CDCl_3$ after isolation via column chromatography.

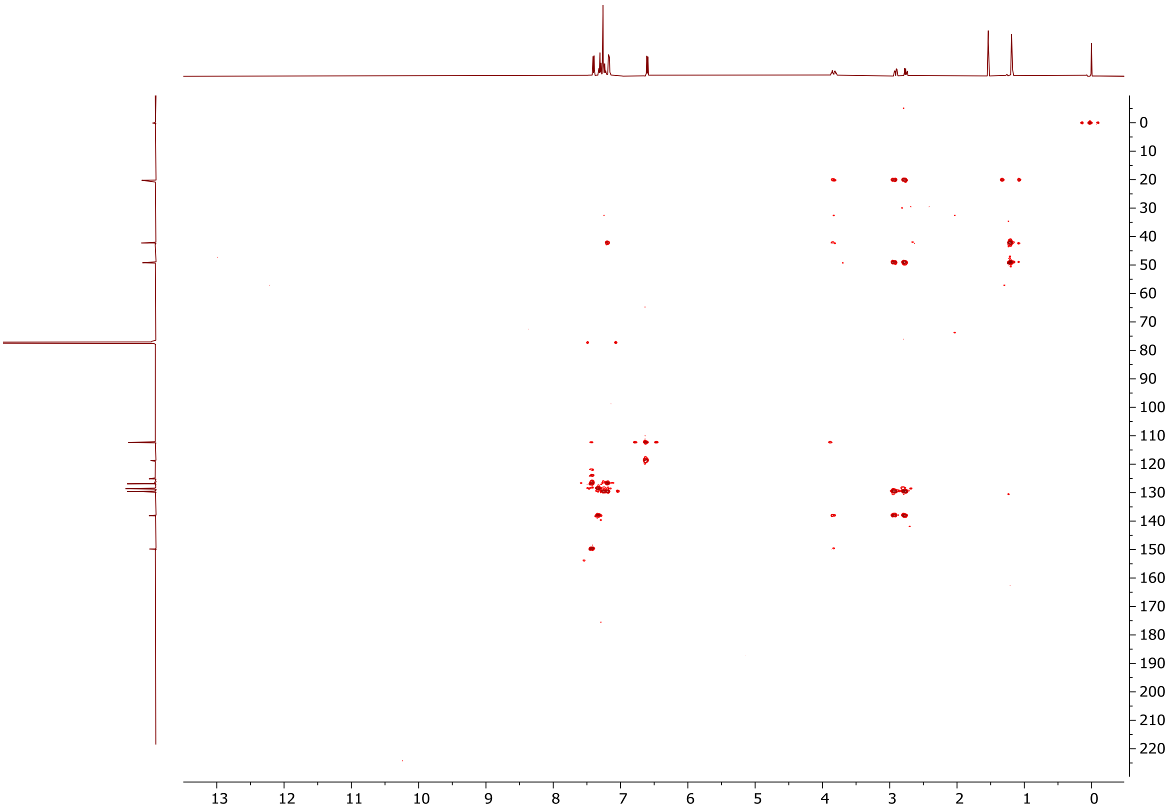

Figure S275: ^{1}H-^{13}C HMBC NMR spectrum of 8x in $CDCl_3$ after isolation via column chromatography.

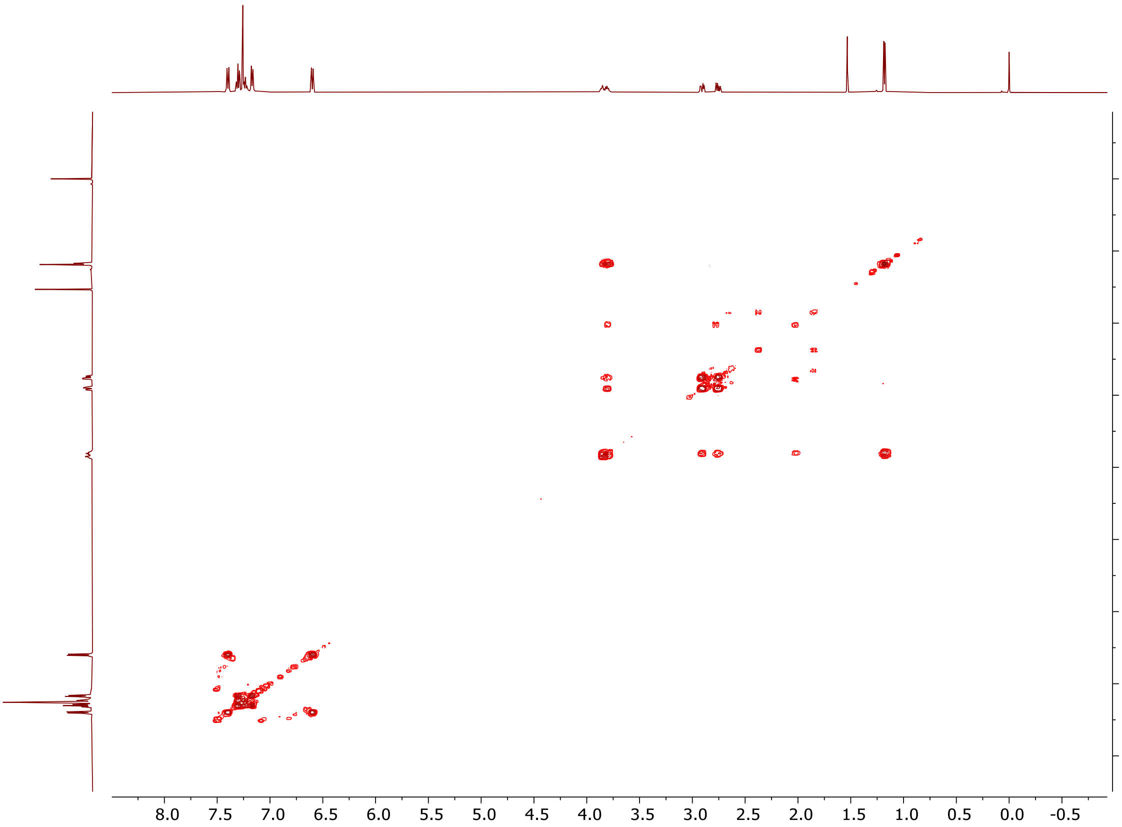

Figure S276: ^{1}H-^{1}H COSY NMR spectrum of 8x in $CDCl_3$ after isolation via column chromatography.

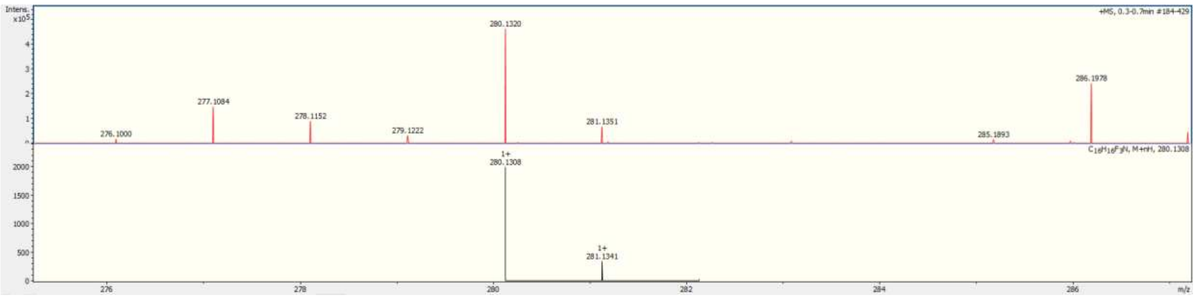

Figure S277: HRMS spectra for compound 8x.

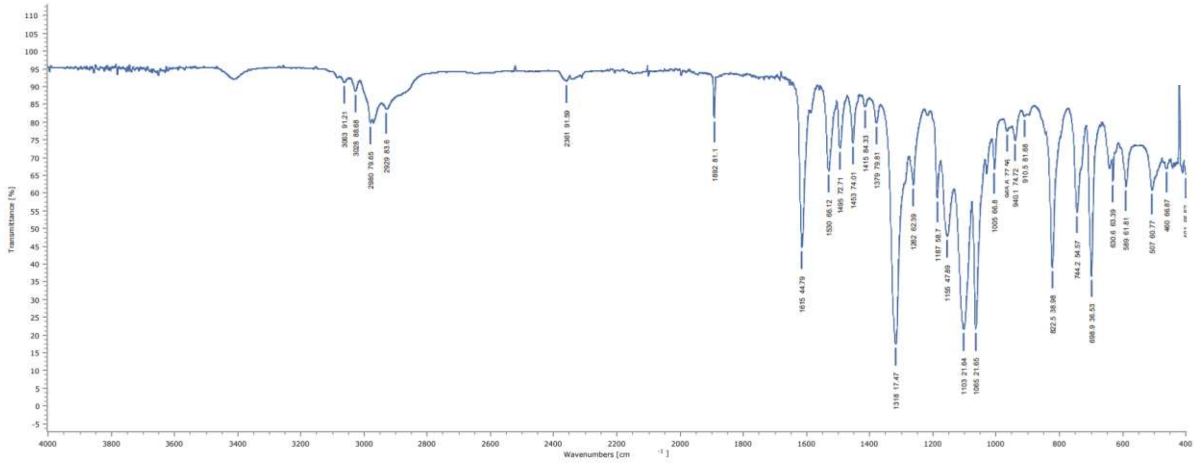

Figure S278: IR spectra for compound 8x.

11.4.24. N-(2,3-dihydro-1H-inden-1-yl)-2-methylnaphthalen-1-amine **8y**

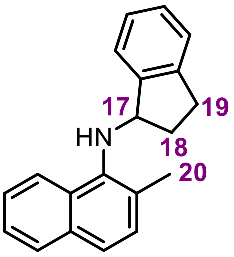

Colourless oil (44.5 mg, 16%)

R_f = 0.20 (30% DCM / 70% hexane)

^{1}H NMR ($CDCl_3$, 500 MHz) δ 8.20 (d, J = 8.4 Hz, 1H, Ar-H), 7.82 (dd, J = 8.2, 1.6 Hz, 1H, Ar-H), 7.48 (d, J = 8.2 Hz, 1H, Ar-H), 7.47 – 4.40 (m, 2H, Ar-H), 7.35 (d, J = 7.4 Hz, 1H, Ar-H), 7.29 (app. d, J = 8.3 Hz, 2H , Ar-H), 7.28 – 7.25 (m, 1H, Ar-H), 7.21 – 7.18 (m, 1H, Ar-H), 5.06 (t, J = 6.8 Hz, 1H, C^{17}), 3.69 (*br* s, 1H, NH), 3.07 (ddd, J = 15.9, 8.4, 4.5 Hz, 1H, C^{19}-H), 2.82 (dt, J = 15.8, 7.8 Hz, 1H, C^{19}-H), 2.44 (dddd, J = 12.7, 8.0, 7.2, 4.5 Hz, 1H, C^{18}-H), 2.35 (s, 3H, C^{20}), 2.01 (dddd, J = 12.8, 8.3, 7.5, 6.6 Hz, 1H, C^{18}-H).

^{13}C{^{1}H} NMR ($CDCl_3$, 126 MHz) δ 145.6 (C^1), 143.3 (C^2), 141.8 (C^3), 133.9 (C^4), 129.5 (C^5), 128.8 (C^6), 128.4 (C^7), 127.8 (C^8), 126.6 (C^9), 125.5 (C^{10}), 125.0 (C^{11}), 125.0 (C^{12}), 124.9 (C^{13}), 124.6 (C^{14}), 123.2 (C^{15}), 122.3 (C^{16}), 64.6 (C^{17}), 35.2 (C^{18}), 30.3 (C^{19}), 18.7 (C^{20}).

HRMS (ESI+): calcd for $[M, C_{20}H_{19}N]^+$ 274.1590, found 274.1592.

IR (Neat): 2954, 2923, 1458, 1374, 804, 744 cm^{-1}.

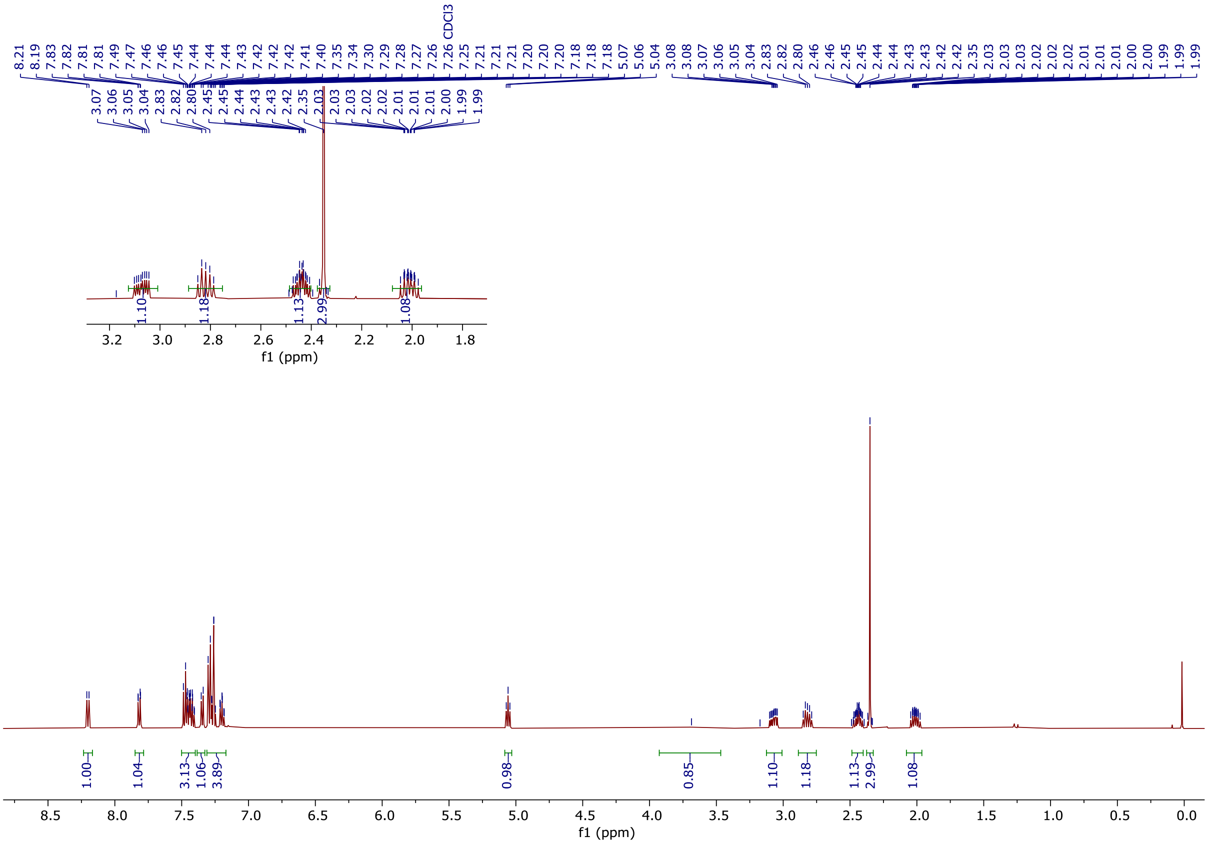

Figure S279: ¹H NMR Spectrum of 8y in $CDCl_3$ after isolation via column chromatography.

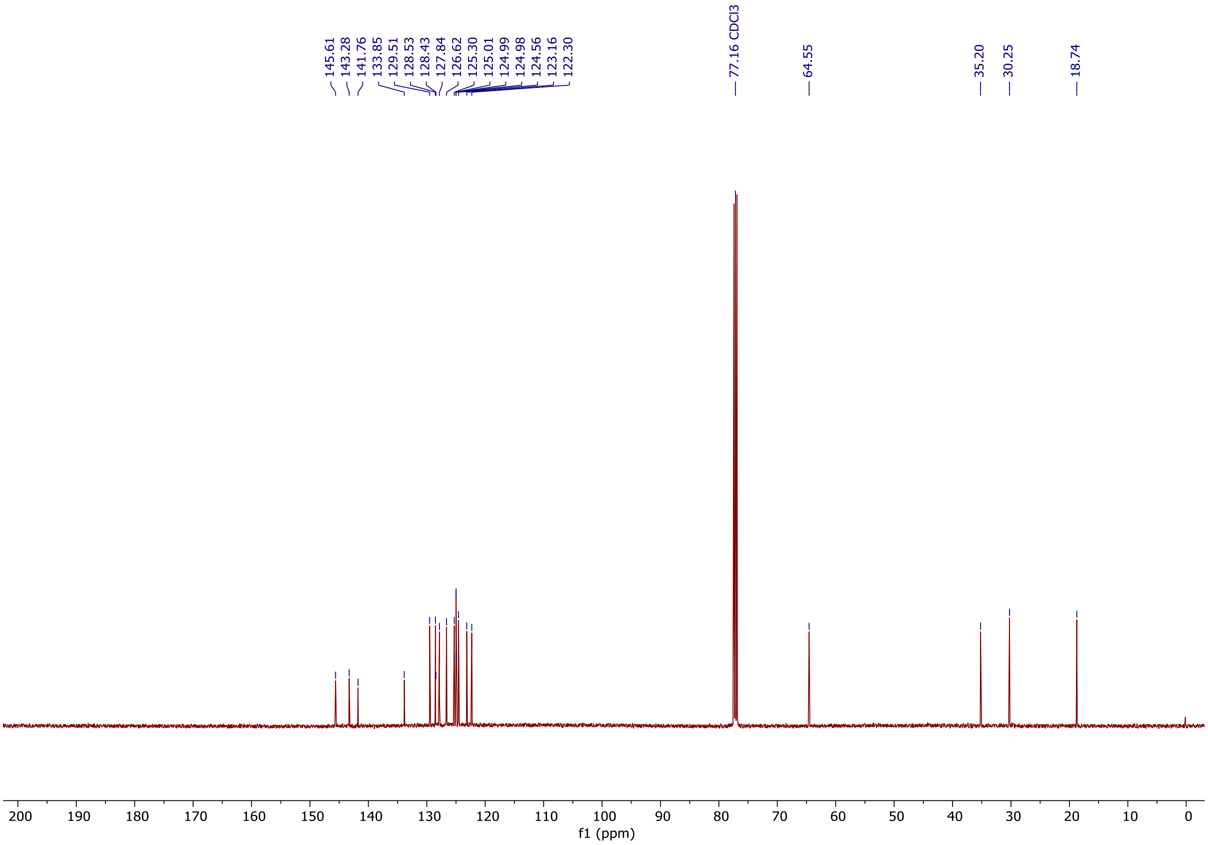

Figure S280: ¹³C NMR Spectrum of 8y in $CDCl_3$ after isolation via column chromatography.

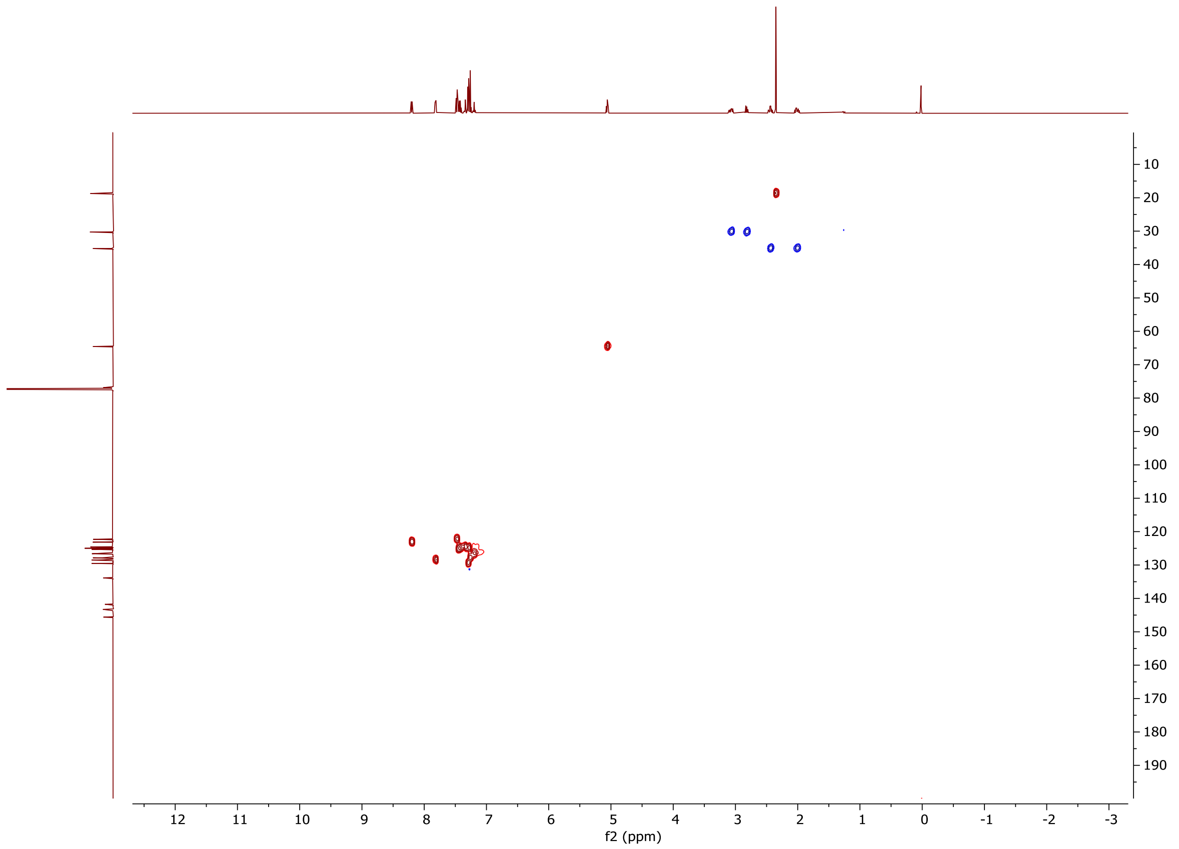

Figure S281: ¹H-¹³C HSQC NMR Spectrum of 8y in $CDCl_3$ after isolation via column chromatography.

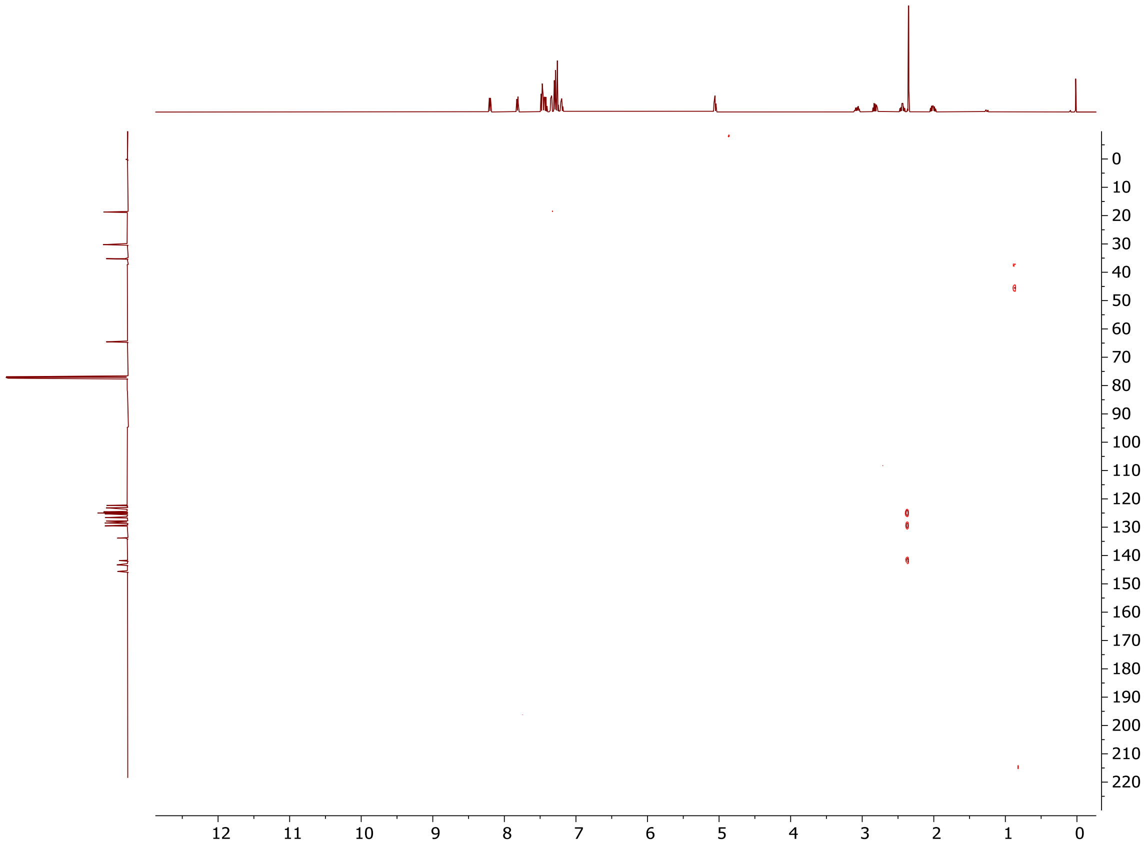

Figure S282: ¹H-¹³C HMBC NMR Spectrum of 8y in $CDCl_3$ after isolation via column chromatography.

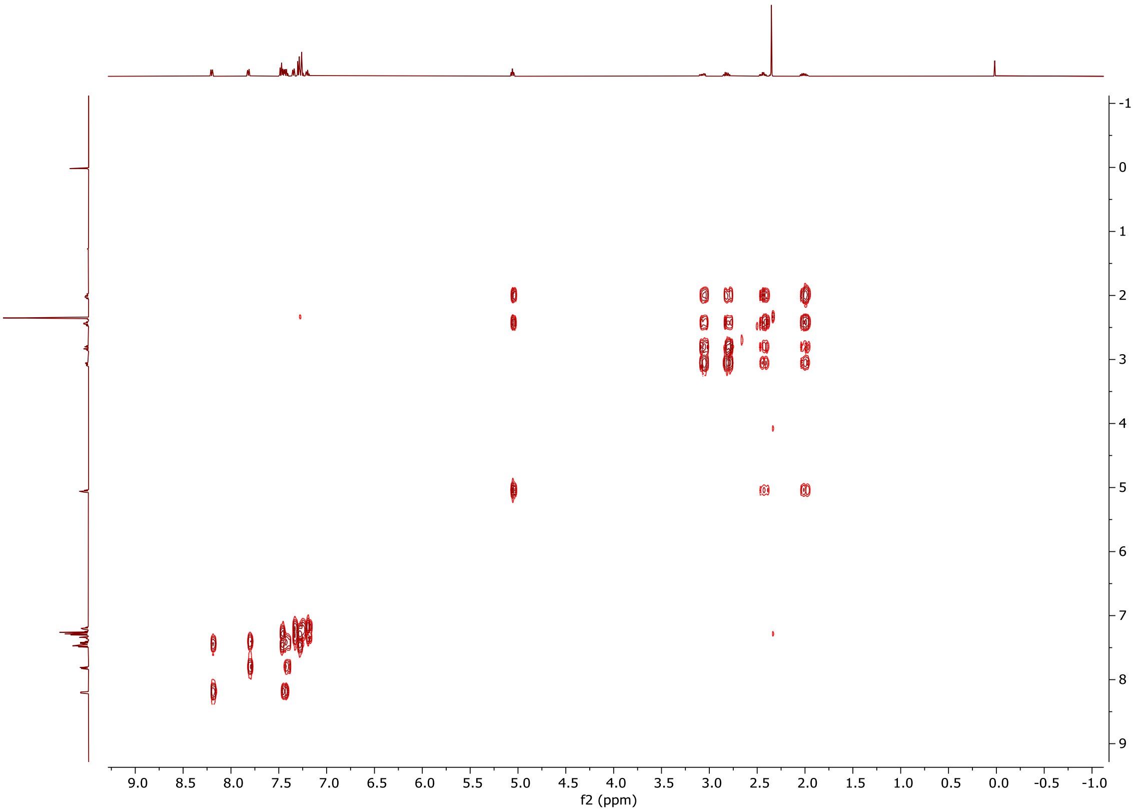

Figure S283: ^1H-^1H COSY NMR Spectrum of 8y in $CDCl_3$ after isolation via column chromatography.

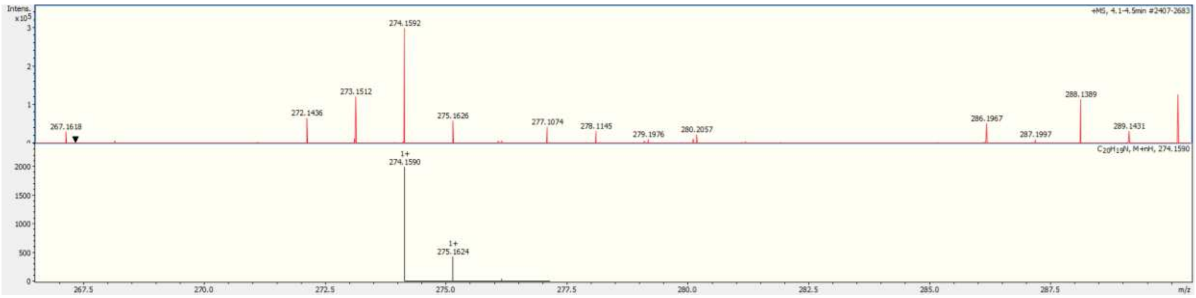

Figure S284: HRMS spectra for compound 8y.

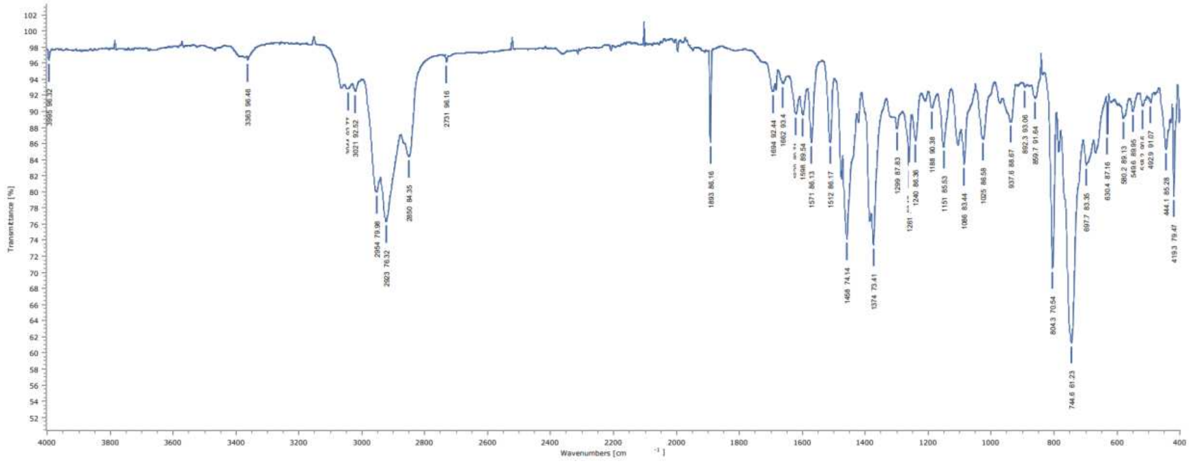

Figure S285: IR spectra for compound 8y.

11.4.25. N-phenyl-N,O-bis(2-phenylpropan-2-yl)hydroxylamine **9a**

Mixture of diastereoisomers

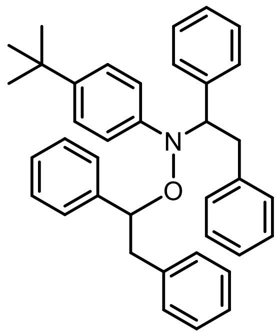

Colourless oil (74 mg, 14%)

R_f = 0.46 (30% DCM / 70% hexane)

^{1}H NMR ($CDCl_3$, 500 MHz) δ 7.15 – 7.12 (m, 4H, Ar-H), 7.08 – 6.91 (m, 32H, Ar-H), 6.84 – 6.68 (m, 12H, Ar – H), 4.45 (t, J = 7.1 Hz, 2H, CH), 4.41 – 4.33 (m, 2H, CH), 3.20 – 2.88 (m, 6H, CH_2), 2.67 – 2.56 (m, 2H, CH_2), 1.19 (app. d, J = 2.6 Hz, 18H, $(CH_3)_3$).

^{13}C{^{1}H} NMR ($CDCl_3$, 126 MHz) δ 148.2, 148.0, 147.0, 146.6, 141.2, 141.0, 139.7, 139.4, 139.3, 139.0, 138.4, 138.4, 129.9, 129.8, 129.7, 129.3, 128.1, 128.1, 128.0, 127.9, 127.9, 127.7, 127.7, 127.6, 127.6, 127.4, 127.4, 127.2, 126.6, 126.0, 126.0, 125.9, 125.8, 125.1, 125.0, 121.7, 121.0, 113.5, 86.1, 85.2, 74.4, 74.2, 42.1, 42.0, 34.4, 34.3, 31.6, 31.6.

HRMS (ESI+): calcd for [M, $C_{38}H_{39}NO$]$^+$ 526.3110, found 526.3106.

IR (Neat): 3028, 2950, 1495, 1452, 12117, 908 cm^{-1}.

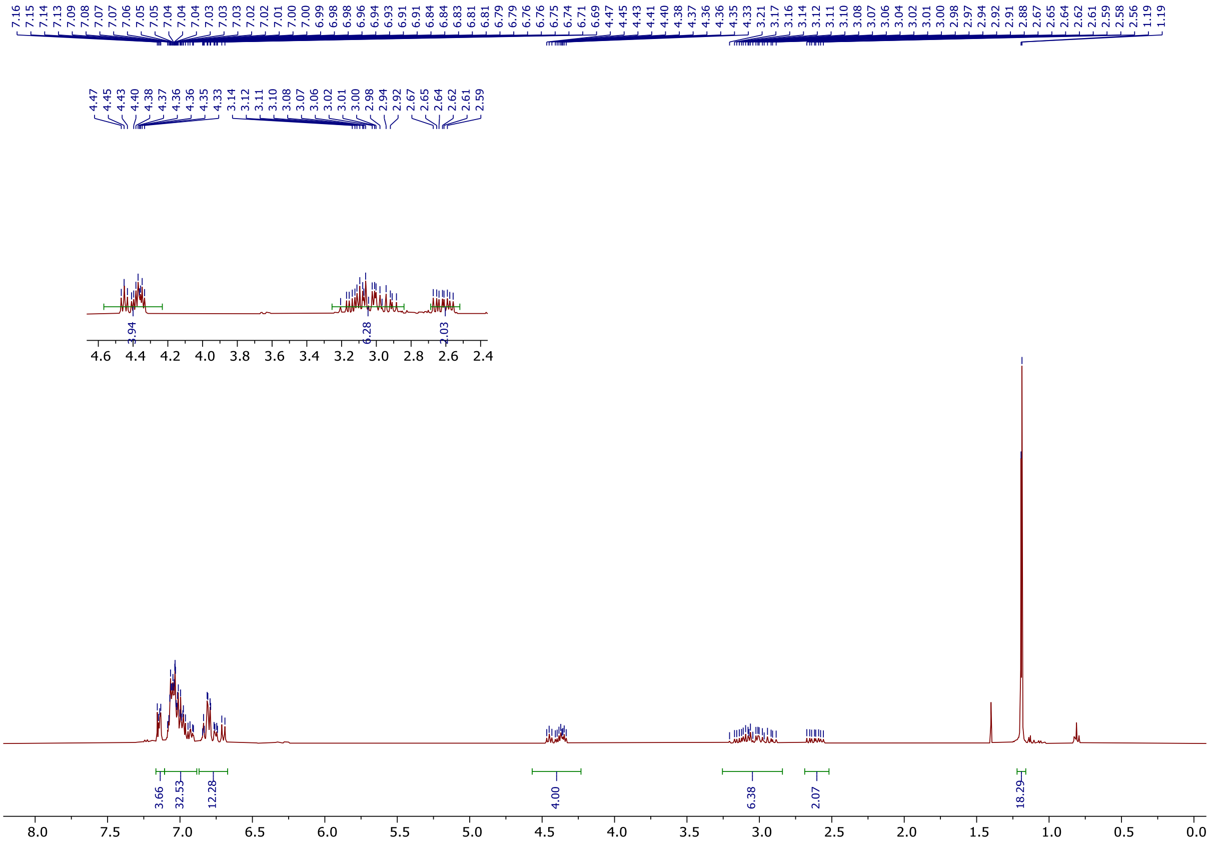

Figure S286: ^{1}H NMR Spectrum of 9a in $CDCl_3$ after isolation via column chromatography.

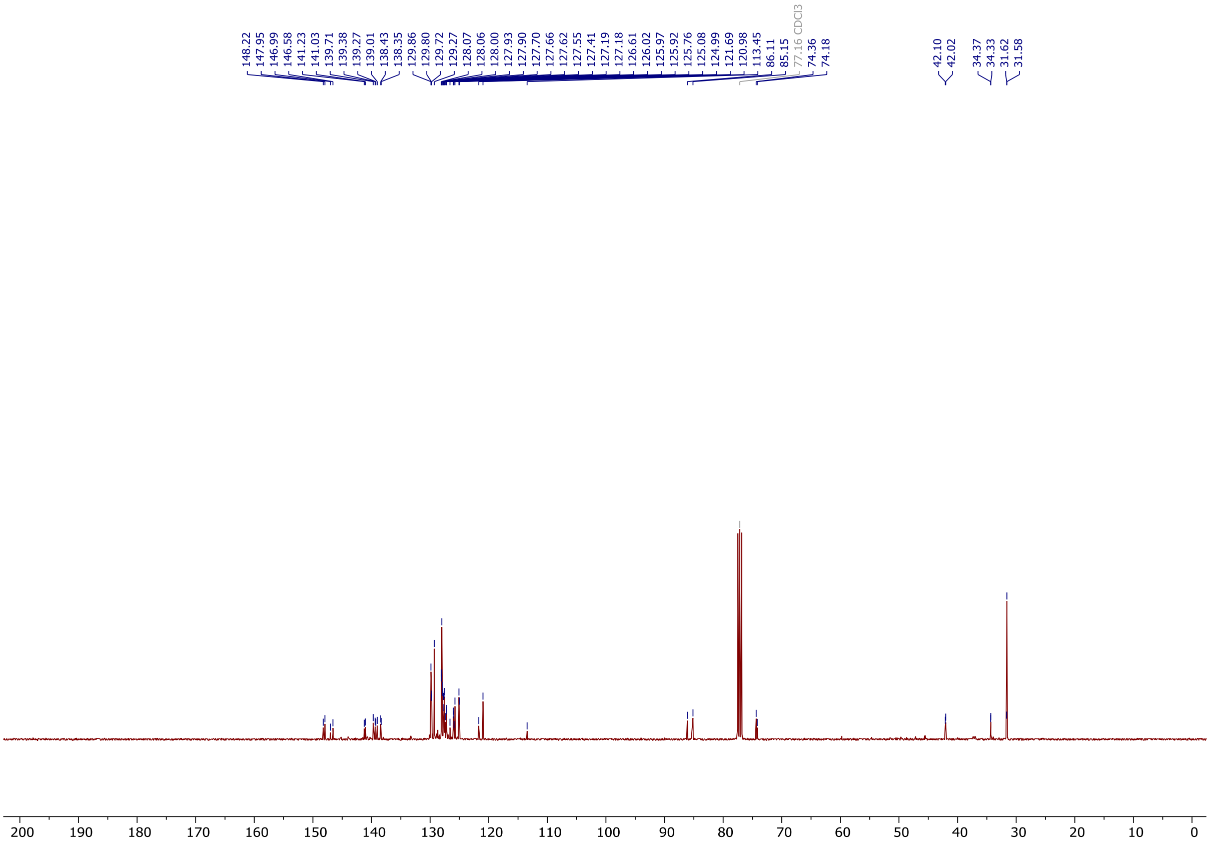

Figure S287: ^{13}C NMR Spectrum of 9a in $CDCl_3$ after isolation via column chromatography.

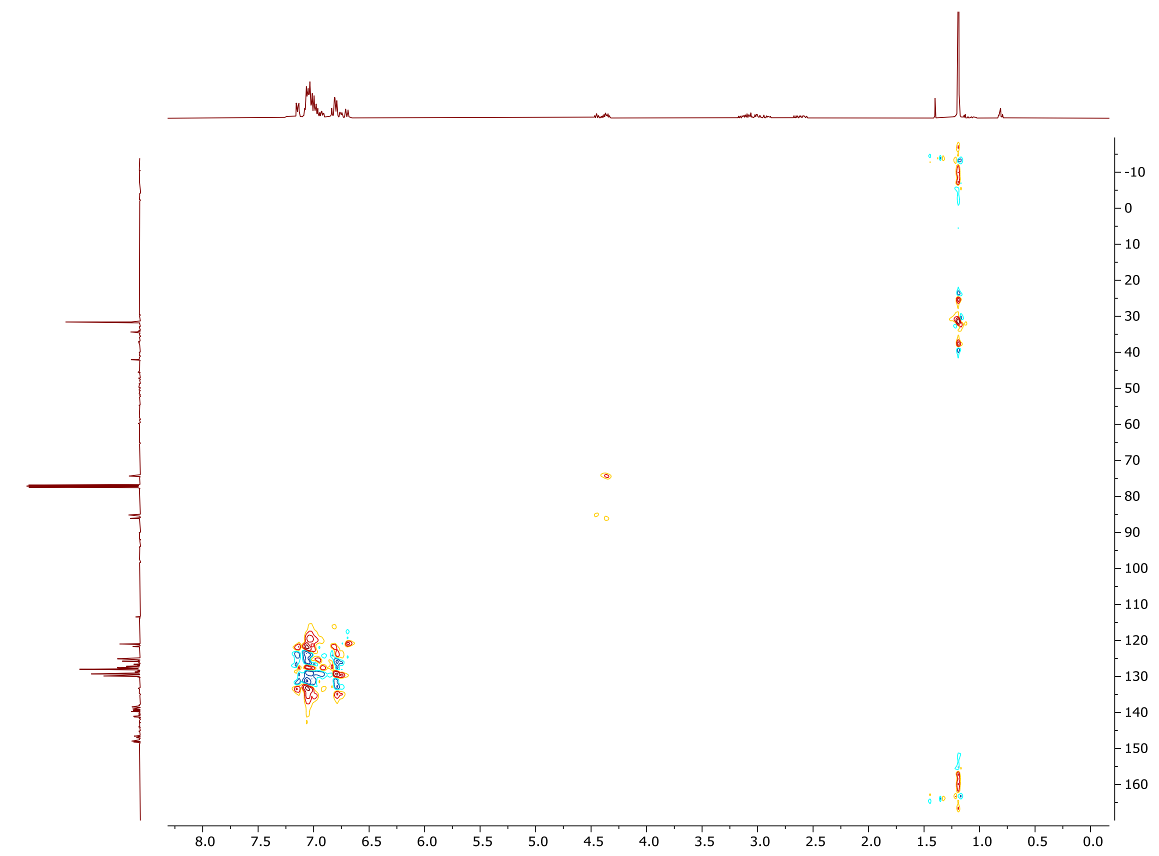

Figure S288: ¹H-¹³C HSQC NMR Spectrum of 9a in $CDCl_3$ after isolation via column chromatography.

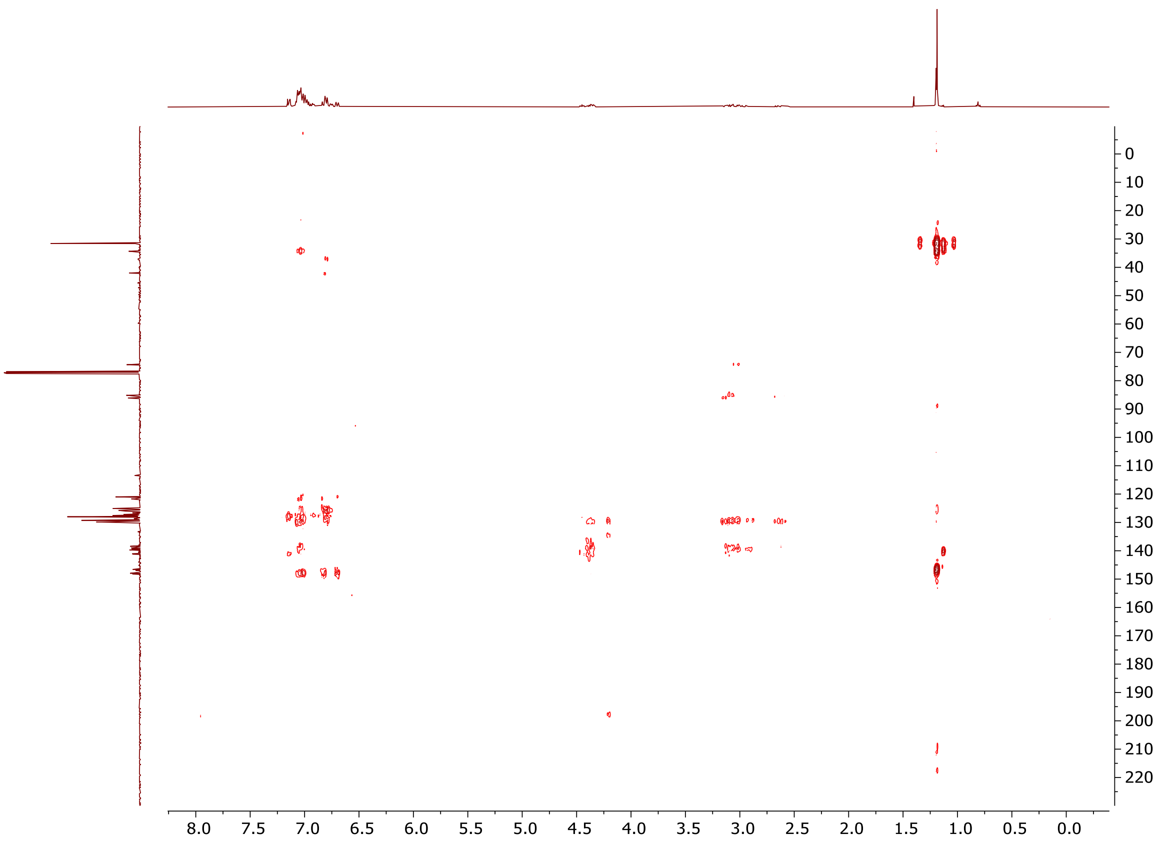

Figure S289: ¹H-¹³C HMBC NMR Spectrum of 9a in $CDCl_3$ after isolation via column chromatography.

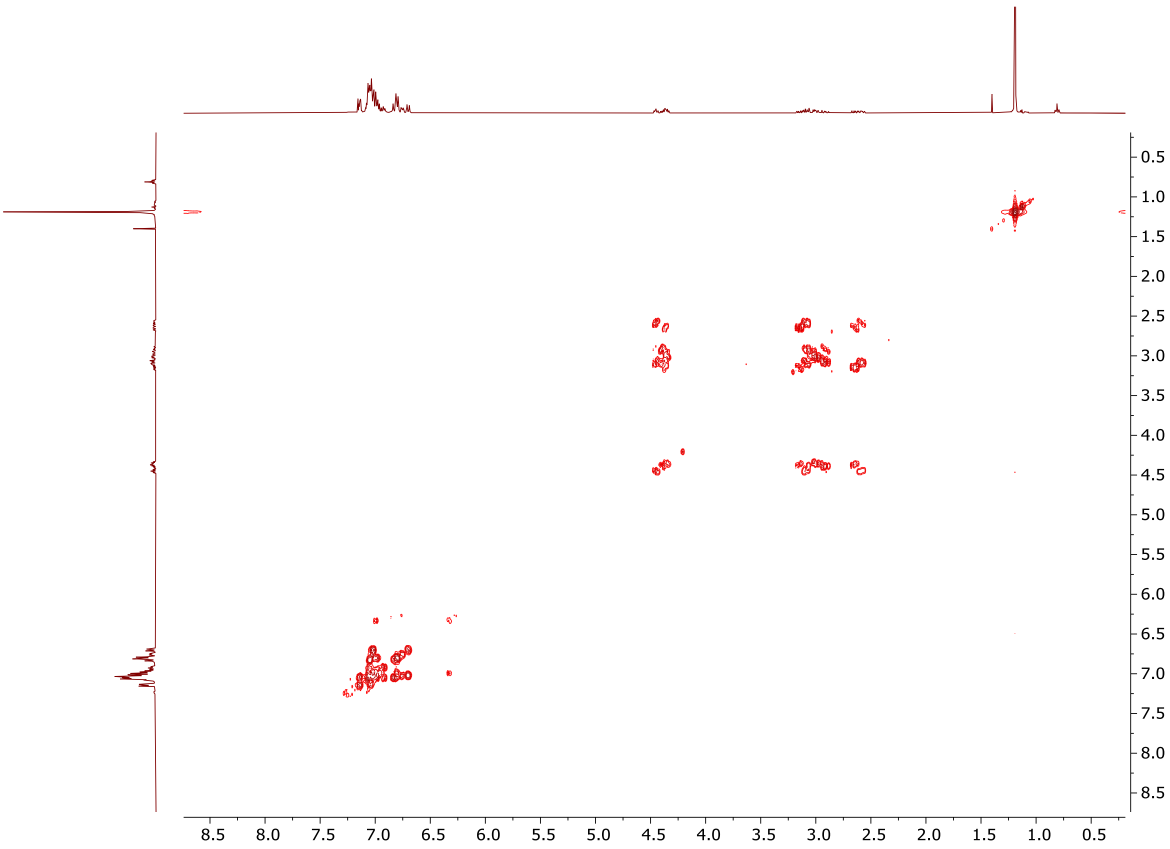

Figure S290: ^{1}H-^{1}H COSY NMR Spectrum of 9a in $CDCl_3$ after isolation via column chromatography.

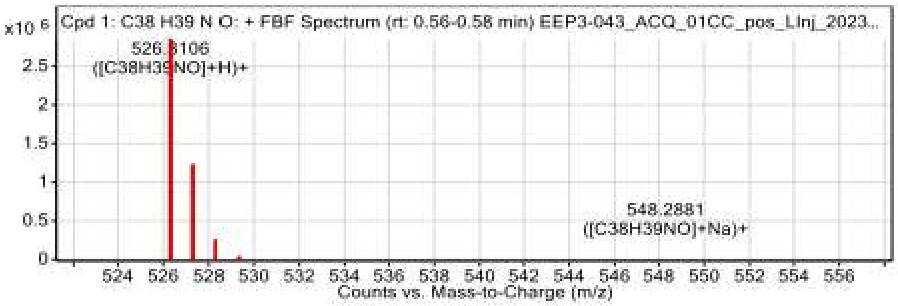

Figure S291: HRMS spectra of compound 9a.

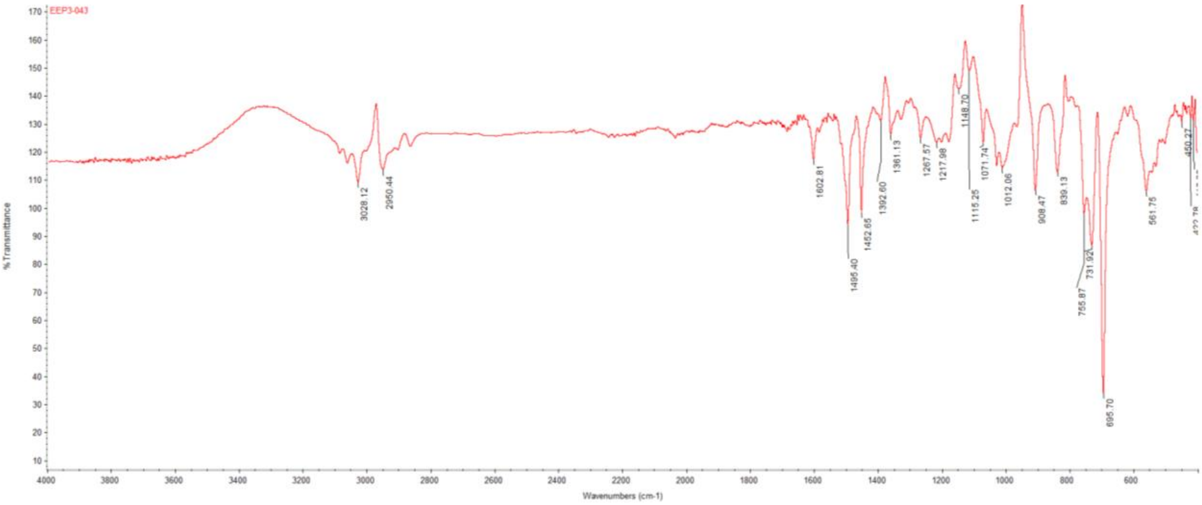

Figure S292: IR spectra for compound 9a.

11.4.26. N-(4-(tert-butyl)phenyl)-N,O-bis(2-phenylpropan-2-yl)hydroxylamine **9b**

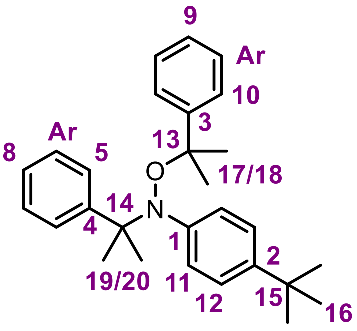

Yellow oil (332 mg, 83%)

R_f = 0.64 (30% DCM / 70% hexane)

^{1}H NMR (CDCl$_3$, 500 MHz) δ 7.52 (d, J = 7.6 Hz, 2H, C^5-H), 7.49 (d, J = 7.9 Hz, 2H, C^{10}-H), 7.30 – 7.21 (m, 6H, Ar, Ar, C^8-H, C^9-H), 7.09 (d, J = 8.1 Hz, 2H, C^{12}-H), 6.93 (d, J = 8.1 Hz, 2H, C^{11}-H), 1.62 (s, 3H, $C^{19/20}$-H), 1.48 (app. d, 6H, C^{17-20}), 1.30 (s, 9H, C^{16}-H), 1.25 (2, 3H, $C^{17/18}$-H).

^{13}C{^{1}H} NMR (CDCl$_3$, 126 MHz) δ 149.1 (C^1), 147.2 (C^2). 147.1 (C^3), 146.2 (C^4), 128.2 (C^5), 127.8 (Ar), 127.4 (Ar), 126.8 (C^8), 126.7 (C^9), 125.8 (C^{10}), 125.4 (C^{11}), 123.8 (C^{12}), 81.2 (C^{13}), 65.1 (C^{14}), 34.3 (C^{15}), 31.6 (C^{16}), 28.3 ($C^{17/18}$), 27.4 ($C^{17/28}$), 26.5 ($C^{19/20}$), 25.0 ($C^{19/20}$).

HRMS (ESI+): calcd for [M, $C_{28}H_{35}NO$]$^+$ 402.2791, found 402.2809.

IR (Neat): 2961, 2865, 1611, 1516, 816, 696 cm^{-1}.

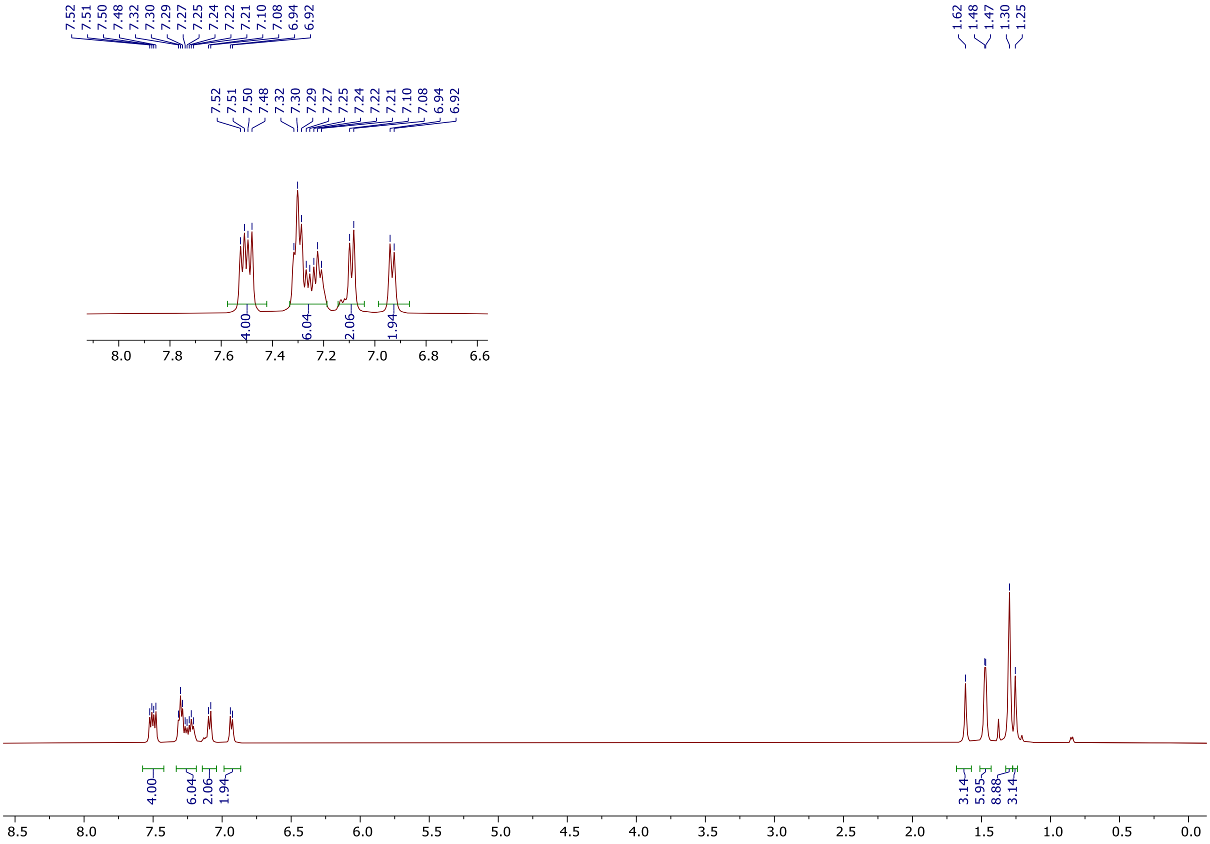

Figure S293: ^{1}H NMR Spectrum of 9b in CDCl$_3$ after isolation via column chromatography.

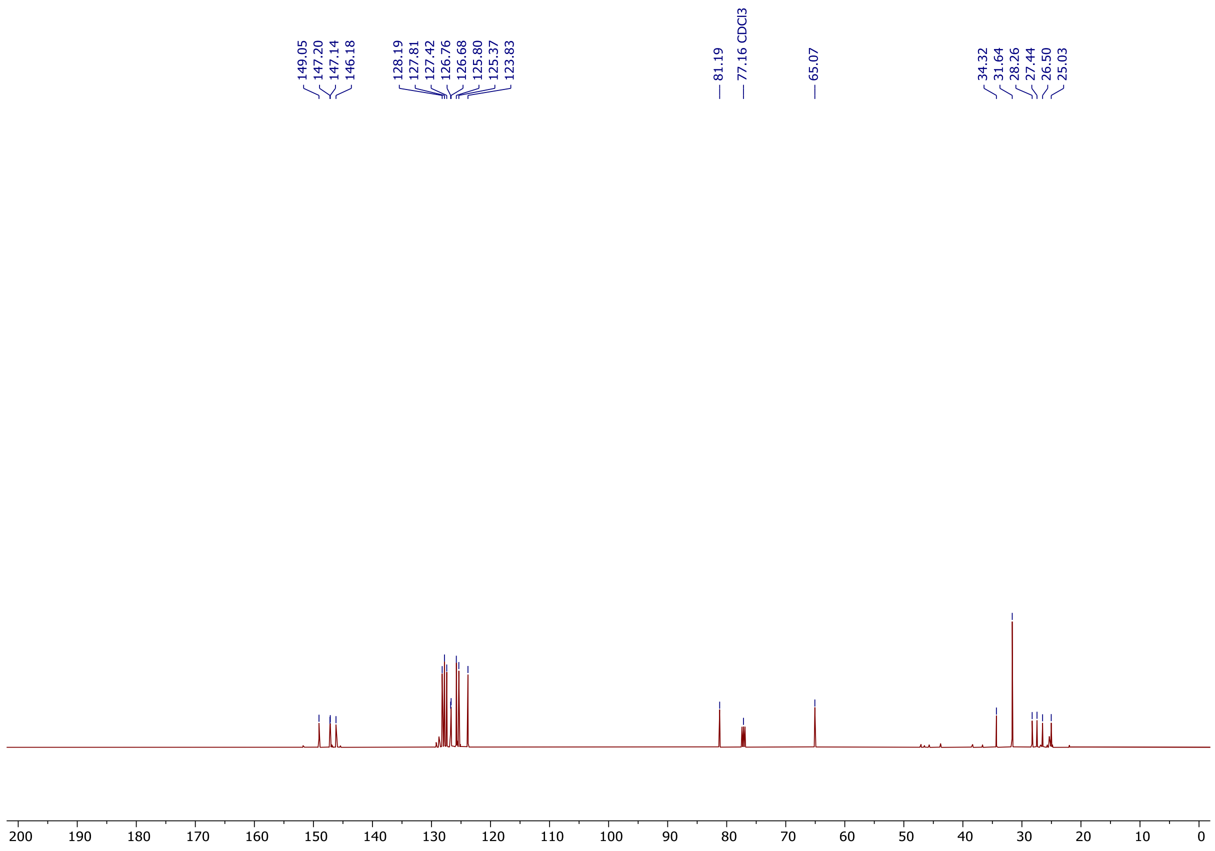

*Figure S294: [13]*C NMR Spectrum of 9b in $CDCl_3$ after isolation via column chromatography.

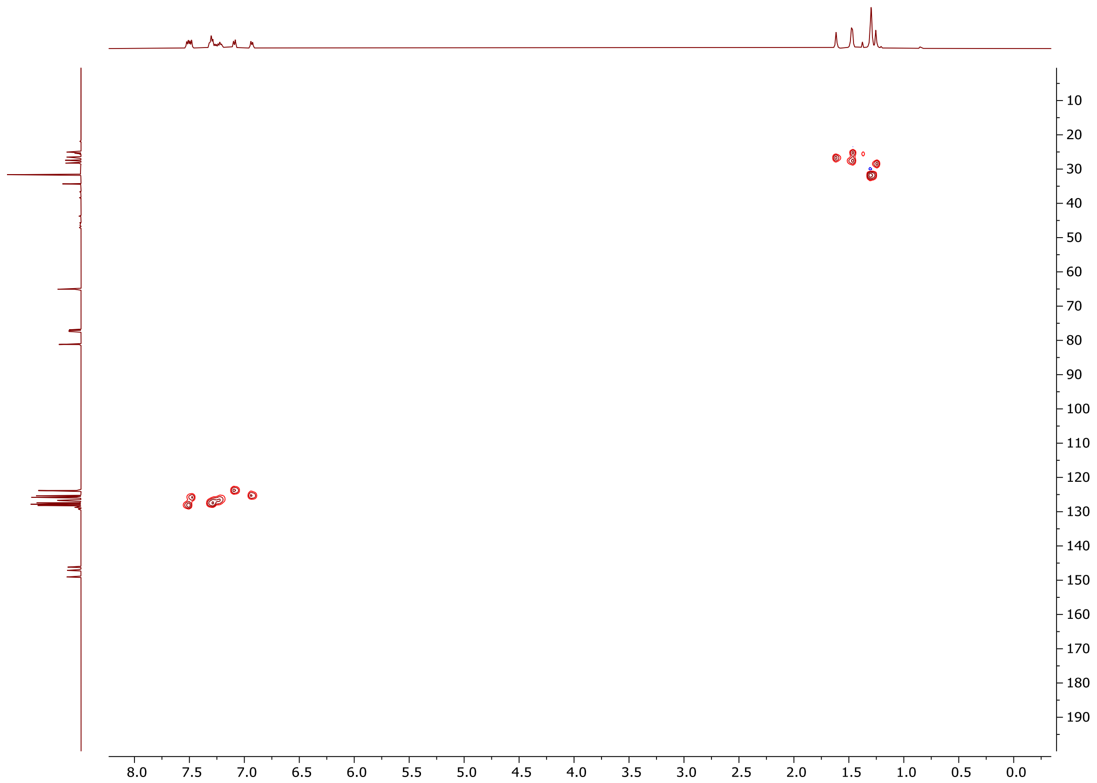

Figure S295: ^{1}H-^{13}C HSQC NMR Spectrum of 9b in $CDCl_3$ after isolation via column chromatography.

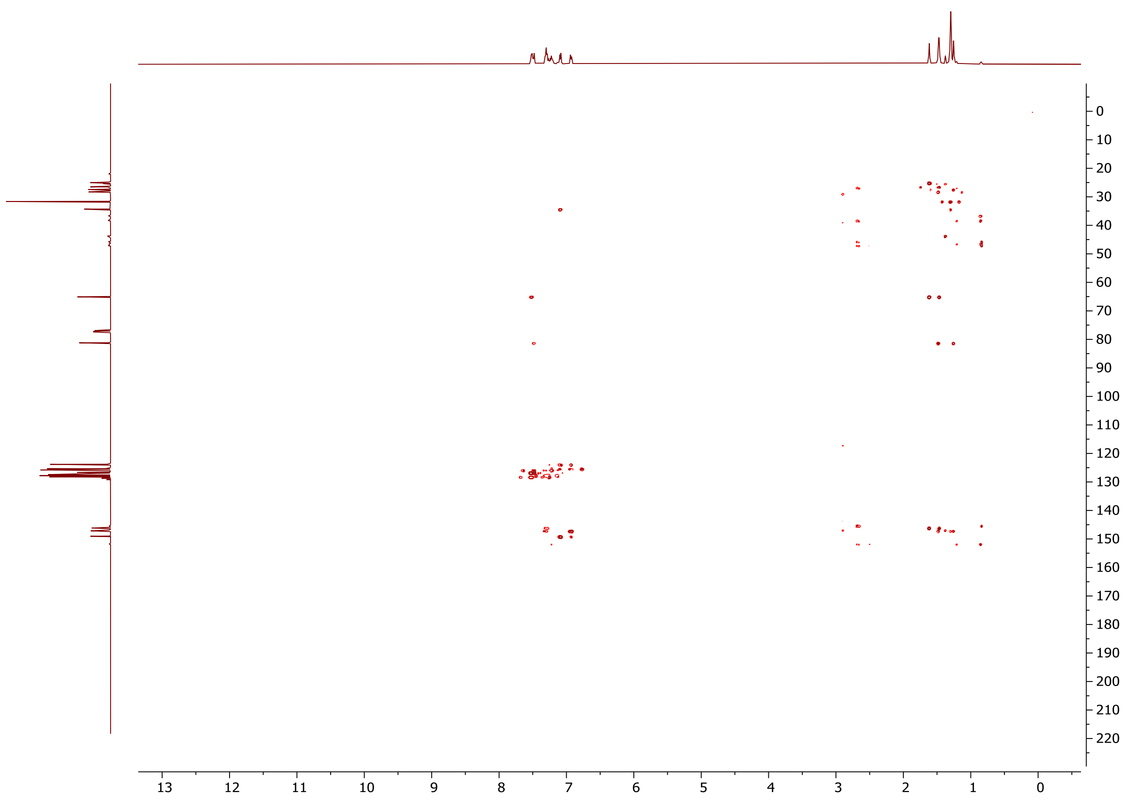

Figure S296: ^{1}H-^{13}C HMBC NMR Spectrum of 9b in $CDCl_3$ after isolation via column chromatography.

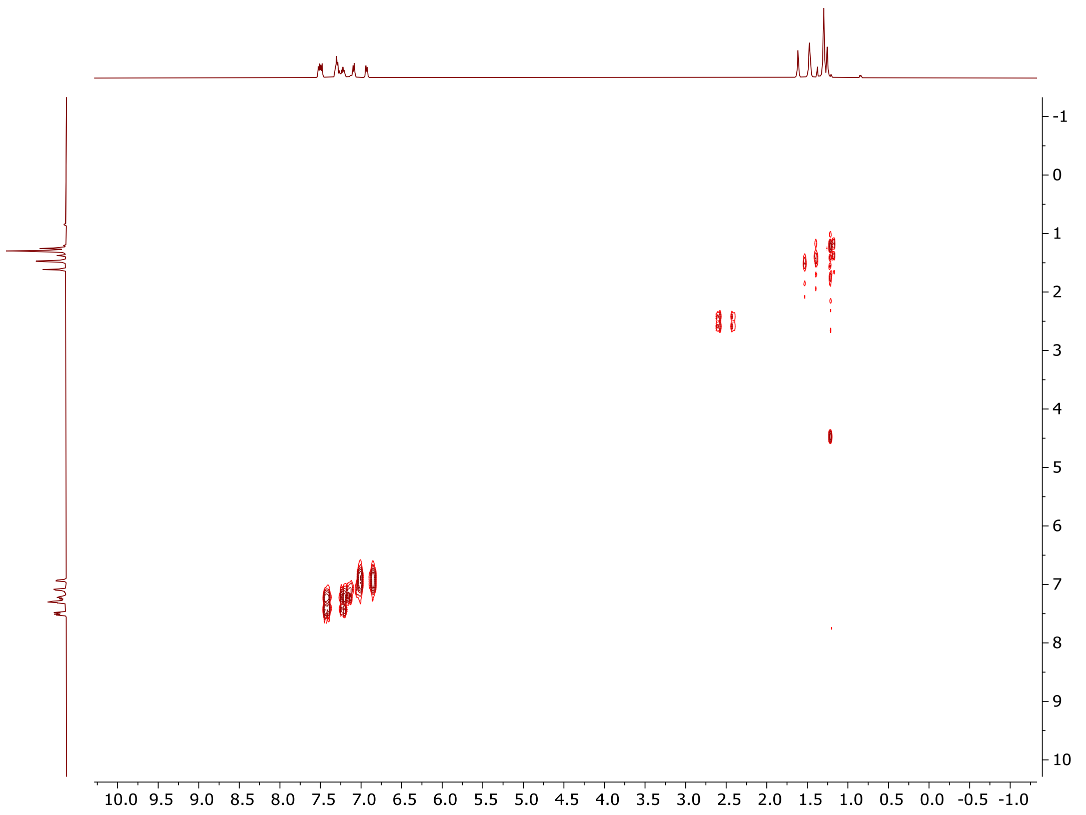

Figure S297: ^{1}H-^{1}H COSY NMR Spectrum of 9b in $CDCl_3$ after isolation via column chromatography.

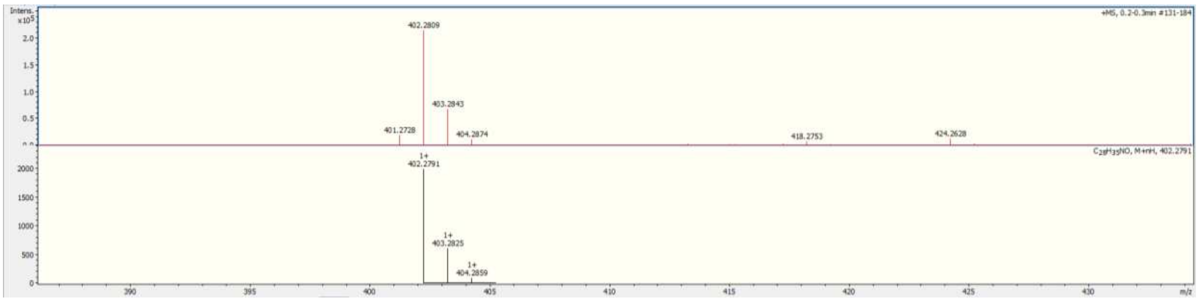

Figure S298: HRMS spectra for compound 9b.

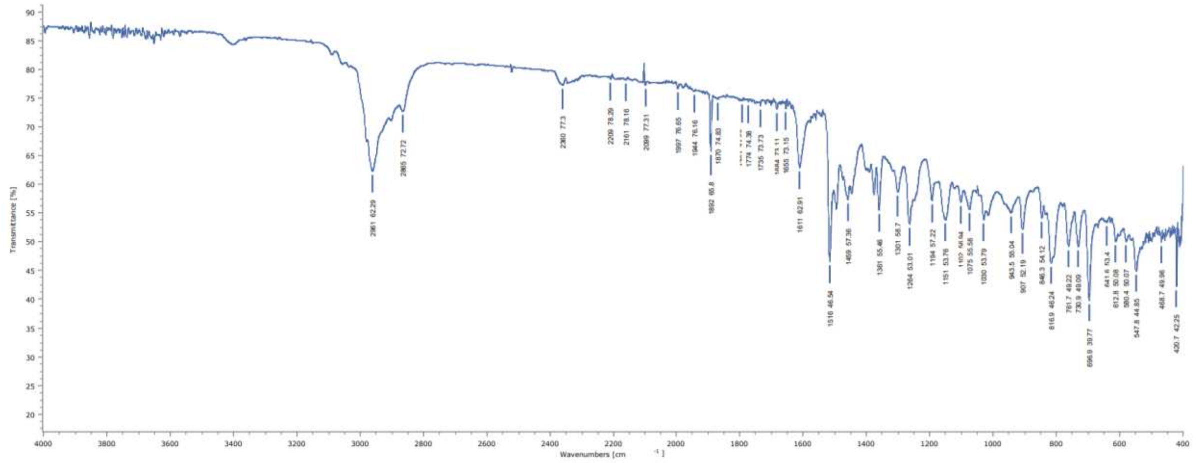

Figure S299: IR spectra for compound 9b.

11.4.27. N-(4-(tert-butyl)phenyl)-N,O-bis(1,2,3,4-tetrahydronaphthalen-1-yl)hydroxylamine **9c**

Mixture of Diastereoisomers

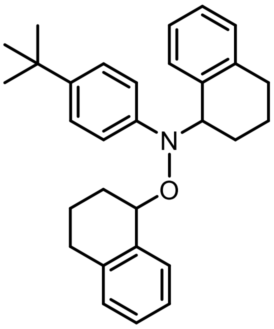

Data Collected Before Zinc Reduction

Bright yellow oil (272 mg, 64%)

^{1}H NMR (CD_3OD, 500 MHz) δ 7.89 (d, J = 7.7 Hz, 1H, Ar-H), 7.71 (d, J = 8.3 1H, Ar-H), 7.33 (d, J=8.6 Hz, 1H, Ar-H), 7.29 (d, J = 7.7 Hz, 1H, Ar-H), 7.25 – 7.02 (m, 16H, Ar-H), 6.98 (d, J = 7.9 Hz, 2H, Ar-H), 6.91 (app. t, J = 7.3 Hz, 1H, Ar-H), 4.57 (app. t, J = 6.9 Hz, 2H, OC-H), 4.49 (app. br s, 2H, NC-H), 2.88 – 2.49 (m,12H, CH_2), 2.20 – 1.46 (m, 12H, CH_2), 1.33 (s, 9H, $(CH_3)_3$), 1.30 (s, 9H, $(CH_3)_3$).

^{13}C{^{1}H} NMR (CD_3OD, 126 MHz) δ 150.1, 146.7, 140.4, 139.3, 139.1, 137.9, 137.8, 136.2, 136.0, 131.9, 131.8, 131.6, 131.1, 129.8, 129.7, 129.7, 129.5, 128.7, 128.6, 127.9, 127.8, 126.5, 126.5, 126.3, 126.2, 126.2, 126.1, 121.0, 120.5, 80.1, 77.8, 68.6, 67.1, 35.0, 34.9, 32.0, 32.0, 31.7, 31.1, 30.7, 30.3, 30.2, 28.7, 28.4, 19.5

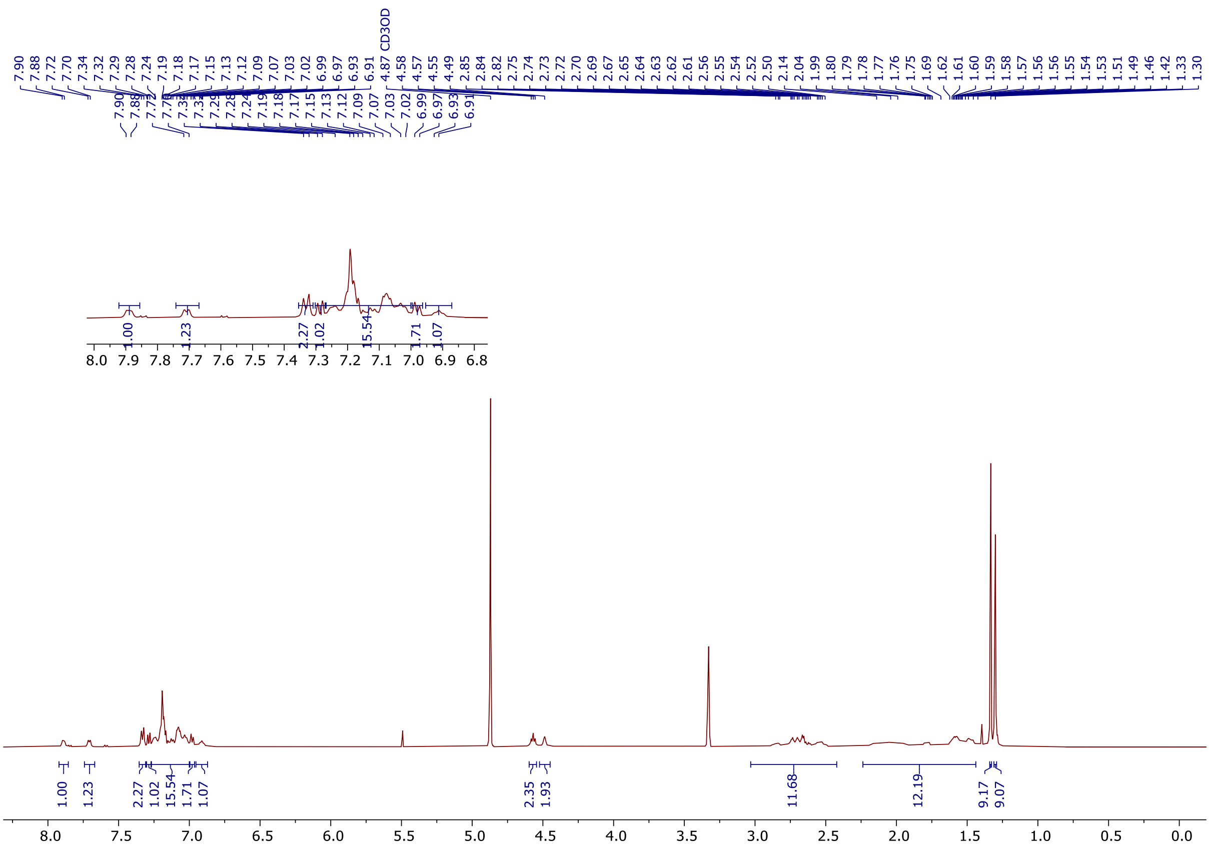

Figure S300: ^{1}H NMR Spectrum of 9c in CD_3OD after isolation via column chromatography.

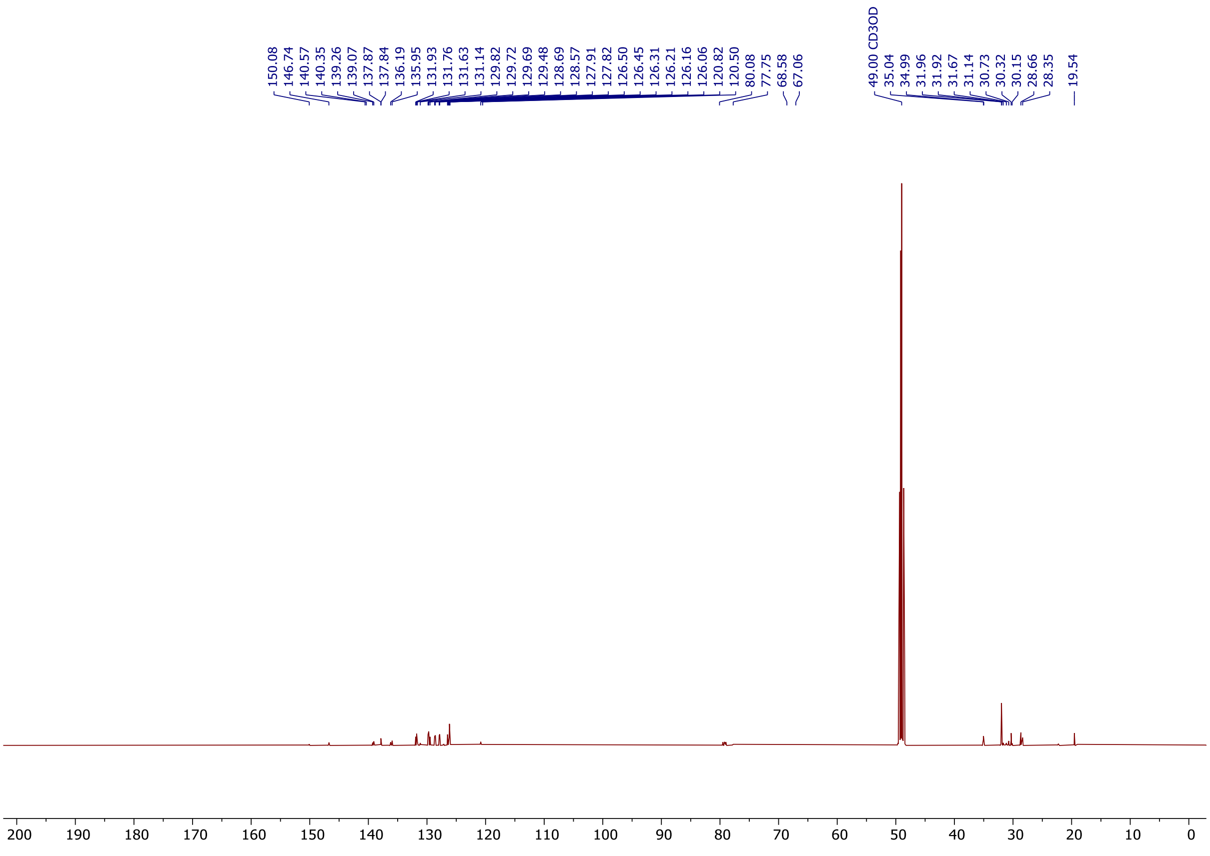

Figure S301: ^{13}C NMR Spectrum of 9c in CD_3OD after isolation via column chromatography.

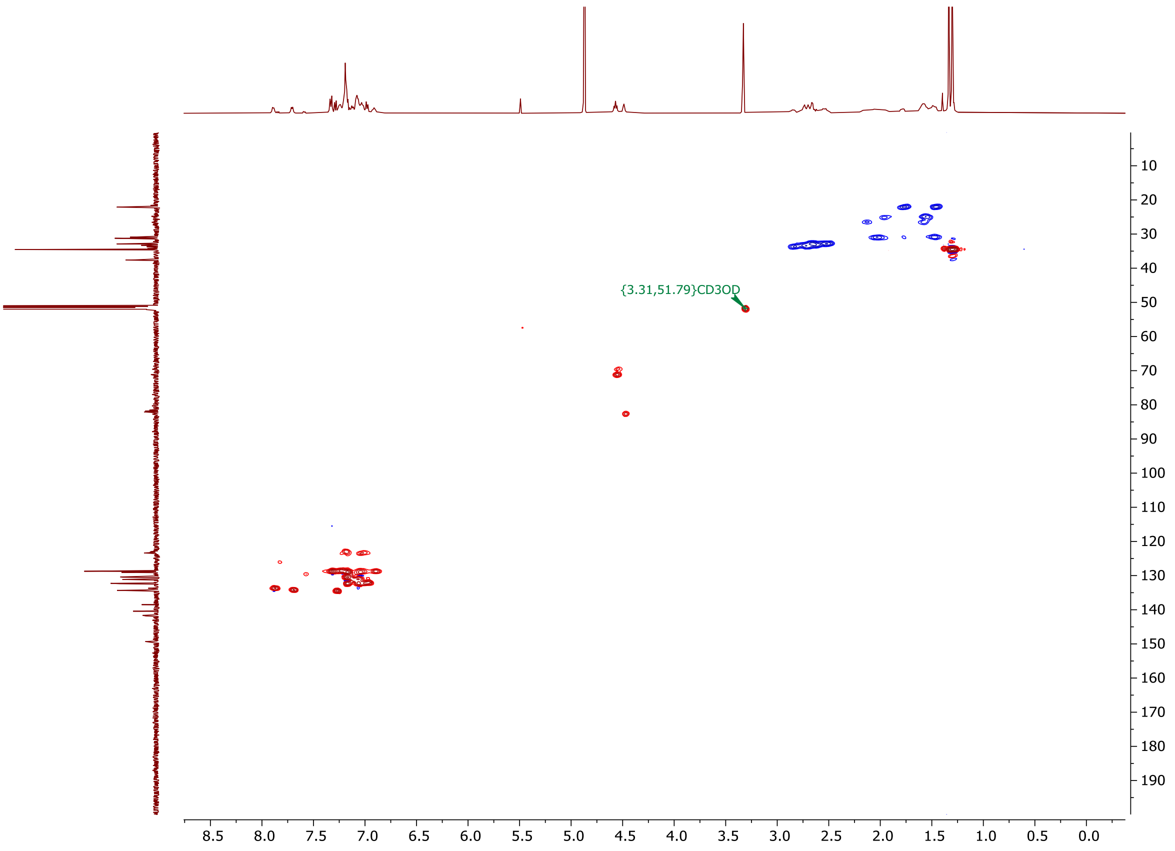

Figure S302: ¹H-¹³C HSQC NMR Spectrum of 9c in CD_3OD after isolation via column chromatography.

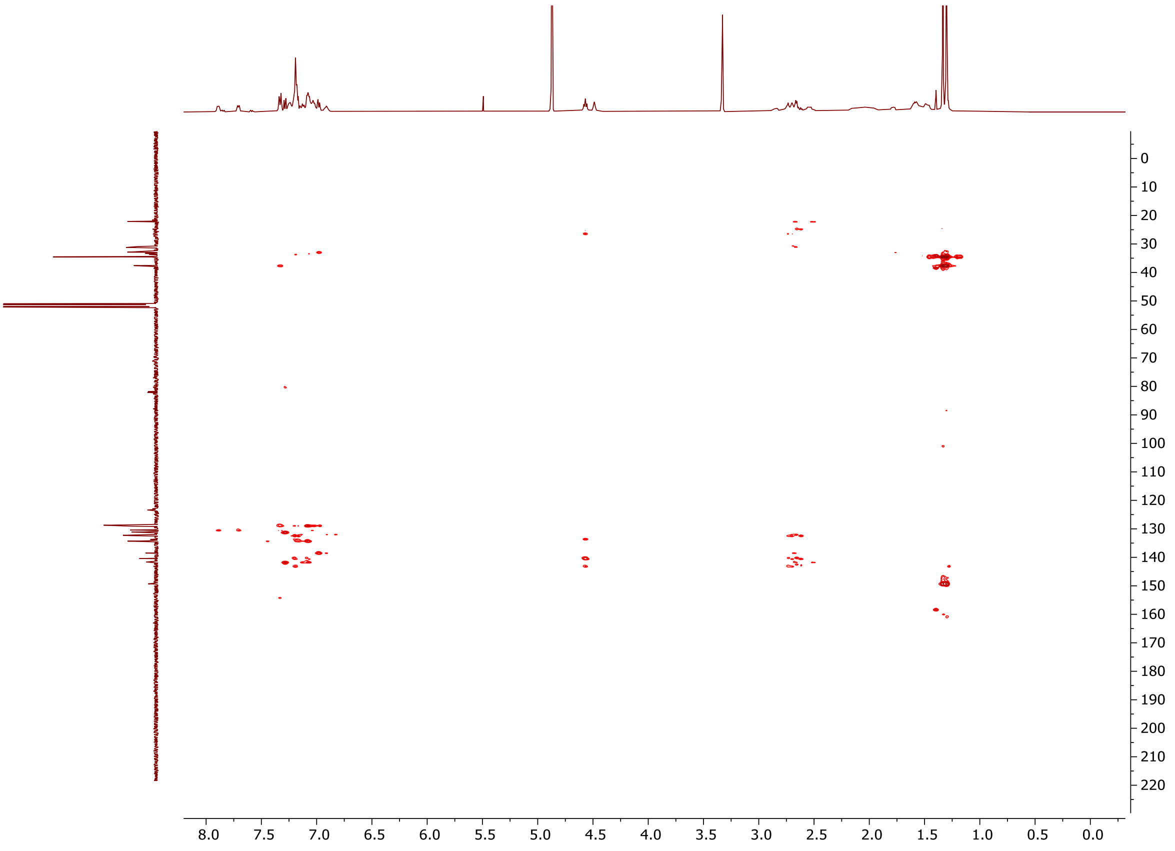

Figure S303: ¹H-¹³C HMBC NMR Spectrum of 9c in CD_3OD after isolation via column chromatography.

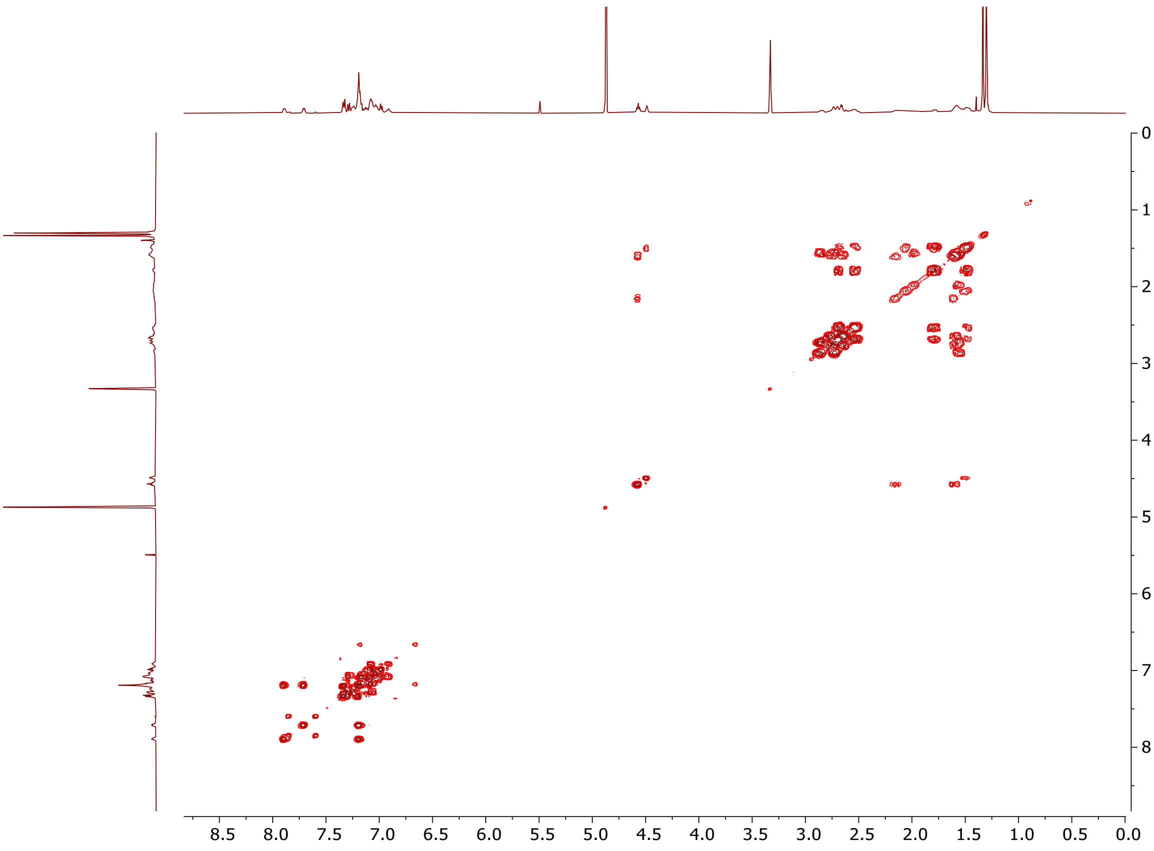

Figure S304: ^{1}H-^{13}C COSY NMR Spectrum of 9c in CD_3OD after isolation via column chromatography

11.4.28. N-(4-(tert-butyl)phenyl)-N,O-bis(4,7-dimethyl-2,3-dihydro-1H-inden-1-yl)hydroxylamine **9e**

Mixture of Diastereisomers

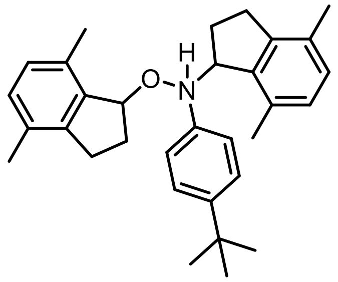

Data Collected Before Zinc Reduction

yellow oil (328 mg, 73%)

R_f = 0.69 (30% DCM / 70% hexane)

^{1}H NMR ($CDCl_3$, 500 MHz) δ 7.57 (app. d, *J* = 8.4 Hz, 2H, Ar-H), 7.49 (app. t, *J* = 8.4 Hz, 4H, Ar-H), 7.30 (app. d, *J* = 8.4 Hz, 1H, Ar-H), 7.26 – 7.12 (m, 6H, Ar-H), 7.06 (app. d, *J* = 7.5 Hz, 1H, Ar-H), 7.01 (app. d, *J* = 7.6 Hz, 1H, Ar-H), 6.65 (d, *J* = 8.4 Hz, 1H, Ar-H), 5.38 (app. d, *J* = 9.2 Hz, 1H, OC-H), 5.01 (dd, *J* = 14.3, 6.7 Hz, 2H, OC-H / NC-H), 4.75 (app. d, *J* = 5.5 Hz, 1H, HC-H), 3.14 (app. dt, *J* = 16.6, 8.5 Hz, 1H, CH_2), 2.93 (dd, *J* = 16.4, 9.1 Hz, 2H, CH_2), 2.82 (s, 3H, CH_3), 2.78 (s, 3H, CH_3), 2.74 – 2.66 (m, 2H, CH_2), 2.58 (s, 3H, CH_3), 2.53 – 2.21 (m, 6H, CH_2), 2.41 (s, 3H, CH_3), 2.37 (s, 3H, CH_3), 2.33 (s, 3H, CH_3), 2.28

(s, 3H, CH_3), 2.04 (app. p, *J* = 9.1 Hz, 1H, CH_2), 1.86 (dd, *J* = 13.2, 6.3 Hz, 2H, CH_2), 1.82 (s, 3H, CH_3), 1.76 – 1.61 (m, 2H, CH_2), 1.53 (s, 9H, $(CH_3)_3$), 1.50 (s, 9H, $(CH_3)_3$).

$^{13}C\{^1H\}$ NMR ($CDCl_3$, 126 MHz) δ 150.79, 149.84, 145.85, 145.51, 145.44, 145.30, 145.06, 144.17, 140.42, 139.88, 139.64, 139.10, 133.20, 133.10, 132.94, 132.39, 131.67, 131.12, 131.09, 130.92, 129.75, 129.31, 128.96, 128.90, 127.85, 127.51, 127.47, 127.29, 125.60, 125.43, 118.82, 117.25, 85.61, 82.46, 73.74, 72.79, 34.31, 34.18, 31.58, 31.47, 30.90, 30.31, 29.61, 29.53, 27.93, 24.83, 23.87, 19.34, 19.01, 18.91, 18.83, 18.79, 18.57, 16.99.

HRMS (ESI+): calcd for [M, $C_{32}H_{39}NO$]$^+$ 454.3110, found 454.1309.

IR (Neat): 2921, 2886, 1607, 1498, 1458, 906 cm^{-1}.

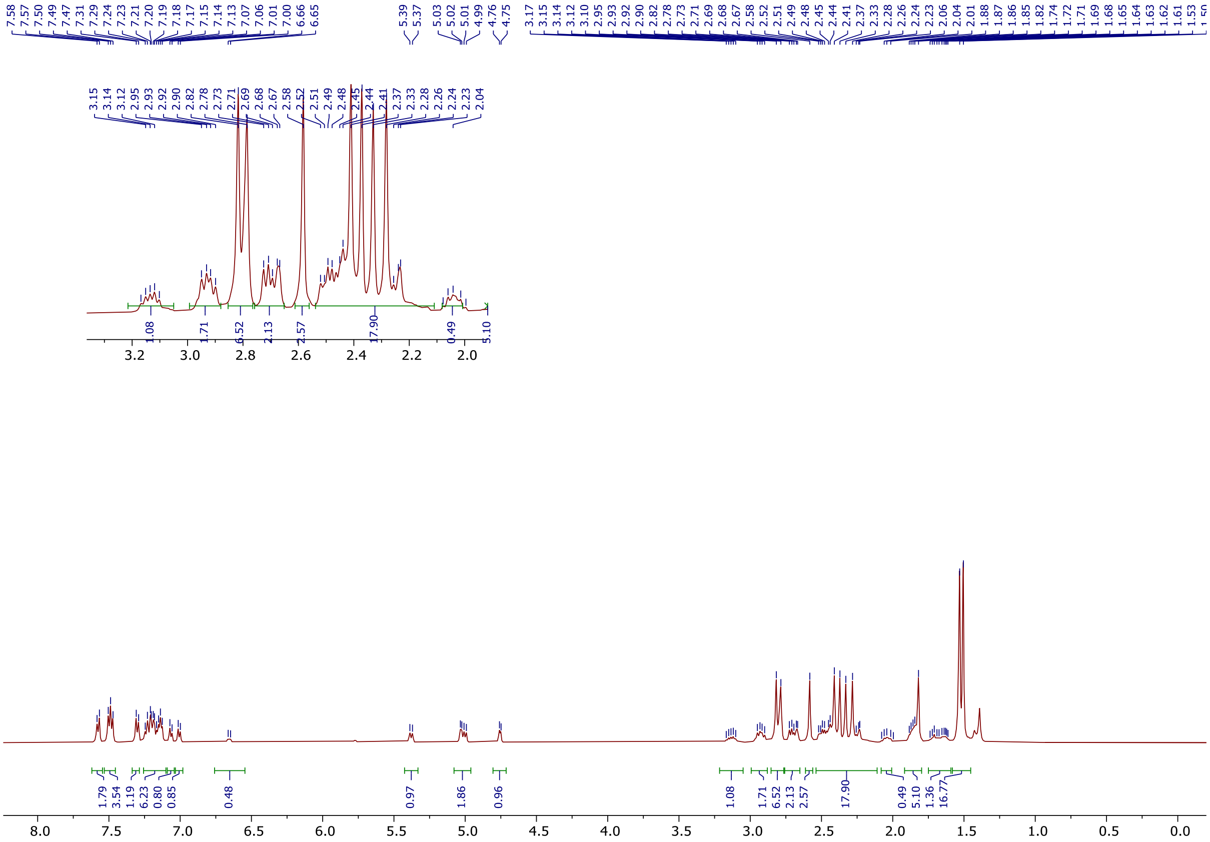

Figure S305: ^{1}H NMR Spectrum of 9e in $CDCl_3$ after isolation via column chromatography.

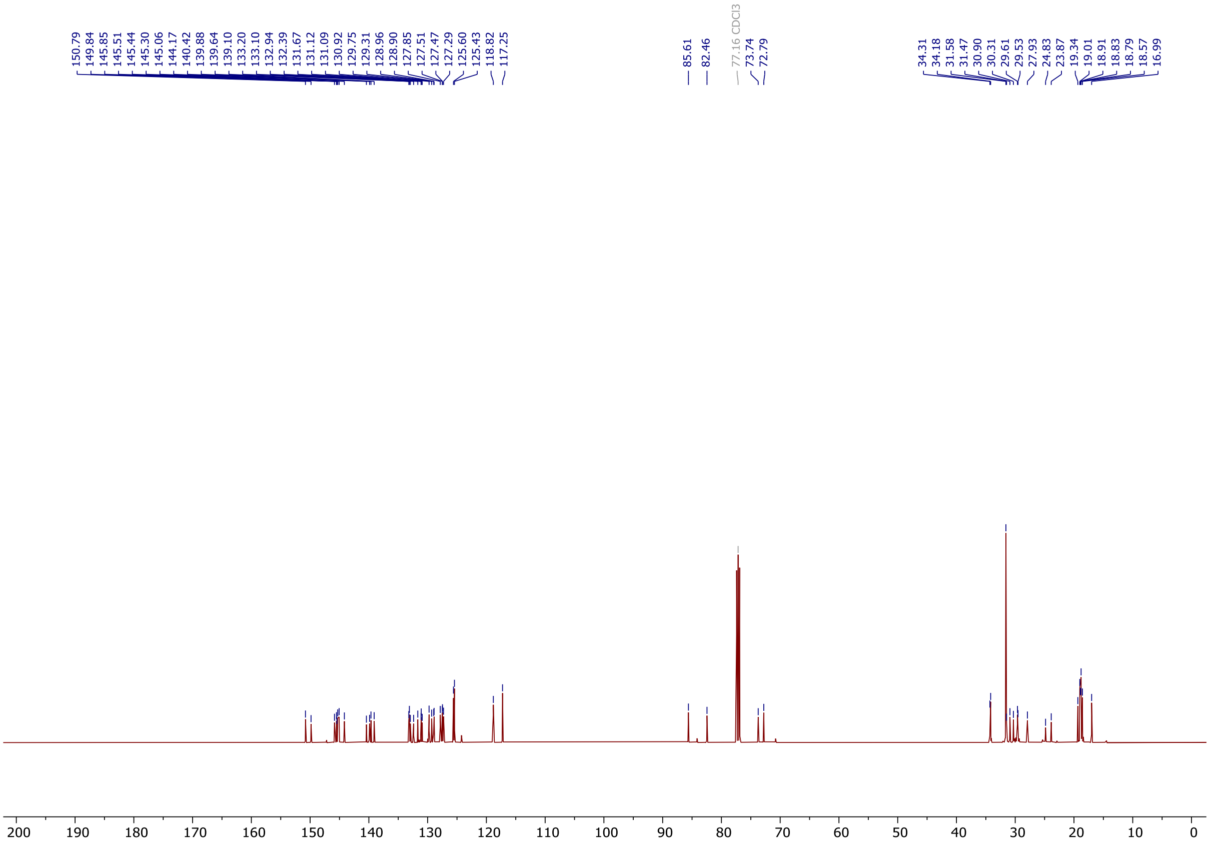

Figure S306: *[13]*C NMR Spectrum of 9e in $CDCl_3$ after isolation via column chromatography.

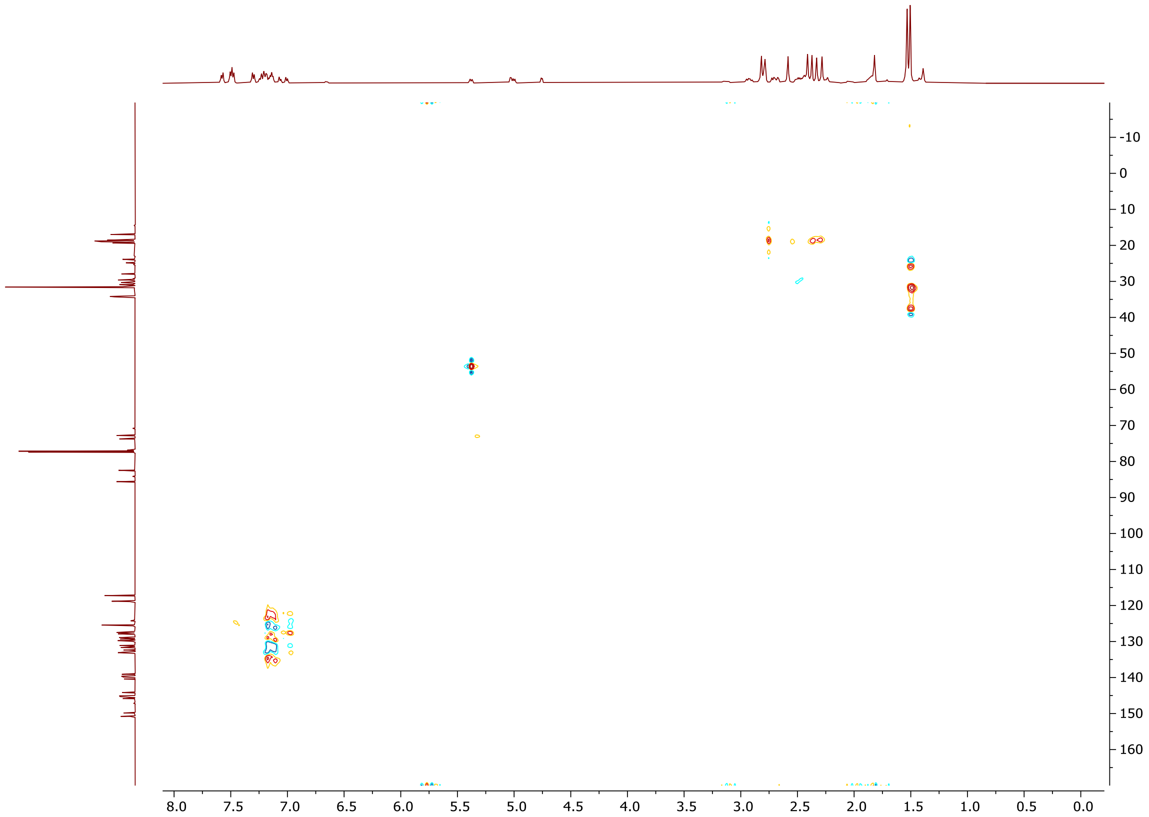

Figure S307: *^{1}H-^{13}C HSQC* NMR Spectrum of 9e in $CDCl_3$ after isolation via column chromatography.

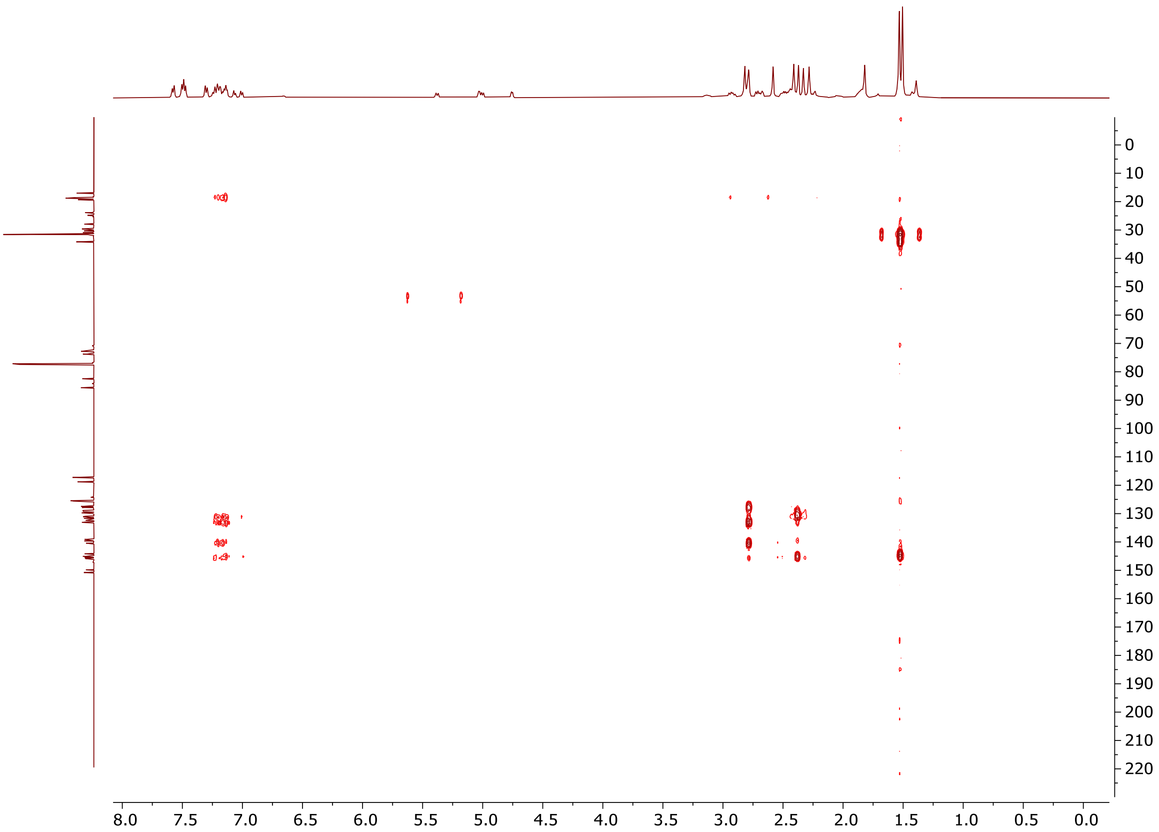

Figure S308: ^{1}H-^{13}C HMBC NMR Spectrum of 9e in $CDCl_3$ after isolation via column chromatography.

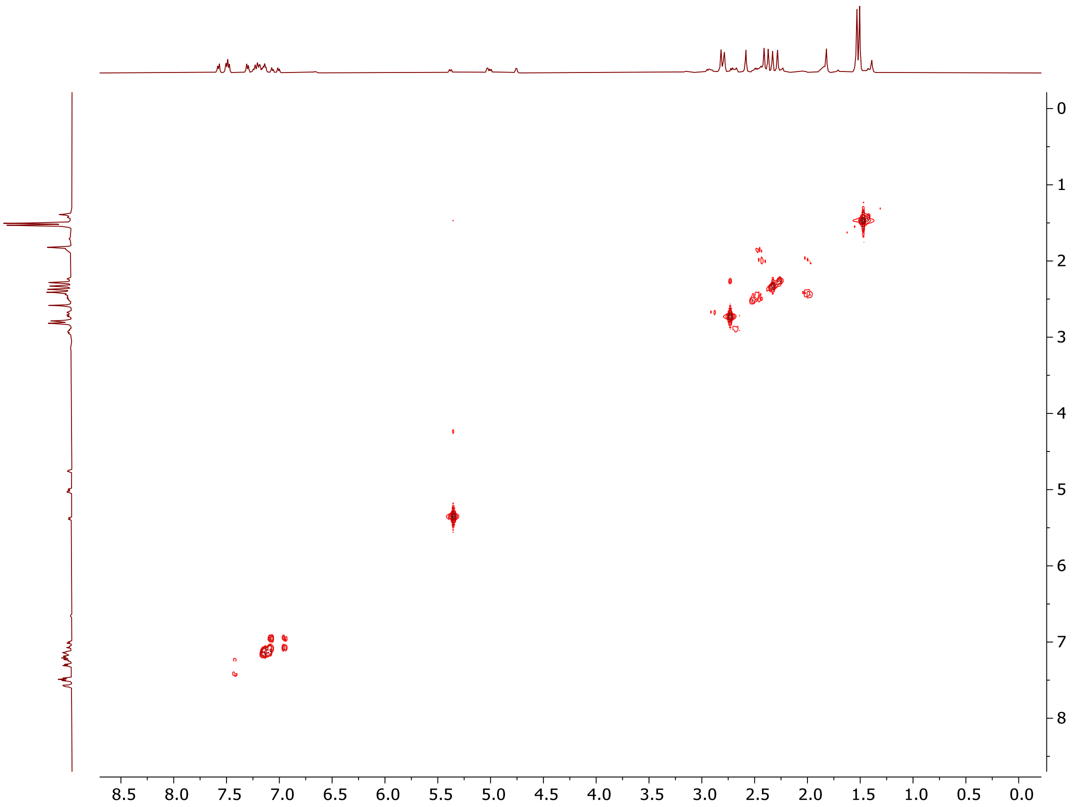

Figure S309: ^{1}H-^{1}H COSY NMR Spectrum of 9e in $CDCl_3$ after isolation via column chromatography.

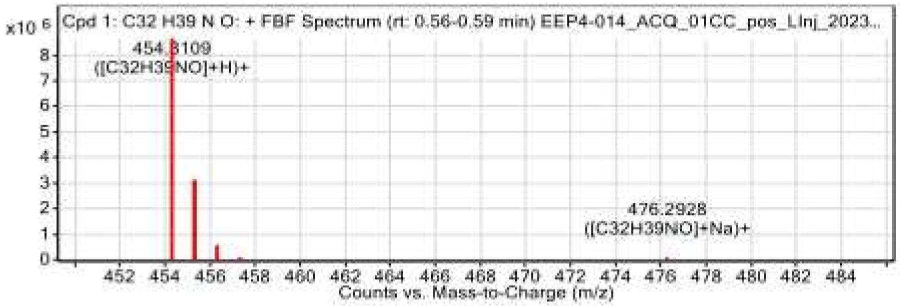

Figure S310: HRMS of compound 9e.

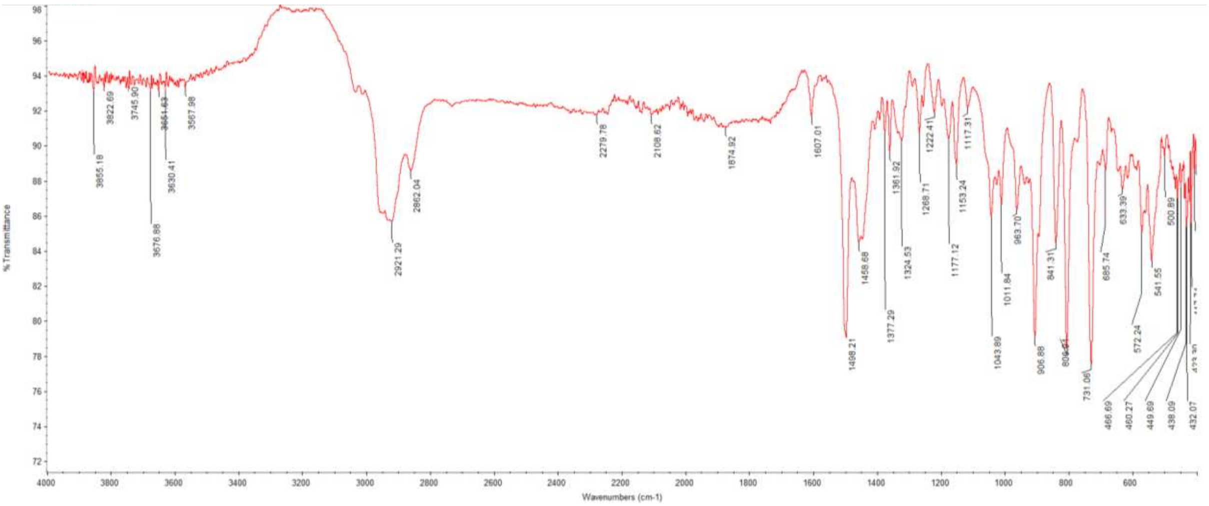

Figure S311: IR spectra for compound 9e.

11.4.29. N-phenyl-N,O-bis(2-phenylpropan-2-yl)hydroxylamine **9n**

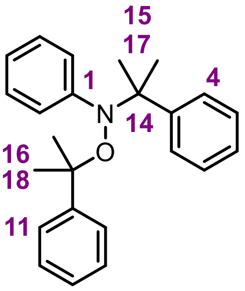

Bright yellow oil (307.8 mg, 89%)

R_f = 0.71 (30% DCM / 70% hexane)

^{1}H NMR ($CDCl_3$, 500 MHz) δ 7.46 – 7.43 (m, 2H, C^{11}-H), 7.42 – 7.39 (m, 2H, C^4-H), 7.29 – 7.17 (m, 6H, Ar-H), 7.00 (ap t, J = 7.6 Hz, 2H, Ar-H), 6.96 – 6.91 (m, 3H, Ar-H), 1.54 (s, 3H, $C^{15/17}$-H), 1.46 (s, 3H, $C^{16/18}$-H), 1.41 (s, 3H, $C^{16/18}$-H), 1.18 (s, 3H, $C^{15/17}$-H).

^{13}C{^{1}H} NMR ($CDCl_3$, 126 MHz) δ 152.0 (C^1), 147.0 (C^2), 145.8 (C^3), 128.1 (C^4), 127.9 (C^5), 127.5 (C^6), 127.1 (C^7), 126.9 (C^8), 126.8 (C^9), 125.9 (C^{10}), 125.8 (C^{11})124.5 (C^{12}), 81.6 (C^{13}), 65.2 (C^{14}), 28.4 (C^{15}), 27.4 (C^{16}), 27.2 (C^{17}), 24.3 (C^{18}).

HRMS (ESI+): calcd for [M, $C_{24}H_{27}NO$]$^+$ 346.2165, found 346.2168.

IR (Neat): 3059, 2981, 1594, 1484, 1148, 762, 694 cm^{-1}.

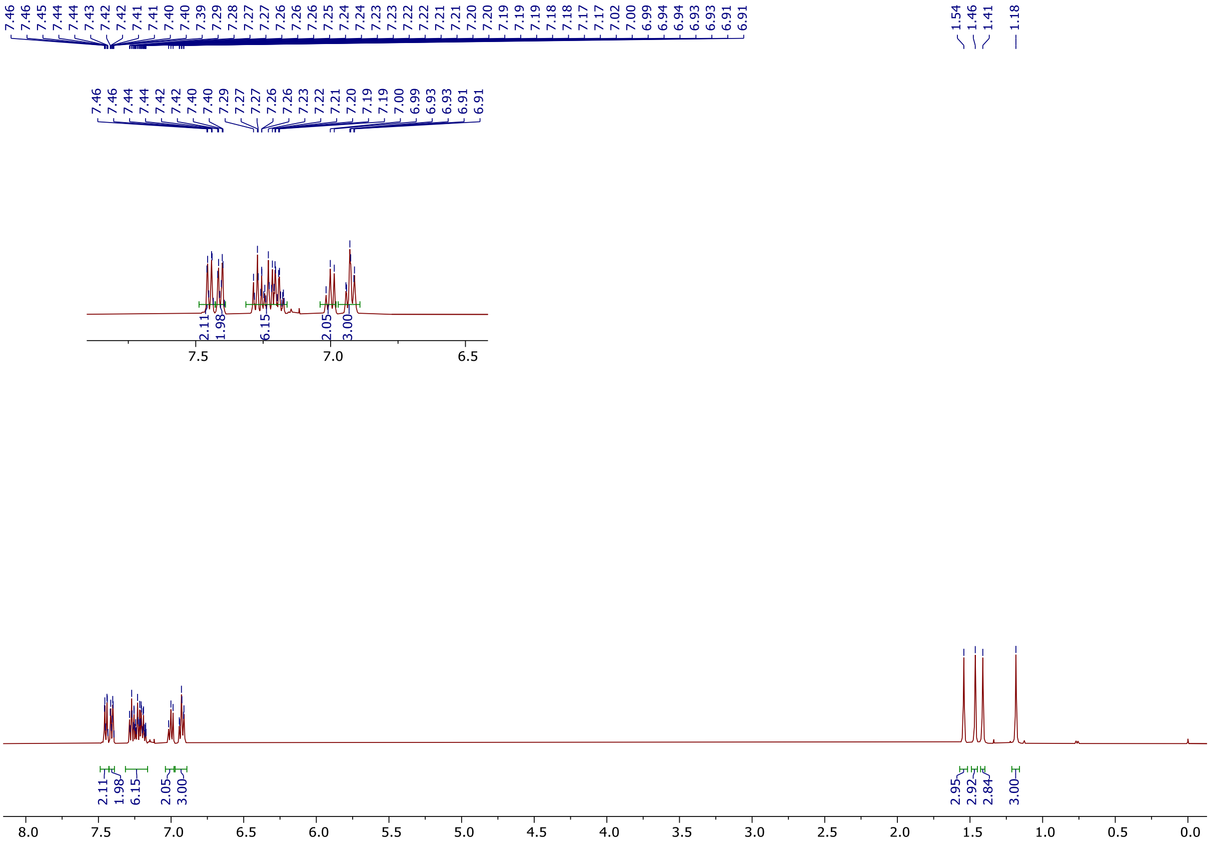

Figure S312: ^{1}H NMR Spectrum of 9n in $CDCl_3$ after isolation via column chromatography.

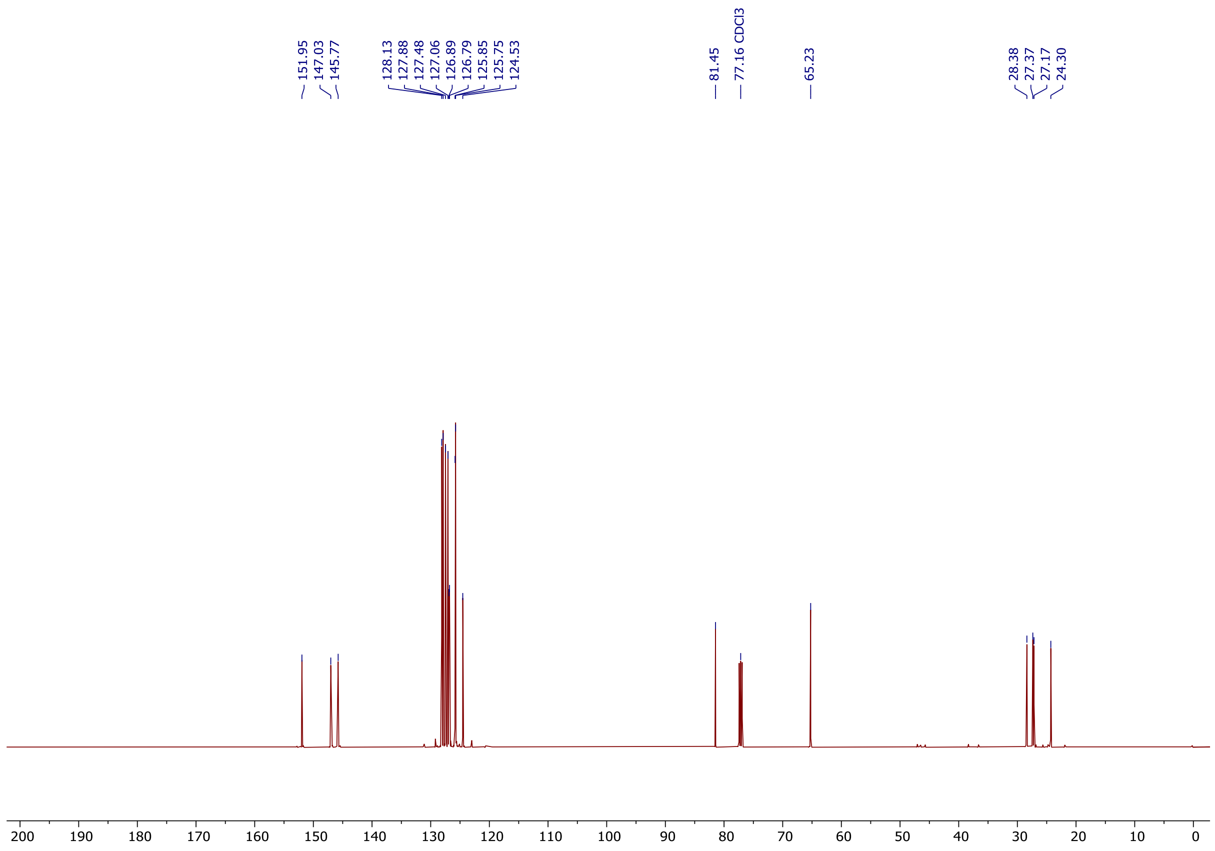

Figure S313: ^{13}C NMR Spectrum of 9n in $CDCl_3$ after isolation via column chromatography.

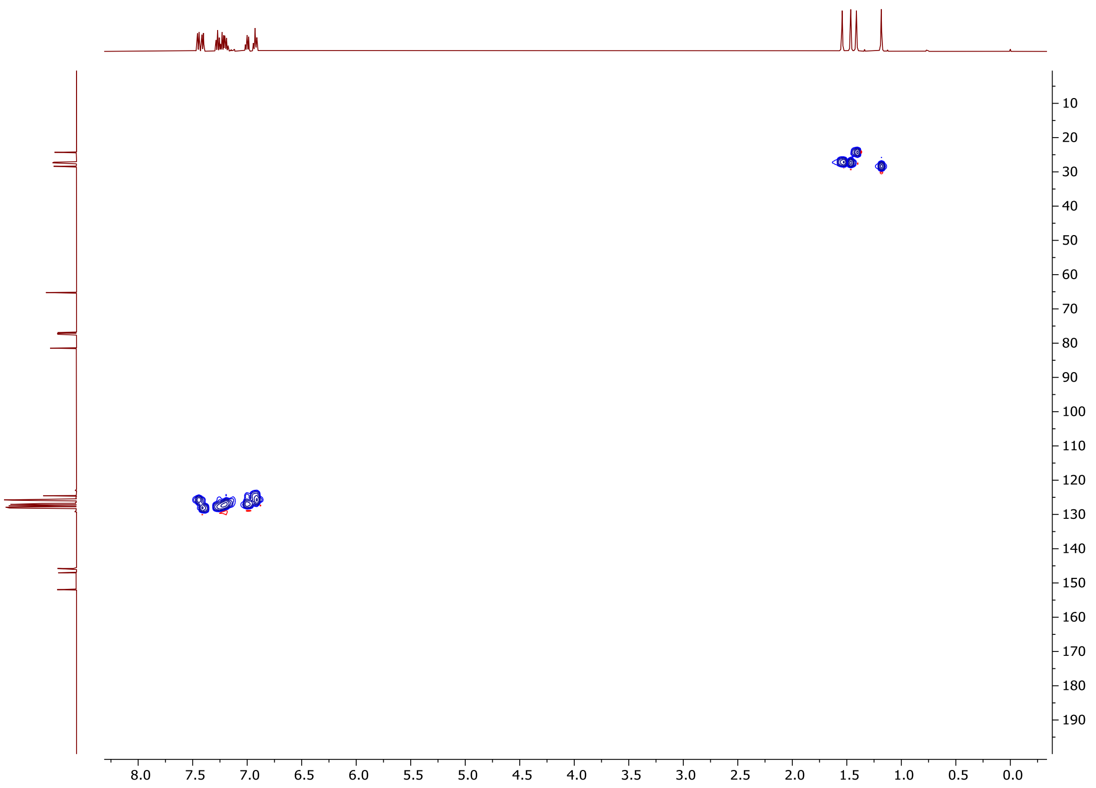

Figure S314: ^{1}*H-*^{13}*C HSQC* NMR Spectrum of 9n in $CDCl_3$ after isolation via column chromatography.

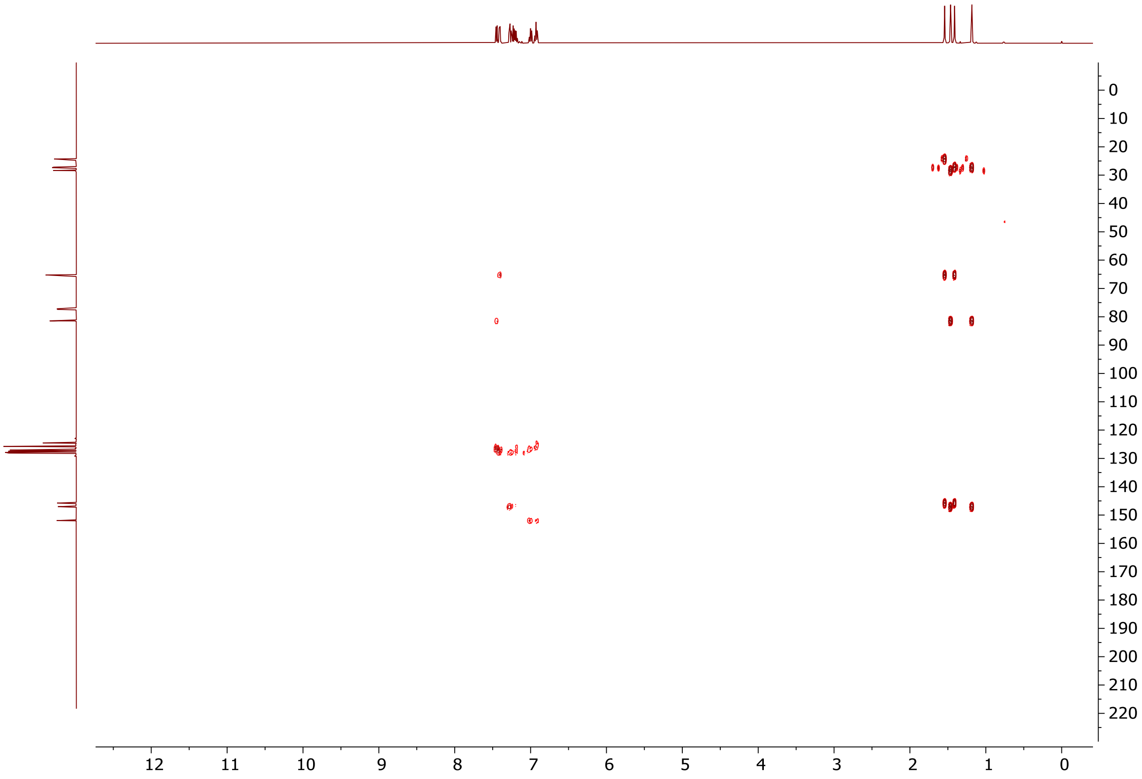

Figure S315: ¹H-¹³C HMBC NMR Spectrum of 9n in $CDCl_3$ after isolation via column chromatography.

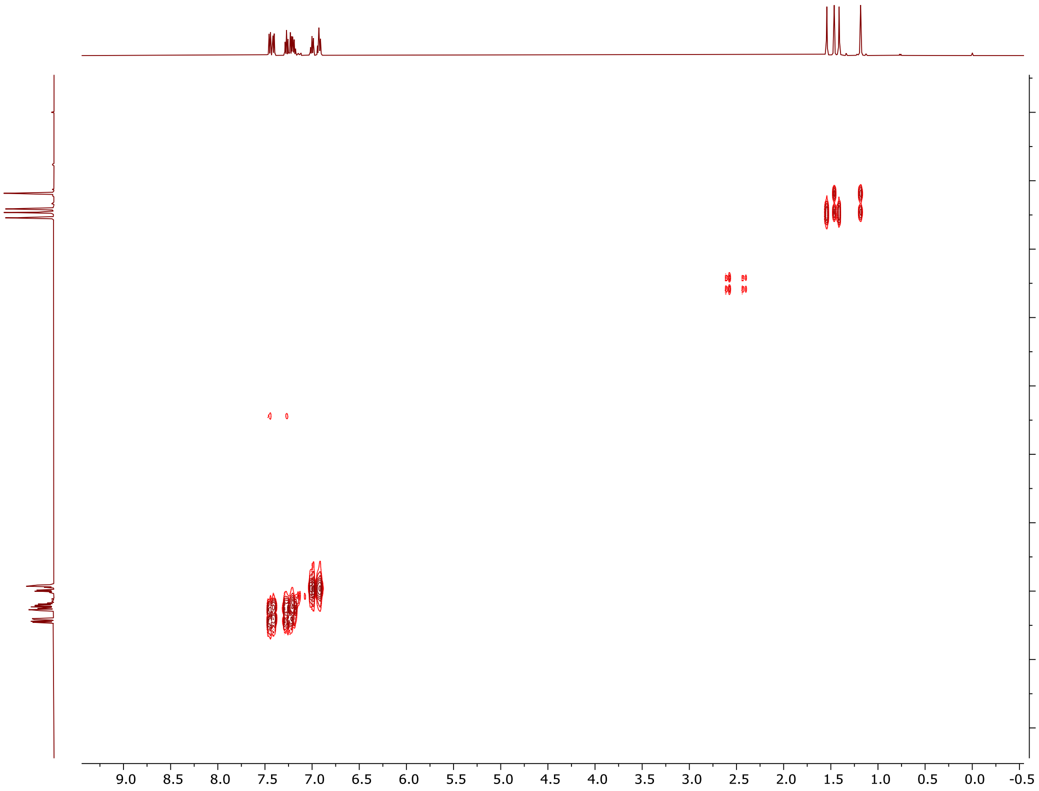

Figure S316: ¹H-¹H COSY NMR Spectrum of 9n in $CDCl_3$ after isolation via column chromatography.

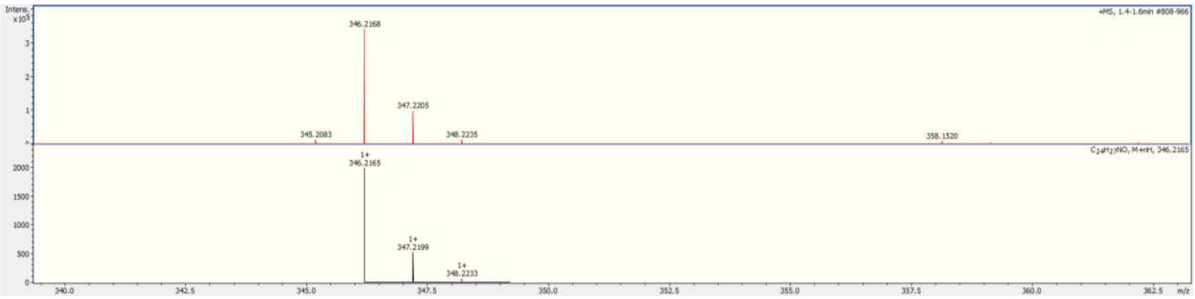

Figure S317: HRMS spectra for compound 9n.

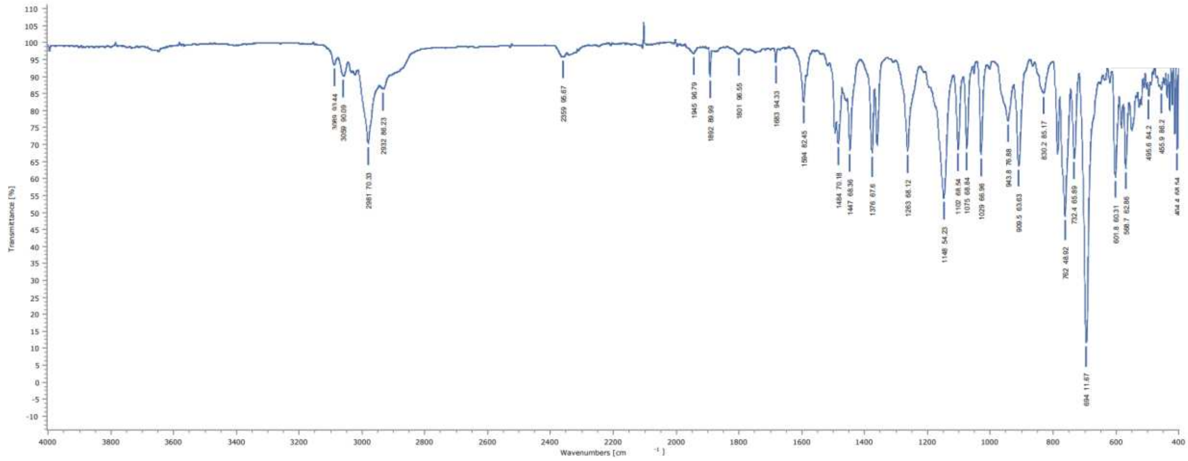

Figure S318: IR spectra for compound 9n.

11.4.30. N,O-bis(2,3-dihydro-1H-inden-1-yl)-N-phenylhydroxylamine **9o**

<u>Mixture of Diastereoisomers</u>

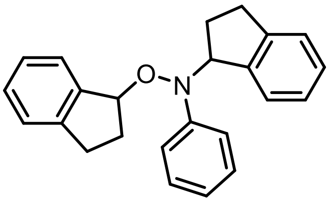

Data Collected Before Zinc Reduction

yellow oil (230 mg, 67%)

^{1}H NMR (CD$_3$OD, 500 MHz) δ 4.47 (d, J = 6.8 Hz, 1H, Ar-H), 7.38 – 7.11 (m, 20H, Ar-H), 7.05 – 6.95 (m, 4H, Ar-H), 6.68 (d, J = 5.8 Hz, 1H, Ar-H), 5.05 (dd, J = 8.4, 2.8 Hz, 1H, OC-H), 4.98 (dd, J = 8.3, 2.6 Hz, 1H, OC-H), 4.97 (app. br s, 1H, NC-H), 4.67 (d, J = 6.0 Hz, 1H, NC-H), 2.98 (app. dt, J = 16.3, 8.3 Hz, 1H, CH$_2$), 2.88 (app. dtd, J = 16.5, 8.2, 4.8 Hz, 2H, CH$_2$), 2.74 (ddd, J = 15.8, 9.3, 3.2 Hz, 1H, CH$_2$), 2.64 (ddd, J = 12.9, 9.3, 4.6 Hz, 1H, CH$_2$), 2.60 (ddd, J = 15.9, 8.6, 2.9 Hz, 1H, CH$_2$), 2.52 (ddd, J = 15.8, 10.0, 5.9 Hz, 1H, CH$_2$), 2.24 – 2.15 (m, 2H, CH$_2$), 2.03 (app. ddt, J = 13.4, 7.8, 2.6 Hz, 1H, CH$_2$), 1.90 – 1.75 (m, 5H, CH$_2$).

^{13}C{^{1}H} NMR (CD$_3$OD, 126 MHz) δ 153.86, 152.98, 147.17, 147.13, 146.29, 146.11, 143.22, 143.15, 143.11, 142.26, 130.07, 129.66, 129.57, 129.49, 129.16, 129.06, 127.20, 127.08, 127.04, 127.03,

127.01, 126.98, 126.88, 126.83, 125.63, 125.53, 125.50, 125.47, 125.24, 123.77, 120.39, 120.08, 88.69, 86.78, 75.16, 74.79, 32.49, 32.39, 31.85, 31.03, 30.87, 27.19, 26.47

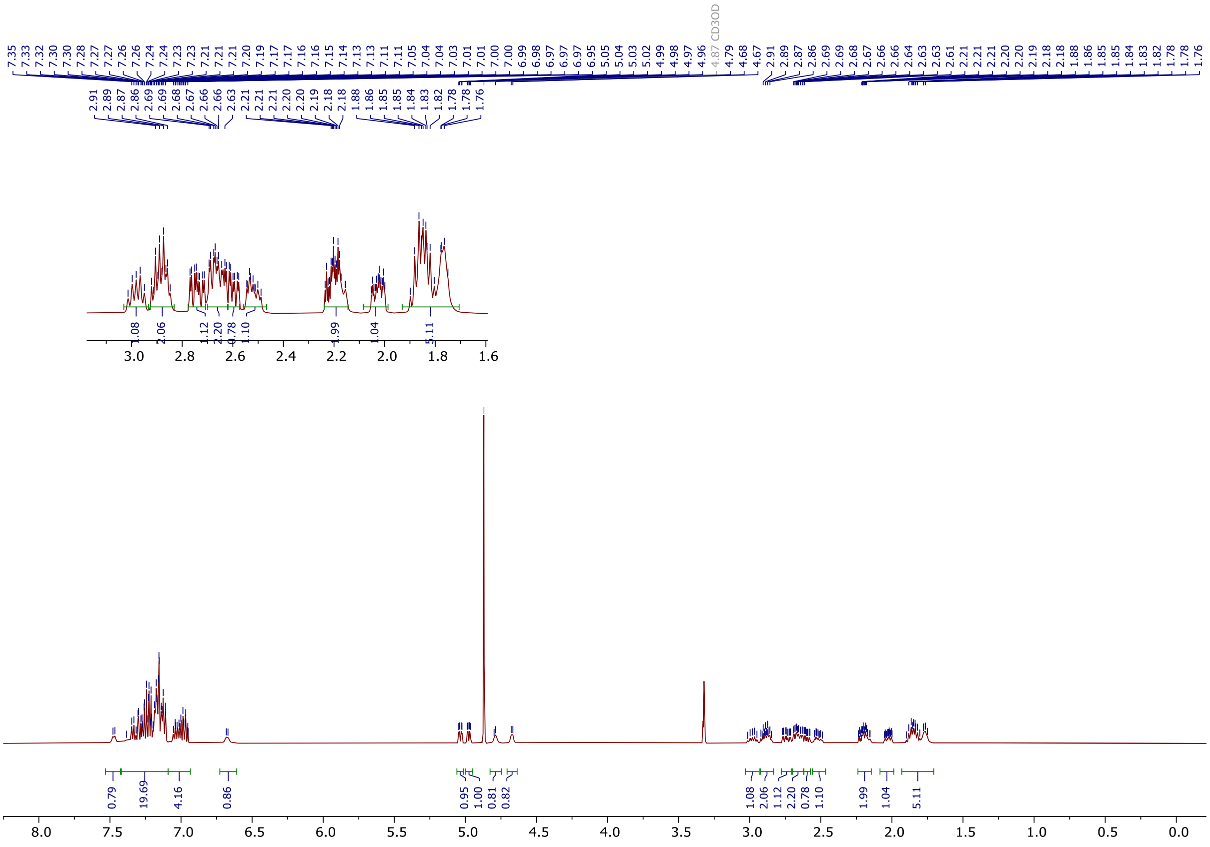

Figure S319: ^{1}H NMR Spectrum of 9o in CD_3OD after isolation via column chromatography.

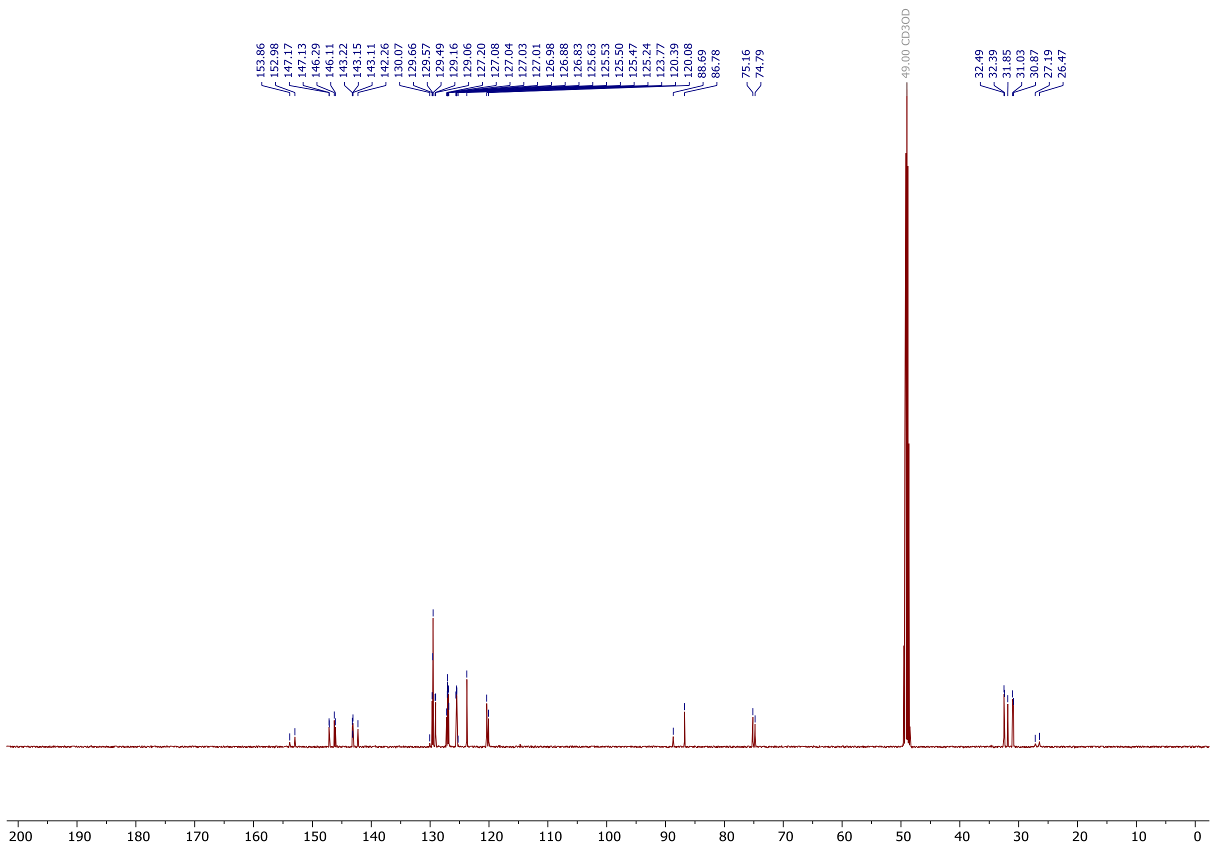

Figure S320: ^{13}C NMR Spectrum of 9o in CD_3OD after isolation via column chromatography.

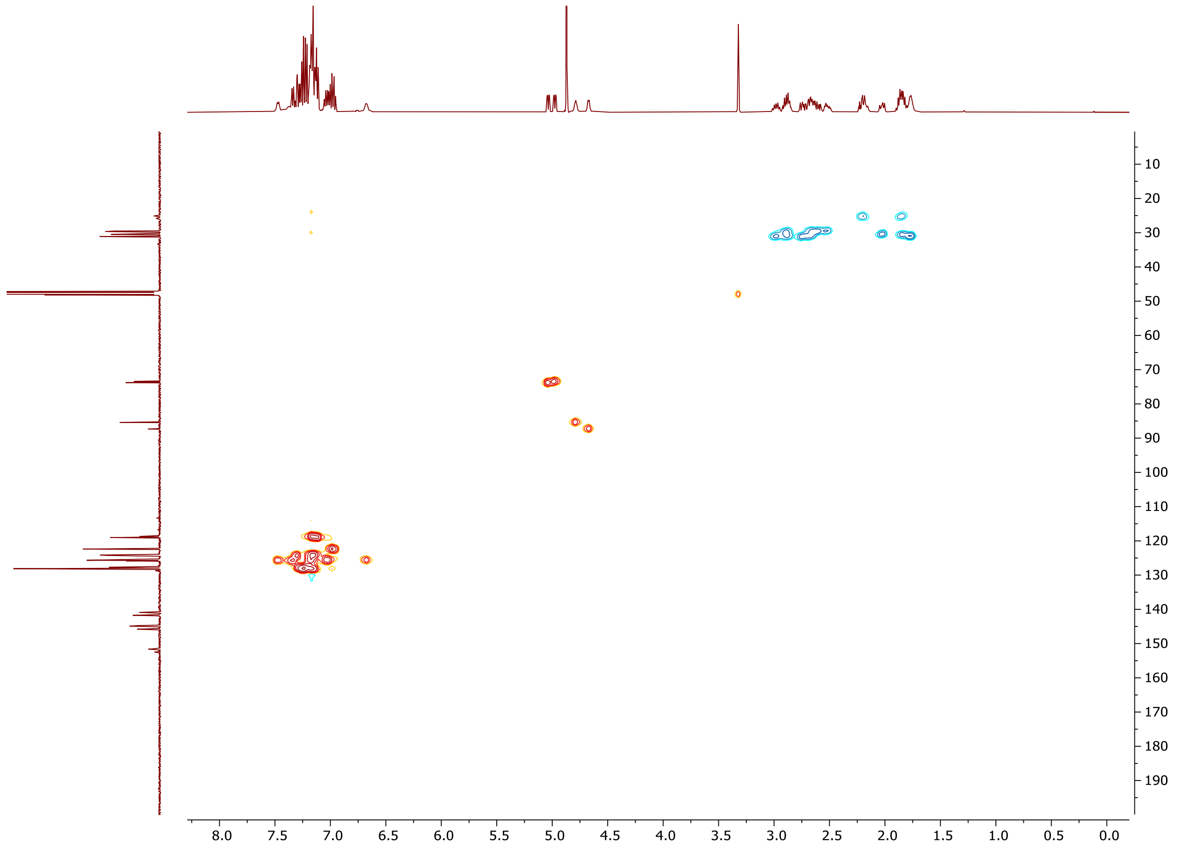

Figure S321: ^{1}H-^{13}C HSQC NMR Spectrum of 9o in CD_3OD after isolation via column chromatography.

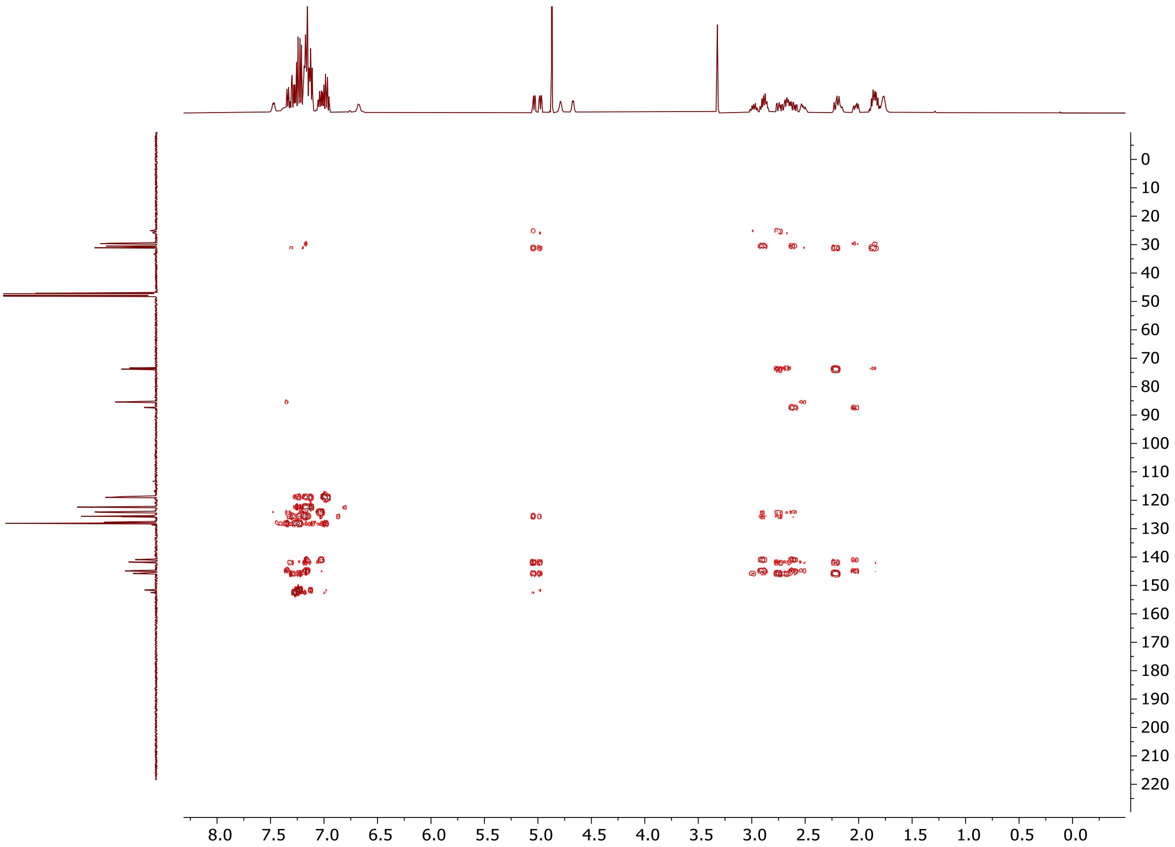

Figure S322: ^{1}H-^{13}C HMBC NMR Spectrum of 9o in CD_3OD after isolation via column chromatography.

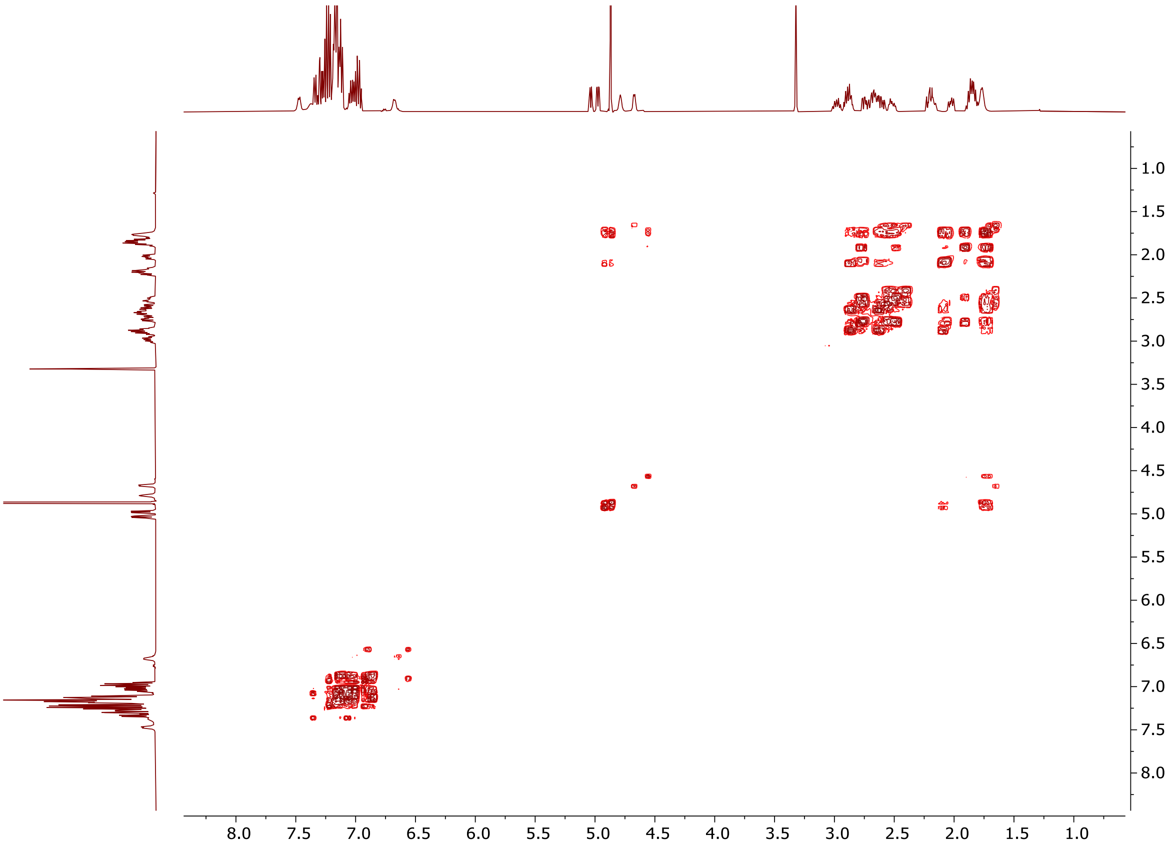

Figure S323: ^{1}H-^{1}H COSY NMR Spectrum of 9o in CD_3OD after isolation via column chromatography

11.4.31. N-(4-(methylthio)phenyl)-N,O-bis(2-phenylpropan-2-yl)hydroxylamine **9s**

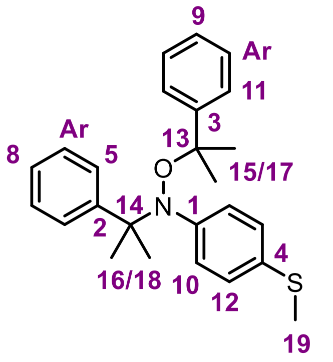

Colourless oil (278 mg, 71%)

R_f = 0.31 (30% DCM / 70% hexane)

^{1}H NMR (CDCl$_3$, 500 MHz) δ 7.54 (d, J = 7.4 Hz, 2H, C^{11}-H), 7.53 (d, J = 7.1 Hz, 2H, C^5-H), 7.41 – 7.31 (m, 2H, Ar, Ar, C^8-H, C^9-H), 7.04 (d, J = 8.6 Hz, C^{12}-H), 6.95 (d, J = 8.6 Hz, 2H, C^{10}-H), 2.47 (s, 3H, C^{19}-H), 1.65 (s, 3H, $C^{15/17}$-H), 1.60 (s, 3H, $C^{16/18}$-H), 1.54 (s, 3H, $C^{15/17}$), 1.33 (s, 3H, $C^{16/18}$-H).

^{13}C{^{1}H} NMR (CDCl$_3$, 126 MHz) δ 149.5 (C^1), 146.8 (C^2), 145.5 (C^3), 133.6 (C^4), 128.1 (Ar), 127.8 (Ar), 127.5 (Ar), 126.9 (C^8), 126.8 (C^9), 126.3 (C^{10}), 125.7 (C^{11}), 125.6 (C^{12}), 81.4 (C^{13}), 65.3 (C^{14}), 28.3 (C^{15}), 27.3 (C^{16}), 27.3 (C^{17}), 24.0 (C^{18}), 16.2 (C^{19}).

HRMS (ESI+): calcd for [M, $C_{25}H_{29}NOS$]$^+$ 392.2043, found 392.2055.

IR (Neat): 3088, 2981, 1893, 1486, 1149, 760, 695 cm^{-1}.

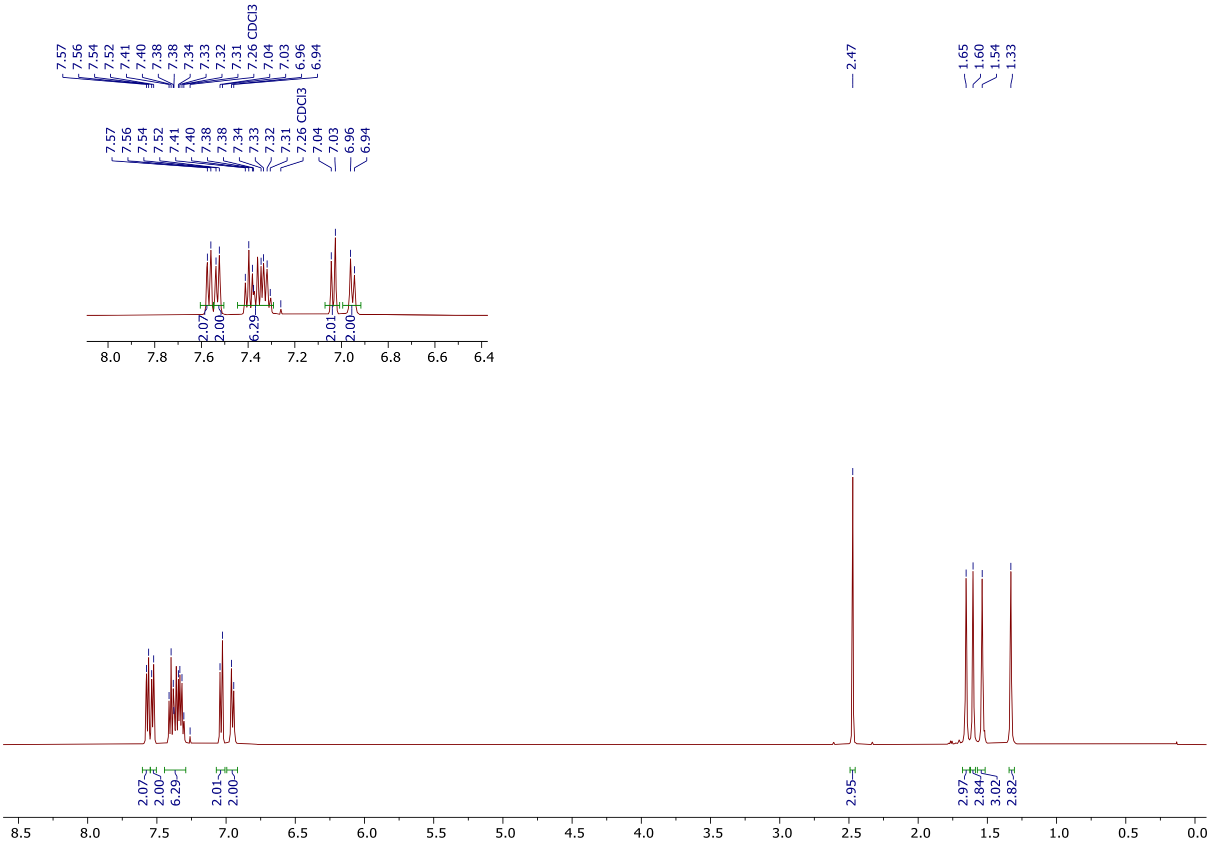

Figure S324: ^{1}H NMR Spectrum of 9s in CDCl$_3$ after isolation via column chromatography.

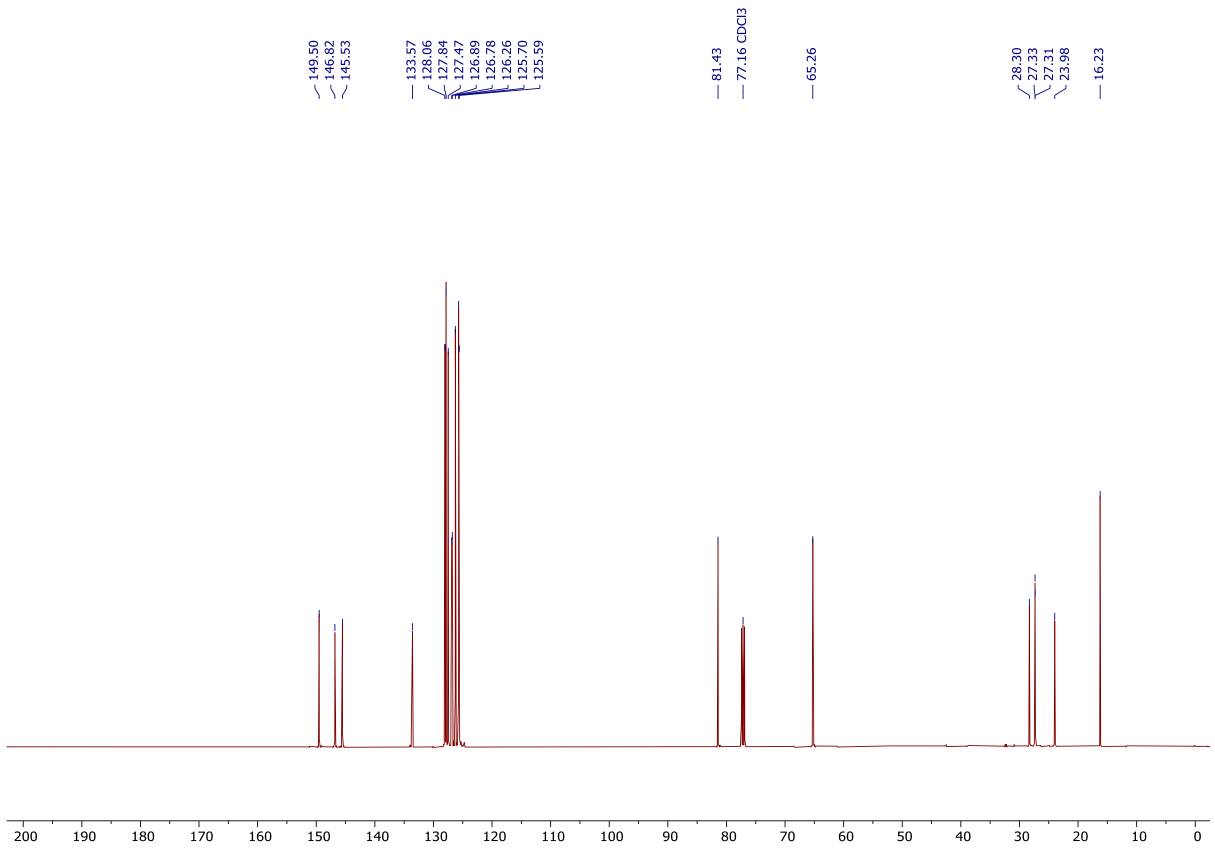

Figure S325: ^{13}C NMR Spectrum of 9s in $CDCl_3$ after isolation via column chromatography.

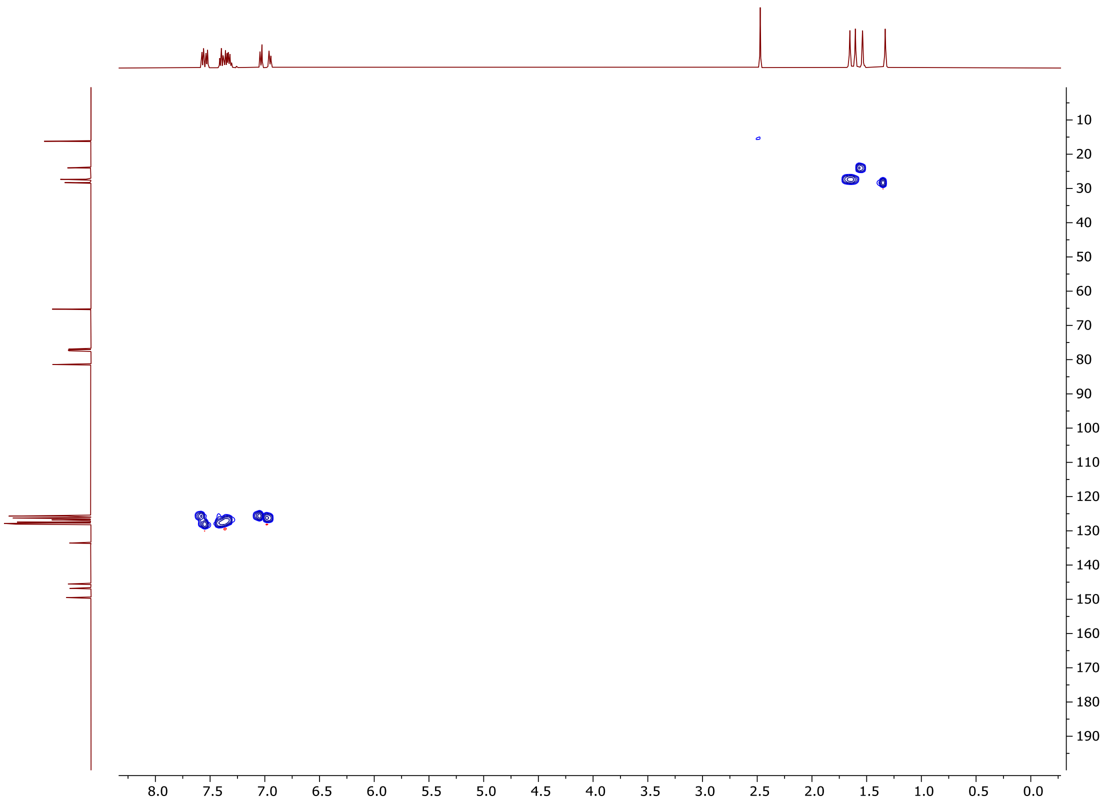

Figure S326: ^{1}H-^{13}C *HSQC* NMR Spectrum of 9s in $CDCl_3$ after isolation via column chromatography.

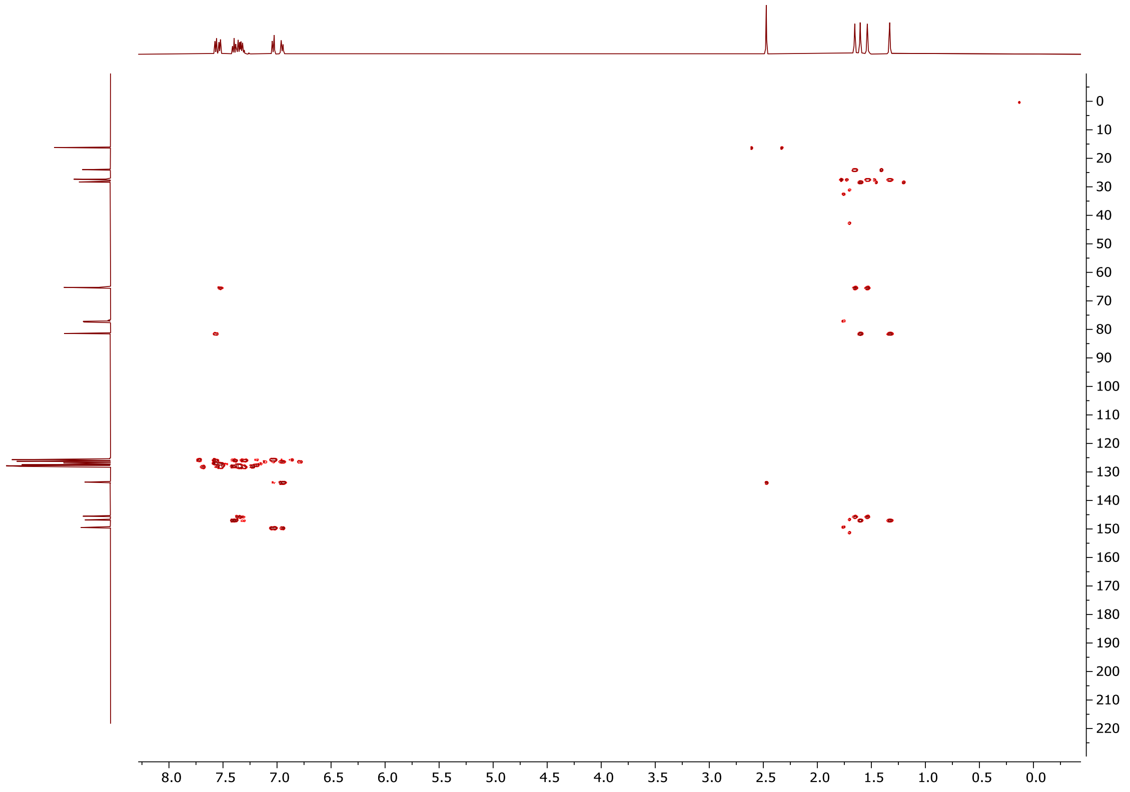

Figure S327: ^{1}H-^{13}C HMBC NMR Spectrum of 9s in $CDCl_3$ after isolation via column chromatography.

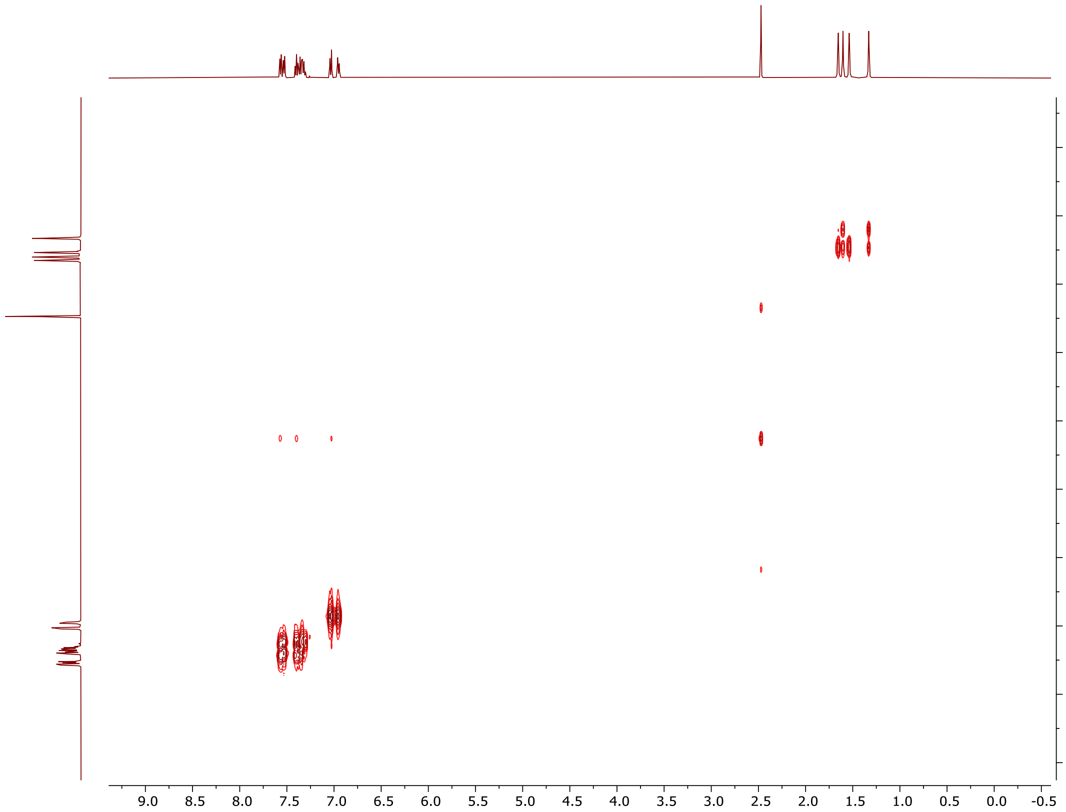

Figure S328: ^{1}H-^{1}H COSY NMR Spectrum of 9s in $CDCl_3$ after isolation via column chromatography.

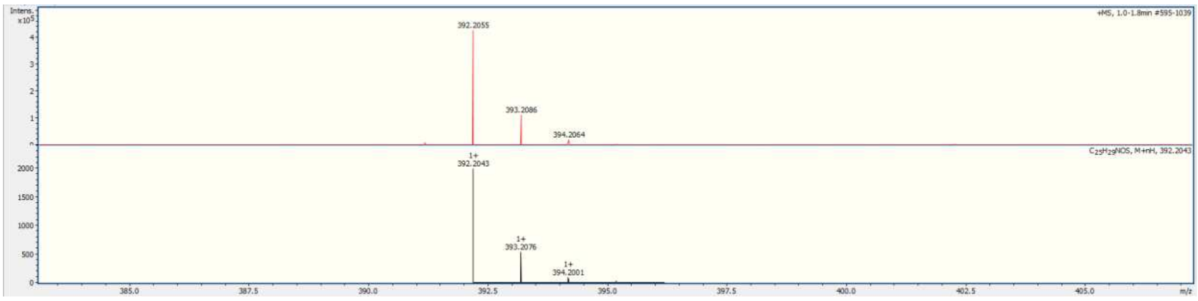

Figure S 329: HRMS spectra for compound 9s.

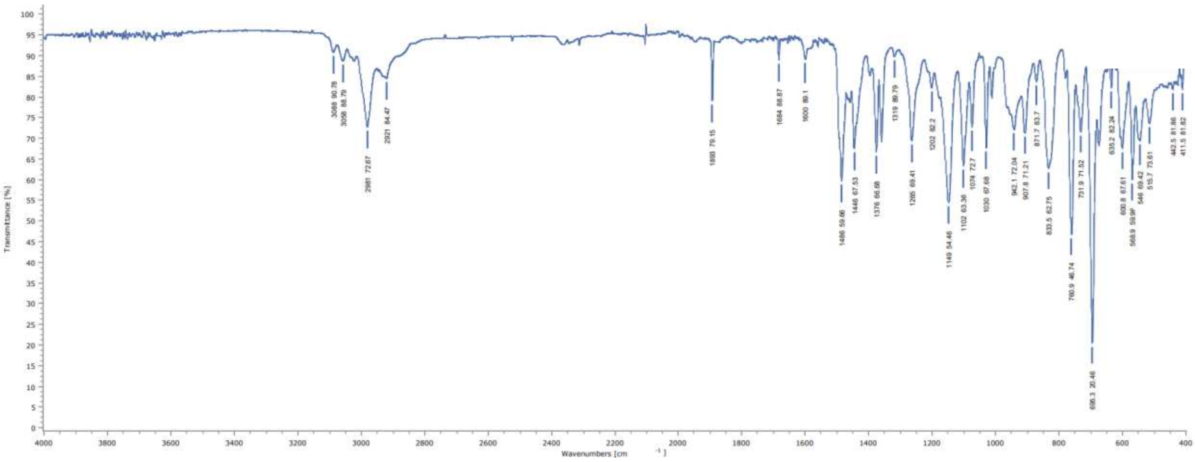

Figure S330: IR spectra for compound 9s.

11.4.32. N,O-bis(2,3-dihydro-1H-inden-1-yl)-N-(4-(trifluoromethyl)phenyl)hydroxylamine **9w**

Mixture of Diastereisomers

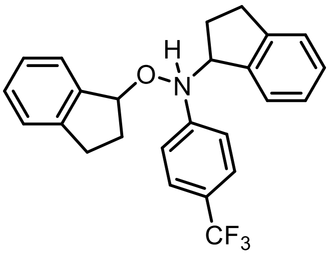

Exact Mass: 410.1732

Data Collected Before Zinc Reduction

Colourless oil (221 mg, 54%)

R_f = 0.65 (30% DCM / 70% hexane)

^{1}H NMR (CDCl$_3$, 500 MHz) δ 7.75 (dd, J = 17.3, 7.3 Hz, 1H, Ar-H), 7.63 – 7.04 (m, 21H, Ar-H), 6.64 (dd, J = 57.7, 7.3 Hz, 1H, Ar-H), 5.21 (d, J = 8.3 Hz, 1H, OC-H), 5.15 (d, J = 8.6 Hz, 1H, OC-H), 4.63 (d, J = 6.6 Hz, 1H, NC-H), 4.47 (d, J = 6.1 Hz, 1H, NC-H), 3.25 – 2.65 (m, 6H, CH_2), 2.58 – 2.49 (m, 1H, CH_2), 2.33 – 1.47 (m, 7H, CH_2).

^{13}C{^{1}H} NMR (CDCl$_3$, 126 MHz) δ 155.51, 154.93, 145.93, 145.31, 145.12, 141.65, 141.43, 140.76, 128.96, 128.79, 128.69, 128.57, 128.41, 126.40, 126.32, 126.21, 126.15, 126.09, 126.02, 126.00,

125.96, 125.92, 125.88, 125.85, 124.99, 124.96, 124.83, 124.81, 123.30, 118.29, 118.13, 88.15, 86.48, 73.78, 73.26, 31.82, 31.74, 31.36, 31.07, 30.82, 30.42, 30.26, 29.86, 25.90.

HRMS (ESI+): calcd for [M, $C_{25}H_{23}F_3NO$]$^+$ 410.1732, found 410.1730.

IR (Neat): 2940, 2849, 1613, 1478, 1321, 1109 cm^{-1}.

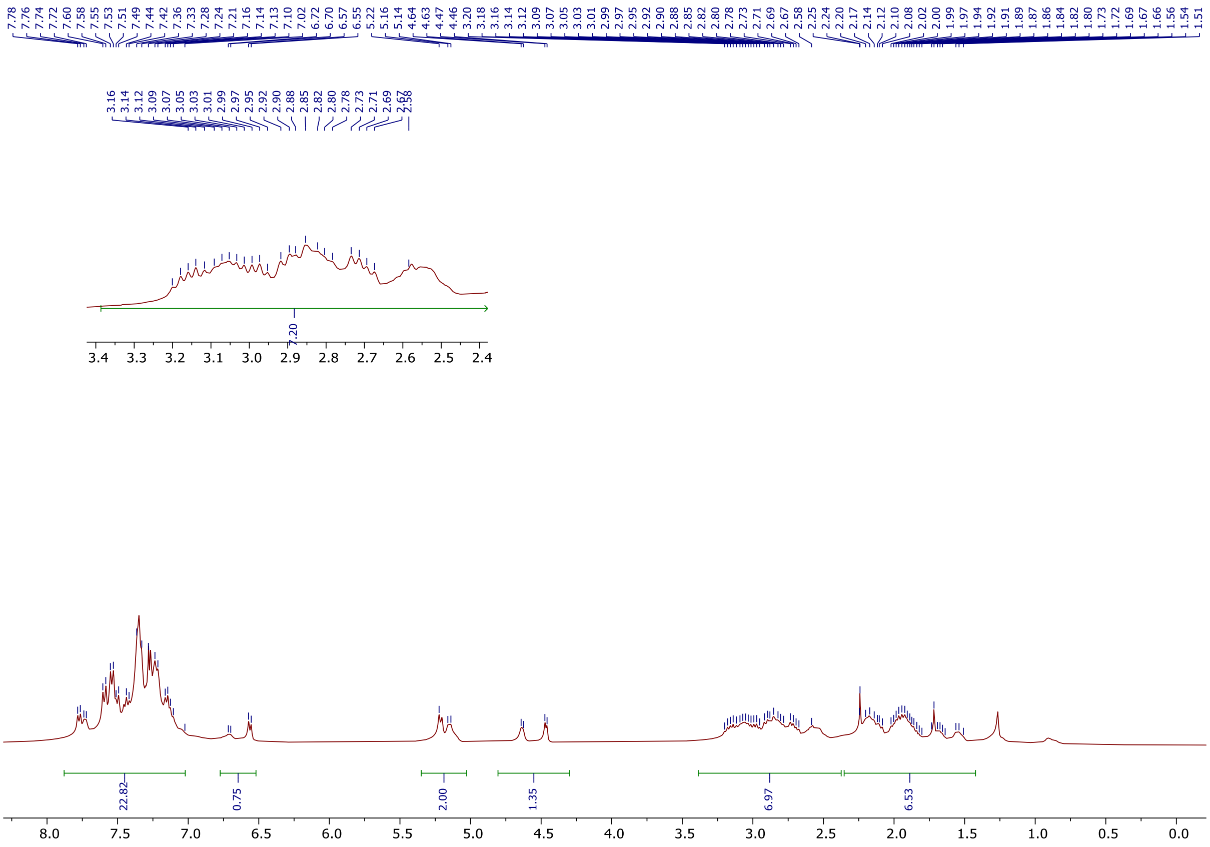

Figure S331: ^{1}H NMR Spectrum of 9w in $CDCl_3$ after isolation via column chromatography.

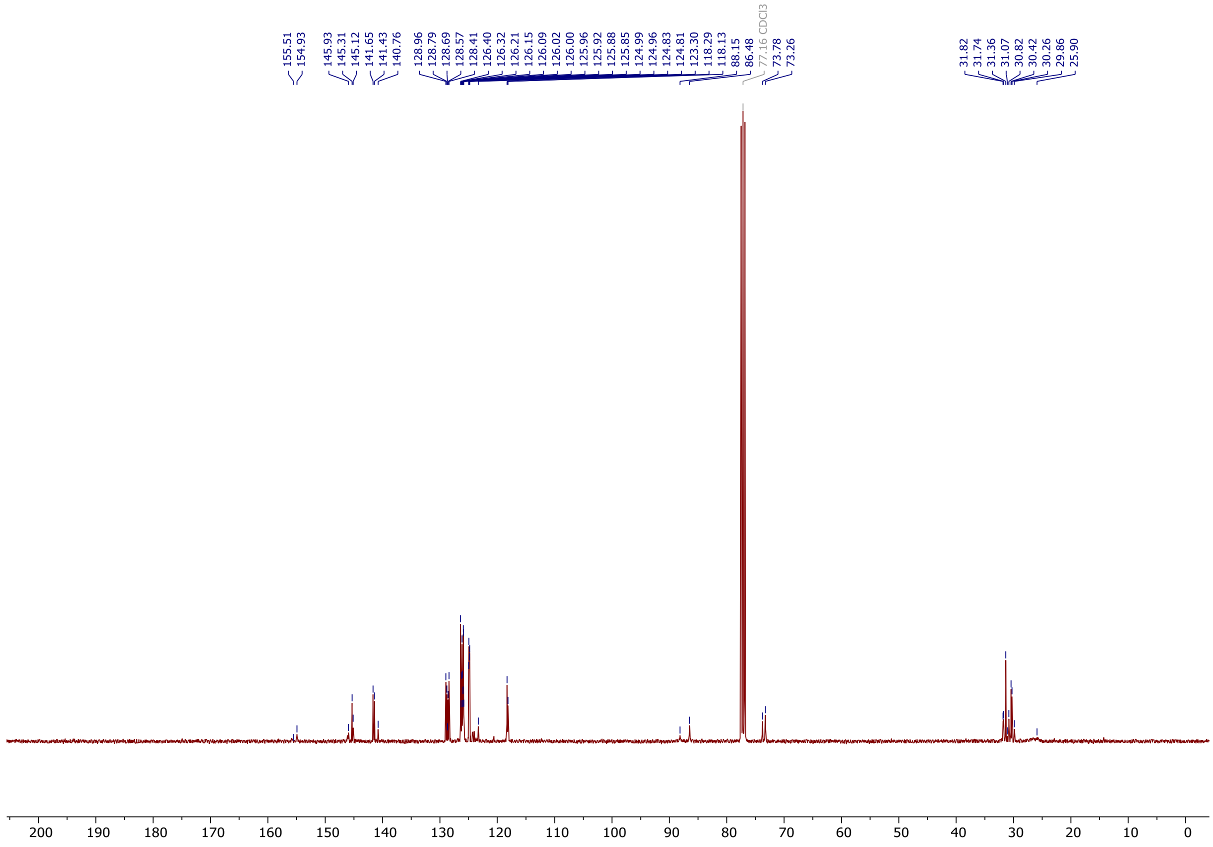

Figure S332: ^{13}C NMR Spectrum of 9w in $CDCl_3$ after isolation via column chromatography.

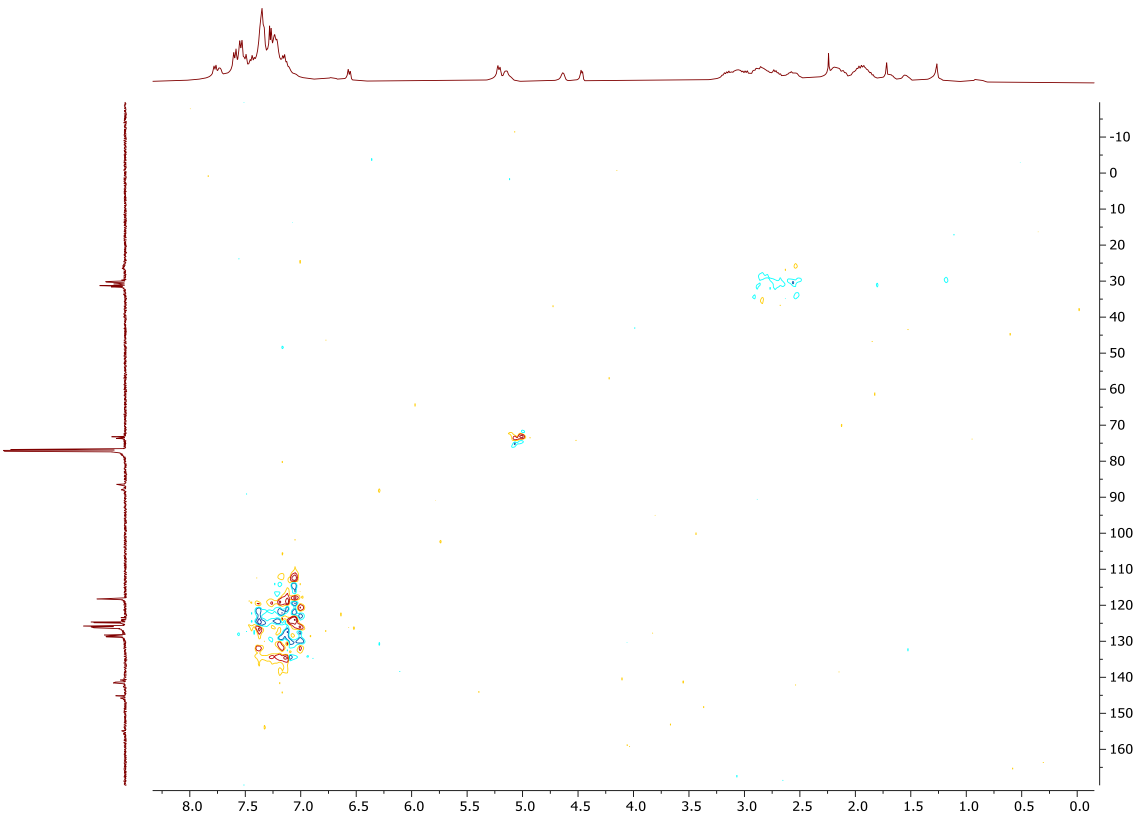

Figure S333: ^{1}H-^{13}C HSQC NMR Spectrum of 9w in $CDCl_3$ after isolation via column chromatography.

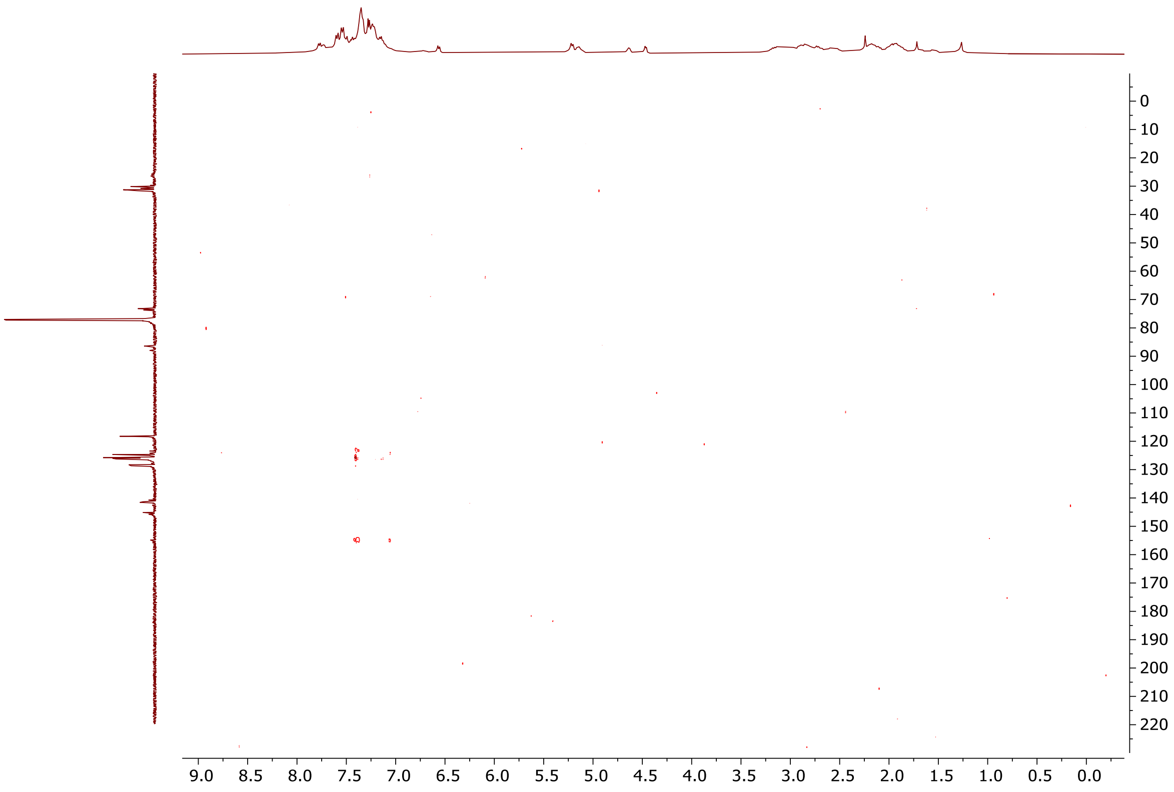

Figure S334: ^{1}H-^{13}C HMBC NMR Spectrum of 9w in $CDCl_3$ after isolation via column chromatography.

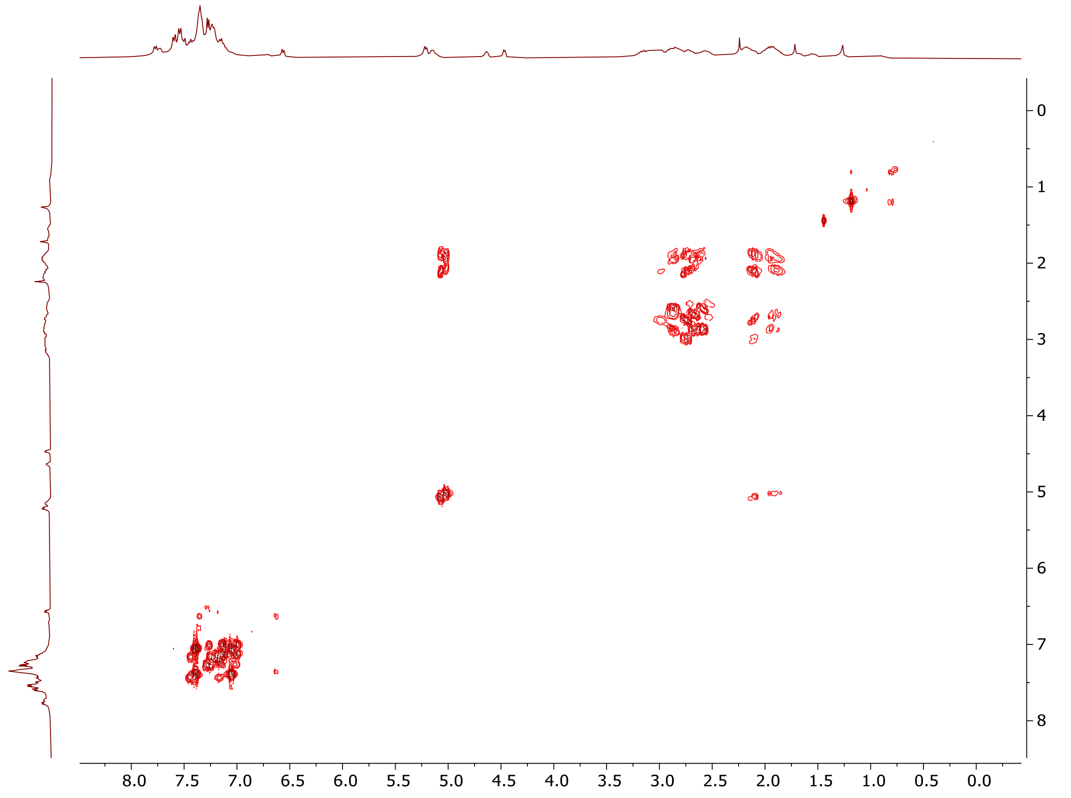

Figure S335: ^{1}H-^{1}H COSY NMR Spectrum of 9w in $CDCl_3$ after isolation via column chromatography.

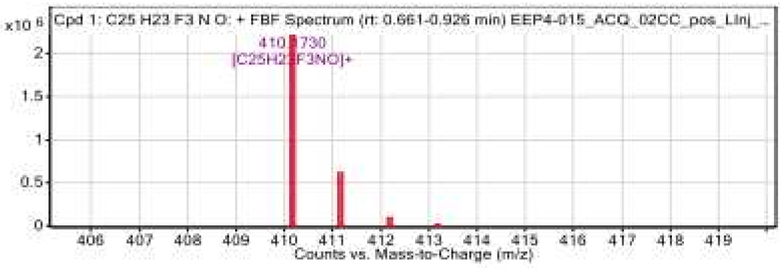

Figure S 336: HRMS spectra of compound 9w.

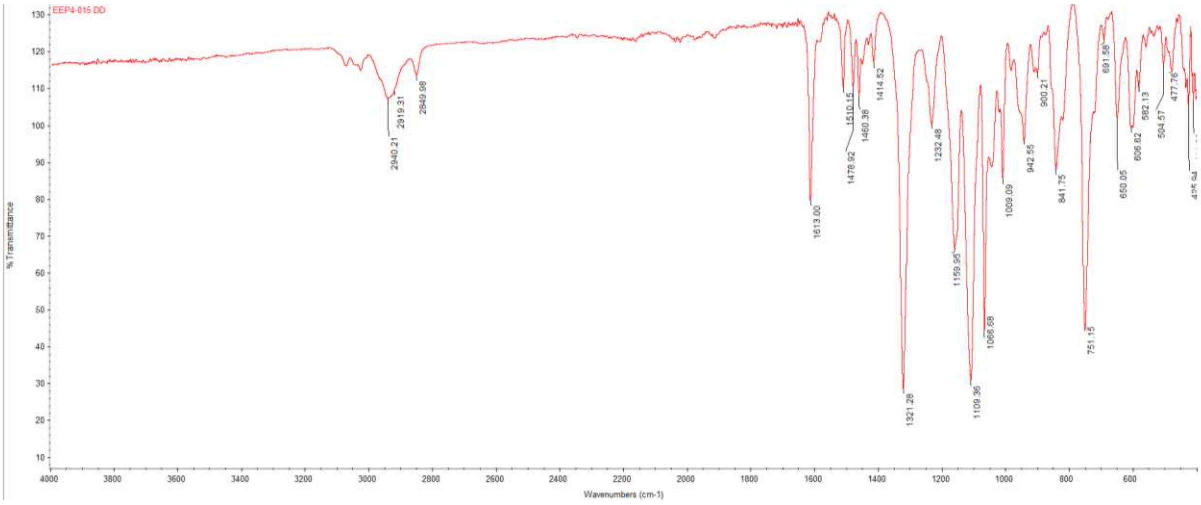

Figure S337: IR Spectra of compound 9w.

11.4.33. N,O-bis(1-phenylpropan-2-yl)-N-(4-(trifluoromethyl)phenyl)hydroxylamine **9x**

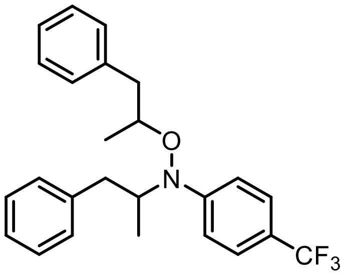

Colourless oil (65 mg, 16%)

R_f = 0.43 (30% DCM / 70% hexane)

^{1}H NMR ($CDCl_3$, 400 MHz) δ 7.46 (app. d, *J* = 7.7 Hz, 5H), 7.36 – 7.08 (m, 21H), 7.03 (app. d, *J* = 7.5 Hz, 2H), 4.01 (app. br s, 2H), 3.68 (app. br s, 2H), 3.01 (dd, *J* = 13.4, 7.1 Hz, 1H), 2.91 – 2.31 (s, 7H), 1.16 (app. br s, 3H), 0.95 (d, *J* = 6.6 Hz, 3H).

^{13}C{^{1}H} NMR ($CDCl_3$, 101 MHz) δ 155.55, 139.87, 139.85, 139.82, 139.72, 139.04, 138.93, 129.84, 129.29, 129.28, 129.26, 129.25, 128.66, 128.64, 128.56, 128.45, 128.42, 128.38, 126.43, 126.38, 126.18, 126.00, 125.93, 125.89, 124.13, 123.81, 123.30, 118.27, 115.38, 112.36, 112.03, 111.82, 66.18, 55.48, 55.17, 42.46, 42.42, 41.89, 41.68, 19.17, 18.70, 19.47, 18.64.

HRMS (ESI+): calcd for [M, $C_{25}H_{26}F_3NO$]$^+$ 414.2045, found 414.2041.

IR (Neat): 2973, 1673, 1613, 1511, 1322, 1109 cm^{-1}.

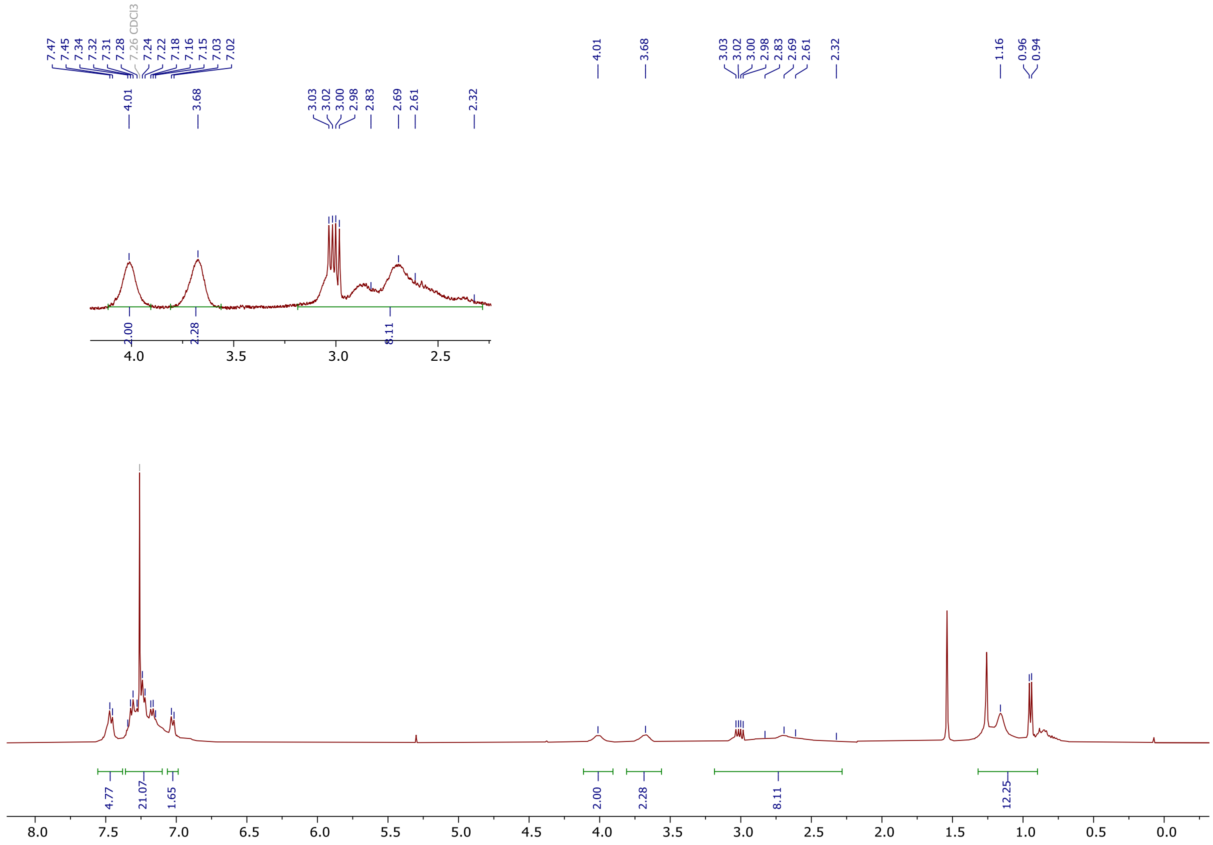

Figure S338: ^{1}H NMR Spectrum of 9x in $CDCl_3$ after isolation via column chromatography.

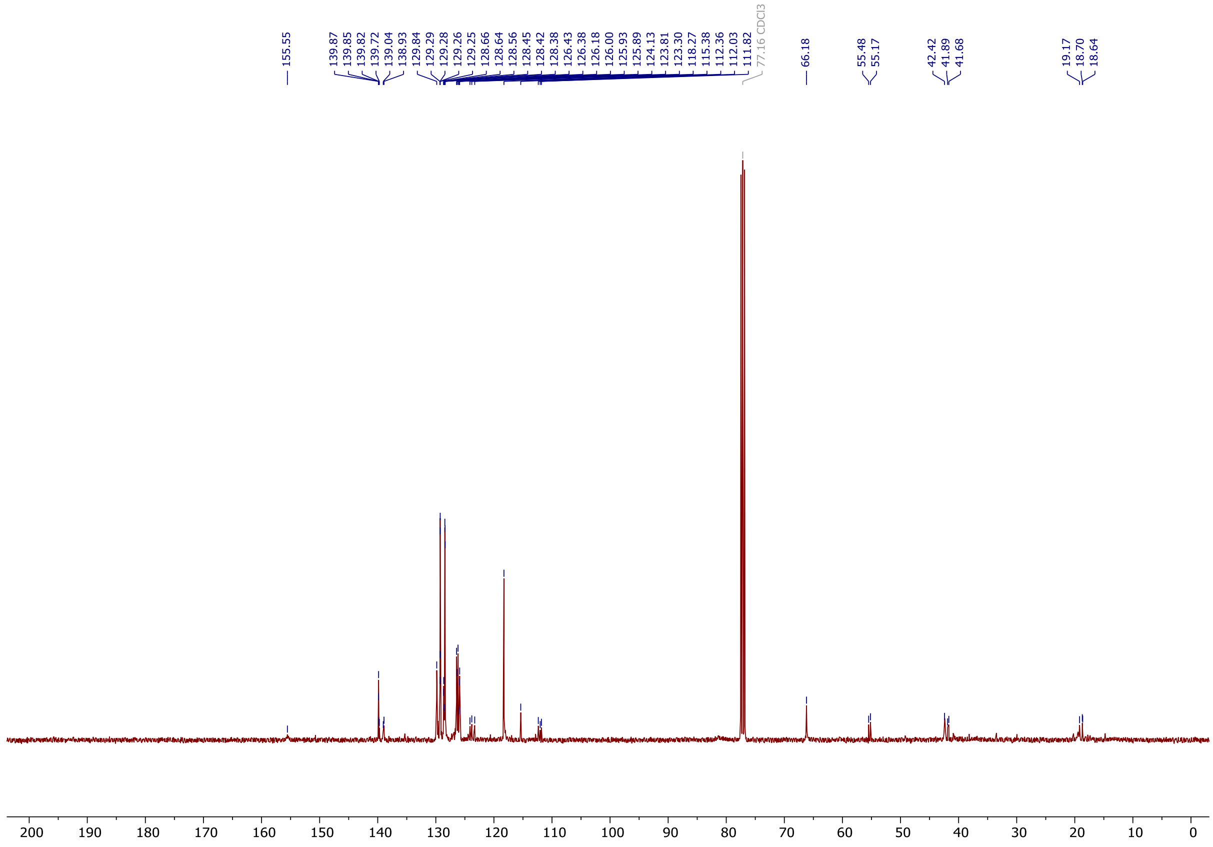

Figure S339: ^{13}C NMR Spectrum of 9x in $CDCl_3$ after isolation via column chromatography.

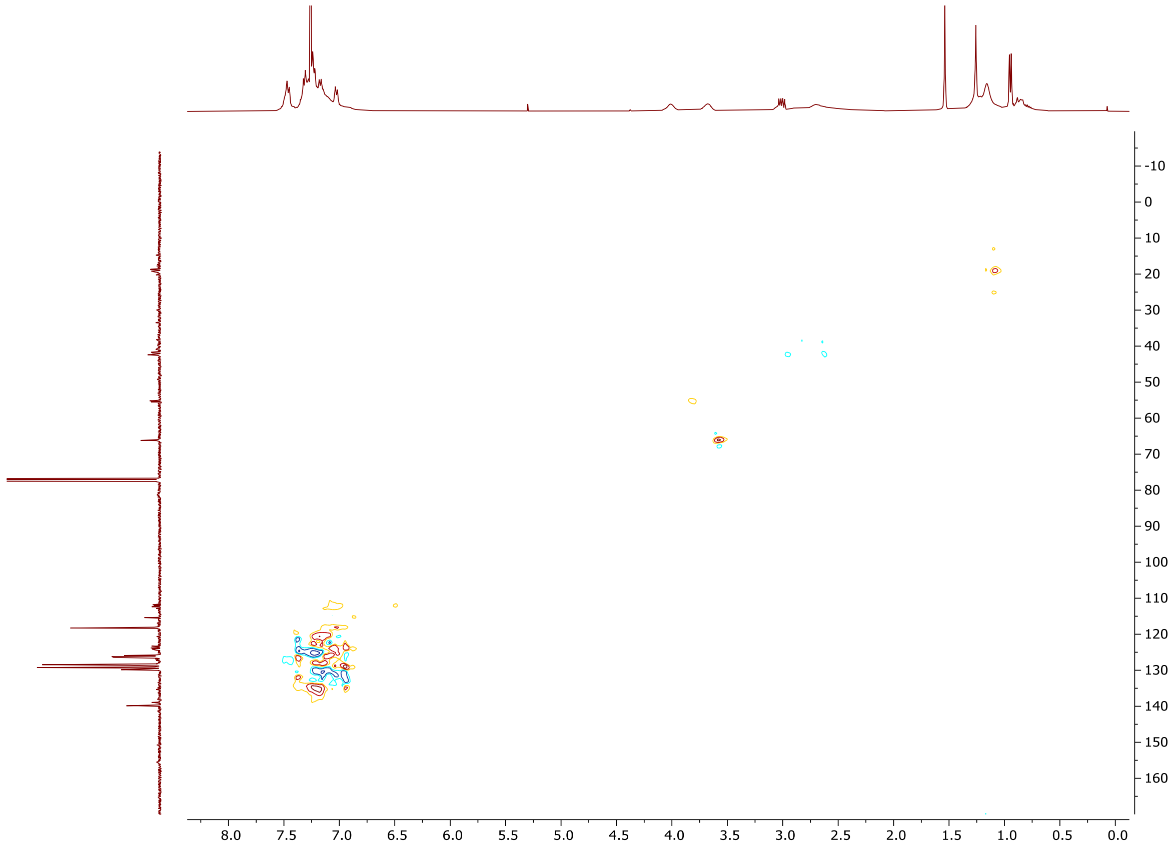

Figure S340: ^{1}H-^{13}C HSQC NMR Spectrum of 9x in $CDCl_3$ after isolation via column chromatography.

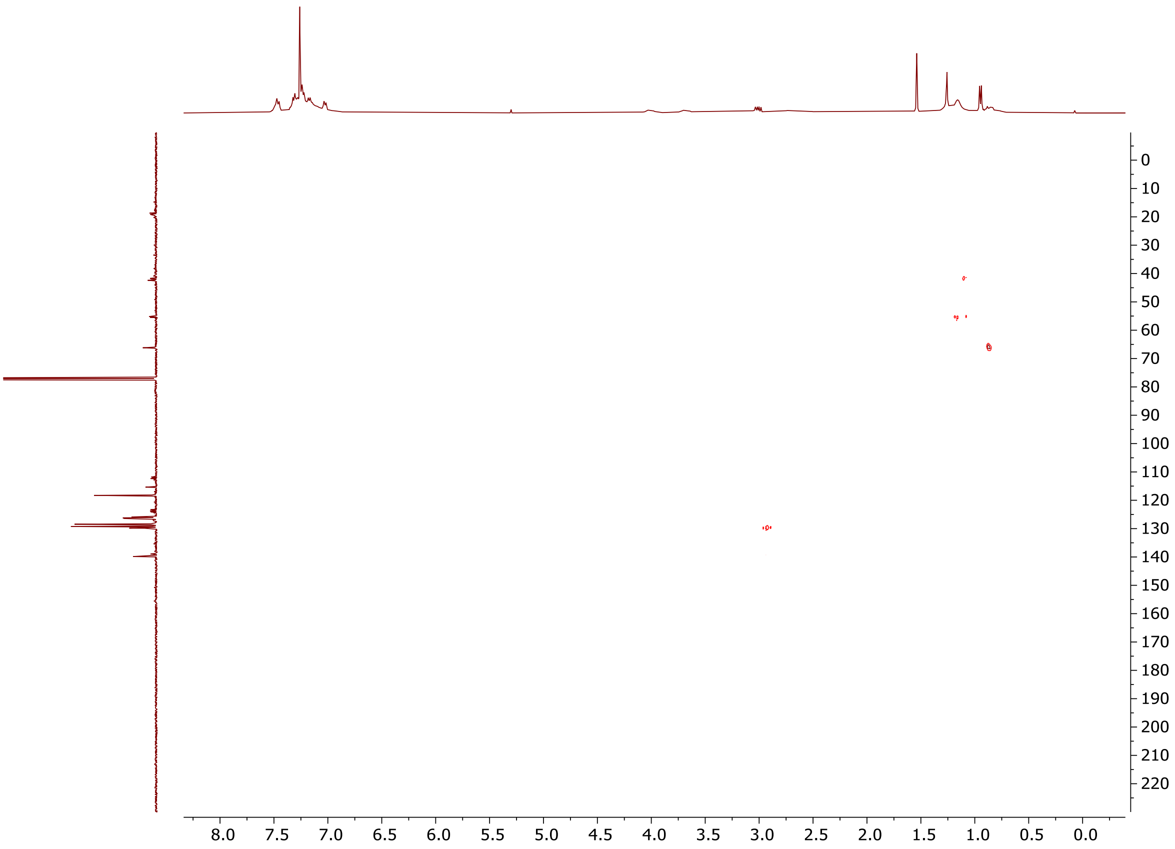

Figure S341: ^{1}H-^{13}C HMBC NMR Spectrum of 9x in $CDCl_3$ after isolation via column chromatography.

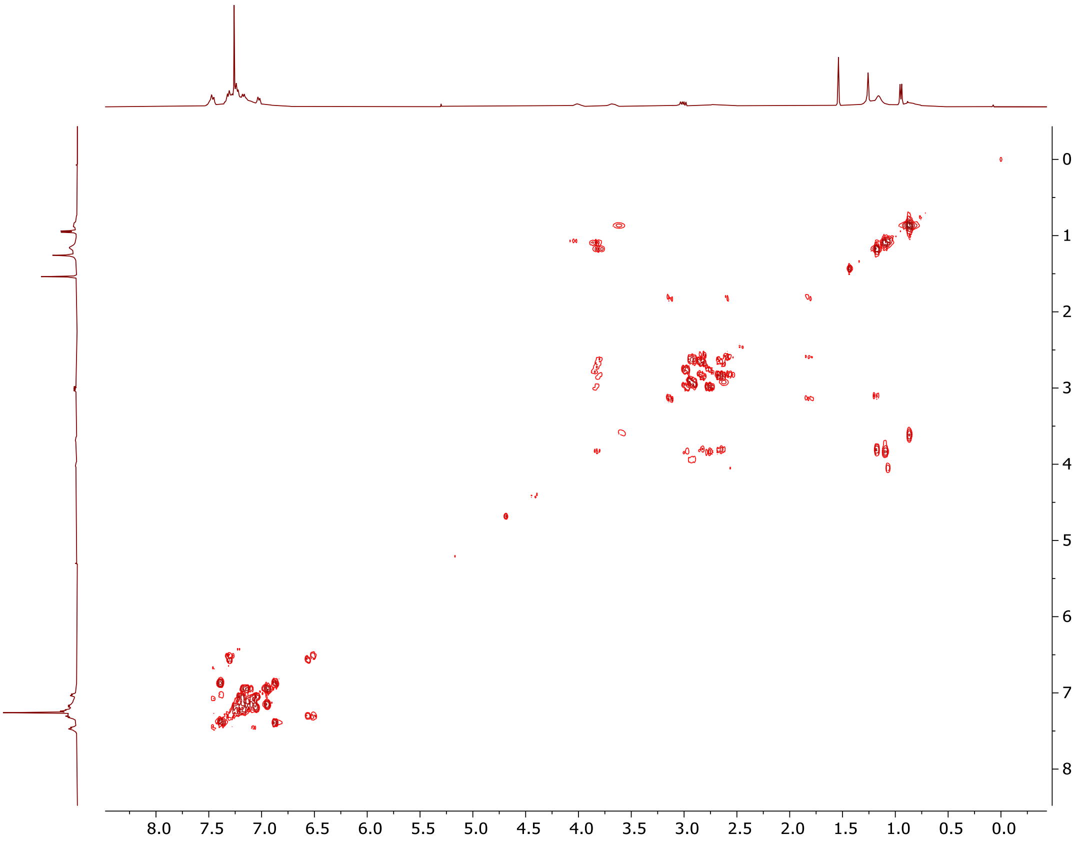

Figure S342: ^{1}H-^{1}H COSY NMR Spectrum of 9x in $CDCl_3$ after isolation via column chromatography.

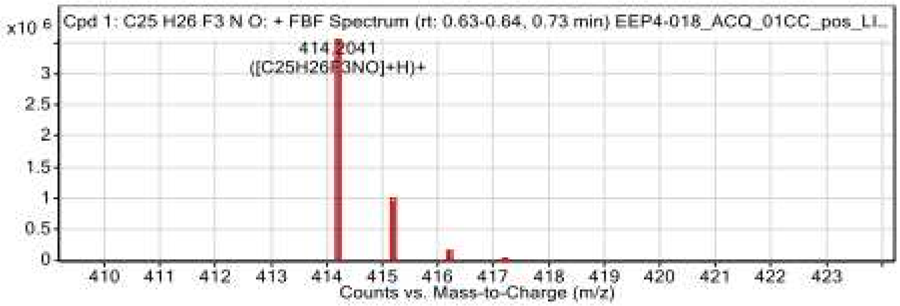

Figure S 343: HRMS Spectra of compound 9x.

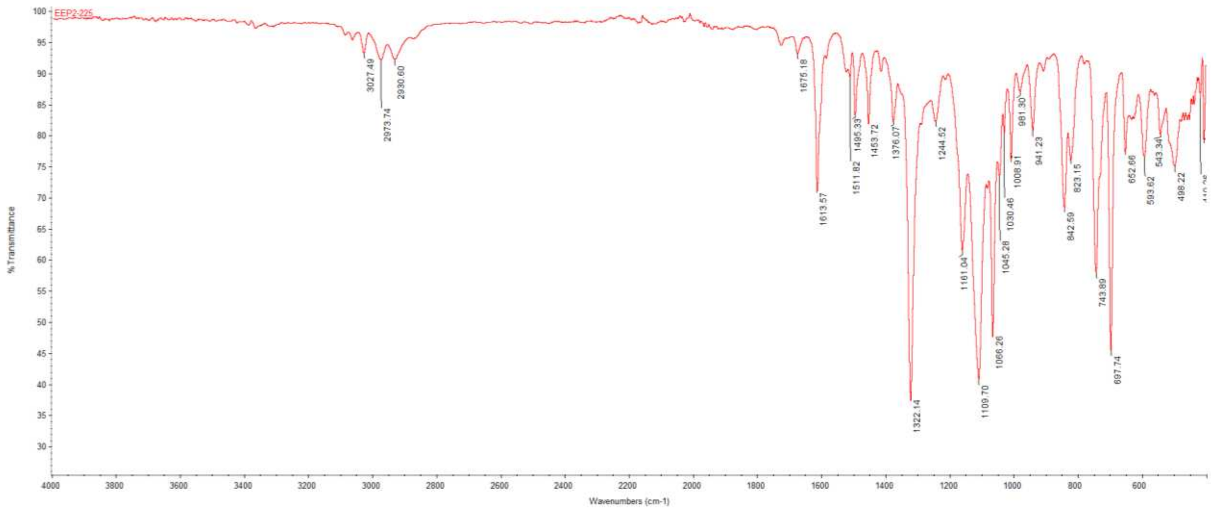

Figure S 344: IR Spectra of compound 9x.

11.4.34. N,O-bis(2,3-dihydro-1H-inden-1-yl)-N-(2-methylnaphthalen-1-yl)hydroxylamine **9y**

Mixture of diastereisomers

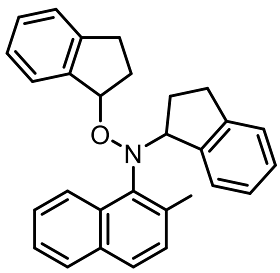

Colourless oil (184 mg,45%)

R_f = 0.57 (30% DCM / 70% hexane)

^{1}H NMR ($CDCl_3$, 500 MHz) δ 8.72 (app. d, *J* = 5.5 Hz, 1H, Ar-H), 8.48 (app. br s, 1H, Ar-H), 7.78 – 7.68 (m, 2H, Ar-H), 7.56 (app. dd, *J* = 8.5, 4.9 Hz, 2H), 7.37 – 6.71 (m, 21H, Ar-H), 6.24 (app. br s, 1H), 5.13 (app. q, *J* = 8.4, 7.4 Hz, 3H, OC-H/NC-H), 4.98 (br s, 1H, NC-H), 3.21 (app. tt, *J* = 16.7, 8.2 Hz, 2H, CH_2), 2.70 (ddd, *J* = 28.8, 14.9, 7.7 Hz, 6H, CH_2), 2.60 (s, 3H, CH_3), 2.49 (app. ddd, *J* = 16.3, 8.1, 5.0 Hz, 3H, CH_2), 2.14 – 1.94 (m, 4H, CH_2/CH_3), 1.84 (aap. ddq, *J* = 35.3, 14.2, 6.8 Hz, 2H, CH_2), 1.66 (app. ddq, *J* = 12.9, 8.5, 4.3 Hz, 2H, CH_2).

^{13}C{^{1}H} NMR ($CDCl_3$, 126 MHz) δ 146.13, 145.51, 144.97, 144.82, 142.50, 142.12, 142.04, 134.37, 133.79, 131.59, 131.36, 129.79, 129.25, 128.43, 128.40, 127.87, 127.79, 127.19, 126.93, 126.15, 126.12, 125.93, 125.74, 125.53, 125.41, 125.36, 124.91, 124.82, 124.80, 124.68, 124.43, 124.20, 86.84, 70.61, 31.99, 31.51, 31.22, 31.14, 30.58, 30.31, 29.86, 20.32, 19.24.

HRMS (ESI+): calcd for [M, $C_{29}H_{27}NO$]$^+$ 406.2171, found 406.2166.

IR (Neat): 2925, 1600, 1507, 1477, 1321, 942 cm^{-1}.

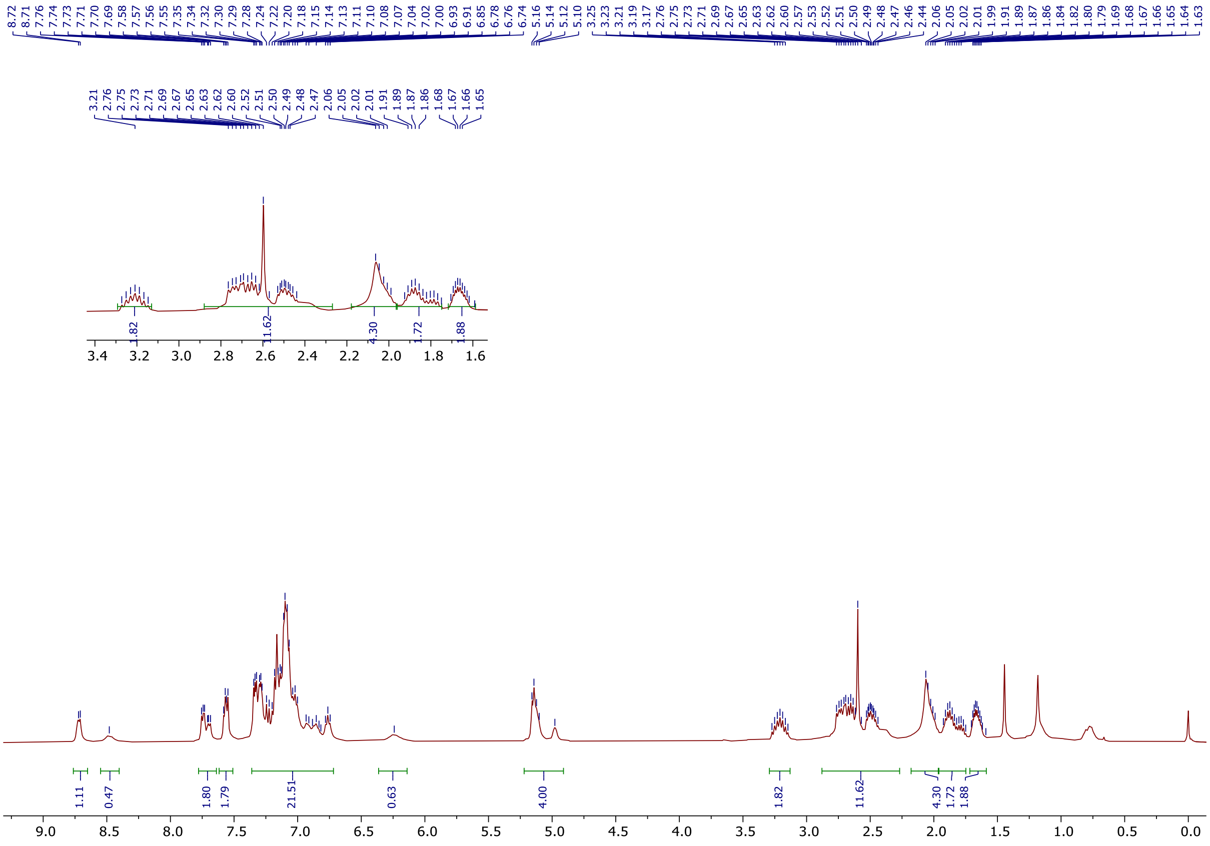

Figure S345: ^{1}H NMR Spectrum of 9y in $CDCl_3$ after isolation via column chromatography.

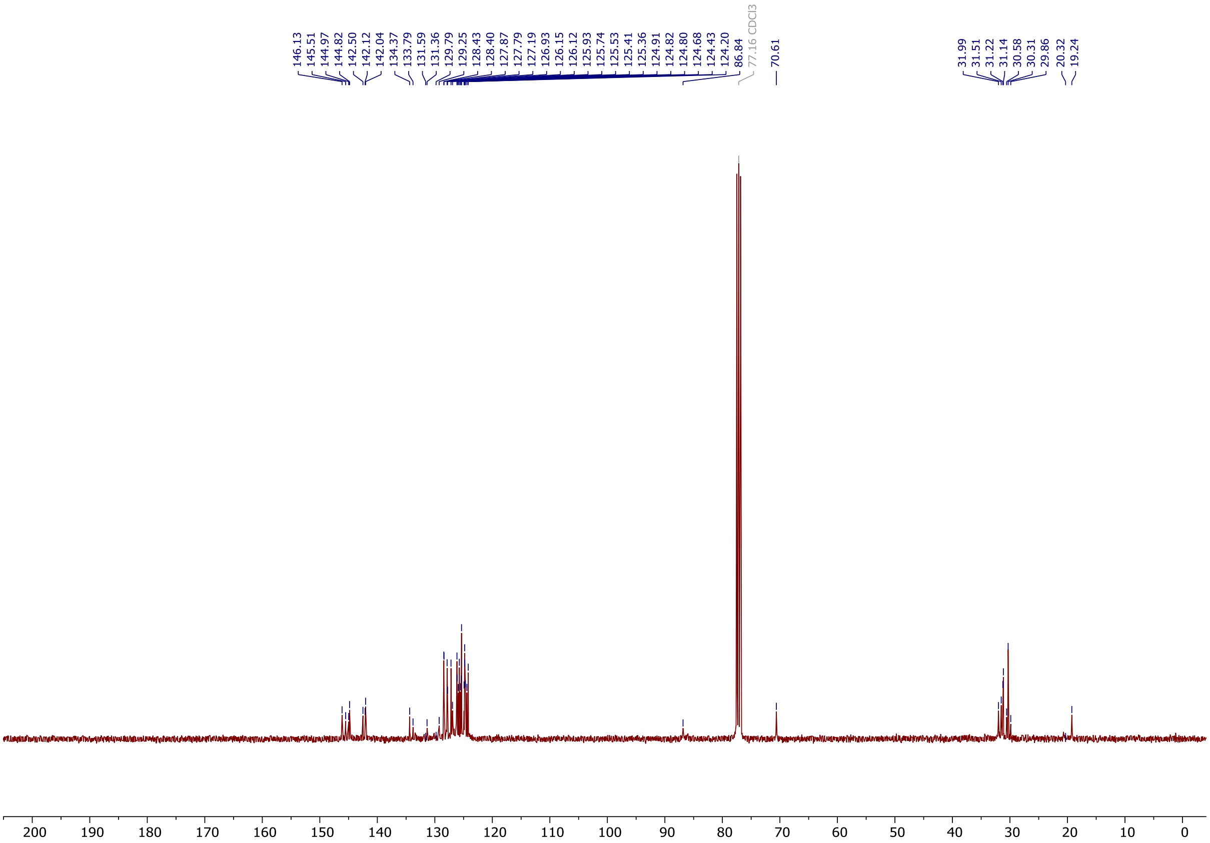

Figure S346: ^{13}C NMR Spectrum of 9y in $CDCl_3$ after isolation via column chromatography.

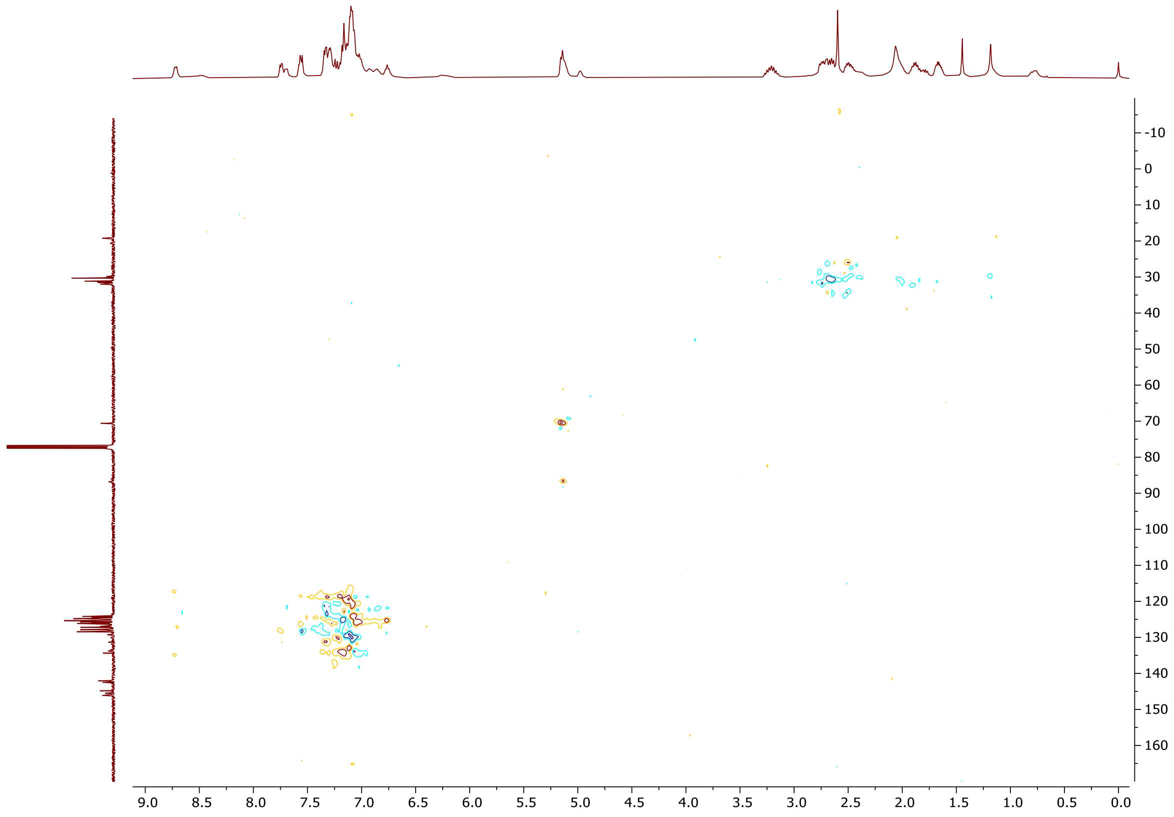

Figure S347: ^{1}H-^{13}C HSQC NMR Spectrum of 9y in $CDCl_3$ after isolation via column chromatography.

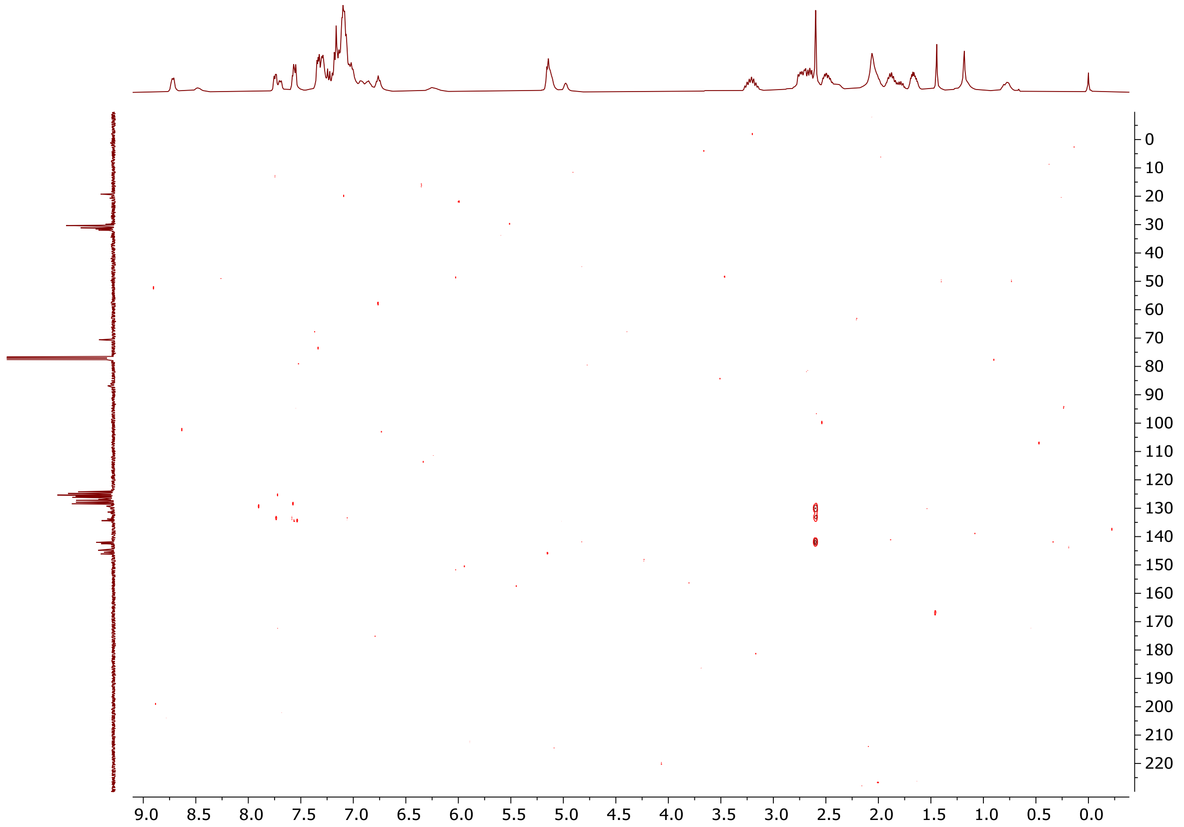

Figure S348: ^{1}H-^{13}C HMBC NMR Spectrum of 9y in $CDCl_3$ after isolation via column chromatography.

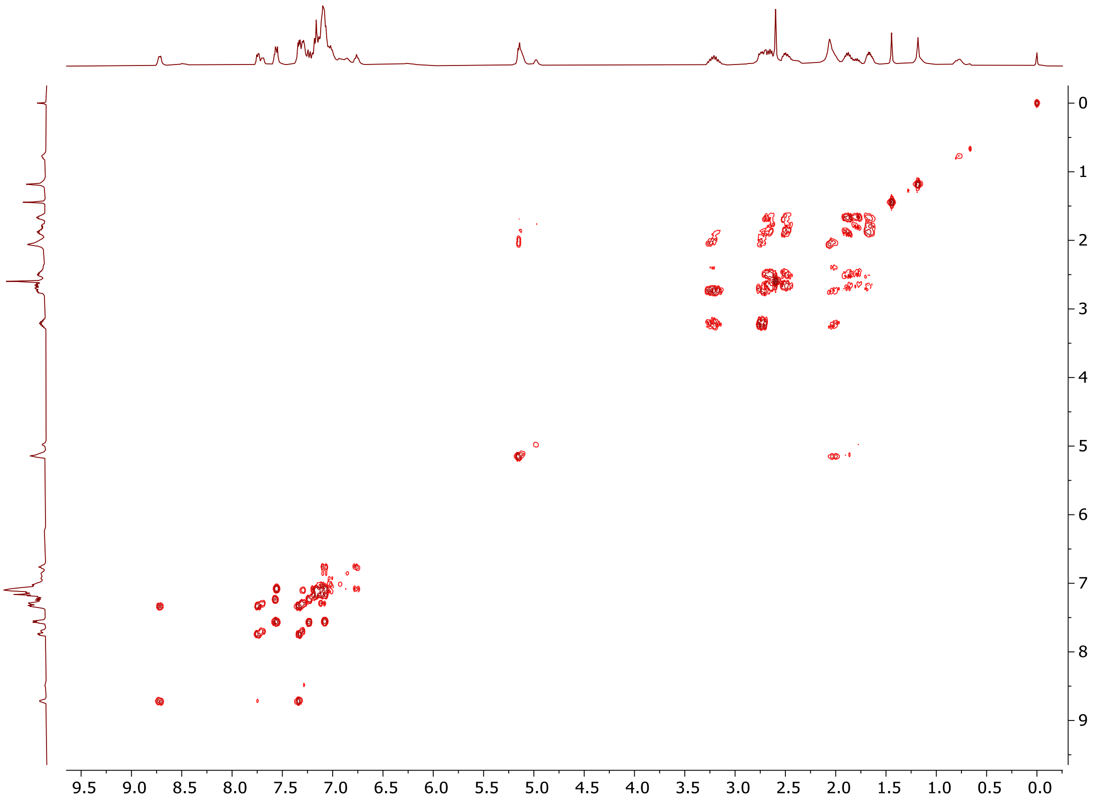

Figure S349: ¹H-¹H COSY NMR Spectrum of 9y in $CDCl_3$ after isolation via column chromatography.

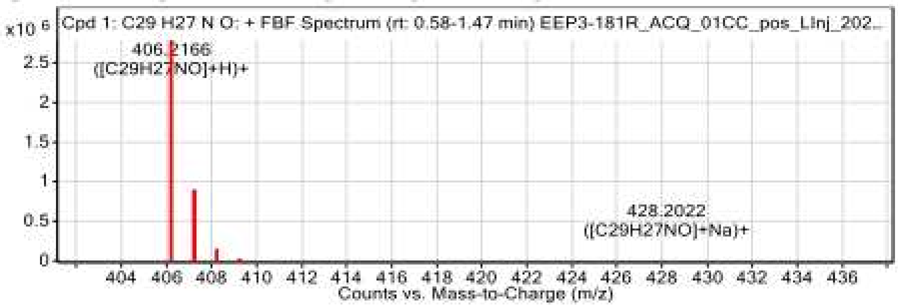

Figure S350: HRMS of compound 9y.

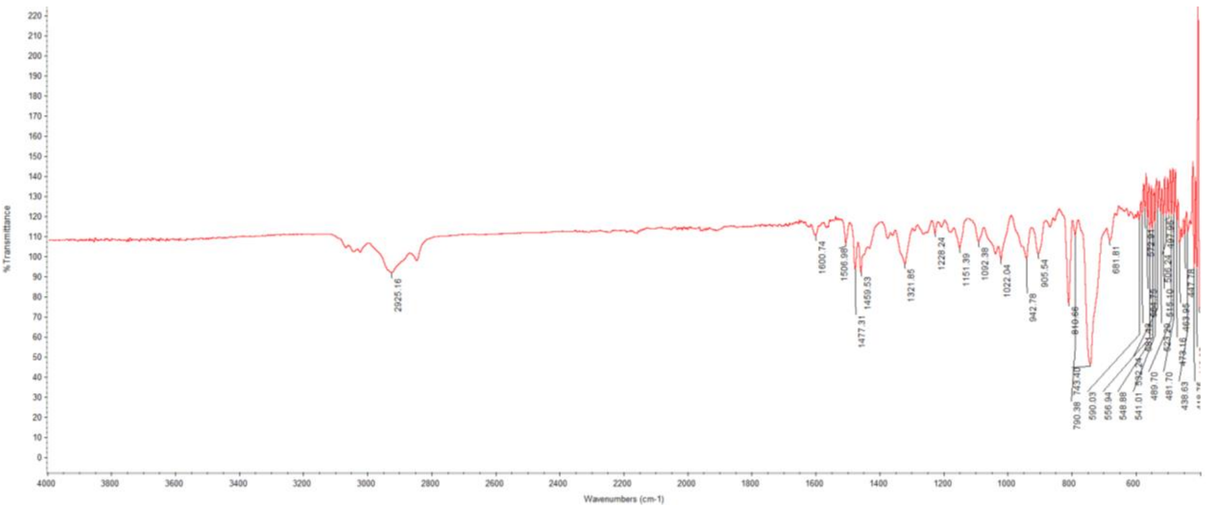

Figure S351: IR Spectra of compound 9y.

12. Electron Paramagnetic Resonance

Samples for EPR measurements were loaded into Young's EPR tubes under an N_2 atmosphere in a glovebox. The X-band CW EPR measurements (298 K) were performed on a Bruker EMX spectrometer utilizing an ER 072 magnet/ ER 081 power supply combination (maximum field 0.6 T), an ER4119HS resonator, operating at 100 kHz field modulation and 10 mW microwave power. Additional frozen solution EPR measurements (T = 120 K) were performed on a Bruker E500 spectrometer utilizing an ER 073 magnet/ ER 083 power supply combination (maximum field 1.45 T), an ER4102ST resonator, operating at 100 kHz field modulation, 0.5 mT modulation depth and 10 mW microwave power.

Simulations of all EPR spectra were performed using garlic or pepper functions within the Easyspin toolbox for Matlab.[20]

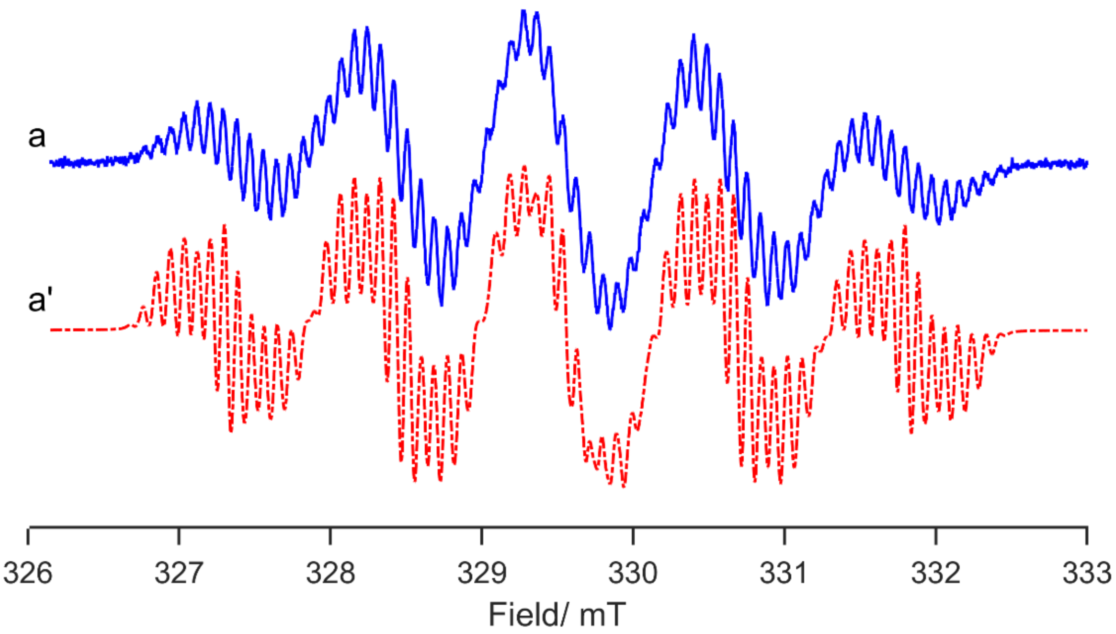

*Figure S352: CW X-band EPR spectrum (T = 298 K) of **1b** + N,N-dimethylnitrosoaniline; (a) Experimental, and (a') simulation.*

Spin Hamiltonian parameters for *N,N*-dimethylnitrosoaniline$^{+\bullet}$ [21-22]

g_{iso} = 2.0595

$a_{iso}(^{14}N)_1$ = 32.03; $a_{iso}(^{14}N)_1$ = 32.77; $a_{iso}(^{1}H)_2$ = 3.33; $a_{iso}(^{1}H)_6$ = 2.42; $a_{iso}(^{1}H)_2$ = 7.33 MHz *[subscript indicates number of equivalent nuclei]*.

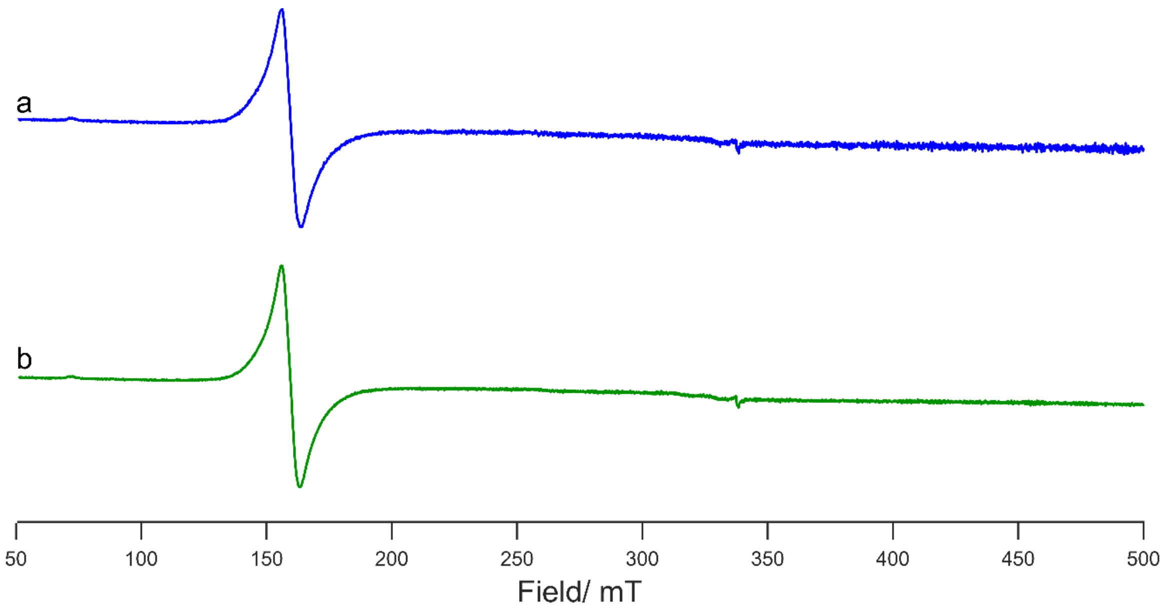

*Figure S353: **1a** in the presence of HBPin, and (a)* N,N-*dimethyl-4-nitrosoaniline (**4c**), or (b)* N,N-*dimethyl-4-nitroaniline. T = 120 K.*

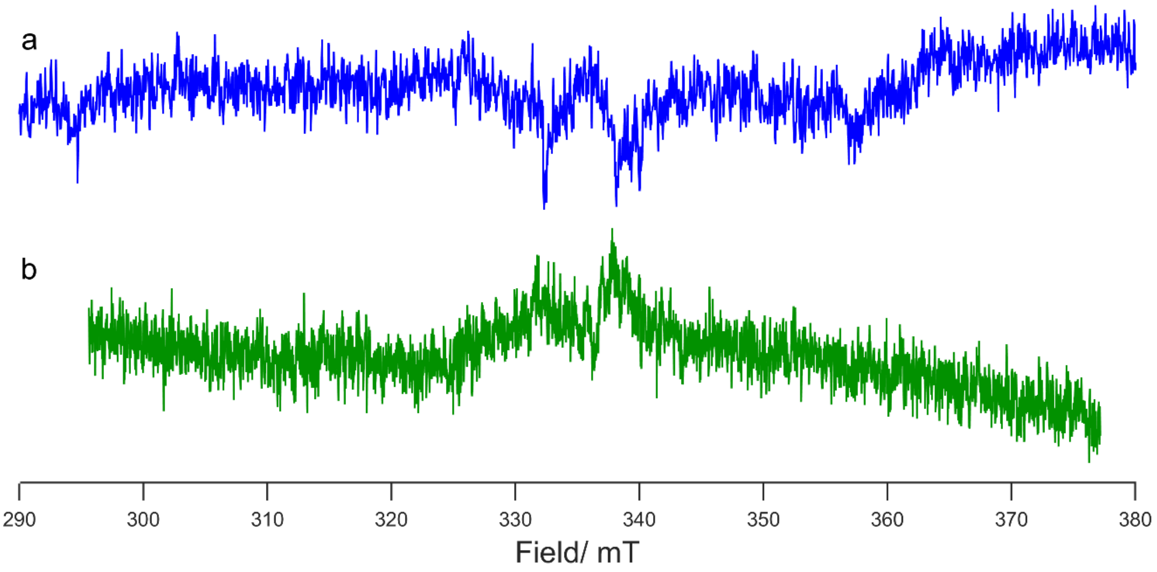

*Figure S354: **1a** in the presence of HBPin, and (a)* N,N-*dimethyl-4-nitrosoaniline (**4c**), or (b)* N,N-*dimethyl-4-nitroaniline. T = 298 K*

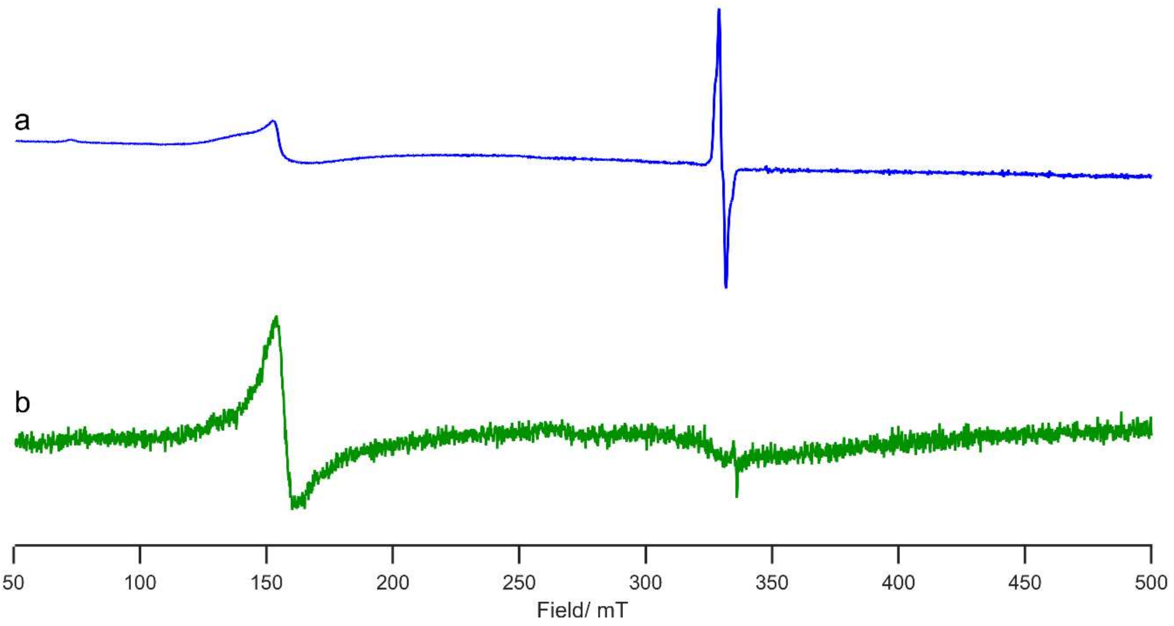

*Figure S355: **1b** in the presence of HBPin, and (a)* N,N*-dimethyl-4-nitrosoaniline (**4c**), or (b)* N,N*-dimethyl-4-nitroaniline. T = 120 K*

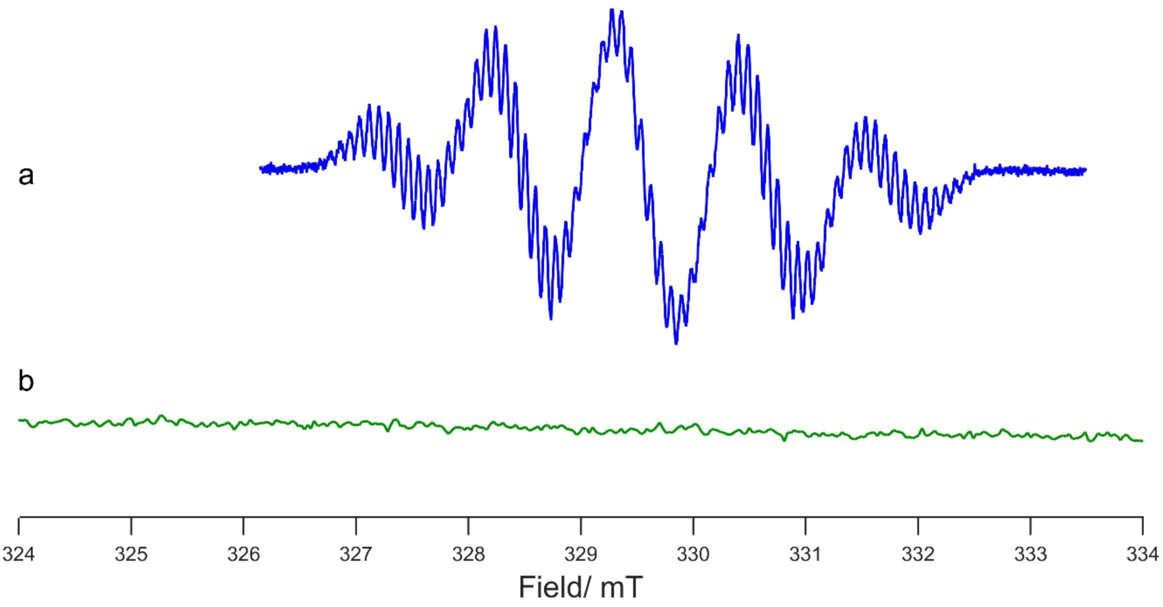

*Figure S356: **1b** in the presence of HBPin, and (a)* N,N*-dimethyl-4-nitrosoaniline (**4c**), or (b)* N,N*-dimethyl-4-nitroaniline. T = 298 K*

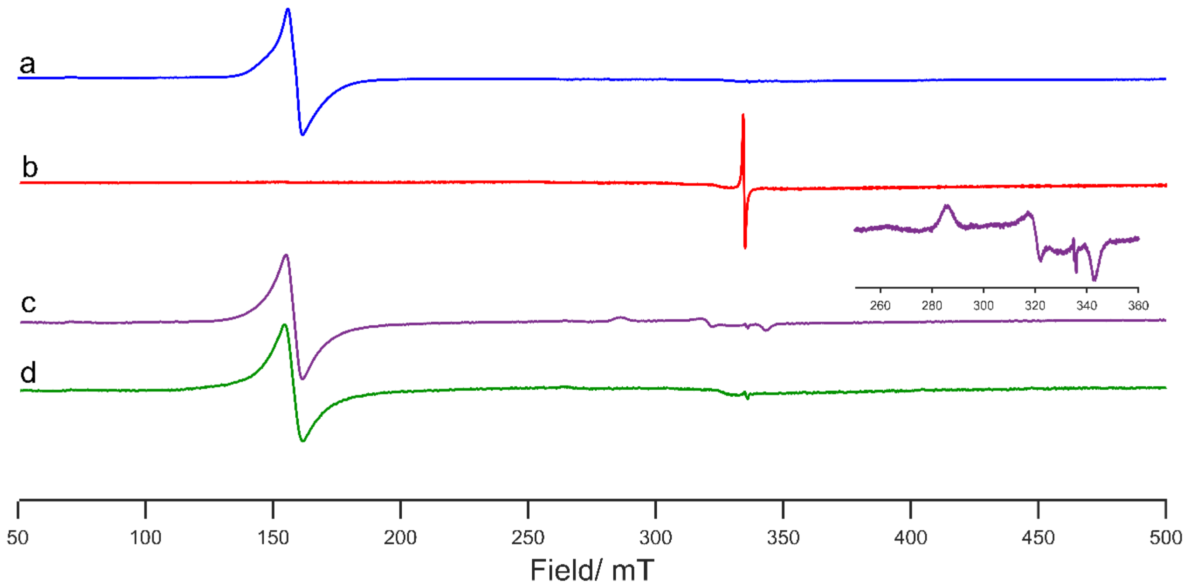

*Figure S357: (a) **1a**, (b) **1b*** (evidence for excess KC_8 is observed), *(c) **1a** with HBpin, and (d) **1a** with $PhSiH_3$ (heated to 50 °C for 1.5 hrs).*

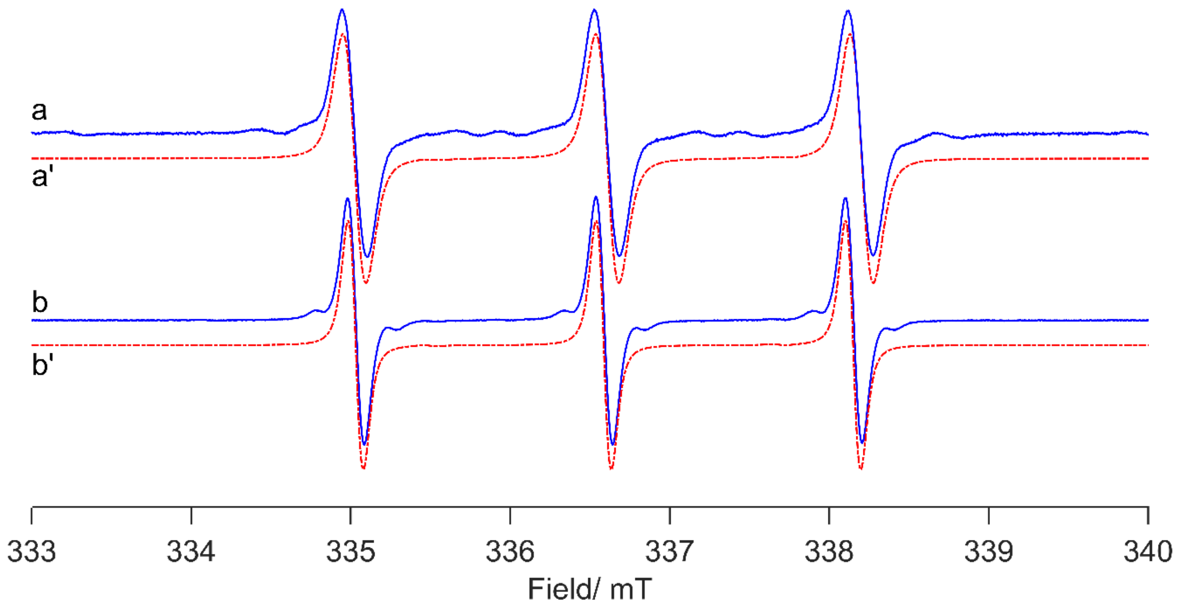

*Figure S358: CW X-band EPR spectrum (T = 298 K) of **1b** in the presence of (a) nitromethane, and (b) tert-nitrobutane. Corresponding simulations presented in (a', b').*

Spin Hamiltonian parameters for nitromethane: g_{iso} = 2.0164, $a_{iso}(^{14}N)_1$ = 44.87 MHz

Spin Hamiltonian parameters for *tert*-nitrobutane: g_{iso} = 2.0171, $a_{iso}(^{14}N)_1$ = 44.04 MHz

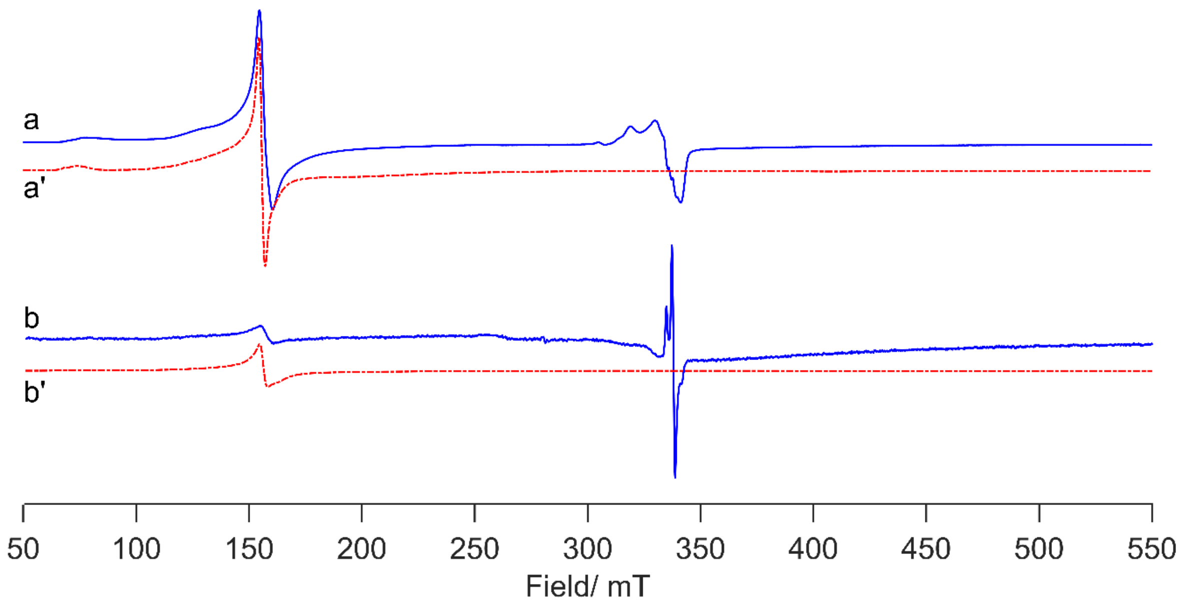

*Figure S359: CW X-band EPR spectrum (T = 120 K) of **1b** in the presence of (a) nitromethane, and (b) tert-nitrobutane. Corresponding simulations presented in (a', b').*

The spin Hamiltonian for a high-spin S = 5/2 system is given by:

$$\hat{H} = \mu g \hat{B}\hat{S} + D\left[\hat{S}_z^2 - \frac{1}{3}S(S+1)\right] + E[\hat{S}_x^2 - \hat{S}_y^2]$$

where D and E represent the axial and rhombic zero-field parameters, that dominate for high-spin systems. The g-value has less influence on the overall signal for high-spin species and is typically close to g_e. The intense signals observed at B ~ 160 mT (commonly identified in the literature by g_{eff} ~ 4-4.3) are characteristic of species with a strong (*i.e.,* D > hν, ~ 0.3 cm^{-1} or 9000 MHz at X-band frequency) and low symmetry crystal field (*i.e.,* E/D ≈ 1/3). The weaker low-field signals (B ~ 80 – 120 mT) are characteristic of S = 5/2 species with a strong crystal field with high symmetry (*i.e.,* E/D ≈ 0), and are typically labelled by g_{eff} = 9 – 6 [23-24]

Spin Hamiltonian parameters for **1b** + nitromethane:

S = 5/2, g_{iso} = 2.020, D = 9100 MHz, E = 1820 MHz (E/D = 0.2)

Spin Hamiltonian parameters for **1b** + *tert*-nitrobutane:

Species 1: **S** = 5/2, g_{iso} = 2.010, D = 8100 MHz, E = 1822 MHz (E/D = 0.225);

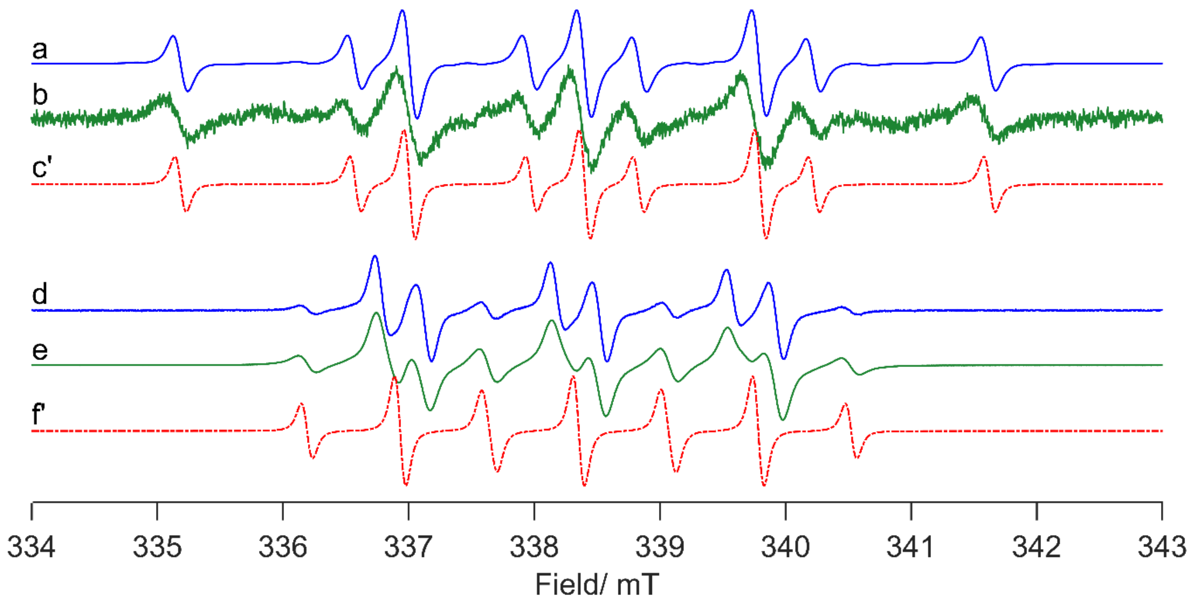

*Figure S360: CW X-band EPR spectrum (T = 298 K) of **1a** with (a, d) HBpin and (b, e) $PhSiH_3$ in the presence of (a, b) DMPO and (d, e) PBN spin traps. Corresponding simulations of (c') H-DMPO and (f') H-PBN adducts.*

Spin Hamiltonian parameters for **H-DMPO**[23]: g_{iso} = 2.0057; $a_{iso}(^{14}N)$ = 39.20, a_{iso} $(^{1}H)_2$ = 51.20 MHz;

Spin Hamiltonian parameters for **H-PBN**[23]: g_{iso} = 2.0060; $a_{iso}(^{14}N)$ = 40.00, a_{iso} $(^{1}H)_2$ = 20.78 MHz

[subscript indicates the number of equivalent nuclei]

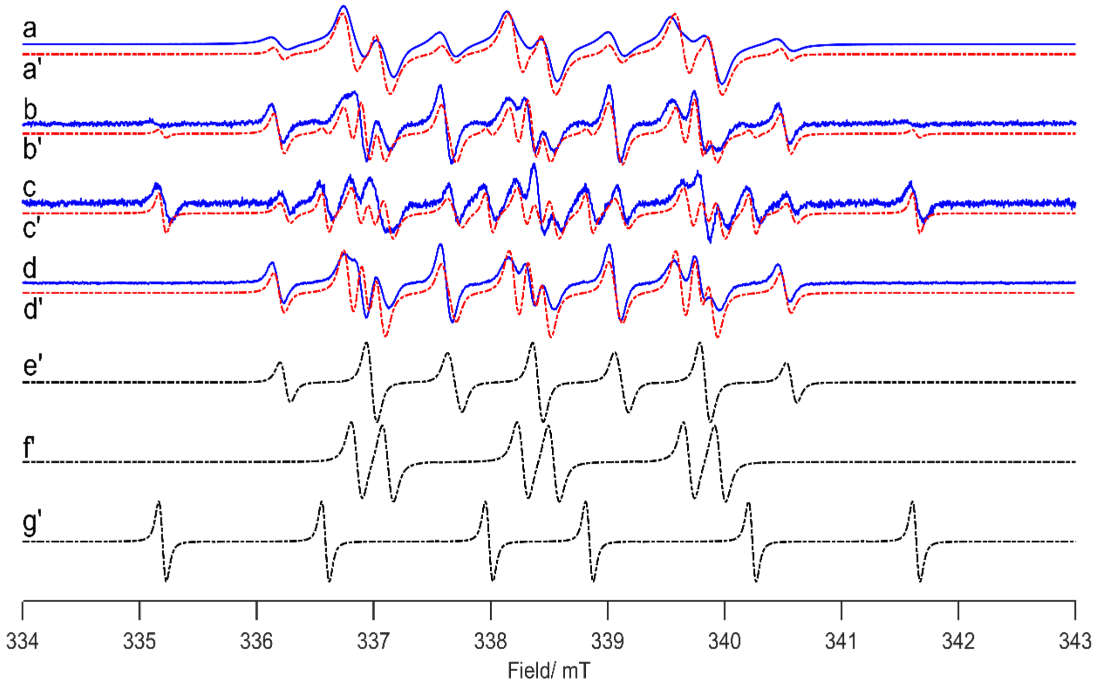

*Figure S361: CW X-band EPR spectrum (T = 298 K) of **1a** (1 mol%) and (a) $PhSiH_3$ (3 equivalents), and $PhSiH_3$(2): tBuNO$_2$-benzene(1) with (b) trans-stilbene(3), (c) indene(3), (d) hexene(3) in the presence of PBN spin trap. Numbers in brackets indicate number of equivalents. Corresponding simulations of reaction aliquots shown in a′ - d′; simulations of (e′) H-PBN, and (f′) $PhSiH_2$-PBN and (g′) amine-PBN adducts.*

Spin Hamiltonian parameters for **H-PBN**[25]: g_{iso} = 2.0060; $a_{iso}(^{14}N)$ = 40.00, a_{iso} $(^{1}H)_2$ = 20.78 MHz;

Spin Hamiltonian parameters for **$PhSiH_2$-PBN**[26]: g_{iso} = 2.0061; $a_{iso}(^{14}N)$ = 39.87, a_{iso} (^{1}H) = 7.44 MHz

Spin Hamiltonian parameters for **amine-PBN**[27]: g_{iso} = 2.0056; $a_{iso}(^{14}N)$ = 39.20, a_{iso} (^{1}H) = 102.46 MHz

*Table 5: Relative weightings of PBN-adducts for reaction of **1a** with $PhSiH_3$, tNO$_2$-benzene and alkene.*

**Sample**	**Relative Weighting**		
	**H-PBN**	**$PhSiH_2$-PBN**	**Amine-PBN**
**$PhSiH_3$**	0.3	1	0
**$PhSiH_3$: *t*BuNO$_2$-benzene: trans-stilbene**	1	0.6	0.1
**$PhSiH_3$: *t*BuNO$_2$-benzene: indene**	0.5	0.6	0.5
**$PhSiH_3$: *t*BuNO$_2$-benzene: hexene**	1	1	0

13 Quantum-Chemical Studies

13.1 Computational Methods

Quantum chemical calculations were carried out with the Gaussian 16[28] and ORCA 5[29,30] programs. With Gaussian 16, molecular geometries were optimzed at the density functional theory (DFT) level employing the generalized gradient approximation (GGA) via the PBE[31,32] functional in conjunction with the D3 atom-pairwise dispersion correction without damping[33] and an implicit polarizable continuum solvent model[34] utilizing acetonitrile as the solvent. The split-valence double-zeta def2-SVP[35] basis set was used together with the corresponding auxiliary Coulomb-fitting basis set of Weigend.[36] At this GGA DFT level, abbreviated as PBE-D3(PCM)/def2-SVP, frequency calculations were performed on the optimized stationary points to characterize minima and transition structures, and to extract thermal contributions to enthalpies and Gibbs energies at 298.15 K. For improved relative energies single-point energy calculations were performed at the hybrid DFT level with 25 % admixture of Fock-exchange via the PBE0[37,38] functional employing the triple-zeta valence polarized def2-TZVP basis set[35] and the same dispersion and solvent corrections as above. The final relative Gibbs energies and orbital eigenvalues are thus reported at the PBE0-D3(PCM)/def2-TZVP // PBE-D3(PCM)/def2-SVP level.

To ascertain relative energies for selected species, correlated *ab initio* calculations were performed with ORCA 5 utilizing the domain-based local pair natural orbital approximation for coupled-cluster calculations including single and double excitations and iterative perturbative triple excitations, DLPNO-CCSD(T1).[39,40] The one-particle space was described with the triple-zeta def2-TZVPP and quadruple-zeta def2-QZVPP basis sets[35] on all atoms. The corresponding auxiliary Coulomb[36] and correlation[41] fitting basis sets were used throughout together with the RIJCOSX[42] algorithm. In addition to Hartree–Fock reference wavefunctions for canonical HF-CC theory we also used Kohn–Sham orbitals from PBE0 single point calculations with the same def2-*n*ZVPP/RIJCOSX one-particle description for KS-CC theory. The latter are referred to as PBE0-DLPNO-CCSD(T1) in the following. The reference and correlation energies were extrapolated to the complete basis set (CBS) limit, CBS(T,Q),[43] according to

$$E_{\text{ref}}(\text{CBS}) = E_{\text{ref}}(Q) + \frac{E_{\text{ref}}(Q) - E_{\text{ref}}(T)}{\exp(\alpha\,(\sqrt{4} - \sqrt{3})) - 1}$$

$$E_{\text{corr}}(\text{CBS}) = \frac{3^{\beta}\,E_{\text{corr}}(T) - 4^{\beta}\,E_{\text{corr}}(Q)}{3^{\beta} - 4^{\beta}}$$

with cardinal numbers 3 for T and 4 for Q, and $\alpha = 7.88$, $\beta = 2.97$.

The `NormalPNO` settings in ORCA convention were employed with pair natural orbital (PNO) thresholds set to `TCutPNO` = 10^{-6} or 10^{-7}, in order to arrive at an extrapolated PNO limit, PNO(6,7),[44] for the correlation energies according to

$$E_{\text{corr}}(\text{PNO(6,7)}) = E_{\text{corr}}(\text{PNO6}) + F_{\text{PNO}} \times (E_{\text{corr}}(\text{PNO7}) - E_{\text{corr}}(\text{PNO6}))$$

where $F_{\text{PNO}} = 1.5$ for a set of `TCutPNO` thresholds differing by one order of magnitude.

13.2 Catalytic cycles for reduction of ^{i}PrNO$_2$

Figure S362 shows the interlinked catalytic cycles for reduction of ^{i}PrNO$_2$ with iron(**III**) hydride intermediate **1c**. The catalytic sequence is computed for the high-spin surface throughout for two reasons: (i) all iron-containing intermediates are high-spin ground state species at the applied level of DFT; (ii) potential intermediate-spin or low-spin transition structures would always have to undergo a spin-orbit coupled spin-crossover twice between the corresponding reactant and product intermediate structures. An estimate of such spin-crossing probabilities is, however, beyond the scope of this work, and we thus presume that the high-spin surface will be more competitive during the catalytic cycle than a multistate scenario with several spin crossover impediments.

The first half-cycle for reduction of ^{i}PrNO$_2$ to ^{i}PrNO and regeneration of the iron(**III**) hydride intermediate **1c** is exergonic by $-59.6\,\mathrm{kcal\,mol^{-1}}$, see Table S6. In the second half-cycle the short-lived nitroso intermediate ^{i}PrNO is further reduced. The formal thermochemistry to yield the amine product ^{i}PrNH$_2$ releases another $-117.7\,\mathrm{kcal\,mol^{-1}}$. The overall thermochemical balance for complete reduction thus amounts to $-177.3\,\mathrm{kcal\,mol^{-1}}$. Formation of product **2** in the second half-cycle is more or less identical in thermochemical terms.

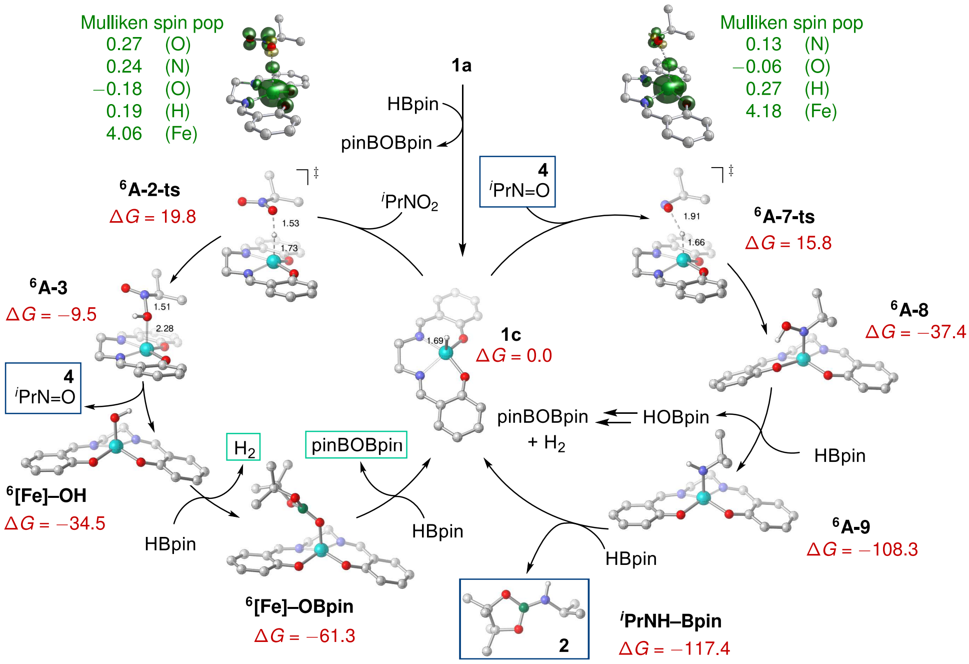

Figure S362: Select stationary points on the interlinked catalytic cycles for the high-spin (HS) surface commencing with iron(III) hydride intermediate **1c** and ^{i}PrNO$_2$. Spin densities at 0.01 a_0^{-3} (α-spin green, β-spin yellow) are shown for the two initial transition structures **^{6}A-2-ts** and **^{6}A-7-ts** along with most significant Mulliken spin populations. Relative Gibbs energies are given in kcal mol^{-1}.

Table S6: Net thermochemistry in kcal mol^{-1} for the two half-cycles and the overall reduction of ^{i}PrNO$_2$ to ^{i}PrNH$_2$ with HBpin.

Net reaction			$\Delta_r H$	$\Delta_r G$
^{i}PrNO$_2$	$\xrightarrow[\text{H}_2 + \text{O(Bpin)}_2]{\text{2 HBpin}}$	^{i}PrNO	−61.9	−59.6
^{i}PrNO	$\xrightarrow[\text{O(Bpin)}_2]{\text{2 HBpin}}$	^{i}PrNH$_2$	−129.5	−117.7
^{i}PrNO$_2$	$\xrightarrow[\text{H}_2 + 2\,\text{O(Bpin)}_2]{\text{4 HBpin}}$	^{i}PrNH$_2$	−191.4	−177.3

The rate-limiting activation step for reduction commences with hydride transfer from **1c** to the nitro substrate. In the lowest-energy path **A** shown in Figure S363 the substrate approaches the iron(III) hydride **1c** towards an adduct complex **^{6}A-1**, which is a minimum structure with respect to ΔH only. After passing the transition structure **^{6}A-2-ts**, the Fe–OH contact is already formed via the shallow minimum structure **^{6}A-3**. The weaker N–O bond is readily cleaved with a small barrier of only 2.5 kcal mol^{-1}, leading to the nitroso–[Fe]OH adduct **^{6}A-5** followed by release of the short-lived nitroso-intermediate **4**. We note in passing that reduction of the salen ligand may occur via a side reaction branching off from **^{6}A-3** (green trace in Figure S363). This is interesting to acknowledge with respect to ligand reduction observed for alkyne cyclotrimerisation via an iron(salan)-boryl on-cycle intermediate.[7] Path **B** (see Figure S364) is close in orientation and energy to path **A**. The equivalent of the shallow minimum structure **^{6}A-3**, however, is rushed by to directly liberate **4**. Path **C** features an adduct complex **^{6}C-1** with an Fe–O contact already formed. The subsequent TS **^{6}C-2-ts** for 1,2-hydrogen shift is energetically disfavoured. Table S7 lists details on the individual species for these paths.

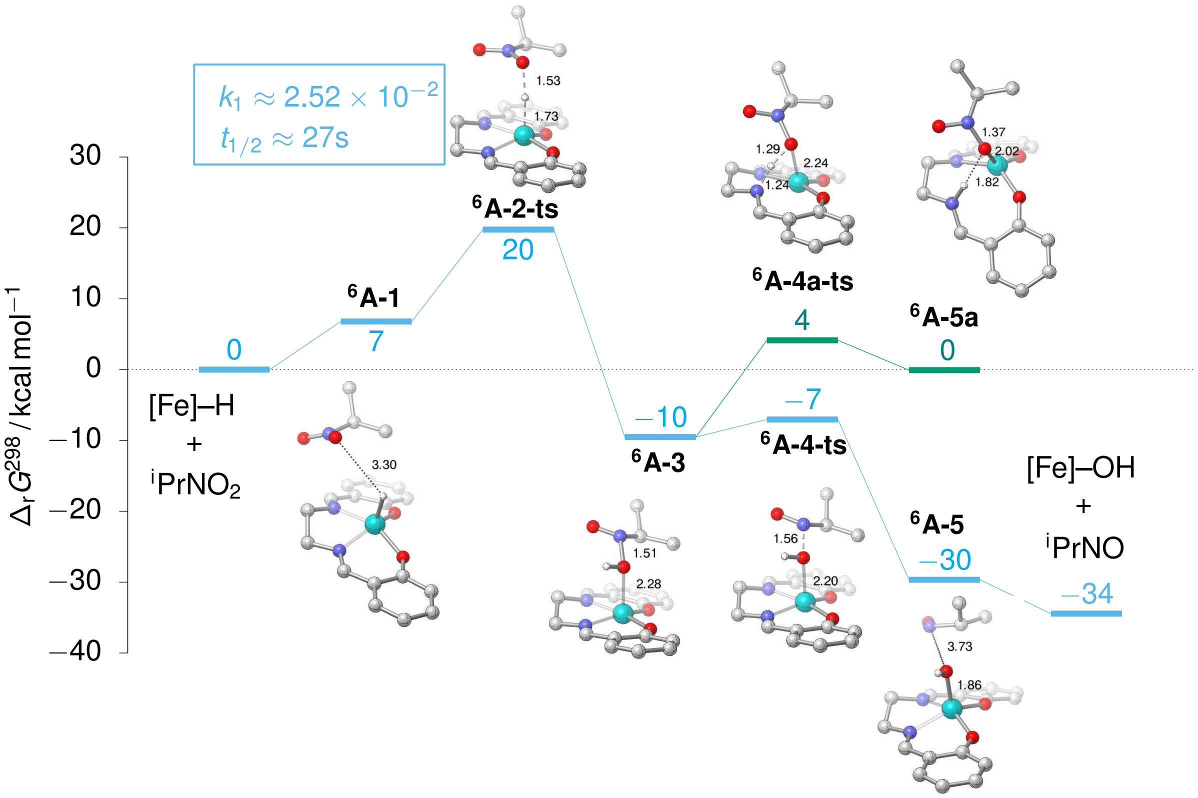

Figure S363: Initial reaction path of first half-cycle for high-spin (HS) iron(III) hydride intermediate **1c** and ^{i}PrNO$_2$. Blue trace corresponds to path A, green trace correponds to side path involving ligand reduction.

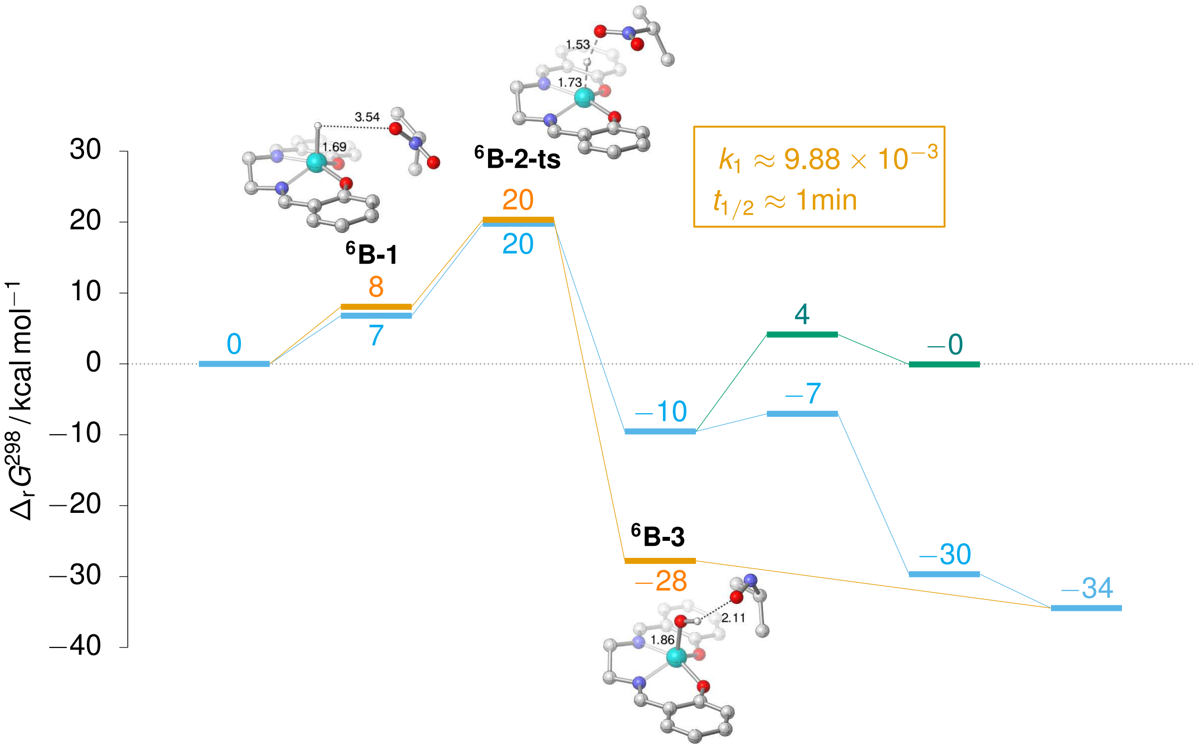

Figure S364: Initial reaction path of first half-cycle for high-spin (HS) iron(III) hydride intermediate **1c** and ^{i}PrNO$_2$. Yellow trace corresponds to path B.

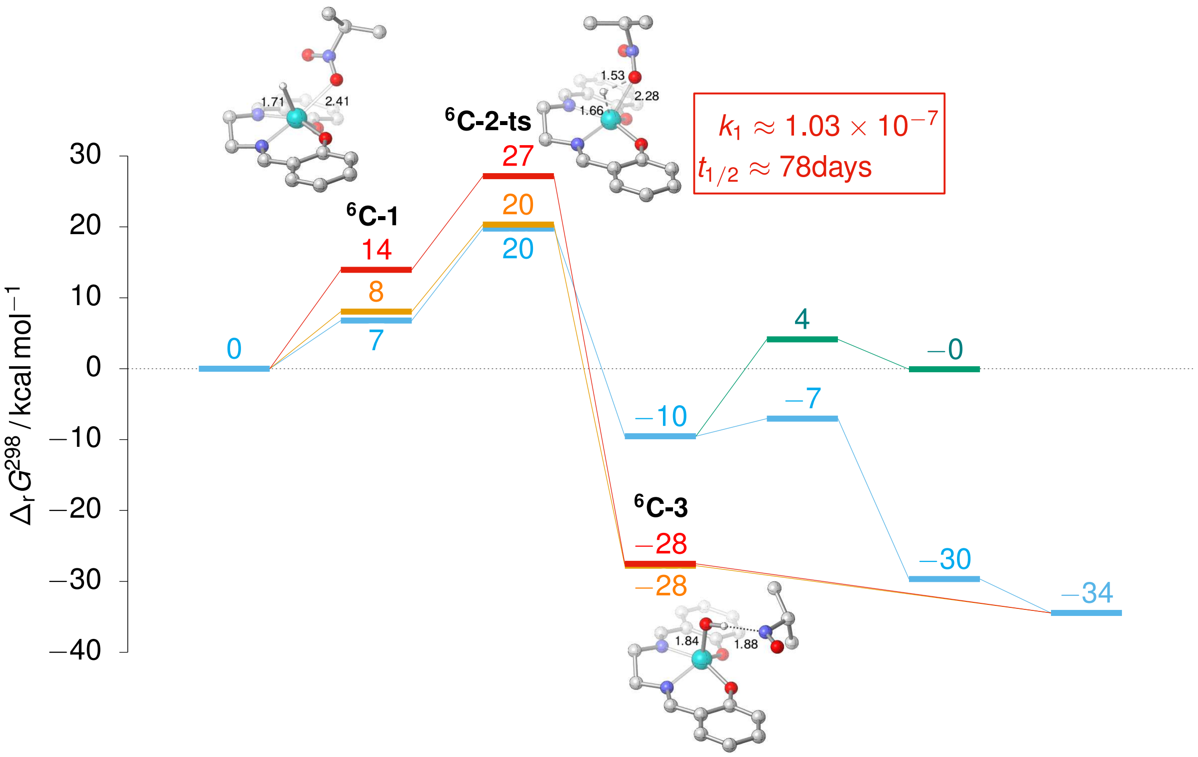

Figure S365: Initial reaction path of first half-cycle for high-spin (HS) iron(III) hydride intermediate **1c** and ^{i}PrNO$_2$. Red trace corresponds to path C.

Table S7: Total energies (Hartree) for individual species on the high-spin surface and for selected species on the intermediate-spin and low-spin surfaces, pertaining to the interlinked catalytic cycles for reduction of ^{i}PrNO$_2$ as sketched in Figure S362. Additional species corresponding to Figures S363–S365 are also included. For transition structures the imaginary frequency is stated in cm^{-1}. Spin expectation values, DFT-corrections to enthalpies and to Gibbs energies are also shown. Relative energies (kcal mol^{-1}) are given for comparison and correspond to the respective reaction sequence within the catalytic cycle.

PBE0-D3(PCM)/def2-TZVP // PBE-D3(PCM)/def2-SVP/W06								
Species[a]	$\langle S^2 \rangle$	E_{tot}	H_{tot}^{298}	G_{tot}^{298}	ν_{imag}	E_{rel}	H_{rel}	G_{rel}
**^{i}PrNO$_2$**	0.000	−323.426 533	0.111 161	0.072 382				
**^{6}[Fe]–H**	8.762	−2141.572 241	0.287 305	0.218 769		0.0	0.0	0.0
**^{6}A-1**	8.762	−2465.007 789	0.400 621	0.310 991		−5.7	−4.3	6.8
**^{6}A-2-ts**	8.852	−2464.986 279	0.397 804	0.310 139	465 i	7.8	7.4	19.8
**^{6}A-3**	8.790	−2465.038 633	0.403 508	0.315 817		−25.0	−21.9	−9.5
**^{6}A-4-ts**	8.790	−2465.034 613	0.402 236	0.315 769	78 i	−22.5	−20.1	−7.0
**^{6}A-5**	8.762	−2465.064 282	0.401 976	0.309 383		−41.1	−38.9	−29.7
**^{6}A-4a-ts**	8.781	−2465.016 733	0.399 591	0.315 694	766 i	−11.3	−10.6	4.1
**^{6}A-5a**	8.782	−2465.026 211	0.404 809	0.318 479		−17.2	−13.2	−0.1
**^{6}B-1**	8.762	−2465.006 670	0.400 867	0.311 898		−5.0	−3.5	8.1
**^{6}B-2-ts**	8.848	−2464.985 856	0.397 806	0.310 610	505 i	8.1	7.7	20.3
**^{6}B-3**	8.766	−2465.062 663	0.402 307	0.310 762		−40.1	−37.7	−27.8
**^{6}C-1**	8.763	−2464.996 266	0.400 362	0.310 852		1.6	2.8	13.9
**^{6}C-2-ts**	8.905	−2464.974 950	0.398 003	0.310 605	672 i	15.0	14.7	27.2
**^{6}C-3**	8.762	−2465.063 832	0.402 637	0.312 357		−40.8	−38.2	−27.5
**^{4}A-1**	3.879	−2464.998 239	0.401 625	0.313 925		0.3	2.3	14.6
**^{4}A-2-ts**	3.876	−2464.971 322	0.398 819	0.314 052	367 i	17.2	17.5	31.6
**^{4}A-3**	3.796	−2465.029 063	0.405 071	0.319 820		−19.0	−14.9	−1.0
**^{2}A-1**	0.797	−2465.008 683	0.403 510	0.318 173		−6.2	−3.1	10.7
**^{2}A-2-ts**	1.708	−2464.998 210	0.398 832	0.313 039	816 i	0.4	0.6	14.1
**^{2}A-3**	1.770	−2465.029 004	0.405 138	0.321 543		−19.0	−14.8	0.1
**^{6}[Fe]–OH**	8.762	−2216.812 753	0.296 017	0.224 632		−35.1	−34.1	−34.5
**HBpin**	0.000	−411.550 486	0.194 908	0.151 670				
**H$_2$**	0.000	−1.168 056	0.013 045	−0.001 811				
**^{6}[Fe]–OBpin**	8.761	−2627.240 465	0.473 707	0.380 552		−63.5	−65.1	−61.3
**pinBOBpin**	0.000	−897.211 129	0.378 847	0.308 610		−58.7	−61.9	−59.6
**^{i}PrNO**	0.000	−248.241 932	0.104 025	0.067 536		0.0	0.0	0.0
**^{6}A-6**	8.763	−2389.821 541	0.393 605	0.304 861		−4.6	−3.2	7.0
**^{6}A-7-ts**	8.797	−2389.809 346	0.392 085	0.306 649	84 i	3.0	3.5	15.8
**^{6}A-8**	8.791	−2389.902 731	0.399 305	0.315 281		−55.6	−50.6	−37.4
**^{6}A-9**	8.776	−2314.766 405	0.393 998	0.311 686		−126.6	−120.5	−108.3
**^{4}A-8**	3.820	−2389.880 700	0.400 045	0.318 871		−41.8	−36.3	−21.3
**^{2}A-6**	0.801	−2389.823 314	0.396 155	0.312 294		−5.7	−2.7	10.6
**^{2}A-7-ts**	1.484	−2389.808 732	0.392 897	0.310 899	429 i	3.4	4.4	18.9
**^{2}A-8**	0.939	−2389.875 239	0.400 862	0.323 490		−38.3	−32.3	−15.0
**Bpin-NH^{i}iPr**	0.000	−584.755 979	0.300 070	0.241 444		−133.7	−128.5	−117.4
**HOBpin**	0.000	−486.799 930	0.201 927	0.155 416				
**^{6}[Fe]–H**	8.762	−2141.572 241	0.287 305	0.218 769		27.6	25.0	18.7[b]
**H**	0.750	−0.501 056	0.002 360	−0.010 654				
**^{5}[Fe]**	6.033	−2141.027 249	0.280 768	0.215 265				

[a]Spin state denoted by superscript: ^{6} – sextet (UKS high-spin); ^{4} – quartet (UKS intermediate-spin); ^{2} – doublet (UKS low-spin).
[b]Homolytic bond dissociation energy for HS(**1c**) towards HS(**1b**) and atomic hydrogen.

13.2.1 Spin state of initial TS A-2-ts for reduction of $^{i}PrNO_2$

According to the DFT level applied there is a low-spin (LS) transition structure **^{2}A-2-ts** for the initial hydride transfer which is lower in energy than the high-spin (HS) electromer **^{6}A-2-ts** (Figure S366). Moreover, **^{2}A-2-ts** falls below the TS for hydride transfer of the second half cycle, which would render the latter as the rate-determining step. This LS species, however, suffers from significant spin contamination. Further exploration of the energetic landscape by means of DLPNO-CCSD(T1) calculations reveals t_1 amplitudes exceeding values of 0.9, which results in a large $\mathcal{T}_1$ diagnostic of 0.08. The singles amplitudes arise from single excitations between almost degenerate orbitals related to MOs delocalised between iron and the NO moiety of the substrate (Figure S367), which mirror the spin density distribution depicted in the right panel of Figure S366. This scenario has been discussed recently in a detailed assessment for a related case involving a singlet biradicaloid intermediate en route to Pd(II) nitrene reactivity.[45] For such species, HF-based coupled-cluster theory has been found to suffer dramatic errors exceeding 200 kcal mol^{-1}, while Kohn-Sham (KS) orbital references substantially improved CCSD(T) results. In that study, the t_1 amplitudes of the critical intermediate were reduced from more than 1.0 in HF-CC theory to less than 0.2 upon use of (more delocalised) KS reference orbitals. Concomitantly, a $\mathcal{T}_1$ diagnostic of as large as 0.24 in HF-CC was lowered to rather uncritical values of less than 0.04 in KS-CC calculations. We observe a similar trend for LS transition structure **^{2}A-2-ts**. KS reference orbitals lower the largest t_1 amplitudes significantly to values of 0.4, thereby reducing the $\mathcal{T}_1$ diagnostic to much more reasonable values below 0.04.

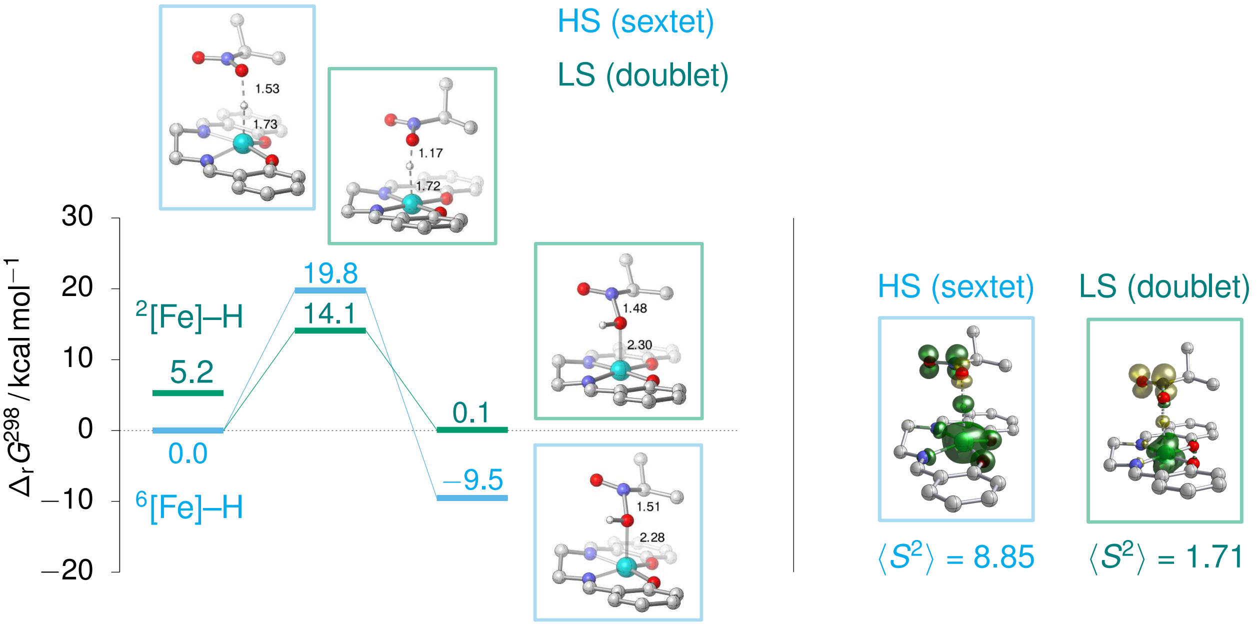

Figure S366: Initial TS for high-spin (HS) and low-spin (LS) PES starting from high-spin iron(III) hydride intermediate **1c** and $^{i}PrNO_2$. Left panel shows relative Gibbs energies and molecular geometries, right panel shows spin density isosurfaces at 0.01 a_0^{-3} (α-spin green, β-spin yellow) for the HS and LS hydride transfer TS.

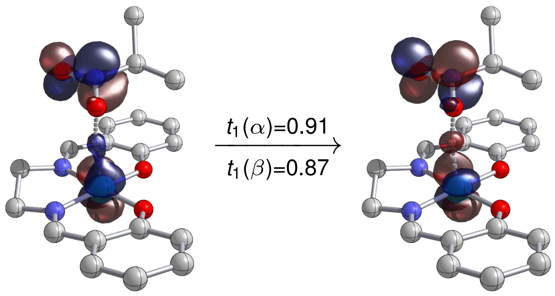

Figure S367: Orbitals associated with the largest PNO t_1 amplitudes in the low-spin transition structure **^{2}A-2-ts**, extracted from DLPNO-CCSD/def2-QZVPP calculations.

While the large t_1 amplitudes and $\mathcal{T}_1$ diagnostic indicate that HF-CC theory is not adequate for a description of the electronic structure of this low-spin TS **^{2}A-2-ts**, a KS-based ansatz appears more suitable. According to PBE0-DLPNO-CCSD(T1) calculations, the LS electromer **^{2}A-2-ts** is still lower in energy than its HS congener. More importantly though, regardless of spin state, the initial barrier associated with nitro reduction, **A-2-ts**, is always larger than the hydride transfer TS for nitroso reduction, **A-7-ts** (Figure S368). Note that at this level of theory the on-site iron hydride species **1c** is also a low-spin ground state species, favoured by $-3.6\,\mathrm{kcal\,mol^{-1}}$ over the HS electromer. The reaction would accordingly be initialised in the LS state and proceed to the HS intermediate ^{6}[Fe]–OH, which is $22.3\,\mathrm{kcal\,mol^{-1}}$ more stable than the LS equivalent. The rate-determining barrier is therefore associated with hydride transfer to ^{i}PrNO$_2$ in the first half-cycle, and it ranges between $18.7\,\mathrm{kcal\,mol^{-1}}$ on the LS surface and $21.1\,\mathrm{kcal\,mol^{-1}}$ on the HS surface. Table S8 lists a comparison of relative energies for the corresponding net barriers and thermochemistry. Details on spin expectation and $\mathcal{T}_1$ diagnostics are collected in Table S9.

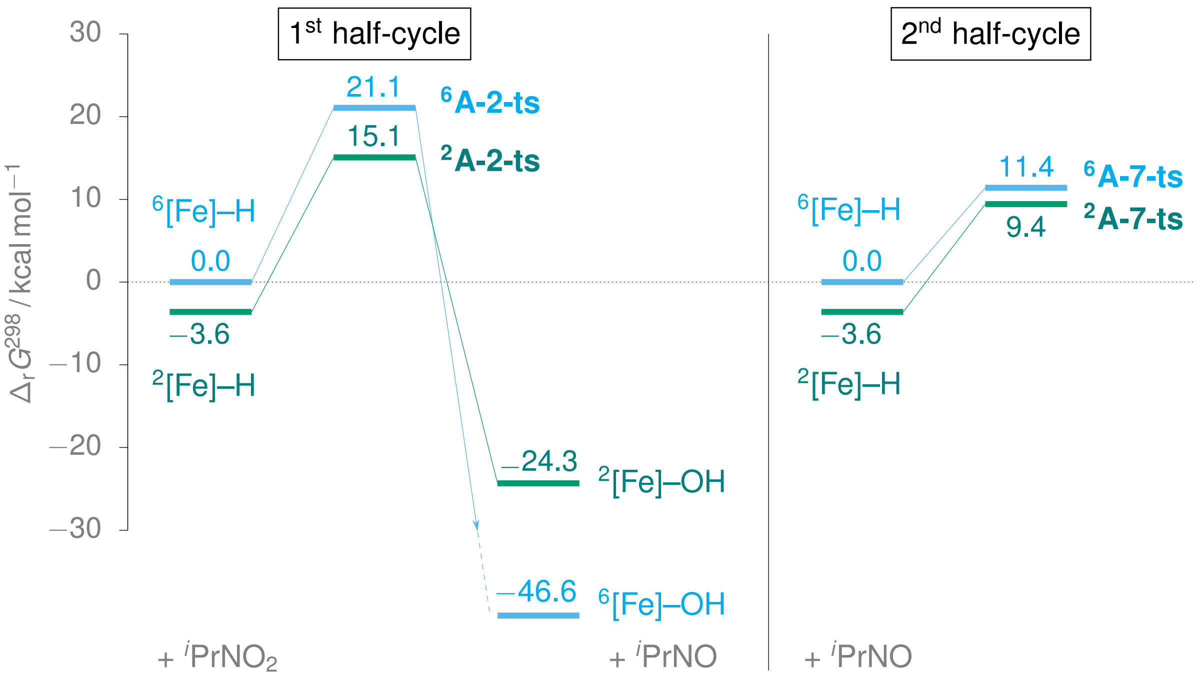

Figure S368: Initial TS for first and second half-cycles at high-spin (HS) and low-spin (LS) PES starting from high-spin iron(III) hydride intermediate **1c** and ^{i}PrNO$_2$ (first half-cycle) and from **1c** and ^{i}PrNO (second half-cycle). Relative Gibbs energies are computed at Kohn-Sham based coupled-cluster theory using PBE0-DLPNO-CCSD(T1)/CBS(T,Q)/PNO(6,7) energies and thermal corrections from PBE-D3(PCM)/def2-SVP calculations.

Table S8: Barriers and reaction energies for hydride transfer in $\mathrm{kcal\,mol^{-1}}$ for the high-spin and low-spin paths.

Net barrier/reaction sequence			$\Delta G^\ddagger$		
			DFT[a]	CCSD(T)[b]	KS-CCSD(T)[c]
^{6}[Fe]–H + ^{i}PrNO$_2$	⟶	**^{6}A-2-ts**	19.8	23.1	21.1
^{6}[Fe]–H + ^{i}PrNO	⟶	**^{6}A-7-ts**	15.8	13.9	11.4
^{6}[Fe]–H + ^{i}PrNO$_2$	⟶	**^{2}A-2-ts**	14.1	30.9	15.1
^{6}[Fe]–H + ^{i}PrNO	⟶	**^{2}A-7-ts**	18.9	23.1	9.4
^{2}[Fe]–H + ^{i}PrNO$_2$	⟶	**^{2}A-2-ts**	8.9	25.7	18.7
^{2}[Fe]–H + ^{i}PrNO	⟶	**^{2}A-7-ts**	13.7	17.8	13.0
^{6}[Fe]–H + ^{i}PrNO$_2$	⟶	^{6}[Fe]–OH + ^{i}PrNO	−34.5	−44.5	−46.6
^{2}[Fe]–H + ^{i}PrNO$_2$	⟶	^{2}[Fe]–OH + ^{i}PrNO	−15.7	−19.3	−20.7

[a]PBE0-D3(PCM)/def2-TZVP
[b]UHF-DLPNO-CCSD(T1)/def2(T,Q)ZVPP/PNO(6,7)
[c]PBE0-DLPNO-CCSD(T1)/def2(T,Q)ZVPP/PNO(6,7)

Table S9: Relative Gibbs energies (kcal mol^{-1}) for spin states of iron(II) salen complex **1b**, iron(III) hydride complex **1c**, iron(III) hydroxide complex [Fe]–OH, the initial TS for hydride transfer to *i*PrNO$_2$ **A-2-ts**, and the TS for second hydride transfer to *i*PrNO **A-7-ts**, computed at RI-PBE-D3(PCM)/def2-SVP/W06 geometries. Relative energies are compared among DFT, HF-CC, and KS-CC theory. Please note that for the latter KS-CC ansatz the choice of functional reference has little effect on the relative energies — see footnote [d] for a comparison of PBE0- and M06L-based orbital references.

Species	DFT[a]		CCSD(T)[b]		KS-CCSD(T)[c]	
Spin state	$\langle S^2 \rangle$	G_{rel}	$\mathcal{T}_1$	G_{rel}	$\mathcal{T}_1$	G_{rel}
[Fe] **1b**						
HS (quintet)	6.033	0.0	0.014	0.0	0.014	0.0
IS (triplet)	2.035	7.7	0.017	12.2	0.013	5.5
LS (singlet)	0.000	46.4	0.020	48.2	0.012	24.4
[Fe]–H **1c**[d]						
HS (sextet)	8.762	0.0	0.020	0.0	0.014	0.0
IS (quartet)	3.819	8.1	0.023	10.5	0.015	7.3
LS (doublet)	0.801	5.2	0.020	5.2	0.012	−3.6
[Fe]–OH						
HS (sextet)	8.762	0.0	0.018	0.0	0.015	0.0
IS (quartet)	3.796	13.3	0.022	23.8	0.013	19.4
LS (doublet)	0.957	23.9	0.024	30.4	0.012	22.3
initial hydride transfer TS **A-2-ts**						
HS (sextet)	8.852	0.0	0.021	0.0	0.017	0.0
IS (quartet)	3.876	11.8	0.020	18.2	0.018	−0.0
LS (doublet)	1.708	−5.7	0.076	7.8	0.037	−6.0
second hydride transfer TS **A-7-ts**						
HS (sextet)	8.797	0.0	0.019	0.0	0.016	0.0
LS (doublet)	1.484	3.1	0.028	9.2	0.017	−2.0

[a]PBE0-D3(PCM)/def2-TZVP
[b]UHF-DLPNO-CCSD(T1)/def2(T,Q)ZVPP/PNO(6,7)
[c]PBE0-DLPNO-CCSD(T1)/def2(T,Q)ZVPP/PNO(6,7)
[d]M06L-DLPNO-CCSD(T1)/def2(T,Q)ZVPP/PNO(6,7): ΔG_{rel}(HS/IS/LS) = (0.0 / 7.7 / −3.8) kcal mol^{-1}

Table S10: Total energies (Hartree) corresponding to Table S9 for spin states of iron(II) salen complex **1b**, iron(III) hydride complex **1c**, iron(III) hydroxide complex [Fe]–OH, as well as CC diagnostics for singles amplitudes. Values computed at the HF-DLPNO-CCSD(T1) level on RI-PBE-D3(PCM)/def2-SVP/W06 geometries.

			**HF-DLPNO-CCSD(T)**			
state	basis	thresh	E_{tot}(ref)	E_{corr}(CCSD)	E_{corr}(T1)	$\mathcal{T}_1$
			[Fe] **1b**			
HS (quintet)	def2-TZVPP	PNO6	−2135.517 305	−3.957 496	−0.187 849	0.013
	def2-TZVPP	PNO7	−2135.517 305	−3.963 293	−0.195 574	0.014
	def2-QZVPP	PNO6	−2135.583 168	−4.172 150	−0.203 795	0.013
	def2-QZVPP	PNO7	−2135.583 168	−4.174 211	−0.213 504	0.014
	CBS(T,Q)/PNO(6,7)		−2135.592 240	−4.330 094	−0.232 374	
IS (triplet)	def2-TZVPP	PNO6	−2135.438 293	−4.001 998	−0.192 904	0.015
	def2-TZVPP	PNO7	−2135.438 293	−4.010 685	−0.202 401	0.017
	def2-QZVPP	PNO6	−2135.497 744	−4.223 575	−0.210 171	0.015
	def2-QZVPP	PNO7	−2135.497 744	−4.228 349	−0.221 585	0.017
	CBS(T,Q)/PNO(6,7)		−2135.505 933	−4.390 520	−0.242 212	
LS (singlet)	def2-TZVPP	PNO6	−2135.350 157	−4.021 126	−0.196 398	0.017
	def2-TZVPP	PNO7	−2135.350 157	−4.035 021	−0.207 190	0.022
	def2-QZVPP	PNO6	−2135.411 923	−4.242 287	−0.213 102	0.016
	def2-QZVPP	PNO7	−2135.411 923	−4.252 213	−0.225 502	0.020
	CBS(T,Q)/PNO(6,7)		−2135.420 431	−4.416 590	−0.245 863	
			[Fe]–H **1c**			
HS (sextet)	def2-TZVPP	PNO6	−2136.059 841	−3.967 014	−0.190 917	0.018
	def2-TZVPP	PNO7	−2136.059 841	−3.972 527	−0.199 319	0.020
	def2-QZVPP	PNO6	−2136.123 419	−4.182 650	−0.206 553	0.018
	def2-QZVPP	PNO7	−2136.123 419	−4.184 540	−0.216 743	0.020
	CBS(T,Q)/PNO(6,7)		−2136.132 176	−4.341 190	−0.235 407	
IS (quartet)	def2-TZVPP	PNO6	−2135.913 674	−4.077 303	−0.202 208	0.022
	def2-TZVPP	PNO7	−2135.913 674	−4.085 709	−0.211 022	0.024
	def2-QZVPP	PNO6	−2135.975 809	−4.297 111	−0.218 353	0.021
	def2-QZVPP	PNO7	−2135.975 809	−4.301 333	−0.229 058	0.023
	CBS(T,Q)/PNO(6,7)		−2135.984 367	−4.461 615	−0.248 471	
LS (doublet)	def2-TZVPP	PNO6	−2135.893 328	−4.093 663	−0.207 510	0.018
	def2-TZVPP	PNO7	−2135.893 328	−4.105 509	−0.218 015	0.021
	def2-QZVPP	PNO6	−2135.954 995	−4.316 394	−0.224 184	0.017
	def2-QZVPP	PNO7	−2135.954 995	−4.324 010	−0.236 391	0.020
	CBS(T,Q)/PNO(6,7)		−2135.963 489	−4.488 104	−0.256 737	
			[Fe]–OH			
HS (sextet)	def2-TZVPP	PNO6	−2211.016 607	−4.194 459	−0.199 777	0.016
	def2-TZVPP	PNO7	−2211.016 607	−4.200 497	−0.208 607	0.018
	def2-QZVPP	PNO6	−2211.084 476	−4.428 832	−0.216 831	0.015
	def2-QZVPP	PNO7	−2211.084 476	−4.430 644	−0.227 543	0.018
	CBS(T,Q)/PNO(6,7)		−2211.093 824	−4.600 466	−0.247 622	
IS (quartet)	def2-TZVPP	PNO6	−2210.906 462	−4.250 488	−0.208 294	0.020
	def2-TZVPP	PNO7	−2210.906 462	−4.259 894	−0.218 380	0.022
	def2-QZVPP	PNO6	−2210.973 101	−4.486 695	−0.225 412	0.019
	def2-QZVPP	PNO7	−2210.973 101	−4.492 264	−0.237 459	0.022
	CBS(T,Q)/PNO(6,7)		−2210.982 280	−4.665 752	−0.258 341	
LS (doublet)	def2-TZVPP	PNO6	−2210.846 167	−4.287 829	−0.215 046	0.021
	def2-TZVPP	PNO7	−2210.846 167	−4.299 844	−0.225 896	0.025
	def2-QZVPP	PNO6	−2210.911 922	−4.526 673	−0.232 273	0.020
	def2-QZVPP	PNO7	−2210.911 922	−4.534 982	−0.245 194	0.024
	CBS(T,Q)/PNO(6,7)		−2210.920 979	−4.711 941	−0.266 718	

Table S11: Total energies (Hartree) corresponding to Table S9 for spin states of hydride transfer TS **A-2-ts** and **A-7-ts**, as well as for substrates *i*PrNO$_2$ and *i*PrNO, together with CC diagnostics for singles amplitudes. Values computed at the HF-DLPNO-CCSD(T1) level on RI-PBE-D3(PCM)/def2-SVP/W06 geometries.

			**HF-DLPNO-CCSD(T)**			
state	basis	thresh	E_{tot}(ref)	E_{corr}(CCSD)	E_{corr}(T1)	$\mathcal{T}_1$
			**A-2-ts**			
HS (sextet)	def2-TZVPP	PNO6	−2457.802 090	−5.300 275	−0.252 292	0.020
	def2-TZVPP	PNO7	−2457.802 090	−5.306 790	−0.262 677	0.021
	def2-QZVPP	PNO6	−2457.879 044	−5.589 657	−0.273 792	0.020
	def2-QZVPP	PNO7	−2457.879 044	−5.590 896	−0.286 362	0.021
	CBS(T,Q)/PNO(6,7)		−2457.889 644	−5.800 011	−0.311 000	
IS (quartet)	def2-TZVPP	PNO6	−2457.714 737	−5.340 371	−0.259 936	0.018
	def2-TZVPP	PNO7	−2457.714 737	−5.350 641	−0.271 768	0.020
	def2-QZVPP	PNO6	−2457.790 258	−5.633 817	−0.281 743	0.018
	def2-QZVPP	PNO7	−2457.790 258	−5.639 013	−0.295 841	0.020
	CBS(T,Q)/PNO(6,7)		−2457.800 660	−5.853 341	−0.321 561	
LS (doublet)	def2-TZVPP	PNO6	−2457.519 562	−5.543 459	−0.243 055	0.072
	def2-TZVPP	PNO7	−2457.519 562	−5.568 291	−0.254 652	0.076
	def2-QZVPP	PNO6	−2457.595 136	−5.836 204	−0.266 295	0.071
	def2-QZVPP	PNO7	−2457.595 136	−5.856 853	−0.279 896	0.076
	CBS(T,Q)/PNO(6,7)		−2457.605 545	−6.079 378	−0.306 140	
			**A-7-ts**			
HS (sextet)	def2-TZVPP	PNO6	−2383.009 249	−5.001 073	−0.236 113	0.017
	def2-TZVPP	PNO7	−2383.009 249	−5.007 475	−0.245 972	0.019
	def2-QZVPP	PNO6	−2383.083 322	−5.272 506	−0.256 072	0.017
	def2-QZVPP	PNO7	−2383.083 322	−5.274 174	−0.268 043	0.019
	CBS(T,Q)/PNO(6,7)		−2383.093 525	−5.470 809	−0.291 160	
LS (doublet)	def2-TZVPP	PNO6	−2382.781 993	−5.170 386	−0.259 878	0.024
	def2-TZVPP	PNO7	−2382.781 993	−5.185 858	−0.271 417	0.028
	def2-QZVPP	PNO6	−2382.853 875	−5.448 272	−0.280 797	0.024
	def2-QZVPP	PNO7	−2382.853 875	−5.458 006	−0.294 392	0.028
	CBS(T,Q)/PNO(6,7)		−2382.863 776	−5.662 339	−0.318 968	
			*i*PrNO$_2$			
singlet	def2-TZVPP	PNO6	−321.866 339	−1.233 370	−0.055 662	0.015
	def2-TZVPP	PNO7	−321.866 339	−1.234 472	−0.057 198	0.016
	def2-QZVPP	PNO6	−321.881 208	−1.304 977	−0.060 962	0.014
	def2-QZVPP	PNO7	−321.881 208	−1.305 408	−0.063 002	0.016
	CBS(T,Q)/PNO(6,7)		−321.883 256	−1.357 921	−0.068 508	
			*i*PrNO			
singlet	def2-TZVPP	PNO6	−246.997 951	−0.991 978	−0.042 065	0.013
	def2-TZVPP	PNO7	−246.997 951	−0.992 727	−0.043 078	0.013
	def2-QZVPP	PNO6	−247.009 391	−1.046 370	−0.046 135	0.013
	def2-QZVPP	PNO7	−247.009 391	−1.046 467	−0.047 433	0.013
	CBS(T,Q)/PNO(6,7)		−247.010 967	−1.086 082	−0.051 413	

Table S12: Total energies (Hartree) corresponding to Table S9 for spin states of iron(II) salen complex **1b**, iron(III) hydride complex **1c**, iron(III) hydroxide complex [Fe]–OH, as well as CC diagnostics for singles amplitudes. Values computed at the PBE0-DLPNO-CCSD(T1) level on RI-PBE-D3(PCM)/def2-SVP/W06 geometries.

PBE0-DLPNO-CCSD(T)						
state	basis	thresh	E_{tot}(ref)	E_{corr}(CCSD)	E_{corr}(T1)	$\mathcal{T}_1$
			[Fe] **1b**			
HS (quintet)	def2-TZVPP	PNO6	−2135.359 681	−4.105 466	−0.208 409	0.014
	def2-TZVPP	PNO7	−2135.359 681	−4.104 926	−0.216 636	0.014
	def2-QZVPP	PNO6	−2135.412 337	−4.323 167	−0.224 995	0.014
	def2-QZVPP	PNO7	−2135.412 337	−4.318 045	−0.234 839	0.014
	CBS(T,Q)/PNO(6,7)		−2135.419 590	−4.471 653	−0.253 842	
IS (triplet)	def2-TZVPP	PNO6	−2135.254 865	−4.177 022	−0.223 239	0.013
	def2-TZVPP	PNO7	−2135.254 865	−4.179 560	−0.232 937	0.013
	def2-QZVPP	PNO6	−2135.306 505	−4.399 166	−0.240 097	0.013
	def2-QZVPP	PNO7	−2135.306 505	−4.396 772	−0.251 438	0.013
	CBS(T,Q)/PNO(6,7)		−2135.313 617	−4.554 645	−0.271 423	
LS (singlet)	def2-TZVPP	PNO6	−2135.126 132	−4.243 648	−0.244 763	0.012
	def2-TZVPP	PNO7	−2135.126 132	−4.249 067	−0.256 322	0.011
	def2-QZVPP	PNO6	−2135.177 992	−4.468 444	−0.262 236	0.012
	def2-QZVPP	PNO7	−2135.177 992	−4.468 630	−0.275 390	0.012
	CBS(T,Q)/PNO(6,7)		−2135.185 135	−4.629 424	−0.296 682	
			[Fe]–H **1c**			
HS (sextet)	def2-TZVPP	PNO6	−2135.877 138	−4.142 940	−0.213 954	0.015
	def2-TZVPP	PNO7	−2135.877 138	−4.138 560	−0.222 500	0.014
	def2-QZVPP	PNO6	−2135.930 503	−4.362 624	−0.230 209	0.015
	def2-QZVPP	PNO7	−2135.930 503	−4.353 398	−0.240 398	0.014
	CBS(T,Q)/PNO(6,7)		−2135.937 853	−4.506 130	−0.259 357	
IS (quartet)	def2-TZVPP	PNO6	−2135.768 670	−4.214 291	−0.228 707	0.015
	def2-TZVPP	PNO7	−2135.768 670	−4.214 527	−0.237 862	0.015
	def2-QZVPP	PNO6	−2135.820 796	−4.437 496	−0.245 391	0.015
	def2-QZVPP	PNO7	−2135.820 796	−4.432 768	−0.256 280	0.015
	CBS(T,Q)/PNO(6,7)		−2135.827 976	−4.590 226	−0.276 011	
LS (doublet)	def2-TZVPP	PNO6	−2135.706 071	−4.273 284	−0.243 443	0.012
	def2-TZVPP	PNO7	−2135.706 071	−4.276 324	−0.253 968	0.012
	def2-QZVPP	PNO6	−2135.757 561	−4.499 950	−0.260 714	0.012
	def2-QZVPP	PNO7	−2135.757 561	−4.497 854	−0.272 777	0.012
	CBS(T,Q)/PNO(6,7)		−2135.764 653	−4.659 001	−0.293 311	
			[Fe]–OH			
HS (sextet)	def2-TZVPP	PNO6	−2210.817 454	−4.388 801	−0.226 507	0.015
	def2-TZVPP	PNO7	−2210.817 454	−4.383 392	−0.235 395	0.014
	def2-QZVPP	PNO6	−2210.875 099	−4.626 693	−0.243 925	0.016
	def2-QZVPP	PNO7	−2210.875 099	−4.616 084	−0.254 582	0.015
	CBS(T,Q)/PNO(6,7)		−2210.883 039	−4.781 217	−0.274 779	
IS (quartet)	def2-TZVPP	PNO6	−2210.703 395	−4.446 939	−0.241 992	0.014
	def2-TZVPP	PNO7	−2210.703 395	−4.446 268	−0.251 542	0.013
	def2-QZVPP	PNO6	−2210.759 641	−4.687 399	−0.259 812	0.014
	def2-QZVPP	PNO7	−2210.759 641	−4.681 541	−0.271 196	0.013
	CBS(T,Q)/PNO(6,7)		−2210.767 388	−4.850 966	−0.292 126	
LS (doublet)	def2-TZVPP	PNO6	−2210.638 446	−4.487 723	−0.254 473	0.012
	def2-TZVPP	PNO7	−2210.638 446	−4.491 063	−0.264 654	0.012
	def2-QZVPP	PNO6	−2210.693 541	−4.731 430	−0.272 720	0.012
	def2-QZVPP	PNO7	−2210.693 541	−4.729 292	−0.284 611	0.012
	CBS(T,Q)/PNO(6,7)		−2210.701 130	−4.902 659	−0.305 973	

Table S13: Total energies (Hartree) corresponding to Table S9 for spin states of hydride transfer TS **A-2-ts** and **A-7-ts**, as well as for substrates *i*$PrNO_2$ and *i*PrNO, together with CC diagnostics for singles amplitudes. Values computed at the PBE0-DLPNO-CCSD(T1) level on RI-PBE-D3(PCM)/def2-SVP/W06 geometries.

			**PBE0-DLPNO-CCSD(T)**			
state	basis	thresh	E_{tot}(ref)	E_{corr}(CCSD)	E_{corr}(T1)	$\mathcal{T}_1$
			**A-2-ts**			
HS (sextet)	def2-TZVPP	PNO6	−2457.624 873	−5.467 275	−0.279 279	0.017
	def2-TZVPP	PNO7	−2457.624 873	−5.463 723	−0.289 635	0.017
	def2-QZVPP	PNO6	−2457.688 904	−5.761 316	−0.301 468	0.017
	def2-QZVPP	PNO7	−2457.688 904	−5.751 349	−0.313 940	0.017
	CBS(T,Q)/PNO(6,7)		−2457.697 723	−5.957 045	−0.338 963	
IS (quartet)	def2-TZVPP	PNO6	−2457.514 932	−5.548 263	−0.297 978	0.018
	def2-TZVPP	PNO7	−2457.514 932	−5.548 774	−0.309 667	0.018
	def2-QZVPP	PNO6	−2457.577 858	−5.846 506	−0.320 603	0.018
	def2-QZVPP	PNO7	−2457.577 858	−5.840 335	−0.334 400	0.018
	CBS(T,Q)/PNO(6,7)		−2457.586 526	−6.050 745	−0.360 401	
LS (doublet)	def2-TZVPP	PNO6	−2457.405 084	−5.637 395	−0.318 600	0.029
	def2-TZVPP	PNO7	−2457.405 084	−5.649 115	−0.323 877	0.037
	def2-QZVPP	PNO6	−2457.466 071	−5.937 076	−0.342 432	0.029
	def2-QZVPP	PNO7	−2457.466 071	−5.942 349	−0.349 201	0.037
	CBS(T,Q)/PNO(6,7)		−2457.474 471	−6.159 806	−0.371 895	
			**A-7-ts**			
HS (sextet)	def2-TZVPP	PNO6	−2382.797 140	−5.205 189	−0.263 231	0.016
	def2-TZVPP	PNO7	−2382.797 140	−5.200 157	−0.273 233	0.016
	def2-QZVPP	PNO6	−2382.858 659	−5.481 355	−0.283 856	0.016
	def2-QZVPP	PNO7	−2382.858 658	−5.470 584	−0.295 914	0.016
	CBS(T,Q)/PNO(6,7)		−2382.867 133	−5.663 390	−0.319 504	
LS (doublet)	def2-TZVPP	PNO6	−2382.591 797	−5.356 605	−0.299 447	0.016
	def2-TZVPP	PNO7	−2382.591 797	−5.361 442	−0.311 346	0.017
	def2-QZVPP	PNO6	−2382.650 831	−5.639 311	−0.321 527	0.016
	def2-QZVPP	PNO7	−2382.650 831	−5.637 193	−0.335 302	0.017
	CBS(T,Q)/PNO(6,7)		−2382.658 962	−5.837 817	−0.360 631	
			i$PrNO_2$			
singlet	def2-TZVPP	PNO6	−321.827 643	−1.265 682	−0.061 568	0.008
	def2-TZVPP	PNO7	−321.827 643	−1.265 995	−0.062 821	0.008
	def2-QZVPP	PNO6	−321.839 566	−1.338 783	−0.067 143	0.008
	def2-QZVPP	PNO7	−321.839 566	−1.337 640	−0.068 786	0.008
	CBS(T,Q)/PNO(6,7)		−321.841 208	−1.389 599	−0.074 170	
			*i*PrNO			
singlet	def2-TZVPP	PNO6	−246.967 988	−1.016 933	−0.045 863	0.009
	def2-TZVPP	PNO7	−246.967 988	−1.017 143	−0.046 694	0.009
	def2-QZVPP	PNO6	−246.976 954	−1.072 391	−0.050 116	0.009
	def2-QZVPP	PNO7	−246.976 954	−1.071 578	−0.051 210	0.009
	CBS(T,Q)/PNO(6,7)		−246.978 189	−1.111 115	−0.055 199	

13.3 Catalytic cycles for reduction of $PhNO_2$

The iron(III)hydride-mediated reduction of $PhNO_2$ follows an analogous reaction pattern to that of $^{i}PrNO_2$ above. Hydride transfer to the nitro moiety is, however, much faster via a lower barrier of 15.6 kcal mol^{-1}. This is fully in line with the experimental observation (see main text). Table S14 lists details on individual species investigated, Figures S369–S371 show the corresponding potential energy surfaces.

Table S14: Total energies (Hartree) for individual species on the high-spin surface pertaining to the interlinked catalytic cycles for reduction of $PhNO_2$ corresponding to Figures S369–S371. For transition structures the imaginary frequency is stated in cm^{-1}. Spin expectation values, DFT-corrections to enthalpies and to Gibbs energies are also shown. Relative energies (kcal mol^{-1}) are given for comparison and correspond to the respective reaction sequence within the catalytic cycle.

	**PBE0-D3(PCM)/def2-TZVP // PBE-D3(PCM)/def2-SVP/W06**							
Species[a]	$\langle S^2 \rangle$	E_{tot}	H_{tot}^{298}	G_{tot}^{298}	ν_{imag}	E_{rel}	H_{rel}	G_{rel}
**$PhNO_2$**	0.000	−436.439 693	0.108 458	0.069 090		0.0	0.0	0.0
**^{6}[Fe]H**	8.762	−2141.572 241	0.287 305	0.218 769		0.0	0.0	0.0
**^{6}A-1.ph**	8.762	−2578.022 751	0.397 612	0.307 316		−6.8	−5.6	5.4
**^{6}A-2-ts.ph**	8.838	−2578.008 219	0.395 833	0.309 042	201 i	2.3	2.4	15.6
**^{6}A-3.ph**	8.771	−2578.083 361	0.399 737	0.307 307		−44.8	−42.3	−32.6
**^{6}B-1.ph**	8.762	−2578.020 390	0.397 760	0.307 647		−5.3	−4.1	7.1
**^{6}B-2-ts.ph**	8.834	−2578.005 596	0.395 616	0.306 778	247 i	4.0	3.9	15.9
**^{6}B-3.ph**	8.809	−2578.055 781	0.400 751	0.310 975		−27.5	−24.4	−13.0
**^{6}B-4-ts.ph**	8.807	−2578.054 093	0.399 691	0.311 999	51 i	−26.5	−24.0	−11.3
**^{6}B-5.ph**	8.763	−2578.086 868	0.400 471	0.310 796		−47.0	−44.1	−32.6
**^{6}B-4H-ts.ph**	8.813	−2578.010 916	0.396 405	0.308 301	1635 i	0.6	1.0	13.5
**^{6}B-5H.ph**	8.765	−2578.067 583	0.401 332	0.312 042		−34.9	−31.4	−19.7
**^{6}C-1.ph**	8.764	−2578.007 843	0.397 368	0.307 274		2.6	3.6	14.8
**^{6}C-2-ts.ph**	8.939	−2577.992 720	0.395 421	0.307 183	580 i	12.1	11.8	24.2
**^{6}C-3.ph**	8.812	−2578.060 244	0.401 049	0.312 897		−30.3	−27.0	−14.6
**^{6}C-4-ts.ph**	8.798	−2578.045 041	0.399 377	0.311 219	259 i	−20.8	−18.5	−6.1
**^{6}C-5.ph**	8.771	−2578.083 361	0.399 737	0.307 307		−44.8	−42.3	−32.6
**^{6}C-4a-ts.ph**	8.800	−2578.043 319	0.396 962	0.311 431	916 i	−19.7	−18.9	−4.9
**^{6}C-5a.ph**	8.806	−2578.051 720	0.402 202	0.312 885		−25.0	−20.9	−9.3
**^{6}C-4b-ts.ph**	8.785	−2578.008 437	0.396 614	0.309 455	1584 i	2.2	2.7	15.8
**^{6}C-5b.ph**	8.763	−2578.068 216	0.401 468	0.313 236		−35.3	−31.7	−19.4
**PhNO**	0.000	−361.262 804	0.102 043	0.064 272		0.0	0.0	0.0
**^{6}[Fe]OH**	8.762	−2216.812 753	0.296 017	0.224 632		−39.9	−38.5	−39.3
**HBpin**	0.000	−411.550 486	0.194 908	0.151 670		0.0	0.0	0.0
**H_2**	0.000	−1.168 056	0.013 045	−0.001 811		0.0	0.0	0.0
**^{6}[Fe]–OBpin**	8.761	−2627.240 465	0.473 707	0.380 552		−68.3	−69.5	−66.2
**pinBOBpin**	0.000	−897.211 129	0.378 847	0.308 610		−63.6	−66.3	−64.4

[a]State denoted by superscript: ^{6} – sextet (UKS high-spin).

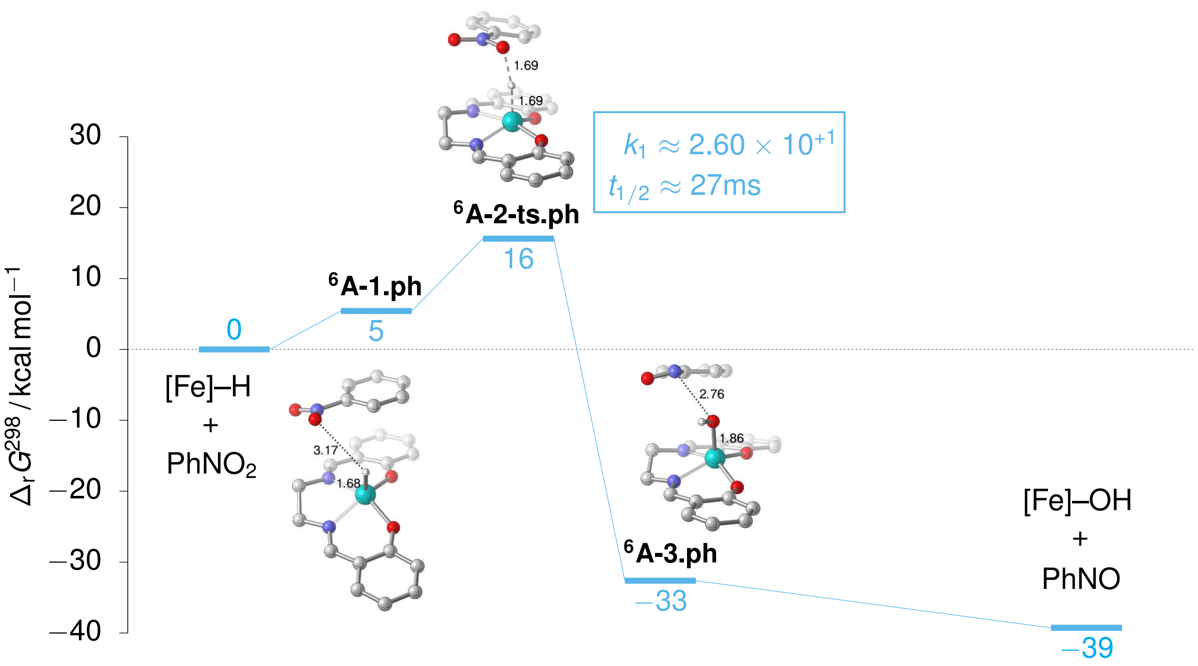

Figure S369: Initial reaction path of first half-cycle for high-spin (HS) iron(III) hydride intermediate **1c** and $PhNO_2$. Blue trace corresponds to path A

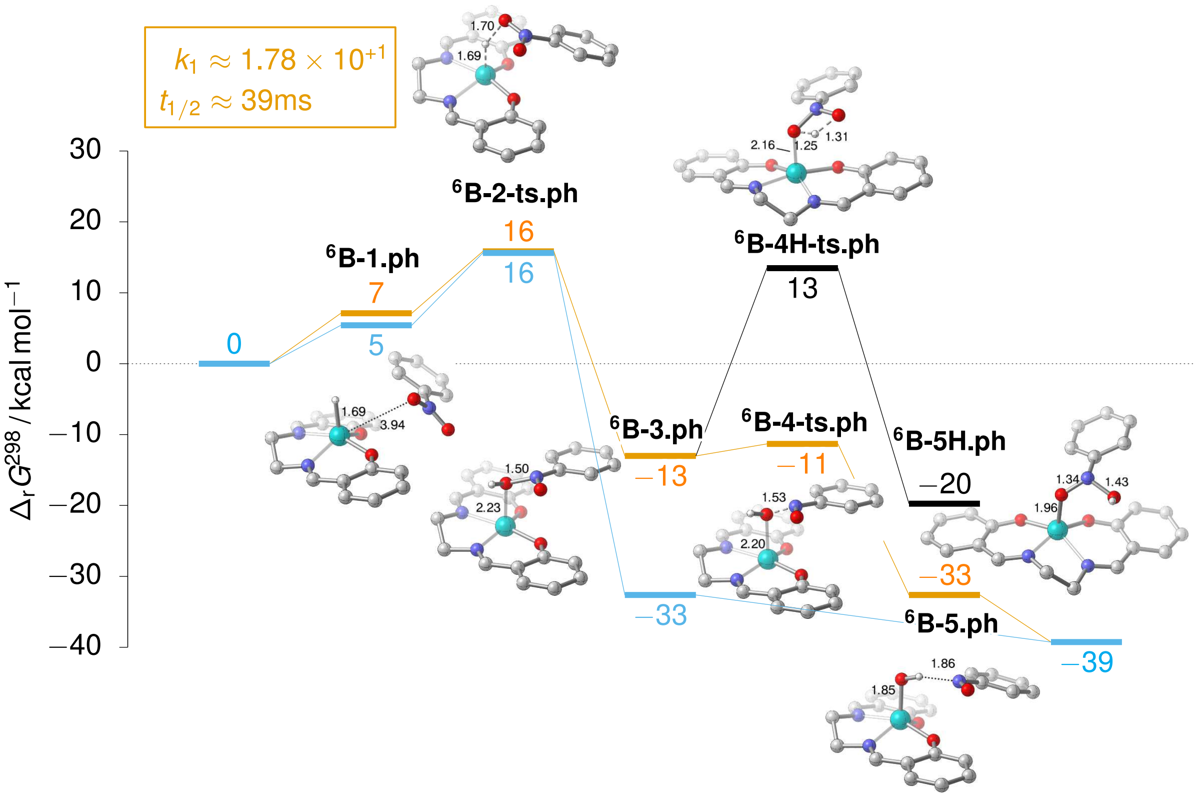

Figure S370: Initial reaction path of first half-cycle for high-spin (HS) iron(III) hydride intermediate **1c** and $PhNO_2$. Yellow trace corresponds to path B, black trace corresponds to side path involving 1,3-H shift at the nitro group.

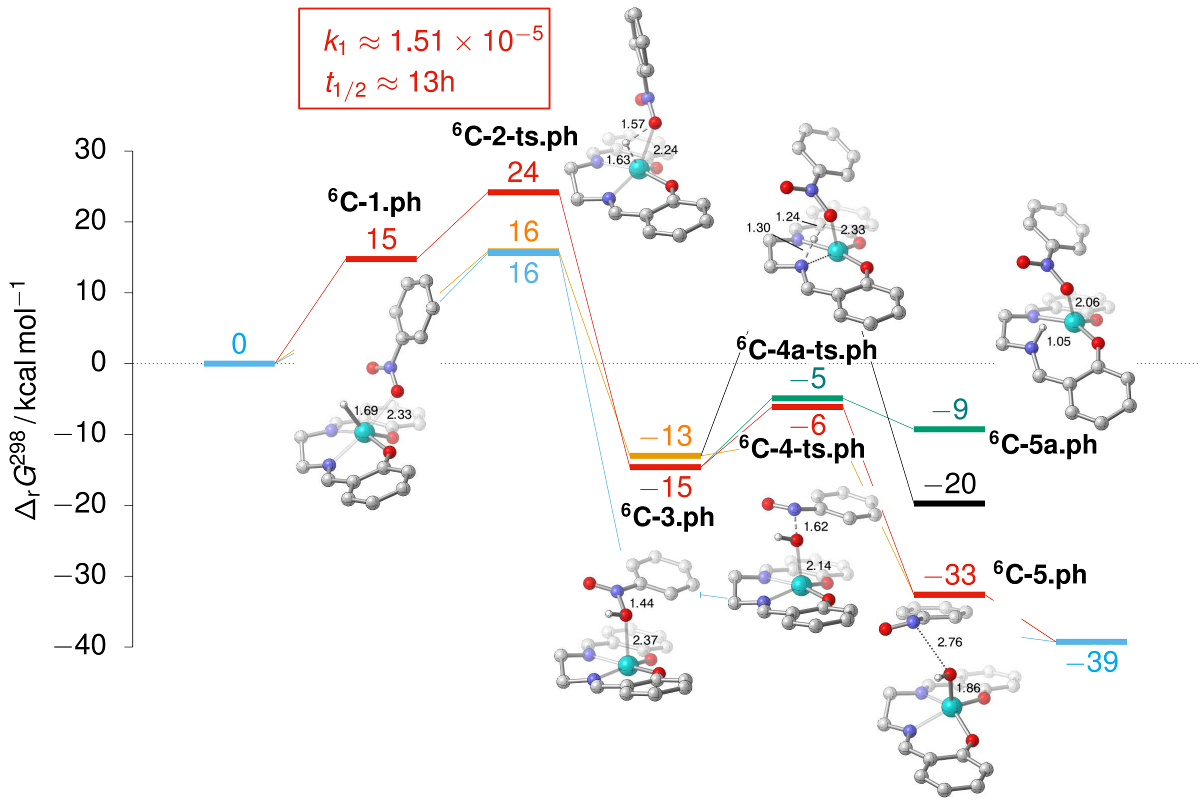

Figure S371: Initial reaction path of first half-cycle for high-spin (HS) iron(III) hydride intermediate **1c** and $PhNO_2$. Red trace corresponds to path C, green trace correponds to side path involving ligand reduction.

13.4 Cartesian coordinates of optimized geometries (Å)

13.4.1 Iron-containing species 1b, 1c, [Fe]–OH, [Fe]–OBpin

35

^{5}[Fe] 1b E_{tot}(UPBE0-D3(Acetonitrile)/def2TZVP // UPBE-D3(Acetonitrile)/def2SVP/W06) = -2141.02724913 $\langle S^2 \rangle$ = 6.0326

Fe	0.000000000000	0.000000000000	0.073145577311
O	0.064760102313	1.553902154977	1.228282850705
O	-0.064760102313	-1.553902154977	1.228282850705
N	0.283280476450	-1.307060519211	-1.527405648381
N	-0.283280476450	1.307060519211	-1.527405648381
C	-0.300888391162	2.615405363520	-1.490601388792
H	-0.443932959216	3.173713312771	-2.442712128343
C	-0.139897459748	3.421794225793	-0.306084429362
C	-0.158194745886	4.838799074217	-0.446731807183
H	-0.301375710022	5.258759685771	-1.456261213947
C	0.000000000000	5.688606411302	0.644676171686
H	-0.017278676025	6.780213676818	0.510272888546
C	0.184586174999	5.125992686568	1.931820777305
H	0.310635416070	5.786658538827	2.804528016220
C	0.207743816653	3.744395988754	2.109399135607
H	0.349184384766	3.299705491799	3.106457273230
C	0.046378641276	2.842098701533	1.011143358238
C	-0.046378641276	-2.842098701533	1.011143358238
C	-0.207743816653	-3.744395988754	2.109399135607
H	-0.349184384766	-3.299705491799	3.106457273230
C	-0.184586174999	-5.125992686568	1.931820777305
H	-0.310635416070	-5.786658538827	2.804528016220
C	-0.000000000000	-5.688606411302	0.644676171686
H	0.017278676025	-6.780213676818	0.510272888546
C	0.158194745886	-4.838799074217	-0.446731807183
H	0.301375710022	-5.258759685771	-1.456261213947
C	0.139897459748	-3.421794225793	-0.306084429362
C	0.300888391162	-2.615405363520	-1.490601388792
H	0.443932959216	-3.173713312771	-2.442712128343
C	0.458397050191	-0.614029445480	-2.796880173151
H	0.246273628431	-1.270487598721	-3.669810848907
H	1.515176558006	-0.273507737203	-2.878239624915
C	-0.458397050191	0.614029445480	-2.796880173151
H	-0.246273628431	1.270487598721	-3.669810848907
H	-1.515176558006	0.273507737203	-2.878239624915

35

^{3}[Fe] 1b E_{tot}(UPBE0-D3(Acetonitrile)/def2TZVP // UPBE-D3(Acetonitrile)/def2SVP/W06) = -2141.01835135 $\langle S^2 \rangle$ = 2.0350

Fe	0.000000000000	0.000000000000	0.225534357033
O	-0.000000000000	1.298165354407	-1.155186280352
O	-0.000000000000	-1.298165354407	-1.155186280352
N	-0.157641072886	-1.270548628033	1.607209732095
N	0.157641072886	1.270548628033	1.607209732095
C	0.171481336796	2.582574918097	1.481273595423
H	0.277458503243	3.190247107765	2.402311911018
C	0.066169274494	3.293324946298	0.241532036317
C	0.052464581559	4.719584402833	0.263555292723
H	0.117253227917	5.225654704780	1.240792620307
C	-0.040021038935	5.467083438245	-0.904933633392
H	-0.050130353377	6.566311843112	-0.865551178232
C	-0.120126390964	4.792536947893	-2.150714529838
H	-0.193063125206	5.374440236133	-3.083355047242
C	-0.106584040063	3.402384139079	-2.210597147858
H	-0.166842655835	2.870751861329	-3.172563045694
C	-0.013227800262	2.599353315333	-1.029322366403
C	0.013227800262	-2.599353315333	-1.029322366403
C	0.106584040063	-3.402384139079	-2.210597147858
H	0.166842655835	-2.870751861329	-3.172563045694
C	0.120126390964	-4.792536947893	-2.150714529838
H	0.193063125206	-5.374440236133	-3.083355047242
C	0.040021038935	-5.467083438245	-0.904933633392
H	0.050130353377	-6.566311843112	-0.865551178232
C	-0.052464581559	-4.719584402833	0.263555292723
H	-0.117253227917	-5.225654704780	1.240792620307
C	-0.066169274494	-3.293324946298	0.241532036317
C	-0.171481336796	-2.582574918097	1.481273595423
H	-0.277458503243	-3.190247107765	2.402311911018
C	-0.370916497985	-0.664932010661	2.929483800500

H	-0.027677448675	-1.330422144616	3.749322725740
H	-1.460694426422	-0.488961573167	3.066455206005
C	0.370916497985	0.664932010661	2.929483800500
H	0.027677448675	1.330422144616	3.749322725740
H	1.460694426422	0.488961573167	3.066455206005

35

[1]**[Fe] 1b** E_{tot}(RPBE0-D3(Acetonitrile)/def2TZVP // RPBE-D3(Acetonitrile)/def2SVP/W06) = -2140.95837362 $\langle S^2 \rangle$ = 0.0000

Fe	0.000000000000	0.000000000000	0.187419511151
O	-0.246477573802	1.329324184407	-1.051904988374
O	0.246477573802	-1.329324184407	-1.051904988374
N	-0.482475606721	-1.165446923010	1.513381724898
N	0.482475606721	1.165446923010	1.513381724898
C	0.688353666540	2.475559816869	1.427786655710
H	0.984138468088	3.016810410088	2.346319318706
C	0.506202852191	3.239787924185	0.233932994093
C	0.752377337171	4.643757338664	0.233146274676
H	1.135048743276	5.110021388702	1.155272921686
C	0.514130548304	5.419110245107	-0.897337927514
H	0.716101497262	6.500474733333	-0.878789786501
C	0.000000000000	4.811706891474	-2.071935569959
H	-0.191330282998	5.424176591085	-2.966737074235
C	-0.266923503868	3.445091294385	-2.101243745553
H	-0.667680328342	2.956145183135	-3.001976770639
C	-0.004841962754	2.619617839797	-0.968950000819
C	0.004841962754	-2.619617839797	-0.968950000819
C	0.266923503868	-3.445091294385	-2.101243745553
H	0.667680328342	-2.956145183135	-3.001976770639
C	-0.000000000000	-4.811706891474	-2.071935569959
H	0.191330282998	-5.424176591085	-2.966737074235
C	-0.514130548304	-5.419110245107	-0.897337927514
H	-0.716101497262	-6.500474733333	-0.878789786501
C	-0.752377337171	-4.643757338664	0.233146274676
H	-1.135048743276	-5.110021388702	1.155272921686
C	-0.506202852191	-3.239787924185	0.233932994093
C	-0.688353666540	-2.475559816869	1.427786655710
H	-0.984138468088	-3.016810410088	2.346319318706
C	-0.558964467930	-0.519243255389	2.833446221867
H	-0.450826160993	-1.255162655433	3.657261166733
H	-1.544102227499	-0.016123004720	2.940694996983
C	0.558964467930	0.519243255389	2.833446221867
H	0.450826160993	1.255162655433	3.657261166733
H	1.544102227499	0.016123004720	2.940694996983

36

[6]**[Fe]--H 1c** E_{tot}(UPBE0-D3(Acetonitrile)/def2TZVP // UPBE-D3(Acetonitrile)/def2SVP/W06) = -2141.57224131 $\langle S^2 \rangle$ = 8.7617

Fe	-0.019663795677	-0.002082873990	0.603532884711
O	-1.490005450097	-1.189184856471	0.176059268612
O	1.445350243459	-1.168042721410	0.153107472156
N	1.363230443662	1.596844377776	0.307651137710
N	-1.242472690736	1.547135711145	-0.235103979966
C	-2.534855868080	1.479340893361	-0.392457692956
H	-3.080665630854	2.404882994130	-0.681091569342
C	-3.348817097076	0.296824601919	-0.247996745627
C	-4.755227527529	0.421266439701	-0.415496343274
H	-5.171328846077	1.421378672590	-0.618863714864
C	-5.600171178931	-0.682995785568	-0.328681327092
H	-6.686154289945	-0.567217138586	-0.456266809348
C	-5.038854361681	-1.957802683659	-0.079519626608
H	-5.695992416551	-2.839306954509	-0.011898938297
C	-3.663014869847	-2.118827064469	0.080866328517
H	-3.221900396981	-3.108122063121	0.273548285767
C	-2.773765749793	-1.004815507834	0.012249574278
C	2.732177332455	-1.017182249037	-0.032976498752
C	3.552604686979	-2.171213395731	-0.208763327706
H	3.057997525893	-3.153421715334	-0.176417601661
C	4.927789730113	-2.059129273188	-0.411140019578
H	5.529556336552	-2.972688896913	-0.540250575648
C	5.557107042107	-0.792938241825	-0.454954129336
H	6.641815220601	-0.714460073209	-0.617201210191
C	4.780650003152	0.352404020434	-0.291786570045
H	5.250245267513	1.348887663964	-0.326278518881
C	3.377972411702	0.276325207783	-0.076027343375
C	2.638467437881	1.508351110071	0.069158968904
H	3.227061673881	2.446450142166	-0.039981809863
C	0.692787793524	2.884286356266	0.380794916141

H	1.350768788485	3.727041761418	0.077417792001
H	0.373246123711	3.055132671692	1.432783602440
C	-0.546618595957	2.800839291271	-0.516407607108
H	-1.205292536882	3.685247404438	-0.378254660910
H	-0.217916642175	2.785684793341	-1.579471950909
H	-0.117631241058	0.096223145284	2.288033308593

36

[4][Fe]--H 1c E_{tot}(UPBE0-D3(Acetonitrile)/def2TZVP // UPBE-D3(Acetonitrile)/def2SVP/W06) = -2141.56177026 $\langle S^2 \rangle$ = 3.8193

Fe	0.014702288976	0.243192357330	0.626186973017
O	-1.435714440951	-0.949220568356	0.869839367052
O	1.159640781822	-1.045159859228	-0.280163384289
N	1.335283253079	1.605685854573	0.462121301018
N	-1.132835464571	1.549508133258	-0.362889311357
C	-2.392240472214	1.400084207524	-0.671734578101
H	-2.918099026293	2.234738331728	-1.181878687268
C	-3.174489705889	0.226477244463	-0.386682897566
C	-4.521400461627	0.188164517944	-0.847244668105
H	-4.901576894684	1.053388310608	-1.414342921966
C	-5.347326356929	-0.904146955694	-0.601879766563
H	-6.383019403044	-0.917141683178	-0.971005269886
C	-4.832723657699	-1.998335511630	0.135784980340
H	-5.474009326109	-2.870976939595	0.337658726444
C	-3.526098497630	-1.987403860335	0.617128106886
H	-3.123897276931	-2.831614999718	1.196939806708
C	-2.646459766744	-0.886082846109	0.376391314483
C	2.469917672912	-1.026437394168	-0.266127437570
C	3.190823281650	-2.220769799661	-0.571780762286
H	2.600527191322	-3.124517761818	-0.785552328523
C	4.584657491077	-2.247082491883	-0.590858453582
H	5.103235414158	-3.191883742497	-0.818955821678
C	5.338662312158	-1.078033941888	-0.329887235793
H	6.437726945791	-1.105806636870	-0.355523342471
C	4.667359226295	0.110556888667	-0.051689709321
H	5.236203531256	1.035542039211	0.136391542321
C	3.247731580781	0.168144687252	0.004421038366
C	2.608205967160	1.444242592314	0.203663235267
H	3.228732261043	2.354891656258	0.083154876808
C	0.698634980517	2.922513133214	0.399636984222
H	1.424703818456	3.715994435524	0.126872745247
H	0.267271814333	3.161181042898	1.395129850550
C	-0.425166445820	2.801841815562	-0.640101789591
H	-1.102755057731	3.680790601093	-0.613908087021
H	0.026250469043	2.742700454577	-1.655043908652
H	0.347377891515	0.126005393616	2.152939564701

36

[2][Fe]--H 1c E_{tot}(UPBE0-D3(Acetonitrile)/def2TZVP // UPBE-D3(Acetonitrile)/def2SVP/W06) = 2141.57190682 $\langle S^2 \rangle$ = 0.8010

Fe	-0.001937817341	0.247553288623	0.057305726218
O	-1.267035817197	-1.125386835743	-0.104105513295
O	1.267821984094	-1.130686180101	0.034490794758
N	1.269138857499	1.613259100900	0.102664523729
N	-1.266661029164	1.608601108578	-0.147817000885
C	-2.578587581831	1.482066585975	-0.139974944258
H	-3.188949391332	2.400392844894	-0.249378212191
C	-3.278968981005	0.237343359068	-0.034594639948
C	-4.703233901042	0.240880749140	0.022409875654
H	-5.223787574478	1.212354312754	0.022716497423
C	-5.430753775708	-0.942978491803	0.075661604918
H	-6.529359777467	-0.920264804739	0.124979861169
C	-4.740330441776	-2.182392762956	0.059922246837
H	-5.309313638440	-3.124724910837	0.099791375146
C	-3.350814587050	-2.223242045451	-0.004624379956
H	-2.805554635917	-3.179127262255	-0.017389756909
C	-2.570773763353	-1.025597058426	-0.042972130389
C	2.571853976244	-1.027185536529	0.002331102258
C	3.352855208097	-2.223785695625	-0.062083901019
H	2.807156642745	-3.179357997456	-0.079265098951
C	4.742997950069	-2.182711861522	-0.097901082963
H	5.312260667591	-3.124560878285	-0.144859325555
C	5.434436771613	-0.943618999259	-0.071435326295
H	6.533807426100	-0.920704288769	-0.098221242780
C	4.706271559565	0.238948366210	-0.010463329573
H	5.226707327295	1.210280433024	0.011082732954
C	3.280739546935	0.235634279005	0.025250799834
C	2.581327128211	1.482863831991	0.089243993279

H	3.193494270314	2.405241911368	0.135818547416
C	0.680012041146	2.944938648674	0.299468076997
H	1.338445657291	3.747322843867	-0.092848451101
H	0.546907396454	3.112102077283	1.390303845140
C	-0.673039765722	2.931292535179	-0.395737339068
H	-1.332342115219	3.750190712586	-0.040392853988
H	-0.536833124583	3.054414212010	-1.492304477694
H	-0.149838319352	0.360882528753	1.525999008806

37

[6][Fe]--OH E_{tot}(UPBE0-D3(Acetonitrile)/def2TZVP // UPBE-D3(Acetonitrile)/def2SVP/W06) = -2216.81275312 $\langle S^2 \rangle$ = 8.7623

Fe	-0.019492832122	0.007096232623	0.510633798803
O	-1.476362758703	-1.206307940550	0.058250910412
O	1.467992176042	-1.181155228746	0.151598861641
N	1.370774941881	1.580348431653	0.124362214812
N	-1.252073959223	1.532217136889	-0.376918241833
C	-2.542790295470	1.455187556346	-0.531578220688
H	-3.096766107283	2.373454412861	-0.829336092755
C	-3.346361971646	0.266549632839	-0.374506074745
C	-4.754344703248	0.377458242429	-0.542485737383
H	-5.178053716693	1.373179680463	-0.752560925036
C	-5.591118245373	-0.731891790399	-0.449103341630
H	-6.677971965384	-0.625457908653	-0.578098488558
C	-5.019294868168	-2.001043940027	-0.191392412400
H	-5.669286413320	-2.887613542775	-0.118852858703
C	-3.643175784716	-2.149222590507	-0.028050558165
H	-3.194150004868	-3.133823328812	0.171287254425
C	-2.759475203254	-1.028973827466	-0.103520086307
C	2.746635117374	-1.035411714239	-0.082298129270
C	3.571177781624	-2.194170065452	-0.209597801064
H	3.084241348900	-3.174832947315	-0.100598805561
C	4.938503429395	-2.088987078279	-0.460825489615
H	5.541929330788	-3.006161273872	-0.551534041485
C	5.558739203858	-0.824958042444	-0.600680380124
H	6.637905957242	-0.751613738491	-0.798729692833
C	4.780149866346	0.324354435999	-0.483144737785
H	5.242876603021	1.319225287620	-0.588457882131
C	3.384117082319	0.255840581208	-0.225608732907
C	2.644229567161	1.491737448120	-0.117038637472
H	3.230470533455	2.428307835523	-0.251964215454
C	0.694051130795	2.862384348695	0.215752057220
H	1.342734255267	3.714306466220	-0.082872776911
H	0.383622682666	3.002906310269	1.274336290468
C	-0.557757565639	2.783718198854	-0.664847513061
H	-1.211355227212	3.670392546394	-0.514945929525
H	-0.242960545930	2.772076551815	-1.732400685564
O	-0.214269413639	0.374670895337	2.322640409067
H	0.644082748190	0.480102438369	2.779775593316

37

[4][Fe]--OH E_{tot}(UPBE0-D3(Acetonitrile)/def2TZVP // UPBE-D3(Acetonitrile)/def2SVP/W06) = -2216.79387772 $\langle S^2 \rangle$ = 3.7964

Fe	-0.007425314177	0.212082203486	0.358807028348
O	-1.319980667524	-1.154569341602	0.229771456239
O	1.303890916669	-1.176307137466	0.008769698830
N	1.318164191656	1.587206535958	0.143520211234
N	-1.214013871409	1.555945976976	-0.411549707278
C	-2.505162941479	1.430449512272	-0.560596809231
H	-3.084482534323	2.321092456389	-0.881240770565
C	-3.249829106301	0.216718193567	-0.361940962683
C	-4.658135649388	0.243768607589	-0.568516496764
H	-5.125855382139	1.201507452852	-0.848670678853
C	-5.437340397779	-0.900591598548	-0.429220740169
H	-6.524259600737	-0.860665089744	-0.590548588569
C	-4.806696706610	-2.119651505269	-0.079816320049
H	-5.410444166623	-3.034293842978	0.031551723637
C	-3.431041528457	-2.182381189013	0.128929745075
H	-2.936597114113	-3.126139354778	0.403368450775
C	-2.602848005841	-1.023942300935	0.007962998486
C	2.593752605635	-1.061131394005	-0.124526485750
C	3.380592256924	-2.247921570261	-0.278200600580
H	2.845774254437	-3.209472204789	-0.267807398404
C	4.763056653001	-2.191196966607	-0.425841339559
H	5.331647565565	-3.128865186712	-0.532341138088
C	5.449911257872	-0.950273882959	-0.443069894196
H	6.542530064532	-0.919409151237	-0.562291785231
C	4.717904359464	0.224013246075	-0.310650868347

H	5.227505622953	1.201036540371	-0.327896781751
C	3.302816074184	0.203398830801	-0.146657513244
C	2.604028428612	1.451220932093	-0.045491461410
H	3.209318308717	2.375240779394	-0.145786453791
C	0.678443458860	2.899364977293	0.214124554304
H	1.367860971850	3.718889490989	-0.076439965660
H	0.349422070459	3.052185959399	1.264320297421
C	-0.546362562399	2.831202524784	-0.697841692497
H	-1.225382152801	3.695817743406	-0.545473120897
H	-0.219058485924	2.830838059740	-1.760757346141
O	-0.148757802392	0.466504940780	2.216125089727
H	-1.083722246791	0.342725271565	2.478030652710

37

^{2}[Fe]--OH E_{tot}(UPBE0-D3(Acetonitrile)/def2TZVP // UPBE-D3(Acetonitrile)/def2SVP/W06) = -2216.78092270 $\langle S^2 \rangle$ = 0.9566

Fe	0.004923934086	0.224670942185	0.002204384657
O	-1.268154476174	-1.164445999607	-0.240734292534
O	1.287480212382	-1.138407106211	-0.198711846953
N	1.283291975380	1.612337945302	0.030772681820
N	-1.256457001931	1.606470569146	-0.317188725434
C	-2.560625574024	1.473502695927	-0.299935634006
H	-3.177425238646	2.384560652419	-0.438483719031
C	-3.261002717381	0.231575865045	-0.144395769078
C	-4.683228758408	0.253489269973	-0.054338525076
H	-5.191444559027	1.231384183402	-0.064722140315
C	-5.424974979761	-0.918283107902	0.043173748907
H	-6.521525492666	-0.880410625407	0.118698571404
C	-4.748799780998	-2.165401381107	0.037530292539
H	-5.327198085862	-3.099941203942	0.112091823713
C	-3.362382350326	-2.225471828133	-0.059565444683
H	-2.829689435799	-3.188387425017	-0.065276606855
C	-2.565066907813	-1.040056030601	-0.141774676230
C	2.591839164460	-1.026315264097	-0.142478028830
C	3.380461047047	-2.214682979494	-0.233361528355
H	2.840951795732	-3.168602173910	-0.333352930394
C	4.771305152279	-2.170653501774	-0.185021304355
H	5.345224571283	-3.108897182670	-0.245439837658
C	5.453900585821	-0.935337699371	-0.056025996441
H	6.552749643123	-0.908219853066	-0.017533592309
C	4.715995175606	0.241923672531	0.019193149626
H	5.230002232210	1.212266453945	0.113692831444
C	3.292299244296	0.233852279574	-0.020253047612
C	2.588858576929	1.484249060320	0.037505323707
H	3.201711888190	2.406536422765	0.085437844365
C	0.668101926091	2.929276138455	0.210487422702
H	1.338594966111	3.751374243009	-0.115579868366
H	0.457327175139	3.057789475255	1.295068327793
C	-0.640291813859	2.914010110511	-0.568575882921
H	-1.310972434498	3.747758247540	-0.274471650546
H	-0.432645926727	3.007747353020	-1.657197145409
O	-0.194332948966	0.238931870649	1.787560540498
H	-0.389798436650	-0.700756583071	1.996766588915

57

^{6}[Fe]--OBpin E_{tot}(UPBE0-D3(Acetonitrile)/def2TZVP // UPBE-D3(Acetonitrile)/def2SVP/W06) = -2627.24046539 $\langle S^2 \rangle$ = 8.7614

Fe	-1.064771294378	-0.395455487003	-0.028298626516
O	-0.112571033195	-2.026171264177	-0.495671589837
O	-2.731374990845	-0.761398770839	-0.921834416035
N	-2.198371232642	0.881524987618	1.243339208849
N	-0.043772683340	-0.557452319056	1.848064940332
C	1.116299228786	-1.130442212432	1.993936137307
H	1.689152172049	-0.936005112349	2.926922323730
C	1.747963403439	-2.014079181086	1.045776711206
C	3.051397817356	-2.500436294618	1.338792505299
H	3.535589363370	-2.169086607647	2.271743329332
C	3.715976862670	-3.368506197114	0.475826227694
H	4.727703073847	-3.727196094279	0.713802668970
C	3.067996352110	-3.784980943506	-0.711644524043
H	3.582801782602	-4.472361221639	-1.401395178161
C	1.785195392914	-3.338307758643	-1.025294811978
H	1.277567461661	-3.657751340397	-1.947565403339
C	1.086534549379	-2.434540682994	-0.169527327324
C	-3.965374640029	-0.338022137479	-0.806406755176
C	-4.955748188133	-0.821752848606	-1.712077117535
H	-4.632234370553	-1.541483900252	-2.478708413885
C	-6.282074743273	-0.398892789606	-1.629614604631

H	-7.018986601681	-0.793962516312	-2.346696802155
C	-6.691534927213	0.526116636789	-0.641028685195
H	-7.739544292714	0.853900306037	-0.583374335996
C	-5.746331370999	1.014610050183	0.258629895533
H	-6.044616901413	1.735223876765	1.037391497155
C	-4.385897284956	0.607692327195	0.203645549939
C	-3.466933907248	1.152743378225	1.176269003957
H	-3.901434690061	1.857906361783	1.919260230497
C	-1.346181262492	1.445902044932	2.275894025365
H	-1.919384898513	2.015745776348	3.038840787549
H	-0.616703474913	2.130328931825	1.789820030039
C	-0.576354431813	0.283800047512	2.914332418743
H	0.223880553840	0.656920548396	3.589264693224
H	-1.281776917802	-0.324595124155	3.522853669849
O	-0.112089643931	0.944273230094	-0.942464533461
B	1.187036222081	1.288876404112	-0.820474539852
O	2.052029444303	1.442600775353	-1.902673368629
O	1.817339783644	1.538405668538	0.401112356484
C	3.399072664767	1.533834271069	-1.361877050892
C	3.139307601959	2.064264240692	0.110906480766
C	3.043638221751	3.593947012979	0.187436077612
C	4.117846908209	1.538376418003	1.159340620456
C	4.225755052374	2.468774220805	-2.241516604799
C	3.987401703707	0.116407353274	-1.388054004781
H	4.360144958654	2.018336117841	-3.245109172618
H	3.733778031264	3.450602171029	-2.370940048238
H	3.936088305022	-0.273396569740	-2.423721660423
H	4.030721217354	4.077384677403	0.051538386790
H	2.349117307968	3.990069464780	-0.579868866649
H	3.411512753678	-0.569096630651	-0.736272377635
H	5.046113363422	0.101850564864	-1.063576646171
H	5.230884604742	2.631563315881	-1.803698623080
H	2.651988674786	3.878805588933	1.184001434476
H	5.155266063398	1.846225444952	0.919797234691
H	4.082554483754	0.435343071558	1.229585650162
H	3.859299432160	1.952962015409	2.154200942976

13.4.2 Species related to HBpin

2
H_2 E_{tot}(RPBE0-D3(Acetonitrile)/def2TZVP // RPBE-D3(Acetonitrile)/def2SVP/W06) = -1.16805599 $\langle S^2 \rangle$ = 0.0000

H	0.000000000000	0.000000000000	0.384232743189
H	0.000000000000	0.000000000000	-0.384232743189

22
HBpin E_{tot}(RPBE0-D3(Acetonitrile)/def2TZVP // RPBE-D3(Acetonitrile)/def2SVP/W06) = -411.55048615 $\langle S^2 \rangle$ = 0.0000

B	0.000000000000	-0.000000000000	1.948345438090
O	-1.002004356641	-0.569656910073	1.207368608211
O	1.002004356641	0.569656910073	1.207368608211
C	-0.556397887792	-0.564955663573	-0.192200891528
C	0.556397887792	0.564955663573	-0.192200891528
C	-0.000000000000	1.965055057560	-0.476186972272
C	1.759551079169	0.278723969669	-1.085956816614
C	-1.759551079169	-0.278723969669	-1.085956816614
C	0.000000000000	-1.965055057560	-0.476186972272
H	-2.481893200745	-1.116160609530	-1.021197240798
H	-2.282290900691	0.648510011217	-0.787821303934
H	-0.783953795703	-2.715306987404	-0.254400704816
H	-0.295832997861	2.076214525499	-1.537205628056
H	-0.878740091137	2.188865122116	0.160071483163
H	0.878740091137	-2.188865122116	0.160071483163
H	0.295832997861	-2.076214525499	-1.537205628056
H	-1.443026836599	-0.181581968246	-2.143412825433
H	0.783953795703	2.715306987404	-0.254400704816
H	1.443026836599	0.181581968246	-2.143412825433
H	2.282290900691	-0.648510011217	-0.787821303934
H	2.481893200745	1.116160609530	-1.021197240798
H	0.000000000000	-0.000000000000	3.160443682894

23
pinB-OH E_{tot}(RPBE0-D3(Acetonitrile)/def2TZVP // RPBE-D3(Acetonitrile)/def2SVP/W06) = -486.79992963 $\langle S^2 \rangle$ = 0.0000

B	-1.594635853959	-0.052347585032	-0.012924188598
O	-0.876828257340	1.043119098033	0.431842854856

O	-0.811051807156	-1.102998054719	-0.447350031845
C	0.513214232854	0.800089203673	0.051538285082
C	0.556769620233	-0.781710341730	-0.049722135054
C	0.800546642041	-1.457345276351	1.305801383219
C	1.517847684382	-1.329419392395	-1.100671179023
C	1.428389337881	1.400190528760	1.114516286923
C	0.733986048679	1.489783801506	-1.300559494186
H	1.334236391175	2.504217497066	1.111387776266
H	1.172137828164	1.037680817929	2.127071571545
H	0.468722801117	2.560921126832	-1.204613911771
H	1.846574928351	-1.329690490579	1.645648483528
H	0.125582552971	-1.049126460442	2.084137663913
H	0.092328243684	1.045091561019	-2.087006950565
H	1.789456775529	1.421636811825	-1.628140088494
H	2.486951761974	1.147854846703	0.905138071875
H	0.596833499648	-2.541681345074	1.207981468142
H	2.558118071864	-1.019722140326	-0.877147390773
H	1.255557760943	-0.980492551942	-2.116470074309
H	1.484117126779	-2.436924155119	-1.099174881552
O	-2.959167680705	-0.131856461511	-0.032438046740
H	-3.365577907217	0.706324612062	0.253952113254

43

pinB-O-Bpin E_{tot}(RPBE0-D3(Acetonitrile)/def2TZVP // RPBE-D3(Acetonitrile)/def2SVP/W06) = -897.21112873 $\langle S^2 \rangle$ = 0.0000

B	-0.000000000000	1.243852234012	0.693457781918
O	0.354507530699	1.502528489567	-0.611965691082
O	-0.338204258616	2.361496785240	1.424476403918
C	0.476317203944	2.956013160203	-0.725174443082
C	-0.439551620397	3.462438852900	0.466818640918
C	-1.918516845703	3.575547630243	0.077317784918
C	0.036620615156	4.745900120664	1.139939103918
C	0.012020636732	3.380603990563	-2.115001772082
C	1.959065321049	3.292848928042	-0.526836563082
H	0.708185595152	2.983850433415	-2.880104402082
H	-1.000303861024	2.999175395458	-2.342731701082
H	2.557502008344	2.727156434117	-1.267534872082
H	-2.099247425157	4.438413719866	-0.592342084082
H	-2.272917248255	2.656424652872	-0.430179364082
H	2.306099280118	3.007029232016	0.485986868918
H	2.155396181730	4.372498700586	-0.673745595082
H	0.002710640429	4.485205979394	-2.201864044082
H	-2.522007066260	3.715922668293	0.995371605918
H	0.056537152152	5.582019065615	0.413070191918
H	1.046914347930	4.630918827064	1.573837651918
H	-0.658973724233	5.020952457414	1.957227510918
O	0.000000000000	0.000000000000	1.273314277918
B	-0.000000000000	-1.243852234012	0.693457781918
O	0.338204258616	-2.361496785240	1.424476403918
O	-0.354507530699	-1.502528489567	-0.611965691082
C	0.439551620397	-3.462438852900	0.466818640918
C	-0.476317203944	-2.956013160203	-0.725174443082
C	-1.959065321049	-3.292848928042	-0.526836563082
C	-0.012020636732	-3.380603990563	-2.115001772082
C	-0.036620615156	-4.745900120664	1.139939103918
C	1.918516845703	-3.575547630243	0.077317784918
H	0.658973724233	-5.020952457414	1.957227510918
H	-1.046914347930	-4.630918827064	1.573837651918
H	2.522007066260	-3.715922668293	0.995371605918
H	-2.155396181730	-4.372498700586	-0.673745595082
H	-2.306099280118	-3.007029232016	0.485986868918
H	2.272917248255	-2.656424652872	-0.430179364082
H	2.099247425157	-4.438413719866	-0.592342084082
H	-0.056537152152	-5.582019065615	0.413070191918
H	-2.557502008344	-2.727156434117	-1.267534872082
H	-0.002710640429	-4.485205979394	-2.201864044082
H	1.000303861024	-2.999175395458	-2.342731701082
H	-0.708185595152	-2.983850433415	-2.880104402082

33

Bpin-NH^{i}Pr E_{tot}(RPBE0-D3(Acetonitrile)/def2TZVP // RPBE-D3(Acetonitrile)/def2SVP/W06) = -584.75597937 $\langle S^2 \rangle$ = 0.0000

B	-0.438012440388	-0.337048601008	-0.246795469808
O	0.576830611828	-1.053243324316	-0.876371196203
O	0.040264666280	0.668575336745	0.590241664641
C	1.820867375080	-0.650906871370	-0.235949237362
C	1.463075370432	0.792006774629	0.311351598644
C	1.625596506147	1.886941471623	-0.752266665156

C	2.185887741574	1.187428143917	1.596504311276
C	2.942361504329	-0.682987324800	-1.271310311260
C	2.102203797385	-1.657520441254	0.888337058679
H	3.127259449437	-1.727108067544	-1.593497708989
H	2.686483434197	-0.089270457146	-2.168368716421
H	2.119165691898	-2.678597130679	0.458931818076
H	2.691093836575	2.103104358694	-0.963262542548
H	1.130567858917	1.599293955608	-1.701116735119
H	1.310341356246	-1.623377485598	1.662846801836
H	3.078781472669	-1.469715031874	1.375760823682
H	3.884061235640	-0.287042636885	-0.841309896074
H	1.148870956948	2.816765989162	-0.384216604942
H	3.283650718303	1.191202798295	1.443790746100
H	1.946712441218	0.497912267728	2.427135918171
H	1.880383612808	2.208300934725	1.900916643287
N	-1.811736568339	-0.597991942291	-0.438899509076
H	-2.090520819372	-1.394767855911	-1.016086136056
C	-2.915835745488	0.166343257467	0.135067814908
H	-2.449036357777	0.998665777930	0.703805573198
C	-3.735235016151	-0.685973698754	1.115602986656
C	-3.801784328404	0.771886605046	-0.963418064532
H	-3.205704769157	1.414954797265	-1.641572427848
H	-4.269025905336	-0.029554408495	-1.575408759940
H	-4.619384533211	1.383670583503	-0.530426030659
H	-4.201010613531	-1.547389357359	0.590259153206
H	-4.550206722037	-0.094656065367	1.581029307650
H	-3.089849618417	-1.087169963427	1.922581987322

13.4.3 Species pertinent to reduction of ^{i}PrNO$_2$

13

^{i}Pr-NO$_2$ E_{tot}(RPBE0-D3(Acetonitrile)/def2TZVP // RPBE-D3(Acetonitrile)/def2SVP/W06) = -323.42653265 $\langle S^2 \rangle$ = 0.0000

O	-1.638988735989	0.800674528425	-0.351043105146
N	-0.851353702105	-0.093187516762	-0.045924700864
O	-1.160489824896	-1.137245997184	0.522080979219
C	0.628539554965	0.148588247704	-0.395718604391
H	0.579831133244	0.427739813785	-1.468243396660
C	1.443023224564	-1.116655193087	-0.195708011025
C	1.107945428914	1.347504540495	0.423142944652
H	0.457619842945	2.225527935070	0.251205275892
H	1.114978581655	1.107228226690	1.505510347812
H	2.139873070602	1.607988267990	0.118433601520
H	1.465722162172	-1.413284137571	0.870688501660
H	2.482590061593	-0.927264520718	-0.528268602096
H	1.037640298950	-1.959676788513	-0.786453790088

12

^{i}Pr-NO E_{tot}(RPBE0-D3(Acetonitrile)/def2TZVP // RPBE-D3(Acetonitrile)/def2SVP/W06) = -248.24193185 $\langle S^2 \rangle$ = 0.0000

N	-0.836359920627	-0.796202967830	0.230101651779
O	-1.782409222302	-0.213850791982	-0.257803004400
C	0.404186434679	0.040328294834	0.409071801029
H	0.538007817281	-0.011299286663	1.517322984787
C	0.269825369805	1.478889057509	-0.060234669991
C	1.550135157415	-0.752608469407	-0.234417880445
H	1.532843687689	-1.810101318009	0.095709652591
H	1.463342382402	-0.730260211337	-1.340070554170
H	2.525541042956	-0.311222621666	0.047452909343
H	0.110656806133	1.520178419050	-1.157117723199
H	1.189766655861	2.046744974748	0.179004410434
H	-0.591246940912	1.980533856917	0.422895289408

13

^{i}Pr-NH$_2$ E_{tot}(RPBE0-D3(Acetonitrile)/def2TZVP // RPBE-D3(Acetonitrile)/def2SVP/W06) = -174.34671343 $\langle S^2 \rangle$ = 0.0000

H	-1.177778873120	1.706233794580	-0.237396962470
N	-0.229876307838	1.424055704242	0.050977014233
H	-0.261853821392	1.447417695025	1.082333885996
C	-0.000332334560	0.031298074844	-0.369895610484
H	-0.010672473294	0.031988549448	-1.484715381204
C	-1.080737800190	-0.953972208841	0.108094342576
C	1.395267919699	-0.407143744794	0.084944899802
H	2.167496192865	0.300817701312	-0.276632888622
H	1.452554216934	-0.434523437034	1.195171579751
H	1.643141779197	-1.421417211972	-0.287254054418

```
H          -1.099551182434      -1.000534012925       1.218753062979
H          -0.901643167901      -1.982303853895      -0.271160313203
H          -2.087745225685      -0.637161881484      -0.234799819803
```

13.4.4 Species pertinent to reduction of ^{i}PrNO$_2$: Path A, high spin

```
49
6A-1.iPr  Etot(UPBE0-D3(Acetonitrile)/def2TZVP // UPBE-D3(Acetonitrile)/def2SVP/W06) = -2465.00778899 <S2> = 8.7616
Fe         -0.812618198688       0.180596827690      -0.182224775157
O          -2.476209258522       1.118862100878      -0.540990721498
O           0.413567971936       1.659012431520      -0.394474151292
N           0.665277656672      -0.627790572706       1.141553360051
N          -1.961023946865      -1.023026686224       1.156541431644
C          -3.248843649195      -1.204150418594       1.060129861079
H          -3.719045499739      -1.997728061040       1.682131275695
C          -4.152591429000      -0.446651818665       0.229373103120
C          -5.523065729717      -0.825727538634       0.204387464365
H          -5.831933604810      -1.707898803042       0.788451491717
C          -6.463872797656      -0.111091368846      -0.533176141196
H          -7.518337774283      -0.422560448377      -0.545647565011
C          -6.043283095974       1.027616217670      -1.260890886362
H          -6.778674713510       1.604159492009      -1.844237453361
C          -4.710033023167       1.435361082391      -1.250820775310
H          -4.378968227111       2.320707121108      -1.813907741366
C          -3.719122693408       0.713011190800      -0.520436614597
C           1.560228854327       2.017368953126       0.126898860132
C           2.163051973486       3.243574482428      -0.281210226456
H           1.623855854458       3.850829641451      -1.023301206678
C           3.390104273753       3.654918292895       0.238633571032
H           3.826737269116       4.607644207566      -0.100292547413
C           4.078273331383       2.868421466221       1.191914145597
H           5.044545240125       3.202005925877       1.596852156584
C           3.512846736727       1.665397355934       1.609996171911
H           4.033351817777       1.035974299541       2.349935111219
C           2.267456764840       1.211949478060       1.096557322810
C           1.760026684037      -0.060131697386       1.555483484840
H           2.386618145689      -0.594032502105       2.302714406393
C           0.249049092698      -1.921428500022       1.659701546379
H           0.908624565937      -2.282686064354       2.477321212297
H           0.309135418548      -2.654929717266       0.825356065096
C          -1.197939958454      -1.783904778309       2.142836933287
H          -1.650729820983      -2.778638246619       2.345107188324
H          -1.201781694869      -1.211840812041       3.097300347989
O           3.263017211693      -2.600945314806       0.501600675791
N           2.873583868116      -2.273604965107      -0.620223329030
O           1.931465461201      -2.793979306401      -1.212971108102
H          -0.677904942543      -0.794786111227      -1.548127003808
C           3.658355466116      -1.160344124513      -1.327453218360
H           3.962729209131      -0.502232389984      -0.489790777958
C           2.763555510820      -0.420029569309      -2.306214967606
C           4.894564717681      -1.806901060596      -1.954530590946
H           5.494075008726      -2.339545272493      -1.191476761333
H           4.603317313663      -2.520314534962      -2.751226672284
H           5.525040713214      -1.016888182663      -2.406250725649
H           2.466276496958      -1.072396015437      -3.149377735273
H           3.321973271967       0.447699368104      -2.708928454742
H           1.847008806711      -0.044250003158      -1.812252516498
```

```
49
6A-2-ts.iPr  Etot(UPBE0-D3(Acetonitrile)/def2TZVP // UPBE-D3(Acetonitrile)/def2SVP/W06) = -2464.98627923 <S2> = 8.8518
Fe         -0.582085560126       0.488406641826       0.177434734221
O          -2.131047728254       1.042273677405      -0.807844672173
O           0.822361725682       1.676057963931      -0.373275683537
N           0.751206710292      -0.212604553038       1.669708461955
N          -1.924901282124      -0.270708689359       1.643432378348
C          -3.200661184784      -0.467426197948       1.463832121159
H          -3.765758018739      -1.019281834316       2.247340288632
C          -3.979212493676      -0.025062832041       0.330748104818
C          -5.367377998918      -0.330672949482       0.311110768667
H          -5.788411797728      -0.903821029139       1.153095592839
C          -6.190176640173       0.078696724719      -0.736469672552
H          -7.261771518769      -0.167767207447      -0.734032479075
C          -5.626545542165       0.818198021910      -1.802188078237
```

H	-6.266323628000	1.148643203611	-2.635756798127
C	-4.268579214097	1.137100409810	-1.815618787522
H	-3.824714375101	1.711283711800	-2.642535024370
C	-3.401166771968	0.726988866541	-0.760891781733
C	2.096146229957	1.786660440917	-0.084336719397
C	2.914884174309	2.661217604813	-0.857476218448
H	2.431473400590	3.214894743999	-1.676045581523
C	4.275950024433	2.805333623744	-0.590561614726
H	4.877876295151	3.487740029698	-1.211259324007
C	4.891024570482	2.089371586519	0.463295444063
H	5.964687854454	2.210185639784	0.667409732255
C	4.116212343331	1.227959123599	1.236429274879
H	4.576987877965	0.657365651080	2.058999182650
C	2.727556029908	1.048435946318	0.987325648060
C	2.003831846807	0.108001506183	1.811134973613
H	2.593363068232	-0.389004695057	2.612721148646
C	0.133083293642	-1.221916285836	2.515031398058
H	0.725701096293	-1.425419077111	3.432919668276
H	0.086393346420	-2.161406575805	1.919957723078
C	-1.278581413648	-0.741945415869	2.864675146994
H	-1.862577446460	-1.543903068169	3.365523159037
H	-1.203836364909	0.115453082902	3.570233000213
O	1.700566158983	-3.077398338227	0.289092472387
N	1.543914232351	-2.468998893202	-0.801249094349
O	0.398025729155	-2.219981133059	-1.288601427369
H	-0.247286478442	-0.970169041967	-0.680667964191
C	2.751446777699	-1.771527147856	-1.385381656800
H	3.082743321350	-1.094969512548	-0.565764263701
C	2.373066654128	-0.937950996226	-2.600861527191
C	3.842837859809	-2.808977716255	-1.645382419811
H	4.028396034510	-3.406153861266	-0.732910373470
H	3.547810304753	-3.493713054306	-2.466787456434
H	4.783630642514	-2.301725588526	-1.935033680619
H	2.000577920531	-1.575030747597	-3.427372305803
H	3.269251653247	-0.390498156156	-2.954399337769
H	1.592795396808	-0.197613593497	-2.343632155779

49

^{6}A-3.^{i}Pr E_{tot}(UPBE0-D3(Acetonitrile)/def2TZVP // UPBE-D3(Acetonitrile)/def2SVP/W06) = -2465.03863275 $\langle S^2 \rangle$ = 8.7896

Fe	0.290253289669	-0.396413519464	-0.471722432816
O	1.818866397015	0.462483378397	-1.315443291813
O	-1.266327829497	0.238909723934	-1.452950794115
N	-1.030471397652	-1.602007921118	0.627464527171
N	1.645207827088	-1.703268919575	0.446583147644
C	2.947953246525	-1.575727244952	0.487812310277
H	3.537494381506	-2.308066288616	1.083245985552
C	3.710068381782	-0.549297368643	-0.181025937629
C	5.118735053257	-0.516598297140	0.025878286304
H	5.562738951989	-1.271789246950	0.695565507339
C	5.931124394471	0.433225002844	-0.587346143510
H	7.016423991420	0.439639007322	-0.408491835384
C	5.338723523728	1.391211808057	-1.446773472251
H	5.969659639379	2.148721251321	-1.938778056328
C	3.965253726748	1.387457512208	-1.679486709671
H	3.497640998368	2.127655630488	-2.346760375568
C	3.099926560147	0.430414810343	-1.062040029452
C	-2.540340717861	-0.054578665943	-1.430284169265
C	-3.429030269135	0.598353831430	-2.340334885410
H	-2.986308369932	1.325692997923	-3.037841187309
C	-4.796378100297	0.332648505472	-2.343478836878
H	-5.448857083126	0.858844355562	-3.058471978794
C	-5.355798929651	-0.602869285462	-1.438182804766
H	-6.436271330792	-0.808522063768	-1.445870467004
C	-4.517883243109	-1.257248402701	-0.539744069130
H	-4.935613978709	-1.988765133611	0.171667700993
C	-3.115802632823	-1.011192814875	-0.503089197983
C	-2.321509989376	-1.730431634523	0.463212515002
H	-2.876881215308	-2.436849609993	1.119789403900
C	-0.349725521688	-2.351458952872	1.673532749560
H	-0.950730111436	-3.214071714231	2.038167048400
H	-0.183569302644	-1.665254657594	2.534030728474
C	1.002414890717	-2.821526371802	1.126171576874
H	1.636223180808	-3.242354045574	1.938030331469
H	0.825498369216	-3.631783630496	0.383471887393
O	-0.619482038474	0.797902513069	3.251445326581
N	-0.455752272992	1.495379123801	2.238271042708
O	0.616552949467	1.003054203887	1.301481966465

H	1.236978661104	0.570743562197	1.930146993934
C	-1.609985678407	1.968346157992	1.404908256437
H	-1.979997622011	1.062377898281	0.875952091412
C	-1.138392350855	3.000848678189	0.386406626048
C	-2.692780876952	2.485350471802	2.346287929645
H	-2.999903435065	1.706737744528	3.068916717217
H	-2.342558097780	3.375203728941	2.907534388178
H	-3.576007114440	2.777335118127	1.746292017772
H	-0.701633628902	3.884845796869	0.892825564487
H	-2.007003010379	3.333624874999	-0.214353545756
H	-0.393103131160	2.571704957747	-0.307326311976

49

^{6}A-4-ts.^{i}Pr E_{tot}(UPBE0-D3(Acetonitrile)/def2TZVP // UPBE-D3(Acetonitrile)/def2SVP/W06) = -2465.03461346 $\langle S^2\rangle$ = 8.7900

Fe	0.285907662349	-0.397032261785	-0.417437741834
O	1.803389396634	0.439920354114	-1.296703408413
O	-1.277305526116	0.198949465240	-1.415144578583
N	-1.028874219176	-1.633734990937	0.671639425386
N	1.647740106185	-1.730505373154	0.468607215763
C	2.949399010317	-1.605280431504	0.491819143589
H	3.546434937597	-2.341714256307	1.074600467104
C	3.704429948356	-0.575167666311	-0.180841406491
C	5.114600764391	-0.544668098052	0.012937259679
H	5.564583393007	-1.304113194032	0.673633378130
C	5.921480954475	0.409156905399	-0.601730484957
H	7.008378247772	0.414229459908	-0.433204193160
C	5.320952311118	1.372638758325	-1.448550644531
H	5.947095213285	2.133456399303	-1.941486116979
C	3.945217348089	1.370481461523	-1.668704533601
H	3.471745228194	2.114505459081	-2.327392657069
C	3.086596622676	0.408871058653	-1.050370371648
C	-2.549025518201	-0.103740963430	-1.390922022484
C	-3.441823540970	0.537690281275	-2.304994272342
H	-3.004582363950	1.265255296954	-3.005643976329
C	-4.806886645200	0.260656303115	-2.307465554396
H	-5.462989386198	0.778115337516	-3.025504185040
C	-5.359761799312	-0.674836113805	-1.398333128433
H	-6.438559847054	-0.888840123843	-1.405726856993
C	-4.517093858041	-1.318489355116	-0.496492613732
H	-4.929140211296	-2.050262847721	0.217898957027
C	-3.117401009925	-1.060652063336	-0.460745161009
C	-2.317445737029	-1.770596424714	0.507861056559
H	-2.868012088906	-2.479587128043	1.165950651422
C	-0.337494912047	-2.378624750507	1.713024902862
H	-0.933140386289	-3.240517971603	2.087611021432
H	-0.162789924566	-1.690693285285	2.570471408001
C	1.009365257837	-2.849338934777	1.151938704705
H	1.650374480515	-3.273592046120	1.956036507462
H	0.823008600694	-3.656653670642	0.408484465705
O	-0.806558134404	0.944008888900	3.214209422808
N	-0.490755522529	1.610849237084	2.218472281435
O	0.587421188902	0.912075825734	1.327477448630
H	1.121679908200	0.467656168021	2.020626625365
C	-1.548352097517	2.114386380562	1.273076145183
H	-1.961579035815	1.207983816693	0.778748414218
C	-0.926450442208	3.039326048104	0.232924154781
C	-2.639274703849	2.782225433842	2.105350112513
H	-3.061349238951	2.079970703476	2.848125863557
H	-2.246315679682	3.671814956120	2.638439489688
H	-3.452277544876	3.112709769414	1.430403034045
H	-0.455742633802	3.917248349420	0.719397439754
H	-1.720237868193	3.400690392960	-0.449484099979
H	-0.168722690002	2.515051862791	-0.377035600762

49

^{6}A-5.^{i}Pr E_{tot}(UPBE0-D3(Acetonitrile)/def2TZVP // UPBE-D3(Acetonitrile)/def2SVP/W06) = -2465.06428206 $\langle S^2\rangle$ = 8.7625

Fe	0.643530986990	0.060267386287	0.074930961466
O	2.212657304943	1.135817810079	0.495700294159
O	-0.728257958470	1.415288525099	0.346968690529
N	-0.728898313500	-0.790391517253	-1.314651384422
N	1.929472004080	-0.962064093070	-1.307192665544
C	3.227781380692	-1.014726475107	-1.213141002284
H	3.778549482691	-1.733887513887	-1.859432702550
C	4.047069115233	-0.195432142219	-0.352824434979
C	5.451958238155	-0.418078286382	-0.348106571718
H	5.853321922379	-1.232885241944	-0.972546630954

C	6.312733386165	0.364770134170	0.416811548523
H	7.395802764618	0.174779321628	0.410358033616
C	5.772825888401	1.414859660128	1.197997184256
H	6.443736132709	2.044489928896	1.803835907609
C	4.402450884590	1.667598717097	1.212690637685
H	3.978024417796	2.483084983430	1.817296805766
C	3.491777370295	0.872149756115	0.452447524489
C	-1.896896831448	1.682705105186	-0.179002573422
C	-2.621733224654	2.826219782125	0.272294976774
H	-2.148427966182	3.452628658376	1.043049288151
C	-3.884624181290	3.131296439985	-0.233551306374
H	-4.415543179765	4.020177120605	0.142484637167
C	-4.493334776918	2.314344084662	-1.215305318304
H	-5.492162977878	2.559582198551	-1.604254833484
C	-3.807368728963	1.194266801016	-1.679925417210
H	-4.265095501283	0.540479903552	-2.439822733600
C	-2.517638221069	0.855121882250	-1.189819468669
C	-1.891067340434	-0.346930295344	-1.687730210245
H	-2.479534055704	-0.940073388658	-2.422233847706
C	-0.190870069453	-2.043279652689	-1.811979415070
H	-0.814080562405	-2.489186767131	-2.617212092640
H	-0.163186322118	-2.748235188973	-0.952379111760
C	1.235894115777	-1.775088141212	-2.302575333646
H	1.773092334185	-2.724563577012	-2.516084711884
H	1.184977902073	-1.195622095518	-3.251289981450
O	-4.080848068919	-2.113629572556	0.037787974457
N	-3.007038537386	-2.224337930250	0.598328214123
O	0.503873323279	-1.239045468205	1.401095033866
H	1.341673850569	-1.680108540631	1.643605866936
C	-2.783425847489	-1.287642889183	1.743560811581
H	-1.904277118610	-0.706598613257	1.373221785198
C	-2.267783907571	-2.140850095411	2.909621282835
C	-3.959328659518	-0.379585327410	2.061195886083
H	-4.280500679432	0.188000747372	1.166176652896
H	-4.828469719156	-0.967605050039	2.421533906900
H	-3.676222605483	0.343636244202	2.850401400308
H	-3.065688122599	-2.806593774698	3.298350158880
H	-1.929379119720	-1.480625421652	3.731505819142
H	-1.403070960449	-2.748035894658	2.581473490314

49

^{6}A-4a-ts.^{i}Pr E_{tot}(UPBE0-D3(Acetonitrile)/def2TZVP // UPBE-D3(Acetonitrile)/def2SVP/W06) = -2465.01673295 $\langle S^2 \rangle$ = 8.7809

Fe	0.037154922591	0.498652111494	-0.001832984997
O	1.680445258827	1.086895290717	0.830302531232
O	-1.423894030127	1.477114955643	0.756122069046
N	-1.149799611237	-0.282753484769	-1.459317839015
N	1.607743638815	-0.584266773960	-1.492314395101
C	2.835439099724	-0.093109786847	-1.614352291836
H	3.298787525300	-0.123700777134	-2.621062993636
C	3.589993975299	0.522662540131	-0.570700612779
C	4.997271179774	0.687625012076	-0.764789804343
H	5.447181565411	0.270623548729	-1.680156753110
C	5.794446578639	1.328105362502	0.172844689600
H	6.878532966703	1.422058169115	0.014199992224
C	5.189357943491	1.869566092574	1.338034707539
H	5.813123826511	2.380366173696	2.088686290009
C	3.817181037541	1.777811816430	1.539609029416
H	3.339919451523	2.216027887492	2.428988169142
C	2.960765228315	1.104808608677	0.609274008311
C	-2.670512296316	1.592811738899	0.367342733714
C	-3.559823750508	2.420104011337	1.117718927291
H	-3.147985783830	2.925489417327	2.004375173423
C	-4.891629921000	2.584791500717	0.744088525090
H	-5.547242085219	3.233397817848	1.346500071294
C	-5.408744334654	1.928453001187	-0.399878000860
H	-6.461133054040	2.061583979550	-0.690479857532
C	-4.568194703999	1.110454171354	-1.148431413431
H	-4.956720788778	0.588330955709	-2.038152381826
C	-3.200039424332	0.914989631933	-0.798833063963
C	-2.417537891427	0.025959924334	-1.619238151860
H	-2.964690966767	-0.455051983181	-2.458072188881
C	-0.575999857623	-1.319092712414	-2.317257554472
H	-1.181629510943	-1.473037831937	-3.236033741413
H	-0.571148304416	-2.279971492365	-1.752450259772
C	0.863537114899	-0.981164619459	-2.695084834462
H	1.337362825241	-1.863897206848	-3.176240017439
H	0.886942292761	-0.144056105579	-3.422881746757

O	0.333118153078	-3.503245122504	-0.176659065804
N	0.120684054154	-2.666456152434	0.750568849023
O	0.823124865955	-1.473356645661	0.712923133021
H	1.310160573515	-1.188389347227	-0.447089070104
C	-1.229210849173	-2.553838845227	1.381082300431
H	-1.860434652050	-1.979860770805	0.658125103694
C	-1.151104180906	-1.776348844931	2.694574325327
C	-1.812428269525	-3.953544002743	1.554302327883
H	-1.842959336641	-4.493512498455	0.590423653067
H	-1.204246294771	-4.545400138900	2.268875354829
H	-2.842114221299	-3.877490489143	1.954343798809
H	-0.458399484755	-2.273432819850	3.403744688771
H	-2.156432553857	-1.740421789323	3.158596307052
H	-0.812252600272	-0.735810148160	2.537517294222

49

^{6}A-5a.^{i}Pr E_{tot}(UPBE0-D3(Acetonitrile)/def2TZVP // UPBE-D3(Acetonitrile)/def2SVP/W06) = -2465.02621120 $\langle S^2 \rangle$ = 8.7821

Fe	-0.093957969289	0.028415448204	-0.460831191911
O	1.707169435041	-0.424040409491	-1.091411085706
O	-1.350789007596	-1.220524346426	-1.185237602064
N	-1.105780485079	0.110563184798	1.326458418739
N	1.667712809109	0.584920356893	1.643860257091
C	2.666329453345	-0.285993551937	1.692373450168
H	2.893039804567	-0.687340815322	2.696833832090
C	3.421823192142	-0.784830839967	0.592734500164
C	4.691936065993	-1.381365309648	0.873831624762
H	5.044867840978	-1.388232718664	1.917645752379
C	5.486229260829	-1.908945888182	-0.133165871084
H	6.471485870580	-2.338266242310	0.099307318669
C	5.007007737733	-1.892023536448	-1.470759999914
H	5.632612611132	-2.305351409109	-2.277835379231
C	3.751169390066	-1.380897584993	-1.775046467975
H	3.362549903983	-1.395149120653	-2.804513002055
C	2.898030091539	-0.819472394085	-0.768659840308
C	-2.382096680993	-1.814339565480	-0.639527452608
C	-3.136604534734	-2.750413700061	-1.411341172366
H	-2.810638810507	-2.924585379992	-2.448078668714
C	-4.234783855327	-3.420450968940	-0.877239472327
H	-4.788219857275	-4.138300512870	-1.503354019862
C	-4.644853805672	-3.189192966563	0.458999042225
H	-5.513314128833	-3.720026236827	0.875749286764
C	-3.934652379259	-2.278453175430	1.234879851109
H	-4.243536212152	-2.082432647127	2.274931586877
C	-2.804190338056	-1.573876847225	0.726680659488
C	-2.151291895344	-0.635771385433	1.604219461445
H	-2.604868092681	-0.534269446694	2.613987807878
C	-0.660089374290	1.073047274529	2.329508868717
H	-1.313959655637	1.057553964173	3.228509353353
H	-0.713505662393	2.095075715755	1.892489738056
C	0.789269055997	0.843087600501	2.785212434831
H	1.131509363743	1.748496374062	3.330804598823
H	0.857263509544	-0.022811386653	3.471908608325
O	0.093691168501	3.690203915992	0.784560939957
N	-0.117254700328	3.105877045039	-0.326044827933
O	0.557534359586	1.940003701117	-0.565889772122
H	1.504772387953	1.200501476481	0.803270723607
C	-1.459744590333	3.187936564735	-0.972392235601
H	-2.077622822473	2.383130560738	-0.496975797732
C	-1.349215085516	2.906220179821	-2.470972528853
C	-2.090882760290	4.544388418925	-0.672945073610
H	-2.167406389883	4.718423382898	0.415673948157
H	-1.486955231314	5.363063478845	-1.115336173810
H	-3.105956602016	4.585201254349	-1.114301211529
H	-0.703446296484	3.662974086079	-2.961715716748
H	-2.354174257958	2.952897496399	-2.934844385200
H	-0.925514768506	1.904065531776	-2.666982655788

48

^{6}A-6.^{i}Pr E_{tot}(UPBE0-D3(Acetonitrile)/def2TZVP // UPBE-D3(Acetonitrile)/def2SVP/W06) = -2389.82154100 $\langle S^2 \rangle$ = 8.7626

Fe	0.722747433046	0.084536200521	0.176372893446
O	2.337655959593	1.093477350638	0.553202799056
O	-0.569982864085	1.513184050941	0.346873877445
N	-0.694792875204	-0.811032930256	-1.148780494749
N	1.945265794936	-1.093541629478	-1.121761125055
C	3.239538180056	-1.208584798828	-1.011999126601
H	3.753796546892	-1.991504564222	-1.612203450638

C	4.098292162866	-0.389536117920	-0.191677302422
C	5.487098638278	-0.693821036717	-0.157066945154
H	5.844999054312	-1.566887755575	-0.726629575695
C	6.385678173523	0.081833359392	0.571551926940
H	7.455418906332	-0.171813190279	0.590956842244
C	5.902008303339	1.206800645564	1.281109006361
H	6.603347398765	1.831353401471	1.856907582698
C	4.548568024922	1.541059072779	1.262421858858
H	4.168154134758	2.415299899383	1.811475842024
C	3.600736734120	0.755521511925	0.540286593709
C	-1.727557406110	1.803363256046	-0.191534779516
C	-2.390402195558	3.010441266666	0.180313278120
H	-1.885607329115	3.661179381744	0.909651581726
C	-3.631338134371	3.350976447853	-0.357350706629
H	-4.113862005895	4.290956354080	-0.046264945535
C	-4.275898332717	2.508881559525	-1.293485690104
H	-5.253653780938	2.786398317926	-1.712892188485
C	-3.652519898586	1.322716656703	-1.675609226792
H	-4.138881561017	0.650283641008	-2.400806785323
C	-2.390447896125	0.941629573844	-1.144453680283
C	-1.825166959155	-0.320013996113	-1.562172859689
H	-2.432741747354	-0.913726362930	-2.280757243478
C	-0.217137195756	-2.101852880348	-1.614173207080
H	-0.852910247040	-2.524226930635	-2.422133791432
H	-0.248562200724	-2.805919974807	-0.753062780893
C	1.227011705695	-1.917256730431	-2.091356414283
H	1.724132162676	-2.897247729844	-2.257828870516
H	1.215115128104	-1.376140061825	-3.063579784705
N	-3.257570709893	-2.509336153709	0.628276853718
O	-2.156705893730	-2.835863880194	1.026914354925
H	0.601735028760	-0.893576607856	1.543054988294
C	-3.842443982544	-1.296941929989	1.296287167610
H	-4.064530880227	-0.645430640765	0.417232663275
C	-2.913416455917	-0.594387294770	2.270819694424
C	-5.188602293873	-1.750098161920	1.882813526914
H	-5.777709559502	-2.316545649323	1.134612272111
H	-5.026510854814	-2.400326623866	2.766696203850
H	-5.777440775615	-0.867749362094	2.199766320201
H	-2.676121214624	-1.248094538959	3.134407899160
H	-3.394396397016	0.327822101827	2.651251561438
H	-1.957263181515	-0.315431914539	1.786036854973

48

^{6}A-7-ts.^{i}Pr E_{tot}(UPBE0-D3(Acetonitrile)/def2TZVP // UPBE-D3(Acetonitrile)/def2SVP/W06) = -2389.80934579 $\langle S^2 \rangle$ = 8.7969

Fe	0.574139346201	-0.234039709214	0.224941259966
O	2.110183377459	-1.121899873669	-0.494327433579
O	-0.745738688794	-1.635714932344	0.314302278525
N	-0.705092541354	0.915066341003	1.390710600423
N	1.957533064874	0.966024348118	1.332835338539
C	3.237157124887	1.053337062941	1.107043007600
H	3.819525893907	1.832572629275	1.646934689543
C	3.998177334523	0.216173959014	0.208659434858
C	5.394871277716	0.450784203947	0.090198997087
H	5.839682772873	1.268101267226	0.680807479704
C	6.196955458487	-0.324546902047	-0.745281100250
H	7.275829504147	-0.127221875894	-0.824056390705
C	5.603015433931	-1.370391912496	-1.489432363656
H	6.226109713533	-1.990884415045	-2.152808066008
C	4.235545187578	-1.629838561758	-1.397252094226
H	3.767564046033	-2.441225044298	-1.974412284810
C	3.389772224653	-0.850272297676	-0.554515010372
C	-2.031720140020	-1.617489134429	0.581456734927
C	-2.822043337929	-2.765004729291	0.280465453637
H	-2.308009313471	-3.630646135648	-0.163363053890
C	-4.195317097590	-2.786022963504	0.526170118722
H	-4.775235768418	-3.687924347663	0.274165537345
C	-4.848213678599	-1.666065698866	1.090503953493
H	-5.930890077058	-1.688917086683	1.281587571076
C	-4.098483102264	-0.531805935423	1.399913098209
H	-4.589350708465	0.352783535716	1.837087289058
C	-2.699806125344	-0.474144752950	1.160259360694
C	-1.995141324563	0.742577017341	1.500039487844
H	-2.610150373202	1.561626925795	1.927326090710
C	-0.091595014588	2.177814118534	1.780986758717
H	-0.686144546166	2.710307980450	2.553531703305
H	-0.032608553426	2.823788652846	0.878009207165
C	1.316487239197	1.860104220335	2.291542187345

H	1.906408510288	2.786439317965	2.461465914223
H	1.233715811521	1.325061919465	3.263815786128
N	-2.312545399872	1.992051984166	-1.023947290849
O	-1.158304912384	2.131878734932	-1.437580805872
H	0.133965881233	0.792576594634	-1.009735032768
C	-3.192771044660	1.118177604588	-1.882802479909
H	-3.804068493013	0.557890884227	-1.141380525918
C	-2.421395388649	0.146951429822	-2.768048404639
C	-4.121857970914	2.069215967265	-2.650207621802
H	-4.619362476190	2.781565236285	-1.961484019327
H	-3.544752775377	2.653685175302	-3.396581718126
H	-4.905885413552	1.495150455999	-3.183431423634
H	-1.835026238864	0.683869961187	-3.540050805904
H	-3.125355363235	-0.541455248018	-3.275856382931
H	-1.713791445223	-0.455540260018	-2.164584282388

48

^{6}**A-8.^{i}Pr** E_{tot}(UPBE0-D3(Acetonitrile)/def2TZVP // UPBE-D3(Acetonitrile)/def2SVP/W06) = -2389.90273056 $\langle S^2 \rangle$ = 8.7908

Fe	0.106027896564	-0.127273561434	-0.014867969499
O	1.772194297568	0.313085407085	-1.049881439234
O	-1.337606606194	-0.462891508289	-1.289596246506
N	-1.233515368185	-0.787900980863	1.473418997790
N	1.436722450471	-1.036517750029	1.391846466007
C	2.717885720918	-1.238552281970	1.263882365885
H	3.248780137324	-1.805323364331	2.061246527502
C	3.546373280321	-0.816679409260	0.156513481956
C	4.924487127701	-1.167976462403	0.183038769637
H	5.301101948199	-1.736694465602	1.049100777676
C	5.791454664803	-0.814423478844	-0.849020175593
H	6.853429281221	-1.097393496151	-0.808158259842
C	5.284354655539	-0.085815119918	-1.950032505921
H	5.957473746373	0.200795954477	-2.773653874850
C	3.938495526467	0.276695403916	-2.007258896062
H	3.536276221440	0.845432517617	-2.859365659942
C	3.028874025481	-0.070024703359	-0.965632435010
C	-2.604748628045	-0.751058150349	-1.167895949368
C	-3.434513624988	-0.779687161132	-2.333053751578
H	-2.953794138047	-0.554676984614	-3.297241545420
C	-4.794445270885	-1.070359078576	-2.254772113157
H	-5.399986344233	-1.074681950803	-3.175240827923
C	-5.406900485348	-1.358672819646	-1.010499162146
H	-6.481102102818	-1.588395298775	-0.957075315360
C	-4.626311017882	-1.348057969665	0.142359561026
H	-5.082705931570	-1.574005864771	1.120252677286
C	-3.235889281701	-1.048111147632	0.104029010437
C	-2.498599463692	-1.073926604488	1.344914098204
H	-3.077476701109	-1.361226931080	2.251316475016
C	-0.570272637202	-0.870988096198	2.763184067790
H	-1.191780501460	-1.378443275009	3.533454754878
H	-0.355485696318	0.162146974091	3.115908227810
C	0.750454922719	-1.614441478194	2.548578652776
H	1.381364289519	-1.581673109171	3.462901610402
H	0.526845436950	-2.683410759855	2.333036365323
N	-0.065455819564	1.802341597369	0.374832818484
O	0.978536968450	2.650890157271	0.031254839138
H	1.574131099431	2.017953557877	-0.455962705628
C	-1.270857681811	2.566190869792	0.661000709906
H	-2.002310257912	1.792752525626	0.980377541739
C	-1.808508670360	3.247020724678	-0.610034150825
C	-1.065022099352	3.553969599968	1.820785183390
H	-0.705342643887	3.024792330305	2.726259484389
H	-0.315929453017	4.323952707115	1.548631483264
H	-2.016100621775	4.065772011418	2.069994533521
H	-1.084033604346	4.000231568637	-0.979060812036
H	-2.773541987744	3.754292946270	-0.408932773936
H	-1.963659980621	2.492166669824	-1.406647248145

47

^{6}**A-9.^{i}Pr** E_{tot}(UPBE0-D3(Acetonitrile)/def2TZVP // UPBE-D3(Acetonitrile)/def2SVP/W06) = -2314.76640529 $\langle S^2 \rangle$ = 8.7760

Fe	0.145267291269	-0.030089253799	0.042573564778
O	1.664139732915	-1.259844102581	0.222448854060
O	-1.333907852330	-1.251178011267	0.400568524777
N	-1.212592144886	1.511949779359	0.640739944891
N	1.451645287618	1.427930421480	0.924735082165
C	2.752248704993	1.361583828913	0.955749877462
H	3.322835399101	2.255261928516	1.295388364556

C	3.549291710743	0.208602341409	0.609401732751
C	4.965628547560	0.332179110342	0.651926515981
H	5.395378302675	1.314116930441	0.910183999879
C	5.803722646595	-0.746582994284	0.380506511095
H	6.896617957018	-0.628885773575	0.414602711535
C	5.225223116624	-2.000486155787	0.066821336962
H	5.876193227950	-2.863642762465	-0.145793363141
C	3.841967216925	-2.161821008939	0.024003219279
H	3.388025927584	-3.134716686439	-0.218237300760
C	2.952908666602	-1.070811268373	0.280254542258
C	-2.609458688290	-1.109471677076	0.646961455045
C	-3.448817483679	-2.265724640631	0.685806993734
H	-2.971438674261	-3.240232305110	0.502536498437
C	-4.815919221616	-2.166700610309	0.938341569238
H	-5.429403569243	-3.081707386359	0.954733127480
C	-5.423775613952	-0.910216760900	1.172622876348
H	-6.503119154115	-0.840327018670	1.371462954865
C	-4.631688599487	0.235706284630	1.147416929341
H	-5.084573641731	1.224255249840	1.329347582168
C	-3.235405215906	0.174383670510	0.887858845942
C	-2.484916889872	1.410151751501	0.885343315147
H	-3.065334989162	2.334024410534	1.107966084796
C	-0.540114837439	2.799278141234	0.654868281038
H	-1.160512200410	3.603356179043	1.108221585991
H	-0.314485940093	3.079397621388	-0.397780336627
C	0.777801812163	2.625526314148	1.415917281445
H	1.415054369649	3.532733038287	1.328747631660
H	0.548814387285	2.478142976061	2.495379341410
N	0.139221727611	0.421764407099	-1.811878025964
H	1.004443721775	0.457922094243	-2.372546732830
C	-1.041697398966	0.462620593340	-2.655997651664
H	-1.919073743177	0.578080617545	-1.977925256700
C	-1.226368006825	-0.861671552248	-3.421651858160
C	-1.017939630266	1.671345678096	-3.605685629369
H	-0.919362764852	2.617095164969	-3.035393134809
H	-0.155899458140	1.600965984947	-4.303871782299
H	-1.943940614705	1.726355362070	-4.214710580366
H	-0.374688188743	-1.033988245016	-4.114041512061
H	-2.161546357153	-0.858326714135	-4.020014274687
H	-1.266857722777	-1.708241685549	-2.707283194315

13.4.5 Species pertinent to reduction of ^{i}PrNO$_2$: Path A, intermediate spin

49

^{4}A-1.^{i}Pr E_{tot}(UPBE0-D3(Acetonitrile)/def2TZVP // UPBE-D3(Acetonitrile)/def2SVP/W06) = -2464.99823895 $\langle S^2 \rangle$ = 3.8793

Fe	0.712494841380	-0.336056041347	0.154605350397
O	2.198340634086	-0.946793939984	-0.762740390236
O	-0.484063669881	-1.811252238230	-0.274141884570
N	-0.645829054316	0.422059368002	1.309353477915
N	1.963861566433	0.544938784878	1.538991486949
C	3.243475506794	0.759239468737	1.382482531571
H	3.769536225070	1.424463081891	2.100811505018
C	4.041617091425	0.209569136302	0.315100332748
C	5.434186014958	0.499406359312	0.280510478927
H	5.854621392225	1.131256555873	1.079690516773
C	6.259212781363	0.003630405197	-0.725588290131
H	7.333544747165	0.238762433098	-0.732072697583
C	5.696869887413	-0.805845381881	-1.742129405472
H	6.339560805614	-1.204062811337	-2.543033712571
C	4.335469423606	-1.103267389577	-1.746792985822
H	3.889157754046	-1.729311692844	-2.533826873097
C	3.467920256471	-0.611909053015	-0.726844297189
C	-1.721933025137	-2.052052716782	0.064537647362
C	-2.389397939348	-3.209932737328	-0.439141606413
H	-1.813074021458	-3.884102500247	-1.090344991903
C	-3.725007365548	-3.463520209356	-0.133974323739
H	-4.213548221363	-4.358805143852	-0.550168484382
C	-4.462746070144	-2.587389274857	0.700791413369
H	-5.515416333544	-2.800073358382	0.937381349290
C	-3.835971229934	-1.459221599744	1.221857339038
H	-4.389885953435	-0.770119493198	1.879904299020
C	-2.475576225287	-1.163822208115	0.922838397642
C	-1.871501461652	-0.002791790831	1.512947494115
H	-2.503587772038	0.588093515749	2.205331795970

C	-0.144248596353	1.577715930132	2.053154607293
H	-0.845280839260	1.871858402949	2.861746922322
H	-0.044767902960	2.428549545018	1.346025533871
C	1.225147456879	1.187034588727	2.618201433366
H	1.759683624358	2.069507193448	3.031408507114
H	1.081034429293	0.454246890106	3.442773414390
O	-2.806350525231	2.833996470834	0.497949148291
N	-2.341060705984	2.488576592650	-0.590071945697
O	-1.267057717036	2.875572962792	-1.045175813211
H	0.494518710953	0.872136602659	-0.778032896870
C	-3.198470969971	1.538229273755	-1.436879061158
H	-3.690783869467	0.903766779791	-0.673693765624
C	-2.323356035691	0.702971387650	-2.355378781345
C	-4.244569711438	2.391695851625	-2.155739347538
H	-4.838979107398	2.983209364942	-1.433141723608
H	-3.761069360864	3.079740438688	-2.877876986622
H	-4.932094182295	1.726887902090	-2.713520322882
H	-1.839731666672	1.330729630244	-3.128150267777
H	-2.957754774165	-0.053290088308	-2.857856661609
H	-1.533020708556	0.174364324946	-1.787854645651

49

^{4}**A-2-ts.^{i}Pr** E_{tot}(UPBE0-D3(Acetonitrile)/def2TZVP // UPBE-D3(Acetonitrile)/def2SVP/W06) = -2464.97132184 $\langle S^2 \rangle$ = 3.8761

Fe	0.538573299527	-0.614458752658	0.365280507025
O	1.877941086688	-0.921200431455	-0.859263066534
O	-0.808604793835	-1.553467497219	-0.585797129886
N	-0.723025905070	-0.145659886360	1.735017561052
N	1.884101123700	-0.139077016203	1.741907005336
C	3.153930971782	0.118980482465	1.535471050496
H	3.749403401470	0.536672607543	2.373102245183
C	3.839483218296	-0.085216563463	0.288804344226
C	5.225159792707	0.226010402173	0.193674127369
H	5.736892208956	0.613987705688	1.089122636294
C	5.929466950808	0.046733979979	-0.993571322696
H	7.000284155966	0.291518781270	-1.047761456305
C	5.251969081701	-0.453277436883	-2.132434976167
H	5.801308359601	-0.597871319906	-3.075915450353
C	3.894322499756	-0.763951170732	-2.077507334659
H	3.358413659410	-1.151568047405	-2.956604973315
C	3.151060869869	-0.592797860889	-0.874191629873
C	-2.100048759194	-1.658486253372	-0.397423833623
C	-2.901818994456	-2.352149389339	-1.351254549444
H	-2.391675327722	-2.795174025598	-2.219445090885
C	-4.282226885443	-2.451349494963	-1.191462380294
H	-4.876400382849	-2.986722017167	-1.948697405534
C	-4.931143963875	-1.871768629748	-0.072046597818
H	-6.021320943974	-1.958546406751	0.043375866744
C	-4.174074684656	-1.191767515863	0.876377174082
H	-4.661049386975	-0.733603598992	1.752114320972
C	-2.761655034503	-1.065247771901	0.741931784138
C	-2.022541874016	-0.342165065815	1.734901095761
H	-2.602827253231	0.098990738742	2.569044154555
C	-0.115239412161	0.676436028889	2.783477235993
H	-0.736629150003	0.678627092051	3.703006252049
H	-0.057399369225	1.716413612733	2.394643022762
C	1.278445716044	0.117109792475	3.047233524795
H	1.889380672611	0.814543097511	3.658311590938
H	1.197361336206	-0.845202946233	3.598340422829
O	-1.795356673848	2.848207496178	0.868897846801
N	-1.383858998418	2.469867900603	-0.254370340497
O	-0.150953970950	2.279872458183	-0.508187262122
H	0.309395867778	0.889258753379	-0.122495982934
C	-2.416043167124	2.009963486589	-1.257336610073
H	-2.981346502165	1.229431010145	-0.699521615793
C	-1.757512099183	1.386866842374	-2.479521668345
C	-3.354608369863	3.175885267307	-1.569110454796
H	-3.756200585203	3.606280990594	-0.632422561699
H	-2.821399215199	3.971092387278	-2.129144113197
H	-4.201684582440	2.821129886702	-2.187820008469
H	-1.168005265210	2.135287835206	-3.044885391048
H	-2.546378452699	0.984070585624	-3.145232239274
H	-1.089302897670	0.555242879194	-2.187543967892

49

^{4}**A-3.^{i}Pr** E_{tot}(UPBE0-D3(Acetonitrile)/def2TZVP // UPBE-D3(Acetonitrile)/def2SVP/W06) = -2465.02906338 $\langle S^2 \rangle$ = 3.7957

Fe	0.312121161266	-0.182378897559	0.706538934577

O	1.604699996831	-1.375928227085	-0.014205061342
O	-0.990788524502	-1.467810439758	0.194802363447
N	-0.958698500063	1.000684745087	1.444389285085
N	1.604826187056	1.006362733363	1.406972877847
C	2.907224731797	0.966044492632	1.202215494453
H	3.530813969523	1.755105235212	1.668254622301
C	3.588395281190	-0.032283010991	0.430949681375
C	4.998334129021	0.085601714882	0.247325186998
H	5.508890769143	0.958673819688	0.685882103293
C	5.725451817973	-0.864583110658	-0.460327964713
H	6.811643628442	-0.752044548068	-0.592451491066
C	5.047789910395	-1.985038491240	-1.007148937205
H	5.613802496655	-2.745451431064	-1.568527464383
C	3.674609297168	-2.136857536045	-0.842690327708
H	3.140610452864	-3.002992743197	-1.262596665381
C	2.890342729174	-1.174249706237	-0.130224336908
C	-2.278094727173	-1.445581231979	0.427679611717
C	-3.073939139675	-2.550915725190	-0.009643483926
H	-2.549088591642	-3.375354298870	-0.515814711236
C	-4.451288722494	-2.582279209778	0.187057381104
H	-5.030009421577	-3.449606965732	-0.168482041840
C	-5.116703951050	-1.510589036904	0.836552304017
H	-6.205819621949	-1.541700801848	0.987380234059
C	-4.374459533745	-0.421077957373	1.277631866522
H	-4.874799559662	0.421979446366	1.781604095867
C	-2.961800295991	-0.352257434032	1.091860958145
C	-2.258614083978	0.809365983536	1.553580718693
H	-2.864237122442	1.612092517148	2.019464430097
C	-0.379307596001	2.293827160678	1.830647504807
H	-0.987229899275	2.806743776290	2.605387900510
H	-0.356608912669	2.938074273909	0.923278812943
C	1.038907411803	2.017008151142	2.311694895741
H	1.655582576360	2.940070514202	2.335895459291
H	1.013958664583	1.596174080792	3.340986305298
O	-0.597688359624	3.013057019053	-1.540987566838
N	-0.501386817303	1.846607636024	-1.964340443793
O	0.584416960801	1.040106503764	-1.376617273894
H	1.238518425311	1.736745706670	-1.140269467749
C	-1.698094970558	0.971898062823	-2.172297649502
H	-2.059848869497	0.721155463253	-1.149813892867
C	-1.306153223384	-0.309194141952	-2.901210757006
C	-2.761925238444	1.790680503730	-2.896927676761
H	-3.003384271496	2.714918370982	-2.339946694157
H	-2.425230475908	2.066627018139	-3.916762307957
H	-3.682102836663	1.181961171772	-2.986169243389
H	-0.862020928927	-0.082214910043	-3.891273975438
H	-2.215543978640	-0.921612197514	-3.057722642397
H	-0.595396317501	-0.905593672938	-2.301754208833

48

^{4}**A-8.^{i}Pr** E_{tot}(UPBE0-D3(Acetonitrile)/def2TZVP // UPBE-D3(Acetonitrile)/def2SVP/W06) = -2389.88069977 $\langle S^2 \rangle$ = 3.8203

Fe	0.161067678815	0.054803574238	0.381870632418
O	1.527472315308	-0.998991647826	-0.475874874865
O	-1.081019978859	-1.403251954943	0.172937720653
N	-1.129591982687	1.156287085961	1.210521253999
N	1.427368596501	1.056924095985	1.394333417651
C	2.729169465366	0.885774193641	1.438874565003
H	3.316311868306	1.535220876029	2.118812972450
C	3.470100940836	-0.088244121503	0.681135748326
C	4.882884810151	-0.151613020784	0.847266014317
H	5.355710911801	0.544930826199	1.558488378317
C	5.663256142416	-1.061358561913	0.137631305381
H	6.752963664459	-1.093261521103	0.283433583807
C	5.035695629210	-1.941492167482	-0.774593717538
H	5.639892665285	-2.665478463846	-1.343636911651
C	3.653444902770	-1.904003348895	-0.961876050295
H	3.155411768409	-2.585780083955	-1.667942765320
C	2.831163887669	-0.990211096950	-0.244639893372
C	-2.372341694690	-1.390574587088	0.352125542846
C	-3.135669510817	-2.551794406767	0.009116061935
H	-2.584133124537	-3.411251373590	-0.401399143334
C	-4.518925567315	-2.588904369327	0.164395705992
H	-5.072091945772	-3.494963186918	-0.130409447800
C	-5.222014859389	-1.478269547653	0.695919758100
H	-6.314552018672	-1.516353360567	0.816763570090
C	-4.508743294835	-0.342853802258	1.067633618526
H	-5.035840526801	0.526496031478	1.493607213528

C	-3.095631968457	-0.261842525203	0.907534520604
C	-2.415126272537	0.920028069294	1.355325129962
H	-3.024565843613	1.686106697226	1.874847607597
C	-0.542728728010	2.405803824250	1.702389358901
H	-1.198351211428	2.910540279831	2.442654836089
H	-0.403137717039	3.092245945258	0.838266893649
C	0.810076839169	2.041022536547	2.298583931155
H	1.456547559693	2.932357492206	2.438230290153
H	0.664480896553	1.564221393819	3.292810922686
N	-0.171995761731	0.865064276148	-1.465457943481
O	0.597035027698	0.363026189265	-2.489452569191
H	1.141150868195	-0.316270016951	-1.987907158877
C	-1.268565162615	1.652522050753	-2.014992633082
H	-1.788801466841	2.051467880979	-1.119539581534
C	-2.256684684093	0.766923925638	-2.794994528272
C	-0.754026005334	2.835972562829	-2.851458168586
H	-0.057524847922	3.461168201091	-2.257164854704
H	-0.216494897550	2.475558273362	-3.750847517640
H	-1.599618679333	3.472224176217	-3.181159048715
H	-1.759979423276	0.323164808863	-3.680791652991
H	-3.124901785484	1.363622075914	-3.139934253609
H	-2.630610458373	-0.056362423124	-2.154737304428

13.4.6 Species pertinent to reduction of ^{i}PrNO$_2$: Path A, low spin

49

^{2}**A-1.^{i}Pr** E_{tot}(UPBE0-D3(Acetonitrile)/def2TZVP // UPBE-D3(Acetonitrile)/def2SVP/W06) = -2465.00868284 $\langle S^2 \rangle$ = 0.7975

Fe	-0.664194762732	-0.255212432964	-0.621738130945
O	-1.996749717448	-1.269147715879	0.215729215153
O	0.504633836872	-1.632698515878	-0.092702189309
N	0.653527429320	0.696740113868	-1.540107798439
N	-1.863835704064	1.007407975473	-1.296840673186
C	-3.139347635648	1.132811008558	-0.989262956032
H	-3.706215809707	1.961882017800	-1.457132972409
C	-3.860146270373	0.256627409735	-0.115050258492
C	-5.228081649241	0.532524809117	0.175639426184
H	-5.681470978376	1.439729527670	-0.255848830912
C	-5.984738080057	-0.312866092460	0.979731328648
H	-7.037871937637	-0.080813230321	1.196455316296
C	-5.384813308496	-1.482338907854	1.513756942260
H	-5.977928059511	-2.158742878154	2.149380811200
C	-4.053656912807	-1.786415479954	1.244822234701
H	-3.578645433566	-2.690891685072	1.654044453128
C	-3.242397947329	-0.931363554223	0.435772460056
C	1.774174589858	-1.770568881502	-0.379182136326
C	2.473833010783	-2.906617631505	0.134909116023
H	1.897239424161	-3.616131169012	0.747333980257
C	3.827433578988	-3.102376248083	-0.120242136308
H	4.335538486036	-3.986316323267	0.296490813305
C	4.560425952621	-2.175220669528	-0.905914280434
H	5.631076787509	-2.336182823810	-1.099634904199
C	3.910318632957	-1.061481023188	-1.424808550238
H	4.464910689202	-0.327932419409	-2.032109584140
C	2.524091623888	-0.827981564095	-1.183161727584
C	1.916345943570	0.356598376038	-1.709349220707
H	2.567896199181	1.051550629991	-2.273669323835
C	0.206997924372	2.028912133457	-1.970564287823
H	0.794346360648	2.398333588920	-2.836480412664
H	0.368208754565	2.729407218850	-1.121975204446
C	-1.273696699517	1.910085509679	-2.297791481334
H	-1.778785945433	2.898118663859	-2.305313499140
H	-1.404537253372	1.452226240196	-3.302518438883
O	2.716461312198	2.659241225706	0.232504041868
N	2.058608037555	2.152683597574	1.141867253963
O	0.926517886101	2.508675389387	1.462744441371
H	-0.473692033562	0.635042434646	0.536165918203
C	2.731861967650	1.016552381218	1.925957261457
H	3.184364464686	0.405376282925	1.118745653049
C	1.704816013425	0.212269506515	2.702281560612
C	3.836931142715	1.641062283445	2.779038848018
H	4.536983069050	2.225438645019	2.152263323896
H	3.407967853851	2.301614748594	3.559173390550
H	4.404698209237	0.833484404335	3.279962648792
H	1.231817338995	0.821108500100	3.496637440708

H	2.217500658427	-0.648859741634	3.174307810489
H	0.918546841044	-0.179584442462	2.028784610219

49

^{2}A-2-ts.^{i}Pr E_{tot}(UPBE0-D3(Acetonitrile)/def2TZVP // UPBE-D3(Acetonitrile)/def2SVP/W06) = -2464.99820983 $\langle S^2 \rangle$ = 1.7078

Fe	0.453759041916	-0.711297764218	-0.491256712037
O	1.688223513264	0.535361617001	-1.161781303832
O	-0.863076860422	0.284137088196	-1.392868300845
N	-0.783996650909	-1.910503434501	0.249550122257
N	1.764111754285	-1.753791538639	0.349440387256
C	3.045874075955	-1.469369814126	0.474350563974
H	3.682888014381	-2.188541854280	1.026023143507
C	3.684775303286	-0.302666712394	-0.056641965742
C	5.072229971182	-0.092767033486	0.197664236069
H	5.608831997212	-0.835039275193	0.810843138735
C	5.744819100617	1.014240796021	-0.307457064061
H	6.814815852383	1.160046323986	-0.098334711244
C	5.034558457926	1.954357247472	-1.097922075071
H	5.558963403204	2.834623623125	-1.502378417044
C	3.681136031796	1.778638443857	-1.370416711258
H	3.121568237354	2.501445481594	-1.983372078843
C	2.957841162911	0.654421737808	-0.864006628159
C	-2.152127908151	0.062451279715	-1.464336251152
C	-2.960264697702	0.948331395811	-2.242017166078
H	-2.448077578754	1.774907345150	-2.757170545129
C	-4.338395875477	0.777440190005	-2.337524918007
H	-4.930898926994	1.480955895349	-2.943588355955
C	-4.986985697025	-0.288095408829	-1.661643262415
H	-6.076768983674	-0.414245879461	-1.740551979094
C	-4.229607377010	-1.166414457375	-0.894932884610
H	-4.717890895802	-1.995333732645	-0.357216856600
C	-2.815912553882	-1.022882390055	-0.774234894846
C	-2.089448872956	-1.929909655490	0.062499121297
H	-2.676918279232	-2.691777088285	0.611358276765
C	-0.196119548732	-2.778035486947	1.280122426936
H	-0.764929778981	-3.724004232654	1.395610754499
H	-0.238602372896	-2.226057435526	2.244470687415
C	1.248579715570	-3.026580280013	0.874443106598
H	1.859065144635	-3.397831661827	1.723730918925
H	1.291618332350	-3.785874808335	0.063393542242
O	-1.717540666398	-0.030707352969	2.750943036592
N	-1.161756065253	0.919306541632	2.176727428705
O	0.117076376600	0.876881169281	1.885341733991
H	0.307645916711	0.151588184849	0.988230639005
C	-1.999938325463	1.991794598344	1.530432401864
H	-2.644820863343	1.414115324113	0.828816685291
C	-1.141570669060	2.971080544303	0.743770626863
C	-2.881819842188	2.633386505958	2.601862457987
H	-3.439238942271	1.859042166292	3.161640715236
H	-2.269396994316	3.222359341326	3.314115109828
H	-3.610638908511	3.313034874346	2.120243759165
H	-0.462109677671	3.538030623352	1.410267551684
H	-1.810548587294	3.690916895883	0.232527972968
H	-0.543998079110	2.444391603510	-0.023159054285

49

^{2}A-3.^{i}Pr E_{tot}(UPBE0-D3(Acetonitrile)/def2TZVP // UPBE-D3(Acetonitrile)/def2SVP/W06) = -2465.02900367 $\langle S^2 \rangle$ = 1.7705

Fe	0.295539723298	-0.038483487233	-0.692865148344
O	1.580018716928	1.319285763726	-0.349890912971
O	-1.011638396470	1.329035700670	-0.511750651956
N	-0.967905992932	-1.365616398830	-1.128944524944
N	1.591443507458	-1.343326091021	-1.116290355508
C	2.896833522815	-1.241371667075	-0.949434252247
H	3.526296062042	-2.109036138588	-1.231525816318
C	3.571821427632	-0.080924111719	-0.446052606723
C	4.982841452539	-0.139870164049	-0.243861742514
H	5.498907478845	-1.091590655199	-0.451574254132
C	5.704122641651	0.961746100557	0.201926263714
H	6.791001205792	0.892185656893	0.356289287928
C	5.020083423132	2.179779701606	0.450966003053
H	5.581645379169	3.059789326568	0.803007077630
C	3.645976413126	2.277580119540	0.255289673418
H	3.107122384657	3.218593963893	0.444449866999
C	2.867057825215	1.161367109147	-0.187559414797
C	-2.295410111183	1.252514974028	-0.754511068451
C	-3.090588896075	2.434202772998	-0.623783921845

H	-2.567890654797	3.359027315343	-0.335825951112
C	-4.465389758305	2.416647210655	-0.840000920342
H	-5.044419732185	3.346623930155	-0.723930601728
C	-5.127544565557	1.215963224427	-1.204355061129
H	-6.214562167274	1.209171774664	-1.372476481066
C	-4.384752476828	0.049144251516	-1.345699601379
H	-4.882734821835	-0.892920750553	-1.628014467150
C	-2.974841055440	0.027900529790	-1.131156637037
C	-2.268019434596	-1.210214961900	-1.286478545989
H	-2.868818743741	-2.103270228452	-1.550602299637
C	-0.382728697223	-2.710570451567	-1.217554251891
H	-0.991136181366	-3.385684628811	-1.855344131230
H	-0.351104600042	-3.136829941302	-0.191058861711
C	1.030990523454	-2.541051223184	-1.758746319566
H	1.656807435186	-3.438918891037	-1.571283312123
H	0.998561019424	-2.371513818117	-2.857432262018
O	-0.638158843473	-2.464805006867	2.202840833195
N	-0.467611361374	-1.243018891022	2.348354800175
O	0.590656542336	-0.635714163451	1.509555809067
H	1.231835714217	-1.380150647186	1.433366428845
C	-1.624453042951	-0.289962575319	2.393652479446
H	-2.017511549652	-0.273290618797	1.351935770065
C	-1.154189087552	1.107234873865	2.782457808834
C	-2.681198617123	-0.869371125190	3.328184068926
H	-2.979005749250	-1.886612320584	3.013138551513
H	-2.309026134101	-0.913347551589	4.371785823203
H	-3.575065145277	-0.216959781705	3.304770558202
H	-0.667317384020	1.098898586641	3.778445098819
H	-2.033771728614	1.778577930321	2.829362246498
H	-0.456021039953	1.514671940228	2.029439807847

48

^{2}A-6.^{i}Pr E_{tot}(UPBE0-D3(Acetonitrile)/def2TZVP // UPBE-D3(Acetonitrile)/def2SVP/W06) = -2389.82331383 $\langle S^2 \rangle$ = 0.8007

Fe	0.481947058961	0.008422536010	0.616130906331
O	1.755715906935	-1.271945277067	0.124038745066
O	-0.771273092283	-1.383949134897	0.416532094210
N	-0.791814407785	1.253728629103	1.179997483986
N	1.751982963110	1.310132258917	1.035315773733
C	3.045957409582	1.262867417838	0.789126353118
H	3.660457562267	2.138566893673	1.077292260677
C	3.727084533453	0.148687425047	0.200999891914
C	5.126014066910	0.244010179681	-0.055510432213
H	5.636327229208	1.193133432269	0.175675958093
C	5.842416306761	-0.825389829917	-0.581616491507
H	6.920894386218	-0.731537149790	-0.776394695711
C	5.168080875019	-2.042286551356	-0.859602815979
H	5.728960636256	-2.894993771669	-1.273793609608
C	3.804301052027	-2.172802616519	-0.615472986746
H	3.271342618038	-3.112118709178	-0.827049764920
C	3.034652678801	-1.089290102594	-0.089023689817
C	-2.052970590546	-1.368105759290	0.694223776732
C	-2.806339974338	-2.569974913377	0.521115186387
H	-2.260479944644	-3.459961814945	0.173436728135
C	-4.174832010833	-2.609353111034	0.770007328888
H	-4.726253964990	-3.551128653349	0.621324579280
C	-4.867084514390	-1.450492134171	1.206645124972
H	-5.949593779536	-1.489509805512	1.397279893795
C	-4.161704671957	-0.266529120419	1.390602221982
H	-4.683805394226	0.643337395934	1.728219080238
C	-2.759230776625	-0.190251395479	1.148478041538
C	-2.085965637109	1.058867667943	1.341644832566
H	-2.705456684841	1.924083828584	1.647616909252
C	-0.251853244714	2.615029078759	1.295119440790
H	-0.844127382242	3.232289797889	2.001813658507
H	-0.305651874834	3.092723961707	0.292656667276
C	1.195322714270	2.473564508629	1.743362008721
H	1.784211934604	3.392849206783	1.545078986836
H	1.235575342886	2.270889698457	2.835750460149
N	-2.141396029369	1.851812142858	-1.678107599321
O	-1.280024697144	2.644954111232	-2.009443593622
H	0.421827523378	0.590802993564	-0.740256841811
C	-2.052575732168	0.505061178908	-2.329985976002
H	-1.591917127547	-0.073300124847	-1.489343821792
C	-1.134023584230	0.444208937797	-3.540620040759
C	-3.476367271350	-0.010300296745	-2.541715488970
H	-4.081782043768	0.136243279822	-1.625238586307
H	-3.969231945756	0.522650106254	-3.380760302314

H	-3.462055163401	-1.091820165682	-2.777810151528
H	-1.525461175837	1.077836584721	-4.363362562063
H	-1.060037290507	-0.597548141326	-3.908261589198
H	-0.116409386862	0.800213027898	-3.289392109295

48

^{2}**A-7-ts.^{i}Pr** E_{tot}(UPBE0-D3(Acetonitrile)/def2TZVP // UPBE-D3(Acetonitrile)/def2SVP/W06) = -2389.80873226 $\langle S^2 \rangle$ = 1.4843

Fe	0.304841373624	0.545671609280	0.471718633460
O	1.546678732717	-0.768089002147	0.962011004038
O	-1.012478298889	-0.542929003801	1.251176454013
N	-0.929624129771	1.844076025301	-0.098682171543
N	1.622589885663	1.787268827855	-0.019022040194
C	2.919044253567	1.571084532216	-0.114392548111
H	3.557209724410	2.407984918218	-0.460036395819
C	3.573361549575	0.337807232013	0.205979461073
C	4.981738124463	0.226227616036	0.015146147513
H	5.524506668972	1.094561704345	-0.392316864363
C	5.666926178142	-0.941700823145	0.331292058825
H	6.753498140422	-1.010205595041	0.174905349358
C	4.948586231072	-2.044807523472	0.859948497714
H	5.483378106857	-2.973954649758	1.112892008443
C	3.573675053967	-1.969291222349	1.063394094490
H	3.007540873047	-2.819469931948	1.473027103142
C	2.838927838455	-0.786210364511	0.744650746613
C	-2.312294285261	-0.375920759250	1.266420713113
C	-3.125623539027	-1.368352852330	1.893697054680
H	-2.609732098297	-2.227535541136	2.348080754544
C	-4.513351369000	-1.260380016390	1.914485221083
H	-5.110817564475	-2.049247771754	2.398110522373
C	-5.165273130201	-0.151505809750	1.316606803500
H	-6.262289300438	-0.075569592414	1.336489565238
C	-4.401656460115	0.837549687519	0.707014220193
H	-4.891499222549	1.707494815032	0.240464981717
C	-2.978763186621	0.758876324239	0.662828856179
C	-2.241726466472	1.806816094487	0.020654774763
H	-2.825367839559	2.638766393528	-0.419680179701
C	-0.305967429611	2.920165516392	-0.880467478869
H	-0.909216134954	3.850957266607	-0.854981553254
H	-0.231022098107	2.582527031575	-1.936865382291
C	1.086319872687	3.125540967144	-0.303259689991
H	1.743823812457	3.691926413076	-0.994810842639
H	1.022104702996	3.691315917428	0.651503671221
N	-0.749155913161	-0.636259890405	-2.960875667217
O	0.357977885218	-0.296997509071	-2.476265974478
H	0.302722344171	-0.013092787004	-1.006459065090
C	-1.600062184281	-1.390660474671	-2.015129428625
H	-1.711867346854	-0.687036032895	-1.152993764230
C	-0.893522256869	-2.642781857799	-1.475824702905
C	-2.954597524451	-1.659955474098	-2.658010093761
H	-3.393250195372	-0.724250748090	-3.057753689078
H	-2.862442041761	-2.385384621180	-3.492265424804
H	-3.656970023442	-2.079019132465	-1.911062150156
H	-0.673250715455	-3.357091820055	-2.295550807138
H	-1.548264328927	-3.145347051347	-0.736734500082
H	0.046785751028	-2.362266398799	-0.964168768141

48

^{2}**A-8.^{i}Pr** E_{tot}(UPBE0-D3(Acetonitrile)/def2TZVP // UPBE-D3(Acetonitrile)/def2SVP/W06) = -2389.87523945 $\langle S^2 \rangle$ = 0.9390

Fe	0.043564011117	0.126605040230	0.432341672015
O	1.380221236777	-1.310040177503	0.374677226379
O	-1.225966242634	-1.270932113992	0.389858099633
N	-1.236042594220	1.461772327925	0.771558903108
N	1.310649175802	1.419540640616	1.044374601368
C	2.614111039013	1.287250560937	1.062339431657
H	3.218157664288	2.136528961659	1.442597321196
C	3.340044968230	0.114810837035	0.651898248351
C	4.763427834623	0.181980745806	0.616612554401
H	5.244418824694	1.143335213308	0.860580260367
C	5.542393969408	-0.921744440113	0.284351323564
H	6.639082221418	-0.843794317932	0.255389427568
C	4.900873369569	-2.150310289160	-0.009824529121
H	5.504116792693	-3.034064872979	-0.271914793924
C	3.512962402174	-2.256678292474	0.030381216401
H	3.008437143378	-3.209387366009	-0.192252733268
C	2.682291174388	-1.139374963128	0.352177259162
C	-2.529463276217	-1.175189295580	0.450313682345

C	-3.312047973838	-2.361523637657	0.294821897796
H	-2.768326574325	-3.300297604471	0.109332989961
C	-4.702438262472	-2.331994879695	0.365185275153
H	-5.271195566452	-3.265822079026	0.230579757862
C	-5.391672500799	-1.117036856504	0.606852676191
H	-6.490082284292	-1.101186059286	0.662061184205
C	-4.659108298741	0.054159572572	0.774484532402
H	-5.176893548400	1.008019283421	0.967149295353
C	-3.236680506292	0.063700632484	0.697461757791
C	-2.537460846749	1.301799737455	0.894069026985
H	-3.145770456097	2.187164483091	1.165390479616
C	-0.657938914928	2.798229706909	0.971214960740
H	-1.328807711604	3.452229038910	1.565631033149
H	-0.515585386182	3.277148032868	-0.022770903119
C	0.690136888774	2.604105718739	1.651402953588
H	1.336934779580	3.501860923077	1.560415462746
H	0.535852243856	2.405419033206	2.735086039624
N	0.262096757078	0.288454512718	-1.375391575264
O	0.275426436808	-0.960709032402	-1.998032062621
H	0.738930371454	-1.502516619856	-1.300947474642
C	-0.386381825902	1.240386640202	-2.282874758644
H	-0.445988683575	2.179066767151	-1.697014160420
C	-1.802006760340	0.820428954961	-2.703763430742
C	0.532563116233	1.489815341877	-3.490821120302
H	1.540739833590	1.804850071891	-3.156042137043
H	0.638274191862	0.566825870137	-4.094283278559
H	0.109616830037	2.286319625540	-4.134987477645
H	-1.770753028623	-0.119911331053	-3.287962121399
H	-2.264514050411	1.607713742886	-3.331998251181
H	-2.445316290980	0.656951611861	-1.818440750738

13.4.7 Species pertinent to reduction of ^{i}PrNO$_2$: Paths B&C

49

^{6}**B-1.^{i}Pr** E_{tot}(UPBE0-D3(Acetonitrile)/def2TZVP // UPBE-D3(Acetonitrile)/def2SVP/W06) = -2465.00667020 $\langle S^2 \rangle$ = 8.7618

Fe	0.175945368053	-0.797377926768	-0.372033376935
O	-1.415603295958	0.053923760435	0.337011174813
O	1.537208853961	0.321832986200	0.424588055593
N	1.730021429264	-2.252241344161	-0.571705174163
N	-0.839242942523	-2.645724054330	0.011693990011
C	-2.125895850368	-2.774569976229	0.176078318490
H	-2.552450869513	-3.801452713806	0.208689996910
C	-3.076345642406	-1.701270421776	0.333176324919
C	-4.453862592714	-2.037709773502	0.440628046017
H	-4.738118680852	-3.101226210444	0.386612076746
C	-5.431121707249	-1.059419549972	0.606443458880
H	-6.492643106554	-1.336061797290	0.682635507690
C	-5.034999517180	0.297197732402	0.671862709557
H	-5.798487172388	1.082074375979	0.793751414339
C	-3.692473373318	0.664742536661	0.583848094119
H	-3.394747949805	1.722851266889	0.614678864445
C	-2.669550161563	-0.315278486272	0.412211627391
C	2.847750032261	0.295899332856	0.464577016852
C	3.556868782238	1.440766227678	0.933693637584
H	2.965210821647	2.313276789577	1.244484421702
C	4.950188013198	1.455355231004	0.995272040519
H	5.463130488098	2.359713013665	1.358977868007
C	5.708923104521	0.328441943973	0.602481027378
H	6.806963868690	0.348189782099	0.656956671641
C	5.042494105061	-0.808237126705	0.149401131995
H	5.613948628897	-1.699930062952	-0.155530347874
C	3.624708006846	-0.855310149972	0.063777844943
C	3.005862700274	-2.077925326161	-0.393777929768
H	3.694854158832	-2.927607176157	-0.597134773436
C	1.190590611579	-3.539296025038	-0.977501089110
H	1.946412887717	-4.352905091465	-0.934523217182
H	0.830127644292	-3.455701590661	-2.026949385004
C	0.001744087463	-3.838202769821	-0.058961204212
H	-0.564337688162	-4.728766075595	-0.407377063461
H	0.384994364154	-4.057464005109	0.962487800581
O	-2.484304731679	3.780058391222	0.020408886270
N	-1.590944602787	3.339714994959	-0.707372618128
O	-1.772594448474	2.572499633881	-1.645447807092
H	0.070112197031	-0.423717388331	-2.019907798967

C	-0.159040713963	3.802912869043	-0.382588592319
H	-0.243345183680	4.906594106838	-0.470927255171
C	0.827935217607	3.243120696731	-1.391213688647
C	0.134150015394	3.420912931578	1.068682474180
H	-0.650079098579	3.812942104041	1.742130449349
H	0.203320922397	2.320020923616	1.167343730973
H	1.104229657865	3.862773140010	1.368402643830
H	0.884020217998	2.140318920904	-1.313383354043
H	1.832240571941	3.655927743702	-1.171109745169
H	0.556024834027	3.520431312422	-2.426784058903

49

^{6}B-2-ts.^{i}Pr E_{tot}(UPBE0-D3(Acetonitrile)/def2TZVP // UPBE-D3(Acetonitrile)/def2SVP/W06) = -2464.98585625 $\langle S^2 \rangle$ = 8.8483

Fe	-0.016971113150	-0.682062621719	0.267567685919
O	-1.515953419744	0.185866068156	1.096275794437
O	1.525396748316	0.047700777781	1.150977792152
N	1.289271658636	-2.065257044768	-0.654240481254
N	-1.333054965534	-2.286869847329	-0.157338279235
C	-2.632082576994	-2.185498289437	-0.205320649491
H	-3.213772116503	-3.031090511400	-0.634650263331
C	-3.410498747714	-1.059971054598	0.256881546952
C	-4.821329916166	-1.103267298476	0.086904019332
H	-5.262569042175	-1.988203330504	-0.399759218028
C	-5.639935606220	-0.061170172931	0.517771752260
H	-6.729012454877	-0.110861179625	0.374383002954
C	-5.049582603431	1.061876099003	1.143225944631
H	-5.686319395294	1.892483009814	1.486742683318
C	-3.669735039303	1.135602042481	1.332778247958
H	-3.204701987079	2.005818145958	1.819294167225
C	-2.806944724839	0.088172390424	0.896741585208
C	2.815754896181	-0.054448575223	0.940472982822
C	3.702723602585	0.853493616117	1.589401712656
H	3.256274752257	1.617215872006	2.243424882993
C	5.082177578475	0.779501847394	1.398569522185
H	5.737214225989	1.499987048601	1.913493694041
C	5.648581749911	-0.205815953375	0.556339982921
H	6.737588160457	-0.258785875716	0.413879614217
C	4.805616754768	-1.106520517090	-0.090941056040
H	5.227255470962	-1.880112725258	-0.753061627694
C	3.393769038148	-1.055154499133	0.070730773937
C	2.590716899463	-2.016410357804	-0.649356444129
H	3.154217446529	-2.764170488258	-1.250112744031
C	0.576509064778	-3.070389754983	-1.431767432686
H	1.213775608034	-3.947760391089	-1.674109615945
H	0.251534694888	-2.607205014247	-2.390030881158
C	-0.665104350783	-3.491461000721	-0.635702263037
H	-1.335401798665	-4.131094421888	-1.249782033093
H	-0.342117113706	-4.083983027186	0.248814442529
O	-1.787972688536	2.683325286658	-1.561070252931
N	-0.554025847604	2.488360286346	-1.658861073424
O	-0.063564218765	1.423559106301	-2.154010453931
H	-0.105469012272	0.239174962880	-1.188639449842
C	0.367395645103	3.416541192112	-0.898025203293
H	0.068525104388	4.425577408407	-1.252751236405
C	1.823328822678	3.134315726556	-1.242126334290
C	0.062175708111	3.299774852312	0.599176855641
H	-1.014507036917	3.473926234645	0.785012256182
H	0.332594835202	2.295564794554	0.980254535386
H	0.648501311640	4.056879938022	1.156085946562
H	2.109417538114	2.119307321651	-0.905808902983
H	2.471438709064	3.870748364123	-0.727035358436
H	2.006976584718	3.207946602511	-2.331241189999

49

^{6}B-3.^{i}Pr E_{tot}(UPBE0-D3(Acetonitrile)/def2TZVP // UPBE-D3(Acetonitrile)/def2SVP/W06) = -2465.06266296 $\langle S^2 \rangle$ = 8.7658

Fe	-0.124135321458	-0.582241391112	-0.028275832332
O	-1.554945737954	0.219392374163	1.021278556872
O	1.371919254626	-0.076462859091	1.108561435161
N	1.145175671856	-2.120515554731	-0.759582586781
N	-1.491291217737	-2.208935950675	-0.322866954870
C	-2.787177821246	-2.102669441341	-0.263820152037
H	-3.407366226526	-2.951792928650	-0.627752987806
C	-3.518146953020	-0.969579663177	0.255857263480
C	-4.937037382650	-0.985114799266	0.170233983115
H	-5.421828486878	-1.849510305115	-0.312063914790
C	-5.710241129054	0.058087411913	0.675284012758

H	-6.806540991576	0.030858407406	0.594548432855
C	-5.063590599561	1.151832330697	1.297761457309
H	-5.663690226974	1.982384047649	1.702181037451
C	-3.674386236852	1.195546718882	1.410635917454
H	-3.166693489040	2.042279013981	1.896487493459
C	-2.856216718642	0.147991410014	0.892504039471
C	2.661854236667	-0.254516941183	0.993120736636
C	3.553016555269	0.561778632803	1.753909266378
H	3.104310620568	1.326239212287	2.405902201380
C	4.935310037480	0.401307271944	1.670272291706
H	5.590985676014	1.054892697220	2.267330320053
C	5.504972979385	-0.587228484325	0.832927640290
H	6.596392006375	-0.707743866280	0.774728005918
C	4.661038167087	-1.406444406927	0.086731441820
H	5.084452360361	-2.186328996571	-0.567108425142
C	3.247030307748	-1.264826356563	0.136930706694
C	2.440331193316	-2.167162456464	-0.652359314927
H	2.993536016037	-2.961294962311	-1.201549831881
C	0.406256410194	-3.078206687990	-1.566126660241
H	1.001535449234	-3.986066813484	-1.804445687509
H	0.128568333811	-2.585227714867	-2.524464191278
C	-0.870785228569	-3.440313085584	-0.799143833681
H	-1.561051743341	-4.043413762464	-1.427971051815
H	-0.593779547134	-4.054093986949	0.086789650457
O	-0.823323749804	3.295648892343	-1.986696215183
N	0.216984926185	3.924072680479	-1.916928020485
O	-0.139364479892	0.419336968970	-1.594407153008
H	-0.702436112316	1.223629220667	-1.588074688899
C	0.981280659905	3.757392855927	-0.636860769799
H	1.402028193396	4.775095643626	-0.472118422808
C	2.143258087251	2.810517312321	-1.007331750755
C	0.151938883668	3.295831504547	0.551700999709
H	-0.696418818057	3.985357421811	0.737054416277
H	-0.255916697788	2.280161463561	0.399774373161
H	0.786068017285	3.269567264628	1.459292201432
H	1.729436695935	1.804938858267	-1.225243863223
H	2.844724756691	2.738062262492	-0.153385514329
H	2.698601923632	3.179273998056	-1.892351620388

49

^{6}C-1.^{i}Pr E_{tot}(UPBE0-D3(Acetonitrile)/def2TZVP // UPBE-D3(Acetonitrile)/def2SVP/W06) = -2464.99626607 $\langle S^2 \rangle$ = 8.7628

Fe	0.272609062291	-0.348281841373	0.163210308277
O	1.738391834541	0.343640024859	-0.915017734252
O	-1.053697006027	-0.545854658134	-1.269878943245
N	-0.917608755960	-1.851609924602	1.123503602724
N	1.728569032047	-1.851820578242	0.776063863279
C	3.021331010818	-1.752548396388	0.661927886083
H	3.659177923586	-2.508926342368	1.171905213350
C	3.731648505089	-0.746161980050	-0.090707475692
C	5.152641046562	-0.777314345159	-0.090412241934
H	5.658299622929	-1.552725712097	0.508142991576
C	5.903032193403	0.138430832812	-0.825175838783
H	7.001946166875	0.100265784457	-0.811062903700
C	5.228511054180	1.116120510696	-1.594832168362
H	5.809998216392	1.844101434959	-2.182760771988
C	3.835913108858	1.173278524981	-1.622534673532
H	3.306356588766	1.931245356732	-2.219128150630
C	3.039223168827	0.256600071931	-0.869867830936
C	-2.333793183766	-0.797167853066	-1.234230582467
C	-3.176322746656	-0.341072770238	-2.294428967575
H	-2.703587441505	0.239042952694	-3.101276297623
C	-4.544332161042	-0.609182250614	-2.300950913423
H	-5.164706301368	-0.231078132860	-3.129211669477
C	-5.145104614917	-1.359678595589	-1.261972468166
H	-6.224764120714	-1.568465552304	-1.277726680097
C	-4.346788327931	-1.833196212567	-0.221913497317
H	-4.793552749180	-2.427469440256	0.592057613066
C	-2.953811627251	-1.563737854974	-0.173468648232
C	-2.172082882430	-2.115143223693	0.907475654856
H	-2.696641494178	-2.828213654605	1.582658166391
C	-0.160026633397	-2.535811521888	2.154992626709
H	-0.704868658549	-3.405242314668	2.582648390886
H	0.055399672923	-1.814966063038	2.973524764467
C	1.159301380885	-2.977491738437	1.512258115341
H	1.864233381477	-3.380089985797	2.271941843686
H	0.943088840478	-3.792660156566	0.785766734336
O	-2.205368255158	1.341058894889	1.148789057719

N	-1.455549295236	2.193509098856	0.683612800839
O	-0.464590850032	1.934463074008	-0.021445902460
H	0.394938361788	0.384791273557	1.700187197879
C	-1.641154486019	3.641289021759	1.119186947280
H	-2.703743166592	3.676430213317	1.426855412326
C	-1.367533133920	4.582412821587	-0.044305812917
C	-0.728433964161	3.855189713708	2.328903781096
H	-0.949652176054	3.121747929072	3.128417658073
H	0.335461733190	3.755615881437	2.035832561301
H	-0.892390742157	4.873459658138	2.730845960885
H	-0.313077343962	4.507313554328	-0.372077283609
H	-1.561451495354	5.622184417760	0.283614680913
H	-2.027369333696	4.363069387886	-0.906120489368

49

^{6}C-2-ts.^{i}Pr E_{tot}(UPBE0-D3(Acetonitrile)/def2TZVP // UPBE-D3(Acetonitrile)/def2SVP/W06) = -2464.97495022 $\langle S^2 \rangle$ = 8.9050

Fe	0.175940997855	-0.432328024540	-0.029018470963
O	1.712750917432	-0.666898468083	-1.169039622803
O	-1.257180065932	-1.137053708597	-1.127385149246
N	-1.161102483842	-0.630034262255	1.597796101883
N	1.479801125467	-1.053458282024	1.564830143476
C	2.781955054609	-1.072440783823	1.521310705815
H	3.341728323436	-1.265734451793	2.463368299672
C	3.588983299296	-0.865212391094	0.343297586010
C	5.004111734004	-0.873365485604	0.482318846215
H	5.429428260567	-1.024177496603	1.487930785623
C	5.846039357242	-0.696892884813	-0.613218927514
H	6.938215407758	-0.703052233925	-0.485116238166
C	5.275382603689	-0.511965347761	-1.895401587205
H	5.931050691096	-0.372628058974	-2.769582619904
C	3.892724622982	-0.504695610251	-2.070539287640
H	3.442622672361	-0.362827358345	-3.064492600627
C	3.003650818405	-0.674441912515	-0.966063047936
C	-2.547620746885	-1.224867568295	-0.964893655911
C	-3.380816231866	-1.507853348641	-2.090903589700
H	-2.887007071094	-1.621975825207	-3.067630764187
C	-4.762806986039	-1.623507551017	-1.961095275277
H	-5.373518954093	-1.832671392316	-2.853883383404
C	-5.391534769021	-1.477643070302	-0.700245665012
H	-6.483081347315	-1.574032188870	-0.607266894089
C	-4.605573044613	-1.215256426399	0.418791231810
H	-5.074358714815	-1.106130928459	1.410506628771
C	-3.193714735682	-1.076374101039	0.322558185322
C	-2.444983107267	-0.840644742443	1.530672228634
H	-3.029739032162	-0.855436085064	2.477307562635
C	-0.489740436727	-0.490207818943	2.879885410527
H	-1.130231993628	-0.804721580025	3.731969090923
H	-0.217649315501	0.578931942980	3.024562901499
C	0.789909096865	-1.332356956025	2.819681195803
H	1.436340415664	-1.146486543162	3.704781014839
H	0.506126094752	-2.408468277604	2.825775984008
O	-2.095531780144	1.833568582659	-0.594165628292
N	-0.987998549070	2.421614015796	-0.640147241076
O	0.139305439824	1.750827517704	-0.688345449089
H	0.441966072289	1.065234188123	0.646669215067
C	-0.887238331037	3.793876009030	-0.033488543028
H	-1.825045069314	4.284238726490	-0.363055194992
C	0.324112316862	4.544432433518	-0.571468092010
C	-0.893650534229	3.639986726669	1.490675524006
H	-1.791442808517	3.081314485100	1.819318541567
H	0.009126612342	3.093852219122	1.835986704072
H	-0.896488825529	4.636977469309	1.972006429912
H	1.263708067459	4.049020940105	-0.258947478165
H	0.323409465491	5.578841201414	-0.175088575909
H	0.305578483668	4.596643174128	-1.677744856124

49

^{6}C-3.^{i}Pr E_{tot}(UPBE0-D3(Acetonitrile)/def2TZVP // UPBE-D3(Acetonitrile)/def2SVP/W06) = -2465.06383204 $\langle S^2 \rangle$ = 8.7624

Fe	-0.178214479734	-0.549443535052	0.127795286767
O	1.353797617166	0.176228373841	-0.852333272997
O	-1.588977746168	0.321311905554	-0.913261805715
N	-1.658441705968	-1.990760884011	0.633308924959
N	0.946809873406	-2.377586683350	0.174981945129
C	2.240927258746	-2.460466580484	0.062587984305
H	2.732391870376	-3.433161883400	0.287864310399
C	3.123545436738	-1.399566939762	-0.364771207681

C	4.522094446828	-1.653031289277	-0.378587539421
H	4.878722401977	-2.633982246820	-0.024132443877
C	5.433361391724	-0.699672999207	-0.828826844793
H	6.512063340962	-0.913201235432	-0.827516132191
C	4.948254505465	0.543856385412	-1.297027284728
H	5.657115271709	1.304074208982	-1.661845471295
C	3.582500560501	0.824355771071	-1.307101750747
H	3.202505525987	1.788967005086	-1.673815006789
C	2.625631621641	-0.124323558601	-0.833749974566
C	-2.893330462636	0.295099208621	-0.821461026958
C	-3.661689678701	1.331131655687	-1.434543645940
H	-3.110533439919	2.131993721835	-1.950049249840
C	-5.054805447425	1.330480932822	-1.383302232692
H	-5.612108348033	2.149427326624	-1.865465324894
C	-5.760058052958	0.295866923543	-0.723816185896
H	-6.859161078011	0.302607107632	-0.690270113825
C	-5.038715000603	-0.730081846228	-0.117726706087
H	-5.568118761473	-1.545650633660	0.401609032497
C	-3.617239529569	-0.758149635889	-0.140049768250
C	-2.946219707031	-1.858877783539	0.513103467479
H	-3.606155184133	-2.645375701031	0.942794947873
C	-1.065162380198	-3.128711472046	1.315885691019
H	-1.778759021016	-3.971439438791	1.443559143650
H	-0.739533352854	-2.795706713314	2.326690956599
C	0.163905256786	-3.562777163513	0.510675864693
H	0.761859231800	-4.318518909120	1.064865849149
H	-0.178296879312	-4.032005346859	-0.438712825509
O	2.780603435765	2.707430957666	1.088112499673
N	1.605109244826	2.482896694445	1.269412483449
O	-0.030992086461	0.234006769765	1.790559361222
H	0.683502964790	0.930211652552	1.783004298021
C	0.669887057327	3.593827933867	0.888710148501
H	1.253990640814	4.538294572738	0.957738907981
C	-0.511637968668	3.559028116964	1.857039973070
C	0.247901680444	3.358356975119	-0.572293762017
H	1.129799591788	3.357804099446	-1.242057210988
H	-0.289773499231	2.397562822205	-0.689399146790
H	-0.427014281233	4.182346206541	-0.878647046917
H	-0.969910616488	2.549126486154	1.842102001279
H	-1.271615813807	4.307008033391	1.557913987821
H	-0.192091670345	3.783415025011	2.894080153726

13.4.8 Species related to reduction of $PhNO_2$

14

Ph-NO_2 E_{tot}(RPBE0-D3(Acetonitrile)/def2TZVP // RPBE-D3(Acetonitrile)/def2SVP/W06) = -436.43969318 $\langle S^2 \rangle$ = 0.0000

C	0.000000000000	0.000000000000	0.246715587448
C	0.000000000000	1.228968722211	-0.430415575248
C	0.000000000000	1.220409068365	-1.831744079770
C	0.000000000000	0.000000000000	-2.531109160786
C	0.000000000000	-1.220409068365	-1.831744079770
C	0.000000000000	-1.228968722211	-0.430415575248
H	0.000000000000	2.163225358650	0.146183675953
H	0.000000000000	2.173184972659	-2.381552013606
H	0.000000000000	0.000000000000	-3.631482314474
H	0.000000000000	-2.173184972659	-2.381552013606
H	0.000000000000	-2.163225358650	0.146183675953
N	0.000000000000	0.000000000000	1.727830674848
O	0.000000000000	-1.090216172066	2.303730097881
O	0.000000000000	1.090216172066	2.303730097881

13

Ph-NO E_{tot}(RPBE0-D3(Acetonitrile)/def2TZVP // RPBE-D3(Acetonitrile)/def2SVP/W06) = -361.26280403 $\langle S^2 \rangle$ = 0.0000

C	0.000000000000	0.605340524443	0.000000000000
C	1.343920369279	0.177701211082	0.000000000000
C	1.630944091278	-1.193461356693	0.000000000000
C	0.575438152129	-2.123124094559	0.000000000000
C	-0.769352734556	-1.690662699259	0.000000000000
C	-1.063130450694	-0.326173432684	0.000000000000
H	2.135714023579	0.942429952851	0.000000000000
H	2.674197916785	-1.542531542114	0.000000000000
H	0.798838539420	-3.201036079793	0.000000000000
H	-1.582120738975	-2.432937685848	0.000000000000

```
H          -2.093956494474       0.058554747571       0.000000000000
N          -0.193057154780       2.029427522633       0.000000000000
O          -1.361023715935       2.408975879366       0.000000000000
```

14

Ph-NH$_2$ E_{tot}(RPBE0-D3(Acetonitrile)/def2TZVP // RPBE-D3(Acetonitrile)/def2SVP/W06) = -287.37537490 $\langle S^2 \rangle$ = 0.0000

```
C          -0.003858577836       0.955723262610       0.000000000000
C          -0.001013820882       0.223094830035      -1.217140645028
C          -0.001013820882      -1.178223014958      -1.209897573477
C          -0.001980356882      -1.896000125035       0.000000000000
C          -0.001013820882      -1.178223014958       1.209897573477
C          -0.001013820882       0.223094830035       1.217140645028
H           0.000657882181       0.770106656411      -2.173703354363
H           0.003041023441      -1.718023007565      -2.170335056124
H          -0.000312893754      -2.996204135211       0.000000000000
H           0.003041023441      -1.718023007565       2.170335056124
H           0.000657882181       0.770106656411       2.173703354363
N          -0.060669936814       2.338096366286       0.000000000000
H           0.238484974846       2.814280833573       0.852222951933
H           0.238484974846       2.814280833573      -0.852222951933
```

50

^{6}A-1.Ph E_{tot}(UPBE0-D3(Acetonitrile)/def2TZVP // UPBE-D3(Acetonitrile)/def2SVP/W06) = -2578.02275091 $\langle S^2 \rangle$ = 8.7617

```
Fe         -0.982146398692      -0.093814007703       0.087896219865
O          -2.529042288477      -1.264101589042       0.234083203617
O           0.353532068019      -1.473182469393      -0.117753223968
N           0.331520069618       1.118059456909      -1.082733458135
N          -2.311321007624       1.247270565658      -0.927241823348
C          -3.606250076903       1.258104915862      -0.775026568589
H          -4.181521261205       2.112222682846      -1.196339109643
C          -4.391871236800       0.236373863128      -0.127378340319
C          -5.793207965512       0.441121996867       0.001894460433
H          -6.217918959398       1.392738430845      -0.357031057729
C          -6.622557604414      -0.526750049970       0.563436909697
H          -7.703338983639      -0.348930511764       0.660303157261
C          -6.054248431684      -1.747333475581       1.000020968322
H          -6.700560555434      -2.523010060791       1.440635529366
C          -4.686254975460      -1.987228855720       0.879155373595
H          -4.240187144779      -2.935240640010       1.215104544685
C          -3.807485040311      -1.007715609369       0.325689745820
C           1.480361505563      -1.602517642278      -0.769363576993
C           2.191799370161      -2.837320493508      -0.703093128382
H           1.754003830731      -3.641979204577      -0.094070873319
C           3.398557481020      -3.014648880353      -1.378527345628
H           3.921706279091      -3.980967844025      -1.302039256344
C           3.958699801591      -1.974107032121      -2.155762196723
H           4.911444653576      -2.123445664973      -2.683975603978
C           3.285903260263      -0.757145369947      -2.239427084174
H           3.707475993941       0.069344254138      -2.834115111833
C           2.057231108386      -0.539284390156      -1.560699815128
C           1.433659641379       0.756413499193      -1.673168076956
H           1.964661325822       1.500057069944      -2.306774906662
C          -0.220193249346       2.450012189505      -1.278775969784
H           0.361352785295       3.045048122333      -2.015098506246
H          -0.183008122025       2.982893052193      -0.302936949080
C          -1.673158879892       2.284455229967      -1.734687584249
H          -2.223034161679       3.249374402102      -1.686961133101
H          -1.678018294837       1.946712423812      -2.795029186239
O           2.740130542449       3.064809882635       0.126704426001
N           2.251826101758       2.430158449949       1.069220160144
O           1.272546953112       2.807491723753       1.720122185633
H          -0.927693687347       0.524967150917       1.650577303368
C           2.858160080213       1.135517481757       1.420421535459
C           4.093990307597       0.789241797679       0.851306689791
C           2.161265334587       0.276143205136       2.283817910759
C           4.649331879730      -0.456449340123       1.166298078842
C           2.723750532663      -0.972941340649       2.575124627716
C           3.962977597106      -1.338329840941       2.020661615878
H           4.591809454679       1.491291356740       0.169975951275
H           1.180735886888       0.580173175950       2.672721679132
H           5.614763894204      -0.748522172026       0.728429656641
H           2.183123113486      -1.672228917792       3.229869387469
H           4.395733331910      -2.323563747081       2.249976511258
```

50

[6]**A-2-ts.Ph** E_{tot}(UPBE0-D3(Acetonitrile)/def2TZVP // UPBE-D3(Acetonitrile)/def2SVP/W06) = -2578.00821932 $\langle S^2 \rangle$ = 8.8379

Fe	-0.915355324525	-0.430273349107	-0.158516389586
O	-2.485038286185	-1.203576956173	0.623986999771
O	0.431375897768	-1.769663472140	0.094197141730
N	0.437940382178	0.518030897752	-1.485763217563
N	-2.231345906832	0.569716279375	-1.505092529960
C	-3.508181818939	0.741392256883	-1.308437049921
H	-4.058041134904	1.435910249023	-1.981373389032
C	-4.305272762154	0.100548043341	-0.289012464839
C	-5.688206777974	0.420662082229	-0.217398298069
H	-6.092323237769	1.156810902358	-0.931036597655
C	-6.526198787125	-0.173069464309	0.724636336330
H	-7.593292330163	0.088888183656	0.766224643004
C	-5.984640155680	-1.119694727187	1.625079030784
H	-6.637166705443	-1.596922486275	2.373209880628
C	-4.632470614918	-1.460185527981	1.581686584509
H	-4.205225785059	-2.194935336520	2.280211338376
C	-3.750144534126	-0.862933016276	0.634816464200
C	1.676146987149	-1.915263992279	-0.286917767575
C	2.429999726334	-3.024471951006	0.196743407137
H	1.925038477571	-3.719196472577	0.884184996493
C	3.757964762530	-3.216210594606	-0.182448166365
H	4.312104118952	-4.082071609812	0.213082657149
C	4.400111099310	-2.315798963356	-1.064044443569
H	5.448689826212	-2.473240397617	-1.355054187154
C	3.685412147674	-1.226611653124	-1.556693889777
H	4.169075743960	-0.511089081213	-2.240745179440
C	2.332523703286	-0.996951870846	-1.189718867009
C	1.658014077314	0.147446724485	-1.751258217539
H	2.247384066627	0.749112793365	-2.477497322464
C	-0.161327202544	1.667186140480	-2.146555078294
H	0.449672705030	2.027102971863	-3.002257011794
H	-0.231549150164	2.491154005594	-1.402007776135
C	-1.563506902827	1.254240411419	-2.608059127054
H	-2.142447339050	2.131752497162	-2.968652301719
H	-1.468820664864	0.537041026368	-3.453641572334
O	1.008957765327	3.430866191432	0.332242775467
N	1.109256238851	2.480615808197	1.151162939372
O	0.125003069228	2.043816167624	1.812818795887
H	-0.636400805802	0.771041132728	0.993029332904
C	2.354761264677	1.752095976166	1.240029687116
C	3.481760424688	2.229388342276	0.542691685512
C	2.409341462074	0.566550580533	1.996224882972
C	4.678820362122	1.507795564581	0.615014889974
C	3.613780362772	-0.148276759785	2.049155829400
C	4.749602178488	0.319384208340	1.366364205701
H	3.389021777434	3.150739415793	-0.046883359832
H	1.503857215888	0.216988543331	2.507811356896
H	5.564959951268	1.870644888512	0.072521234922
H	3.661654537793	-1.086308844444	2.621663728188
H	5.689970588857	-0.249722507589	1.409121810704

50

[6]**A-3.Ph** E_{tot}(UPBE0-D3(Acetonitrile)/def2TZVP // UPBE-D3(Acetonitrile)/def2SVP/W06) = -2578.08336068 $\langle S^2 \rangle$ = 8.7707

Fe	-0.736368768038	-0.404015561981	0.058158233322
O	-2.381011179314	-1.418391379705	0.385900966980
O	0.545265901151	-1.774795784881	0.527206796321
N	0.750378900297	0.295920239906	-1.303081691488
N	-1.887867597438	0.602190216240	-1.443031089035
C	-3.174415023033	0.792452934238	-1.356815041147
H	-3.652298083544	1.523068590258	-2.046921785859
C	-4.064145855936	0.107458663036	-0.451117020290
C	-5.433168106705	0.493380019240	-0.426074831465
H	-5.749783027072	1.335638829743	-1.062776078733
C	-6.362260225141	-0.164988675271	0.374715603023
H	-7.415216841678	0.151829225345	0.386015920889
C	-5.932388409473	-1.256312017342	1.168551944459
H	-6.659358850041	-1.788832794345	1.802298810875
C	-4.602186975595	-1.670564873198	1.160935190501
H	-4.264342672443	-2.519221589944	1.774488941356
C	-3.618919215037	-1.002981593652	0.367304387357
C	1.732959032746	-2.125840651407	0.106292886491
C	2.386791712609	-3.242845646015	0.708805678902
H	1.849889805827	-3.770184087531	1.511549471162
C	3.656887900400	-3.648651827524	0.299940438278
H	4.130095188056	-4.514284816565	0.790154509721
C	4.342130659235	-2.965639461255	-0.732079107202

H	5.343291905777	-3.292117542206	-1.048738092695
C	3.729585112460	-1.869222259062	-1.336190857403
H	4.251478707917	-1.313583645083	-2.132227863399
C	2.439623046528	-1.425359257467	-0.941862596102
C	1.901247857962	-0.242922176724	-1.573008971602
H	2.549538165783	0.240893911864	-2.336600295018
C	0.305876224805	1.505583975038	-1.973733725229
H	0.985020706836	1.807148880066	-2.800733358366
H	0.288432382098	2.328709336639	-1.225439305783
C	-1.115357751547	1.258524382165	-2.491702706789
H	-1.589206795392	2.204136293531	-2.834719075258
H	-1.065176905296	0.570409605643	-3.364989606138
O	0.460392822683	3.935452914775	0.598440064517
N	0.844248861559	3.148183877120	1.463462313651
O	-0.722673511567	0.874551003325	1.403645872811
H	-1.470626483169	1.502797313252	1.427638672398
C	2.036841962577	2.413519779850	1.150446990775
C	2.920616606852	2.801498008169	0.119693838948
C	2.316661971340	1.297816778836	1.966940709152
C	4.090729927509	2.064866854871	-0.088146099554
C	3.479454046946	0.554637333614	1.738360258271
C	4.367755396107	0.941241064214	0.717032724862
H	2.670432508100	3.686852068887	-0.483548212272
H	1.578527122100	1.018581549863	2.731791928513
H	4.797506408179	2.361037282617	-0.878331376882
H	3.695316638351	-0.337236050782	2.345222469723
H	5.283035300395	0.356259763158	0.541015757048

50

^{6}**B-1.Ph** E_{tot}(UPBE0-D3(Acetonitrile)/def2TZVP // UPBE-D3(Acetonitrile)/def2SVP/W06) = -2578.02038976 $\langle S^2 \rangle$ = 8.7618

Fe	-0.481231551793	-0.921658966013	-0.340540271168
O	-1.682460155849	0.382886603861	0.424730786792
O	1.133226798403	-0.394803456118	0.575543133798
N	0.462887172776	-2.818641303400	-0.587770984649
N	-2.109340988976	-2.301560552424	-0.125008767778
C	-3.362391848721	-1.965164629888	0.004542881217
H	-4.133038165382	-2.764870658881	-0.062207063689
C	-3.865171403358	-0.634216001529	0.243632752068
C	-5.273433497772	-0.445299593976	0.306761154054
H	-5.926283614670	-1.319974624320	0.153255613783
C	-5.828481874823	0.808075823259	0.553560591958
H	-6.919735620872	0.937426287574	0.595033884222
C	-4.966412874345	1.914092930063	0.748278720720
H	-5.395417056967	2.911203952312	0.937069779487
C	-3.580737287969	1.766640493912	0.704935706273
H	-2.901773372755	2.622429186397	0.833170044806
C	-2.985393294160	0.494444520628	0.452468964611
C	2.330731655099	-0.907343854012	0.705269899174
C	3.357000633275	-0.131902354526	1.321689597522
H	3.091636533705	0.880950082410	1.657242016725
C	4.649782555216	-0.630315193300	1.474346224127
H	5.418025537620	0.002045939788	1.947034701418
C	4.986539364852	-1.931169239640	1.031087455189
H	6.008877900711	-2.316299337927	1.156273392415
C	4.001246159131	-2.717691133439	0.438409128850
H	4.239559017102	-3.737284829823	0.094352856162
C	2.674320870131	-2.239706835325	0.259642723032
C	1.700021932550	-3.126548236355	-0.332201868332
H	2.055757519011	-4.153681663813	-0.570831400902
C	-0.470960063512	-3.794621042300	-1.123762656296
H	-0.059013567530	-4.826867782831	-1.122711352282
H	-0.706213268996	-3.516035123038	-2.175004643821
C	-1.748817594624	-3.708500839848	-0.281521455489
H	-2.571205194807	-4.303439306281	-0.734295152269
H	-1.541028427638	-4.130110124676	0.727151916641
O	-0.686913350862	3.708846068946	0.721889386829
N	-0.308196017694	3.192135590159	-0.335829186247
O	-1.055651859177	2.863195478847	-1.256960942621
H	-0.439392117061	-0.511890264936	-1.982576056996
C	1.138559547107	2.962586157730	-0.506325569951
C	2.035540335865	3.626331257278	0.344672869362
C	1.568360338942	2.093445496493	-1.519770083903
C	3.409334107262	3.425415629542	0.155114921415
C	2.944399574076	1.887270626384	-1.681805614009
C	3.862841474091	2.556560652655	-0.854038765817
H	1.647623813400	4.289808586260	1.128828955637
H	0.824716010231	1.571565937985	-2.137264045944

H	4.130291716020	3.945498233936	0.803013677030
H	3.301970432725	1.192052007672	-2.455708802064
H	4.942326800036	2.391765111067	-0.989063072312

50

^{6}**B-2-ts.Ph** E_{tot}(UPBE0-D3(Acetonitrile)/def2TZVP // UPBE-D3(Acetonitrile)/def2SVP/W06) = -2578.00559579 $\langle S^2 \rangle$ = 8.8339

Fe	-0.492407157324	-0.840687644463	-0.157576002396
O	1.163627692671	-0.199843097109	-0.884012127819
O	-1.809585696158	0.355865679959	-0.872335529862
N	-2.094293811119	-2.042989775416	0.511988142966
N	0.396467251199	-2.767935393482	-0.143723550622
C	1.678981914776	-2.997638850647	-0.202249038701
H	2.041062889154	-4.027666269635	0.010745210234
C	2.699400771566	-2.034098384503	-0.540333820485
C	4.053429437935	-2.467366280475	-0.560826232700
H	4.273059383501	-3.512284113662	-0.287965086589
C	5.088077079575	-1.606361234860	-0.920969408700
H	6.129589347397	-1.958998351154	-0.929370166241
C	4.779456917949	-0.274210499433	-1.283335124188
H	5.588979509051	0.413492504583	-1.574964151879
C	3.462950081880	0.185235603856	-1.275201299091
H	3.214419872052	1.220793710603	-1.548127421570
C	2.387526176828	-0.667815236662	-0.893035022714
C	-3.099562252711	0.494509787889	-0.689870194468
C	-3.750228261465	1.666355597392	-1.176566246992
H	-3.125900899886	2.416722552262	-1.684527806076
C	-5.122099413408	1.853779430382	-1.011396073154
H	-5.591239511427	2.772643955970	-1.397217890524
C	-5.916371976574	0.881335400587	-0.359238942554
H	-6.997948976715	1.036512443364	-0.236097616479
C	-5.308041185738	-0.274666753808	0.124838652110
H	-5.909774949722	-1.043533686521	0.636085780294
C	-3.910671226257	-0.496529184066	-0.016675904070
C	-3.355548786552	-1.718705643921	0.516465379082
H	-4.085272129877	-2.426460542354	0.968307809786
C	-1.634341446695	-3.304561332967	1.074330523261
H	-2.458152046881	-4.040441476701	1.193456246656
H	-1.202923768908	-3.103362580782	2.080000961157
C	-0.533010645178	-3.851239823531	0.157869683973
H	-0.025596433255	-4.725906280869	0.619017150710
H	-0.994394477354	-4.187994653123	-0.796911266050
O	2.692896741071	0.689087995374	2.128359204272
N	1.572217483270	1.235892482009	1.999727887287
O	0.515085876769	0.757955267045	2.502655336319
H	-0.190153290598	-0.357079862452	1.429339968042
C	1.434962400165	2.374319182182	1.117642882072
C	2.593153924676	3.026461093486	0.655279555719
C	0.150699116071	2.788684210753	0.718066519537
C	2.456586500557	4.110044186663	-0.221900665867
C	0.032269157777	3.864054779154	-0.172549739495
C	1.179712406941	4.529284199269	-0.641330803278
H	3.574642363695	2.662778875519	0.985964254225
H	-0.726294666316	2.243732866309	1.089257902757
H	3.355337385289	4.630028548940	-0.586856357948
H	-0.967559507665	4.183450817136	-0.504559009207
H	1.079788467920	5.376930864220	-1.335972945478

50

^{6}**B-3.Ph** E_{tot}(UPBE0-D3(Acetonitrile)/def2TZVP // UPBE-D3(Acetonitrile)/def2SVP/W06) = -2578.05578066 $\langle S^2 \rangle$ = 8.8087

Fe	-0.045020329587	-0.881213730361	-0.358353028170
O	1.460955998736	0.137417350159	-1.050347381998
O	-1.625975169972	0.019086761082	-1.021438185113
N	-1.338684923361	-2.307133116396	0.497390005570
N	1.299819698457	-2.427566676430	0.053243476357
C	2.607043360794	-2.352011918050	0.016853546772
H	3.199125644734	-3.242179246302	0.326020961263
C	3.371828963755	-1.206012549983	-0.403829026990
C	4.792342866455	-1.276761497990	-0.320763753301
H	5.244067124323	-2.199562293778	0.079600206562
C	5.605871571968	-0.224320041182	-0.729035200090
H	6.700443597420	-0.301843523352	-0.652659702376
C	5.003541014232	0.948403628941	-1.251230624526
H	5.637175556741	1.787128535702	-1.581466307819
C	3.619611132814	1.054491334308	-1.352057138664
H	3.142464401142	1.962201084814	-1.751921243621
C	2.753092928952	-0.002866405086	-0.928749126954

C	-2.909688725741	-0.151233529835	-0.848343706892
C	-3.825299802327	0.794040293145	-1.407537310802
H	-3.394507414148	1.638361945089	-1.966759511656
C	-5.202983805792	0.658872380716	-1.247755699149
H	-5.875838192783	1.410239520664	-1.691157515957
C	-5.745807867708	-0.430617394248	-0.523699468548
H	-6.834234408590	-0.533141653077	-0.402826150823
C	-4.880572653680	-1.368385679333	0.034379184273
H	-5.285252131970	-2.222242878628	0.602738442918
C	-3.467631321427	-1.261792830240	-0.101488577602
C	-2.644280601598	-2.272727263132	0.518922140033
H	-3.191904876869	-3.076437533513	1.060475407975
C	-0.623876097168	-3.363456677569	1.200761663976
H	-1.244649294165	-4.276002841822	1.340436258324
H	-0.347456630755	-2.994820670071	2.215342973699
C	0.654125664834	-3.682541218901	0.418936216145
H	1.325170324181	-4.347488208375	1.006440981595
H	0.378386514517	-4.223664367879	-0.514055066460
O	2.192281124514	1.152882260745	1.817023962850
N	0.962844716187	1.267414195985	1.720831372546
O	0.173017155502	-0.011015291473	1.681028488072
H	0.718213436582	-0.582729963389	2.267813969723
C	0.262864260571	2.323396148285	1.119537900022
C	1.010378541170	3.436174350977	0.661610131592
C	-1.151514637653	2.327374736894	1.056759226078
C	0.333769713901	4.538910675100	0.129748361055
C	-1.803449166094	3.440471491885	0.518072611387
C	-1.071836638032	4.550355212080	0.052249325930
H	2.106347847991	3.402749503544	0.723480421545
H	-1.717962607131	1.459103270211	1.413963547687
H	0.913735593619	5.400105411345	-0.235816083300
H	-2.902300627430	3.436359990036	0.456610802458
H	-1.595756810708	5.420871961255	-0.369516753594

50

^{6}B-4H-ts.Ph E_{tot}(UPBE0-D3(Acetonitrile)/def2TZVP // UPBE-D3(Acetonitrile)/def2SVP/W06) = -2578.01091574 $\langle S^2 \rangle$ = 8.8131

Fe	-0.307716993239	-0.750240897032	-0.259206096493
O	1.314369001781	0.017621363071	-0.982510804518
O	-1.770002302339	0.319265066123	-0.928848375298
N	-1.767177598762	-2.046301493316	0.553702419159
N	0.798392584322	-2.550654478655	-0.041098107041
C	2.091671240414	-2.687991713844	-0.170101453515
H	2.543949675935	-3.681181270635	0.046522346961
C	3.013411235719	-1.655608012175	-0.570500175057
C	4.403421431302	-1.961549138303	-0.588907650665
H	4.718049639721	-2.975578617435	-0.292057597812
C	5.354837012682	-1.018690522069	-0.967559552530
H	6.424434739947	-1.274553314004	-0.971651794379
C	4.924983518491	0.275560509235	-1.352066474193
H	5.668593330331	1.029005291332	-1.657237408828
C	3.573332413192	0.611002826653	-1.350691444258
H	3.230766777467	1.614581932849	-1.643243671018
C	2.574064465094	-0.330889006661	-0.956620814086
C	-3.069742889031	0.285793856273	-0.781910027140
C	-3.863437647032	1.335483800314	-1.336779364973
H	-3.332523182548	2.140917310092	-1.866601583648
C	-5.251561375362	1.341933099582	-1.211132944676
H	-5.830341477927	2.169639991660	-1.651033322035
C	-5.924125971758	0.300162701706	-0.528201959928
H	-7.019911409009	0.310483890233	-0.435535976778
C	-5.176984097963	-0.736627060616	0.025754309887
H	-5.682742563167	-1.555845698460	0.562773464186
C	-3.758482618382	-0.774189547471	-0.075525720895
C	-3.060139170285	-1.881988378725	0.533615167400
H	-3.702239708583	-2.647260850214	1.024356971110
C	-1.177273649987	-3.205516424890	1.206208079595
H	-1.917425506807	-4.016092233965	1.384807138600
H	-0.776308169328	-2.886466407401	2.194492315372
C	-0.019646572202	-3.699413205770	0.332140991758
H	0.570074508333	-4.483655057210	0.855138956387
H	-0.438134958550	-4.152336050447	-0.594507835405
O	2.082434183760	0.346244026049	2.146213588898
N	0.949274869004	1.002288618910	1.884377677519
O	0.038610942673	-0.054844172488	1.753753219570
H	1.130790517991	-0.558831537191	2.087724677911
C	0.860530671331	2.193806963632	1.183057914663
C	2.034036923303	2.962753053465	0.981846748676

C	-0.404958007020	2.681918808098	0.770082404636
C	1.929606021872	4.209011097313	0.354136286176
C	-0.478724018329	3.925174124071	0.137366860589
C	0.680911352539	4.698578450271	-0.073907359430
H	3.000501944274	2.555440491539	1.306816785845
H	-1.300466967595	2.065912097309	0.923430441215
H	2.840970042790	4.803558227420	0.187297501572
H	-1.459096727039	4.295800447789	-0.199656960119
H	0.610596117490	5.676808208412	-0.572033829179

50

^{6}B-4-ts.Ph E_{tot}(UPBE0-D3(Acetonitrile)/def2TZVP // UPBE-D3(Acetonitrile)/def2SVP/W06) = -2578.05409279 $\langle S^2 \rangle$ = 8.8070

Fe	-0.069806575135	-0.864406595158	-0.345788808628
O	1.445499752132	0.133923370919	-1.048163134718
O	-1.638903451392	0.059315758881	-1.002569142739
N	-1.382801058969	-2.295605801011	0.477769675333
N	1.254826059687	-2.438977062447	0.031867545651
C	2.562534662910	-2.377528616005	-0.004003253427
H	3.144416544436	-3.277993867879	0.294620850583
C	3.340546705807	-1.236418290484	-0.413076953925
C	4.760082469065	-1.324432212941	-0.330390985516
H	5.200989179439	-2.255232463661	0.063431260546
C	5.585752448161	-0.278886016668	-0.731749300064
H	6.679337200878	-0.369512556410	-0.655837009127
C	4.997063592536	0.903613289403	-1.247607245093
H	5.640602729575	1.736413106894	-1.573673569211
C	3.614568555097	1.026847675702	-1.347694198521
H	3.148333693052	1.941886802935	-1.743399644866
C	2.735794318645	-0.022276844724	-0.928843283558
C	-2.924787986759	-0.098010412070	-0.833379165770
C	-3.827483020741	0.869076621845	-1.375747149677
H	-3.385327052830	1.718331546391	-1.918347376076
C	-5.207048387119	0.747543546450	-1.221044256655
H	-5.869811079671	1.515459888485	-1.651086885610
C	-5.764563575420	-0.349060594832	-0.519436230883
H	-6.854392321081	-0.440666472030	-0.402631191317
C	-4.911875206383	-1.307994396336	0.021933847332
H	-5.328026951536	-2.167662083081	0.572945937384
C	-3.497597169142	-1.215735054343	-0.109081912891
C	-2.687595888499	-2.247753047318	0.494521269621
H	-3.246150671076	-3.055634582873	1.018489782770
C	-0.680738925424	-3.371519276947	1.163843582838
H	-1.311728918251	-4.279276669351	1.287829214626
H	-0.401566947375	-3.022595580537	2.184595845321
C	0.594695562301	-3.691938128090	0.378285919460
H	1.257810032217	-4.373299409737	0.955861139374
H	0.314068467310	-4.215456103842	-0.563261246336
O	2.211755267064	1.067553874517	1.839087446029
N	0.986443302095	1.229445936782	1.774608172092
O	0.147495608181	-0.046300383578	1.683914703080
H	0.659736247437	-0.637556093668	2.279902375608
C	0.326790341180	2.297925220136	1.138605865056
C	1.114922796134	3.368406338806	0.653172827628
C	-1.085656330979	2.356160972783	1.082770795155
C	0.479852485810	4.486025428257	0.100085105358
C	-1.697014539429	3.481662363266	0.522168328963
C	-0.924257540298	4.551129860721	0.029028447846
H	2.209035359349	3.291617299543	0.710048416078
H	-1.682459292576	1.517221277770	1.460840312831
H	1.091265599984	5.315309761363	-0.287442769563
H	-2.795439820687	3.519755983547	0.464804769607
H	-1.415688777387	5.432448651373	-0.409230232685

50

^{6}B-5H.Ph E_{tot}(UPBE0-D3(Acetonitrile)/def2TZVP // UPBE-D3(Acetonitrile)/def2SVP/W06) = -2578.06758295 $\langle S^2 \rangle$ = 8.7654

Fe	-0.611465202398	-0.460789189571	-0.037210940652
O	0.923071997070	-0.117317393932	-1.155553519063
O	-1.928103139577	0.719350854797	-0.831923450173
N	-2.254677392732	-1.489651425842	0.826343249150
N	0.121178057003	-2.463929344897	0.090278741755
C	1.342114510653	-2.835474294943	-0.173038049300
H	1.650051591207	-3.870655856425	0.095095595499
C	2.358265349947	-2.027696095308	-0.803601217370
C	3.655756876692	-2.582573893991	-0.969591005506
H	3.836526989487	-3.607929745787	-0.607460856131
C	4.687727441320	-1.860240550108	-1.566102046809

```
H    5.687157136663   -2.304597617081   -1.680259457540
C    4.431304071426   -0.544945752850   -2.020581475705
H    5.240587637246    0.038851335125   -2.487302803809
C    3.166805211690    0.026880037858   -1.885089399001
H    2.961946434324    1.052788167974   -2.225897610093
C    2.094338438990   -0.687076017561   -1.274046866111
C   -3.231083526564    0.822219218432   -0.770677675974
C   -3.881982971579    1.878824943618   -1.476496858347
H   -3.245059080853    2.569450152799   -2.049346382290
C   -5.267304020255    2.031712088865   -1.442199873594
H   -5.732340535643    2.860366158666   -1.999559999258
C   -6.080336730992    1.139091234116   -0.704631427757
H   -7.172399695590    1.266188315594   -0.683620838011
C   -5.474732604171    0.097117340027   -0.005763942111
H   -6.089893536767   -0.609526142638    0.575003655504
C   -4.065273461103   -0.088642771628   -0.015621957767
C   -3.517824805684   -1.197965952116    0.731273687824
H   -4.255825688589   -1.843788680583    1.257447554006
C   -1.792544807353   -2.645636571815    1.575342500349
H   -2.625641519540   -3.298007064394    1.916582654405
H   -1.244105792383   -2.279080167431    2.471525273900
C   -0.818845874873   -3.415789753944    0.674843065360
H   -0.303347653996   -4.223316771832    1.238776208129
H   -1.395586312266   -3.892173541377   -0.149071163570
O    1.938723878846   -0.460286026812    2.001180929793
N    1.131751100281    0.719401016537    1.998846595256
O   -0.128227244543    0.478047808982    1.612048917227
H    1.711867156795   -0.874561875826    2.859492524959
C    1.801038529355    1.780966583548    1.343959028856
C    3.200938500112    1.932036515274    1.481384582887
C    1.058463609687    2.735258633643    0.611188555449
C    3.841682544920    3.027800412564    0.890276143682
C    1.718067958567    3.826254705095    0.028307741071
C    3.108827698412    3.984330775265    0.162489556776
H    3.767713318028    1.173766637188    2.037373440862
H   -0.022928493029    2.589948895628    0.487712101072
H    4.932917794497    3.132271759886    0.995313988746
H    1.133509816016    4.559874875296   -0.548817604839
H    3.619047775352    4.842841424941   -0.299543351385
```

50

^{6}B-5.Ph E_{tot}(UPBE0-D3(Acetonitrile)/def2TZVP // UPBE-D3(Acetonitrile)/def2SVP/W06) = -2578.08686803 $\langle S^2 \rangle$ = 8.7630

```
Fe  -0.317886540873   -0.876408940757    0.186154287926
O    1.285932569873   -0.127642773198   -0.651201647028
O   -1.633378475459    0.170504610505   -0.804530693724
N   -1.893122027474   -2.263981039952    0.501942497583
N    0.683258621957   -2.772280388138   -0.004520066232
C    1.961130357184   -2.940847405986   -0.184721071364
H    2.376206762749   -3.970734210626   -0.111103132565
C    2.916881750789   -1.908060918487   -0.513737212129
C    4.282827743266   -2.274347999841   -0.649262811964
H    4.564476629485   -3.322530374225   -0.457082070554
C    5.255388010480   -1.346658205661   -1.020540808917
H    6.307925133509   -1.650115497188   -1.116854331587
C    4.866879264710   -0.012590155096   -1.281757355400
H    5.624386949686    0.728016884706   -1.583519913128
C    3.534069534914    0.382160917007   -1.165864127269
H    3.223146566426    1.416107994627   -1.376111689940
C    2.519502419629   -0.540640004594   -0.767299619944
C   -2.936082940451    0.235830975392   -0.720050120885
C   -3.616348267457    1.379406391507   -1.239919554972
H   -2.997841720856    2.176159395829   -1.679840518508
C   -5.005601076231    1.484387785389   -1.189803843576
H   -5.493277752879    2.384748871155   -1.596287335830
C   -5.794651660007    0.452745986373   -0.627716161527
H   -6.890073849006    0.543066830511   -0.594421110482
C   -5.161244682872   -0.678921413961   -0.118730976456
H   -5.757824695974   -1.495380870995    0.320275237449
C   -3.746422076415   -0.816765632863   -0.141860007309
C   -3.166600344984   -2.026162324602    0.397165352363
H   -3.886502656427   -2.800881479742    0.743960723279
C   -1.380718429251   -3.504145225402    1.058431223485
H   -2.148937914944   -4.306530401926    1.099916210510
H   -1.035518565302   -3.299570364483    2.096485604143
C   -0.181715252439   -3.930688020251    0.205317402654
H    0.361769445292   -4.781100403759    0.670960160344
H   -0.552709558960   -4.269609556880   -0.787711898583
```

O	3.057650365841	1.495210066953	1.622722070860
N	1.832820840054	1.575888277320	1.644514319987
O	-0.207219686356	-0.326747215748	1.948424302321
H	0.601226508442	0.250214174976	2.065828656389
C	1.245857716052	2.698152925454	0.980563890953
C	2.017391169090	3.705127302588	0.356755638321
C	-0.165652863683	2.749486786619	0.981005156525
C	1.363566716401	4.765991232505	-0.272441046966
C	-0.809700835711	3.818076899165	0.345984436834
C	-0.047675950955	4.821869386210	-0.279148367744
H	3.113759737529	3.623339153798	0.388836925922
H	-0.717173241773	1.924873109621	1.459234343270
H	1.945058020413	5.561019445486	-0.763448558926
H	-1.908793130505	3.866502585078	0.329488232969
H	-0.555314649535	5.660596032238	-0.779706627320

50

^{6}C-1.Ph E_{tot}(UPBE0-D3(Acetonitrile)/def2TZVP // UPBE-D3(Acetonitrile)/def2SVP/W06) = -2578.00784261 $\langle S^2 \rangle$ = 8.7637

Fe	0.712611073352	-0.537342087200	0.151727517060
O	1.930921802684	0.488808126483	-0.965731132127
O	-0.336090827797	-1.373050750653	-1.275805195109
N	0.063189549716	-2.227551528690	1.289130185084
N	2.554969487420	-1.308104663068	1.039936509189
C	3.725983456749	-0.743806143773	0.975149622841
H	4.541241908076	-1.133857490615	1.624891035919
C	4.088366708532	0.356012658336	0.113702353648
C	5.410590100261	0.871315606947	0.190229659110
H	6.102283722769	0.428480528298	0.925507746873
C	5.838627453437	1.907402073226	-0.637383709012
H	6.864684335538	2.295734491754	-0.562001150975
C	4.933500653837	2.449768953595	-1.580767490416
H	5.260666823155	3.267176065939	-2.242954935006
C	3.630686360994	1.965259990440	-1.687033268649
H	2.922648660242	2.383186029360	-2.418379220537
C	3.158475602316	0.913576451658	-0.843106423860
C	-1.428595651927	-2.086281275562	-1.250803864662
C	-2.277143732352	-2.123743505999	-2.399607451890
H	-1.980024118128	-1.515465425733	-3.267124817682
C	-3.441037368161	-2.890665219393	-2.416703633816
H	-4.077201536889	-2.886367619848	-3.316161978173
C	-3.814511481496	-3.673845829018	-1.298248723919
H	-4.733188992052	-4.277903571843	-1.322743139706
C	-2.997624123564	-3.672103369009	-0.168765847622
H	-3.265301262959	-4.283481415201	0.708572276277
C	-1.814382224616	-2.889808606990	-0.109951608602
C	-0.984600382981	-2.965092031309	1.068016700940
H	-1.268867265138	-3.732010018215	1.823010332370
C	0.922715701492	-2.454010409599	2.436935080144
H	0.687847825945	-3.402510320382	2.966602456802
H	0.795186017211	-1.609301574740	3.148213298929
C	2.363335628856	-2.461088403363	1.914466411917
H	3.096151281693	-2.479808380675	2.750486885769
H	2.515295509675	-3.383344620312	1.310036358796
O	-2.199149419625	0.190159612003	0.840847271650
N	-1.789444514297	1.218044922832	0.290201263731
O	-0.693587184463	1.264056967530	-0.322499361647
H	0.479783109936	0.342030837222	1.576146756364
C	-2.547759724033	2.453846863470	0.412451208215
C	-3.804245484274	2.410501495655	1.042779262623
C	-2.012724262902	3.647801255038	-0.103985260948
C	-4.537874833960	3.597531313529	1.157605082824
C	-2.759496704332	4.825560139147	0.023948836065
C	-4.019005277118	4.803272354392	0.650875249136
H	-4.180300607521	1.454138942777	1.429175726665
H	-1.027954307141	3.635337206719	-0.588642066533
H	-5.522918910290	3.581757008847	1.647040210303
H	-2.354616594656	5.769700154375	-0.369526510869
H	-4.600742621430	5.732464510541	0.744753818292

50

^{6}C-2-ts.Ph E_{tot}(UPBE0-D3(Acetonitrile)/def2TZVP // UPBE-D3(Acetonitrile)/def2SVP/W06) = -2577.99272033 $\langle S^2 \rangle$ = 8.9389

Fe	0.475249316481	-0.815854598920	-0.007232797595
O	1.993989515658	-0.577063799391	-1.159136480431
O	-0.749848745601	-1.827791517325	-1.110185503761
N	-0.749828602186	-1.357086474196	1.614971726563
N	1.915057751404	-1.218991052475	1.535253813126

C	3.188986918446	-0.950846004607	1.481452300355
H	3.795793058423	-1.073717959103	2.405723450570
C	3.908537494558	-0.501185504180	0.314644305377
C	5.298323958071	-0.228886315664	0.440411985776
H	5.770460084161	-0.363343946267	1.427207521789
C	6.058717180863	0.197053954473	-0.646211940213
H	7.132335061695	0.404166021051	-0.529276341536
C	5.430619121041	0.357724951489	-1.904332043679
H	6.022461609044	0.692661494669	-2.770845417896
C	4.070838136881	0.096993978115	-2.065467769716
H	3.576186713988	0.219097956454	-3.040585342649
C	3.264948575901	-0.335147710956	-0.969680730161
C	-1.984909255744	-2.214057730824	-0.948033875958
C	-2.735622926134	-2.665732133090	-2.076141593155
H	-2.235053198329	-2.649327548503	-3.055854643825
C	-4.053555276485	-3.096883668576	-1.943586232702
H	-4.606093719640	-3.427299931770	-2.837538012094
C	-4.690470810503	-3.118123579432	-0.678621106588
H	-5.730089433812	-3.464006494920	-0.584323509672
C	-3.978793154472	-2.700543727636	0.442966566900
H	-4.453335637784	-2.718741161935	1.437660280742
C	-2.637920293023	-2.238709007647	0.343586765555
C	-1.948651589522	-1.864089288910	1.551001653918
H	-2.494896316324	-2.048050506293	2.502382721414
C	-0.098355540869	-1.132066467569	2.895645542056
H	-0.644542877121	-1.606757386050	3.738811220730
H	-0.045802409192	-0.036981546677	3.083733047706
C	1.323895617834	-1.694165960826	2.781672748559
H	1.937165011216	-1.422060930290	3.667770316855
H	1.262857652500	-2.804288592321	2.735501691742
O	-2.330908042328	0.836666602314	-0.432267495118
N	-1.380422788247	1.656884154859	-0.475870627037
O	-0.134254140560	1.254496152494	-0.616060026740
H	0.421358766122	0.645295907246	0.715841943392
C	-1.566710096321	3.036048073239	-0.175159193874
C	-2.885555347033	3.518106748530	-0.015191261460
C	-0.458241072162	3.904029102106	-0.051684051714
C	-3.087074240374	4.873616459117	0.267617873539
C	-0.682823927073	5.256084009879	0.236600166546
C	-1.991557893177	5.749086147521	0.395112763696
H	-3.721289821723	2.812911909280	-0.113094855198
H	0.555894599339	3.505573511784	-0.182387454418
H	-4.112970921646	5.252476832790	0.392863229087
H	0.177721916120	5.934906656873	0.338190986677
H	-2.157869073936	6.813761436528	0.617366325543

50

^{6}C-3.Ph E_{tot}(UPBE0-D3(Acetonitrile)/def2TZVP // UPBE-D3(Acetonitrile)/def2SVP/W06) = -2578.06024372 $\langle S^2 \rangle$ = 8.8124

Fe	0.710633956705	-0.542870119160	-0.654221820432
O	2.072076792786	0.784416293551	-1.080257946071
O	-0.909127764319	0.036799107458	-1.547524046254
N	-0.429629134215	-2.088103449732	0.189091703503
N	2.226859827866	-1.749394845302	0.104637375901
C	3.484219033350	-1.422705022532	0.273339112550
H	4.169743073341	-2.165372676852	0.738906414401
C	4.079266635615	-0.161196284525	-0.095313141129
C	5.457110814776	0.042707956459	0.202386410454
H	6.005475683838	-0.771360895422	0.704982338941
C	6.113608620581	1.226900672954	-0.121443629641
H	7.178304970461	1.359941720404	0.120784902356
C	5.389323762372	2.258263896141	-0.768652091505
H	5.895951517365	3.200842726327	-1.031017993792
C	4.041282566598	2.095051111922	-1.079901826087
H	3.472299863401	2.891182873599	-1.584257398427
C	3.334621117282	0.892986013654	-0.760704678936
C	-2.161068434095	-0.333210494328	-1.493001960172
C	-3.140603496431	0.382560822993	-2.248851373810
H	-2.783922384711	1.224664425506	-2.860941472560
C	-4.491257691431	0.045774045156	-2.199198861135
H	-5.217051197110	0.627900189862	-2.789348237180
C	-4.941224442004	-1.029682548060	-1.394043118936
H	-6.009607684843	-1.288742409970	-1.356130099642
C	-4.010918698130	-1.754836809852	-0.654392066992
H	-4.342322240819	-2.597338115558	-0.024875680516
C	-2.622345396439	-1.441937257549	-0.679989937176
C	-1.726352754208	-2.245145512463	0.114275676187
H	-2.201842344892	-3.057992050740	0.707367563587

C	0.351787878250	-2.943979153311	1.072178524818
H	-0.116232200048	-3.943280638341	1.213485292121
H	0.405841201484	-2.457437781623	2.072073084059
C	1.761895488494	-3.076535375697	0.489842689174
H	2.447443951200	-3.569340320441	1.214305295189
H	1.718512527835	-3.715288562531	-0.420790537426
O	-0.816968134613	-0.402805538447	2.988126040649
N	-0.678971547405	0.472404681270	2.117148220885
O	0.591536003325	0.554945847704	1.441312792145
H	1.203336822535	0.120652809694	2.079854432154
C	-1.655464872414	1.303496201159	1.571916186086
C	-2.990459119639	1.130679220396	2.019952171369
C	-1.330883875577	2.306720532285	0.623493407835
C	-3.991065479830	1.962435931026	1.511802717416
C	-2.353432770865	3.122410907860	0.131967418580
C	-3.682813475234	2.961698187538	0.568274981758
H	-3.209433474937	0.340773060093	2.750412029857
H	-0.298386082436	2.418390387058	0.271413883013
H	-5.028624465281	1.824254843828	1.851546341473
H	-2.107932377454	3.897126586969	-0.609996537151
H	-4.477339700536	3.609114293150	0.168978220766

50

^{6}C-4a-ts.Ph E_{tot}(UPBE0-D3(Acetonitrile)/def2TZVP // UPBE-D3(Acetonitrile)/def2SVP/W06) = -2578.04331923 $\langle S^2 \rangle$ = 8.8004

Fe	0.510082039009	-0.691440498392	0.224392107210
O	2.099312550114	-1.108488629041	-0.775388452113
O	-0.857832238279	-1.916185833620	-0.310130457476
N	-0.714399973598	0.141474856877	1.625924328473
N	1.963950300724	0.720966244157	1.374545390868
C	3.275705743455	0.492558940960	1.386102407653
H	3.831879722522	0.741177494373	2.312349708103
C	4.018291678755	-0.094879806857	0.319629584563
C	5.444648396168	0.005546297234	0.347222561842
H	5.908189177120	0.593629226821	1.155537228275
C	6.236682823828	-0.594567095074	-0.620912257425
H	7.330627927975	-0.485787126857	-0.594016745871
C	5.616929758825	-1.360032164732	-1.644341930503
H	6.237951331276	-1.840729157361	-2.416744667222
C	4.236691268702	-1.522945410349	-1.679682431869
H	3.752504038009	-2.131275420623	-2.458263256659
C	3.382182233854	-0.899432357955	-0.715695002316
C	-2.115097597381	-2.017580261724	0.043378058662
C	-2.953697277624	-2.962969143711	-0.620037328366
H	-2.494989352181	-3.574646754258	-1.411471527605
C	-4.299147494857	-3.100742160227	-0.285759187600
H	-4.917738230292	-3.838059707094	-0.821550432535
C	-4.877896517643	-2.304301683182	0.732810374028
H	-5.940832852852	-2.417409717978	0.991612731764
C	-4.084040266328	-1.376485622233	1.400040547251
H	-4.519619282319	-0.746654612586	2.192872301107
C	-2.705903809644	-1.201733738635	1.083173709231
C	-1.971343043052	-0.196440124636	1.805499540767
H	-2.550453064656	0.354342743015	2.576916757926
C	-0.193122879929	1.273601735037	2.396890027764
H	-0.716856878101	1.379079357614	3.370703053877
H	-0.372999397235	2.212256496969	1.828179353204
C	1.305631583074	1.115028025445	2.630048261750
H	1.729682128274	2.066150134337	3.017697415137
H	1.493470665515	0.326903609962	3.388464060275
O	0.280554631141	3.420783026285	-0.019187913503
N	-0.059383106637	2.374727968688	-0.632132027601
O	0.866771893730	1.373515266987	-0.784294973776
H	1.476576887198	1.221232354662	0.283260878419
C	-1.365179002933	2.110457322046	-1.066574021194
C	-2.381622958788	3.052440810486	-0.759511833074
C	-1.682168508176	0.929069530358	-1.785288631990
C	-3.701407469303	2.784098396570	-1.133767780256
C	-3.011206186999	0.682687092924	-2.145017355977
C	-4.030256220190	1.598258471389	-1.820521818323
H	-2.107742736391	3.966685386237	-0.216896936777
H	-0.894545076173	0.209981960586	-2.040915033947
H	-4.489440552724	3.509975834758	-0.880341276583
H	-3.254267392405	-0.246564103752	-2.682385298010
H	-5.073200836750	1.390168507614	-2.101665535276

50

^{6}C-4b-ts.Ph E_{tot}(UPBE0-D3(Acetonitrile)/def2TZVP // UPBE-D3(Acetonitrile)/def2SVP/W06) = -2578.00843704 $\langle S^2 \rangle$ = 8.7853

Fe	0.623300083882	-0.576993150176	0.096331783665
O	2.238632198895	-1.187497622733	-0.771687699525
O	-0.764686532074	-1.787325569818	-0.463535361508
N	-0.774263436884	0.185490542010	1.499066150111
N	1.900738526219	0.263096671365	1.588791437636
C	3.196350139138	0.395800313395	1.503748335625
H	3.728019315999	0.935862644027	2.318308804615
C	4.031260400951	-0.102553544241	0.438391670283
C	5.426251865480	0.172156084495	0.494047347167
H	5.806941235021	0.768993995958	1.338951523888
C	6.302675288460	-0.292874138845	-0.482829898103
H	7.377358281507	-0.067761556501	-0.422073538156
C	5.790337552451	-1.063443870038	-1.554655548920
H	6.474023289173	-1.438949090110	-2.332519621075
C	4.430865543575	-1.356516732190	-1.640486563464
H	4.026807599975	-1.956031470628	-2.470059446050
C	3.504897032344	-0.888749211586	-0.658317690342
C	-2.008595510818	-1.998046075277	-0.109246203840
C	-2.774935674211	-2.980553243252	-0.804175441864
H	-2.276279830125	-3.527282486596	-1.618463141870
C	-4.105503081315	-3.231206551699	-0.471783276159
H	-4.668356543702	-3.993120505986	-1.034027068209
C	-4.739186120337	-2.521418724160	0.575907580649
H	-5.788882513651	-2.725603144681	0.832271172789
C	-4.013780879130	-1.559885163878	1.275208298789
H	-4.491007075193	-0.992041359272	2.090449737584
C	-2.658968271167	-1.269970506380	0.957357250802
C	-1.989252397914	-0.235504929827	1.707906493323
H	-2.584658299958	0.239574848274	2.518741848576
C	-0.199289235345	1.233998616645	2.328851466905
H	-0.832740784580	1.474194845038	3.210338531129
H	-0.094236811896	2.149422550544	1.703947590758
C	1.194340278965	0.766449716853	2.763007907647
H	1.752574275501	1.583437913172	3.269754640912
H	1.082244402606	-0.069406631724	3.489409316306
O	0.458591326200	3.220094920489	-0.377187801267
N	-0.172448868966	2.302856692494	-1.148883302498
O	0.717795898933	1.224662472249	-1.028545470556
C	-1.552719430104	2.097457661050	-1.094255960601
C	-2.119237105189	0.945060386127	-1.688847483623
C	-2.377997550449	3.096831516398	-0.526929139950
C	-3.508705044706	0.787619437170	-1.681145359905
C	-3.767229265812	2.920432333842	-0.539915797523
C	-4.341460272105	1.769576825687	-1.111258336359
H	-1.465260742038	0.172917279434	-2.116009253637
H	-1.908129446176	3.980932455283	-0.075618062403
H	-3.946246683444	-0.124509683219	-2.114670141612
H	-4.410725758339	3.690296561366	-0.086681185300
H	-5.433178562683	1.634583767327	-1.107876922575
H	1.244376216276	2.138768828553	-0.427448398079

50

^{6}C-4-ts.Ph E_{tot}(UPBE0-D3(Acetonitrile)/def2TZVP // UPBE-D3(Acetonitrile)/def2SVP/W06) = -2578.04504121 $\langle S^2 \rangle$ = 8.7981

Fe	-0.660597370068	0.404832050144	-0.439304194580
O	-2.051289579034	-0.817974783540	-1.007126233057
O	0.934205122141	-0.169206441111	-1.380557716458
N	0.462423384316	2.094051023838	0.171296987262
N	-2.200445546242	1.757212064056	0.081366122084
C	-3.473563021247	1.479797205702	0.164682077538
H	-4.168944941730	2.268793974566	0.528431698750
C	-4.084223055381	0.218039513882	-0.183356601395
C	-5.482940371569	0.069614947020	0.031709344209
H	-6.035902339540	0.919428513709	0.464827039247
C	-6.154749794071	-1.107699887269	-0.288779531812
H	-7.236176817802	-1.200162895267	-0.111134827492
C	-5.424240749245	-2.182808409185	-0.849332328042
H	-5.942626986089	-3.119691744836	-1.108681636878
C	-4.054337051463	-2.071796316220	-1.081218518242
H	-3.480773283925	-2.902499170431	-1.519802234203
C	-3.334236141808	-0.880215610731	-0.760109680361
C	2.170144857590	0.247835108704	-1.431497049773
C	3.137475888786	-0.506948471559	-2.163901757607
H	2.788199486057	-1.425694238577	-2.658002712836
C	4.470507070139	-0.110815588762	-2.231481075006
H	5.189248791051	-0.724852130150	-2.797419679388
C	4.914280895951	1.065425159981	-1.578121962077

```
H          5.969625302966       1.370132949711      -1.634022746834
C          3.993130447898       1.830926664788      -0.868087118634
H          4.317829054825       2.752310291082      -0.356921097450
C          2.623031673966       1.457857636592      -0.775001584781
C          1.735229984353       2.305641815950      -0.019235459218
H          2.200448145523       3.206885880519       0.439545076185
C         -0.321093717716       2.998877690793       0.997790127880
H          0.145651110154       4.004435994272       1.089261949344
H         -0.383600264576       2.563020797629       2.019869494056
C         -1.725558212197       3.100920753604       0.395020653319
H         -2.416561512315       3.642211116431       1.077844155719
H         -1.666906583086       3.681120149858      -0.553219563413
O          1.067434850982       0.509537516836       3.022365258545
N          0.737064485727      -0.531622390684       2.436856596431
O         -0.614324094741      -0.377062895527       1.548449673084
C          1.650816587407      -1.312404992373       1.676132679643
C          1.197266627298      -2.409251965504       0.910292096819
C          3.031688687693      -1.053209970897       1.818650769982
C          2.135634704061      -3.232929402365       0.281482350798
C          3.955956198491      -1.897867427183       1.191213185423
C          3.515912278383      -2.987483439796       0.420891509197
H          0.118772176329      -2.583821018278       0.799533566874
H          3.350256258330      -0.189754476839       2.418285923455
H          1.785985167813      -4.079198976726      -0.329236359790
H          5.032252114964      -1.691020635157       1.292943070689
H          4.245823031100      -3.643489158924      -0.076448479869
H         -1.175375673587       0.097349328906       2.198117858414
```

50

^{6}C-5a.Ph E_{tot}(UPBE0-D3(Acetonitrile)/def2TZVP // UPBE-D3(Acetonitrile)/def2SVP/W06) = -2578.05171957 $\langle S^2 \rangle$ = 8.8062

```
Fe         0.487093019039      -0.342614763503      -0.203701475691
O          2.225282310228      -0.483727589619      -1.042352026724
O         -0.629592835440      -1.837052244003      -0.605235483978
N         -0.590763028179       0.126981582816       1.470044644053
N          2.116418577500       0.982357963417       1.413316645460
C          3.346190914173       0.461533466442       1.494940387412
H          3.753837404050       0.372884167362       2.517656727101
C          4.115349731239      -0.051720345735       0.419442641799
C          5.520270512233      -0.248277471470       0.623703465040
H          5.957037801953       0.071564752137       1.583052689475
C          6.326328911315      -0.794355318213      -0.361649133714
H          7.406884365718      -0.912924369654      -0.195865610867
C          5.740167509853      -1.206896935418      -1.590226221301
H          6.376852093500      -1.638852746316      -2.378377415449
C          4.372944578504      -1.090218653875      -1.805071124546
H          3.908912642018      -1.434190134384      -2.741538768783
C          3.503293841913      -0.517028571279      -0.821842384152
C         -1.783003457465      -2.189402109394      -0.090041974200
C         -2.534018502690      -3.228772035168      -0.715382935174
H         -2.104872559470      -3.684741641618      -1.620259756015
C         -3.761592937278      -3.646857482979      -0.205685334545
H         -4.315161063163      -4.450022608605      -0.717370476932
C         -4.301663019901      -3.052135320303       0.960428103411
H         -5.270599435783      -3.386530335130       1.358949751386
C         -3.590887411917      -2.036705994701       1.593065312170
H         -3.999819904770      -1.559984127272       2.498806240673
C         -2.338005025973      -1.573248241935       1.096978354515
C         -1.700278901444      -0.491215362379       1.803785170612
H         -2.233628945618      -0.138837592818       2.712403464251
C         -0.180672359591       1.280329951933       2.271111260260
H         -0.758395626982       1.345353852995       3.218027538910
H         -0.391173640719       2.210005283670       1.698714736336
C          1.309484586243       1.251905091162       2.609512313845
H          1.596558577379       2.227475601770       3.057099064606
H          1.527514763161       0.455534661345       3.347161970983
O         -0.147015004448       3.518958511341      -0.069375589014
N         -0.485783589837       2.452074306972      -0.663105545043
O          0.473990143970       1.562741623044      -0.976324475290
H          1.795253764340       1.427505937905       0.523637665527
C         -1.823065602889       2.133246755149      -0.931023846161
C         -2.843460406897       2.998081782428      -0.448897654260
C         -2.174716163571       0.959996534573      -1.651632293461
C         -4.184381495494       2.663463831261      -0.652946376563
C         -3.524708173446       0.644197774534      -1.837147270972
C         -4.540287321002       1.483630684491      -1.339485293672
H         -2.547715204967       3.907605505447       0.089978203301
H         -1.391094202703       0.303462761990      -2.050554346474
```

H	-4.969263568024	3.331603325924	-0.265230493412
H	-3.786751889058	-0.277353662001	-2.379637460864
H	-5.598953332236	1.223853665059	-1.487515965067

50

^{6}**C-5b.Ph** E_{tot}(UPBE0-D3(Acetonitrile)/def2TZVP // UPBE-D3(Acetonitrile)/def2SVP/W06) = -2578.06821615 $\langle S^2 \rangle$ = 8.7628

Fe	0.617179086744	-0.474896496016	0.090960246894
O	2.193833712716	-1.307355959111	-0.694366247906
O	-0.724152272251	-1.770295614440	-0.410031252394
N	-0.741158768603	0.132474211241	1.616438969007
N	1.922961723059	0.107941989272	1.679175976394
C	3.199346136841	0.316101426669	1.522161986592
H	3.753282874230	0.844473501878	2.329778162978
C	3.989852130983	-0.100390939523	0.388453793977
C	5.362145877244	0.269988534423	0.350439707443
H	5.757814645488	0.893350301159	1.168927489047
C	6.199473498443	-0.135521809104	-0.686273576977
H	7.256886252237	0.165780722920	-0.701616776490
C	5.669463342248	-0.948325641937	-1.716630519545
H	6.322113869071	-1.280000641150	-2.539848435018
C	4.331413135646	-1.339551892801	-1.707290394920
H	3.915803409355	-1.972984616299	-2.505253068053
C	3.442839383422	-0.924767445035	-0.669292623289
C	-1.983461497973	-1.958305367520	-0.105226780488
C	-2.748912107091	-2.906519480935	-0.846333948170
H	-2.237999025042	-3.446903780773	-1.657116276137
C	-4.095365723839	-3.131399144043	-0.561471233651
H	-4.657453803631	-3.866167535686	-1.159505831581
C	-4.745908679882	-2.432796094225	0.483022098587
H	-5.807567183421	-2.618128145067	0.701299859822
C	-4.019175680555	-1.510333487036	1.232002194559
H	-4.505678532292	-0.957196045489	2.051762945569
C	-2.649305499789	-1.245857351198	0.961457972154
C	-1.964300355921	-0.280443575208	1.785446537066
H	-2.549078780741	0.132884846971	2.636814924455
C	-0.129900545315	1.061181055729	2.557058373159
H	-0.747418758499	1.207192120614	3.469404351384
H	-0.006482482014	2.036609594470	2.040203756030
C	1.256285322896	0.509376428031	2.912200531246
H	1.849344857844	1.253858399300	3.486883188026
H	1.134391046252	-0.394632152159	3.549629127789
O	0.365384824254	3.015614117729	0.010992021711
N	-0.073355516588	2.187725733422	-1.069040651079
O	0.636040702331	1.050662620573	-1.150067898159
C	-1.482751699972	2.072770679099	-1.079547811908
C	-2.076301881113	0.957151640550	-1.715475274732
C	-2.304639365465	3.091266694710	-0.541322367722
C	-3.471749578683	0.858298841915	-1.788517271066
C	-3.698629320004	2.973757501616	-0.625313720785
C	-4.294609562716	1.859934989901	-1.245208539796
H	-1.431933093987	0.167927543926	-2.122609193961
H	-1.836419561396	3.954187258013	-0.051073741488
H	-3.919338721686	-0.028080883272	-2.264370243031
H	-4.329242978834	3.765509333870	-0.191257038789
H	-5.390018264954	1.772043125201	-1.299875510520
H	1.139506198776	3.477897707993	-0.371411945947

50

^{6}**C-5.Ph** E_{tot}(UPBE0-D3(Acetonitrile)/def2TZVP // UPBE-D3(Acetonitrile)/def2SVP/W06) = -2578.08336070 $\langle S^2 \rangle$ = 8.7707

Fe	0.736369398534	-0.404009489240	-0.058161373876
O	2.381014467900	-1.418381984532	-0.385911522962
O	-0.545259592973	-1.774797452498	-0.527202105141
N	-0.750376456783	0.295921980388	1.303081850793
N	1.887869597630	0.602197105766	1.443025667765
C	3.174417722679	0.792456495620	1.356812884054
H	3.652300667741	1.523070345597	2.046921643831
C	4.064149862148	0.107461393748	0.451116722840
C	5.433173310960	0.493379150513	0.426080575251
H	5.749788173163	1.335635391858	1.062785299027
C	6.362266644194	-0.164989862464	-0.374708180088
H	7.415224164748	0.151825207257	-0.386003694001
C	5.932394726563	-1.256309857661	-1.168549199906
H	6.659366064467	-1.788830809575	-1.802294914167
C	4.602192217458	-1.670559269266	-1.160938365619
H	4.264347878326	-2.519213409766	-1.774495695405
C	3.618923211395	-1.002975649439	-0.367309219720

C	-1.732952577669	-2.125842913126	-0.106288259967
C	-2.386783856000	-3.242849178599	-0.708800099688
H	-1.849881127798	-3.770187893713	-1.511543186549
C	-3.656879834437	-3.648656070323	-0.299934939637
H	-4.130086187496	-4.514289936556	-0.790148378336
C	-4.342123468964	-2.965643316976	0.732083762951
H	-5.343284503879	-3.292122039575	1.048742732130
C	-3.729579122071	-1.869225014957	1.336194600423
H	-4.251473521365	-1.313586019406	2.132230767259
C	-2.439617566723	-1.425361070873	0.941866111735
C	-1.901244148270	-0.242922450862	1.573010838647
H	-2.549534560508	0.240893228344	2.336602244141
C	-0.305875205204	1.505587353138	1.973731661295
H	-0.985018488460	1.807151462337	2.800732574652
H	-0.288435665267	2.328711839679	1.225436125601
C	1.115360423891	1.258531735156	2.491697608968
H	1.589208098615	2.204145358456	2.834711046552
H	1.065183156499	0.570418660351	3.364986005047
O	-0.460403960337	3.935448661957	-0.598440774865
N	-0.844262085431	3.148180976072	-1.463463220281
O	0.722662089453	0.874550831074	-1.403653982097
C	-2.036852871889	2.413514349959	-1.150445653615
C	-2.316672107207	1.297811635537	-1.966940205944
C	-2.920627001267	2.801490195467	-0.119691105740
C	-3.479462862531	0.554630434408	-1.738359604592
C	-4.090739205522	2.064857154724	0.088148735073
C	-4.367763982550	0.941232051912	-0.717031160206
H	-1.578537383845	1.018578847767	-2.731792558453
H	-2.670443696811	3.686843962940	0.483551797548
H	-3.695324720903	-0.337242437159	-2.345222734474
H	-4.797515475727	2.361025915208	0.878334861341
H	-5.283043065043	0.356249642719	-0.541014208525
H	1.470608557516	1.502804746083	-1.427652052970

13.4.9 Olefin substrates related to hydroamination reaction

25

cinnamate E_{tot}(RPBE0-D3(Acetonitrile)/def2TZVP // RPBE-D3(Acetonitrile)/def2SVP/W06) = -576.401011 E(LUMO) = -0.0791

C	2.308294274275	-1.140683923774	-0.019677812289
C	1.844750125149	0.200386174699	-0.014721473862
C	3.676132086545	-1.423575215519	0.058993819604
H	4.017862695669	-2.469878207898	0.053924724138
C	2.803519145844	1.241072394217	0.071850868696
C	4.614938425796	-0.377338369859	0.144370550511
H	2.457941166228	2.286952371267	0.076603692089
H	5.690140935394	-0.604817912721	0.205935182817
C	4.173848344354	0.956347600711	0.150457479432
H	4.901797330464	1.779176209801	0.216859595145
H	1.588377991327	-1.970136507173	-0.085977601050
C	0.427702696817	0.554653519784	-0.094640970467
H	0.196362559644	1.634871576889	-0.081561787381
C	-0.636714274096	-0.285320418613	-0.183656114254
H	-0.533237834977	-1.380776900168	-0.205314327591
C	-2.012148131416	0.251997486879	-0.257065497956
O	-2.913359777893	-0.762504057108	-0.345772813297
C	-4.310991501567	-0.393292278048	-0.410386898328
H	-4.412659830249	0.521271408886	-1.029600493610
H	-4.799495920280	-1.238447293910	-0.933299496398
C	-4.898259149620	-0.182318421956	0.976666961968
H	-5.986062718077	0.016369634786	0.898209545228
H	-4.755318379049	-1.081002505721	1.609354832179
H	-4.424543403881	0.684858855803	1.477071026665
O	-2.322589827695	1.438752554488	-0.246145983024

26

trans stilbene E_{tot}(RPBE0-D3(Acetonitrile)/def2TZVP // RPBE-D3(Acetonitrile)/def2SVP/W06) = -540.251987 E(LUMO) = -0.0675

C	2.843486953981	-1.288924334736	-0.000004714088
C	1.943723622057	-0.192029185082	0.000001183485
C	4.231870709149	-1.093257978295	-0.000006193594
H	4.906276150355	-1.963485792079	-0.000011278983
C	2.497441062173	1.115409498527	0.000006616325
C	4.760145083238	0.208445642853	-0.000001201932
H	1.833973043832	1.993402867863	0.000012492139

H	5.849420882254	0.366680175109	-0.000002227143
C	3.883473580461	1.310279034510	0.000005306223
H	4.288032179225	2.334315218608	0.000009528881
H	2.435856246619	-2.312618828439	-0.000008378298
C	-3.883475434614	-1.310278018530	0.000005070111
C	-4.760145506956	-0.208443561104	0.000000215670
C	-2.497442625647	-1.115410302562	0.000004542785
H	-5.849421451856	-0.366676752955	0.000000673926
H	-1.833975584192	-1.993404537555	0.000008094617
C	-4.231869671238	1.093259418005	-0.000005088492
C	-1.943723352457	0.192027853605	-0.000000764970
H	-4.906274004369	1.963488026822	-0.000008881968
C	-2.843485629493	1.288924204200	-0.000005614238
H	-2.435853600452	2.312618375687	-0.000010272915
H	-4.288035244437	-2.334313639224	0.000009514859
C	0.502427544683	-0.460029381027	0.000001696042
H	0.238914308514	-1.531997503753	0.000004154922
C	-0.502426374758	0.460026671899	-0.000000918151
H	-0.238912688973	1.531995016317	-0.000004231097

19

α-methyl styrene E_{tot}(RPBE0-D3(Acetonitrile)/def2TZVP // RPBE-D3(Acetonitrile)/def2SVP/W06) = -348.663872 E(LUMO) = -0.0425

C	2.633694144669	-0.074135778420	0.006073411088
C	1.868400522789	-1.240644084722	0.159037354126
C	1.978700080064	1.159800512265	-0.158185310572
H	2.365431279963	-2.215287111938	0.283253576227
H	2.565123182292	2.081430351674	-0.296659489931
C	0.465836005663	-1.175037304753	0.152727401263
C	0.579421196024	1.223825680042	-0.161609779043
H	-0.107399471150	-2.104805722503	0.278591053682
H	0.089572194806	2.196202434772	-0.320576399068
C	-0.212795476783	0.059838788004	0.006444843054
H	3.733312554342	-0.125006309574	0.004864422691
C	-1.703325389247	0.122547766681	0.017437377742
C	-2.461154605047	-1.152128835377	-0.275167250197
H	-2.309508332713	-1.908137907501	0.524707134512
H	-3.548617412947	-0.962778090770	-0.350798558958
H	-2.118111129111	-1.622647348467	-1.220440108087
C	-2.372156359180	1.269257806263	0.295604224584
H	-1.856508384724	2.204999064985	0.559986561092
H	-3.473015194466	1.296083339417	0.282898175567

20

dihydronaphthalene E_{tot}(RPBE0-D3(Acetonitrile)/def2TZVP // RPBE-D3(Acetonitrile)/def2SVP/W06) = -386.755316 E(LUMO) = -0.0394

C	-2.504442632775	-0.727934303410	0.102235176962
C	-2.534754172895	0.676446456174	0.095350618273
C	-1.273235971194	-1.402306372983	0.001561612973
H	-3.494606245547	1.210699189954	0.168717262364
H	-1.251071933883	-2.504350169458	-0.003562401669
C	-1.335116014677	1.398477631770	-0.002545263523
C	-0.065838458993	-0.693237084298	-0.100267999273
H	-1.351908792994	2.500393791761	-0.000752861330
C	1.268172523090	-1.382643391452	-0.300177795206
C	-0.093265396652	0.731726528846	-0.092508543891
C	1.175760349210	1.468744701978	-0.154599502164
H	-3.440241948295	-1.302312236225	0.183078540338
C	2.365188531786	0.852765164067	0.058098382867
C	2.432957312100	-0.624634829508	0.353255876194
H	1.130515693708	2.557673074607	-0.321572184779
H	3.297965353349	1.440332283166	0.071679992930
H	2.414167672895	-0.767920496681	1.461151169528
H	3.401273232291	-1.048620743891	0.017509920760
H	1.458089250145	-1.440019777797	-1.398233963449
H	1.223261304324	-2.430301922544	0.059569146030

17

indene E_{tot}(RPBE0-D3(Acetonitrile)/def2TZVP // RPBE-D3(Acetonitrile)/def2SVP/W06) = -347.471292 E(LUMO) = -0.0334

C	1.007041888799	1.425813671935	0.000003556401
C	-0.216534056816	0.730721623038	0.000003417875
C	-0.236622827391	-0.693570574636	0.000005268057
C	0.957464379548	-1.421522548909	0.000001506879
C	2.181601133888	-0.721729823760	-0.000004082218
C	2.203752928150	0.686869569347	-0.000001987995
C	-1.604284637462	1.206294320476	-0.000000655103
C	-2.451009567765	0.138866694136	-0.000003363009

C	-1.674427664832	-1.154052351362	0.000001324308
H	1.027843885180	2.526880751542	0.000004156448
H	0.946397479624	-2.523049090811	0.000000446237
H	3.129723215774	-1.281464761930	-0.000009470775
H	3.170230236288	1.214696402333	-0.000004219029
H	-1.896635762029	2.266450197080	-0.000007137985
H	-3.549269459733	0.184972009885	-0.000012663292
H	-1.915091268770	-1.787316248581	0.884446313932
H	-1.915087783044	-1.787312741102	-0.884447336699

23

4,7-dimethyl indene E_{tot}(RPBE0-D3(Acetonitrile)/def2TZVP // RPBE-D3(Acetonitrile)/def2SVP/W06) = -426.038865 E(LUMO) = -0.0328

C	1.504692813461	-0.706130722465	0.000000334408
C	0.700693854972	0.458570089407	-0.000000019467
C	-0.717517162866	0.379478531730	-0.000000078051
C	-1.392166753953	-0.853892238187	-0.000000373127
C	-0.585237233091	-2.013596283965	0.000000145461
C	0.820835670786	-1.938992053494	0.000000938237
C	1.076448376799	1.878150955633	-0.000000494839
C	-0.047529137039	2.648883503985	0.000000385876
C	-1.279255583267	1.779989022487	0.000000028108
H	-1.071209573691	-3.002800354112	0.000000101258
H	1.406489823995	-2.872860386762	0.000002043853
H	2.111279228554	2.249544716378	-0.000000579889
H	-0.079375589669	3.747605350664	0.000000457896
H	-1.931729688901	1.974338573824	0.882986303543
H	-1.931735241827	1.974340635501	-0.882981437867
C	3.010460557296	-0.625076876164	-0.000000443461
H	3.389689109619	-0.078107306915	0.889296198920
H	3.471930035574	-1.631651297858	0.000008784139
H	3.389688192634	-0.078125533262	-0.889309028602
C	-2.898081891409	-0.922536138691	-0.000000568120
H	-3.326679796945	-0.411546110928	-0.888593595004
H	-3.261727468184	-1.968280737327	0.000000375753
H	-3.326680101295	-0.411544290855	0.888591245839

15

benzofuran E_{tot}(RPBE0-D3(Acetonitrile)/def2TZVP // RPBE-D3(Acetonitrile)/def2SVP/W06) = -383.374546 E(LUMO) = -0.0329

C	0.973187284406	1.449467605280	0.000004685553
C	-0.262208001052	0.763729773088	-0.000010717056
C	-0.255655884023	-0.657036209743	-0.000005860757
C	0.912224155924	-1.427081600522	0.000005034145
C	2.125792595027	-0.721512338247	-0.000002774171
C	2.154094881123	0.694935677413	0.000002315735
C	-1.658285469426	1.139692547244	0.000000669303
C	-2.363382790988	-0.035440280155	0.000003905468
O	-1.539423822123	-1.136072264795	0.000000788171
H	1.005251868520	2.549500566308	-0.000000405449
H	0.873359285324	-2.525742203196	0.000006325610
H	3.073320728828	-1.281408096167	-0.000006627133
H	3.126494913589	1.210733148438	0.000006435812
H	-2.080798633265	2.150623533204	0.000004973631
H	-3.436838211953	-0.255659876375	-0.000000557166

20

2-methyl indene E_{tot}(RPBE0-D3(Acetonitrile)/def2TZVP // RPBE-D3(Acetonitrile)/def2SVP/W06) = -386.757661 E(LUMO) = -0.0299

C	1.451100621100	-1.438474159409	0.000007556151
C	0.242772377895	-0.717105624216	0.000003349533
C	0.257272696888	0.707466987230	-0.000003639223
C	1.466437994567	1.408076368019	-0.000004542580
C	2.676162822041	0.681466501649	-0.000002498516
C	2.665109403204	-0.726574062051	0.000000785122
C	-1.153514302041	-1.158755669382	-0.000009295387
C	-1.990442189702	-0.077616139316	-0.000000618730
C	-1.170187181414	1.197071211947	0.000006624395
H	1.448025561252	-2.539829901519	0.000007995623
H	1.479616176440	2.509702822973	-0.000002911165
H	3.636503009055	1.219963280634	-0.000002854533
H	3.618943591132	-1.277163209036	0.000000048356
H	-1.473976362343	-2.211300353894	-0.000015309015
H	-1.400501542756	1.832701998879	-0.885245304586
H	-1.400494243114	1.832693265588	0.885267144424
C	-3.484426014978	-0.073854348539	0.000001069221
H	-3.885750997417	0.461918688489	0.887843788597
H	-3.885749273228	0.461866021801	-0.887874750954

```
H          -3.898333284380      -1.100759009508       0.000029413335
```

23

2-allylanisole E_{tot}(RPBE0-D3(Acetonitrile)/def2TZVP // RPBE-D3(Acetonitrile)/def2SVP/W06) = -463.104853 E(LUMO) = -0.0190

```
C           2.411241429205      -1.400672503625       0.176877595363
C           1.106800884773      -1.744395490315      -0.223112945256
C           2.720559258461      -0.054885370009       0.417880301142
H           0.853641693664      -2.799289125824      -0.418406588940
H           3.735747194956       0.234891057278       0.730786986846
C           0.104619981417      -0.774313595377      -0.387691143021
C           1.741332872976       0.941970769416       0.260009558988
H           2.003261971699       1.991610016054       0.449877807346
C           0.435290495433       0.587938686171      -0.138155987674
H           3.177842416451      -2.180945402093       0.295960499824
C          -1.314033038682      -1.145866388476      -0.776254715541
H          -1.292954033650      -2.140255154785      -1.271944188752
H          -1.711206035529      -0.412622228849      -1.507510617822
C          -2.231701252363      -1.210914819666       0.423047539396
H          -1.966562157570      -1.973999573602       1.178732380352
C          -3.295678291522      -0.416295549389       0.634329061569
H          -3.919250518563      -0.516094251062       1.537164184256
H          -3.578703011325       0.365871182334      -0.090428021364
O          -0.575343514119       1.482888307141      -0.321658631652
C          -0.323434694049       2.861545764770      -0.091447845766
H          -0.017245387170       3.055265063189       0.960253635918
H           0.462124960598       3.258531826642      -0.771854168108
H          -1.273934854501       3.389261112587      -0.292251371545
```

19

allylbenzene E_{tot}(RPBE0-D3(Acetonitrile)/def2TZVP // RPBE-D3(Acetonitrile)/def2SVP/W06) = -348.657247 E(LUMO) = -0.0185

```
C           2.321012776450       0.886777615792      -0.242012350829
C           0.997699949839       1.288757266288       0.009967653078
C           2.659881746949      -0.475823875117      -0.221572262877
H           0.738592539371       2.359888130367      -0.005588823225
H           3.695771013131      -0.792802320168      -0.417935489082
C          -0.006604194003       0.339675321841       0.287522387374
C           1.666099671101      -1.433014342184       0.050891562403
H           1.922318820384      -2.503904831891       0.068988236705
C           0.346027666293      -1.027356738070       0.299364785129
H          -0.431078145703      -1.781519324743       0.504222194535
H           3.091115690708       1.644413204298      -0.455499885315
C          -1.443346179800       0.761758327692       0.555780113124
H          -1.488016622665       1.872041677456       0.586075675766
H          -1.760836784772       0.399412326227       1.557464967980
C          -2.403040768106       0.249181343981      -0.488799160813
H          -2.202865403441       0.585057315974      -1.523362610147
C          -3.438135304932      -0.577344586415      -0.253376722980
H          -4.099054229096      -0.919337112453      -1.065685955276
H          -3.663519060668      -0.938911067913       0.764725666402
```

18

1-hexene E_{tot}(RPBE0-D3(Acetonitrile)/def2TZVP // RPBE-D3(Acetonitrile)/def2SVP/W06) = -235.635805 E(LUMO) = 0.0076

```
C          -0.775792391489      -0.586636089019       0.224268529907
H          -0.640019046807      -1.251417462765       1.109049040692
C          -2.045303521368       0.200489366857       0.392065743463
H          -2.083892045504       0.862419348567       1.278979636987
H          -0.858923353102      -1.255961277408      -0.659899413389
C          -3.099719373822       0.177090249834      -0.443935737317
H          -3.104393518847      -0.463049382637      -1.343364830659
H          -3.996730608237       0.790441281491      -0.261513378141
C           3.021698125139       0.384390899521      -0.169836714591
H           2.958245011246       1.031396239055      -1.070129183859
H           3.140282167069       1.053476003613       0.708505522731
C           1.779751187936      -0.498555911028      -0.031920205607
H           1.706617187162      -1.182494449245      -0.907083561197
H           1.885750531397      -1.160388702946       0.856857295828
H           3.947522731847      -0.219869148667      -0.257198392910
C           0.476578464197       0.298870679986       0.091163636322
H           0.541912575358       0.979564681268       0.970334872266
H           0.360353424859       0.961987692767      -0.795369121410
```

16

cyclohexene E_{tot}(RPBE0-D3(Acetonitrile)/def2TZVP // RPBE-D3(Acetonitrile)/def2SVP/W06) = -234.443874 E(LUMO) = 0.0165

```
C           0.698215645199       1.193175155813       0.318312421540
C           1.500152815075      -0.046516219423      -0.108616120849
```

C	0.673028101341	-1.307663691951	-0.057424963447
C	-0.672803054849	-1.307768285705	0.057417051027
C	-1.500137277913	-0.046745067635	0.108623809261
C	-0.698426465245	1.193056771177	-0.318318106262
H	1.251230502436	2.118870932806	0.055874048195
H	0.590373486720	1.194432627470	1.426132519620
H	2.403781740661	-0.164793589268	0.529029974255
H	1.898173400203	0.089486735516	-1.142354051011
H	1.211576122695	-2.270016823877	-0.115120375671
H	-1.211223060291	-2.270188970570	0.115108976990
H	-2.403774784236	-0.165199970434	-0.528974960009
H	-1.898154203197	0.089169272645	1.142382252136
H	-1.251579092875	2.118677367055	-0.055901188768
H	-0.590582693765	1.194330444999	-1.426141743358

19

1-methylcyclohexene E_{tot}(RPBE0-D3(Acetonitrile)/def2TZVP // RPBE-D3(Acetonitrile)/def2SVP/W06) = -273.728952 E(LUMO) = 0.0199

C	1.151821751672	1.219803613805	-0.308742000850
C	-0.332672888020	1.266337625517	0.078706401418
C	-1.015974959359	-0.085412386734	0.003382342608
C	-0.299949261193	-1.231906269927	-0.090140346972
C	1.207237649144	-1.295069240721	-0.101322644971
C	1.851933621967	0.024304346142	0.347532353957
H	1.650392143043	2.172022080694	-0.031895536419
H	1.239821883167	1.129012486265	-1.414651779033
H	-0.879789372718	1.989227543478	-0.566998713502
H	-0.449294478859	1.666641126095	1.114029756257
H	-0.843744726191	-2.191467539502	-0.161878771025
H	1.551770452018	-2.134766255283	0.541854784268
H	1.558917119194	-1.560952170300	-1.127055214856
H	2.936966521883	0.023306182754	0.113994080010
H	1.766880527175	0.117144399369	1.453505805137
C	-2.520919280161	-0.073241927281	0.050872934907
H	-2.945031520648	0.502221309571	-0.801594930308
H	-2.949529987442	-1.094757972978	0.027214971522
H	-2.886218364917	0.433474245034	0.971741307373

14 References

[1] Gallagher, K. J.; Webster, R. L., Room temperature hydrophosphination using a simple iron salen pre-catalyst. *Chem. Commun.* **2014**, *50* (81), 12 109–12 111.

[2] Timelthaler, D.; Schofberger, W.; Topf, C., Selective and Additive-Free Hydrogenation of Nitroarenes Mediated by a DMSO-Tagged Molecular Cobalt Corrole Catalyst. *Eur. J. Org. Chem.* **2021**, *2021* (14), 2114–2120.

[3] Panda, S.; Nanda, A.; Behera, R. R.; Ghosh, R.; Bagh, B., Cobalt catalyzed chemoselective reduction of nitroarenes: hydrosilylation under thermal and photochemical reaction conditions. *Chem. Commun.* **2023**, *59* (30), 4527–4530.

[4] Jackson, D. M.; Ashley, R. L.; Brownfield, C. B.; Morrison, D. R.; Morrison, R. W., Rapid Conventional and Microwave-Assisted Decarboxylation of L-Histidine and Other Amino Acids via Organocatalysis with R-Carvone Under Superheated Conditions. *Synth. Commun.* **2015**, *45* (23), 2691–2700.

[5] Hosoya, H.; Misal Castro, L. C.; Sultan, I.; Nakajima, Y.; Ohmura, T.; Sato, K.; Tsurugi, H.; Suginome, M.; Mashima, K., 4,4′-Bipyridyl-Catalyzed Reduction of Nitroarenes by Bis(neopentylglycolato)diboron. *Org. Lett.* **2019**, *21* (24), 9812–9817.

[6] Zhao, L.; Hu, C.; Cong, X.; Deng, G.; Liu, L. L.; Luo, M.; Zeng, X., Cyclic (Alkyl)(amino)carbene Ligand-Promoted Nitro Deoxygenative Hydroboration with Chromium Catalysis: Scope, Mechanism, and Applications. *J. Am. Chem. Soc.* **2021**, *143* (3), 1618–1629.

[7] Hood, T. M.; Lau, S.; Diefenbach, M.; Firmstone, L.; Mahon, M.; Krewald, V.; Webster, R. L., The Complex Reactivity of $[(salen)Fe]_2(\mu\text{–}O)$ with HBpin and Its Implications in Catalysis. *ACS Catal.* **2023**, *13* (17), 11 841–11 850.

[8] Provis-Evans, C. B.; Lau, S.; Krewald, V.; Webster, R. L., Regioselective Alkyne Cyclotrimerization with an In Situ-Generated [Fe(II)H(salen)]·Bpin Catalyst. *ACS Catal.* **2020**, *10* (17), 10 157–10 168.

[9] Braunschweig, H.; Guethlein, F.; Mailänder, L.; Marder, T. B., Synthesis of Catechol-, Pinacol-, and Neopentylglycolborane through the Heterogeneous Catalytic B–B Hydrogenolysis of Diboranes(4). *Chem. Eur. J.* **2013**, *19* (44), 14 831–14 835.

[10] Wei, C. S.; Jiménez-Hoyos, C. A.; Videa, M. F.; Hartwig, J. F.; Hall, M. B., Origins of the Selectivity for Borylation of Primary over Secondary C–H Bonds Catalyzed by Cp^*-Rhodium Complexes. *J. Am. Chem. Soc.* **2010**, *132* (9), 3078–3091.

[11] Zhu, S.; Xu, W.; Hong, D.; Wu, W.; Chai, F.; Zhu, X.; Zhou, S.; Wang, S., Rare-Earth Metal Complexes Supported by 1,3-Functionalized Indolyl-Based Ligands for Efficient Hydrosilylation of Alkenes. *Inorg. Chem.* **2023**, *62* (1), 381–391.

[12] Evans, R.; Deng, Z.; Rogerson, A. K.; McLachlan, A. S.; Richards, J. J.; Nilsson, M.; Morris, G. A., Quantitative Interpretation of Diffusion-Ordered NMR Spectra: Can We Rationalize Small Molecule Diffusion Coefficients?. *Angew. Chem. Int. Ed.* **2013**, *52* (11), 3199–3202.

[13] Wu, X.; Ding, G.; Lu, W.; Yang, L.; Wang, J.; Zhang, Y.; Xie, X.; Zhang, Z., Nickel-Catalyzed Hydrosilylation of Terminal Alkenes with Primary Silanes via Electrophilic Silicon–Hydrogen Bond Activation. *Org. Lett.* **2021**, *23* (4), 1434–1439.

[14] Tseberlidis, G.; Zardi, P.; Caselli, A.; Cancogni, D.; Fusari, M.; Lay, L.; Gallo, E., Glycoporphyrin Catalysts for Efficient C–H Bond Aminations by Organic Azides. *Organometallics* **2015**, *34* (15), 3774–3781.

[15] Fang, Z. X.; Zhang, Y. J.; Guo, Y. S.; Jin, Q. H.; Zhu, H. Y.; Xiu, H. S.; Liu, Z. H.; Wang, Y., The [4+1] cyclization reaction of 2-hydroxylimides and trimethylsulfoxonium iodide for the synthesis of 3-amino-2,3-dihydrobenzofurans. *New J. Chem.* **2022**, *46* (38), 18 124–18 127.

[16] Werkmeister, S.; Junge, K.; Beller, M., Copper-catalyzed reductive amination of aromatic and aliphatic ketones with anilines using environmental-friendly molecular hydrogen. *Green Chem.* **2012**, *14* (9), 2371–2374.

[17] Xu, S.; Guo, H.; Liu, Y.; Chang, W.; Feng, J.; He, X.; Zhang, Z., Rh(I)-Catalyzed Coupling of Azides with Boronic Acids Under Neutral Conditions. *Org. Lett.* **2022**, *24* (30), 5546–5551.

[18] Song, G.; Yang, L.; Li, J. S.; Tang, W. J.; Zhang, W.; Cao, R.; Wang, C.; Xiao, J.; Xue, D., Chiral Arylated Amines via C–N Coupling of Chiral Amines with Aryl Bromides Promoted by Light. *Angew. Chem. Int. Ed.* **2021**, *60* (39), 21 536–21 542.

[19] Stoll, S.; Schweiger, A., EasySpin, a comprehensive software package for spectral simulation and analysis in EPR. *J. Magn. Reson.* **2006**, *178* (1), 42–55.

[20] Leedy, D. W.; Adams, R. N., The reduction of N,N-dimethyl-p-nitrosoaniline. *J. Electroanal. Chem.* **1967**, *14* (1), 119–122.

[21] Nelson, R. F.; Adams, R. N., Anion and cation radicals of N,N-dimethyl-p-nitroaniline. *J. Phys. Chem.* **1968**, *72* (2), 740–742.

[22] Cotton, S. A., Some aspects of the coordination chemistry of iron(III). *Coord. Chem. Rev.* **1972**, *8* (3), 185–223.

[23] Domracheva, N.; Pyataev, A.; Manapov, R.; Gruzdev, M.; Chervonova, U.; Kolker, A., Structural, Magnetic and Dynamic Characterization of Liquid Crystalline Iron(III) Schiff Base Complexes with Asymmetric Ligands. *Eur. J. Inorg. Chem.* **2011**, (8), 1219–1229.

[24] Gaffney, B. J., EPR of Mononuclear Non-Heme Iron Proteins. *Biol. Magn. Reson.* **2009**, *28*, 233–268.

[25] Buettner, G. R., Spin Trapping: ESR parameters of spin adducts 1474 1528V. *Free Radic. Biol. Med.* **1987**, *3* (4), 259–303.

[26] Chandra, H.; Davidson, I. M. T.; Symons, M. C. R., Use of Spin Traps in the Study of Silyl Radicals in the Gas-Phase. *J. Chem. Soc., Faraday Trans. 1* **1983**, *79*, 2705–2711.

[27] Meijere, A. d.; Chaplinski, V.; Gerson, F.; Merstetter, P.; Haselbach, E., Radical Cations of Trialkylamines: ESR Spectra and Structures. *J. Org. Chem.* **1999**, *64* (19), 6951–6959.

[28] Gaussian 16, Revision C.01. Frisch, M. J.; Trucks, G. W.; Schlegel, H. B.; Scuseria, G. E.; Robb, M. A.; Cheeseman, J. R.; Scalmani, G.; Barone, V.; Petersson, G. A.; Nakatsuji, H.; Li, X.; Caricato, M.; Marenich, A. V.; Bloino, J.; Janesko, B. G.; Gomperts, R.; Mennucci, B.; Hratchian, H. P.; Ortiz, J. V.; Izmaylov, A. F.; Sonnenberg, J. L.; Williams-Young, D.; Ding, F.; Lipparini, F.; Egidi, F.; Goings, J.; Peng, B.; Petrone, A.; Henderson, T.; Ranasinghe, D.; Zakrzewski, V. G.; Gao, J.; Rega, N.; Zheng, G.; Liang, W.; Hada, M.; Ehara, M.; Toyota, K.; Fukuda, R.; Hasegawa, J.; Ishida, M.; Nakajima, T.; Honda, Y.; Kitao, O.; Nakai, H.; Vreven, T.; Throssell, K.; Montgomery, Jr., J. A.; Peralta, J. E.; Ogliaro, F.; Bearpark, M. J.; Heyd, J. J.; Brothers, E. N.; Kudin, K. N.; Staroverov,

V. N.; Keith, T. A.; Kobayashi, R.; Normand, J.; Raghavachari, K.; Rendell, A. P.; Burant, J. C.; Iyengar, S. S.; Tomasi, J.; Cossi, M.; Millam, J. M.; Klene, M.; Adamo, C.; Cammi, R.; Ochterski, J. W.; Martin, R. L.; Morokuma, K.; Farkas, Ö.; Foresman, J. B.; Fox, D. J. (Gaussian, Inc., Wallingford, CT), **2019**, see `http://www.gaussian.com`.

[29] Neese, F., Software update: The ORCA program system – Version 5.0. *WIREs Comput. Mol. Sci.* **2022**, *12* (5), e1606.

[30] ORCA version 5.0.4, an *ab initio*, DFT and semiempirical SCF-MO package. Neese, F.; *Technical Directorship* F. Wennmohs; *with contributions from* D. Aravena; Atanasov, M.; Auer, A. A.; Becker, U.; Bistoni, G.; Bykov, D.; Chilkuri, V. G.; Datta, D.; Dutta, A. K.; Ehlert, S.; Ganyushin, D.; Garcia, M.; Guo, Y.; Hansen, A.; Helmich-Paris, B.; Huntington, L.; Izsák, R.; Kettner, M.; Kollmar, C.; Kossmann, S.; Krupička, M.; Lang, L.; Lechner, M.; Lenk, D.; Liakos, D. G.; Manganas, D.; Pantazis, D. A.; Papadopoulos, A.; Petrenko, T.; Pinski, P.; Pracht, P.; Reimann, C.; Retegan, M.; Riplinger, C.; Risthaus, T.; Roemelt, M.; Saitow, M.; Sandhöfer, B.; Schapiro, I.; Sen, A.; Sivalingam, K.; de Souza, B.; Stoychev, G.; Van den Heuvel, W.; Wezisla, B.; *and with contributions from collaborators* M. Kállay; Grimme, S.; Valeev, E.; Chan, G.; Pittner, J.; Brehm, M.; Goerigk, L.; Åsgeirsson, V.; Ungur, L. (Max-Planck-Institut für Kohlenforschung, Mülheim a. d. Ruhr, Germany), **2023**, see `https://orcaforum.kofo.mpg.de/`.

[31] Perdew, J. P.; Burke, K.; Ernzerhof, M., Generalized Gradient Approximation Made Simple. *Phys. Rev. Lett.* **1996**, *77* (18), 3865–3868.

[32] Perdew, J. P.; Burke, K.; Ernzerhof, M., Erratum: Generalized Gradient Approximation Made Simple [Phys. Rev. Lett. 77, 3865 (1996)]. *Phys. Rev. Lett.* **1997**, *78* (7), 1396–1396.

[33] Grimme, S.; Antony, J.; Ehrlich, S.; Krieg, H., A consistent and accurate *ab initio* parametrization of density functional dispersion correction (DFT-D) for the 94 elements H–Pu. *J. Chem. Phys.* **2010**, *132* (15), 154 104.

[34] Tomasi, J.; Mennucci, B.; Cammi, R., Quantum Mechanical Continuum Solvation Models. *Chem. Rev.* **2005**, *105* (8), 2999–3094.

[35] Weigend, F.; Ahlrichs, R., Balanced basis sets of split valence, triple zeta valence and quadruple zeta valence quality for H to Rn: Design and assessment of accuracy. *Phys. Chem. Chem. Phys.* **2005**, *7* (18), 3297–3305.

[36] Weigend, F., Accurate Coulomb-fitting basis sets for H to Rn. *Phys. Chem. Chem. Phys.* **2006**, *8* (9), 1057–1065.

[37] Perdew, J. P.; Ernzerhof, M.; Burke, K., Rationale for mixing exact exchange with density functional approximations. *J. Chem. Phys.* **1996**, *105* (22), 9982–9985.

[38] Adamo, C.; Barone, V., Toward reliable density functional methods without adjustable parameters: The PBE0 model. *J. Chem. Phys.* **1999**, *110* (13), 6158–6170.

[39] Riplinger, C.; Pinski, P.; Becker, U.; Valeev, E. F.; Neese, F., Sparse maps — A systematic infrastructure for reduced-scaling electronic structure methods. II. Linear scaling domain based pair natural orbital coupled cluster theory. *J. Chem. Phys.* **2016**, *144* (2), 024 109.

[40] Guo, Y.; Riplinger, C.; Becker, U.; Liakos, D. G.; Minenkov, Y.; Cavallo, L.; Neese, F., Communication: An improved linear scaling perturbative triples correction for the domain based local pair-natural orbital based singles and doubles coupled cluster method [DLPNO-CCSD(T)]. *J. Chem. Phys.* **2018**, *148* (1), 011 101.

[41] Hellweg, A.; Hättig, C.; Höfener, S.; Klopper, W., Optimized accurate auxiliary basis sets for RI-MP2 and RI-CC2 calculations for the atoms Rb to Rn. *Theor. Chem. Acc.* **2007**, *117* (4), 587–597.

[42] Neese, F.; Wennmohs, F.; Hansen, A.; Becker, U., Efficient, approximate and parallel Hartree–Fock and hybrid DFT calculations. A 'chain-of-spheres' algorithm for the Hartree–Fock exchange. *Chem. Phys.* **2009**, *356* (1–3), 98–109.

[43] Neese, F.; Valeev, E. F., Revisiting the Atomic Natural Orbital Approach for Basis Sets: Robust Systematic Basis Sets for Explicitly Correlated and Conventional Correlated *ab initio* Methods?. *J. Chem. Theory Comput.* **2011**, *7* (1), 33–43.

[44] Altun, A.; Neese, F.; Bistoni, G., Extrapolation to the Limit of a Complete Pair Natural Orbital Space in Local Coupled-Cluster Calculations. *J. Chem. Theory Comput.* **2020**, *16* (10), 6142–6149.

[45] Verplancke, H.; Diefenbach, M.; Lienert, J. N.; Ugandi, M.; Kitsaras, M.-P.; Roemelt, M.; Stopkowicz, S.; Holthausen, M. C., Another Torture Track for Quantum Chemistry: Reinvestigation of the Benzaldehyde Amidation by Nitrogen-Atom Transfer from Platinum(II) and Palladium(II) Metallonitrenes. *Isr. J. Chem.* **2023**, *63* (7–8), e202300 060.
